# Global, regional, and national burden of meningitis and its aetiologies, 1990–2019: a systematic analysis for the Global Burden of Disease Study 2019

**DOI:** 10.1016/S1474-4422(23)00195-3

**Published:** 2023-08

**Authors:** GBD 2019 Meningitis Antimicrobial Resistance Collaborators, Han Yong Wunrow, Rose G Bender, Avina Vongpradith, Sarah Brooke Sirota, Lucien R Swetschinski, Amanda Novotney, Authia P Gray, Kevin S Ikuta, Fablina Sharara, Eve E Wool, Amirali Aali, Sherief Abd-Elsalam, Ashkan Abdollahi, Jeza Muhamad Abdul Aziz, Hassan Abidi, Richard Gyan Aboagye, Hassan Abolhassani, Eman Abu-Gharbieh, Lawan Hassan Adamu, Tigist Demssew Adane, Isaac Yeboah Addo, Oyelola A Adegboye, Tayo Alex Adekiya, Mohammad Adnan, Qorinah Estiningtyas Sakilah Adnani, Saira Afzal, Shahin Aghamiri, Zahra Babaei Aghdam, Antonella Agodi, Bright Opoku Ahinkorah, Aqeel Ahmad, Sajjad Ahmad, Mohadese Ahmadzade, Ali Ahmed, Ayman Ahmed, Jivan Qasim Ahmed, Meqdad Saleh Ahmed, Karolina Akinosoglou, Addis Aklilu, Maxwell Akonde, Fares Alahdab, Tareq Mohammed Ali AL-Ahdal, Fahad Mashhour Alanezi, Ahmed Hassan Albelbeisi, Tsegaye Begashaw B Alemayehu, Kefyalew Addis Alene, Ayman Al-Eyadhy, Adel Ali Saeed Al-Gheethi, Abid Ali, Beriwan Abdulqadir Ali, Liaqat Ali, Syed Shujait Ali, Yousef Alimohamadi, Vahid Alipour, Syed Mohamed Aljunid, Sami Almustanyir, Rajaa M Al-Raddadi, Nelson Alvis-Guzman, Yaser Mohammed Al-Worafi, Hany Aly, Edward Kwabena Ameyaw, Robert Ancuceanu, Adnan Ansar, Golnoosh Ansari, Anayochukwu Edward Anyasodor, Jalal Arabloo, Aleksandr Y Aravkin, Demelash Areda, Anton A Artamonov, Judie Arulappan, Raphael Taiwo Aruleba, Muhammad Asaduzzaman, Kendalem Asmare Atalell, Seyyed Shamsadin Athari, Daniel Atlaw, Maha Moh'd Wahbi Atout, Sameh Attia, Tewachew Awoke, Melese Kitu Ayalew, Tegegn Mulatu Ayana, Alemu Degu Ayele, Sina Azadnajafabad, Khalil Azizian, Muhammad Badar, Ashish D Badiye, Nayereh Baghcheghi, Mahboube Bagheri, Sara Bagherieh, Saeed Bahadory, Atif Amin Baig, Aleksandra Barac, Shirin Barati, Mainak Bardhan, Zarrin Basharat, Azadeh Bashiri, Buddha Basnyat, Quique Bassat, Saurav Basu, Nebiyou Simegnew Bayileyegn, Neeraj Bedi, Amir Hossein Behnoush, Abebe Ayalew Bekel, Melaku Ashagrie Belete, Olorunjuwon Omolaja Bello, Akshaya Srikanth Bhagavathula, Dinesh Bhandari, Pankaj Bhardwaj, Sonu Bhaskar, Ajay Nagesh Bhat, Ali Bijani, Niloufar Bineshfar, Archith Boloor, Souad Bouaoud, Danilo Buonsenso, Katrin Burkart, Luis Alberto Cámera, Carlos A Castañeda-Orjuela, Achille Cernigliaro, Jaykaran Charan, Vijay Kumar Chattu, Patrick R Ching, Hitesh Chopra, Sonali Gajanan Choudhari, Devasahayam J Christopher, Dinh-Toi Chu, Rosa A S Couto, Natália Cruz-Martins, Omid Dadras, Xiaochen Dai, Lalit Dandona, Rakhi Dandona, Saswati Das, Nihar Ranjan Dash, Mohsen Dashti, Fernando Pio De la Hoz, Sisay Abebe Debela, Demeke Dejen, Hiwot Dejene, Dessalegn Demeke, Feleke Mekonnen Demeke, Berecha Hundessa Demessa, Andreas K Demetriades, Solomon Demissie, Diriba Dereje, Emina Dervišević, Hardik Dineshbhai Desai, Anteneh Mengist Dessie, Fikreab Desta, Kuldeep Dhama, Shirin Djalalinia, Thanh Chi Do, Masoud Dodangeh, Milad Dodangeh, Regina-Mae Villanueva Dominguez, Deepa Dongarwar, Haneil Larson Dsouza, Oyewole Christopher Durojaiye, Arkadiusz Marian Dziedzic, Martin Herbas Ekat, Michael Ekholuenetale, Temitope Cyrus Ekundayo, Maysaa El Sayed Zaki, Hassan El-Abid, Muhammed Elhadi, Victor Gabriel El-Hajj, Waseem El-Huneidi, Amro A El-Sakka, Hawi Leul Esayas, Adeniyi Francis Fagbamigbe, Shahab Falahi, Jawad Fares, Ali Fatehizadeh, Syeda Anum Fatima Fatima, Nicholas A Feasey, Ginenus Fekadu, Getahun Fetensa, Desalegn Feyissa, Florian Fischer, Behzad Foroutan, Peter Andras Gaal, Muktar A Gadanya, Abduzhappar Gaipov, Balasankar Ganesan, Mesfin Gebrehiwot, Kahsu Gebrekirstos Gebrekidan, Teferi Gebru Gebremeskel, Getachew Muluye Gedef, Yibeltal Yismaw Gela, Urge Gerema, Bradford D Gessner, Motuma Erena Getachew, Keyghobad Ghadiri, Kazem Ghaffari, Seyyed-Hadi Ghamari, Reza Ghanbari, Ramy Mohamed Mohmaed Ghazy, Ghozali Ghozali, Admasu Belay AB Gizaw, Ekaterina Vladimirovna Glushkova, Mohamad Goldust, Mahaveer Golechha, Habtamu Alganeh Guadie, Rashid Abdi Guled, Mohak Gupta, Sapna Gupta, Veer Bala Gupta, Vijai Kumar Gupta, Vivek Kumar Gupta, Najah R Hadi, Arvin Haj-Mirzaian, Sebastian Haller, Samer Hamidi, Shafiul Haque, Harapan Harapan, Ahmed I Hasaballah, Ikramul Hasan, Hamidreza Hasani, Mohammad Hasanian, Hadi Hassankhani, Mohammed Bheser Hassen, Khezar Hayat, Mohammad Heidari, Mahsa Heidari-Foroozan, Reza Heidari-Soureshjani, Kamal Hezam, Ramesh Holla, Nobuyuki Horita, Md Mahbub Hossain, Mohammad-Salar Hosseini, Mehdi Hosseinzadeh, Sorin Hostiuc, Salman Hussain, Nawfal R Hussein, Segun Emmanuel Ibitoye, Olayinka Stephen Ilesanmi, Irena M Ilic, Milena D Ilic, Mohammad Tarique Imam, Kenneth Chukwuemeka Iregbu, Nahlah Elkudssiah Ismail, Chidozie C D Iwu, Chinwe Jaja, Mihajlo Jakovljevic, Elham Jamshidi, Amirreza Javadi Mamaghani, Javad Javidnia, Mohammad Jokar, Nabi Jomehzadeh, Nitin Joseph, Charity Ehimwenma Joshua, Jacek Jerzy Jozwiak, Zubair Kabir, Laleh R Kalankesh, Rohollah Kalhor, Vineet Kumar Kamal, Himal Kandel, Ibraheem M Karaye, André Karch, Hanie Karimi, Harkiran Kaur, Navjot Kaur, Mohammad Keykhaei, Himanshu Khajuria, Amirmohammad Khalaji, Ajmal Khan, Imteyaz A Khan, Maseer Khan, Taimoor Khan, Khaled Khatab, Moawiah Mohammad Khatatbeh, Hamid Reza Khayat Kashani, Jagdish Khubchandani, Min Seo Kim, Adnan Kisa, Sezer Kisa, Farzad Kompani, Hamid Reza Koohestani, Nikhil Kothari, Kewal Krishan, Yuvaraj Krishnamoorthy, Mukhtar Kulimbet, Manoj Kumar, Senthil D Kumaran, Ambily Kuttikkattu, Alexander Kwarteng, Tri Laksono, Iván Landires, Dennis Odai Laryea, Basira Kankia Lawal, Thao Thi Thu Le, Caterina Ledda, Sang-woong Lee, Seung Lee, Gebretsadik Kiros Lema, Miriam Levi, Stephen S Lim, Xuefeng Liu, Graciliana Lopes, Ricardo Lutzky Saute, Pedro Henrique Machado Teixeira, Ata Mahmoodpoor, Mansour Adam Mahmoud, Elaheh Malakan Rad, Kashish Malhotra, Ahmad Azam Malik, Bernardo Alfonso Martinez-Guerra, Miquel Martorell, Vasundhara Mathur, Mahsa Mayeli, John Robert Carabeo Medina, Addisu Melese, Ziad A Memish, Alexios-Fotios A Mentis, Muayad Aghali Merza, Tomislav Mestrovic, Irmina Maria Michalek, Le Huu Nhat Minh, Alireza Mirahmadi, Omid Mirmosayyeb, Awoke Misganaw, Arup Kumar Misra, Javad Moghadasi, Nouh Saad Mohamed, Yousef Mohammad, Esmaeil Mohammadi, Shafiu Mohammed, Maryam Mojarrad Sani, Hoda Mojiri-forushani, Ali H Mokdad, Sara Momtazmanesh, Lorenzo Monasta, Mohammad Ali Moni, Elias Mossialos, Ebrahim Mostafavi, Majid Motaghinejad, Amin Mousavi Khaneghah, Sumaira Mubarik, Lorenzo Muccioli, Jibran Sualeh Muhammad, Francesk Mulita, Temesgen Mulugeta, Efrén Murillo-Zamora, Ghulam Mustafa, Saravanan Muthupandian, Ahamarshan Jayaraman Nagarajan, Firzan Nainu, Tapas Sadasivan Nair, Shumaila Nargus, Hasan Nassereldine, Zuhair S Natto, Biswa Prakash Nayak, Ionut Negoi, Ruxandra Irina Negoi, Seyed Aria Nejadghaderi, Hien Quang Nguyen, Phat Tuan Nguyen, Van Thanh Nguyen, Robina Khan Niazi, Nafise Noroozi, Hasti Nouraei, Virginia Nuñez-Samudio, Khan M Nuruzzaman, Vincent Ebuka Nwatah, Chimezie Igwegbe Nzoputam, Ogochukwu Janet Nzoputam, Bogdan Oancea, Rahman Md Obaidur, Ismail A Odetokun, Ropo Ebenezer Ogunsakin, Osaretin Christabel Okonji, Andrew T Olagunju, Latera Tesfaye Olana, Isaac Iyinoluwa Olufadewa, Yinka Doris Oluwafemi, Kemal Sherefa Oumer, Amel Ouyahia, Mahesh P A, Keyvan Pakshir, Padmavali Nanaji Palange, Shahina Pardhan, Romil R Parikh, Jay Patel, Urvish K Patel, Shankargouda Patil, Uttam Paudel, Shrikant Pawar, Umberto Pensato, João Perdigão, Marcos Pereira, Mario F P Peres, Ionela-Roxana Petcu, Marina Pinheiro, Zahra Zahid Piracha, Nayanum Pokhrel, Maarten J Postma, Elton Junio Sady Prates, Ibrahim Qattea, Pankaja Raghav Raghav, Leila Rahbarnia, Vafa Rahimi-Movaghar, Mosiur Rahman, Muhammad Aziz Rahman, Vahid Rahmanian, Niloufar Rahnavard, Hazem Ramadan, Premkumar Ramasubramani, Usha Rani, Indu Ramachandra Rao, Deepthi Rapaka, Zubair Ahmed Ratan, Salman Rawaf, Elrashdy Moustafa Mohamed Redwan, Robert C Reiner Jr, Nazila Rezaei, Abanoub Riad, Tércia Moreira Ribeiro da Silva, Tamalee Roberts, Gisela Robles Aguilar, Jefferson Antonio Buendia Rodriguez, Victor Daniel Rosenthal, Basema Saddik, Saeid Sadeghian, Umar Saeed, Azam Safary, Fatemeh Saheb Sharif-Askari, Narjes Saheb Sharif-Askari, Amirhossein Sahebkar, Monalisha Sahu, Seyed Aidin Sajedi, Morteza Saki, Saina Salahi, Sarvenaz Salahi, Mohamed A Saleh, Malik Sallam, Sara Samadzadeh, Abdallah M Samy, Rama Krishna Sanjeev, Maheswar Satpathy, Allen Seylani, Abubakar Sha'aban, Mahan Shafie, Pritik A Shah, Shayan Shahrokhi, Kiana Shahzamani, Masood Ali Shaikh, Sunder Sham, Mohammed Shannawaz, Aziz Sheikh, Suchitra M Shenoy, Pavanchand H Shetty, Jae Il Shin, Fereshteh Shokri, Seyed Afshin Shorofi, Sunil Shrestha, Migbar Mekonnen Sibhat, Emmanuel Edwar Siddig, Luís Manuel Lopes Rodrigues Silva, Harpreet Singh, Jasvinder A Singh, Paramdeep Singh, Surjit Singh, Robert Sinto, Anna Aleksandrovna Skryabina, Bogdan Socea, Anton Sokhan, Ranjan Solanki, Yonatan Solomon, Prashant Sood, Sergey Soshnikov, Andy Stergachis, Mu'awiyyah Babale Sufiyan, Rizwan Suliankatchi Abdulkader, Abida Sultana, Sree Sudha T Y, Ensiyeh Taheri, Elahe Taki, Jacques JL Lukenze Tamuzi, Ker-Kan Tan, Nathan Y Tat, Mohamad-Hani Temsah, Dufera Rikitu Terefa, Pugazhenthan Thangaraju, Nigusie Selomon Tibebu, Jansje Henny Vera Ticoalu, Tala Tillawi, Marius Belmondo Tincho, Imad I Tleyjeh, Razie Toghroli, Marcos Roberto Tovani-Palone, Derara Girma Tufa, Paul Turner, Irfan Ullah, Chukwuma David Umeokonkwo, Bhaskaran Unnikrishnan, Seyed Mohammad Vahabi, Asokan Govindaraj Vaithinathan, Rohollah Valizadeh, Shoban Babu Varthya, Theo Vos, Yasir Waheed, Mandaras Tariku Walde, Cong Wang, Kosala Gayan Weerakoon, Nuwan Darshana Wickramasinghe, Andrea Sylvia Winkler, Melat Woldemariam, Nahom Alemseged Worku, Claire Wright, Dereje Y Yada, Sajad Yaghoubi, Gahin Abdulraheem Tayib Yahya Yahya, Chalachew Yenew Yenew Yenew, Metin Yesiltepe, Siyan Yi, Vahit Yiğit, Yuyi You, Hadiza Yusuf, Fathiah Zakham, Muhammad Zaman, Sojib Bin Zaman, Iman Zare, Zahra Zareshahrabadi, Armin Zarrintan, Mikhail Sergeevich Zastrozhin, Haijun Zhang, Jingya Zhang, Zhi-Jiang Zhang, Peng Zheng, Mohammad Zoladl, Alimuddin Zumla, Simon I Hay, Christopher J L Murray, Mohsen Naghavi, Hmwe Hmwe Kyu

## Abstract

**Background:**

Although meningitis is largely preventable, it still causes hundreds of thousands of deaths globally each year. WHO set ambitious goals to reduce meningitis cases by 2030, and assessing trends in the global meningitis burden can help track progress and identify gaps in achieving these goals. Using data from the Global Burden of Diseases, Injuries, and Risk Factors Study (GBD) 2019, we aimed to assess incident cases and deaths due to acute infectious meningitis by aetiology and age from 1990 to 2019, for 204 countries and territories.

**Methods:**

We modelled meningitis mortality using vital registration, verbal autopsy, sample-based vital registration, and mortality surveillance data. Meningitis morbidity was modelled with a Bayesian compartmental model, using data from the published literature identified by a systematic review, as well as surveillance data, inpatient hospital admissions, health insurance claims, and cause-specific meningitis mortality estimates. For aetiology estimation, data from multiple causes of death, vital registration, hospital discharge, microbial laboratory, and literature studies were analysed by use of a network analysis model to estimate the proportion of meningitis deaths and cases attributable to the following aetiologies: *Neisseria meningitidis*, *Streptococcus pneumoniae*, *Haemophilus influenzae*, group B *Streptococcus*, *Escherichia coli*, *Klebsiella pneumoniae*, *Listeria monocytogenes*, *Staphylococcus aureus*, viruses, and a residual other pathogen category.

**Findings:**

In 2019, there were an estimated 236 000 deaths (95% uncertainty interval [UI] 204 000–277 000) and 2·51 million (2·11–2·99) incident cases due to meningitis globally. The burden was greatest in children younger than 5 years, with 112 000 deaths (87 400–145 000) and 1·28 million incident cases (0·947–1·71) in 2019. Age-standardised mortality rates decreased from 7·5 (6·6–8·4) per 100 000 population in 1990 to 3·3 (2·8–3·9) per 100 000 population in 2019. The highest proportion of total all-age meningitis deaths in 2019 was attributable to *S pneumoniae* (18·1% [17·1–19·2]), followed by *N meningitidis* (13·6% [12·7–14·4]) and *K pneumoniae* (12·2% [10·2–14·3]). Between 1990 and 2019, *H influenzae* showed the largest reduction in the number of deaths among children younger than 5 years (76·5% [69·5–81·8]), followed by *N meningitidis* (72·3% [64·4–78·5]) and viruses (58·2% [47·1–67·3]).

**Interpretation:**

Substantial progress has been made in reducing meningitis mortality over the past three decades. However, more meningitis-related deaths might be prevented by quickly scaling up immunisation and expanding access to health services. Further reduction in the global meningitis burden should be possible through low-cost multivalent vaccines, increased access to accurate and rapid diagnostic assays, enhanced surveillance, and early treatment.

**Funding:**

Bill & Melinda Gates Foundation.

## Introduction

Meningitis is a disease defined by inflammation of the meninges, the layers of membranes that cover the brain and spinal cord.^1^ In addition to mortality, meningitis can result in long-term sequelae such as cognitive impairment, hearing loss, motor weakness or paralysis, incoordination, and epilepsy.^2^, ^3^ Bacterial and viral infections are key causes of acute meningitis. Viral meningitis is more common than bacterial meningitis but is associated with lower rates of mortality and complications.^4^ By contrast, bacterial meningitis is more likely to be associated with a poor prognosis and require prompt treatment.^5^, ^6^

A systematic review published in 2018 found that the aetiologies responsible for the highest proportion of bacterial meningitis cases in all regions globally were *Streptococcus pneumoniae* and *Neisseria meningitidis* (or meningococcus), with *Haemophilus influenzae* also posing a high burden in selected regions.^7^ Although vaccines can prevent infections due to these three aetiologies, not all countries have fully immunised their populations. For example, global infant third-dose vaccine coverage for *H influenzae* type b (Hib) is 72%, and third-dose vaccine coverage for *S pneumoniae* is estimated to be only 51%.^8^ Rates of meningitis incidence and mortality are highest in the meningitis belt, which consists of 26 countries in Africa spanning from Senegal to Ethiopia. This region has historically reported epidemics of *N meningitidis* serogroup A.^9^ To reduce the incidence rate of meningitis, 24 of the 26 countries in the meningitis belt had introduced meningococcal serogroup A conjugate vaccine (MACV [MenAfriVac]) campaigns by the end of 2021.^10^, ^11^ Globally, 53 countries have meningococcal conjugate vaccines in their routine immunisation schedule, including 27 countries with quadrivalent meningococcal ACYW vaccines and nine countries with meningococcal serogroup B vaccines.^12^

**Research in context**

**Evidence before this study**

The global burden of meningitis and the burden attributable to a subset of aetiologies have been estimated by multiple groups, including WHO and the Maternal and Child Epidemiology Estimation Group (WHO-MCEE) and the Global Burden of Diseases, Injuries, and Risk Factors Study (GBD). We searched PubMed with the search terms “meningitis” [MeSH] AND (“mortality” OR “incidence”) AND “global”, with no language restrictions, for articles published from database inception to April 26, 2023. We did not identify any studies that evaluated global levels of and trends in meningitis burden attributable to a comprehensive set of aetiologies across countries.

**Added value of this study**

This study uses data from GBD 2019 and improves upon previous GBD estimates in several ways. First, we added many new data sources on meningitis morbidity and mortality since GBD 2017. Second, we used a standardised approach to enhance the comparability of non-fatal data sources using a novel Bayesian meta-regression tool. Third, we produced estimates of meningitis burden attributable to ten different aetiologies (*Escherichia coli, Haemophilus influenzae, Klebsiella pneumoniae, Listeria monocytogenes, Neisseria meningitidis, Staphylococcus aureus*, group B *Streptococcus, Streptococcus pneumoniae,* viruses, and a residual other pathogen category), six of which are new inclusions to GBD. Finally, we address a key limitation of earlier GBD reports by producing non-fatal and fatal aetiology proportions that are linked to each other, using location-specific, age-specific, year-specific, and aetiology-specific case-fatality ratio estimates.

**Implications of all the available evidence**

There has been considerable progress in reducing the burden of infectious meningitis since 1990. However, the reduction has not been equal across locations, and countries in the African meningitis belt and south Asia still have a large burden. Meningitis attributable to *S pneumoniae*, which has a high case-fatality ratio, is responsible for more deaths than any other bacterial aetiology. There is a need to maintain and increasecoverage of pneumococcal, *H influenzae* type b, and multivalent meningococcal vaccines. For all aetiologies, improved surveillance systems, early diagnosis (through low-cost, accurate, and rapid tests), and improvements in health-care access and treatment are necessary to reduce the burden of meningitis and track progress. Our analysis highlights the importance of controlling bacterial meningitis not attributable to vaccine-preventable aetiologies. *K pneumoniae* was associated with more mortality than other aetiologies that do not have an available vaccine and was the third-largest cause of deaths attributable to infectious meningitis overall. New vaccines and vaccine programmes are in development for *K pneumoniae*, group B *Streptococcus,* and other forms of bacterial meningitis. The development of, and access to, vaccines are essential in reducing the global meningitis burden and in preventing the over-reliance on antibiotics that worsens antimicrobial resistance.

Reducing meningitis incidence and death rates is the focus of WHO's Defeating Meningitis by 2030 global roadmap, a plan developed in a global collaboration between key country stakeholders (ie, representatives from governments, global health organisations, public health bodies, academia, the private sector, and civil society) and international organisations.^13^ The global roadmap outlines three main goals: eliminating bacterial meningitis epidemics, reducing cases of and deaths from vaccine-preventable bacterial meningitis, and reducing disability and improving quality of life after recovery from acute meningitis.^13^ Robust and comparable estimates of meningitis incidence and mortality are invaluable in tracking progress and identifying gaps towards achieving these goals.

Here, we present results from the Global Burden of Diseases, Injuries, and Risk Factors Study (GBD) 2019, synthesised with results from the global burden of antimicrobial resistance study done by collaborators of the Global Research on Antimicrobial Resistance project, describing the burden and trends of acute infectious meningitis and ten aetiologies for 204 countries and territories from 1990 to 2019. Notable limitations of GBD 2016 and GBD 2017 were the divergent methods used for estimation of fatal and non-fatal aetiology proportions for *S pneumoniae*, *N meningitidis,* and Hib, and the absence of estimates for other important pathogens.^2^, ^14^, ^15^ As part of the study assessing the global burden of antimicrobial resistance, we developed a new method of pathogen estimation that addresses the limitations of these earlier GBD publications^16^ and provide the first estimates of meningitis burden attributable to a comprehensive set of pathogens. This manuscript was produced as part of the GBD Collaborator Network and in accordance with the GBD Protocol.

## Methods

### Modelling overview

GBD 2019 produces estimates of meningitis mortality and morbidity by age and sex for 204 countries and territories, for the years between 1990 and 2019. The study investigating the global burden of antimicrobial resistance produced estimates of the aetiology-specific fatal and non-fatal burden of selected infectious syndromes, including meningitis, between 1990 and 2019. Modelling was done at the 1000 draw level, and 95% uncertainty intervals (UIs) were computed as the 25th and 975th ranked values of 1000 draws. We used the GBD 2019 global population age standard to calculate age-standardised rates. Below, we summarise key methods from GBD 2019 and the study of the global burden of antimicrobial resistance for estimation of the burden of meningitis and its aetiologies. More details of these methods, including a flowchart for estimation of meningitis mortality and non-fatal burden, are provided in appendix 1 (pp 3, 8). Full descriptions of GBD 2019 and the study estimating the global burden of antimicrobial resistance have been previously published.^16^, ^17^ We present our results stratified by Socio-Demographic Index (SDI) quintiles. SDI is a composite indicator computed based on three variables (income per capita, average years of schooling, and total fertility rate) for each country.^18^ All count data are presented to three significant figures and all proportions and rates are presented to one decimal place.

### Mortality estimation

The data used for estimation of mortality due to meningitis originated from vital registration, verbal autopsy, sample-based vital registration, minimally invasive tissue sampling from the Child Health and Mortality Prevention Surveillance (CHAMPS),^19^ and epidemiological surveillance, comprising a total of 24 726 site-years (21 852 site-years from vital registration, 825 site-years from sample-based vital registration, 1432 site-years from verbal autopsy, 611 site-years from surveillance sources, and six site-years from minimally invasive tissue sampling). The data were processed with a set of standard algorithms accounting for incompleteness, misclassification of the underlying cause of death, garbage coding, and stochastic variability. The International Classification of Diseases ninth revision (ICD-9) and ICD-10 codes mapped to meningitis are provided in appendix 1 (p 4).

We estimated overall meningitis mortality using the Cause of Death Ensemble model (CODEm), which has widely been used to produce global estimates of cause-specific mortality.^14^, ^17^, ^20^ We modelled mortality separately for children aged 0–4 years and those aged 5 years and older because these two populations have very different meningitis mortality distributions and trends. The CODEm strategy evaluates various potential models with different combinations of covariates and model classes (mixed-effects linear models and spatiotemporal Gaussian process regression models).^21^ A full list of covariates is provided in appendix 1 (p 4). For this analysis, 26 countries in sub-Saharan Africa listed by WHO as being at risk for meningitis epidemics are considered meningitis belt countries.^22^ Models were weighted with out-of-sample predictive validity and combined in one ensemble model. Meningitis mortality estimates are scaled in a process called CoDCorrect so that all-cause mortality and the sum of cause-specific mortality are consistent.^17^, ^23^

### Morbidity estimation

The data used for morbidity estimation originated from published studies identified via a systematic review (appendix 1 p 9), surveillance data, cause-specific meningitis mortality estimates (calculated as described above), claims data, and inpatient data. We estimated overall meningitis morbidity using DisMod-MR (version 2.1), a Bayesian meta-regression tool that includes a compartmental model to estimate prevalence, incidence, remission, and mortality.^17^ Before modelling in DisMod-MR (version 2.1), we enhanced data comparability by applying a standardised approach to adjust claims and surveillance data to the level of inpatient data. More details of the adjustments are provided in appendix 1 (p 10).

### Aetiology-specific estimation

Data used for aetiology estimation originated from multiple cause of death vital registration data, hospital discharge data, microbial laboratory data, and published studies from the literature. We calculated mortality and morbidity estimates for each of ten aetiologies of meningitis: *Escherichia coli, H influenzae, Klebsiella pneumoniae, Listeria monocytogenes, N meningitidis, Staphylococcus aureus*, group B *Streptococcus, S pneumoniae,* viruses, and a residual other pathogen category. The ICD-9 and ICD-10 codes mapped to each aetiology are listed in appendix 1 (p 11). A key methodological improvement compared with GBD 2016 and GBD 2017 that we made in this study was to estimate the aetiologies using consistent methods for non-fatal and fatal proportions (ie, fatal aetiology proportions were derived from non-fatal aetiology proportions in combination with case-fatality ratio (CRF) estimates, as described below).

First, CFRs for each pathogen were modelled with the Bayesian meta-regression tool MR-BRT (meta-regression Bayesian, regularised, trimmed).^24^ CFRs were calculated as a function of age group, pathogen, and the Healthcare Access and Quality (HAQ) Index, with random effects on data source.^16^, ^24^, ^25^ The HAQ Index is a composite indicator of 32 causes of amenable mortality that measures health-care access and quality over time. We additionally controlled for data provided only from intensive care units (which would be biased towards higher CFRs) and modelled the effects of the proportion of pneumococcal conjugate vaccine (PCV) and Hib vaccinations among individuals aged 15 years or younger on the CFRs for *S pneumoniae* and *H influenzae*, respectively. The modelled CFRs were used to back-calculate case quantities from data sources that reported only deaths. Next, incidence proportions were estimated with multinomial estimation^16^ as part of a network analysis model, which allows for the inclusion of data sources that are considered partial observations (ie, which do not contain all ten pathogen groups modelled in the study). More details of this approach are provided in the online appendix of the published study on the global burden of antimicrobial resistance (pp 39–44).^16^

### Role of the funding source

The funder of the study had no role in study design, data collection, data analysis, data interpretation, or the writing of the report.

## Results

### Non-fatal and fatal burden of meningitis

In 2019, 2·51 million (95% UI 2·11–2·99) new cases of meningitis occurred globally among all age groups (table 1), of which 1·28 million (0·947–1·71) occurred in children younger than 5 years (table 2). Age-standardised incidence rates per 100 000 population were highest in western sub-Saharan Africa, with an incidence rate of 105·8 (90·1–124·1) per 100 000 population, followed by eastern sub-Saharan Africa with an incidence rate of 97·3 (83·4–112·3) per 100 000 population and central sub-Saharan Africa with an incidence rate of 87·2 (75·6–99·6) per 100 000 population. Overall, the age-standardised global incidence rate was 35·4 (29·6–42·5) cases per 100 000 population, varying from 4·3 (3·6–5·1) per 100 000 population in the USA to 257·1 (221·2–297·7) per 100 000 population in South Sudan (appendix 2 pp 2–19). Globally, in 2019, the incidence rate in children younger than 5 years (hereafter referred to as the under-5 incidence rate) was 192·4 (142·8–258·6) per 100 000 population, down from 312·0 (231·6–418·6) per 100 000 population in 1990, representing a 38·3% (37·0–39·5) decrease (table 2). By SDI quintile, under-5 incidence rates in 2019 ranged from 34·4 (23·5–49·9) per 100 000 population in high SDI quintiles to 400·2 (302·8–529·4) per 100 000 population in low SDI quintiles (table 2). Globally, in 2019, the incidence rate of children aged 0–27 days (neonates) was 854·8 (609·2–1183·3) per 100 000 population, down from 1301·2 per (932·4–1767·2) per 100 000 population in 1990, representing a 34·3% (32·7–35·8) decrease (table 3).

**Table 1:** All-age meningitis incidence and mortality counts and age-standardised rates in 1990 and 2019, and percentage change in deaths, cases, and age-standardised incidence and mortality rates between 1990 and 2019, by SDI quintile, globally, and for the seven GBD super-regions and 21 GBD regions

		**Mortality**				**Incidence**				**Percentage change (1990–2019)**			
		1990 counts	Age- standardised rate (per 100 000), 1990	2019 counts	Age- standardised rate (per 100 000), 2019	1990 counts	Age- standardised rate (per 100 000), 1990	2019 counts	Age- standardised rate (per 100 000), 2019	Mortality	Age- standardised mortality rate	Incidence	Age- standardised incidence rate
Global		433 000 (376 000 to 494 000)	7·5 (6·6 to 8·4)	236 000 (204 000 to 277 000)	3·3 (2·8 to 3·9)	3 290 000 (2 700 000 to 4 000 000)	55·3 (45·9 to 66·7)	2 510 000 (2 110 000 to 2 990 000)	35·4 (29·6 to 42·5)	−45·4% (−53·5 to −35·8)	−56·0% (−62·5 to −48·3)	−23·8% (−26·5 to −20·4)	−35·9% (−37·0 to −34·6)
	Low SDI	164 000 (137 000 to 196 000)	24·8 (21·5 to 28·5)	129 000 (106 000 to 157 000)	11·2 (9·6 to 13·2)	1 050 000 (864 000 to 1 280 000)	145·4 (125·0 to 168·7)	1 100 000 (914 000 to 1 330 000)	82·3 (70·3 to 96·0)	−21·4% (−35·5 to −3·2)	−54·7% (−61·8 to −46·1)	4·8% (2·0 to 7·9)	−43·4% (−44·4 to −42·6)
	Low-middle SDI	148 000 (129 000 to 170 000)	11·7 (10·5 to 13·2)	65 800 (57 800 to 75 400)	4·1 (3·6 to 4·6)	1 070 000 (876 000 to 1 310 000)	77·1 (64·8 to 91·4)	725 000 (606 000 to 860 000)	42·1 (35·4 to 50·0)	−55·5% (−62·5 to −47·2)	−65·3% (−70·2 to −59·7)	−32·4% (−35·7 to −28·3)	−45·4% (−46·7 to −43·8)
	Middle SDI	86 200 (76 800 to 96 700)	4·8 (4·3 to 5·4)	29 400 (26 300 to 33 100)	1·4 (1·2 to 1·6)	715 000 (573 000 to 890 000)	37·7 (30·8 to 46·3)	425 000 (349 000 to 508 000)	20·5 (16·8 to 24·7)	−65·9% (−70·8 to −59·8)	−71·6% (−75·5 to −66·8)	−40·5% (−44·4 to −35·3)	−45·6% (−47·7 to −43·0)
	High-middle SDI	27 400 (25 000 to 30 000)	2·6 (2·4 to 2·8)	8520 (7840 to 9170)	0·6 (0·6 to 0·7)	306 000 (250 000 to 368 000)	28·2 (23·0 to 34·2)	166 000 (135 000 to 197 000)	14·9 (12·0 to 18·0)	−68·9% (−72·8 to −64·8)	−76·6% (−79·7 to −73·2)	−45·9% (−49·5 to −41·4)	−47·0% (−49·6 to −44·1)
	High SDI	6590 (6370 to 6810)	0·8 (0·8 to 0·8)	3300 (3090 to 3480)	0·3 (0·2 to 0·3)	147 000 (118 000 to 178 000)	20·7 (16·5 to 25·2)	91 800 (74 600 to 110 000)	11·1 (8·9 to 13·5)	−49·9% (−52·4 to −47·5)	−68·8% (−70·5 to −67·2)	−37·5% (−41·5 to −32·3)	−46·2% (−48·2 to −43·5)
Central Europe, eastern Europe, and central Asia		8190 (7840 to 8550)	2·2 (2·1 to 2·3)	2700 (2440 to 2960)	0·6 (0·6 to 0·7)	130 000 (107 000 to 155 000)	34·3 (28·3 to 40·7)	70 800 (57 000 to 86 000)	20·9 (16·6 to 25·6)	−67·0% (−70·3 to −63·3)	−71·4% (−74·5 to −67·9)	−45·7% (−48·6 to −42·5)	−39·2% (−43·1 to −35·5)
	Central Asia	3030 (2790 to 3290)	3·7 (3·5 to 4·0)	638 (552 to 752)	0·7 (0·6 to 0·8)	41 100 (34 500 to 48 400)	49·9 (42·2 to 58·6)	27 500 (22 000 to 33 800)	28·9 (23·1 to 35·6)	−79·0% (−82·2 to −74·7)	−81·2% (−83·9 to −77·5)	−33·2% (−39·2 to −27·6)	−42·1% (−47·0 to −37·4)
	Central Europe	1740 (1670 to 1830)	1·6 (1·6 to 1·7)	421 (357 to 484)	0·3 (0·3 to 0·3)	22 600 (18 400 to 27 100)	21·5 (17·5 to 25·9)	10 300 (8220 to 12 300)	12·2 (9·6 to 15·1)	−75·8% (−79·6 to −71·9)	−81·7% (−84·8 to −78·5)	−54·6% (−57·2 to −50·9)	−43·2% (−46·7 to −39·7)
	Eastern Europe	3420 (3280 to 3570)	1·7 (1·6 to 1·8)	1640 (1480 to 1800)	0·7 (0·6 to 0·8)	66 700 (54 000 to 79 800)	34·5 (27·8 to 41·6)	33 000 (26 700 to 39 800)	19·9 (15·7 to 24·4)	−52·0% (−57·3 to −46·7)	−58·5% (−62·9 to −54·0)	−50·5% (−53·2 to −47·4)	−42·5% (−46·0 to −38·7)
High income		7690 (7460 to 7930)	0·9 (0·9 to 0·9)	3820 (3570 to 4010)	0·3 (0·3 to 0·3)	164 000 (132 000 to 200 000)	21·3 (17·0 to 25·9)	96 000 (78 200 to 114 000)	10·8 (8·7 to 13·1)	−50·3% (−52·8 to −48·2)	−68·9% (−70·7 to −67·3)	−41·5% (−45·2 to −37·0)	−49·1% (−51·2 to −46·5)
	Australasia	125 (117 to 134)	0·7 (0·6 to 0·7)	59 (54 to 64)	0·2 (0·2 to 0·2)	4460 (3560 to 5500)	25·1 (19·7 to 31·1)	3490 (2790 to 4280)	15·3 (11·9 to 19·2)	−53·0% (−57·6 to −48·1)	−72·1% (−75·5 to −68·5)	−21·7% (−26·4 to −16·1)	−39·3% (−43·5 to −34·9)
	High-income Asia Pacific	1220 (1170 to 1280)	0·8 (0·7 to 0·8)	491 (427 to 531)	0·1 (0·1 to 0·1)	45 600 (35 500 to 57 300)	31·8 (24·8 to 39·9)	27 700 (22 200 to 33 600)	21·2 (16·7 to 26·5)	−59·9% (−64·6 to −56·6)	−82·5% (−83·9 to −81·0)	−39·3% (−44·7 to −32·3)	−33·3% (−38·0 to −28·0)
	High-income North America	1960 (1870 to 2080)	0·7 (0·7 to 0·7)	1240 (1170 to 1280)	0·3 (0·3 to 0·3)	29 800 (23 500 to 36 700)	11·6 (9·1 to 14·5)	16 300 (13 600 to 19 300)	4·4 (3·7 to 5·2)	−37·0% (−40·7 to −33·9)	−58·9% (−61·5 to −56·7)	−45·2% (−52·5 to −36·4)	−61·9% (−66·4 to −56·1)
	Southern Latin America	1190 (1140 to 1250)	2·5 (2·3 to 2·6)	580 (524 to 631)	0·8 (0·7 to 0·9)	11 400 (9500 to 13 700)	22·7 (18·9 to 27·0)	6780 (5550 to 8200)	11·4 (9·2 to 13·9)	−51·4% (−56·3 to −46·0)	−66·8% (−70·4 to −63·0)	−40·7% (−44·7 to −36·4)	−49·9% (−52·9 to −46·7)
	Western Europe	3180 (3080 to 3300)	0·8 (0·8 to 0·9)	1450 (1350 to 1550)	0·2 (0·2 to 0·3)	72 900 (59 400 to 87 100)	23·8 (19·3 to 28·9)	41 700 (34 000 to 49 600)	12·5 (10·0 to 15·2)	−54·3% (−57·1 to −51·7)	−71·4% (−73·5 to −69·5)	−42·7% (−45·3 to −39·7)	−47·4% (−49·2 to −45·4)
Latin America and Caribbean		18 100 (16 700 to 19 600)	4·1 (3·9 to 4·4)	5560 (4720 to 6510)	1·0 (0·9 to 1·2)	140 000 (115 000 to 166 000)	31·2 (25·9 to 36·5)	93 000 (76 000 to 111 000)	16·8 (13·7 to 20·0)	−69·2% (−74·8 to −63·0)	−75·2% (−79·7 to −70·3)	−33·5% (−39·8 to −25·8)	−46·3% (−49·9 to −42·3)
	Andean Latin America	1320 (1150 to 1530)	2·9 (2·6 to 3·4)	395 (314 to 494)	0·6 (0·5 to 0·8)	7020 (5830 to 8360)	14·7 (12·5 to 17·1)	4400 (3590 to 5270)	6·9 (5·7 to 8·3)	−70·0% (−77·4 to −60·7)	−77·9% (−83·2 to −71·4)	−37·3% (−42·5 to −31·8)	−52·7% (−56·2 to −48·9)
	Caribbean	3470 (2930 to 4080)	8·9 (7·6 to 10·4)	1420 (1080 to 1860)	3·4 (2·5 to 4·5)	14 200 (12 000 to 16 600)	36·3 (31·2 to 42·0)	10 000 (8390 to 11 700)	23·4 (19·4 to 27·6)	−59·0% (−70·7 to −43·6)	−62·1% (−73·2 to −47·6)	−29·3% (−32·8 to −25·5)	−35·6% (−39·0 to −32·5)
	Central Latin America	4720 (4400 to 5070)	2·6 (2·4 to 2·7)	1670 (1370 to 2030)	0·7 (0·6 to 0·9)	37 300 (30 000 to 45 400)	18·8 (15·6 to 22·5)	19 900 (16 000 to 24 200)	8·4 (6·7 to 10·2)	−64·6% (−71·9 to −56·2)	−72·9% (−78·4 to −66·5)	−46·6% (−51·1 to −41·1)	−55·4% (−58·1 to −52·6)
	Tropical Latin America	8550 (7700 to 9660)	5·3 (4·8 to 5·9)	2070 (1870 to 2290)	1·0 (0·9 to 1·1)	81 500 (66 200 to 96 600)	48·7 (40·1 to 57·4)	58 700 (47 800 to 70 600)	28·1 (22·8 to 34·0)	−75·7% (−79·4 to −71·7)	−80·8% (−83·7 to −77·6)	−28·0% (−36·3 to −18·3)	−42·4% (−47·9 to −36·7)
North Africa and Middle East		17 500 (14 400 to 21 500)	4·2 (3·6 to 5·1)	6280 (5330 to 7400)	1·2 (1·0 to 1·4)	143 000 (118 000 to 171 000)	35·7 (30·5 to 41·2)	129 000 (107 000 to 153 000)	22·5 (18·9 to 26·4)	−64·1% (−72·2 to −54·5)	−71·6% (−77·1 to −64·9)	−9·9% (−16·3 to −1·9)	−37·0% (−39·9 to −33·9)
South Asia		131 000 (113 000 to 150 000)	11·2 (10·0 to 12·5)	55 500 (48 400 to 64 400)	3·5 (3·0 to 4·0)	1 150 000 (935 000 to 1 410 000)	85·9 (71·6 to 103·0)	742 000 (610 000 to 883 000)	43·6 (36·2 to 52·3)	−57·5% (−65·0 to −49·0)	−69·0% (−74·0 to −63·3)	−35·3% (−38·7 to −31·2)	−49·2% (−50·7 to −47·5)
Southeast Asia, east Asia, and Oceania		76 800 (65 800 to 88 900)	4·5 (3·9 to 5·2)	18 200 (16 300 to 20 500)	1·0 (0·9 to 1·2)	452 000 (357 000 to 575 000)	25·7 (20·4 to 32·6)	177 000 (147 000 to 212 000)	10·9 (8·8 to 13·3)	−76·3% (−80·3 to −71·7)	−77·4% (−81·2 to −73·2)	−60·9% (−63·5 to −57·6)	−57·6% (−59·3 to −55·3)
	East Asia	38 600 (33 800 to 43 700)	3·4 (3·0 to 3·8)	6790 (5940 to 7680)	0·5 (0·5 to 0·6)	234 000 (182 000 to 302 000)	19·7 (15·4 to 25·3)	50 700 (41 600 to 60 300)	4·8 (3·9 to 6·0)	−82·4% (−85·4 to −78·7)	−85·0% (−87·5 to −81·9)	−78·4% (−80·4 to −75·8)	−75·4% (−77·0 to −73·7)
	Oceania	783 (599 to 1000)	9·3 (7·4 to 11·5)	693 (497 to 937)	4·4 (3·3 to 5·7)	5530 (4580 to 6530)	74·5 (64·7 to 84·9)	6690 (5650 to 7860)	46·0 (39·6 to 52·8)	−11·5% (−38·6 to 28·1)	−52·7% (−65·5 to −34·4)	21·1% (14·0 to 30·2)	−38·2% (−41·3 to −34·5)
	Southeast Asia	37 400 (30 300 to 46 800)	6·9 (5·7 to 8·4)	10 700 (9320 to 12 400)	1·8 (1·6 to 2·1)	213 000 (169 000 to 266 000)	38·1 (30·9 to 47·3)	120 000 (98 900 to 144 000)	20·1 (16·6 to 24·4)	−71·3% (−77·6 to −63·2)	−73·1% (−78·8 to −66·2)	−43·7% (−47·9 to −38·0)	−47·2% (−49·8 to −43·8)
Sub-Saharan Africa		174 000 (143 000 to 208 000)	29·3 (25·4 to 33·8)	144 000 (117 000 to 176 000)	14·3 (12·2 to 16·7)	1 110 000 (920 000 to 1 350 000)	167·3 (143·4 to 194·1)	1 200 000 (998 000 to 1 450 000)	96·3 (82·6 to 111·7)	−17·0% (−32·2 to 3·7)	−51·3% (−58·6 to −42·0)	7·8% (4·8 to 11·2)	−42·4% (−43·4 to −41·5)
	Central sub-Saharan Africa	14 500 (11 400 to 18 300)	19·6 (15·9 to 23·7)	9990 (7720 to 12 900)	8·9 (6·8 to 11·2)	107 000 (88 200 to 128 000)	135·3 (116·9 to 155·9)	128 000 (108 000 to 153 000)	87·2 (75·6 to 99·6)	−31·1% (−49·4 to −10·9)	−54·6% (−65·4 to −42·7)	19·8% (11·8 to 29·0)	−35·5% (−38·6 to −32·0)
	Eastern sub- Saharan Africa	69 000 (57 800 to 81 900)	32·7 (28·1 to 37·4)	42 400 (35 800 to 50 700)	13·2 (11·5 to 15·1)	429 000 (354 000 to 519 000)	171·7 (146·6 to 198·2)	436 000 (364 000 to 525 000)	97·3 (83·4 to 112·3)	−38·5% (−50·1 to −24·2)	−59·5% (−65·1 to −52·5)	1·8% (−3·0 to 6·8)	−43·4% (−45·0 to −41·7)
	Southern sub- Saharan Africa	4070 (3500 to 4610)	8·0 (7·0 to 8·9)	4130 (3520 to 4770)	6·1 (5·3 to 6·9)	33 200 (27 700 to 39 600)	59·6 (51·0 to 69·0)	34 000 (29 000 to 39 500)	45·8 (39·3 to 52·6)	1·4% (−15·9 to 23·0)	−24·0% (−35·8 to −10·7)	2·5% (−3·1 to 9·2)	−23·2% (−25·6 to −20·5)
	Western sub- Saharan Africa	86 200 (69 300 to 107 000)	34·7 (29·1 to 41·2)	87 600 (68 300 to 110 000)	18·4 (15·0 to 22·1)	544 000 (447 000 to 665 000)	198·8 (170·6 to 230·9)	602 000 (494 000 to 737 000)	105·8 (90·1 to 124·1)	1·6% (−19·7 to 31·2)	−47·0% (−56·2 to −35·3)	10·6% (8·1 to 13·2)	−46·8% (−47·6 to −45·8)

Data in parentheses are 95% uncertainty intervals. Count data are presented to three significant figures and rates are presented to one decimal place. SDI=Socio-demographic Index. GBD=Global Burden of Diseases, Injuries, and Risk Factors Study.

**Table 2:** Under-5 meningitis incidence and mortality rates and counts in 1990 and 2019, and percentage change in deaths, cases, and incidence and mortality rates between 1990 and 2019, by SDI quintile, globally, and for seven GBD super-regions and 21 GBD regions

		**Mortality**				**Incidence**				**Percentage change (1990–2019)**			
		1990 counts	Mortality rate (per 100 000), 1990	2019 counts	Mortality rate (per 100 000), 2019	1990 counts	Incidence rate (per 100 000), 1990	2019 counts	Incidence rate (per 100 000), 2019	Mortality	Mortality rate	Incidence	Incidence rate
Global		284 000 (237 000 to 339 000)	45·0 (37·4 to 53·6)	112 000 (87 400 to 145 000)	16·9 (13·2 to 21·9)	1 970 000 (1 460 000 to 2 650 000)	312·0 (231·6 to 418·6)	1 280 000 (947 000 to 1 710 000)	192·4 (142·8 to 258·6)	−60·5% (−69·2 to −49·8)	−62·3% (−70·6 to −52·1)	−35·3% (−36·5 to −34·0)	−38·3% (−39·5 to −37·0)
	Low SDI	118 000 (94 200 to 146 000)	122·3 (97·5 to 151·4)	77 600 (59 100 to 102 000)	45·4 (34·6 to 59·8)	719 000 (549 000 to 938 000)	744·8 (568·8 to 971·2)	684 000 (517 000 to 904 000)	400·2 (302·8 to 529·4)	−34·3% (−49·2 to −14·0)	−62·9% (−71·3 to −51·4)	−4·9% (−7·6 to −2·1)	−46·3% (−47·8 to −44·7)
	Low-middle SDI	93 100 (76 600 to 114 000)	55·3 (45·6 to 67·8)	24 400 (19 000 to 31 500)	14·1 (11·0 to 18·3)	639 000 (473 000 to 854 000)	380·0 (281·0 to 507·6)	348 000 (254 000 to 466 000)	202·0 (147·2 to 270·2)	−73·8% (−80·3 to −65·0)	−74·4% (−80·7 to −65·9)	−45·6% (−47·4 to −43·6)	−46·8% (−48·6 to −44·9)
	Middle SDI	55 600 (47 000 to 65 500)	27·1 (22·9 to 32·0)	8320 (6680 to 10 400)	4·5 (3·6 to 5·6)	420 000 (301 000 to 572 000)	205·0 (147·0 to 279·4)	175 000 (125 000 to 243 000)	95·0 (68·1 to 132·2)	−85·0% (−88·4 to −80·9)	−83·4% (−87·1 to −78·8)	−58·3% (−59·7 to −56·6)	−53·7% (−55·2 to −51·7)
	High-middle SDI	15 400 (13 400 to 17 800)	14·7 (12·8 to 17·0)	1560 (1310 to 1860)	1·9 (1·6 to 2·2)	147 000 (107 000 to 202 000)	140·4 (102·2 to 192·9)	49 900 (35 100 to 70 900)	60·4 (42·5 to 85·8)	−89·9% (−92·1 to −87·2)	−87·2% (−90·0 to −83·8)	−66·0% (−67·8 to −63·9)	−57·0% (−59·3 to −54·4)
	High SDI	1710 (1610 to 1840)	3·0 (2·8 to 3·2)	296 (256 to 334)	0·6 (0·5 to 0·6)	45 900 (32 100 to 65 800)	79·7 (55·6 to 114·1)	18 000 (12 300 to 26 100)	34·4 (23·5 to 49·9)	−82·7% (−85·2 to −80·5)	−81·0% (−83·7 to −78·5)	−60·7% (−62·2 to −59·5)	−56·8% (−58·5 to −55·4)
Central Europe, eastern Europe, and central Asia		4180 (3880 to 4520)	11·7 (10·9 to 12·7)	450 (368 to 565)	1·6 (1·3 to 2·0)	55 800 (42 300 to 75 600)	156·7 (118·6 to 212·2)	21 200 (15 100 to 30 200)	77·1 (54·8 to 109·5)	−89·2% (−91·4 to −86·2)	−86·0% (−88·8 to −82·1)	−62·0% (−65·0 to −58·4)	−50·8% (−54·8 to −46·2)
	Central Asia	1900 (1680 to 2130)	20·0 (17·7 to 22·5)	186 (142 to 269)	1·9 (1·5 to 2·8)	20 200 (15 800 to 26 200)	213·2 (166·7 to 276·3)	10 100 (7290 to 14 100)	105·6 (76·1 to 147·5)	−90·2% (−92·7 to −85·0)	−90·3% (−92·8 to −85·2)	−50·0% (−55·6 to −42·7)	−50·5% (−56·1 to −43·3)
	Central Europe	801 (737 to 882)	9·0 (8·3 to 9·9)	42 (32 to 52)	0·7 (0·6 to 0·9)	8680 (6440 to 11 800)	97·2 (72·1 to 132·5)	2530 (1780 to 3660)	44·8 (31·4 to 64·8)	−94·8% (−96·1 to −93·3)	−91·8% (−93·9 to −89·4)	−70·8% (−73·2 to −67·6)	−53·8% (−57·6 to −48·8)
	Eastern Europe	1480 (1370 to 1620)	8·6 (8·0 to 9·4)	223 (183 to 267)	1·8 (1·5 to 2·2)	26 900 (19 600 to 37 300)	156·4 (113·6 to 216·6)	8600 (5920 to 12 500)	69·7 (48·0 to 101·2)	−84·9% (−88·1 to −81·8)	−78·9% (−83·4 to −74·6)	−68·1% (−70·4 to −65·4)	−55·4% (−58·7 to −51·6)
High income		2230 (2110 to 2390)	3·6 (3·4 to 3·9)	380 (327 to 435)	0·7 (0·6 to 0·8)	51 600 (36 600 to 73 000)	84·0 (59·6 to 118·9)	19 000 (13 100 to 27 100)	33·4 (23·0 to 47·7)	−83·0% (−85·6 to −80·5)	−81·7% (−84·4 to −79·0)	−63·2% (−64·7 to −61·9)	−60·3% (−61·9 to −58·9)
	Australasia	51 (44 to 58)	3·3 (2·9 to 3·8)	12 (9 to 15)	0·7 (0·5 to 0·8)	1640 (1180 to 2250)	106·2 (76·4 to 146·0)	1040 (706 to 1530)	57·1 (38·8 to 84·0)	−76·6% (−82·6 to −69·8)	−80·2% (−85·2 to −74·3)	−36·5% (−44·6 to −28·2)	−46·2% (−53·0 to −39·1)
	High-income Asia Pacific	228 (195 to 265)	2·2 (1·9 to 2·6)	17 (14 to 19)	0·2 (0·2 to 0·3)	10 600 (7160 to 15 700)	103·2 (69·8 to 153·0)	3320 (2230 to 4820)	45·5 (30·6 to 66·1)	−92·7% (−94·0 to −91·0)	−89·7% (−91·6 to −87·4)	−68·6% (−70·5 to −66·7)	−55·9% (−58·5 to −53·2)
	High-income North America	587 (539 to 662)	2·7 (2·5 to 3·1)	137 (122 to 152)	0·7 (0·6 to 0·7)	9940 (6380 to 15 300)	46·2 (29·7 to 71·3)	2200 (1490 to 3200)	10·5 (7·1 to 15·2)	−76·7% (−80·0 to −73·3)	−76·1% (−79·5 to −72·6)	−77·9% (−79·5 to −75·4)	−77·3% (−79·0 to −74·8)
	Southern Latin America	552 (499 to 608)	10·7 (9·7 to 11·8)	81 (60 to 105)	1·7 (1·2 to 2·2)	5620 (4360 to 7460)	108·9 (84·5 to 144·5)	2180 (1550 to 3100)	44·8 (32·0 to 63·8)	−85·3% (−89·4 to −80·3)	−84·4% (−88·8 to −79·1)	−61·3% (−66·0 to −56·2)	−58·8% (−63·9 to −53·4)
	Western Europe	817 (760 to 894)	3·6 (3·3 to 3·9)	134 (111 to 157)	0·6 (0·5 to 0·7)	23 800 (17 200 to 33 000)	103·7 (74·8 to 143·7)	10 300 (7150 to 14 800)	46·7 (32·5 to 67·1)	−83·6% (−86·9 to −80·7)	−82·9% (−86·3 to −79·8)	−56·9% (−59·3 to −54·7)	−55·0% (−57·6 to −52·7)
Latin America and Caribbean		12 400 (11 200 to 14 000)	24·8 (22·4 to 28·0)	2040 (1520 to 2630)	4·2 (3·2 to 5·5)	71 600 (54 000 to 95 400)	143·2 (107·9 to 190·6)	25 000 (18 000 to 35 100)	52·1 (37·4 to 73·1)	−83·6% (−88·2 to −78·2)	−82·9% (−87·7 to −77·3)	−65·0% (−67·3 to −61·9)	−63·6% (−66·0 to −60·3)
	Andean Latin America	868 (715 to 1050)	15·9 (13·1 to 19·3)	112 (80 to 161)	1·8 (1·3 to 2·5)	4500 (3480 to 5790)	82·5 (63·9 to 106·3)	2120 (1540 to 2900)	33·5 (24·4 to 45·8)	−87·1% (−91·1 to −80·7)	−88·9% (−92·4 to −83·4)	−52·9% (−57·7 to −47·2)	−59·5% (−63·6 to −54·6)
	Caribbean	2600 (2080 to 3190)	62·9 (50·2 to 77·0)	947 (633 to 1350)	24·0 (16·0 to 34·2)	8470 (6590 to 10 800)	204·7 (159·2 to 260·3)	5070 (3750 to 6730)	128·4 (95·0 to 170·3)	−63·6% (−77·2 to −44·2)	−61·9% (−76·1 to −41·5)	−40·1% (−44·9 to −35·4)	−37·3% (−42·2 to −32·3)
	Central Latin America	2970 (2670 to 3300)	13·0 (11·7 to 14·4)	365 (261 to 490)	1·7 (1·2 to 2·3)	21 400 (15 900 to 28 700)	93·5 (69·5 to 125·4)	6930 (4870 to 9940)	32·0 (22·5 to 45·9)	−87·7% (−91·5 to −83·1)	−87·0% (−91·1 to −82·2)	−67·6% (−70·2 to −64·1)	−65·8% (−68·5 to −62·1)
	Tropical Latin America	5980 (5130 to 7090)	34·1 (29·2 to 40·4)	613 (472 to 767)	3·8 (2·9 to 4·8)	37 300 (27 500 to 50 200)	212·4 (156·9 to 285·9)	10 900 (7730 to 15 900)	67·7 (47·9 to 98·5)	−89·7% (−92·5 to −86·4)	−88·8% (−91·8 to −85·2)	−70·7% (−73·5 to −67·4)	−68·1% (−71·1 to −64·5)
North Africa and Middle East		12 500 (9850 to 16 400)	23·5 (18·5 to 30·7)	2310 (1740 to 3100)	3·9 (2·9 to 5·2)	84 700 (63 700 to 112 000)	158·8 (119·4 to 210·1)	48 800 (35 500 to 67 700)	81·7 (59·4 to 113·4)	−81·5% (−87·0 to −74·1)	−83·5% (−88·4 to −76·9)	−42·4% (−46·7 to −37·5)	−48·5% (−52·4 to −44·2)
South Asia		77 100 (62 300 to 93 300)	47·6 (38·5 to 57·7)	21 400 (16 200 to 28 200)	13·0 (9·9 to 17·1)	659 000 (479 000 to 893 000)	407·1 (296·0 to 551·8)	338 000 (245 000 to 461 000)	205·7 (148·8 to 280·3)	−72·3% (−80·3 to −62·2)	−72·7% (−80·6 to −62·9)	−48·7% (−51·0 to −46·5)	−49·5% (−51·7 to −47·4)
Southeast Asia, east Asia, and Oceania		54 100 (44 300 to 66 300)	30·0 (24·6 to 36·8)	6500 (5300 to 7950)	4·6 (3·8 to 5·7)	277 000 (194 000 to 385 000)	153·4 (107·6 to 213·8)	80 600 (57 400 to 110 000)	57·4 (40·9 to 78·6)	−88·0% (−90·8 to −84·6)	−84·6% (−88·2 to −80·3)	−70·9% (−71·9 to −69·6)	−62·6% (−63·9 to −60·9)
	East Asia	25 700 (21 300 to 30 300)	21·4 (17·7 to 25·3)	1510 (1210 to 1840)	1·8 (1·4 to 2·2)	131 000 (88 100 to 191 000)	109·1 (73·6 to 159·1)	21 200 (14 500 to 30 300)	25·2 (17·2 to 36·1)	−94·1% (−95·6 to −92·2)	−91·7% (−93·7 to −88·9)	−83·8% (−84·9 to −82·6)	−76·9% (−78·4 to −75·2)
	Oceania	579 (413 to 785)	58·9 (42·0 to 79·9)	469 (305 to 687)	25·3 (16·5 to 37·1)	3270 (2480 to 4220)	333·0 (252·2 to 428·9)	3780 (2920 to 4840)	204·2 (157·6 to 261·6)	−18·9% (−49·7 to 32·1)	−56·9% (−73·3 to −29·8)	15·4% (6·1 to 28·4)	−38·7% (−43·6 to −31·8)
	Southeast Asia	27 900 (20 900 to 37 200)	46·8 (35·2 to 62·6)	4520 (3510 to 5800)	8·3 (6·4 to 10·6)	143 000 (104 000 to 193 000)	239·6 (175·2 to 324·9)	55 600 (40 200 to 75 600)	102·1 (73·8 to 138·7)	−83·8% (−88·5 to −77·4)	−82·3% (−87·4 to −75·3)	−61·0% (−62·8 to −58·9)	−57·4% (−59·4 to −55·1)
Sub-Saharan Africa		122 000 (95 800 to 155 000)	135·7 (106·9 to 172·5)	79 200 (59 200 to 104 000)	47·8 (35·8 to 63·0)	773 000 (591 000 to 1 010 000)	862·9 (659·9 to 1126·3)	743 000 (561 000 to 982 000)	448·3 (338·6 to 592·9)	−34·9% (−49·7 to −13·1)	−64·8% (−72·8 to −53·0)	−4·0% (−6·8 to −0·9)	−48·0% (−49·6 to −46·4)
	Central sub-Saharan Africa	10 100 (7230 to 13 700)	93·5 (67·3 to 127·6)	3400 (2240 to 5270)	16·4 (10·8 to 25·5)	74 100 (56 500 to 94 300)	689·4 (525·4 to 876·8)	70 500 (53 700 to 93 600)	340·6 (259·4 to 452·4)	−66·2% (−77·2 to −50·1)	−82·5% (−88·2 to −74·1)	−4·9% (−12·6 to 4·3)	−50·6% (−54·6 to −45·8)
	Eastern sub-Saharan Africa	45 100 (35 600 to 56 200)	125·2 (98·9 to 156·1)	17 100 (12 700 to 23 000)	26·7 (19·8 to 35·9)	288 000 (217 000 to 378 000)	798·9 (603·6 to 1049·1)	245 000 (184 000 to 327 000)	382·0 (287·5 to 509·2)	−62·0% (−71·5 to −48·9)	−78·6% (−84·0 to −71·3)	−14·8% (−18·7 to −10·4)	−52·2% (−54·3 to −49·7)
	Southern sub- Saharan Africa	1810 (1370 to 2270)	25·3 (19·1 to 31·7)	784 (571 to 1070)	9·7 (7·1 to 13·3)	15 900 (11 800 to 21 200)	222·0 (164·7 to 295·5)	11 300 (8330 to 15 200)	139·5 (102·9 to 187·2)	−56·7% (−69·8 to −36·1)	−61·6% (−73·2 to −43·4)	−29·1% (−32·5 to −25·2)	−37·2% (−40·2 to −33·7)
	Western sub- Saharan Africa	64 600 (50 000 to 84 600)	181·2 (140·3 to 237·2)	57 800 (42 700 to 76 000)	79·5 (58·8 to 104·4)	396 000 (304 000 to 517 000)	1108·7 (853·4 to 1449·5)	416 000 (314 000 to 546 000)	571·7 (431·4 to 751·1)	−10·5% (−32·0 to 21·3)	−56·1% (−66·7 to −40·5)	5·1% (2·4 to 8·0)	−48·4% (−49·8 to −47·0)

Data in parentheses are 95% uncertainty intervals. Count data are presented to three significant figures and rates are presented to one decimal place. SDI=Socio-demographic Index. GBD=Global Burden of Diseases, Injuries, and Risk Factors Study.

**Table 3:** Neonatal meningitis incidence and mortality rates and counts in 1990 and 2019, and percentage change in deaths, cases, and incidence and mortality rates between 1990 and 2019, by SDI quintile, globally, and for seven GBD super-regions and 21 GBD regions

		**Mortality**				**Incidence**				**Percentage change (1990–2019)**			
		1990 counts	Mortality rate (per 100 000), 1990	2019 counts	Mortality rate (per 100 000), 2019	1990 counts	Incidence rate (per 100 000), 1990	2019 counts	Incidence rate (per 100 000), 2019	Mortality	Mortality rate	Incidence	Incidence rate
Global		30 600 (26 600 to 36 200)	296·7 (257·2 to 350·9)	14 000 (11 200 to 18 200)	137·2 (109·5 to 177·8)	134 000 (96 300 to 182 000)	1301·2 (932·4 to 1767·2)	87 300 (62 300 to 121 000)	854·8 (609·2 to 1183·3)	−54·2% (−63·1 to −42·4)	−53·8% (−62·8 to −41·8)	−35·0% (−36·5 to −33·4)	−34·3% (−35·8 to −32·7)
	Low SDI	13 700 (11 700 to 16 600)	778·1 (661·5 to 942·2)	9330 (7290 to 12 200)	329·3 (257·2 to 429·4)	57 800 (42 400 to 77 200)	3278·8 (2403·4 to 4381·1)	52 800 (38 200 to 72 100)	1863·6 (1346·4 to 2545·6)	−31·9% (−46·5 to −13·3)	−57·7% (−66·8 to −46·1)	−8·6% (−12·0 to −5·3)	−43·2% (−45·3 to −41·1)
	Low-middle SDI	9700 (7920 to 11 800)	342·3 (279·4 to 416·7)	3270 (2510 to 4330)	122·3 (94·0 to 161·8)	40 200 (28 600 to 55 100)	1419·6 (1007·7 to 1944·6)	22 000 (15 400 to 30 700)	822·4 (574·3 to 1149·1)	−66·3% (−75·2 to −53·3)	−64·3% (−73·7 to −50·5)	−45·3% (−47·1 to −43·3)	−42·1% (−44·0 to −39·9)
	Middle SDI	5510 (4700 to 6680)	169·3 (144·5 to 205·4)	1170 (930 to 1470)	43·0 (34·2 to 54·2)	26 000 (18 000 to 36 100)	798·3 (553·2 to 1110·4)	9550 (6530 to 13 700)	351·6 (240·3 to 502·6)	−78·8% (−83·6 to −72·7)	−74·6% (−80·3 to −67·3)	−63·2% (−64·6 to −62·0)	−56·0% (−57·6 to −54·5)
	High-middle SDI	1470 (1320 to 1690)	93·9 (83·9 to 107·4)	184 (152 to 225)	15·4 (12·7 to 18·8)	8150 (5730 to 11 500)	519·1 (364·9 to 730·6)	2240 (1540 to 3240)	186·7 (128·2 to 270·5)	−87·5% (−90·1 to −84·3)	−83·6% (−87·1 to −79·5)	−72·5% (−74·0 to −71·1)	−64·0% (−66·0 to −62·2)
	High SDI	210 (193 to 229)	23·4 (21·4 to 25·5)	48 (41 to 56)	6·1 (5·2 to 7·1)	2130 (1530 to 2990)	236·8 (170·4 to 332·4)	690 (487 to 1000)	87·7 (61·8 to 127·1)	−77·3% (−80·9 to −73·2)	−74·0% (−78·2 to −69·4)	−67·6% (−69·2 to −66·0)	−63·0% (−64·9 to −61·2)
Central Europe, eastern Europe, and Central Asia		653 (606 to 719)	131·2 (121·8 to 144·5)	66 (53 to 83)	16·6 (13·4 to 20·8)	3150 (2360 to 4200)	633·8 (474·2 to 843·4)	826 (595 to 1170)	208·0 (149·7 to 293·6)	−89·9% (−92·0 to −87·3)	−87·4% (−90·0 to −84·1)	−73·8% (−76·0 to −71·6)	−67·2% (−70·0 to −64·4)
	Central Asia	274 (244 to 309)	185·4 (165·3 to 209·2)	34 (26 to 48)	24·0 (18·1 to 33·2)	1400 (1100 to 1740)	948·8 (742·4 to 1182·4)	438 (326 to 598)	305·9 (227·7 to 417·6)	−87·4% (−90·7 to −82·1)	−87·0% (−90·4 to −81·6)	−68·7% (−72·6 to −64·0)	−67·8% (−71·8 to −62·9)
	Central Europe	110 (99 to 127)	86·7 (77·5 to 99·5)	8 (6 to 11)	10·2 (7·8 to 13·0)	469 (355 to 613)	368·1 (279·0 to 481·9)	92 (68 to 130)	112·9 (83·0 to 158·3)	−92·4% (−94·3 to −90·1)	−88·2% (−91·1 to −84·6)	−80·3% (−81·8 to −78·5)	−69·3% (−71·7 to −66·6)
	Eastern Europe	269 (243 to 312)	120·7 (108·9 to 140·2)	23 (19 to 28)	13·4 (10·8 to 16·4)	1280 (887 to 1820)	576·8 (398·6 to 819·2)	295 (201 to 435)	171·7 (116·7 to 252·9)	−91·4% (−93·7 to −89·3)	−88·9% (−91·8 to −86·2)	−77·0% (−78·8 to −75·6)	−70·2% (−72·5 to −68·4)
High income		275 (256 to 298)	28·8 (26·8 to 31·2)	58 (49 to 68)	6·8 (5·7 to 8·0)	2510 (1850 to 3450)	262·7 (193·5 to 360·8)	748 (537 to 1070)	87·4 (62·8 to 124·7)	−78·8% (−82·3 to −74·8)	−76·3% (−80·3 to −71·8)	−70·2% (−71·7 to −68·9)	−66·7% (−68·4 to −65·2)
	Australasia	8 (6 to 9)	32·1 (26·7 to 38·2)	2 (2 to 3)	8·5 (6·5 to 11·0)	74 (52 to 102)	306·8 (217·7 to 423·4)	35 (25 to 50)	126·2 (89·8 to 178·8)	−69·2% (−77·9 to −57·9)	−73·5% (−81·0 to −63·8)	−52·2% (−59·2 to −45·8)	−58·9% (−64·9 to −53·3)
	High-income Asia Pacific	39 (33 to 46)	25·8 (21·9 to 30·4)	3 (2 to 3)	2·6 (2·1 to 3·0)	401 (282 to 580)	267·3 (188·4 to 386·6)	88 (58 to 132)	83·3 (55·6 to 125·5)	−93·0% (−94·6 to −91·2)	−90·0% (−92·3 to −87·5)	−78·1% (−80·4 to −75·7)	−68·8% (−72·0 to −65·3)
	High-income North America	59 (51 to 69)	16·9 (14·6 to 19·9)	21 (19 to 24)	6·6 (5·9 to 7·5)	431 (275 to 651)	123·6 (78·9 to 186·8)	111 (72 to 159)	34·6 (22·6 to 49·6)	−64·0% (−70·2 to −56·5)	−60·7% (−67·5 to −52·6)	−74·3% (−76·2 to −71·5)	−72·0% (−74·1 to −68·9)
	Southern Latin America	71 (62 to 80)	89·0 (78·8 to 100·8)	9 (6 to 13)	12·5 (8·8 to 17·1)	391 (313 to 489)	493·2 (394·6 to 616·3)	101 (76 to 137)	137·6 (103·6 to 186·0)	−87·0% (−91·0 to −81·8)	−86·0% (−90·3 to −80·4)	−74·1% (−77·4 to −70·1)	−72·1% (−75·6 to −67·8)
	Western Europe	100 (90 to 111)	28·2 (25·6 to 31·4)	23 (18 to 28)	7·0 (5·6 to 8·5)	1210 (898 to 1670)	343·2 (254·1 to 471·7)	413 (300 to 587)	125·6 (91·2 to 178·6)	−77·0% (−82·0 to −71·3)	−75·3% (−80·6 to −69·1)	−66·0% (−68·3 to −63·7)	−63·4% (−66·0 to −61·0)
Latin America and Caribbean		1280 (1120 to 1490)	160·0 (139·4 to 185·6)	304 (207 to 419)	41·1 (28·0 to 56·6)	4830 (3550 to 6440)	601·9 (442·4 to 802·6)	1350 (970 to 1840)	182·7 (131·2 to 248·6)	−76·3% (−84·3 to −65·4)	−74·3% (−82·9 to −62·4)	−72·0% (−73·6 to −70·6)	−69·7% (−71·4 to −68·1)
	Andean Latin America	105 (83 to 137)	115·7 (91·7 to 150·4)	18 (13 to 27)	18·6 (12·8 to 26·7)	391 (296 to 505)	430·7 (325·4 to 556·1)	139 (103 to 184)	139·8 (103·9 to 185·0)	−82·4% (−88·7 to −73·9)	−83·9% (−89·7 to −76·2)	−64·4% (−68·0 to −60·7)	−67·5% (−70·8 to −64·2)
	Caribbean	440 (313 to 603)	644·4 (459·0 to 883·3)	209 (127 to 318)	339·8 (206·2 to 515·9)	760 (576 to 978)	1113·3 (843·2 to 1431·9)	418 (302 to 552)	678·0 (490·2 to 896·2)	−52·4% (−73·0 to −19·1)	−47·3% (−70·1 to −10·3)	−45·1% (−49·9 to −38·9)	−39·1% (−44·5 to −32·3)
	Central Latin America	384 (335 to 438)	102·6 (89·6 to 116·9)	52 (37 to 71)	15·6 (11·2 to 21·2)	1610 (1220 to 2090)	429·0 (325·0 to 557·7)	365 (272 to 489)	109·4 (81·5 to 146·5)	−86·4% (−90·6 to −81·3)	−84·8% (−89·4 to −79·1)	−77·2% (−78·6 to −75·4)	−74·5% (−76·0 to −72·5)
	Tropical Latin America	355 (305 to 416)	131·9 (113·4 to 154·5)	24 (19 to 30)	9·8 (7·6 to 12·4)	2070 (1420 to 2920)	770·4 (525·7 to 1083·0)	429 (288 to 633)	175·5 (117·6 to 259·0)	−93·3% (−95·0 to −91·3)	−92·6% (−94·5 to −90·4)	−79·3% (−81·5 to −77·9)	−77·2% (−79·6 to −75·7)
North Africa and Middle East		1850 (1450 to 2390)	209·9 (164·9 to 271·3)	389 (276 to 541)	42·3 (30·0 to 58·8)	6320 (4560 to 8450)	718·6 (517·7 to 960·7)	2820 (2020 to 3960)	306·9 (219·6 to 430·6)	−79·0% (−85·9 to −68·4)	−79·9% (−86·5 to −69·7)	−55·3% (−58·9 to −51·7)	−57·3% (−60·7 to −53·8)
South Asia		7820 (6420 to 9470)	292·4 (240·1 to 353·7)	3120 (2320 to 4230)	123·6 (92·1 to 167·8)	35 800 (24 900 to 50 300)	1339·3 (929·6 to 1879·0)	18 500 (12 500 to 26 400)	733·7 (497·6 to 1046·4)	−60·2% (−72·5 to −43·7)	−57·7% (−70·8 to −40·2)	−48·4% (−50·6 to −46·2)	−45·2% (−47·5 to −42·8)
Southeast Asia, east Asia, and Oceania		4160 (3290 to 5450)	145·2 (114·6 to 190·3)	627 (489 to 805)	30·8 (24·0 to 39·5)	16 900 (11 500 to 23 700)	589·9 (401·5 to 826·0)	4500 (3090 to 6300)	221·3 (151·7 to 309·6)	−84·9% (−89·1 to −79·3)	−78·8% (−84·7 to −70·9)	−73·4% (−74·4 to −72·1)	−62·5% (−64·0 to −60·7)
	East Asia	1400 (1150 to 1660)	73·5 (60·7 to 87·3)	128 (100 to 157)	10·9 (8·5 to 13·3)	6830 (4360 to 9950)	359·2 (229·5 to 523·7)	1000 (650 to 1450)	84·9 (55·3 to 122·8)	−90·8% (−93·2 to −88·1)	−85·2% (−89·1 to −80·8)	−85·4% (−86·2 to −84·5)	−76·4% (−77·8 to −75·0)
	Oceania	46 (30 to 68)	274·6 (181·5 to 408·8)	46 (26 to 72)	149·6 (83·0 to 233·0)	249 (183 to 329)	1488·1 (1092·8 to 1962·6)	285 (204 to 387)	916·6 (657·5 to 1247·7)	0·9% (−46·5 to 78·6)	−45·5% (−71·1 to −3·6)	14·1% (0·7 to 29·5)	−38·4% (−45·7 to −30·1)
	Southeast Asia	2720 (2000 to 3920)	286·3 (210·9 to 412·7)	453 (339 to 615)	54·7 (41·0 to 74·3)	9830 (6950 to 13 300)	1035·9 (732·0 to 1398·0)	3220 (2240 to 4470)	389·1 (270·2 to 540·3)	−83·3% (−88·7 to −75·1)	−80·9% (−87·1 to −71·4)	−67·3% (−68·8 to −65·6)	−62·4% (−64·2 to −60·5)
Sub-Saharan Africa		14 600 (12 200 to 18 400)	885·7 (738·5 to 1115·3)	9460 (7220 to 12 500)	344·0 (262·6 to 456·3)	64 800 (47 500 to 86 700)	3933·5 (2881·7 to 5263·3)	58 600 (42 500 to 80 100)	2130·9 (1546·7 to 2914·2)	−35·1% (−49·2 to −16·7)	−61·2% (−69·6 to −50·1)	−9·5% (−12·6 to −6·1)	−45·8% (−47·7 to −43·7)
	Central sub- Saharan Africa	1540 (1040 to 2220)	769·5 (523·4 to 1111·4)	750 (484 to 1200)	223·8 (144·5 to 359·0)	6620 (4810 to 8760)	3317·3 (2410·4 to 4389·0)	5560 (4060 to 7590)	1660·4 (1210·6 to 2265·6)	−51·1% (−70·1 to −17·4)	−70·9% (−82·2 to −50·8)	−15·9% (−25·6 to −7·0)	−49·9% (−55·7 to −44·7)
	Eastern sub- Saharan Africa	5990 (4930 to 7500)	900·9 (740·9 to 1127·4)	3000 (2230 to 4040)	282·6 (210·7 to 381·3)	24 300 (17 800 to 32 400)	3650·9 (2674·3 to 4876·7)	19 300 (13 900 to 26 900)	1819·8 (1309·6 to 2538·8)	−49·9% (−62·5 to −34·1)	−68·6% (−76·5 to −58·7)	−20·5% (−24·6 to −15·5)	−50·2% (−52·8 to −47·0)
	Southern sub- Saharan Africa	195 (155 to 242)	168·1 (134·2 to 209·1)	110 (79 to 157)	87·8 (63·0 to 125·0)	1060 (758 to 1450)	912·5 (654·7 to 1255·2)	729 (516 to 1020)	581·1 (411·3 to 814·2)	−43·4% (−60·9 to −14·7)	−47·7% (−63·9 to −21·3)	−31·0% (−35·4 to −26·7)	−36·3% (−40·3 to −32·4)
	Western sub- Saharan Africa	6870 (5600 to 8910)	1030·2 (840·1 to 1336·3)	5600 (4270 to 7490)	455·9 (347·7 to 609·3)	32 800 (24 200 to 43 700)	4924·8 (3634·3 to 6563·5)	33 000 (24 100 to 44 500)	2686·1 (1959·9 to 3622·4)	−18·4% (−37·6 to 7·3)	−55·7% (−66·2 to −41·8)	0·5% (−2·9 to 3·7)	−45·5% (−47·3 to −43·7)

Data in parentheses are 95% uncertainty intervals. Count data are presented to three significant figures and rates are presented to one decimal place. Neonates are defined as those aged 0–27 days. SDI=Socio-demographic Index. GBD=Global Burden of Diseases, Injuries, and Risk Factors Study.

**Figure 1:**
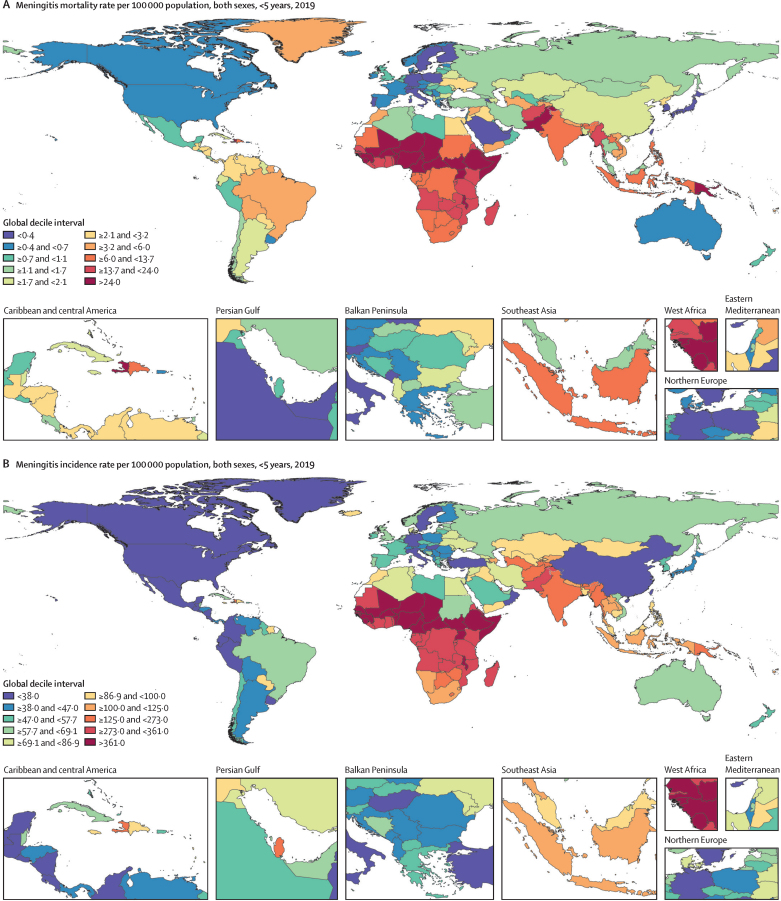
Meningitis mortality (A) and incidence (B) rates per 100 000 population among children younger than 5 years in 2019

In 2019, 236 000 deaths (95% UI 204 000–277 000) were attributable to meningitis globally (table 1), of which 112 000 (87 400–145 000) were in children younger than 5 years (table 2). Western sub-Saharan Africa had the highest age-standardised mortality rate (18·4 [15·0–22·1] per 100 000 population), followed by eastern sub-Saharan Africa (13·2 [11·5–15·1] per 100 000 population) and central sub-Saharan Africa (8·9 [6·8–11·2] per 100 000 population; table 1). Overall, the age-standardised mortality rate globally was 3·3 (2·8–3·9) per 100 000 population, down from 7·5 (6·6–8·4) per 100 000 population in 1990, representing a 56·0% (48·3–62·5) decrease. In 2019, rates varied from 0·1 (0·1–0·1) deaths per 100 000 population in Singapore to 26·3 (18·4–38·7) deaths per 100 000 population in Somalia (figure 1; appendix 2 pp 2–19). Worldwide in 2019, the mortality rate in children younger than 5 years (hereafter referred to as the under-5 mortality rate) due to meningitis was 16·9 (13·2–21·9) per 100 000 population, down from 45·0 (37·4–53·6) per 100 000 population in 1990, representing a 62·3% (52·1–70·6) decrease (table 2). Worldwide in 2019, the neonatal mortality rate due to meningitis was 137·2 (109·5–177·8) per 100 000 population, down from 296·7 (257·2–350·9) per 100 000 population in 1990, representing a 53·8% (41·8–62·8) decrease (table 3). More detailed results on meningitis incidence and mortality for all age groups by sex, country, and year are available online via the GBD Results Tool.

### Aetiology-specific results

In 2019, viral meningitis comprised 29·9% (95% UI 28·9–30·8) of total all-age meningitis cases, ranging regionally from 27·9% (26·9–29·0) in central sub-Saharan Africa to 37·4% (36·0–38·7) in the high-income Asia Pacific (appendix 2 pp 21–175). *N meningitidis* comprised 17·3% (16·5–18·0) of total cases, ranging from 8·5% (7·9–9·0) in Australasia to 21·4% (20·4–22·5) in central sub-Saharan Africa. *S pneumoniae* comprised 13·0% (12·4–13·6) of total cases, ranging from 10·4% (10·0–10·7) in eastern Europe to 14·7% (14·1–15·3) in Tropical Latin America. In the sub-Saharan African super-region, which has the highest incidence, viruses were estimated to be responsible for 28·6% (27·5–29·8) of cases, *N meningitidis* for 17·0% (15·8–18·1) of cases, and *S pneumoniae* for 13·6% (12·8–14·4) of cases. In 2019, the highest case burden in children younger than 5 years was attributable to viruses, comprising 25·1% (24·0–26·3) of total cases, followed by *N meningitidis* (16·9% [15·9–18·0]) and *S pneumoniae* (13·3% [12·5–14·0]). The highest case burden in neonates was attributable to viruses, comprising 37·1% (34·1–40·3) of total cases, followed by group B *Streptococcus* (20·4% [17·9–23·3]) and *N meningitidis* (9·7% [8·3–11·4]).

**Figure 2:**
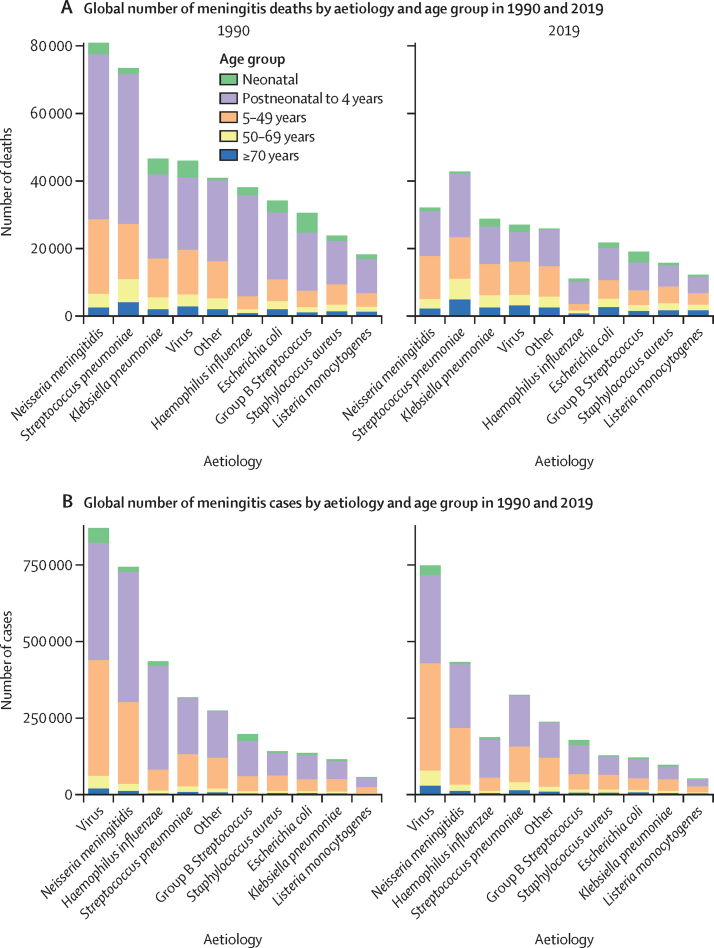
Global number of meningitis deaths (A) and cases (B) by aetiology and age group in 1990 and 2019

The largest proportion of total all-age meningitis deaths was attributable to *S pneumoniae*, comprising 18·1% (95% UI 17·1–19·2) of total all-age meningitis deaths, followed by *N meningitidis* (13·6% [12·7–14·4]) and *K pneumoniae* (12·2% [10·2–14·3]; figure 2; appendix 2 pp 21–175). In children younger than 5 years, the proportions of deaths due to *S pneumoniae*, *N meningitidis*, and *K pneumoniae* were similar to those for adults, but group B *Streptococcus* was responsible for a much larger fraction of deaths due to its high incidence proportion in neonates (appendix 2 pp 21–175). The largest proportion of deaths due to meningitis in 2019 in children younger than 5 years was attributable to *S pneumoniae*, comprising 17·3% (16·0–18·6) of total deaths due to meningitis in children younger than 5 years, followed by *N meningitidis* (12·9% [11·7–13·9]) and *K pneumoniae* (12·0% [9·7–14·8]). In 2019, the highest proportion of deaths due to meningitis in neonates was attributable to group B *Streptococcus* (22·8% [19·9–25·9]), followed by *K pneumoniae* (17·1% [13·6–21·1]) and viruses (15·3% [13·5–17·2]). Overall, 3200 (2420–4230) neonatal meningitis deaths were attributable to group B *Streptococcus* in 2019, down from 5920 (4870–7290) in 1990. Between 1990 and 2019, the largest reduction in deaths was seen from *H influenzae* among children younger than 5 years (76·5% [69·5–81·8]), followed by *N meningitidis* (72·3% [64·4–78·5]) and viruses (58·2% [47·1–67·3]; appendix 2 pp 176–77).

Between 1990 and 2019, the largest reduction in both mortality and incidence was for meningitis attributable to *H influenzae*. In 1990, 435 000 (95% UI 340 000–561 000) global all-age cases and 38 000 (31 500–45 400) global all-age deaths were attributable to *H influenzae* meningitis; of these, 354 000 cases (260 000–475 000) and 32 200 deaths (26 100–39 200) occurred in children younger than 5 years (appendix 2 pp 176–77). In 2019, there were 187 000 (151 000–234 000) all-age cases attributable to *H influenzae*, corresponding to a reduction of 56·9% (54·1–59·5), and 11 100 (9080–13 500) total deaths attributable to *H influenzae*, corresponding to a reduction of 70·8% (64·2–76·2; appendix 2 pp 176–77).

Between 1990 and 2019, the second largest reduction in mortality and incidence was for meningitis attributable to *N meningitidis*. In 1990, 744 000 (95% UI 608 000–902 000) all-age cases and 80 900 (69 300–95 100) all-age deaths were attributable to *N meningitidis* meningitis globally; of these, 442 000 (329 000–588 000) cases and 52 300 (42 200–64 300) deaths occurred in children younger than 5 years (appendix 2 pp 176–77). In 2019, there were 433 000 (361 000–518 000) all-age *N meningitidis* cases, corresponding to a reduction of 41·7% (38·4–44·8), and 32 100 (27 600–38 200) all-age deaths, corresponding to a reduction of 60·2% (52·7–66·4; appendix 2 pp 176–77).

### Meningitis belt results

A high burden of mortality and incidence is concentrated in the African meningitis belt, especially for children younger than 5 years. In 2019, there were 70 600 (95% UI 52 700–93 200) under-5 deaths attributed to meningitis in the meningitis belt, corresponding to 62·9% (57·5–67·8) of under-5 meningitis deaths globally; there were 637 000 (480 000–843 000) under-5 incident cases in the meningitis belt, corresponding to 50·0% (48·1–51·7) of under-5 meningitis cases globally (appendix 2 pp 178–79).

In 2019, age-standardised mortality rates in the meningitis belt ranged from 1·9 (95% UI 1·3–2·5) per 100 000 population in Sudan to 24·5 (17·5–32·9) per 100 000 population in Niger and 25·4 (19·0–33·3) per 100 000 population in Mali. Incidence rates ranged from 14·0 (11·9–16·4) per 100 000 population in Sudan to 257·1 (221·2–297·7) per 100 000 population in South Sudan (appendix 2 pp 2–19).

Even in the meningitis belt, which is at high risk of epidemics of meningococcal meningitis, *S pneumoniae* was responsible for the largest proportion of meningitis-related mortality in 2019, comprising 18·5% (95% UI 17·2–19·8) of total all-age meningitis deaths (121 000 [98 600–149 000]; appendix 2 pp 178, 180). The second largest proportion was attributed to *N meningitidis*, comprising 12·6% (11·4–13·8) of total all-age meningitis deaths. The all-age mortality rate due to *S pneumoniae* meningitis dropped in the meningitis belt, from 6·5 (5·3–8·0) per 100 000 population in 1990 to 2·6 (2·1–3·2) per 100 000 population in 2019, representing a 60·0% (50·6–68·0) decrease. The all-age mortality rate due to *N meningitidis* decreased from 7·4 (6·0–9·0) per 100 000 population in 1990 to 1·7 (1·4–2·2) per 100 000 population in 2019, representing a 76·2% (69·7–81·1) decrease (appendix 2 p 180).

Among children younger than 5 years, the largest proportion of meningitis-related mortality was attributed to *S pneumoniae* in the meningitis belt in 2019, comprising 17·7% (95% UI 16·2–19·2) of total under-5 meningitis deaths (70 600 [52 700–93 200]; appendix 2 pp 178, 180). The second largest proportion was attributed to *K pneumoniae*, comprising 12·0% (9·5–14·9) of total under-5 meningitis deaths. The under-5 meningitis mortality rate due to *S pneumoniae* dropped from 23·4 (17·9–30·4) per 100 000 population in 1990 to 9·2 (6·7–12·5) per 100 000 population in 2019, representing a 60·5% (47·6–70·8) decrease. Mortality rates due to *K pneumoniae* among children younger than 5 years decreased from 14·4 (10·0–20·2) per 100 000 population in 1990 to 6·2 (4·2–8·7) per 100 000 population in 2019, representing a 56·6% (42·4–67·7) decrease (appendix 2 p 180).

## Discussion

This study presents what are to our knowledge the most comprehensive estimates of the meningitis burden attributable to a comprehensive set of pathogens by age group across countries, contributing to our understanding of the pathogen-specific burden of meningitis. In 2019, the highest proportion of meningitis deaths both in all age groups and in children younger than 5 years was attributable to *S pneumoniae*, followed by *N meningitidis* and *K pneumoniae*, and the highest proportion of deaths in neonates was attributable to group B *Streptococcus*. *S pneumoniae*, *N meningitidis*, and *H influenzae* are the three pathogens responsible for the most meningitis-related deaths among children younger than 5 years, and there have been large reductions in the number of deaths attributable to all three pathogens since 1990.

A reduction in the burden of these pathogens could be attributed to successful vaccination rollouts over the past 30 years, with the highly effective Hib, pneumococcal, and meningococcal conjugate vaccines playing a key role.^26^ To achieve the goals of the WHO global roadmap to reduce cases of vaccine-preventable bacterial meningitis by 50% and deaths by 70% by 2030, continued vaccination against these pathogens is essential.^13^ Progress towards the roadmap goal of eliminating meningitis epidemics, which most commonly occur in the meningitis belt, is largely attributable to the rollout of vaccination programmes: specifically, the highly successful MenAfriVac campaign.^27^ An analysis of enhanced surveillance data from nine countries in the meningitis belt that completed MenAfriVac campaigns from 2005 to 2015 estimated that confirmed cases of *N meningitidis* serogroup A had declined by more than 99%.^27^ Our modelled results for meningococcus, which include serogroup A and other serogroups not covered by the vaccine, estimate a 41·7% decline in cases. A key factor in the successful rollout of MenAfriVac is its much lower cost than other comparable conjugate vaccines, improving the feasibility of mass rollout and sustainable vaccination.^27^, ^28^, ^29^

Although MenAfriVac campaigns successfully reduced the incidence rate of *N meningitidis* serogroup A in the meningitis belt, both sporadic and epidemic cases due to serogroups W, X, and C have increased.^30^, ^31^ Niger, in particular, began reporting an increase in both the number and proportion of serogroup W cases in the same year as the introduction of MenAfriVac.^32^ This observation underscores the need for an affordable multivalent meningococcal vaccine.^33^ Serotype replacement is also an issue for *S pneumoniae*: serotypes not covered by the 13-valent pneumococcal conjugate vaccine (PCV13), such as 12F and 23B, have resulted in a relative increase in the incidence of invasive disease in the post-vaccine era.^34^ The fact that meningitis is multipathogenic and that only some of the pathogens are currently vaccine preventable might have contributed to a slower decline in under-5 mortality rates for meningitis between 1990 and 2019 (62·3% [95% UI 52·1–70·6]) compared with diseases that are caused by a vaccine-preventable single pathogen, such as measles (90·5% [87·6–93·0]) and tetanus (91·9% [86·3–94·2]).^17^

For the first time, we have quantified the global burden of meningitis attributable to *K pneumoniae* and have identified this pathogen as the third leading cause of meningitis mortality after *S pneumoniae* and *N meningitidis*. Unlike *S pneumoniae* and *N meningitidis*, which are the most common causes of community-acquired meningitis, *K pneumoniae* meningitis is usually hospital-acquired and has higher mortality rates, especially in patients older than 60 years with comorbidities.^1^, ^35^, ^36^, ^37^ A systematic review found that *K pneumoniae* is the leading cause of Gram-negative meningitis and bacteraemia in low-income and middle-income countries.^38^ Despite its high fatal burden, *K pneumoniae* is not included as a priority pathogen in WHO's meningitis global roadmap,^13^ possibly because of a scarcity of global data on meningitis due to *K pneumonaie* before this study. *K pneumoniae* is also not included in most commercially available PCR or rapid diagnostic tests.^39^ Developing rapid diagnostic tests to detect this pathogen would improve tracking of this disease and its high burden.

The emerging shift in the cause of bacterial meningitis mortality to *K pneumoniae* emphasises the need for better control of hospital-acquired infections and antimicrobial stewardship. Many of these *K pneumoniae* strains have been reported to possess broad and threatening antimicrobial resistance genes, including extended-spectrum beta-lactamase with carbapenemase genes,^40^, ^41^ and these carbapenem-resistant strains are classified as critical on the WHO global priority antimicrobial resistance pathogen list.^42^ Many vaccines for *K pneumoniae* are in development and in clinical trials, with one of the most promising being multicomponent conjugate vaccines.^42^ These new vaccines might be able to protect against the multitude of infectious syndromes and antimicrobial resistance that *K pneumoniae* is known to produce.^43^ The rollout of Hib and *S pneumoniae* conjugate vaccines has also been shown to reduce the need for antibiotic use and helped slow the development of antimicrobial resistance.^44^

Group B *Streptococcus* comprises the highest proportion of neonatal meningitis deaths, although the absolute number of meningitis deaths due to group B *Streptococcus* in neonates has decreased from 5920 (95% UI 4870–7290) in 1990 to 3200 (2420–4230) in 2019. Unlike the key pathogens responsible for post-neonatal childhood meningitis, group B *Streptococcus* is not vaccine preventable. Instead, prevention of neonatal group B *Streptococcus* currently relies on prenatal testing of the mother and intrapartum antibiotic prophylaxis.^45^ A global meta-analysis found that the risk of early-onset group B *Streptococcus* in neonates was 1·1% in settings without an intrapartum antibiotic prophylaxis policy, and 0·3% in settings with an intrapartum antibiotic prophylaxis policy.^46^ An analysis of global intrapartum antibiotic prophylaxis policies in 90 countries found that 40 of 44 high-income countries had a policy, while only three of 20 countries in sub-Saharan Africa and one of three in east Asia reported having such a policy.^47^ Intrapartum antibiotic prophylaxis does not protect against late-onset group B *Streptococcus* disease occurring in infants aged 7–89 days,^48^, ^49^ which is responsible for about a third of the total neonatal group B *Streptococcus* cases. A maternal vaccine against group B *Streptococcus* might therefore be a solution, protecting against both early-onset and late-onset group B *Streptococcus*, and preventing frequent antibiotic use that can be associated with the development of antimicrobial resistance.^50^ Such a vaccine is currently a WHO priority and is predicted to be a cost-effective, financially sustainable, and feasible intervention.^51^ In addition to vaccination, reducing rates of short gestation and low birthweight might play an important role in preventing early-onset group B *Streptococcus*.^45^

Improvements to laboratory systems for both patient treatment and population surveillance are other key pillars in the WHO meningitis roadmap.^13^, ^39^ Specific microbial diagnosis guides appropriate treatment, including antibiotic selection. In the meningitis belt, population-level microbial surveillance enables the timely rollout of reactive vaccination campaigns against meningococcal strains other than serogroup A.^22^, ^52^, ^53^ One such strategy to improve laboratory systems is to invest in infrastructure that reduces the transportation time of CSF samples and to reinforce safe-handling procedures between collection facilities and laboratories.^54^, ^55^ Another strategy is the development and rollout of next-generation rapid diagnostic tests (RDTs). Their low cost and ease of operation accelerate the diagnosis of meningitis in low-resource settings, substantially improving patient care, surveillance, and outbreak response;^39^ development of next-generation RDTs is therefore a WHO priority.^56^ However, downsides of existing RDTs include the absence of many pathogens in the test, the inability to distinguish between serotypes, and the inability to assess for antimicrobial resistance.^39^, ^57^ As the prevalence of antimicrobial resistance rises, especially in non-vaccine-preventable pathogens such as *K pneumoniae*, the need for global antimicrobial resistance surveillance systems grows. The WHO Global Antimicrobial Resistance and Use Surveillance System (GLASS),^58^ which provides a standardised approach to collection, analysis, and sharing of clinical antimicrobial resistance data for surveillance, is expanding. In 2020, 251 surveillance sites in the WHO African region, where meningitis poses the highest burden, submitted data to GLASS, up from 35 surveillance sites in 2017.^58^ The concurrent expansion of next-generation RDTs for point-of-care decision making, and enhanced microbial surveillance systems, including antimicrobial resistance surveillance to support evidence-based antibiotic use, can help improve outcomes for patients with infectious meningitis globally.

Our estimates of meningitis mortality in children younger than 5 years differ from those produced by other groups, both for the overall burden and for the pathogen-specific burden.^59^ The 2017 WHO and Maternal and Child Epidemiology Estimation Group (WHO-MCEE)^59^ estimate for global under-5 deaths due to meningitis and encephalitis was 142 841, which is lower than, but within the uncertainty interval of, the GBD 2019 estimate published previously for meningitis and encephalitis combined in the same year (143 000 [95% UI 115 000–179 000];^17^ WHO-MCEE combines meningitis and encephalitis, whereas GBD models them as mutually exclusive causes). A 2018 global systematic review by Oordt-Speets and colleagues^7^ of 56 published studies found that *S pneumoniae* and *N meningitidis* were the most common all-age bacterial meningitis pathogens in all regions, with mean case proportions ranging from 25·1% to 41·2% for *S pneumoniae* and from 9·1% to 36·2% *N meningitidis*, across regions. Here, we estimate that *N meningitidis* comprised 17·3% (16·5–18·0) of global all-age meningitis cases in 2019, ranging from 8·5% in Australasia to 21·4% in central sub-Saharan Africa, while *S pneumoniae* comprised 13·0% (12·4–13·6) of global all-age meningitis cases in the same year, ranging from 10·4% in eastern Europe to 14·7% in Tropical Latin America (appendix 2 pp 21–175). Additionally, the systematic review by Oordt-Speets and colleagues^7^ estimated that, in the African region, which had the most all-age data sources, *S pneumoniae* was responsible for 41·2% (34·1–48·4) of bacterial meningitis cases and *N meningitidis* was responsible for 36·2% (26·6–46·4) of bacterial meningitis cases. In our study, in the sub-Saharan African region, *S pneumoniae* was estimated to be responsible for 13·6% (12·8–14·4) of cases and *N meningitidis* was estimated to be responsible for 17·0% (15·8–18·1) of cases. Part of the reason for this discrepancy is that the previous systematic review only included 56 articles published between April 25, 2012, and April 25, 2017, whereas we include scientific literature published between 1990 and 2019, in addition to non-literature sources, such as multiple cause of death data and laboratory data. Owing to the historical importance and vaccine-preventable nature of *S pneumoniae, N meningitidis, and H influenzae,* many literature studies look specifically for these pathogens through rapid tests and assays that are not used for other pathogens, thus leading to a potential over-representation of these pathogens in literature.

This study has some limitations. First, the estimates of the overall meningitis and aetiology-specific burden are limited by data availability and quality, especially in areas of the meningitis belt where the burden is the greatest in settings with low health-care access. Even when data are available, heterogeneous data sources (eg, surveillance versus inpatient data) might not be directly comparable. We applied a standardised approach to adjust for systematic bias among different data sources before modelling. Locations with sparse or no data must rely on covariates and regional trends to predict estimates. Additionally, many of these locations rely on verbal autopsy data. A multisite validation study by Lozano and colleagues^60^ shows that verbal autopsy of meningitis has only modest performance. Strong, population-based surveillance systems are preferred and needed to drive response, inform case management, assess vaccine impact, and track progress. Second, we directly apply meningitis aetiology proportions from the global burden of antimicrobial resistance study to GBD estimates of meningitis cases and deaths, even though the two studies have slightly different definitions of meningitis. More specifically, for fatal cases, the GBD definition of meningitis includes only instances in which meningitis was the underlying cause of death, whereas the antimicrobial resistance study definition includes any instance where meningitis was present in the causal chain, regardless of the underlying cause of death. Third, meningitis cases due to *Mycobacterium tuberculosis* or HIV-associated opportunistic infections, including *Cryptococcus neoformans,* were not included in the present study, as GBD classifies them with the underlying diseases tuberculosis and HIV, respectively. Fourth, due to the poor specificity of data documenting cases of viral meningitis, we modelled all viral aetiologies collectively, rather than distinguishing individual viruses of scientific interest, such as enteroviruses and herpes viruses. Fifth, we did not explicitly search for data sources that used molecular methods such as genome sequencing to identify viral pathogens, but such sources could be emphasised in a future systematic review. Sixth, we assumed the distribution of cases of meningitis with unknown aetiology (ie, those not identified through physician diagnosis or by microbiological means as either viral meningitis or a specified bacteria) was the same as the distribution of meningitis cases in which the aetiology was defined. This assumption could be violated if certain pathogens are more difficult to detect than others, or in cases where a pathogen is irregularly tested for within a laboratory. Seventh, age-standardised estimates allow for comparisons between populations with potentially different age distributions, but they should not be interpreted as actual rates. Meningitis incidence and mortality estimates that are not age-standardised are available online via the GBD Results Tool described above. Eighth, we did not assess risk factors for meningitis, but these could be the subject of future research. Finally, presenting *K pneumoniae* results for all children younger than 5 years might mask its higher burden in children younger than 1 year. We plan to report results for more detailed age groups in future GBD rounds.

Our study presents the burden of meningitis and its aetiologies before the COVID-19 pandemic. Evidence suggests that social distancing associated with COVID-19 has resulted in a lower incidence of meningitis and other invasive infections attributable to selected pathogens transmitted via the respiratory route, including *S pneumoniae, N meningitidis,* and *H influenzae*, during the pandemic, while the incidence of group B *Streptococcus* remained unchanged.^61^, ^62^ However, data for other pathogens remain unavailable. At the same time, the COVID-19 pandemic disrupted delivery of vaccines in 2020.^63^ Although this disruption is unlikely to affect meningitis rates due to persistent herd immunity, growing vaccine hesitancy fuelled by the pandemic will pose a challenge to preventing and controlling meningitis in the years ahead.^64^ As data become available for more countries and more pathogens, we will be able to comprehensively quantify the indirect effects of the pandemic on the incidence of meningitis and its aetiologies in future rounds of GBD.

Although meningitis incidence and mortality rates have decreased globally since 1990, progress lags behind that for other vaccine-preventable diseases. Moreover, although increased vaccine coverage in low-income and middle-income countries might have driven reductions in the meningitis burden, the reduction is not equal across locations. There is a continued need for low-cost multivalent vaccines as a preventive measure and for epidemic control in the meningitis belt. Further strengthening laboratory capacity to diagnose meningitis accurately and rapidly will also assist in the control of epidemics. Countries outside the African meningitis belt with high meningitis burdens, such as those in south Asia, are also affected by these policy needs. Additional enhanced surveillance data will improve country-specific burden estimates, which will help track progress towards reducing the global burden of meningitis by 2030.

## Data sharing

To download the data used in these analyses, please visit the Global Health Data Exchange GBD 2019 website.

## Declaration of interests

K Akinosoglou reports payment or honoraria for lectures, presentations, speakers’ bureau fees, manuscript writing or educational events from Pfizer Hellas, Gilead Sciences, Merck Sharp and Dohme, Glaxosmithkline Greece, as payments to the University of Patras; and support for attending meetings or travel, or both, to Pfizer Hellas, Gilead Sciences, Merck Sharp and Dohme, Glaxosmithkline Greece, and Norma Hellas; all outside the submitted work. R Ancuceanu reports payment or honoraria for lectures, presentations, speakers’ bureau fees, manuscript writing or educational events from AbbVie, Sandoz, B. Braun, and Laropharm, all outside the submitted work. S Bhaskar reports leadership or fiduciary roles in other board, society, committee or advocacy groups, paid or unpaid, with Rotary Club of Sydney, Australia as Board Director, with Rotary District 9675, Australia as Chair of Diversity Equity & Inclusion, and with Global Health Hub, Berlin as Founding Member/Chair & Co-Manager, Global Health and Migration Hub Community, all outside the submitted work. D Buonsenso reports payment or honoraria for lectures, presentations, speakers bureau fees, manuscript writing or educational events and participation on an advisory board from Pfizer for the Pneumococcal 22 Vaccine Advisory Board 2022, outside the submitted work. A Demetriades reports payment or honoraria for speakers’ bureau fees from Integra, Stryker, and Safe Orthopaedics; leadership or fiduciary roles, unpaid, as a board member (non-stipendiary) with the European Association of Neurosurgical Societies, Global Neuro Foundation and on the steering committee (non-stipendiary) of AO Spine Knowledge Forum Degenerative; all outside the submitted work. B D Gessner reports support for the present manuscript from Pfizer through salary payments; and stock or stock options through their employment with Pfizer; all outside the submitted work. N E Ismail reports a leadership or fiduciary role as an unpaid council member and bursar of the Malaysian Academy of Pharmacy, outside the submitted work. J J Jozwiak reports payment or honoraria for lectures, presentations, speakers’ bureau fees, manuscript writing or educational events from Novartis and Adamed as personal payments, outside the submitted work. K Krishan reports non-financial support from the UGC Centre of Advanced Study, CAS II, Department of Anthropology, Panjab University, Chandigarh, India, outside the submitted work. A-F A Mentis reports grants or contracts from “MilkSafe: A novel pipeline to enrich formula milk using omics technologies”, a research co financed by the European Regional Development Fund of the European Union and Greek national funds through the Operational Program Competitiveness, Entrepreneurship and Innovation, under the call RESEARCH-CREATE - -INNOVATE (project code: T2EDK-02222), as well as from ELIDEK (Hellenic Foundation for Research and Innovation, MIMS-860); payment for expert testimony as a peer-reviewer for Fondazione Cariplo, Italy; leadership or fiduciary roles in other boards, societies, committees or advocacy groups, paid or unpaid, by serving as Editorial Board Member for the journals *Systematic Reviews* and *Annals of Epidemiology*, and as Associate Editor for *Translational Psychiatry*; stocks in a family winery; and other financial or non-financial interests as a scientific officer with the BGI Group; all outside the submitted work. M J Postma reports stock or stock options from Health-Ecore (Zeist NL, 25%) and PAG BV (Groningen, NL, 100%) outside the submitted work. S Shrestha reports other financial interests in the School of Pharmacy at Monash University Malaysia by receiving the Graduate Research Merit Scholarship to pursue his PhD, outside the submitted work. L Silva reports grants or contracts from CENTRO-04-3559-FSE-000162, Fundo Social Europeu, outside the submitted work. J A Singh reports consulting fees from Crealta/Horizon, Medisys, Fidia, PK Med, Two Labs, Adept Field Solutions, Clinical Care Options, Clearview Healthcare Partners, Putnam Associates, Focus Forward, Navigant Consulting, Spherix, MedIQ, Jupiter Life Science, UBM, Trio Health, Medscape, WebMD, and Practice Point Communications, the National Institutes of Health, and the American College of Rheumatology; payment or honoraria for speakers’ bureau fees from Simply Speaking; support for attending meetings or travel from the steering committee of OMERACT; participation on a Data Safety Monitoring Board or Advisory Board with the US Food and Drug Administration Arthritis Advisory Committee; a leadership or fiduciary role in board, society, committee or advocacy group, paid or unpaid, with OMERACT as a steering committee member, with the Veterans Affairs Rheumatology Field Advisory Committee as Chair (unpaid), and with the UAB Cochrane Musculoskeletal Group Satellite Center on Network Meta-analysis and as editor and director (unpaid); stock or stock options in TPT Global Tech, Vaxart Pharmaceuticals, Aytu BioPharma, Adaptimmune Therapeutics, GeoVax Labs, Pieris Pharmaceuticals, Enzolytics, Seres Therapeutics, Tonix Pharmaceuticals, and Charlotte's Web Holdings, and previously owned stock options in Amarin, Viking, and Moderna Pharmaceuticals; all outside the submitted work. C Wright is an employee of Meningitis Research Foundation, which receives grants in support of their charitable objectives from GlaxoSmithKline, Pfizer, Sanofi Pasteur, Serum Institute, and the Tableau Foundation, outside the submitted work.
