# Supplementary material for: Global, regional, and national burden of meningitis and its aetiologies, 1990–2019: a systematic analysis for the Global Burden of Disease Study 2019
Source: Lancet Neurol. 2023 Aug;22(8):685–711. doi: 10.1016/S1474-4422(23)00195-3 (PMC10356620; doi:10.1016/S1474-4422(23)00195-3)
Supplement: Supplementary appendix 1 [file mmc1.pdf]

THE LANCET
Neurology

Supplementary appendix 1

This appendix formed part of the original submission and has been peer reviewed. We post it as supplied by the authors.

Supplement to: GBD 2019 Meningitis and Antimicrobial Resistance Collaborators. Global, regional, and national burden of meningitis and its aetiologies, 1990–2019: a systematic analysis for the Global Burden of Disease Study 2019. *Lancet Neurol* 2023; **22:** 685–711.

Appendix 1

"Global, regional, and national burden of meningitis and its aetiologies, 1990-2019: a systematic analysis for the Global Burden of Disease Study 2019"

This appendix provides further methodological detail and results for "Global, regional, and national burden of meningitis and its aetiologies, 1990-2019: a systematic analysis for the Global Burden of Disease Study 2019"

All the material in the paper itself is novel although it builds off previous GBD works. However, parts of the supplemental methods appendix include sections adapted from the GBD Capstones previously published in The Lancet[1] and previous IHME work on antimicrobial resistance.[2]

Table of Contents

Methods ... *3*
- Case Definition ... 3
- Cause of Death Modelling Strategy ... 3
- Modelling fatal meningitis ... 3
- Cause of Death Input data ... 4
- Non-Fatal Modelling strategy ... 8
- Non-Fatal Input data ... 9

Aetiology Estimation ... **11**
- Aetiologies Input Data ... 11
- Non-fatal Aetiologies Modelling Strategy ... 12
- Fatal Aetiology Modelling Strategy ... 14

Statement of GATHER Compliance ... **16**

References ... **18**

Author Contributions ... **19**
- Managing the overall research enterprise ... 19
- Writing the first draft of the manuscript ... 19
- Primary responsibility for applying analytical methods to produce estimates ... 19
- Primary responsibility for seeking, cataloguing, extracting, or cleaning data; designing or coding figures and tables ... 19
- Providing data or critical feedback on data sources ... 19
- Developing methods or computational machinery ... 20
- Providing critical feedback on methods or results ... 20
- Drafting the work or revising it critically for important intellectual content ... 22
- Managing the estimation or publications process ... 24

Methods

Case Definition

Meningitis is a disease caused by inflammation of the meninges, the protective membrane surrounding the brain and spinal cord, and is typically caused by an infection in the cerebrospinal fluid. Symptoms include headache, fever, stiff neck, and sometimes seizures. Included in the GBD modelling were cases meeting ICD-9 diagnostic criteria for meningitis due to bacteria or viruses (036, 036.0-036.3, 036.43, 036.6, 036.8, 036.81, 036.82, 036.89, 036.9, 047-047.4, 047.8-048, 048.2, 048.6, 049-049.2, 049.8-049.9, 320.0-320.5, 320.7-320.82, 320.89, 321-321.8, 322-322.2, 322.9, 320, 320.9) and ICD-10 diagnostic criteria for meningitis due to bacteria or viruses (A39-A39.9, A87-A87.9, and G00.0-G00.8).

Cause of Death Modelling Strategy

Appendix Figure 1: Flowchart of meningitis mortality estimation

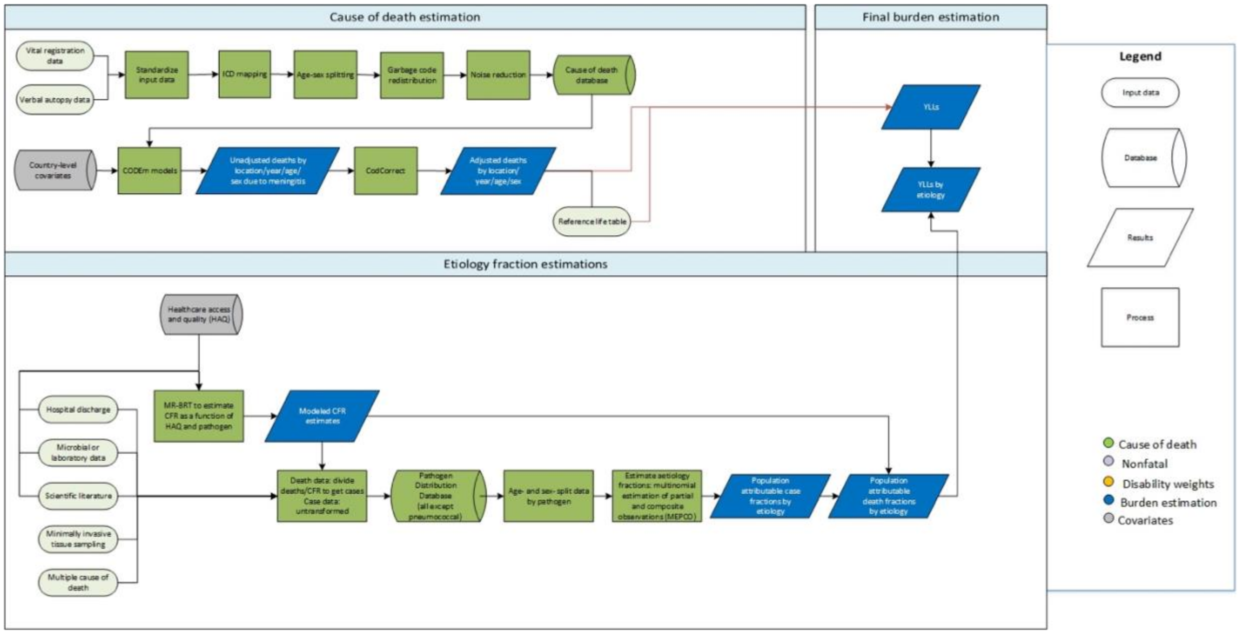

Modelling fatal meningitis

We modelled deaths due to all meningitis with two CODEm models, separately for each sex and two age categories – under 5 and 5 years and above. The mortality trends differ substantially between children and adults, and there are a significant number of data sources that only have data for children under 5. The two models used the same covariates (with the exception of the covariate for underweight, which is age-specific) and otherwise standard CODEm parameters. The final sex-specific models for deaths due to all meningitis were a hybridised model of separate global and data-rich models for males and females.

Appendix Table 2: Covariates used for meningitis cause-of-death ensemble modelling

Covariate Name	Level	Direction
Meningitis belt (proportion of population in belt)	1	+
MenAfriVac coverage	1	-
H. influenzae type B proportion covered	1	-
PCV3 coverage proportion	1	-
Age- and sex-specific summary exposure value (SEV) for child underweight	2	+
Logit-transformed water (proportion with access)	2	-
Maternal care and immunization	2	-
Healthcare Access and Quality Index	2	-
Log-transformed lag distributed income	3	-
Sanitation (proportion with access)	3	-
Maternal education (years per capita)	3	-
Socio-demographic Index	3	-

Cause of Death Input data

Input data for the overall meningitis model came from the cause of death database, which includes vital registration (VR) and verbal autopsy (VA) data. We outliered data in instances where garbage code redistribution and noise reduction, in combination with small sample sizes, resulted in unreasonable cause fractions when compared to regional, super-regional, and global rates, and data that violated well-established time or age trends. Outliering methods were consistent across both VR and VA data.

Appendix Table 3: International Classification of Disease codes used in estimating meningitis

ICD Version	ICD Code	Code Name
9	036	Meningococcal infection
	036.0	Meningococcal meningitis
	036.1	Meningococcal encephalitis
	036.2	Meningococcemia
	036.3	Waterhouse-Friderichsen syndrome, meningococcal
	036.43	Meningococcal myocarditis
	036.6	Meningococcal myocarditis
	036.8	Other specified meningococcal infections
	036.81	Meningococcal optic neuritis
	036.82	Meningococcal arthropathy
	036.89	Other specified meningococcal infections
	036.9	Meningococcal infection unspecified
	047	Meningitis due to enterovirus

	047.0	Meningitis due to coxsackie virus
	047.1	Meningitis due to echo virus
	047.2	ECHO virus
	047.3	ECHO virus
	047.4	ECHO virus
	047.8	Other specified viral meningitis
	047.9	Unspecified viral meningitis
	048	Other enterovirus diseases of central nervous system
	048.2	Other enterovirus diseases of central nervous system
	048.6	Other enterovirus diseases of central nervous system
	049	Other non-arthropod-borne viral diseases of central nervous system
	049.0	Lymphocytic choriomeningitis
	049.1	Meningitis due to adenovirus
	049.2	Meningitis due to adenovirus
	049.8	Other specified non-arthropod-borne viral diseases of central nervous system
	049.9	Unspecified non-arthropod-borne viral diseases of central nervous system
	320.0	Hemophilus meningitis
	320.1	Pneumococcal meningitis
	320.2	Streptococcal meningitis
	320.3	Staphylococcal meningitis
	320.4	Tuberculous meningitis
	320.5	Meningococcal meningitis
	320.7	Meningitis in other bacterial diseases classified elsewhere
	320.8	Meningitis due to other specified bacteria
	320.81	Anaerobic meningitis
	320.82	Meningitis due to gram-negative bacteria, not elsewhere classified
	320.89	Meningitis due to other specified bacteria
	321	Meningitis due to other organisms
	321.0	Cryptococcal meningitis
	321.1	Meningitis in other fungal diseases
	321.2	Meningitis due to viruses not elsewhere classified
	321.3	Meningitis due to trypanosomiasis
	321.4	Meningitis in sarcoidosis
	321.5	Meningitis due to mumps virus
	321.6	Meningitis due to lymphocytic choriomeningitis virus
	321.7	Meningitis due to other and unspecified viruses
	321.8	Meningitis due to other nonbacterial organisms classified elsewhere
	322	Meningitis of unspecified cause

	322.0	Nonpyogenic meningitis
	322.1	Eosinophilic meningitis
	322.2	Chronic meningitis
	322.9	Meningitis, unspecified
	320	Bacterial meningitis
	320.9	Meningitis due to unspecified bacterium
10	A39	Meningococcal infection
	A39.0	Meningococcal meningitis
	A39.1	Waterhouse-Friderichsen syndrome
	A39.2	Acute meningococcemia
	A39.3	Chronic meningococcemia
	A39.4	Meningococcemia, unspecified
	A39.5	Meningococcal heart disease
	A39.50	Meningococcal carditis, unspecified
	A39.51	Meningococcal endocarditis
	A39.52	Meningococcal myocarditis
	A39.53	Meningococcal pericarditis
	A39.8	Other meningococcal infections
	A39.81	Meningococcal encephalitis
	A39.82	Meningococcal retrobulbar neuritis
	A39.83	Meningococcal arthritis
	A39.84	Postmeningococcal arthritis
	A39.89	Other meningococcal infections
	A39.9	Meningococcal infection, unspecified
	A87	Viral meningitis
	A87.0	Enteroviral meningitis
	A87.1	Adenoviral meningitis
	A87.2	Lymphocytic choriomeningitis
	A87.8	Other viral meningitis
	A87.9	Viral meningitis, unspecified
	D86.81	Sarcoid meningitis
	G00.0	Hemophilus meningitis
	G00.1	Pneumococcal meningitis
	G00.2	Streptococcal meningitis
	G00.3	Staphylococcal meningitis
	G00.8	Other bacterial meningitis
	G03	Meningitis due to other and unspecified causes
	G03.0	Nonpyogenic meningitis
	G03.1	Chronic meningitis
	G03.2	Benign recurrent meningitis [Mollaret]

	G03.8	Meningitis due to other specified causes
	G00	Bacterial meningitis, not elsewhere classified
	G00.9	Bacterial meningitis, unspecified
	G01	Meningitis in bacterial diseases classified elsewhere
	G02	Meningitis in other infectious and parasitic diseases classified elsewhere
	G02.0	Meningitis in bacterial diseases classified elsewhere
	G02.1	Meningitis in bacterial diseases classified elsewhere
	G02.8	Meningitis in bacterial diseases classified elsewhere
	G03.9	Meningitis, unspecified

Non-Fatal Modelling strategy

Appendix Figure 3: Flowchart of meningitis non-fatal burden estimation

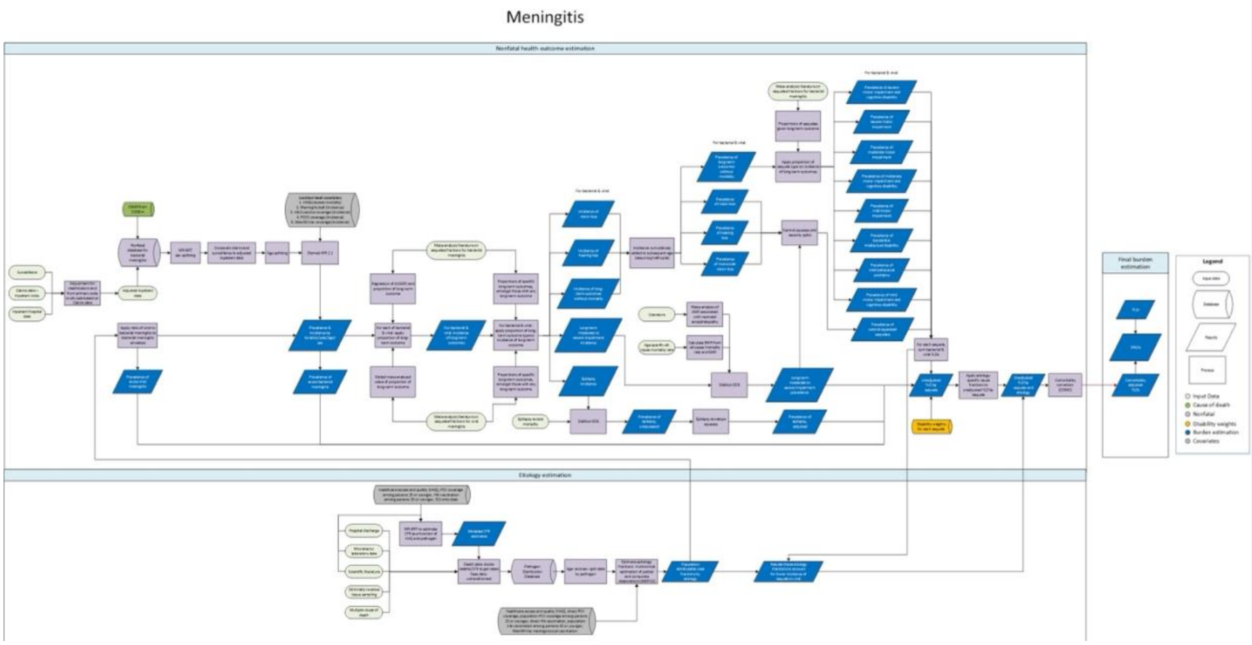

Non-fatal outcomes were modelled using a combination of custom models, DisMod-MR 2.1, and in GBD 2017, we added the use of an ordinary differential equations solver (ODE) for more timely and accurate estimates. First, the overall incidence and prevalence of bacterial meningitis were modelled to estimate the short-term morbidity due to acute infection. This DisMod model had a set duration (1/remission) of four weeks with a range ±2 weeks. We also imposed caps on excess mortality for neonates and elders based on the highest excess mortality estimates from GBD 2019. We used the function in DisMod-MR 2.1 to pull in cause-specific mortality rate (CSMR) data from our CODEm and CODcorrect analyses. We calculated excess mortality rate to estimate priors by dividing CSMR by prevalence, calculated from remission and incidence. To help inform trends where we lack data, we applied a country-level covariate for proportion of the population at the subnational and country levels that lives within the meningitis belt in sub-Saharan Africa (2). In GBD 2017 we added country-level covariates for coverage of Hib3 vaccine and the MenAfriVac vaccine initiative to the parent meningitis model. In GBD 2019, we added a country-level covariate for coverage of PCV3. We also outliered incidence input data points with zero cases that were pulling down final estimates. Betas and exponentiated values (which can be interpreted as an odds ratio) are shown in the tables below country-level covariates. To estimate total bacterial plus viral meningitis from the bacterial meningitis model, we used age- and sex-specific ratios between bacterial and viral cases from a combination of hospital data and USA claims data. These ratios were applied to bacterial meningitis incidence to estimate incidence and prevalence of viral meningitis and then both were summed to estimate total meningitis. In addition, we also modelled the long-term outcomes from bacterial meningitis infection.

Appendix Table 4: Summary of covariates used in the meningitis DisMod-MR meta-regression model

Covariate	Type	Parameter	Exponentiated beta (95% Uncertainty Interval)
Hib3 vaccine coverage	Country-level	Incidence	0.67 (0.65, 0.70)
PCV3 coverage	Country-level	Incidence	0.76 (0.75, 0.78)
Meningitis belt	Country-level	Incidence	7.28 (7.05, 7.39)
MenAfriVac initiative	Country-level	Incidence	0.14 (0.14, 0.14)
Healthcare Access and Quality index	Country-level	Excess mortality	0.998 (0.993, 0.999)

Non-Fatal Input data

Model inputs

In the GBD 2010 study, a systematic review of literature was conducted to capture studies of incidence and excess mortality rate for all bacterial meningitis cases. For each of the four aetiologies, literature included excess mortality rate, incidence, proportion, remission, and standardised mortality ratio. The inclusion criteria stipulated that: (1) the publication year must be between 1980 and 2010; (2) "caseness" was based on diagnoses by antigen test, blood test, cerebrospinal fluid test, polymerase chain reaction test, or latex agglutination test; (3) sufficient information must be provided on study method and sample characteristics to assess the quality of the study; and (4) study samples must be representative of the general population. No limitation was set on the language of publication. For GBD 2013, the search strategy was replicated to capture epidemiological studies published between 2010 and 2013. The search strategy was repeated in 2015 only to capture excess mortality. For GBD 2019, the search strategy was again replicated to capture epidemiological studies published between 2015 and 2019. The PubMed search terms were: ("meningitis"[MeSH Terms] OR "meningitis"[Title/Abstract]) AND ("incidence"[Title/Abstract] OR "incidence"[MeSH Terms]) AND (2015[Date – Publication] : 3000[Date – Publication]) NOT ("animals"[MeSH Terms] NOT "humans"[MeSH Terms])

Appendix Figure 4: Prisma Diagram

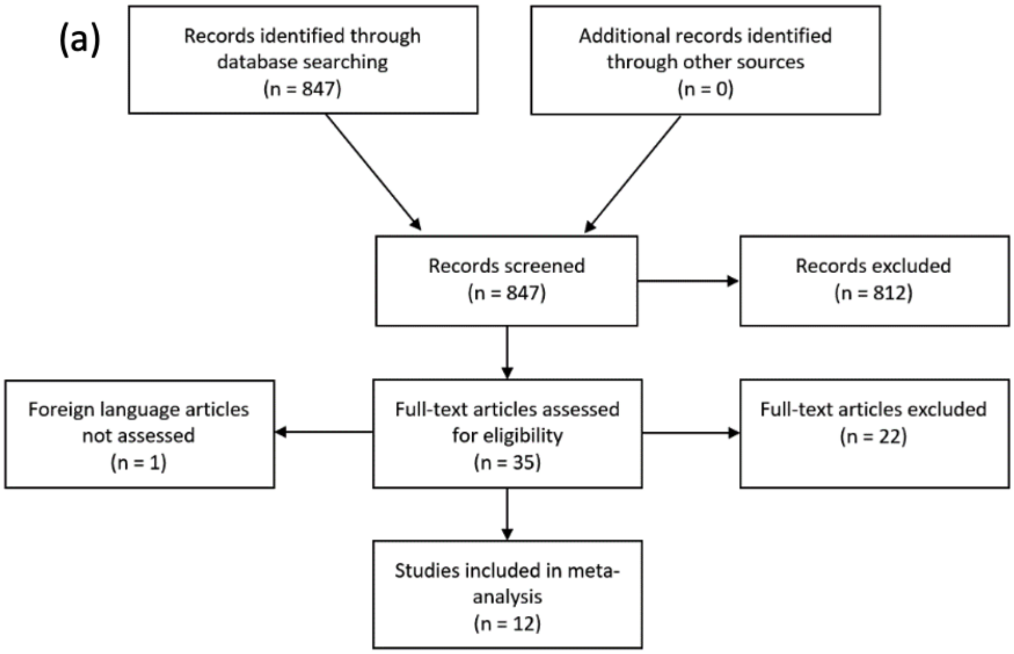

Additional sources we included in the acute bacterial meningitis model were surveillance data, inpatient-only hospital data and USA claims data from 2000, 2010, and 2012, 2015, primary diagnosis and inpatient only. Sequelae and severity splits were informed by a meta-analysis, Edmond and colleagues (1), while an internal meta-analysis informed mortality estimates for long-term moderate to severe impairments.

Bias corrections

Hospital data were flagged with a covariate for inpatient hospital data and was used as the reference category. Claims data were flagged with year-specific covariates. Both claims and surveillance data were crosswalked up to the reference category.

To inform the Marketscan crosswalk we used 1470 paired observations from Arizona, Colorado, Iowa, Maryland, New York, Washington, and Wisconsin. To inform the Marketscan data from 2000, we used 626 paired observations from Alaska, Arizona, Arkansas, California, Colorado, Florida, Iowa, Maryland, Michigan, Nevada, New Jersey, New York, North Carolina, Washington, and Wisconsin. To inform the surveillance data crosswalk, we used 1809 paired observations from 34 locations in High Income North America, Europe, and Latin America.

Appendix Table 5: Data Availability

Measure	Total sources	Countries with data
All measures	925	108
Incidence	349	68
Excess mortality rate	52	38
Case fatality rate	545	100
Proportion	57	39

Aetiology Estimation

Aetiologies Input Data

Input data for aetiology estimation consisted of multiple cause of death, vital registration, hospital discharge, and microbial data, as well as two independent, deduplicated, systematic literature reviews. For data sources that provided ICD codes (multiple cause of death, vital registration, hospital discharge, and some microbial data), these codes were used to identify patients with meningitis and the culprit pathogen, when detailed. For the microbial data that did not provide ICD codes, we identified pathogens associated with meningitis using cerebrospinal fluid samples. The table below documents the ICD codes used to identify meningitis cases with known aetiologies.

Appendix Table 6: ICD Codes Used in Aetiology Estimation

Type of meningitis	ICD 10 code(s)	ICD9 code(s)
Meningitis due to *Listeria monocytogenes*	A32.1	--
Meningitis due to *Neisseria meningitidis*	A39-A39.0	036-036.1, 320.5-320.8
Meningitis due to *Haemophilus influenzae*	G00.0	320.0
Meningitis due to *Streptococcus pneumoniae*	G00.1	320.1
Meningitis due to Group B *Streptococcus*	G00.2	320.2
Meningitis due to *Staphylococcus aureus*	G00.3	320.3
Meningitis due to virus	A87-A87.9, G03.0	047-049.9

Data on pathogens cultured from human infections were solicited from a wide array of international stakeholders (representing every inhabited continent). These included research hospitals, surveillance networks, and infection databases maintained by private laboratories and medical technology companies. For a full list of non-literature sources used for our estimates, please refer to the following article appendix (section 2).[2]

We estimated mutually-exclusive proportions of meningitis cases attributable to the following set of pathogens: *Escherichia coli,* group B *Streptococcus, Haemophilus influenzae, Klebsiella pneumoniae, Listeria monocytogenes, Neisseria meningitidis, Staphylococcus aureus, Streptococcus pneumoniae,* and viruses, as well as a residual, 'other pathogen' category. These proportions were estimated for five aggregate age groups: neonatal, post-neonatal to 5 years, 5 to 50 years, 50 to 70 years, and 70 years or older.

Consistent with methods we used to estimate aetiologies for 6 other infectious syndromes, for meningitis we dropped from the analysis all records where no pathogen was detected, or the patient diagnosis indicated an unspecified bacterium. This assumes that the distribution of pathogens among cases with known aetiology are the same as those with unknown aetiology; in other words, that the probability of detection is the same for every pathogen. This

assumption may break down if certain pathogens are more difficult to detect than others, or in cases where a pathogen is irregularly tested for within a laboratory.

Non-fatal Aetiologies Modelling Strategy

Aetiology proportions were calculated using an entirely new method from that applied in previous rounds of the GBD. Working from the assumption that aetiologies would follow a multinomial distribution, we estimated aetiology fractions using a method previously described as multinomial estimation of partial and composite observations (MEPCO). Due to the unique pattern of meningitis in neonates, particularly the high prevalence of group B *Streptococcus*, we modeled neonatal and adult meningitis aetiology proportions separately.

In order to use both partial and compositional data, we constructed a network model with the dependent variable as the log ratio of cases between different pathogens and estimated over a flexible parameterisation of multinomial parameters using a maximum likelihood approach. Consider a given infectious syndrome with a multinomial distribution of n mutually exclusive, collectively exhaustive aetiologies with probabilities $p = (p_1, \dots, p_n)$, so that each $p_j \in (0,1)$ and $\sum_j p_j = 1$. The likelihood of an observation of $c = (c_1, \dots, c_n)$, where c_j = number of cases of pathogen j in a total sample of N infections ($\sum_j c_j = N$), is:

$$P(c|p) = N! \prod_{j=1}^{n} \frac{p_j^{c_j}}{c_j!} \qquad (6.3.1.1)$$

We modelled the probabilities using a composition of a link function with a linear predictor:

$$p_{i,j} = \exp\left(x_{i,j}^T \beta_j\right) \qquad (6.3.1.2)$$

for observations i, a vector of covariates $x_{i,j}$, and a vector of coefficientsβ_j for each pathogen j. However, we did not observe these probabilities directly. Rather, we observed ratios between sums of these probabilities, which reduce to ratios between sums of cases within each study. These observations therefore take the form:

$$y_i = \frac{cases\ of\ pathogen\ A}{cases\ of\ pathogen\ B} = \frac{\sum_{j=1}^{n} w_{i,j}^a \exp\left(x_{i,j}^T \beta_j\right)}{\sum_{j=1}^{n} w_{i,j}^b \exp\left(x_{i,j}^T \beta_j\right)} \qquad (6.3.1.3)$$

where $w_{i,j}^a$ is a weight of 0 or 1 that selects the mutually exclusive, collectively exhaustive most-detailed pathogens that make up observed pathogen A, which may be a composite observation. For example, for the "other bacterial, non-GBS" pathogen, $w_{i,j}$ would be 1 for *Staphyloccocus aureus, S. pneumoniae, Haemophilus influenzae, Neisseria meningitidis, Listeria monocytogenes, K. pneumoniae, E. coli*, and other pathogens and 0 for GBS and virus. We dropped all observations where either the numerator or denominator had 0 observed cases in order to make this calculation and a forthcoming log transform possible. This may bias the model towards overestimating less common pathogens.

It is not possible to infer all coefficients β_j from the observations, since they are all relative. However, if we fix all of the coefficients for one pathogen to 0 as a reference group, then we obtain a well-posed inverse problem, as long as there is enough data to estimate the remaining coefficients. Without loss of generality, we assumed $\beta_1 = 0$ for all elements and obtain estimates of the remaining $\beta_2, \dots, \beta_n$ by minimising the sum of the residuals between log-transformed observations y and corresponding log-transformed predictions from equation 6.3.1.3:

$$\min_{\beta_2,\ldots,\beta_n} f(\beta) := \sum_i \frac{1}{\sigma_i^2}\left[\ln(y_i) - \ln\left(\sum_{j=1}^{n} w_{i,j}^a\, exp\left(x_{i,j}^T \beta_j\right)\right) + \ln\left(\sum_{j=1}^{n} w_{i,j}^b\, exp\left(x_{i,j}^T \beta_j\right)\right)\right]^2 \quad (6.3.1.4)$$

where σ_i^2 are variances corresponding to the data points. Equation 6.3.4 is a nonlinear likelihood minimisation problem that that we optimised using a standard implementation of the Gauss-Newton method.[3] We then re-normalised the optimal coefficients to obtain final predictions of the probabilities of each pathogen:

$$p_{i,j} = \frac{\exp\left(x_{i,j}^T \beta_j\right)}{\sum_{\hat{j}} \exp\left(x_{i,\hat{j}}^T \beta_{\hat{j}}\right)} \quad (6.3.1.5)$$

To quantify the uncertainty of this estimate, we used asymptotic statistics to obtain the posterior distribution of $(\beta_2, \ldots, \beta_n)$. Specifically, using the Gauss-Newton Hessian approximation gave us the asymptotic information matrix for all β_j except for the reference pathogen, allowing us to sample draws of $\beta = (\beta_1 = 0, \beta_2, \ldots, \beta_n)$. For each β draw and given feature x, we obtained a corresponding draw of p using equation 6.3.1.5.

This network regression with covariates framework allowed us to use partial and composite data that reported on one or only a few pathogens, or that reported multiple pathogens aggregated together. Networks, however, can be unstable with sparse data and stable estimates have in some cases required the use of Bayesian priors in these models. In particular, we imposed Gaussian priors with mean 0 and non-zero variance on all coefficients except intercepts, to bias the model away from spurious effects driven by data sparsity. For the neonatal model, a prior standard deviation of 0.2 was used. For the non-neonatal model, we used a standard deviation of 0.1. The table below indicates the set of covariates used in each of our non-fatal aetiology models.

Appendix Table 7: Non-fatal Aetiology Covariates

Covariate	Model
Age group (neonatal, post-neonatal to 5, 5 to 50, 50 to 70, 70 plus)	Non-neonatal
Healthcare Access and Quality Index	Neonatal, Non-neonatal
Proportion of people who as infants were vaccinated with PCV	Non-neonatal
Proportion of population age 15 or younger vaccinated against pneumococcus	Neonatal, Non-neonatal
Proportion of people who as infants were vaccinated against *Haemophilus influenzae* type B	Non-neonatal
Proportion of population age 15 or younger vaccinated against *Haemophilus influenzae* type B	Neonatal, Non-neonatal
Proportion of population covered by ’10-’15 MenAfriVac rollout for meningococcal meningitis	Neonatal, Non-neonatal

Fatal Aetiology Modelling Strategy

To generate aetiology fraction estimates for fatal meningitis, we took our aetiology fractions estimated for nonfatal meningitis and multiplied them by a set of pathogen-specific case fatality rates (CFRs). We defined the CFR as the proportion of cases in which the patient presented with meningitis and later died of that same case of meningitis (at any point in time). CFRs were estimated using ICD-coded hospital data, microbial data with patient discharge status, and a systematic literature review examining mortality associated with various pathogens. We employed the MR-BRT framework (meta-regression, Bayesian, regularized, trimmed) to predict CFRs as a function of pathogen, crude age (neonatal, post neonatal-5 years, 5-50 years, 50-70 years, and 70 years and older), HAQ Index, an interaction term between pathogen and the proportions of the population age 15 or younger that had received PCV and *Haemophilus influenzae* type B vaccinations[4], and a data source random effect. We additionally controlled for data provided from ICU-only sources (which would be biased towards higher CFRs). The general specification of the model is as follows:

$$logit(y_i) = X_i\beta + u_i 1 + \epsilon_i, \qquad \epsilon_i \sim N(0, \Sigma_i), \qquad u_i \sim N(0, \gamma)$$

where

- y_i contains CFRs for data source i
- Design matrix X_i contains as columns the model covariates
- β are fixed effect multipliers
- ϵ_i are observation error terms with known variances
- u_i are data source-specific random intercepts with unknown covariance γ

The modelling environment allows specification of priors on γ for which we used the following:

- Prior on β for pathogen:vaccination interaction: We assumed vaccination would have no impact on CFRs of unrelated pathogens, and for all combinations of the pathogen:vaccination interaction that were not *Streptococcus pneumoniae*:PCV vaccination or *Haemophilus influenzae*:Hib vaccination we coerced the βs to 0 using model priors. For the *Streptococcus pneumoniae*:PCV vaccination and *Haemophilus influenzae*:Hib vaccination interaction terms, we employed a negativity prior to enforce case-fatality rates for these pathogens to decrease as vaccination was introduced.
- Prior on γ, data source random effect: Many input data-sources cover only a single country, leading to low variability in HAQ Index within each data-source. To emphasize the contribution of HAQ Index over data-source in the modelled estimates, we implemented a strong Gaussian prior (mean 0, standard error 0.001) on γ.

There was significant variability with respect to the quality of input data we were able to collect for CFR estimation for different pathogens. For those pathogens with 'rich' data, defined by our method as having at least 10 high quality data points below a moderate HAQ Index (0.7), we modelled a unique effect of HAQ Index, achieved by interacting the HAQ Index fixed-effect with the pathogen-specific fixed-effect. This process allowed the relative deadliness of pathogens to vary depending on a location's HAQ Index. For those pathogens with fewer than 10 high quality data points below 0.7 HAQ Index, or those whose results in the interaction models indicated an unrealistically large influence of HAQ Index (e.g. 70% CFR in low HAQ Index countries, 1% CFR in high HAQ Index countries), we modelled a pathogen-specific intercept with an HAQ Index fixed-effect shared across the pathogens. As a consequence of the single fixed-effect on HAQ Index, a pathogen that was predicted to be the deadliest in low HAQ Index

countries would also be predicted to be the deadliest in high HAQ Index countries. The table below details which pathogens were modelled with each of the above methods.

Appendix Table 8: Modelling Strategy for Fatal Etiologies

Modelled with distinct intercept and effect of HAQi	Modelled with distinct intercept, pooled effect of HAQi
Haemophilus influenzae, *Streptococcus pneumoniae*, and viral meningitis	*Escherichia coli*, group B *Streptococcus*, *Klebsiella pneumoniae*, *Listeria monocytogenes*, *Neisseria meningitidis*, and *Staphylococcus aureus*

To estimate the CFRs for those infections associated with residual 'other pathogens', we pooled all bacterial data together and estimated a single CFR curve from age, HAQ Index, and the data source heterogeneity covariates. Nonfatal pathogen proportions $p_{i,j}$ for a given demographic group i and pathogen j were then converted to deaths using the CFRs estimates for demographic group i as follows:

$$p_{i,j}^{deaths} = \frac{p_{i,j} \times CFR_i}{\sum_{\hat{j}} p_{i,\hat{j}} \times CFR_i}$$

A more thorough account of these methods, including model validation, has been described previously elsewhere.[2]

Statement of GATHER Compliance

Appendix Table 9. Checklist of information that should be included in reports of global health estimates, with description of compliance and location of information for "Global, regional, and national burden of meningitis and its aetiologies, 1990—2019: a systematic analysis for the Global Burden of Disease Study 2019"

#	GATHER checklist item	Description of compliance	Reference
**Objectives and funding**			
1	Define the indicators, populations, and time periods for which estimates were made.	Narrative provided in paper and methods appendix describing indicators, definitions, and populations	Main text (Methods) and methods appendix
2	List the funding sources for the work.	Funding sources listed in paper	Summary (Funding)
**Data Inputs**			
For all data inputs from multiple sources that are synthesized as part of the study:			
3	Describe how the data were identified and how the data were accessed.	Narrative description of data seeking methods provided	Main text (Methods) and methods appendix
4	Specify the inclusion and exclusion criteria. Identify all ad-hoc exclusions.	Narrative about inclusion and exclusion criteria by data type provided	Main text (Methods) and methods appendix
5	Provide information on all included data sources and their main characteristics. For each data source used, report reference information or contact name/institution, population represented, data collection method, year(s) of data collection, sex and age range, diagnostic criteria or measurement method, and sample size, as relevant.	An interactive, online data source tool that provides metadata for data sources by component, geography, cause, risk, or impairment has been developed + a record showing what sources were used explicitly for this meningitis	Online data citation tool for the larger GBD 2019 and AMR analyses: http://ghdx.healthdata. org/gbd-2019 https://ghdx.healthdata.org/record/ihme-data/global-bacterial-antimicrobial-resistance-burden-estimates-2019<br><br>Record specifically showing input data for this manuscript: https://ghdx.healthdata.org/record/ihme-data/gbd-2019-meniningitis-by-pathogen-burden-estimates-1990-2019

		specific pathogen estimation and all-meningitis estimation	
6	Identify and describe any categories of input data that have potentially important biases (e.g., based on characteristics listed in item 5).	Summary of known biases by cause included in methods appendix	Main text (Methods) and Methods appendix
For data inputs that contribute to the analysis but were not synthesized as part of the study:			
7	Describe and give sources for any other data inputs.	Downloads of input data available through online tools, including data visualization tools for the larger GBD 2019 and AMR analyses	Online data citation tool for the larger GBD 2019 and AMR analyses: http://ghdx.healthdata. org/gbd-2019 https://ghdx.healthdata.org/record/ihme-data/global-bacterial-antimicrobial-resistance-burden-estimates-2019
For all data inputs:			
8	Provide all data inputs in a file format from which data can be efficiently extracted (e.g., a spreadsheet as opposed to a PDF), including all relevant meta-data listed in item 5. For any data inputs that cannot be shared due to ethical or legal	Downloads of input data available through online tools, including data visualization tools + a record showing what sources were used explicitly for this meningitis specific pathogen estimation and all-meningitis estimation	Online data citation tool for the larger GBD 2019 and AMR analyses: http://ghdx.healthdata. org/gbd-2019 https://ghdx.healthdata.org/record/ihme-data/global-bacterial-antimicrobial-resistance-burden-estimates-2019 Record specifically showing input data for this manuscript: https://ghdx.healthdata.org/record/ihme-data/gbd-2019-meningitis-by-pathogen-burden-estimates-1990-2019

References

1 Vos T, Lim SS, Abbafati C, *et al.* Global burden of 369 diseases and injuries in 204 countries and territories, 1990–2019: a systematic analysis for the Global Burden of Disease Study 2019. *The Lancet* 2020; **396**: 1204–22.

2 Murray CJ, Ikuta KS, Sharara F, *et al.* Global burden of bacterial antimicrobial resistance in 2019: a systematic analysis. *The Lancet* 2022; **399**: 629–55.

3 Numerical Optimization. Springer New York, 2006 DOI:10.1007/978-0-387-40065-5.

4 Galles NC, Liu PY, Updike RL, *et al.* Measuring routine childhood vaccination coverage in 204 countries and territories, 1980–2019: a systematic analysis for the Global Burden of Disease Study 2020, Release 1. *The Lancet* 2021; **398**: 503–21.

Author Contributions

Managing the overall research enterprise

Simon I Hay, Hmwe Hmwe Kyu, Christopher J L Murray, Mohsen Naghavi, Amanda Novotney, and Eve E Wool.

Writing the first draft of the manuscript

Rose G Bender, Hmwe Hmwe Kyu, Sarah Brooke Sirota, Avina Vongpradith, and Han Yong Wunrow.

Primary responsibility for applying analytical methods to produce estimates

Rose G Bender, Authia P Gray, Kevin S Ikuta, Fablina Sharara, Lucien R Swetschinski, and Han Yong Wunrow.

Primary responsibility for seeking, cataloguing, extracting, or cleaning data; designing or coding figures and tables

Authia P Gray, Fablina Sharara, Sarah Brooke Sirota, and Avina Vongpradith.

Providing data or critical feedback on data sources

Ashkan Abdollahi, Hassan Abidi, Richard Gyan Aboagye, Hassan Abolhassani, Qorinah Estiningtyas Sakilah Adnani, Saira Afzal, Bright Opoku Ahinkorah, Sajjad Ahmad, Ali Ahmed, Ayman Ahmed, Meqdad Saleh Ahmed, Addis Aklilu, Fares Alahdab, Tareq Mohammed Ali AL-Ahdal, Fahad Mashhour Alanezi, Ahmed Hassan Albelbeisi, Tsegaye Begashaw B Alemayehu, Kefyalew Addis Alene, Abid Ali, Beriwan Abdulqadir Ali, Liaqat Ali, Syed Shujait Ali, Syed Mohamed Aljunid, Sami Almustanyir, Nelson Alvis-Guzman, Jalal Arabloo, Judie Arulappan, Kendalem Asmare Atalell, Seyyed Shamsadin Athari, Alemu Degu Ayele, Ashish D Badiye, Sara Bagherieh, Atif Amin Baig, Shirin Barati, Mainak Bardhan, Zarrin Basharat, Buddha Basnyat, Neeraj Bedi, Melaku Ashagrie Belete, Rose G Bender, Akshaya Srikanth Bhagavathula, Sonu Bhaskar, Ajay Nagesh Bhat, Archith Boloor, Souad Bouaoud, Carlos A Castañeda-Orjuela, Vijay Kumar Chattu, Dinh-Toi Chu, Rosa A S Couto, Natália Cruz-Martins, Xiaochen Dai, Lalit Dandona, Rakhi Dandona, Fernando Pio De la Hoz, Demeke Dejen, Hiwot Dejene, Berecha Hundessa Demessa, Andreas K Demetriades, Hardik Dineshbhai Desai, Anteneh Mengist Dessie, Fikreab Desta, Thanh Chi Do, Masoud Dodangeh, Milad Dodangeh, Haneil Larson Dsouza, Oyewole Christopher Durojaiye, Michael Ekholuenetale, Maysaa El Sayed Zaki, Hassan El-Abid, Hawi Leul Esayas, Adeniyi Francis Fagbamigbe, Jawad Fares, Ali Fatehizadeh, Nicholas A Feasey, Desalegn Feyissa, Peter Andras Gaal, Muktar A Gadanya, Balasankar Ganesan, Getachew Muluye Gedef, Kazem Ghaffari, Reza Ghanbari, Ekaterina Vladimirovna Glushkova, Mahaveer Golechha, Habtamu Alganeh Guadie, Sapna Gupta, Vijai Kumar Gupta, Vivek Kumar Gupta, Arvin Haj-Mirzaian, Shafiul Haque, Harapan Harapan, Hadi Hassankhani, Simon I Hay, Nobuyuki Horita, Md Mahbub Hossain, Mehdi Hosseinzadeh, Salman Hussain, Nawfal R Hussein, Segun Emmanuel Ibitoye, Olayinka Stephen Ilesanmi, Nahlah Elkudssiah Ismail, Mihajlo Jakovljevic, Amirreza Javadi Mamaghani, Javad Javidnia, Charity Ehimwenma Joshua, Jacek Jerzy Jozwiak, Zubair Kabir, Himal Kandel, André Karch, Harkiran Kaur, Himanshu Khajuria, Imteyaz A Khan,

Maseer Khan, Khaled Khatab, Adnan Kisa, Sezer Kisa, Nikhil Kothari, Kewal Krishan, Manoj Kumar, Senthil D Kumaran, Hmwe Hmwe Kyu, Tri Laksono, Dennis Odai Laryea, Thao Thi Thu Le, Caterina Ledda, Sang-woong Lee, Seung Lee, Stephen S Lim, Xuefeng Liu, Ata Mahmoodpoor, Kashish Malhotra, Bernardo Alfonso Martinez-Guerra, Addisu Melese, Le Huu Nhat Minh, Awoke Misganaw, Arup Kumar Misra, Shafiu Mohammed, Ali H Mokdad, Sara Momtazmanesh, Lorenzo Monasta, Mohammad Ali Moni, Elias Mossialos, Ebrahim Mostafavi, Sumaira Mubarik, Jibran Sualeh Muhammad, Francesk Mulita, Christopher J L Murray, Ghulam Mustafa, Saravanan Muthupandian, Ahamarshan Jayaraman Nagarajan, Mohsen Naghavi, Shumaila Nargus, Zuhair S Natto, Biswa Prakash Nayak, Ionut Negoi, Ruxandra Irina Negoi, Hien Quang Nguyen, Phat Tuan Nguyen, Van Thanh Nguyen, Robina Khan Niazi, Hasti Nouraei, Khan M Nuruzzaman, Vincent Ebuka Nwatah, Chimezie Igwegbe Nzoputam, Ogochukwu Janet Nzoputam, Bogdan Oancea, Osaretin Christabel Okonji, Andrew T Olagunju, Amel Ouyahia, Mahesh P A, Keyvan Pakshir, Shahina Pardhan, Jay Patel, Urvish K Patel, Shankargouda Patil, Uttam Paudel, Shrikant Pawar, Marcos Pereira, Mario F P Peres, Zahra Zahid Piracha, Maarten J Postma, Vafa Rahimi-Movaghar, Zubair Ahmed Ratan, Salman Rawaf, Elrashdy Moustafa Mohamed Redwan, Tércia Moreira Ribeiro da Silva, Tamalee Roberts, Gisela Robles Aguilar, Jefferson Antonio Buendia Rodriguez, Victor Daniel Rosenthal, Basema Saddik, Umar Saeed, Narjes Saheb Sharif-Askari, Monalisha Sahu, Sara Samadzadeh, Abdallah M Samy, Maheswar Satpathy, Allen Seylani, Pritik A Shah, Masood Ali Shaikh, Sunder Sham, Mohammed Shannawaz, Pavanchand H Shetty, Jae Il Shin, Sunil Shrestha, Jasvinder A Singh, Robert Sinto, Anna Aleksandrovna Skryabina, Bogdan Socea, Yonatan Solomon, Sergey Soshnikov, Andy Stergachis, Rizwan Suliankatchi Abdulkader, Abida Sultana, Ker-Kan Tan, Pugazhenthan Thangaraju, Tala Tillawi, Marius Belmondo Tincho, Marcos Roberto Tovani-Palone, Paul Turner, Irfan Ullah, Bhaskaran Unnikrishnan, Shoban Babu Varthya, Avina Vongpradith, Theo Vos, Yasir Waheed, Mandaras Tariku Walde, Kosala Gayan Weerakoon, Melat Woldemariam, Han Yong Wunrow, Chalachew Yenew Yenew Yenew, Metin Yesiltepe, Siyan Yi, Muhammad Zaman, Iman Zare, Zahra Zareshahrabadi, Mikhail Sergeevich Zastrozhin, Haijun Zhang, Zhi-Jiang Zhang, Mohammad Zoladl, and Alimuddin Zumla.

Developing methods or computational machinery

Ashkan Abdollahi, Qorinah Estiningtyas Sakilah Adnani, Tareq Mohammed Ali AL-Ahdal, Aleksandr Y Aravkin, Judie Arulappan, Shirin Barati, Rose G Bender, Souad Bouaoud, Rosa A S Couto, Xiaochen Dai, Demeke Dejen, Hiwot Dejene, Hardik Dineshbhai Desai, Thanh Chi Do, Masoud Dodangeh, Milad Dodangeh, Hawi Leul Esayas, Ali Fatehizadeh, Desalegn Feyissa, Kazem Ghaffari, Mehdi Hosseinzadeh, Kevin S Ikuta, Amirreza Javadi Mamaghani, Adnan Kisa, Manoj Kumar, Thao Thi Thu Le, Sang-woong Lee, Le Huu Nhat Minh, Javad Moghadasi, Ali H Mokdad, Mohammad Ali Moni, Francesk Mulita, Christopher J L Murray, Mohsen Naghavi, Hien Quang Nguyen, Phat Tuan Nguyen, Van Thanh Nguyen, Robert C Reiner Jr, Umar Saeed, Abdallah M Samy, Maheswar Satpathy, Fablina Sharara, Lucien R Swetschinski, Theo Vos, Han Yong Wunrow, Mikhail Sergeevich Zastrozhin, and Peng Zheng.

Providing critical feedback on methods or results

Amirali Aali, Sherief Abd-Elsalam, Ashkan Abdollahi, Hassan Abidi, Richard Gyan Aboagye, Hassan Abolhassani, Lawan Hassan Adamu, Tigist Demssew Adane, Isaac Yeboah Addo, Oyelola A Adegboye, Tayo Alex Adekiya, Mohammad Adnan, Qorinah Estiningtyas Sakilah Adnani, Saira Afzal, Shahin Aghamiri, Antonella Agodi, Bright Opoku Ahinkorah, Aqeel Ahmad, Sajjad Ahmad, Mohadese Ahmadzade, Ali Ahmed, Ayman Ahmed, Jivan Qasim Ahmed, Meqdad Saleh Ahmed, Karolina Akinosoglou, Addis Aklilu, Maxwell Akonde, Fares Alahdab, Tareq Mohammed Ali AL-Ahdal, Fahad Mashhour Alanezi, Ahmed Hassan Albelbeisi, Tsegaye Begashaw B Alemayehu, Kefyalew Addis Alene, Ayman Al-Eyadhy, Adel Ali Saeed Al-Gheethi, Abid Ali, Liaqat Ali, Syed Shujait Ali, Yousef Alimohamadi, Vahid Alipour, Syed Mohamed Aljunid, Sami Almustanyir, Nelson Alvis-Guzman, Hany Aly, Edward Kwabena Ameyaw, Robert Ancuceanu, Adnan Ansar, Golnoosh Ansari, Anayochukwu Edward Anyasodor, Jalal Arabloo, Demelash Areda, Anton A Artamonov, Judie Arulappan, Raphael Taiwo Aruleba, Muhammad Asaduzzaman, Kendalem Asmare Atalell, Seyyed Shamsadin Athari, Daniel Atlaw, Maha Moh'd Wahbi Atout, Sameh Attia, Tewachew Awoke, Melese Kitu Ayalew, Tegegn Mulatu Ayana, Alemu Degu Ayele, Sina Azadnajafabad, Khalil Azizian, Muhammad Badar, Ashish D Badiye, Nayereh Baghcheghi, Sara Bagherieh, Saeed Bahadory, Atif Amin Baig, Aleksandra Barac, Shirin Barati, Mainak Bardhan, Zarrin Basharat, Quique Bassat, Saurav Basu, Nebiyou Simegnew Bayileyegn, Amir Hossein Behnoush, Abebe Ayalew Bekel, Melaku Ashagrie Belete, Olorunjuwon Omolaja Bello, Rose G Bender, Akshaya Srikanth Bhagavathula, Dinesh Bhandari,

Pankaj Bhardwaj, Sonu Bhaskar, Ajay Nagesh Bhat, Ali Bijani, Archith Boloor, Souad Bouaoud, Danilo Buonsenso, Katrin Burkart, Luis Alberto Cámera, Carlos A Castañeda-Orjuela, Achille Cernigliaro, Jaykaran Charan, Vijay Kumar Chattu, Hitesh Chopra, Sonali Gajanan Choudhari, Devasahayam J Christopher, Dinh-Toi Chu, Rosa A S Couto, Natália Cruz-Martins, Omid Dadras, Xiaochen Dai, Lalit Dandona, Rakhi Dandona, Saswati Das, Nihar Ranjan Dash, Mohsen Dashti, Fernando Pio De la Hoz, Sisay Abebe Debela, Demeke Dejen, Hiwot Dejene, Dessalegn Demeke, Feleke Mekonnen Demeke, Berecha Hundessa Demessa, Andreas K Demetriades, Solomon Demissie, Emina Dervišević, Hardik Dineshbhai Desai, Anteneh Mengist Dessie, Fikreab Desta, Kuldeep Dhama, Shirin Djalalinia, Thanh Chi Do, Masoud Dodangeh, Milad Dodangeh, Deepa Dongarwar, Haneil Larson Dsouza, Oyewole Christopher Durojaiye, Arkadiusz Marian Dziedzic, Michael Ekholuenetale, Temitope Cyrus Ekundayo, Maysaa El Sayed Zaki, Hassan El-Abid, Muhammed Elhadi, Victor Gabriel El-Hajj, Waseem El-Huneidi, Amro A El-Sakka, Hawi Leul Esayas, Adeniyi Francis Fagbamigbe, Shahab Falahi, Jawad Fares, Ali Fatehizadeh, Syeda Anum Fatima Fatima, Nicholas A Feasey, Ginenus Fekadu, Getahun Fetensa, Desalegn Feyissa, Florian Fischer, Peter Andras Gaal, Muktar A Gadanya, Abduzhappar Gaipov, Balasankar Ganesan, Mesfin Gebrehiwot, Kahsu Gebrekirstos Gebrekidan, Teferi Gebru Gebremeskel, Getachew Muluye Gedef, Urge Gerema, Motuma Erena Getachew, Keyghobad Ghadiri, Kazem Ghaffari, Reza Ghanbari, Ramy Mohamed Mohmaed Ghazy, Admasu Belay AB Gizaw, Ekaterina Vladimirovna Glushkova, Mohamad Goldust, Mahaveer Golechha, Habtamu Alganeh Guadie, Rashid Abdi Guled, Sapna Gupta, Vijai Kumar Gupta, Vivek Kumar Gupta, Najah R Hadi, Arvin Haj-Mirzaian, Sebastian Haller, Samer Hamidi, Shafiul Haque, Harapan Harapan, Ahmed I Hasaballah, Ikramul Hasan, Hamidreza Hasani, Mohammed Bheser Hassen, Simon I Hay, Khezar Hayat, Mohammad Heidari, Mahsa Heidari-Foroozan, Reza Heidari-Soureshjani, Kamal Hezam, Ramesh Holla, Nobuyuki Horita, Md Mahbub Hossain, Mohammad-Salar Hosseini, Mehdi Hosseinzadeh, Salman Hussain, Nawfal R Hussein, Segun Emmanuel Ibitoye, Kevin S Ikuta, Olayinka Stephen Ilesanmi, Irena M Ilic, Milena D Ilic, Mohammad Tarique Imam, Kenneth Chukwuemeka Iregbu, Nahlah Elkudssiah Ismail, Chidozie C D Iwu, Mihajlo Jakovljevic, Elham Jamshidi, Amirreza Javadi Mamaghani, Javad Javidnia, Mohammad Jokar, Nabi Jomehzadeh, Nitin Joseph, Charity Ehimwenma Joshua, Jacek Jerzy Jozwiak, Zubair Kabir, Laleh R Kalankesh, Rohollah Kalhor, Vineet Kumar Kamal, Himal Kandel, Ibraheem M Karaye, André Karch, Hanie Karimi, Harkiran Kaur, Navjot Kaur, Mohammad Keykhaei, Himanshu Khajuria, Amirmohammad Khalaji, Ajmal Khan, Imteyaz A Khan, Maseer Khan, Taimoor Khan, Khaled Khatab, Moawiah Mohammad Khatatbeh, Hamid Reza Khayat Kashani, Jagdish Khubchandani, Min Seo Kim, Adnan Kisa, Sezer Kisa, Farzad Kompani, Hamid Reza Koohestani, Nikhil Kothari, Kewal Krishan, Yuvaraj Krishnamoorthy, Manoj Kumar, Senthil D Kumaran, Ambily Kuttikkattu, Hmwe Hmwe Kyu, Dennis Odai Laryea, Basira Kankia Lawal, Thao Thi Thu Le, Caterina Ledda, Sang-woong Lee, Seung Lee, Gebretsadik Kiros Lema, Miriam Levi, Stephen S Lim, Xuefeng Liu, Graciliana Lopes, Pedro Henrique Machado Teixeira, Ata Mahmoodpoor, Mansour Adam Mahmoud, Elaheh Malakan Rad, Kashish Malhotra, Ahmad Azam Malik, Bernardo Alfonso Martinez-Guerra, Miquel Martorell, Vasundhara Mathur, Mahsa Mayeli, John Robert Carabeo Medina, Addisu Melese, Ziad A Memish, Alexios-Fotios A Mentis, Muayad Aghali Merza, Tomislav Mestrovic, Irmina Maria Michalek, Le Huu Nhat Minh, Alireza Mirahmadi, Omid Mirmosayyeb, Arup Kumar Misra, Nouh Saad Mohamed, Yousef Mohammad, Esmaeil Mohammadi, Shafiu Mohammed, Maryam Mojarrad Sani, Hoda Mojiri-forushani, Ali H Mokdad, Sara Momtazmanesh, Mohammad Ali Moni, Elias Mossialos, Ebrahim Mostafavi, Majid Motaghinejad, Amin Mousavi Khaneghah, Sumaira Mubarik, Lorenzo Muccioli, Jibran Sualeh Muhammad, Francesk Mulita, Temesgen Mulugeta, Efrén Murillo-Zamora, Christopher J L Murray, Ghulam Mustafa, Saravanan Muthupandian, Ahamarshan Jayaraman Nagarajan, Mohsen Naghavi, Firzan Nainu, Tapas Sadasivan Nair, Shumaila Nargus, Hasan Nassereldine, Zuhair S Natto, Biswa Prakash Nayak, Ionut Negoi, Ruxandra Irina Negoi, Seyed Aria Nejadghaderi, Hien Quang Nguyen, Phat Tuan Nguyen, Van Thanh Nguyen, Robina Khan Niazi, Nafise Noroozi, Amanda Novotney, Khan M Nuruzzaman, Vincent Ebuka Nwatah, Chimezie Igwegbe Nzoputam, Ogochukwu Janet Nzoputam, Bogdan Oancea, Rahman Md Obaidur, Ismail A Odetokun, Ropo Ebenezer Ogunsakin, Osaretin Christabel Okonji, Andrew T Olagunju, Isaac Iyinoluwa Olufadewa, Yinka Doris Oluwafemi, Amel Ouyahia, Mahesh P A, Padmavali Nanaji Palange, Shahina Pardhan, Romil R Parikh, Jay Patel, Urvish K Patel, Shankargouda Patil, Uttam Paudel, Shrikant Pawar, Marcos Pereira, Mario F P Peres, Ionela-Roxana Petcu, Zahra Zahid Piracha, Nayanum Pokhrel, Maarten J Postma, Elton Junio Sady Prates, Ibrahim Qattea, Pankaja Raghav Raghav, Leila Rahbarnia, Vafa Rahimi-

Movaghar, Mosiur Rahman, Muhammad Aziz Rahman, Vahid Rahmanian, Niloufar Rahnavard, Hazem Ramadan, Premkumar Ramasubramani, Usha Rani, Indu Ramachandra Rao, Zubair Ahmed Ratan, Salman Rawaf, Elrashdy Moustafa Mohamed Redwan, Nazila Rezaei, Abanoub Riad, Tércia Moreira Ribeiro da Silva, Jefferson Antonio Buendia Rodriguez, Victor Daniel Rosenthal, Basema Saddik, Saeid Sadeghian, Umar Saeed, Azam Safary, Fatemeh Saheb Sharif-Askari, Narjes Saheb Sharif-Askari, Monalisha Sahu, Seyed Aidin Sajedi, Morteza Saki, Mohamed A Saleh, Malik Sallam, Sara Samadzadeh, Abdallah M Samy, Rama Krishna Sanjeev, Maheswar Satpathy, Abubakar Sha'aban, Mahan Shafie, Pritik A Shah, Shayan Shahrokhi, Masood Ali Shaikh, Mohammed Shannawaz, Fablina Sharara, Aziz Sheikh, Suchitra M Shenoy, Pavanchand H Shetty, Jae Il Shin, Fereshteh Shokri, Seyed Afshin Shorofi, Sunil Shrestha, Migbar Mekonnen Sibhat, Emmanuel Edwar Siddig, Luís Manuel Lopes Rodrigues Silva, Jasvinder A Singh, Paramdeep Singh, Robert Sinto, Anna Aleksandrovna Skryabina, Yonatan Solomon, Prashant Sood, Sergey Soshnikov, Andy Stergachis, Mu'awiyyah Babale Sufiyan, Rizwan Suliankatchi Abdulkader, Abida Sultana, Lucien R Swetschinski, Ensiyeh Taheri, Elahe Taki, Jacques JL Lukenze Tamuzi, Ker-Kan Tan, Mohamad-Hani Temsah, Pugazhenthan Thangaraju, Jansje Henny Vera Ticoalu, Tala Tillawi, Marius Belmondo Tincho, Imad I Tleyjeh, Razie Toghroli, Marcos Roberto Tovani-Palone, Derara Girma Tufa, Paul Turner, Irfan Ullah, Chukwuma David Umeokonkwo, Bhaskaran Unnikrishnan, Seyed Mohammad Vahabi, Asokan Govindaraj Vaithinathan, Rohollah Valizadeh, Shoban Babu Varthya, Avina Vongpradith, Theo Vos, Yasir Waheed, Mandaras Tariku Walde, Cong Wang, Kosala Gayan Weerakoon, Nuwan Darshana Wickramasinghe, Andrea Sylvia Winkler, Melat Woldemariam, Nahom Alemseged Worku, Claire Wright, Han Yong Wunrow, Chalachew Yenew Yenew Yenew, Metin Yesiltepe, Siyan Yi, Vahit Yiğit, Yuyi You, Hadiza Yusuf, Fathiah Zakham, Muhammad Zaman, Sojib Bin Zaman, Armin Zarrintan, Mikhail Sergeevich Zastrozhin, Haijun Zhang, Jingya Zhang, Zhi-Jiang Zhang, Mohammad Zoladl, and Alimuddin Zumla.

Drafting the work or revising it critically for important intellectual content

Amirali Aali, Sherief Abd-Elsalam, Ashkan Abdollahi, Jeza Muhamad Abdul Aziz, Hassan Abidi, Hassan Abolhassani, Eman Abu-Gharbieh, Lawan Hassan Adamu, Isaac Yeboah Addo, Oyelola A Adegboye, Tayo Alex Adekiya, Mohammad Adnan, Qorinah Estiningtyas Sakilah Adnani, Saira Afzal, Shahin Aghamiri, Zahra Babaei Aghdam, Antonella Agodi, Bright Opoku Ahinkorah, Mohadese Ahmadzade, Ali Ahmed, Ayman Ahmed, Jivan Qasim Ahmed, Meqdad Saleh Ahmed, Karolina Akinosoglou, Fares Alahdab, Tareq Mohammed Ali AL-Ahdal, Ahmed Hassan Albelbeisi, Tsegaye Begashaw B Alemayehu, Kefyalew Addis Alene, Abid Ali, Liaqat Ali, Syed Shujait Ali, Sami Almustanyir, Rajaa M Al-Raddadi, Nelson Alvis-Guzman, Yaser Mohammed Al-Worafi, Hany Aly, Robert Ancuceanu, Golnoosh Ansari, Anayochukwu Edward Anyasodor, Jalal Arabloo, Judie Arulappan, Raphael Taiwo Aruleba, Muhammad Asaduzzaman, Kendalem Asmare Atalell, Seyyed Shamsadin Athari, Daniel Atlaw, Maha Moh'd Wahbi Atout, Sameh Attia, Tegegn Mulatu Ayana, Alemu Degu Ayele, Sina Azadnajafabad, Muhammad Badar, Ashish D Badiye, Mahboube Bagheri, Sara Bagherieh, Atif Amin Baig, Aleksandra Barac, Shirin Barati, Mainak Bardhan, Zarrin Basharat, Azadeh Bashiri, Quique Bassat, Saurav Basu, Nebiyou Simegnew Bayileyegn, Neeraj Bedi, Amir Hossein Behnoush, Abebe Ayalew Bekel, Melaku Ashagrie Belete, Olorunjuwon Omolaja Bello, Rose G Bender, Akshaya Srikanth Bhagavathula, Niloufar Bineshfar, Dinesh Bhandari, Sonu Bhaskar, Danilo Buonsenso, Achille Cernigliaro, Vijay Kumar Chattu, Patrick R Ching, Hitesh Chopra, Devasahayam J Christopher, Dinh-Toi Chu, Rosa A S Couto, Natália Cruz-Martins, Saswati Das, Nihar Ranjan Dash, Mohsen Dashti, Demeke Dejen, Hiwot Dejene, Dessalegn Demeke, Berecha Hundessa Demessa, Andreas K Demetriades, Diriba Dereje, Emina Dervišević, Hardik Dineshbhai Desai, Thanh Chi Do, Masoud Dodangeh, Milad Dodangeh, Regina-Mae Villanueva Dominguez, Deepa Dongarwar, Haneil Larson Dsouza, Oyewole Christopher Durojaiye, Arkadiusz Marian Dziedzic, Martin Herbas Ekat, Maysaa El Sayed Zaki, Hassan El-Abid, Muhammed Elhadi, Victor Gabriel El-Hajj, Amro A El-Sakka, Hawi Leul Esayas, Adeniyi Francis Fagbamigbe, Jawad Fares, Ali Fatehizadeh, Syeda Anum Fatima Fatima, Nicholas A Feasey, Getahun Fetensa, Desalegn Feyissa, Florian Fischer, Behzad Foroutan, Peter Andras Gaal, Muktar A Gadanya, Abduzhappar Gaipov, Balasankar Ganesan, Kahsu Gebrekirstos Gebrekidan, Teferi Gebru Gebremeskel, Getachew Muluye Gedef, Yibeltal Yismaw Gela, Bradford D Gessner, Motuma Erena Getachew, Kazem Ghaffari, Seyyed-Hadi Ghamari, Reza Ghanbari, Ramy Mohamed Mohmaed Ghazy, Ghozali Ghozali, Habtamu Alganeh Guadie, Mohak Gupta, Sapna Gupta, Veer Bala Gupta, Vivek Kumar Gupta, Najah R Hadi, Shafiul Haque, Harapan Harapan, Ahmed I Hasaballah, Ikramul Hasan, Hamidreza

Hasani, Mohammad Hasanian, Simon I Hay, Khezar Hayat, Reza Heidari-Soureshjani, Kamal Hezam, Ramesh Holla, Nobuyuki Horita, Md Mahbub Hossain, Mohammad-Salar Hosseini, Sorin Hostiuc, Salman Hussain, Nawfal R Hussein, Segun Emmanuel Ibitoye, Olayinka Stephen Ilesanmi, Irena M Ilic, Milena D Ilic, Kenneth Chukwuemeka Iregbu, Nahlah Elkudssiah Ismail, Chidozie C D Iwu, Chinwe Jaja, Mihajlo Jakovljevic, Amirreza Javadi Mamaghani, Nabi Jomehzadeh, Nitin Joseph, Jacek Jerzy Jozwiak, Himal Kandel, André Karch, Hanie Karimi, Navjot Kaur, Himanshu Khajuria, Amirmohammad Khalaji, Ajmal Khan, Maseer Khan, Taimoor Khan, Khaled Khatab, Moawiah Mohammad Khatatbeh, Jagdish Khubchandani, Min Seo Kim, Adnan Kisa, Sezer Kisa, Farzad Kompani, Nikhil Kothari, Kewal Krishan, Yuvaraj Krishnamoorthy, Mukhtar Kulimbet, Manoj Kumar, Senthil D Kumaran, Alexander Kwarteng, Hmwe Hmwe Kyu, Iván Landires, Dennis Odai Laryea, Basira Kankia Lawal, Thao Thi Thu Le, Gebretsadik Kiros Lema, Miriam Levi, Graciliana Lopes, Ricardo Lutzky Saute, Pedro Henrique Machado Teixeira, Ata Mahmoodpoor, Mansour Adam Mahmoud, Elaheh Malakan Rad, Ahmad Azam Malik, Bernardo Alfonso Martinez-Guerra, Miquel Martorell, Vasundhara Mathur, Mahsa Mayeli, Ziad A Memish, Alexios-Fotios A Mentis, Muayad Aghali Merza, Tomislav Mestrovic, Irmina Maria Michalek, Le Huu Nhat Minh, Alireza Mirahmadi, Omid Mirmosayyeb, Awoke Misganaw, Nouh Saad Mohamed, Yousef Mohammad, Esmaeil Mohammadi, Shafiu Mohammed, Maryam Mojarrad Sani, Hoda Mojiri-forushani, Ali H Mokdad, Sara Momtazmanesh, Lorenzo Monasta, Mohammad Ali Moni, Ebrahim Mostafavi, Majid Motaghinejad, Amin Mousavi Khaneghah, Jibran Sualeh Muhammad, Temesgen Mulugeta, Efrén Murillo-Zamora, Christopher J L Murray, Ghulam Mustafa, Saravanan Muthupandian, Ahamarshan Jayaraman Nagarajan, Mohsen Naghavi, Firzan Nainu, Shumaila Nargus, Hasan Nassereldine, Zuhair S Natto, Ionut Negoi, Ruxandra Irina Negoi, Seyed Aria Nejadghaderi, Hien Quang Nguyen, Phat Tuan Nguyen, Van Thanh Nguyen, Robina Khan Niazi, Amanda Novotney, Virginia Nuñez-Samudio, Khan M Nuruzzaman, Vincent Ebuka Nwatah, Chimezie Igwegbe Nzoputam, Ogochukwu Janet Nzoputam, Bogdan Oancea, Ismail A Odetokun, Osaretin Christabel Okonji, Andrew T Olagunju, Latera Tesfaye Olana, Kemal Sherefa Oumer, Amel Ouyahia, Mahesh P A, Shahina Pardhan, Jay Patel, Urvish K Patel, Shankargouda Patil, Uttam Paudel, Shrikant Pawar, Umberto Pensato, João Perdigão, Marcos Pereira, Mario F P Peres, Ionela-Roxana Petcu, Marina Pinheiro, Zahra Zahid Piracha, Maarten J Postma, Elton Junio Sady Prates, Pankaja Raghav Raghav, Vafa Rahimi-Movaghar, Niloufar Rahnavard, Hazem Ramadan, Premkumar Ramasubramani, Deepthi Rapaka, Zubair Ahmed Ratan, Salman Rawaf, Elrashdy Moustafa Mohamed Redwan, Nazila Rezaei, Abanoub Riad, Tércia Moreira Ribeiro da Silva, Jefferson Antonio Buendia Rodriguez, Victor Daniel Rosenthal, Basema Saddik, Umar Saeed, Fatemeh Saheb Sharif-Askari, Narjes Saheb Sharif-Askari, Amirhossein Sahebkar, Monalisha Sahu, Seyed Aidin Sajedi, Morteza Saki, Saina Salahi, Sarvenaz Salahi, Sara Samadzadeh, Abdallah M Samy, Maheswar Satpathy, Allen Seylani, Abubakar Sha'aban, Mahan Shafie, Kiana Shahzamani, Mohammed Shannawaz, Suchitra M Shenoy, Pavanchand H Shetty, Fereshteh Shokri, Seyed Afshin Shorofi, Sunil Shrestha, Emmanuel Edwar Siddig, Luís Manuel Lopes Rodrigues Silva, Harpreet Singh, Jasvinder A Singh, Paramdeep Singh, Surjit Singh, Robert Sinto, Sarah Brooke Sirota, Anna Aleksandrovna Skryabina, Bogdan Socea, Anton Sokhan, Ranjan Solanki, Yonatan Solomon, Prashant Sood, Sergey Soshnikov, Mu'awiyyah Babale Sufiyan, Lucien R Swetschinski, Sree Sudha T Y, Jacques JL Lukenze Tamuzi, Ker-Kan Tan, Nathan Y Tat, Mohamad-Hani Temsah, Dufera Rikitu Terefa, Pugazhenthan Thangaraju, Nigusie Selomon Tibebu, Jansje Henny Vera Ticoalu, Tala Tillawi, Marius Belmondo Tincho, Imad I Tleyjeh, Marcos Roberto Tovani-Palone, Paul Turner, Irfan Ullah, Chukwuma David Umeokonkwo, Bhaskaran Unnikrishnan, Asokan Govindaraj Vaithinathan, Shoban Babu Varthya, Avina Vongpradith, Yasir Waheed, Kosala Gayan Weerakoon, Nuwan Darshana Wickramasinghe, Andrea Sylvia Winkler, Claire Wright, Han Yong Wunrow, Dereje Y Yada, Sajad Yaghoubi, Gahin Abdulraheem Tayib Yahya Yahya, Metin Yesiltepe, Vahit Yiğit, Yuyi You, Hadiza Yusuf, Fathiah Zakham, Muhammad Zaman, Sojib Bin Zaman, Iman Zare, Armin Zarrintan, Mikhail Sergeevich Zastrozhin, Mohammad Zoladl, and Alimuddin Zumla.

Managing the estimation or publications process

Shirin Barati, Rosa A S Couto, Demeke Dejen, Hiwot Dejene, Thanh Chi Do, Maysaa El Sayed Zaki, Hawi Leul Esayas, Ali Fatehizadeh, Desalegn Feyissa, Getachew Muluye Gedef, Kazem Ghaffari, Simon I Hay, Amirreza Javadi Mamaghani, Manoj Kumar, Hmwe Hmwe Kyu, Thao Thi Thu Le, Le Huu Nhat Minh, Ali H Mokdad, Christopher J L Murray, Mohsen Naghavi, Hien Quang Nguyen, Phat Tuan Nguyen, Van Thanh

Nguyen, Amanda Novotney, Mahesh P A, Abdallah M Samy, Maheswar Satpathy, Lucien R Swetschinski, Pugazhenthan Thangaraju, Shoban Babu Varthya, and Mikhail Sergeevich Zastrozhin.
