# Supplementary material for: Global, regional, and national burden of meningitis and its aetiologies, 1990–2019: a systematic analysis for the Global Burden of Disease Study 2019
Source: Lancet Neurol. 2023 Aug;22(8):685–711. doi: 10.1016/S1474-4422(23)00195-3 (PMC10356620; doi:10.1016/S1474-4422(23)00195-3)
Supplement: Supplementary appendix 2 [file mmc2.pdf]

THE LANCET
Neurology

Supplementary appendix 2

This appendix formed part of the original submission and has been peer reviewed. We post it as supplied by the authors.

Supplement to: GBD 2019 Meningitis and Antimicrobial Resistance Collaborators. Global, regional, and national burden of meningitis and its aetiologies, 1990–2019: a systematic analysis for the Global Burden of Disease Study 2019. *Lancet Neurol* 2023; **22:** 685–711.

Contents

Appendix Table S1: Incidence and mortality counts and rates per 100,000 population by super-region, region, and country for children under 5 years old and all ages 2

Appendix Table S2: Fatal and non-fatal meningitis aetiology proportions in 2019 for neonates, children under 5 years old, and all ages by super-region, region, country, and globally 16

Appendix Table S3: Global meningitis cases and deaths in 1990 and 2019 and percent change between 1990 and 2019 by aetiology, children under 5 years old and all ages........ 94

Appendix Table S4: Meningitis belt meningitis cases and deaths in 1990 and 2019 and percent change between 1990 and 2019 by aetiology, children under 5 years old and all ages 96

Appendix Table S5: Proportions of global meningitis cases and deaths occurring in the meningitis belt, 1990 and 2019, children under 5 and all ages 98

Appendix Table S6: Aetiology proportions (%) and mortality rates (per 100,000 population) in the meningitis belt, 1990 and 2019, children under 5 and all ages 99

Appendix Table S1: Incidence and mortality counts and rates per 100,000 population by super-region, region, and country for children under 5 years old and all ages

	All Ages				Under 5			
**Location**	**Number of Deaths (95% CI)**	**Age-Standardized Mortality Rate (95% CI)**	**Number of Cases (95% CI)**	**Age-Standardized Incidence Rate (95% CI)**	**Number of Deaths (95% CI)**	**Age-Standardized Mortality Rate (95% CI)**	**Number of Cases (95% CI)**	**Age-Standardized Incidence Rate (95% CI)**
**Global**	**236,000 (204,000 to 277,000)**	**3.3 (2.8 to 3.9)**	**2,510,000 (2,110,000 to 2,990,000)**	**35.4 (29.6 to 42.5)**	**112,000 (87,400 to 145,000)**	**16.9 (13.2 to 21.9)**	**1,280,000 (947,000 to 1,710,000)**	**192.4 (142.8 to 258.6)**
**Central Europe, Eastern Europe, and Central Asia**	**2,700 (2,440 to 2,960)**	**0.6 (0.6 to 0.7)**	**70,800 (57,000 to 86,000)**	**20.9 (16.6 to 25.6)**	**450 (368 to 565)**	**1.6 (1.3 to 2.0)**	**21,200 (15,100 to 30,200)**	**77.1 (54.8 to 109.5)**
Central Asia	638 (552 to 752)	0.7 (0.6 to 0.8)	27,500 (22,000 to 33,800)	28.9 (23.1 to 35.6)	186 (142 to 269)	1.9 (1.5 to 2.8)	10,100 (7,290 to 14,100)	105.6 (76.1 to 147.5)
Armenia	5(4 to 6)	0.2 (0.1 to 0.2)	708(557 to 874)	28.1 (21.6 to 34.8)	0(0 to 1)	0.2 (0.2 to 0.3)	176(114 to 271)	86.2 (56.0 to 132.7)
Azerbaijan	62(49 to 77)	0.7 (0.6 to 0.9)	2,710(2,180 to 3,340)	29.8 (24.0 to 36.6)	16(10 to 25)	2.1 (1.3 to 3.3)	838(599 to 1,200)	110.3 (78.9 to 157.6)
Georgia	21(17 to 24)	0.5 (0.4 to 0.6)	782(638 to 960)	26.6 (21.1 to 32.8)	3(2 to 4)	1.2 (0.8 to 1.7)	214(155 to 295)	86.9 (62.8 to 119.5)
Kazakhstan	136(114 to 158)	0.7 (0.6 to 0.9)	4,840(3,850 to 6,010)	26.7 (21.1 to 33.1)	39(27 to 56)	2.1 (1.5 to 3.0)	1,710(1,200 to 2,360)	92.6 (64.9 to 128.3)
Kyrgyzstan	38(33 to 43)	0.6 (0.5 to 0.7)	2,120(1,670 to 2,680)	29.9 (23.8 to 37.5)	12(9 to 16)	1.6 (1.2 to 2.1)	827(598 to 1,190)	110.0 (79.5 to 157.8)
Mongolia	24(18 to 30)	0.7 (0.6 to 1.0)	1,090(873 to 1,360)	30.6 (24.5 to 38.0)	5(3 to 8)	1.3 (0.8 to 2.0)	356(256 to 527)	90.3 (64.9 to 133.6)
Tajikistan	125(94 to 185)	1.3 (1.0 to 1.8)	3,870(3,080 to 4,750)	35.5 (28.5 to 43.0)	57(34 to 118)	4.7 (2.8 to 9.8)	1,770(1,280 to 2,460)	146.6 (106.3 to 204.4)
Turkmenistan	52(43 to 64)	1.0 (0.8 to 1.2)	1,790(1,450 to 2,170)	33.7 (27.5 to 40.9)	20(14 to 27)	3.6 (2.5 to 4.9)	718(537 to 990)	130.4 (97.6 to 179.8)
Uzbekistan	175(144 to 212)	0.5 (0.4 to 0.6)	9,580(7,570 to 12,000)	26.9 (21.3 to 33.5)	34(25 to 46)	0.9 (0.7 to 1.3)	3,510(2,470 to 5,110)	97.0 (68.4 to 141.4)
Central Europe	421 (357 to 484)	0.3 (0.3 to 0.3)	10,300 (8,220 to 12,300)	12.2 (9.6 to 15.1)	42 (32 to 52)	0.7 (0.6 to 0.9)	2,540 (1,780 to 3,660)	44.8 (31.4 to 64.8)
Albania	13(10 to 16)	0.5 (0.4 to 0.6)	254(206 to 307)	11.6 (9.3 to 14.2)	3(2 to 5)	1.8 (1.0 to 3.0)	77(55 to 107)	47.0 (33.7 to 65.4)
Bosnia and Herzegovina	9(7 to 11)	0.3 (0.2 to 0.3)	379(303 to 464)	16.4 (12.9 to 20.2)	1(1 to 2)	1.0 (0.7 to 1.3)	96(67 to 140)	65.7 (45.9 to 95.9)

	All Ages				Under 5			
Location	Number of Deaths (95% CI)	Age-Standardized Mortality Rate (95% CI)	Number of Cases (95% CI)	Age-Standardized Incidence Rate (95% CI)	Number of Deaths (95% CI)	Age-Standardized Mortality Rate (95% CI)	Number of Cases (95% CI)	Age-Standardized Incidence Rate (95% CI)
Bulgaria	39(31 to 49)	0.5 (0.4 to 0.6)	584(474 to 702)	11.8 (9.4 to 14.6)	5(4 to 7)	1.7 (1.2 to 2.3)	143(103 to 203)	45.6 (32.7 to 64.8)
Croatia	17(13 to 21)	0.3 (0.2 to 0.3)	398(335 to 464)	12.4 (10.3 to 15.1)	1(1 to 2)	0.6 (0.4 to 0.8)	82(60 to 111)	44.4 (32.6 to 59.6)
Czechia	38(31 to 48)	0.3 (0.2 to 0.3)	1,030(823 to 1,250)	12.9 (10.0 to 15.9)	3(2 to 5)	0.6 (0.4 to 0.8)	271(189 to 384)	47.7 (33.2 to 67.6)
Hungary	50(41 to 62)	0.4 (0.3 to 0.5)	762(610 to 915)	10.6 (8.1 to 13.2)	4(2 to 5)	0.8 (0.5 to 1.2)	159(109 to 232)	36.2 (24.8 to 52.8)
Montenegro	1(1 to 2)	0.2 (0.1 to 0.2)	65(51 to 79)	13.2 (10.2 to 16.4)	0(0 to 0)	0.4 (0.2 to 0.5)	15(10 to 23)	44.0 (30.1 to 66.0)
North Macedonia	5(4 to 7)	0.3 (0.2 to 0.3)	231(183 to 284)	13.9 (11.0 to 17.3)	1(1 to 2)	1.3 (0.9 to 1.7)	58(41 to 80)	50.6 (35.8 to 70.6)
Poland	135(102 to 167)	0.3 (0.2 to 0.3)	3,620(2,860 to 4,400)	12.9 (9.9 to 16.2)	8(5 to 11)	0.4 (0.3 to 0.6)	862(572 to 1,290)	45.2 (29.9 to 67.8)
Romania	60(48 to 73)	0.3 (0.2 to 0.4)	1,360(1,100 to 1,640)	10.0 (7.9 to 12.5)	9(6 to 12)	0.9 (0.6 to 1.2)	406(292 to 559)	43.2 (31.1 to 59.5)
Serbia	27(21 to 33)	0.2 (0.2 to 0.3)	699(558 to 840)	10.5 (8.2 to 12.9)	3(2 to 4)	0.6 (0.4 to 0.8)	173(119 to 245)	38.3 (26.3 to 54.2)
Slovakia	20(16 to 26)	0.3 (0.3 to 0.5)	658(526 to 801)	15.7 (12.2 to 19.3)	3(2 to 5)	1.2 (0.8 to 1.7)	143(99 to 208)	50.0 (34.6 to 72.8)
Slovenia	6(4 to 7)	0.2 (0.1 to 0.2)	219(173 to 263)	14.3 (11.1 to 17.6)	0(0 to 0)	0.2 (0.1 to 0.3)	49(35 to 71)	49.1 (34.6 to 70.6)
Eastern Europe	1,640 (1,480 to 1,800)	0.7 (0.6 to 0.8)	33,000 (26,700 to 39,800)	19.9 (15.7 to 24.4)	223 (183 to 267)	1.8 (1.5 to 2.2)	8,600 (5,920 to 12,500)	69.7 (48.0 to 101.2)
Belarus	49(37 to 63)	0.5 (0.4 to 0.6)	1,760(1,440 to 2,160)	23.3 (18.7 to 29.0)	10(7 to 14)	1.8 (1.2 to 2.5)	442(314 to 644)	78.4 (55.7 to 114.3)
Estonia	6(5 to 7)	0.3 (0.3 to 0.4)	188(150 to 227)	18.3 (14.2 to 22.7)	0(0 to 1)	0.7 (0.5 to 1.0)	41(28 to 62)	58.2 (39.8 to 88.8)
Latvia	8(7 to 10)	0.3 (0.3 to 0.4)	232(187 to 280)	16.2 (12.8 to 19.9)	1(0 to 1)	0.7 (0.5 to 1.0)	64(44 to 93)	61.3 (42.7 to 89.4)
Lithuania	16(13 to 19)	0.4 (0.4 to 0.5)	349(286 to 421)	16.5 (13.1 to 20.4)	1(1 to 2)	0.9 (0.6 to 1.3)	83(57 to 117)	57.7 (39.8 to 81.8)
Republic of Moldova	20(17 to 23)	0.5 (0.5 to 0.6)	550(449 to 665)	20.4 (16.3 to 25.1)	3(2 to 4)	1.8 (1.3 to 2.4)	145(105 to 202)	83.4 (60.4 to 116.7)
Russian Federation	1,140 (996 to 1,300)	0.7 (0.6 to 0.8)	23,000 (18,500 to 27,800)	19.4 (15.3 to 23.8)	140 (110 to 173)	1.5 (1.2 to 1.9)	6,250 (4,290 to 9,110)	68.4 (46.9 to 99.7)

Location	All Ages				Under 5			
	Number of Deaths (95% CI)	Age-Standardized Mortality Rate (95% CI)	Number of Cases (95% CI)	Age-Standardized Incidence Rate (95% CI)	Number of Deaths (95% CI)	Age-Standardized Mortality Rate (95% CI)	Number of Cases (95% CI)	Age-Standardized Incidence Rate (95% CI)
Ukraine	399(338 to 469)	0.9 (0.8 to 1.1)	6,940(5,620 to 8,350)	21.1 (16.6 to 25.8)	67(50 to 87)	3.1 (2.3 to 4.1)	1,570(1,090 to 2,190)	73.4 (51.0 to 102.3)
**High-income**	**3,820 (3,570 to 4,010)**	**0.3 (0.3 to 0.3)**	**96,000 (78,200 to 114,000)**	**10.8 (8.7 to 13.1)**	**380 (327 to 435)**	**0.7 (0.6 to 0.8)**	**19,000 (13,100 to 27,100)**	**33.4 (23.0 to 47.7)**
Australasia	59(54 to 64)	0.2 (0.2 to 0.2)	3,490(2,790 to 4,280)	15.3 (11.9 to 19.2)	12(9 to 15)	0.7 (0.5 to 0.8)	1,040(706 to 1,530)	57.1 (38.8 to 84.0)
Australia	46(42 to 50)	0.2 (0.2 to 0.2)	2,950(2,340 to 3,630)	15.4 (11.9 to 19.5)	9(7 to 12)	0.6 (0.4 to 0.8)	883(598 to 1,310)	57.9 (39.2 to 86.0)
New Zealand	13(12 to 14)	0.3 (0.2 to 0.3)	544(444 to 667)	14.7 (11.8 to 18.7)	3(2 to 4)	1.0 (0.8 to 1.3)	157(108 to 226)	53.4 (36.8 to 76.9)
High-income Asia Pacific	491 (427 to 531)	0.1 (0.1 to 0.1)	27,700 (22,200 to 33,600)	21.2 (16.7 to 26.5)	17 (14 to 19)	0.2 (0.2 to 0.3)	3,320 (2,230 to 4,820)	45.5 (30.6 to 66.1)
Brunei Darussalam	2(1 to 2)	0.5 (0.5 to 0.6)	103(80 to 131)	25.5 (19.7 to 32.0)	0(0 to 0)	1.0 (0.7 to 1.3)	22(15 to 32)	69.6 (48.8 to 102.8)
Japan	407 (351 to 443)	0.1 (0.1 to 0.2)	16,200 (13,400 to 19,400)	18.6 (14.8 to 23.0)	12 (10 to 13)	0.3 (0.2 to 0.3)	1,930 (1,320 to 2,780)	40.3 (27.6 to 58.1)
Republic of Korea	77 (67 to 88)	0.1 (0.1 to 0.1)	10,400 (8,130 to 13,200)	26.7 (20.6 to 34.0)	4 (3 to 5)	0.2 (0.1 to 0.2)	1,220 (797 to 1,790)	56.3 (36.6 to 82.2)
Singapore	5(4 to 5)	0.1 (0.1 to 0.1)	913(700 to 1,150)	21.0 (16.0 to 26.8)	0(0 to 1)	0.2 (0.1 to 0.2)	142(91 to 214)	48.9 (31.3 to 73.9)
High-income North America	1,240 (1,180 to 1,280)	0.3 (0.3 to 0.3)	16,300 (13,600 to 19,300)	4.4 (3.7 to 5.2)	137 (122 to 152)	0.7 (0.6 to 0.7)	2,200 (1,490 to 3,200)	10.5 (7.1 to 15.2)
Canada	89(81 to 98)	0.2 (0.2 to 0.2)	1,880(1,510 to 2,290)	5.5 (4.4 to 6.6)	13(10 to 17)	0.7 (0.5 to 0.9)	314(217 to 462)	16.3 (11.3 to 24.0)
Greenland	1(1 to 1)	1.7 (1.3 to 2.3)	6(5 to 7)	9.8 (8.4 to 11.3)	0(0 to 0)	4.4 (2.6 to 7.1)	1(1 to 1)	28.4 (21.2 to 37.3)
United States of America	1,150(1,090 to 1,190)	0.3 (0.3 to 0.3)	14,500(12,000 to 17,100)	4.3 (3.6 to 5.1)	124(111 to 138)	0.6 (0.6 to 0.7)	1,880(1,270 to 2,740)	9.9 (6.7 to 14.4)
Southern Latin America	580(524 to 631)	0.8 (0.7 to 0.9)	6,780(5,550 to 8,200)	11.4 (9.2 to 13.9)	81(61 to 105)	1.7 (1.2 to 2.2)	2,180(1,550 to 3,100)	44.8 (32.0 to 63.8)
Argentina	453(407 to 496)	1.0 (0.9 to 1.1)	4,730(3,860 to 5,700)	11.5 (9.3 to 14.0)	67(49 to 89)	1.9 (1.4 to 2.6)	1,520(1,100 to 2,120)	43.9 (31.6 to 61.1)

	All Ages				Under 5			
Location	Number of Deaths (95% CI)	Age-Standardized Mortality Rate (95% CI)	Number of Cases (95% CI)	Age-Standardized Incidence Rate (95% CI)	Number of Deaths (95% CI)	Age-Standardized Mortality Rate (95% CI)	Number of Cases (95% CI)	Age-Standardized Incidence Rate (95% CI)
Chile	113(100 to 127)	0.6 (0.5 to 0.6)	1,770(1,420 to 2,150)	11.7 (9.2 to 14.5)	13(9 to 18)	1.1 (0.8 to 1.5)	578(408 to 869)	49.9 (35.2 to 75.0)
Uruguay	14(13 to 16)	0.3 (0.3 to 0.3)	286(229 to 347)	9.5 (7.4 to 11.7)	1(1 to 1)	0.5 (0.3 to 0.6)	78(52 to 115)	33.8 (22.5 to 49.9)
Western Europe	1,450 (1,350 to 1,550)	0.2 (0.2 to 0.3)	41,700 (34,000 to 49,600)	12.5 (10.0 to 15.2)	134 (111 to 157)	0.6 (0.5 to 0.7)	10,300 (7,150 to 14,800)	46.7 (32.5 to 67.1)
Andorra	0(0 to 0)	0.1 (0.1 to 0.1)	7(5 to 8)	11.6 (8.9 to 14.4)	0(0 to 0)	0.2 (0.1 to 0.3)	1(1 to 2)	38.9 (25.5 to 59.4)
Austria	31(28 to 34)	0.2 (0.2 to 0.3)	768(643 to 901)	10.9 (8.8 to 13.3)	3(2 to 4)	0.6 (0.5 to 0.8)	180(129 to 252)	40.9 (29.4 to 57.3)
Belgium	45(41 to 50)	0.3 (0.3 to 0.3)	1,140(917 to 1,360)	12.7 (9.9 to 15.6)	6(4 to 8)	0.9 (0.7 to 1.2)	293(197 to 421)	47.4 (31.9 to 68.1)
Cyprus	3(2 to 3)	0.2 (0.1 to 0.2)	66(53 to 80)	6.6 (5.2 to 8.4)	0(0 to 0)	0.4 (0.3 to 0.6)	23(16 to 35)	31.2 (20.9 to 46.9)
Denmark	31(28 to 34)	0.3 (0.3 to 0.4)	941(769 to 1,140)	20.5 (16.3 to 25.3)	2(1 to 2)	0.6 (0.4 to 0.8)	217(151 to 310)	70.4 (49.0 to 100.8)
Finland	16(14 to 18)	0.2 (0.2 to 0.2)	452(361 to 546)	10.7 (8.4 to 13.3)	1(0 to 1)	0.2 (0.2 to 0.3)	100(66 to 146)	38.1 (25.3 to 55.9)
France	226(200 to 247)	0.2 (0.2 to 0.3)	7,000(5,700 to 8,380)	13.6 (10.8 to 16.7)	23(18 to 29)	0.6 (0.5 to 0.8)	2,060(1,450 to 2,970)	56.5 (39.7 to 81.4)
Germany	216(190 to 238)	0.2 (0.2 to 0.2)	5,650(4,540 to 6,810)	9.2 (7.2 to 11.5)	15(12 to 19)	0.4 (0.3 to 0.5)	1,420(956 to 2,110)	36.1 (24.4 to 53.9)
Greece	30(26 to 33)	0.2 (0.2 to 0.3)	762(617 to 916)	10.8 (8.5 to 13.5)	3(2 to 4)	0.7 (0.5 to 0.9)	217(152 to 319)	48.1 (33.5 to 70.6)
Iceland	1(1 to 1)	0.3 (0.2 to 0.4)	79(64 to 96)	27.4 (21.8 to 33.4)	0(0 to 0)	0.6 (0.4 to 1.0)	20(14 to 29)	93.7 (65.0 to 136.8)
Ireland	10(9 to 11)	0.2 (0.2 to 0.2)	539(427 to 656)	13.4 (10.5 to 16.6)	2(1 to 2)	0.6 (0.4 to 0.7)	172(118 to 259)	54.0 (37.2 to 81.7)
Israel	28(25 to 31)	0.3 (0.2 to 0.3)	989(786 to 1,220)	10.6 (8.4 to 13.2)	5(4 to 7)	0.5 (0.4 to 0.7)	375(260 to 553)	39.6 (27.5 to 58.4)
Italy	185(168 to 197)	0.2 (0.2 to 0.2)	2,960(2,440 to 3,520)	5.5 (4.5 to 6.6)	7(6 to 9)	0.3 (0.3 to 0.4)	321(216 to 466)	13.6 (9.1 to 19.7)
Luxembourg	1(1 to 1)	0.2 (0.1 to 0.2)	55(43 to 66)	11.5 (9.0 to 14.1)	0(0 to 0)	0.4 (0.2 to 0.6)	14(10 to 20)	43.8 (31.0 to 62.8)
Malta	2(1 to 2)	0.3 (0.2 to 0.3)	53(43 to 63)	16.3 (12.9 to 20.0)	0(0 to 0)	1.0 (0.6 to 1.4)	14(10 to 20)	62.4 (44.8 to 90.3)
Monaco	0(0 to 0)	0.2 (0.2 to 0.3)	3(3 to 4)	12.1 (9.4 to 14.9)	0(0 to 0)	0.5 (0.3 to 0.7)	1(0 to 1)	42.9 (29.2 to 64.0)

	All Ages				Under 5			
Location	Number of Deaths (95% CI)	Age-Standardized Mortality Rate (95% CI)	Number of Cases (95% CI)	Age-Standardized Incidence Rate (95% CI)	Number of Deaths (95% CI)	Age-Standardized Mortality Rate (95% CI)	Number of Cases (95% CI)	Age-Standardized Incidence Rate (95% CI)
Netherlands	96(87 to 105)	0.4 (0.4 to 0.5)	1,900(1,550 to 2,240)	14.6 (11.6 to 17.7)	10(7 to 12)	1.1 (0.8 to 1.4)	547(378 to 799)	62.1 (43.0 to 90.8)
Norway	19(17 to 20)	0.3 (0.2 to 0.3)	1,040(825 to 1,280)	24.1 (18.8 to 30.3)	1(1 to 2)	0.5 (0.4 to 0.6)	198(134 to 300)	67.3 (45.6 to 102.0)
Portugal	36(30 to 41)	0.2 (0.2 to 0.3)	1,680(1,340 to 2,060)	21.5 (16.9 to 26.5)	2(1 to 3)	0.4 (0.3 to 0.6)	262(181 to 366)	63.0 (43.7 to 88.1)
San Marino	0(0 to 0)	0.2 (0.2 to 0.4)	4(3 to 5)	14.7 (11.7 to 18.1)	0(0 to 0)	0.7 (0.4 to 1.0)	1(1 to 1)	53.2 (36.2 to 77.3)
Spain	155(130 to 175)	0.2 (0.2 to 0.3)	4,350(3,500 to 5,270)	13.0 (10.2 to 15.8)	11(8 to 15)	0.5 (0.4 to 0.7)	969(676 to 1,400)	48.1 (33.6 to 69.4)
Sweden	31(28 to 34)	0.2 (0.2 to 0.2)	811(610 to 1,030)	10.0 (7.2 to 13.3)	2(2 to 3)	0.3 (0.3 to 0.4)	196(118 to 337)	32.8 (19.7 to 56.6)
Switzerland	27(23 to 30)	0.2 (0.2 to 0.2)	626(505 to 752)	9.5 (7.4 to 11.9)	1(1 to 1)	0.2 (0.2 to 0.3)	182(118 to 272)	40.8 (26.5 to 60.8)
United Kingdom	265(250 to 278)	0.3 (0.3 to 0.4)	9,830(7,990 to 11,700)	18.1 (14.5 to 22.0)	39(32 to 47)	1.0 (0.8 to 1.2)	2,480(1,710 to 3,520)	63.5 (43.9 to 90.4)
**Latin America and Caribbean**	**5,560 (4,720 to 6,510)**	**1.0 (0.9 to 1.2)**	**93,000 (76,000 to 111,000)**	**16.8 (13.7 to 20.0)**	**2,040 (1,520 to 2,630)**	**4.2 (3.2 to 5.5)**	**25,000 (18,000 to 35,100)**	**52.1 (37.4 to 73.1)**
Andean Latin America	395(314 to 494)	0.6 (0.5 to 0.8)	4,400(3,590 to 5,270)	6.9 (5.7 to 8.3)	112(80 to 161)	1.8 (1.3 to 2.5)	2,120(1,540 to 2,900)	33.5 (24.4 to 45.8)
Bolivia (Plurinational State of)	134(100 to 175)	1.2 (0.9 to 1.5)	1,030(856 to 1,240)	7.7 (6.4 to 9.1)	53(34 to 80)	3.5 (2.3 to 5.3)	589(439 to 771)	38.9 (29.1 to 51.0)
Ecuador	118(93 to 148)	0.7 (0.6 to 0.9)	1,190(978 to 1,440)	6.9 (5.7 to 8.3)	30(19 to 45)	1.7 (1.1 to 2.6)	581(427 to 777)	34.0 (25.0 to 45.4)
Peru	143(102 to 196)	0.4 (0.3 to 0.6)	2,180(1,760 to 2,640)	6.7 (5.4 to 8.1)	29(17 to 49)	0.9 (0.5 to 1.6)	949(655 to 1,370)	30.5 (21.0 to 44.0)
Caribbean	1,420 (1,080 to 1,860)	3.4 (2.5 to 4.5)	10,000 (8,390 to 11,700)	23.4 (19.4 to 27.6)	947 (633 to 1,350)	24.0 (16.0 to 34.2)	5,070 (3,760 to 6,720)	128.4 (95.0 to 170.3)
Antigua and Barbuda	1(1 to 1)	1.0 (0.8 to 1.2)	13(10 to 15)	17.2 (14.0 to 20.7)	0(0 to 0)	3.0 (1.9 to 4.6)	3(2 to 5)	66.1 (47.7 to 94.0)
Bahamas	3(2 to 3)	0.8 (0.6 to 1.0)	45(37 to 55)	14.6 (11.9 to 17.7)	0(0 to 1)	1.7 (1.1 to 2.5)	13(9 to 18)	57.6 (41.6 to 82.2)
Barbados	3(2 to 4)	0.9 (0.7 to 1.1)	38(31 to 45)	16.6 (13.5 to 20.2)	0(0 to 0)	2.0 (1.3 to 3.0)	11(8 to 15)	72.2 (51.7 to 100.5)
Belize	2(2 to 3)	0.7 (0.5 to 0.8)	69(56 to 85)	17.2 (13.9 to 20.8)	1(1 to 1)	2.1 (1.5 to 2.9)	26(19 to 36)	69.1 (50.2 to 95.1)

Location	All Ages				Under 5			
	Number of Deaths (95% CI)	Age-Standardized Mortality Rate (95% CI)	Number of Cases (95% CI)	Age-Standardized Incidence Rate (95% CI)	Number of Deaths (95% CI)	Age-Standardized Mortality Rate (95% CI)	Number of Cases (95% CI)	Age-Standardized Incidence Rate (95% CI)
Bermuda	0(0 to 0)	0.3 (0.2 to 0.4)	11(9 to 13)	22.5 (17.8 to 27.6)	0(0 to 0)	0.8 (0.6 to 1.2)	2(1 to 3)	77.8 (53.5 to 116.7)
Cuba	94(76 to 115)	0.7 (0.6 to 0.9)	1,460(1,200 to 1,740)	16.2 (13.2 to 19.6)	11(8 to 14)	1.9 (1.4 to 2.6)	335(242 to 467)	59.6 (43.1 to 83.1)
Dominica	1(1 to 1)	1.3 (1.0 to 1.8)	11(9 to 13)	19.3 (15.8 to 23.3)	0(0 to 0)	5.5 (3.5 to 8.3)	4(3 to 5)	89.0 (63.7 to 122.4)
Dominican Republic	217(152 to 301)	2.1 (1.5 to 2.9)	2,270(1,890 to 2,670)	20.9 (17.4 to 24.5)	91(55 to 144)	8.3 (5.0 to 13.2)	1,100(799 to 1,450)	100.3 (73.0 to 132.4)
Grenada	0(0 to 1)	0.5 (0.4 to 0.6)	14(12 to 17)	15.9 (12.9 to 19.3)	0(0 to 0)	1.2 (0.8 to 1.9)	4(3 to 6)	63.0 (44.7 to 90.7)
Guyana	6(4 to 8)	0.9 (0.6 to 1.1)	102(83 to 125)	13.7 (11.1 to 16.7)	1(1 to 2)	2.0 (1.3 to 3.0)	38(27 to 55)	54.2 (38.7 to 76.9)
Haiti	997(683 to 1,370)	7.1 (5.0 to 9.6)	4,490(3,660 to 5,410)	31.8 (26.3 to 37.8)	801(522 to 1,180)	52.6 (34.3 to 77.1)	3,030(2,240 to 3,950)	198.7 (146.9 to 259.4)
Jamaica	21(16 to 27)	0.8 (0.6 to 1.0)	471(385 to 565)	20.0 (16.3 to 24.2)	3(2 to 5)	1.7 (1.1 to 2.4)	168(119 to 229)	91.0 (64.2 to 123.8)
Puerto Rico	9(7 to 11)	0.2 (0.2 to 0.3)	376(301 to 453)	14.7 (11.5 to 18.1)	1(1 to 1)	0.6 (0.4 to 0.9)	72(50 to 106)	55.0 (37.8 to 80.2)
Saint Kitts and Nevis	1(0 to 1)	1.0 (0.7 to 1.2)	9(7 to 10)	18.0 (14.7 to 21.7)	0(0 to 0)	2.5 (1.5 to 3.6)	3(2 to 4)	77.3 (55.7 to 104.4)
Saint Lucia	2(1 to 2)	0.9 (0.7 to 1.2)	25(21 to 30)	18.3 (14.9 to 21.9)	0(0 to 0)	2.4 (1.5 to 3.5)	7(5 to 9)	74.5 (54.9 to 102.4)
Saint Vincent and the Grenadines	1(1 to 2)	1.2 (0.9 to 1.4)	18(15 to 21)	18.5 (15.2 to 22.1)	0(0 to 0)	3.3 (2.1 to 4.9)	6(5 to 8)	79.0 (58.3 to 108.3)
Suriname	7(5 to 9)	1.3 (1.0 to 1.7)	101(83 to 121)	19.5 (16.0 to 23.5)	2(2 to 4)	5.4 (3.4 to 8.1)	42(31 to 56)	91.1 (66.8 to 123.3)
Trinidad and Tobago	11(8 to 15)	0.8 (0.5 to 1.0)	160(130 to 192)	13.6 (10.9 to 16.5)	1(1 to 1)	1.1 (0.7 to 1.6)	38(26 to 56)	46.3 (31.9 to 67.3)
United States Virgin Islands	0(0 to 0)	0.3 (0.2 to 0.4)	14(11 to 17)	16.4 (12.9 to 20.4)	0(0 to 0)	0.4 (0.2 to 0.6)	4(3 to 6)	61.9 (41.2 to 91.9)
Central Latin America	1,670 (1,360 to 2,030)	0.7 (0.6 to 0.9)	19,900 (16,000 to 24,200)	8.4 (6.7 to 10.2)	365 (261 to 490)	1.7 (1.2 to 2.3)	6,930 (4,870 to 9,940)	32.0 (22.5 to 45.9)
Colombia	377(280 to 489)	0.8 (0.6 to 1.0)	3,740(3,100 to 4,470)	8.6 (7.0 to 10.3)	87(56 to 125)	2.3 (1.5 to 3.3)	1,450(1,070 to 1,940)	38.0 (28.0 to 50.6)
Costa Rica	35(26 to 45)	0.7 (0.6 to 1.0)	366(303 to 437)	8.8 (7.2 to 10.7)	4(3 to 6)	1.3 (0.8 to 1.9)	129(95 to 177)	38.0 (27.9 to 52.1)
El Salvador	52(38 to 69)	0.9 (0.6 to 1.1)	474(391 to 568)	7.8 (6.4 to 9.3)	6(3 to 9)	1.0 (0.6 to 1.5)	152(108 to 212)	26.6 (19.0 to 37.1)

	All Ages				Under 5			
Location	Number of Deaths (95% CI)	Age-Standardized Mortality Rate (95% CI)	Number of Cases (95% CI)	Age-Standardized Incidence Rate (95% CI)	Number of Deaths (95% CI)	Age-Standardized Mortality Rate (95% CI)	Number of Cases (95% CI)	Age-Standardized Incidence Rate (95% CI)
Guatemala	242(187 to 312)	1.6 (1.3 to 2.1)	1,700(1,410 to 2,030)	9.6 (8.1 to 11.3)	53(36 to 75)	2.6 (1.8 to 3.8)	617(455 to 844)	31.0 (22.9 to 42.4)
Honduras	109(74 to 143)	1.5 (1.0 to 2.0)	953(771 to 1,170)	9.3 (7.7 to 11.2)	24(13 to 40)	2.1 (1.1 to 3.6)	446(313 to 623)	39.5 (27.7 to 55.2)
Mexico	522(447 to 600)	0.4 (0.4 to 0.5)	9,200(7,050 to 11,600)	7.8 (6.0 to 9.8)	93(67 to 125)	0.9 (0.6 to 1.2)	2,750(1,770 to 4,290)	26.3 (16.9 to 41.0)
Nicaragua	44(35 to 54)	0.8 (0.6 to 0.9)	485(391 to 590)	7.4 (6.0 to 8.9)	15(10 to 21)	2.2 (1.5 to 3.1)	196(144 to 267)	29.7 (21.8 to 40.6)
Panama	36(26 to 47)	0.9 (0.6 to 1.2)	361(298 to 434)	9.0 (7.4 to 10.8)	11(7 to 17)	3.0 (1.9 to 4.4)	147(108 to 202)	38.1 (28.0 to 52.4)
Venezuela (Bolivarian Republic of)	254(188 to 337)	0.9 (0.7 to 1.3)	2,650(2,190 to 3,180)	10.2 (8.5 to 12.3)	71(46 to 103)	3.1 (2.0 to 4.5)	1,030(765 to 1,380)	45.1 (33.4 to 60.0)
Tropical Latin America	2,070 (1,870 to 2,290)	1.0 (0.9 to 1.1)	58,700 (47,800 to 70,600)	28.1 (22.8 to 34.0)	613 (472 to 767)	3.8 (2.9 to 4.8)	10,900 (7,730 to 15,900)	67.7 (47.9 to 98.5)
Brazil	2,010 (1,820 to 2,200)	1.0 (0.9 to 1.1)	57,100 (46,500 to 68,800)	28.2 (22.9 to 34.2)	595 (464 to 743)	3.8 (3.0 to 4.8)	10,300 (7,280 to 15,000)	66.5 (47.0 to 96.9)
Paraguay	65(47 to 86)	1.0 (0.8 to 1.4)	1,560(1,260 to 1,880)	22.9 (18.7 to 27.8)	18(11 to 29)	2.9 (1.7 to 4.5)	622(451 to 862)	97.7 (70.8 to 135.5)
**North Africa and Middle East**	**6,280 (5,330 to 7,400)**	**1.2 (1.0 to 1.4)**	**129,000 (107,000 to 153,000)**	**22.5 (18.9 to 26.4)**	**2,310 (1,740 to 3,100)**	**3.9 (2.9 to 5.2)**	**48,800 (35,500 to 67,700)**	**81.7 (59.4 to 113.4)**
Afghanistan	1,560 (1,130 to 2,160)	4.3 (3.1 to 5.7)	25,200 (21,800 to 28,900)	86.2 (76.3 to 96.4)	953 (618 to 1,500)	14.3 (9.3 to 22.5)	8,490 (6,480 to 11,000)	127.8 (97.6 to 165.3)
Algeria	292(234 to 363)	0.9 (0.7 to 1.1)	7,860(6,440 to 9,450)	19.8 (16.4 to 23.5)	64(41 to 101)	1.5 (1.0 to 2.4)	3,310(2,320 to 4,700)	77.3 (54.3 to 109.7)
Bahrain	3(3 to 4)	0.4 (0.3 to 0.5)	208(165 to 256)	19.5 (15.7 to 23.5)	0(0 to 1)	0.5 (0.4 to 0.7)	42(28 to 63)	59.9 (39.2 to 88.6)
Egypt	764 (512 to 1,070)	0.8 (0.6 to 1.2)	21,600 (17,600 to 26,800)	21.9 (18.1 to 26.7)	246 (129 to 401)	2.3 (1.2 to 3.7)	9,330 (6,390 to 13,600)	86.2 (59.0 to 126.0)
Iran (Islamic Republic of)	577(515 to 647)	0.8 (0.7 to 0.9)	19,800(16,100 to 24,000)	25.8 (21.1 to 31.0)	80(54 to 110)	1.1 (0.8 to 1.6)	5,930(4,070 to 8,710)	83.9 (57.6 to 123.3)
Iraq	427(336 to 535)	1.3 (1.0 to 1.5)	9,390(7,790 to 11,100)	22.9 (19.3 to 26.8)	143(93 to 218)	3.1 (2.0 to 4.7)	4,550(3,380 to 6,120)	97.7 (72.6 to 131.3)

Location	All Ages				Under 5			
	Number of Deaths (95% CI)	Age-Standardized Mortality Rate (95% CI)	Number of Cases (95% CI)	Age-Standardized Incidence Rate (95% CI)	Number of Deaths (95% CI)	Age-Standardized Mortality Rate (95% CI)	Number of Cases (95% CI)	Age-Standardized Incidence Rate (95% CI)
Jordan	83(66 to 104)	0.9 (0.8 to 1.2)	3,030(2,460 to 3,740)	27.4 (22.5 to 33.1)	27(18 to 41)	2.2 (1.5 to 3.4)	1,170(842 to 1,670)	97.2 (70.2 to 139.2)
Kuwait	12(10 to 15)	0.4 (0.3 to 0.5)	669(534 to 826)	19.2 (15.6 to 23.2)	2(2 to 3)	0.8 (0.5 to 1.1)	219(154 to 317)	73.2 (51.5 to 106.2)
Lebanon	34(25 to 45)	0.7 (0.5 to 0.9)	1,020(846 to 1,220)	20.0 (16.5 to 23.7)	4(2 to 7)	0.8 (0.4 to 1.3)	353(240 to 501)	69.6 (47.4 to 98.9)
Libya	43(32 to 56)	0.8 (0.6 to 1.0)	965(783 to 1,160)	17.7 (14.6 to 21.4)	5(3 to 7)	1.1 (0.7 to 1.6)	245(164 to 357)	57.7 (38.6 to 84.3)
Morocco	400(288 to 511)	1.3 (1.0 to 1.7)	7,010(5,790 to 8,280)	22.1 (18.5 to 26.2)	109(52 to 195)	3.6 (1.7 to 6.4)	2,800(1,970 to 3,930)	91.7 (64.6 to 129.1)
Oman	14(12 to 16)	0.9 (0.8 to 1.1)	274(216 to 341)	8.6 (7.1 to 10.4)	3(2 to 4)	0.7 (0.5 to 1.1)	126(79 to 191)	32.3 (20.1 to 48.7)
Palestine	28(23 to 33)	0.9 (0.8 to 1.1)	912(725 to 1,120)	18.6 (15.5 to 22.2)	10(7 to 15)	1.7 (1.1 to 2.4)	392(274 to 557)	62.9 (44.0 to 89.3)
Qatar	5(4 to 7)	0.6 (0.4 to 0.8)	1,260(987 to 1,590)	56.3 (46.3 to 68.2)	1(1 to 1)	0.7 (0.5 to 1.0)	212(150 to 310)	149.9 (106.3 to 218.7)
Saudi Arabia	180(134 to 240)	0.9 (0.7 to 1.1)	4,380(3,420 to 5,390)	17.0 (13.6 to 20.6)	4(2 to 6)	0.2 (0.1 to 0.3)	1,170(736 to 1,790)	51.5 (32.3 to 78.5)
Sudan	662(452 to 951)	1.9 (1.3 to 2.5)	5,970(4,870 to 7,160)	14.0 (11.9 to 16.4)	333(164 to 621)	6.0 (3.0 to 11.3)	3,470(2,560 to 4,540)	63.0 (46.5 to 82.3)
Syrian Arab Republic	214(165 to 276)	1.7 (1.4 to 2.2)	3,020(2,510 to 3,580)	23.7 (19.9 to 27.8)	49(30 to 74)	4.3 (2.7 to 6.5)	911(681 to 1,250)	80.1 (59.9 to 109.7)
Tunisia	85(61 to 115)	0.8 (0.6 to 1.0)	2,270(1,850 to 2,670)	21.8 (17.9 to 25.8)	12(7 to 18)	1.4 (0.8 to 2.1)	649(458 to 916)	74.7 (52.7 to 105.4)
Turkey	351(288 to 422)	0.5 (0.4 to 0.6)	4,630(3,850 to 5,450)	6.7 (5.5 to 8.0)	68(48 to 96)	1.4 (1.0 to 2.0)	1,130(778 to 1,640)	23.1 (15.9 to 33.6)
United Arab Emirates	54(32 to 80)	0.8 (0.5 to 1.2)	1,240(997 to 1,520)	28.2 (24.1 to 33.0)	1(1 to 3)	0.4 (0.2 to 0.8)	209(140 to 324)	62.6 (42.0 to 97.2)
Yemen	479(330 to 684)	2.0 (1.4 to 2.7)	7,680(6,260 to 9,390)	23.5 (20.0 to 27.6)	196(106 to 358)	4.4 (2.4 to 8.0)	4,030(2,940 to 5,620)	90.6 (66.2 to 126.2)
**South Asia**	**55,500 (48,400 to 64,400)**	**3.5 (3.0 to 4.0)**	**742,000 (610,000 to 883,000)**	**43.6 (36.2 to 52.3)**	**21,400 (16,200 to 28,200)**	**13.0 (9.9 to 17.1)**	**338,000 (245,000 to 461,000)**	**205.7 (148.8 to 280.3)**
Bangladesh	2,320 (1,840 to 2,830)	1.6 (1.3 to 2.0)	35,700 (29,000 to 43,000)	23.9 (19.5 to 28.8)	257 (117 to 445)	1.9 (0.9 to 3.2)	13,000 (8,740 to 18,400)	94.3 (63.5 to 134.1)
Bhutan	22(14 to 31)	3.4 (2.3 to 4.8)	238(196 to 283)	34.5 (28.5 to 41.2)	9(5 to 15)	13.7 (6.9 to 22.3)	103(78 to 139)	157.7 (119.0 to 212.0)

	All Ages				Under 5			
Location	Number of Deaths (95% CI)	Age-Standardized Mortality Rate (95% CI)	Number of Cases (95% CI)	Age-Standardized Incidence Rate (95% CI)	Number of Deaths (95% CI)	Age-Standardized Mortality Rate (95% CI)	Number of Cases (95% CI)	Age-Standardized Incidence Rate (95% CI)
India	34,700 (29,700 to 40,000)	2.9 (2.5 to 3.3)	552,000 (455,000 to 654,000)	43.6 (36.1 to 52.0)	10,300 (7,450 to 13,700)	8.8 (6.4 to 11.7)	229,000 (166,000 to 315,000)	195.6 (141.5 to 269.1)
Nepal	469(360 to 603)	2.4 (1.8 to 3.0)	6,060(4,880 to 7,550)	21.0 (17.1 to 25.9)	154(92 to 249)	5.1 (3.1 to 8.3)	3,260(2,320 to 4,600)	108.6 (77.3 to 153.4)
Pakistan	18,000 (14,100 to 22,900)	7.9 (6.5 to 9.7)	148,000 (119,000 to 183,000)	55.9 (46.1 to 67.4)	10,600 (7,220 to 15,400)	34.8 (23.7 to 50.3)	92,800 (67,400 to 124,000)	304.1 (220.7 to 405.2)
**Southeast Asia, East Asia, and Oceania**	**18,200 (16,300 to 20,500)**	**1.0 (0.9 to 1.2)**	**177,000 (147,000 to 212,000)**	**10.9 (8.8 to 13.3)**	**6,500 (5,300 to 7,940)**	**4.6 (3.8 to 5.7)**	**80,600 (57,400 to 110,000)**	**57.4 (40.9 to 78.6)**
East Asia	6,790 (5,940 to 7,680)	0.5 (0.5 to 0.6)	50,700 (41,600 to 60,300)	4.8 (3.9 to 6.0)	1,510 (1,210 to 1,840)	1.8 (1.4 to 2.2)	21,200 (14,500 to 30,300)	25.2 (17.2 to 36.1)
China	6,460 (5,620 to 7,340)	0.5 (0.4 to 0.6)	47,600 (39,000 to 56,700)	4.7 (3.7 to 5.8)	1,460 (1,180 to 1,770)	1.8 (1.4 to 2.2)	20,100 (13,700 to 28,800)	24.6 (16.8 to 35.3)
Democratic People's Republic of Korea	223(166 to 295)	0.9 (0.7 to 1.2)	1,930(1,580 to 2,290)	9.4 (7.6 to 11.4)	45(26 to 73)	2.7 (1.6 to 4.4)	807(581 to 1,090)	48.5 (34.9 to 65.6)
Taiwan (Province of China)	105(82 to 135)	0.3 (0.3 to 0.4)	1,130(933 to 1,370)	7.0 (5.5 to 9.0)	4(3 to 5)	0.4 (0.3 to 0.5)	318(214 to 478)	32.4 (21.8 to 48.7)
Oceania	693(497 to 937)	4.4 (3.3 to 5.7)	6,690(5,650 to 7,860)	46.0 (39.6 to 52.8)	469(305 to 687)	25.3 (16.5 to 37.1)	3,780(2,920 to 4,840)	204.2 (157.6 to 261.6)
American Samoa	1(1 to 1)	1.4 (1.1 to 1.7)	22(18 to 26)	42.9 (36.1 to 50.5)	0(0 to 0)	2.6 (1.5 to 4.0)	7(5 to 10)	134.9 (95.7 to 189.6)
Cook Islands	0(0 to 0)	0.2 (0.1 to 0.2)	6(5 to 8)	36.7 (29.7 to 44.4)	0(0 to 0)	0.1 (0.1 to 0.2)	2(1 to 2)	114.0 (74.2 to 170.3)
Fiji	13(10 to 18)	1.6 (1.2 to 2.1)	284(236 to 339)	34.2 (28.9 to 40.3)	4(2 to 6)	4.3 (2.7 to 6.8)	102(75 to 144)	115.5 (84.1 to 162.4)
Guam	2(1 to 2)	1.0 (0.8 to 1.3)	75(63 to 88)	44.6 (37.4 to 52.7)	1(0 to 1)	3.2 (2.1 to 4.6)	25(18 to 34)	156.1 (114.1 to 213.8)
Kiribati	11(9 to 14)	10.5 (8.4 to 12.6)	76(66 to 88)	66.1 (57.7 to 75.1)	3(2 to 4)	18.1 (10.8 to 27.7)	26(20 to 35)	180.3 (134.3 to 236.1)
Marshall Islands	1(1 to 1)	2.0 (1.4 to 2.7)	18(15 to 22)	35.6 (30.3 to 42.1)	0(0 to 0)	3.4 (2.0 to 5.6)	7(5 to 10)	116.3 (84.3 to 165.1)
Micronesia (Federated States of)	2(1 to 2)	1.8 (1.2 to 2.4)	30(25 to 36)	33.2 (27.8 to 38.6)	0(0 to 0)	2.0 (1.1 to 3.4)	10(7 to 14)	104.4 (73.8 to 144.8)
Nauru	0(0 to 0)	2.3 (1.8 to 2.9)	4(3 to 4)	33.5 (28.5 to 39.3)	0(0 to 0)	7.7 (4.7 to 11.4)	2(1 to 2)	117.1 (85.6 to 166.5)

Location	All Ages				Under 5			
	Number of Deaths (95% CI)	Age-Standardized Mortality Rate (95% CI)	Number of Cases (95% CI)	Age-Standardized Incidence Rate (95% CI)	Number of Deaths (95% CI)	Age-Standardized Mortality Rate (95% CI)	Number of Cases (95% CI)	Age-Standardized Incidence Rate (95% CI)
Niue	0(0 to 0)	1.0 (0.7 to 1.3)	0(0 to 1)	28.0 (23.3 to 33.5)	0(0 to 0)	3.0 (1.8 to 4.9)	0(0 to 0)	92.2 (64.9 to 133.5)
Northern Mariana Islands	0(0 to 0)	0.9 (0.7 to 1.1)	15(12 to 17)	43.0 (36.0 to 51.2)	0(0 to 0)	1.9 (1.2 to 2.8)	3(2 to 5)	142.0 (102.9 to 200.2)
Palau	0(0 to 0)	0.8 (0.7 to 1.0)	5(4 to 5)	32.2 (26.8 to 38.2)	0(0 to 0)	1.3 (0.8 to 2.1)	1(1 to 1)	95.0 (67.1 to 137.6)
Papua New Guinea	599(423 to 830)	4.9 (3.5 to 6.4)	5,380(4,510 to 6,330)	47.8 (41.0 to 54.8)	432(279 to 637)	29.8 (19.3 to 44.0)	3,230(2,480 to 4,100)	222.9 (171.4 to 282.7)
Samoa	2(2 to 3)	1.3 (0.9 to 1.7)	75(61 to 90)	37.7 (31.3 to 44.6)	0(0 to 1)	1.8 (1.0 to 2.9)	24(17 to 35)	115.7 (80.5 to 165.9)
Solomon Islands	17(11 to 22)	2.7 (1.8 to 3.5)	218(181 to 259)	32.7 (27.9 to 38.0)	4(2 to 6)	3.7 (2.2 to 6.2)	93(66 to 127)	96.4 (69.1 to 132.5)
Tokelau	0(0 to 0)	1.1 (0.8 to 1.5)	1(0 to 1)	39.7 (33.2 to 46.7)	0(0 to 0)	2.0 (1.2 to 3.2)	0(0 to 0)	122.1 (85.8 to 173.1)
Tonga	5(4 to 7)	5.3 (4.0 to 7.0)	55(48 to 65)	54.0 (46.4 to 62.1)	2(1 to 2)	13.2 (7.4 to 20.3)	21(16 to 28)	172.6 (129.5 to 227.8)
Tuvalu	0(0 to 0)	1.6 (1.2 to 2.1)	4(3 to 4)	34.8 (29.2 to 41.0)	0(0 to 0)	2.8 (1.6 to 4.6)	1(1 to 2)	113.8 (82.2 to 156.4)
Vanuatu	6(4 to 8)	2.2 (1.5 to 2.9)	112(93 to 137)	38.2 (32.5 to 44.9)	2(1 to 3)	5.0 (2.9 to 8.2)	48(35 to 64)	126.3 (93.0 to 171.2)
Southeast Asia	10,700 (9,320 to 12,400)	1.8 (1.6 to 2.1)	120,000 (98,900 to 144,000)	20.1 (16.6 to 24.4)	4,520 (3,510 to 5,800)	8.3 (6.4 to 10.6)	55,600 (40,200 to 75,600)	102.1 (73.8 to 138.7)
Cambodia	129(89 to 190)	0.8 (0.5 to 1.1)	2,800(2,200 to 3,460)	16.1 (12.8 to 19.9)	106(66 to 166)	6.0 (3.8 to 9.4)	1,560(1,120 to 2,140)	88.0 (63.1 to 120.9)
Indonesia	4,720 (3,910 to 5,600)	2.3 (1.9 to 2.7)	38,800 (31,200 to 48,100)	18.1 (14.4 to 22.7)	2,140 (1,530 to 2,940)	10.9 (7.8 to 14.9)	21,400 (15,100 to 29,800)	108.8 (76.4 to 151.1)
Lao People's Democratic Republic	162(108 to 236)	2.2 (1.5 to 3.2)	1,550(1,280 to 1,880)	20.1 (16.7 to 24.2)	91(51 to 154)	11.4 (6.3 to 19.2)	898(672 to 1,170)	112.0 (83.9 to 146.5)
Malaysia	291(229 to 367)	1.0 (0.8 to 1.3)	5,590(4,500 to 6,800)	19.7 (15.8 to 24.3)	34(21 to 50)	1.3 (0.8 to 1.9)	2,460(1,770 to 3,480)	95.6 (69.0 to 135.6)
Maldives	2(1 to 2)	0.5 (0.4 to 0.6)	79(62 to 98)	17.5 (13.9 to 21.8)	1(0 to 1)	1.3 (0.8 to 2.1)	31(21 to 45)	74.8 (51.8 to 108.8)
Mauritius	5(4 to 7)	0.4 (0.3 to 0.6)	164(132 to 198)	18.7 (14.7 to 23.5)	1(1 to 1)	1.6 (1.1 to 2.2)	65(47 to 92)	100.6 (73.5 to 142.4)
Myanmar	1,250 (828 to 1,840)	2.5 (1.7 to 3.7)	11,300 (9,250 to 13,400)	21.9 (18.0 to 26.0)	934 (559 to 1,460)	18.6 (11.1 to 29.0)	6,750 (5,000 to 8,760)	134.6 (99.7 to 174.6)

	All Ages				Under 5			
**Location**	**Number of Deaths (95% CI)**	**Age-Standardized Mortality Rate (95% CI)**	**Number of Cases (95% CI)**	**Age-Standardized Incidence Rate (95% CI)**	**Number of Deaths (95% CI)**	**Age-Standardized Mortality Rate (95% CI)**	**Number of Cases (95% CI)**	**Age-Standardized Incidence Rate (95% CI)**
Philippines	2,060 (1,730 to 2,400)	1.8 (1.5 to 2.1)	27,700 (22,600 to 33,200)	22.9 (18.8 to 27.4)	862 (641 to 1,130)	6.8 (5.1 to 8.9)	11,900 (8,620 to 16,500)	94.0 (68.1 to 130.1)
Seychelles	2(2 to 3)	2.3 (2.0 to 2.7)	21(17 to 25)	23.7 (19.7 to 29.2)	0(0 to 0)	2.2 (1.4 to 3.1)	8(6 to 12)	112.7 (80.9 to 157.3)
Sri Lanka	297(220 to 392)	1.4 (1.1 to 1.9)	9,110(7,460 to 11,000)	48.0 (39.2 to 58.5)	21(12 to 31)	1.4 (0.8 to 2.0)	2,690(1,980 to 3,750)	173.8 (128.1 to 242.6)
Thailand	1,040 (788 to 1,340)	1.3 (1.0 to 1.6)	13,900 (11,500 to 16,400)	26.5 (21.4 to 32.2)	35 (20 to 52)	1.1 (0.7 to 1.7)	3,230 (2,310 to 4,480)	103.3 (74.0 to 143.4)
Timor-Leste	31(18 to 41)	2.0 (1.2 to 2.7)	316(255 to 381)	19.9 (16.3 to 23.8)	19(10 to 28)	10.8 (5.6 to 16.2)	177(134 to 231)	100.5 (76.0 to 131.5)
Viet Nam	747(569 to 956)	0.9 (0.7 to 1.2)	8,270(6,730 to 10,200)	10.7 (8.6 to 13.5)	273(154 to 434)	3.9 (2.2 to 6.3)	4,340(3,060 to 6,110)	62.5 (44.0 to 88.1)
**Sub-Saharan Africa**	**144,000 (117,000 to 176,000)**	**14.3 (12.2 to 16.7)**	**1,200,000 (998,000 to 1,450,000)**	**96.3 (82.6 to 111.7)**	**79,200 (59,200 to 104,000)**	**47.8 (35.8 to 63.0)**	**743,000 (561,000 to 982,000)**	**448.3 (338.6 to 592.9)**
Central Sub-Saharan Africa	10,000 (7,720 to 12,900)	8.9 (6.8 to 11.2)	128,000 (108,000 to 153,000)	87.2 (75.6 to 99.6)	3,400 (2,240 to 5,270)	16.4 (10.8 to 25.5)	70,500 (53,700 to 93,600)	340.6 (259.4 to 452.4)
Angola	2,520 (1,790 to 3,340)	9.2 (6.8 to 12.0)	31,600 (26,700 to 37,600)	90.1 (78.0 to 103.1)	1,060 (672 to 1,600)	20.7 (13.1 to 31.1)	18,000 (13,700 to 23,600)	349.1 (266.8 to 458.0)
Central African Republic	902(652 to 1,210)	17.9 (12.9 to 23.8)	8,310(7,000 to 9,700)	136.6 (117.4 to 154.4)	384(232 to 589)	45.6 (27.6 to 70.0)	4,640(3,530 to 5,970)	551.4 (419.9 to 709.5)
Congo	284(211 to 379)	6.9 (5.3 to 9.0)	4,030(3,400 to 4,830)	73.8 (63.3 to 85.8)	56(35 to 92)	8.0 (5.0 to 13.2)	1,940(1,440 to 2,610)	278.6 (206.5 to 375.8)
Democratic Republic of the Congo	6,150 (4,590 to 8,110)	8.5 (6.3 to 11.0)	81,700 (68,700 to 98,600)	84.4 (72.8 to 96.8)	1,860 (1,030 to 3,390)	13.6 (7.6 to 24.9)	44,700 (33,500 to 59,800)	328.2 (246.0 to 439.0)
Equatorial Guinea	55(34 to 85)	5.4 (3.5 to 7.9)	1,080(899 to 1,340)	74.1 (63.5 to 87.9)	17(9 to 30)	9.4 (4.9 to 16.3)	580(428 to 824)	313.3 (231.3 to 445.4)
Gabon	86(63 to 114)	6.0 (4.5 to 7.9)	1,390(1,170 to 1,690)	79.1 (67.3 to 94.1)	18(10 to 30)	8.8 (4.9 to 14.6)	660(493 to 890)	326.9 (243.9 to 440.7)
Eastern Sub-Saharan Africa	42,400 (35,800 to 50,700)	13.2 (11.5 to 15.1)	436,000 (364,000 to 525,000)	97.3 (83.4 to 112.3)	17,100 (12,700 to 23,000)	26.7 (19.8 to 35.9)	245,000 (184,000 to 327,000)	382.0 (287.5 to 509.2)
Burundi	1,240(892 to 1,680)	13.1 (9.7 to 17.0)	11,900(9,820 to 14,400)	87.0 (75.0 to 99.7)	450(236 to 815)	21.6 (11.3 to 39.2)	7,130(5,330 to 9,350)	342.9 (256.4 to 449.6)

Location	All Ages				Under 5			
	Number of Deaths (95% CI)	Age-Standardized Mortality Rate (95% CI)	Number of Cases (95% CI)	Age-Standardized Incidence Rate (95% CI)	Number of Deaths (95% CI)	Age-Standardized Mortality Rate (95% CI)	Number of Cases (95% CI)	Age-Standardized Incidence Rate (95% CI)
Comoros	70(50 to 88)	11.6 (8.6 to 14.7)	749(638 to 871)	107.1 (91.5 to 123.1)	19(12 to 28)	23.8 (14.8 to 36.3)	320(243 to 420)	409.4 (311.2 to 538.2)
Djibouti	110(76 to 154)	11.5 (8.3 to 15.9)	1,180(1,010 to 1,400)	95.0 (81.8 to 109.2)	40(25 to 62)	25.4 (15.9 to 38.8)	586(441 to 762)	368.1 (277.4 to 478.4)
Eritrea	693(487 to 952)	14.8 (10.9 to 19.8)	7,070(5,930 to 8,560)	106.0 (91.6 to 122.1)	187(111 to 298)	20.2 (11.9 to 32.0)	3,350(2,480 to 4,560)	360.8 (266.8 to 490.4)
Ethiopia	11,300 (9,370 to 13,600)	14.1 (12.0 to 16.5)	117,000 (95,900 to 143,000)	99.3 (83.9 to 115.8)	4,210 (3,020 to 5,870)	25.2 (18.1 to 35.2)	64,600 (47,400 to 88,300)	387.5 (284.1 to 529.2)
Kenya	4,400 (3,660 to 5,400)	13.1 (10.9 to 15.9)	47,500 (39,400 to 56,200)	98.1 (82.9 to 113.8)	916 (646 to 1,310)	14.3 (10.1 to 20.4)	20,000 (14,300 to 27,900)	312.4 (222.3 to 434.9)
Madagascar	2,080 (1,570 to 2,610)	10.4 (7.7 to 13.1)	23,900 (20,300 to 28,600)	86.5 (74.6 to 99.7)	630 (407 to 922)	16.0 (10.3 to 23.4)	12,200 (9,110 to 16,400)	310.6 (231.4 to 417.1)
Malawi	2,370 (1,840 to 3,030)	16.5 (13.3 to 20.3)	18,400 (15,500 to 21,800)	98.0 (84.3 to 111.9)	892 (570 to 1,360)	34.5 (22.1 to 52.8)	9,850 (7,340 to 12,900)	380.9 (283.7 to 498.5)
Mozambique	2,730 (2,010 to 3,680)	12.3 (9.4 to 15.8)	26,500 (22,000 to 32,000)	81.8 (70.3 to 94.2)	1,230 (785 to 1,940)	24.0 (15.4 to 37.9)	15,200 (11,300 to 20,500)	297.2 (222.0 to 400.8)
Rwanda	995(752 to 1,300)	10.1 (7.9 to 12.5)	10,200(8,410 to 12,100)	78.6 (66.9 to 91.3)	361(206 to 590)	21.8 (12.5 to 35.7)	5,480(4,000 to 7,320)	332.2 (242.5 to 443.7)
Somalia	4,670 (3,340 to 6,720)	26.3 (18.4 to 38.7)	35,300 (29,500 to 41,600)	151.4 (131.1 to 172.6)	2,410 (1,490 to 3,630)	64.7 (39.8 to 97.3)	20,700 (15,600 to 26,500)	555.8 (418.2 to 710.6)
South Sudan	1,970 (1,350 to 2,780)	19.2 (13.9 to 25.8)	28,200 (23,600 to 33,900)	257.1 (221.2 to 297.7)	1,400 (839 to 2,130)	92.3 (55.4 to 140.6)	17,800 (13,300 to 23,300)	1172.2 (878.3 to 1533.5)
Uganda	3,940 (2,810 to 5,380)	11.0 (8.6 to 13.7)	43,500 (35,600 to 53,700)	87.1 (74.5 to 101.1)	2,160 (1,240 to 3,470)	30.4 (17.5 to 48.9)	29,400 (22,300 to 39,100)	414.2 (314.8 to 551.5)
United Republic of Tanzania	3,760 (2,910 to 4,910)	8.1 (6.5 to 10.0)	46,400 (37,900 to 56,700)	70.7 (59.8 to 81.9)	1,620 (1,000 to 2,620)	17.4 (10.8 to 28.2)	28,500 (21,000 to 38,400)	306.4 (225.8 to 413.0)
Zambia	2,060 (1,590 to 2,670)	15.6 (12.1 to 19.5)	18,400 (15,500 to 21,800)	96.5 (83.8 to 110.1)	609 (376 to 963)	21.6 (13.3 to 34.1)	9,620 (7,300 to 12,900)	341.1 (259.1 to 457.4)

	All Ages				Under 5			
Location	Number of Deaths (95% CI)	Age-Standardized Mortality Rate (95% CI)	Number of Cases (95% CI)	Age-Standardized Incidence Rate (95% CI)	Number of Deaths (95% CI)	Age-Standardized Mortality Rate (95% CI)	Number of Cases (95% CI)	Age-Standardized Incidence Rate (95% CI)
Southern Sub-Saharan Africa	4,130 (3,520 to 4,770)	6.1 (5.3 to 6.9)	34,000 (29,000 to 39,500)	45.8 (39.3 to 52.6)	784 (571 to 1,080)	9.7 (7.1 to 13.3)	11,300 (8,330 to 15,200)	139.5 (102.9 to 187.2)
Botswana	123(85 to 166)	6.5 (4.5 to 8.6)	1,190(1,000 to 1,400)	54.7 (46.8 to 63.2)	28(15 to 46)	11.7 (6.4 to 19.4)	408(299 to 555)	172.4 (126.5 to 234.6)
Eswatini	73(52 to 98)	7.9 (5.7 to 10.4)	441(384 to 515)	40.5 (35.5 to 46.3)	19(11 to 29)	13.3 (7.9 to 20.8)	150(113 to 199)	106.7 (80.6 to 141.8)
Lesotho	162(116 to 214)	9.6 (6.8 to 12.6)	1,090(927 to 1,240)	55.2 (47.4 to 62.4)	27(18 to 41)	12.5 (8.1 to 18.8)	320(237 to 424)	146.4 (108.4 to 194.2)
Namibia	100(71 to 133)	5.3 (4.0 to 6.9)	929(787 to 1,100)	39.7 (34.4 to 45.4)	21(12 to 34)	7.1 (4.1 to 11.5)	367(270 to 506)	123.5 (91.1 to 170.5)
South Africa	2,220 (1,970 to 2,480)	4.6 (4.1 to 5.1)	21,200 (17,700 to 24,800)	41.1 (34.6 to 47.9)	334 (248 to 441)	6.6 (4.9 to 8.7)	6,360 (4,500 to 8,760)	124.9 (88.3 to 172.0)
Zimbabwe	1,450(1,050 to 1,910)	12.6 (8.9 to 16.5)	9,170(7,900 to 10,700)	62.9 (55.2 to 71.4)	355(223 to 555)	16.8 (10.5 to 26.3)	3,690(2,810 to 4,820)	174.9 (133.1 to 228.3)
Western Sub-Saharan Africa	87,600 (68,300 to 110,000)	18.4 (15.0 to 22.1)	602,000 (494,000 to 737,000)	105.8 (90.1 to 124.1)	57,800 (42,700 to 76,000)	79.5 (58.8 to 104.4)	416,000 (314,000 to 546,000)	571.7 (431.4 to 751.1)
Benin	1,980 (1,340 to 2,840)	15.2 (11.2 to 20.7)	13,500 (11,000 to 16,500)	84.4 (72.3 to 97.8)	1,240 (763 to 1,940)	55.8 (34.2 to 87.2)	9,280 (6,920 to 12,200)	416.7 (310.4 to 546.6)
Burkina Faso	5,260 (3,720 to 7,530)	21.2 (16.2 to 27.6)	27,000 (22,600 to 31,900)	95.4 (83.8 to 107.5)	3,460 (2,140 to 5,570)	85.5 (53.0 to 137.7)	17,900 (13,700 to 22,500)	442.0 (338.2 to 556.1)
Cabo Verde	18(15 to 21)	3.7 (3.1 to 4.4)	584(491 to 696)	108.5 (91.5 to 129.3)	2(1 to 3)	4.1 (1.9 to 6.5)	255(188 to 348)	479.4 (353.7 to 655.3)
Cameroon	2,790 (1,930 to 3,760)	11.7 (8.4 to 15.6)	34,700 (28,600 to 42,100)	110.8 (94.8 to 128.6)	1,180 (692 to 1,870)	28.2 (16.4 to 44.4)	20,500 (15,100 to 27,600)	486.8 (358.4 to 656.3)
Chad	4,620 (3,260 to 6,300)	24.1 (18.3 to 30.7)	29,800 (24,400 to 35,600)	127.7 (110.1 to 145.4)	3,200 (1,920 to 4,760)	95.9 (57.7 to 142.8)	21,900 (16,700 to 27,200)	655.9 (501.1 to 816.7)
Côte d'Ivoire	2,260 (1,540 to 3,080)	10.7 (7.7 to 14.1)	23,400 (19,600 to 28,100)	79.1 (67.9 to 90.6)	925 (502 to 1,530)	23.1 (12.5 to 38.0)	14,500 (10,900 to 18,800)	360.5 (271.3 to 468.2)
Gambia	212(156 to 280)	13.6 (10.1 to 17.4)	1,980(1,650 to 2,390)	83.6 (71.9 to 97.1)	54(30 to 89)	16.3 (9.2 to 26.9)	1,060(781 to 1,430)	320.3 (236.9 to 434.5)

	All Ages				Under 5			
Location	Number of Deaths (95% CI)	Age-Standardized Mortality Rate (95% CI)	Number of Cases (95% CI)	Age-Standardized Incidence Rate (95% CI)	Number of Deaths (95% CI)	Age-Standardized Mortality Rate (95% CI)	Number of Cases (95% CI)	Age-Standardized Incidence Rate (95% CI)
Ghana	3,490 (2,550 to 4,480)	14.8 (11.3 to 18.5)	25,100 (21,600 to 28,800)	82.0 (72.0 to 91.7)	817 (441 to 1,360)	20.6 (11.1 to 34.1)	12,100 (9,220 to 15,800)	304.7 (232.2 to 397.2)
Guinea	3,260 (2,260 to 4,530)	23.9 (17.7 to 31.2)	26,600 (22,100 to 32,100)	173.4 (149.4 to 201.8)	2,060 (1,290 to 3,180)	96.3 (60.0 to 148.6)	17,200 (13,100 to 22,600)	801.0 (611.0 to 1052.2)
Guinea-Bissau	267(203 to 353)	18.9 (14.4 to 24.5)	3,860(3,210 to 4,660)	189.0 (162.9 to 218.4)	89(53 to 140)	30.9 (18.6 to 48.6)	2,120(1,570 to 2,900)	736.3 (545.0 to 1007.7)
Liberia	432(307 to 587)	12.1 (9.0 to 16.1)	4,080(3,390 to 4,990)	83.3 (71.5 to 97.2)	128(71 to 219)	20.2 (11.2 to 34.6)	2,230(1,640 to 2,980)	351.5 (258.4 to 470.4)
Mali	6,260 (4,410 to 8,570)	25.4 (19.0 to 33.3)	38,000 (31,200 to 45,900)	125.7 (108.3 to 144.1)	4,100 (2,650 to 5,870)	100.8 (65.2 to 144.5)	27,300 (20,900 to 35,300)	671.9 (514.0 to 867.4)
Mauritania	217(147 to 307)	7.4 (5.3 to 9.9)	2,670(2,220 to 3,250)	62.7 (53.7 to 74.2)	53(29 to 93)	10.0 (5.5 to 17.4)	1,580(1,170 to 2,130)	297.3 (220.3 to 401.5)
Niger	7,770 (5,120 to 11,200)	24.5 (17.5 to 32.9)	48,900 (40,300 to 59,300)	141.5 (122.0 to 163.5)	6,250 (3,860 to 9,400)	128.9 (79.5 to 193.7)	37,600 (29,200 to 47,800)	775.4 (602.3 to 985.9)
Nigeria	44,900 (33,700 to 58,200)	18.9 (14.9 to 23.6)	287,000 (230,000 to 361,000)	101.8 (83.9 to 123.8)	32,700 (23,500 to 44,200)	97.5 (70.2 to 131.9)	211,000 (156,000 to 285,000)	629.8 (466.1 to 848.8)
Sao Tome and Principe	5(4 to 6)	3.4 (2.5 to 4.5)	114(93 to 140)	55.1 (45.8 to 66.1)	1(1 to 2)	4.5 (2.6 to 7.6)	64(46 to 89)	273.2 (195.7 to 381.6)
Senegal	1,560 (1,130 to 2,050)	13.4 (10.1 to 17.1)	20,200 (16,900 to 24,100)	125.4 (108.2 to 144.2)	441 (265 to 715)	20.5 (12.3 to 33.2)	10,800 (7,970 to 14,500)	503.2 (370.5 to 674.6)
Sierra Leone	1,630(1,060 to 2,500)	20.0 (14.0 to 28.4)	9,170(7,620 to 10,900)	99.5 (85.7 to 114.3)	946(559 to 1,550)	76.9 (45.5 to 126.4)	5,350(4,060 to 6,970)	435.4 (330.0 to 567.2)
Togo	625(460 to 833)	10.5 (7.9 to 13.3)	5,430(4,520 to 6,540)	65.2 (56.0 to 75.5)	184(106 to 327)	16.5 (9.5 to 29.4)	3,070(2,260 to 4,140)	275.4 (202.4 to 371.4)

Appendix Table S2: Fatal and non-fatal meningitis aetiology proportions in 2019 for neonates, children under 5 years old, and all ages by super-region, region, country, and globally

Location	Aetiology	Neonatal		Under 5		All Ages	
		Nonfatal	Fatal	Nonfatal	Fatal	Nonfatal	Fatal
**Global**	*E. coli*	6.1 (4.7-7.7)	11.7 (9.2-14.6)	5.4 (4.5 - 6.5)	10.0 (8.4 - 11.9)	4.8 (4.1 - 5.6)	9.2 (7.8 - 10.7)
	Group B *Streptococcus*	20.4 (17.9-23.3)	22.8 (19.9-25.9)	8.8 (8.1 - 9.6)	10.2 (9.3 - 11.2)	7.1 (6.6 - 7.7)	8.0 (7.3 - 8.8)
	H. influenzae	9.6 (8.2-11.3)	6.6 (5.5-8.0)	10.4 (9.7 - 11.0)	6.7 (6.2 - 7.3)	7.5 (7.0 - 7.9)	4.7 (4.3 - 5.0)
	K. pneumoniae	5.4 (4.2-6.9)	17.1 (13.6-21.1)	3.8 (3.0 - 4.8)	12.0 (9.7 - 14.8)	3.8 (3.2 - 4.6)	12.2 (10.2 - 14.3)
	L. monocytogenes	1.9 (1.3-2.7)	4.7 (3.1-6.7)	2.1 (1.5 - 2.7)	4.9 (3.7 - 6.3)	2.1 (1.7 - 2.5)	5.2 (4.3 - 6.2)
	N. meningitidis	9.7 (8.3-11.4)	8.5 (7.0-10.2)	16.9 (15.9 - 18.0)	12.9 (11.7 - 13.9)	17.3 (16.5 - 18.0)	13.6 (12.7 - 14.4)
	Other	2.4 (1.5-3.5)	3.1 (2.0-4.6)	9.2 (6.6 - 12.8)	10.0 (7.2 - 13.8)	9.5 (7.7 - 11.6)	10.9 (9.0 - 13.3)
	S. aureus	4.3 (3.2-5.6)	5.7 (4.2-7.4)	5.0 (4.4 - 5.6)	6.2 (5.4 - 7.1)	5.1 (4.6 - 5.6)	6.7 (6.0 - 7.4)
	S. pneumoniae	3.0 (2.5-3.7)	4.5 (3.7-5.5)	13.3 (12.5 - 14.0)	17.3 (16.0 - 18.6)	13.0 (12.4 - 13.6)	18.1 (17.1 - 19.2)
	Virus	37.1 (34.1-40.3)	15.3 (13.5-17.2)	25.1 (24.0 - 26.3)	9.8 (9.2 - 10.5)	29.9 (28.9 - 30.8)	11.4 (10.9 - 12.0)
**Central Europe, Eastern Europe, and Central Asia**	*E. coli*	6.6 (5.5-7.7)	13.0 (11.0-15.1)	6.0 (5.2 - 6.8)	11.6 (10.4 - 12.9)	5.0 (4.6 - 5.5)	10.5 (9.7 - 11.4)
	Group B *Streptococcus*	23.7 (22.2-25.3)	26.7 (24.9-28.5)	9.3 (8.7 - 9.9)	11.9 (11.2 - 12.6)	6.8 (6.6 - 7.1)	7.7 (7.4 - 8.1)
	H. influenzae	8.2 (7.0-9.5)	5.2 (4.3-6.1)	12.0 (11.3 - 12.8)	7.1 (6.5 - 7.7)	7.2 (6.9 - 7.5)	4.1 (3.9 - 4.3)
	K. pneumoniae	5.5 (4.4-6.8)	18.4 (15.2-22.2)	3.8 (3.1 - 4.6)	13.4 (11.5 - 15.8)	4.0 (3.6 - 4.5)	14.0 (12.8 - 15.2)
	L. monocytogenes	1.9 (1.3-2.6)	4.8 (3.4-6.5)	2.3 (1.8 - 3.0)	5.8 (4.6 - 7.1)	2.3 (2.1 - 2.6)	6.7 (6.1 - 7.2)
	N. meningitidis	8.7 (7.9-9.6)	7.5 (6.6-8.4)	13.3 (12.5 - 14.1)	10.7 (9.9 - 11.4)	13.7 (13.3 - 14.1)	10.4 (9.9 - 10.8)
	Other	2.0 (1.2-3.2)	2.6 (1.6-4.1)	10.1 (7.0 - 14.0)	10.8 (7.6 - 14.7)	10.4 (8.5 - 12.6)	12.8 (10.3 - 15.7)
	S. aureus	4.2 (3.5-5.1)	5.6 (4.5-6.7)	5.0 (4.5 - 5.4)	6.2 (5.6 - 6.7)	5.0 (4.8 - 5.3)	7.0 (6.7 - 7.4)
	S. pneumoniae	2.9 (2.5-3.3)	3.9 (3.4-4.6)	12.1 (11.3 - 12.8)	14.1 (13.1 - 15.1)	11.8 (11.4 - 12.1)	15.8 (15.1 - 16.5)
	Virus	36.3 (33.7-39.0)	12.2 (10.8-13.5)	26.1 (24.8 - 27.2)	8.4 (7.9 - 9.0)	33.7 (32.7 - 34.6)	10.9 (10.5 - 11.3)
**Central Asia**	*E. coli*	6.6 (5.4-7.9)	12.9 (10.8-15.2)	6.0 (5.1 - 7.0)	11.3 (9.9 - 12.8)	4.8 (4.3 - 5.4)	9.6 (8.7 - 10.7)
	Group B *Streptococcus*	24.0 (22.0-26.1)	26.8 (24.5-29.2)	9.5 (8.8 - 10.1)	12.5 (11.6 - 13.3)	7.0 (6.6 - 7.4)	8.4 (7.9 - 8.8)
	H. influenzae	8.1 (6.8-9.6)	5.2 (4.3-6.2)	8.7 (8.1 - 9.3)	5.0 (4.5 - 5.4)	5.7 (5.4 - 6.0)	3.2 (3.0 - 3.4)
	K. pneumoniae	5.6 (4.4-7.0)	18.3 (15.0-22.1)	3.8 (3.1 - 4.7)	13.1 (11.2 - 15.4)	3.9 (3.4 - 4.4)	13.4 (11.8 - 15.0)
	L. monocytogenes	1.9 (1.3-2.6)	4.7 (3.3-6.5)	2.3 (1.7 - 2.9)	5.5 (4.3 - 6.8)	2.1 (1.9 - 2.5)	5.7 (5.1 - 6.4)
	N. meningitidis	8.2 (7.2-9.2)	7.1 (6.2-8.1)	15.0 (14.1 - 15.8)	11.7 (10.9 - 12.5)	15.8 (15.2 - 16.3)	13.0 (12.4 - 13.6)
	Other	2.1 (1.3-3.3)	2.7 (1.7-4.1)	10.0 (7.1 - 13.8)	10.1 (7.1 - 13.7)	10.0 (8.2 - 12.2)	12.0 (10.0 - 14.3)
	S. aureus	4.2 (3.4-5.2)	5.5 (4.4-6.8)	5.2 (4.6 - 5.7)	6.2 (5.6 - 6.9)	5.1 (4.7 - 5.4)	6.8 (6.4 - 7.3)

Location	Aetiology	Neonatal		Under 5		All Ages	
		Nonfatal	Fatal	Nonfatal	Fatal	Nonfatal	Fatal
	S. pneumoniae	2.9 (2.4-3.3)	3.9 (3.3-4.6)	13.6 (12.8 - 14.4)	15.7 (14.4 - 17.2)	13.0 (12.5 - 13.4)	16.6 (15.9 - 17.4)
	Virus	36.5 (33.7-39.3)	12.8 (11.5-14.3)	26.0 (24.7 - 27.2)	9.0 (8.5 - 9.5)	32.6 (31.7 - 33.5)	11.1 (10.7 - 11.5)
Armenia	*E. coli*	6.6 (5.5-7.7)	13.0 (11.0-15.1)	6.3 (5.5 - 7.3)	12.3 (10.9 - 13.9)	4.9 (4.5 - 5.4)	10.6 (9.7 - 11.5)
	Group B *Streptococcus*	24.2 (22.4-26.2)	27.4 (25.1-29.5)	9.7 (8.9 - 10.4)	12.4 (11.5 - 13.3)	6.9 (6.6 - 7.2)	7.7 (7.3 - 8.0)
	H. influenzae	8.0 (6.7-9.5)	5.0 (4.1-6.0)	9.6 (8.9 - 10.4)	5.6 (5.1 - 6.1)	5.7 (5.4 - 6.0)	3.1 (2.9 - 3.2)
	K. pneumoniae	5.7 (4.5-7.0)	19.3 (16.0-23.0)	3.9 (3.2 - 4.7)	13.8 (11.8 - 16.2)	4.0 (3.5 - 4.5)	13.9 (12.6 - 15.1)
	L. monocytogenes	1.9 (1.3-2.6)	4.9 (3.5-6.6)	2.5 (1.9 - 3.1)	6.1 (4.8 - 7.6)	2.3 (2.1 - 2.5)	6.8 (6.3 - 7.4)
	N. meningitidis	8.0 (7.0-8.9)	6.9 (6.0-7.8)	12.5 (11.6 - 13.3)	10.0 (9.2 - 10.8)	13.5 (13.0 - 13.9)	10.2 (9.7 - 10.7)
	Other	2.0 (1.2-3.2)	2.6 (1.6-4.1)	10.6 (7.3 - 14.8)	11.5 (7.9 - 15.8)	10.5 (8.5 - 12.8)	13.0 (10.4 - 16.0)
	S. aureus	4.3 (3.5-5.2)	5.7 (4.7-6.9)	5.3 (4.8 - 5.9)	6.7 (6.0 - 7.4)	5.2 (5.0 - 5.5)	7.4 (7.0 - 7.9)
	S. pneumoniae	2.9 (2.4-3.3)	3.3 (2.8-3.9)	12.9 (12.0 - 13.9)	13.0 (12.0 - 14.1)	12.6 (12.1 - 13.0)	16.0 (15.3 - 16.7)
	Virus	36.5 (33.8-39.3)	11.9 (10.6-13.3)	26.7 (25.3 - 28.0)	8.6 (8.0 - 9.1)	34.6 (33.5 - 35.5)	11.3 (10.8 - 11.8)
Azerbaijan	*E. coli*	6.3 (5.2-7.6)	12.6 (10.4-14.8)	6.0 (5.1 - 6.9)	11.8 (10.5 - 13.3)	4.8 (4.2 - 5.3)	9.9 (9.0 - 10.9)
	Group B *Streptococcus*	21.7 (19.9-23.8)	24.8 (22.7-26.9)	9.3 (8.6 - 10.0)	14.3 (13.3 - 15.2)	6.8 (6.4 - 7.1)	8.8 (8.3 - 9.2)
	H. influenzae	8.8 (7.6-10.3)	5.7 (4.8-6.7)	11.2 (10.5 - 12.0)	6.5 (6.0 - 7.0)	6.9 (6.6 - 7.3)	4.1 (3.9 - 4.3)
	K. pneumoniae	5.6 (4.5-7.1)	18.9 (15.5-22.9)	3.8 (3.1 - 4.7)	14.5 (12.5 - 16.7)	3.9 (3.4 - 4.5)	14.1 (12.4 - 15.6)
	L. monocytogenes	1.9 (1.3-2.6)	4.9 (3.5-6.7)	2.3 (1.7 - 2.9)	5.6 (4.5 - 6.9)	2.1 (1.9 - 2.4)	5.9 (5.3 - 6.5)
	N. meningitidis	9.1 (8.1-10.1)	7.9 (6.9-8.9)	13.8 (12.9 - 14.7)	10.6 (9.8 - 11.3)	14.9 (14.4 - 15.5)	12.5 (11.9 - 13.1)
	Other	2.1 (1.3-3.3)	2.8 (1.7-4.3)	10.0 (7.0 - 14.0)	9.4 (6.8 - 12.7)	10.1 (8.3 - 12.2)	12.2 (10.2 - 14.6)
	S. aureus	4.4 (3.5-5.4)	5.9 (4.7-7.1)	5.2 (4.7 - 5.8)	6.5 (5.9 - 7.2)	5.1 (4.8 - 5.5)	7.1 (6.7 - 7.6)
	S. pneumoniae	3.0 (2.5-3.5)	3.6 (3.0-4.2)	12.2 (11.4 - 13.0)	10.9 (10.1 - 11.8)	11.9 (11.4 - 12.3)	13.7 (13.1 - 14.4)
	Virus	37.0 (34.3-39.9)	13.0 (11.7-14.5)	26.1 (24.8 - 27.4)	9.8 (9.3 - 10.4)	33.4 (32.4 - 34.3)	11.7 (11.3 - 12.2)
Georgia	*E. coli*	6.6 (5.4-7.7)	12.9 (10.8-15.0)	6.1 (5.2 - 7.0)	11.8 (10.4 - 13.2)	4.9 (4.4 - 5.4)	10.1 (9.2 - 11.1)
	Group B *Streptococcus*	23.3 (21.6-25.1)	26.4 (24.4-28.3)	9.4 (8.7 - 10.0)	12.2 (11.3 - 12.9)	6.7 (6.4 - 7.0)	7.6 (7.2 - 8.0)
	H. influenzae	8.3 (7.1-9.7)	5.3 (4.4-6.3)	10.4 (9.7 - 11.2)	6.0 (5.6 - 6.5)	6.2 (5.9 - 6.5)	3.4 (3.2 - 3.6)
	K. pneumoniae	5.5 (4.4-6.9)	18.7 (15.4-22.4)	3.9 (3.1 - 4.7)	13.5 (11.5 - 15.9)	4.0 (3.5 - 4.5)	13.6 (12.2 - 14.9)
	L. monocytogenes	1.9 (1.3-2.6)	4.8 (3.4-6.6)	2.3 (1.8 - 3.0)	5.8 (4.6 - 7.2)	2.3 (2.0 - 2.5)	6.4 (5.8 - 7.0)
	N. meningitidis	8.6 (7.8-9.6)	7.4 (6.5-8.4)	13.8 (12.9 - 14.6)	10.9 (10.1 - 11.7)	14.6 (14.1 - 15.1)	11.3 (10.8 - 11.8)
	Other	2.1 (1.3-3.2)	2.7 (1.7-4.1)	10.2 (7.1 - 14.2)	10.8 (7.6 - 14.8)	10.3 (8.4 - 12.3)	12.5 (10.2 - 15.1)
	S. aureus	4.3 (3.5-5.2)	5.7 (4.6-6.8)	5.1 (4.6 - 5.6)	6.4 (5.8 - 7.1)	5.1 (4.8 - 5.4)	7.1 (6.7 - 7.6)

Location	Aetiology	Neonatal		Under 5		All Ages	
		Nonfatal	Fatal	Nonfatal	Fatal	Nonfatal	Fatal
	S. pneumoniae	2.9 (2.5-3.4)	3.8 (3.2-4.4)	12.5 (11.7 - 13.3)	13.7 (12.8 - 14.7)	12.2 (11.8 - 12.6)	16.6 (15.9 - 17.3)
	Virus	36.6 (33.9-39.3)	12.4 (11.1-13.8)	26.3 (25.0 - 27.5)	8.8 (8.3 - 9.4)	33.7 (32.7 - 34.6)	11.4 (10.9 - 11.8)
Kazakhstan	*E. coli*	6.5 (5.4-7.7)	12.8 (10.7-15.1)	6.3 (5.4 - 7.2)	12.3 (10.8 - 14.0)	5.0 (4.5 - 5.6)	10.6 (9.7 - 11.6)
	Group B *Streptococcus*	23.9 (21.7-26.3)	27.0 (24.4-29.7)	10.0 (9.3 - 10.8)	12.3 (11.3 - 13.2)	7.4 (7.0 - 7.8)	8.8 (8.3 - 9.3)
	H. influenzae	8.1 (6.6-9.7)	5.1 (4.1-6.2)	9.1 (8.4 - 9.9)	5.4 (4.9 - 5.9)	5.8 (5.5 - 6.2)	3.5 (3.3 - 3.7)
	K. pneumoniae	5.8 (4.6-7.3)	19.7 (16.2-23.5)	3.9 (3.2 - 4.7)	13.7 (11.6 - 16.1)	3.9 (3.4 - 4.4)	14.1 (12.6 - 15.5)
	L. monocytogenes	1.9 (1.3-2.6)	4.9 (3.5-6.8)	2.4 (1.8 - 3.1)	6.2 (4.8 - 7.8)	2.3 (2.0 - 2.6)	6.5 (5.8 - 7.1)
	N. meningitidis	7.7 (6.7-8.8)	6.7 (5.7-7.7)	12.4 (11.5 - 13.2)	10.1 (9.3 - 11.0)	13.4 (12.9 - 13.9)	10.9 (10.3 - 11.4)
	Other	2.1 (1.3-3.2)	2.7 (1.7-4.2)	10.4 (7.1 - 14.5)	11.7 (8.1 - 16.0)	10.3 (8.3 - 12.7)	13.0 (10.6 - 15.7)
	S. aureus	4.4 (3.5-5.3)	5.8 (4.7-7.1)	5.4 (4.9 - 6.0)	7.0 (6.2 - 7.7)	5.3 (5.0 - 5.6)	7.5 (7.1 - 8.0)
	S. pneumoniae	2.9 (2.4-3.4)	3.0 (2.5-3.6)	13.4 (12.4 - 14.4)	12.6 (11.6 - 13.7)	13.0 (12.5 - 13.6)	14.3 (13.6 - 15.0)
	Virus	36.8 (33.9-39.7)	12.2 (10.8-13.7)	26.7 (25.3 - 28.0)	8.7 (8.1 - 9.3)	33.5 (32.4 - 34.4)	10.9 (10.5 - 11.4)
Kyrgyzstan	*E. coli*	6.6 (5.5-7.8)	12.9 (10.9-15.2)	6.1 (5.2 - 7.1)	11.7 (10.3 - 13.3)	4.9 (4.3 - 5.5)	9.8 (8.8 - 10.8)
	Group B *Streptococcus*	24.1 (22.1-26.2)	27.0 (24.7-29.3)	9.6 (8.8 - 10.3)	12.8 (11.9 - 13.7)	7.1 (6.7 - 7.5)	8.6 (8.1 - 9.0)
	H. influenzae	8.1 (6.8-9.6)	5.1 (4.2-6.2)	8.9 (8.3 - 9.6)	5.2 (4.8 - 5.7)	5.8 (5.5 - 6.1)	3.3 (3.1 - 3.5)
	K. pneumoniae	5.6 (4.4-6.9)	18.5 (15.2-22.2)	3.9 (3.1 - 4.7)	13.5 (11.5 - 15.8)	3.9 (3.4 - 4.4)	13.5 (12.0 - 15.1)
	L. monocytogenes	1.9 (1.3-2.6)	4.8 (3.4-6.6)	2.3 (1.8 - 3.0)	5.7 (4.5 - 7.1)	2.2 (1.9 - 2.5)	5.8 (5.1 - 6.4)
	N. meningitidis	8.1 (7.2-9.1)	7.0 (6.1-7.9)	14.4 (13.4 - 15.4)	11.0 (10.2 - 11.9)	15.6 (15.1 - 16.2)	12.7 (12.1 - 13.3)
	Other	2.1 (1.3-3.3)	2.7 (1.7-4.1)	10.1 (7.1 - 14.1)	10.4 (7.3 - 14.2)	10.1 (8.2 - 12.3)	12.1 (10.0 - 14.4)
	S. aureus	4.2 (3.4-5.2)	5.6 (4.5-6.8)	5.2 (4.7 - 5.8)	6.4 (5.8 - 7.1)	5.1 (4.7 - 5.4)	6.9 (6.4 - 7.3)
	S. pneumoniae	2.8 (2.4-3.3)	3.7 (3.2-4.3)	13.1 (12.2 - 14.1)	14.3 (13.2 - 15.4)	12.8 (12.3 - 13.3)	16.4 (15.7 - 17.1)
	Virus	36.5 (33.7-39.3)	12.7 (11.3-14.1)	26.3 (24.9 - 27.6)	9.1 (8.5 - 9.6)	32.5 (31.6 - 33.4)	11.0 (10.6 - 11.5)
Mongolia	*E. coli*	6.9 (5.6-8.4)	13.1 (10.8-15.7)	5.7 (4.7 - 6.7)	10.3 (8.8 - 11.8)	4.5 (3.9 - 5.2)	8.6 (7.5 - 9.8)
	Group B *Streptococcus*	26.0 (23.4-28.8)	28.7 (25.7-31.7)	9.0 (8.3 - 9.6)	10.7 (9.8 - 11.4)	6.5 (6.0 - 6.9)	7.0 (6.6 - 7.5)
	H. influenzae	7.6 (6.1-9.3)	4.8 (3.8-6.0)	6.4 (5.9 - 6.9)	3.7 (3.4 - 4.0)	4.0 (3.8 - 4.3)	2.2 (2.1 - 2.4)
	K. pneumoniae	5.5 (4.2-7.0)	17.5 (14.0-21.7)	3.7 (3.0 - 4.7)	11.8 (9.8 - 14.2)	3.8 (3.2 - 4.4)	12.2 (10.5 - 14.0)
	L. monocytogenes	1.8 (1.3-2.6)	4.5 (3.1-6.4)	2.2 (1.6 - 2.8)	5.1 (3.9 - 6.4)	2.0 (1.7 - 2.4)	5.2 (4.5 - 6.0)
	N. meningitidis	7.5 (6.5-8.7)	6.3 (5.3-7.4)	18.1 (17.1 - 19.0)	13.4 (12.5 - 14.3)	18.7 (18.0 - 19.3)	14.8 (14.0 - 15.4)
	Other	2.1 (1.3-3.3)	2.7 (1.6-4.1)	9.7 (6.8 - 13.4)	10.0 (7.0 - 13.6)	9.7 (8.0 - 11.7)	11.4 (9.6 - 13.5)
	S. aureus	4.0 (3.2-5.0)	5.2 (4.1-6.5)	4.9 (4.3 - 5.4)	5.7 (5.1 - 6.3)	4.8 (4.4 - 5.2)	6.3 (5.8 - 6.8)

Location	Aetiology	Neonatal		Under 5		All Ages	
		Nonfatal	Fatal	Nonfatal	Fatal	Nonfatal	Fatal
	S. pneumoniae	2.7 (2.2-3.2)	4.3 (3.5-5.1)	15.7 (14.8 - 16.6)	21.0 (19.6 - 22.4)	14.3 (13.7 - 14.8)	21.3 (20.3 - 22.2)
	Virus	35.9 (32.9-39.0)	12.9 (11.4-14.5)	24.8 (23.6 - 26.0)	8.3 (7.9 - 8.8)	31.7 (30.8 - 32.7)	10.9 (10.5 - 11.4)
Tajikistan	*E. coli*	6.8 (5.5-8.3)	13.0 (10.6-15.6)	5.4 (4.5 - 6.4)	10.0 (8.7 - 11.5)	4.6 (3.9 - 5.3)	8.8 (7.7 - 10.0)
	Group B *Streptococcus*	24.8 (22.3-27.5)	27.4 (24.6-30.2)	8.9 (8.2 - 9.5)	11.4 (10.6 - 12.2)	6.8 (6.3 - 7.2)	8.3 (7.7 - 8.8)
	H. influenzae	8.0 (6.6-9.6)	5.1 (4.1-6.3)	6.8 (6.3 - 7.3)	4.0 (3.7 - 4.4)	4.9 (4.5 - 5.2)	2.9 (2.7 - 3.1)
	K. pneumoniae	5.3 (4.1-6.8)	17.1 (13.5-21.2)	3.7 (2.9 - 4.6)	11.9 (9.9 - 14.2)	3.7 (3.1 - 4.4)	12.1 (10.4 - 13.9)
	L. monocytogenes	1.8 (1.3-2.5)	4.5 (3.2-6.3)	2.1 (1.6 - 2.7)	4.8 (3.7 - 6.0)	2.0 (1.7 - 2.4)	4.9 (4.2 - 5.7)
	N. meningitidis	8.2 (7.2-9.5)	7.0 (6.0-8.2)	19.4 (18.3 - 20.4)	13.8 (12.9 - 14.7)	19.7 (19.0 - 20.5)	15.1 (14.4 - 15.8)
	Other	2.1 (1.3-3.3)	2.7 (1.6-4.1)	9.2 (6.5 - 12.8)	9.0 (6.5 - 12.4)	9.4 (7.7 - 11.5)	10.5 (8.7 - 12.6)
	S. aureus	4.0 (3.2-5.1)	5.2 (4.1-6.6)	4.6 (4.1 - 5.1)	5.4 (4.9 - 6.0)	4.7 (4.3 - 5.1)	5.9 (5.4 - 6.4)
	S. pneumoniae	2.8 (2.3-3.3)	4.7 (3.9-5.6)	15.3 (14.4 - 16.2)	20.8 (19.4 - 22.2)	13.9 (13.3 - 14.5)	21.0 (20.1 - 22.1)
	Virus	36.1 (33.2-39.0)	13.3 (11.8-14.9)	24.5 (23.4 - 25.6)	8.7 (8.2 - 9.2)	30.3 (29.3 - 31.2)	10.4 (10.0 - 10.8)
Turkmenistan	*E. coli*	6.8 (5.6-8.1)	13.1 (11.0-15.3)	6.1 (5.2 - 7.1)	11.5 (10.2 - 13.0)	4.9 (4.4 - 5.5)	9.6 (8.7 - 10.6)
	Group B *Streptococcus*	24.7 (22.8-26.7)	27.5 (25.3-29.8)	9.5 (8.8 - 10.2)	13.5 (12.6 - 14.3)	7.0 (6.6 - 7.4)	8.9 (8.4 - 9.3)
	H. influenzae	7.9 (6.7-9.4)	5.0 (4.1-6.0)	8.4 (7.8 - 9.1)	4.9 (4.5 - 5.3)	5.7 (5.4 - 6.0)	3.3 (3.1 - 3.5)
	K. pneumoniae	5.4 (4.2-6.7)	17.7 (14.4-21.5)	3.9 (3.2 - 4.8)	13.4 (11.4 - 15.5)	3.9 (3.4 - 4.5)	13.3 (11.8 - 14.9)
	L. monocytogenes	1.8 (1.3-2.5)	4.7 (3.3-6.4)	2.3 (1.8 - 3.0)	5.4 (4.3 - 6.7)	2.1 (1.8 - 2.5)	5.4 (4.8 - 6.1)
	N. meningitidis	8.4 (7.5-9.4)	7.1 (6.2-8.1)	15.3 (14.3 - 16.3)	11.1 (10.3 - 11.9)	16.5 (16.0 - 17.1)	13.3 (12.7 - 13.9)
	Other	2.1 (1.2-3.2)	2.6 (1.6-4.0)	10.0 (7.0 - 13.9)	9.5 (6.8 - 13.0)	10.0 (8.2 - 12.2)	11.4 (9.5 - 13.7)
	S. aureus	4.1 (3.3-5.0)	5.3 (4.3-6.5)	5.1 (4.5 - 5.6)	6.0 (5.4 - 6.6)	4.9 (4.6 - 5.2)	6.3 (5.9 - 6.8)
	S. pneumoniae	2.8 (2.4-3.3)	4.4 (3.7-5.1)	12.9 (12.0 - 13.8)	15.6 (14.4 - 16.7)	12.4 (11.9 - 13.0)	17.5 (16.8 - 18.4)
	Virus	36.1 (33.4-38.8)	12.5 (11.2-13.9)	26.4 (25.1 - 27.7)	9.1 (8.6 - 9.7)	32.4 (31.4 - 33.3)	10.8 (10.4 - 11.2)
Uzbekistan	*E. coli*	6.6 (5.4-7.8)	12.8 (10.7-15.1)	6.1 (5.2 - 7.1)	11.8 (10.4 - 13.4)	4.8 (4.3 - 5.4)	9.4 (8.4 - 10.5)
	Group B *Streptococcus*	23.7 (21.7-26.0)	26.7 (24.3-29.1)	9.5 (8.8 - 10.3)	13.4 (12.4 - 14.3)	7.0 (6.6 - 7.4)	8.0 (7.6 - 8.5)
	H. influenzae	8.2 (6.8-9.7)	5.2 (4.2-6.3)	8.9 (8.2 - 9.6)	5.3 (4.8 - 5.7)	5.7 (5.4 - 6.0)	3.0 (2.8 - 3.2)
	K. pneumoniae	5.7 (4.5-7.0)	18.8 (15.5-22.7)	3.8 (3.1 - 4.7)	13.8 (11.7 - 16.0)	3.9 (3.3 - 4.4)	13.7 (12.0 - 15.4)
	L. monocytogenes	1.9 (1.3-2.6)	4.8 (3.4-6.7)	2.3 (1.8 - 3.0)	5.7 (4.5 - 7.1)	2.1 (1.8 - 2.5)	5.7 (5.1 - 6.4)
	N. meningitidis	8.1 (7.1-9.1)	6.9 (6.0-8.0)	14.2 (13.2 - 15.1)	10.8 (9.9 - 11.6)	15.4 (14.9 - 16.0)	13.4 (12.7 - 14.0)
	Other	2.1 (1.3-3.3)	2.7 (1.7-4.2)	10.2 (7.1 - 14.2)	10.2 (7.2 - 13.9)	10.1 (8.2 - 12.3)	12.5 (10.4 - 14.9)
	S. aureus	4.3 (3.4-5.3)	5.7 (4.5-7.0)	5.3 (4.7 - 5.9)	6.5 (5.9 - 7.2)	5.1 (4.8 - 5.5)	7.0 (6.6 - 7.5)

Location	Aetiology	Neonatal		Under 5		All Ages	
		Nonfatal	Fatal	Nonfatal	Fatal	Nonfatal	Fatal
	S. pneumoniae	2.9 (2.4-3.3)	3.5 (3.0-4.2)	13.3 (12.4 - 14.3)	13.3 (12.3 - 14.3)	12.9 (12.4 - 13.4)	15.5 (14.8 - 16.3)
	Virus	36.7 (33.8-39.5)	12.8 (11.4-14.3)	26.3 (24.8 - 27.5)	9.3 (8.7 - 9.9)	32.8 (31.8 - 33.7)	11.6 (11.1 - 12.1)
**Central Europe**	*E. coli*	6.9 (5.9-7.9)	13.5 (11.6-15.5)	6.5 (5.7 - 7.2)	12.7 (11.4 - 13.9)	5.4 (5.0 - 5.8)	11.4 (10.6 - 12.3)
	Group B *Streptococcus*	26.8 (25.1-28.6)	29.5 (27.5-31.5)	10.1 (9.4 - 10.8)	14.2 (13.4 - 15.0)	7.2 (6.9 - 7.5)	8.0 (7.6 - 8.4)
	H. influenzae	7.2 (5.9-8.7)	4.5 (3.6-5.4)	8.8 (8.1 - 9.4)	5.1 (4.7 - 5.6)	5.2 (4.9 - 5.4)	2.8 (2.6 - 3.0)
	K. pneumoniae	5.5 (4.4-6.9)	19.0 (15.6-22.7)	3.9 (3.2 - 4.7)	14.4 (12.5 - 16.5)	4.1 (3.7 - 4.5)	13.9 (12.8 - 15.0)
	L. monocytogenes	1.8 (1.3-2.5)	4.8 (3.5-6.5)	2.5 (1.9 - 3.2)	6.2 (5.0 - 7.7)	2.5 (2.3 - 2.8)	7.5 (6.9 - 8.0)
	N. meningitidis	7.4 (6.5-8.3)	6.4 (5.6-7.2)	12.0 (11.2 - 12.7)	9.0 (8.4 - 9.7)	12.1 (11.6 - 12.5)	8.4 (8.0 - 8.8)
	Other	1.9 (1.1-3.1)	2.5 (1.5-3.9)	10.7 (7.3 - 15.1)	10.8 (7.6 - 14.9)	10.7 (8.5 - 13.3)	12.7 (9.8 - 16.3)
	S. aureus	4.1 (3.4-5.0)	5.5 (4.5-6.6)	5.0 (4.6 - 5.5)	6.4 (5.8 - 7.0)	5.2 (4.9 - 5.4)	7.2 (6.9 - 7.6)
	S. pneumoniae	2.7 (2.3-3.2)	3.5 (3.0-4.1)	13.4 (12.6 - 14.2)	12.9 (12.0 - 13.9)	13.0 (12.6 - 13.5)	17.4 (16.6 - 18.2)
	Virus	35.5 (32.9-38.3)	10.9 (9.7-12.1)	27.1 (25.6 - 28.4)	8.3 (7.8 - 8.8)	34.5 (33.4 - 35.5)	10.5 (10.0 - 11.0)
Albania	*E. coli*	6.5 (5.3-7.7)	12.9 (10.8-15.2)	6.5 (5.7 - 7.4)	13.0 (11.6 - 14.6)	5.4 (5.0 - 5.9)	12.0 (11.1 - 13.0)
	Group B *Streptococcus*	23.9 (21.5-26.3)	27.2 (24.5-30.0)	10.5 (9.7 - 11.3)	13.0 (11.9 - 14.1)	7.7 (7.3 - 8.0)	8.9 (8.5 - 9.4)
	H. influenzae	8.0 (6.5-9.7)	4.9 (3.9-6.1)	9.9 (9.1 - 10.8)	5.8 (5.3 - 6.4)	6.2 (5.9 - 6.6)	3.7 (3.4 - 3.9)
	K. pneumoniae	5.9 (4.7-7.4)	20.5 (16.8-24.6)	3.9 (3.2 - 4.7)	14.4 (12.3 - 16.9)	4.1 (3.7 - 4.5)	14.2 (13.0 - 15.4)
	L. monocytogenes	1.9 (1.4-2.7)	5.0 (3.6-7.0)	2.5 (1.9 - 3.2)	6.6 (5.2 - 8.3)	2.5 (2.3 - 2.8)	7.5 (6.9 - 8.2)
	N. meningitidis	7.7 (6.6-8.8)	6.7 (5.7-7.7)	10.5 (9.7 - 11.3)	8.8 (8.0 - 9.6)	11.0 (10.5 - 11.5)	8.6 (8.2 - 9.1)
	Other	2.0 (1.2-3.2)	2.6 (1.6-4.2)	10.6 (7.2 - 14.9)	12.1 (8.3 - 17.0)	10.7 (8.5 - 13.2)	13.2 (10.2 - 16.6)
	S. aureus	4.5 (3.6-5.5)	6.0 (4.9-7.3)	5.5 (4.9 - 6.0)	7.2 (6.4 - 7.9)	5.5 (5.2 - 5.8)	7.8 (7.4 - 8.3)
	S. pneumoniae	2.9 (2.3-3.6)	2.6 (2.1-3.3)	12.7 (11.8 - 13.7)	10.6 (9.7 - 11.5)	12.8 (12.3 - 13.4)	13.0 (12.2 - 13.7)
	Virus	36.8 (33.9-39.7)	11.5 (10.1-12.8)	27.3 (25.8 - 28.7)	8.4 (7.8 - 9.0)	34.0 (32.9 - 35.0)	11.0 (10.5 - 11.5)
Bosnia and Herzegovina	*E. coli*	7.0 (6.0-8.1)	13.6 (11.8-15.6)	5.9 (5.2 - 6.7)	12.2 (11.1 - 13.4)	5.2 (4.7 - 5.6)	11.0 (10.1 - 11.8)
	Group B *Streptococcus*	26.4 (24.8-28.1)	29.5 (27.7-31.3)	9.5 (8.9 - 10.0)	18.6 (17.7 - 19.6)	6.8 (6.5 - 7.1)	8.5 (8.1 - 8.8)
	H. influenzae	7.4 (6.2-8.8)	4.5 (3.7-5.4)	10.2 (9.5 - 10.8)	5.1 (4.7 - 5.6)	5.7 (5.4 - 6.0)	3.0 (2.8 - 3.2)
	K. pneumoniae	5.3 (4.2-6.6)	18.0 (14.8-21.7)	3.8 (3.2 - 4.7)	15.0 (13.1 - 17.2)	4.1 (3.7 - 4.5)	13.6 (12.5 - 14.6)
	L. monocytogenes	1.8 (1.3-2.5)	4.7 (3.4-6.3)	2.3 (1.8 - 3.0)	5.3 (4.3 - 6.4)	2.4 (2.2 - 2.7)	6.9 (6.4 - 7.4)
	N. meningitidis	8.0 (7.1-8.9)	6.8 (5.9-7.6)	14.4 (13.5 - 15.1)	9.4 (8.8 - 10.0)	13.9 (13.4 - 14.3)	9.1 (8.7 - 9.5)
	Other	1.9 (1.1-3.0)	2.4 (1.5-3.9)	10.1 (7.1 - 13.9)	7.5 (5.5 - 10.1)	10.5 (8.5 - 12.8)	11.6 (9.0 - 14.7)
	S. aureus	4.0 (3.3-4.9)	5.3 (4.3-6.4)	4.6 (4.1 - 5.0)	5.5 (4.9 - 6.1)	4.9 (4.7 - 5.2)	6.6 (6.2 - 7.0)

Location	Aetiology	Neonatal		Under 5		All Ages	
		Nonfatal	Fatal	Nonfatal	Fatal	Nonfatal	Fatal
	S. pneumoniae	2.7 (2.3-3.2)	4.2 (3.6-5.0)	13.1 (12.3 - 13.9)	12.3 (11.5 - 13.2)	12.5 (12.0 - 12.9)	19.2 (18.3 - 20.1)
	Virus	35.4 (32.7-38.1)	11.1 (9.9-12.3)	26.1 (24.8 - 27.2)	9.1 (8.5 - 9.7)	34.0 (32.9 - 34.9)	10.5 (10.1 - 11.0)
Bulgaria	*E. coli*	6.4 (5.2-7.6)	12.8 (10.6-15.1)	6.3 (5.5 - 7.1)	12.7 (11.3 - 14.1)	5.3 (4.9 - 5.8)	11.4 (10.5 - 12.3)
	Group B *Streptococcus*	22.7 (20.5-25.0)	26.0 (23.4-28.6)	10.1 (9.3 - 10.8)	13.1 (12.1 - 14.1)	7.3 (7.0 - 7.6)	8.4 (8.0 - 8.8)
	H. influenzae	8.4 (7.0-10.0)	5.3 (4.3-6.4)	11.0 (10.1 - 11.9)	6.5 (5.9 - 7.1)	6.6 (6.2 - 6.9)	3.8 (3.5 - 4.0)
	K. pneumoniae	5.8 (4.6-7.3)	20.3 (16.6-24.3)	3.9 (3.2 - 4.7)	14.5 (12.4 - 16.8)	4.1 (3.7 - 4.5)	14.3 (13.1 - 15.6)
	L. monocytogenes	1.9 (1.4-2.7)	5.1 (3.6-7.0)	2.5 (1.9 - 3.1)	6.4 (5.0 - 7.9)	2.5 (2.3 - 2.8)	7.4 (6.8 - 8.0)
	N. meningitidis	8.3 (7.2-9.4)	7.2 (6.2-8.3)	11.1 (10.3 - 11.9)	9.3 (8.5 - 10.1)	11.5 (11.0 - 12.0)	9.0 (8.5 - 9.5)
	Other	2.1 (1.2-3.2)	2.7 (1.6-4.2)	10.4 (7.1 - 14.5)	11.6 (8.0 - 16.0)	10.6 (8.5 - 13.0)	13.2 (10.4 - 16.6)
	S. aureus	4.5 (3.6-5.5)	6.0 (4.9-7.3)	5.4 (4.8 - 5.9)	7.0 (6.3 - 7.8)	5.6 (5.3 - 5.8)	7.9 (7.5 - 8.4)
	S. pneumoniae	3.0 (2.4-3.6)	2.8 (2.3-3.4)	12.3 (11.5 - 13.3)	10.3 (9.5 - 11.2)	12.5 (12.0 - 13.1)	13.3 (12.6 - 14.0)
	Virus	37.0 (34.3-39.8)	11.9 (10.6-13.3)	27.0 (25.5 - 28.2)	8.7 (8.2 - 9.3)	34.1 (33.0 - 35.0)	11.3 (10.8 - 11.8)
Croatia	*E. coli*	7.2 (6.2-8.3)	14.1 (12.2-16.1)	6.4 (5.6 - 7.3)	12.3 (11.0 - 13.6)	5.6 (5.2 - 6.1)	11.9 (10.8 - 12.9)
	Group B *Streptococcus*	29.9 (27.9-32.0)	33.0 (30.7-35.3)	10.2 (9.5 - 10.9)	14.6 (13.8 - 15.4)	7.2 (6.8 - 7.6)	7.5 (7.1 - 8.0)
	H. influenzae	6.4 (5.0-8.0)	3.7 (2.9-4.7)	7.3 (6.6 - 8.0)	3.9 (3.5 - 4.2)	4.4 (4.1 - 4.7)	2.3 (2.1 - 2.5)
	K. pneumoniae	5.4 (4.2-6.7)	18.3 (14.7-22.2)	4.0 (3.2 - 4.9)	14.0 (12.1 - 16.2)	4.2 (3.9 - 4.6)	13.5 (12.3 - 14.7)
	L. monocytogenes	1.8 (1.3-2.4)	4.6 (3.3-6.3)	2.6 (1.9 - 3.3)	6.0 (4.8 - 7.5)	2.7 (2.5 - 3.0)	7.9 (7.2 - 8.5)
	N. meningitidis	6.7 (5.8-7.7)	5.6 (4.7-6.4)	12.4 (11.6 - 13.2)	9.0 (8.3 - 9.7)	11.6 (11.0 - 12.1)	7.4 (7.0 - 7.8)
	Other	1.8 (1.0-2.9)	2.3 (1.3-3.7)	10.6 (7.3 - 15.0)	10.2 (7.1 - 14.2)	10.9 (8.5 - 13.9)	12.3 (9.0 - 16.5)
	S. aureus	3.9 (3.1-4.8)	5.1 (4.0-6.2)	4.7 (4.2 - 5.2)	5.6 (5.0 - 6.2)	5.1 (4.8 - 5.4)	6.7 (6.3 - 7.2)
	S. pneumoniae	2.5 (2.1-3.1)	3.7 (3.0-4.5)	14.8 (13.7 - 15.7)	16.9 (15.6 - 18.2)	13.4 (12.8 - 14.0)	20.4 (19.1 - 21.5)
	Virus	34.4 (31.6-37.3)	9.7 (8.5-10.9)	27.0 (25.5 - 28.3)	7.4 (7.0 - 7.9)	34.8 (33.6 - 36.0)	9.9 (9.4 - 10.5)
Czechia	*E. coli*	7.2 (6.2-8.4)	14.1 (12.2-16.1)	6.4 (5.6 - 7.3)	12.3 (11.0 - 13.6)	5.5 (5.1 - 6.0)	11.5 (10.6 - 12.4)
	Group B *Streptococcus*	30.2 (28.1-32.4)	33.2 (30.9-35.6)	10.2 (9.5 - 10.9)	14.7 (13.9 - 15.6)	7.3 (7.0 - 7.7)	7.7 (7.3 - 8.1)
	H. influenzae	6.3 (4.9-8.0)	3.7 (2.8-4.7)	6.9 (6.3 - 7.6)	3.7 (3.3 - 4.0)	4.3 (4.0 - 4.7)	2.3 (2.1 - 2.5)
	K. pneumoniae	5.4 (4.2-6.7)	18.3 (14.7-22.1)	3.9 (3.2 - 4.8)	14.0 (12.0 - 16.1)	4.2 (3.8 - 4.6)	13.7 (12.6 - 14.7)
	L. monocytogenes	1.8 (1.3-2.4)	4.6 (3.3-6.3)	2.5 (1.9 - 3.3)	6.0 (4.7 - 7.4)	2.6 (2.4 - 2.9)	7.6 (7.0 - 8.2)
	N. meningitidis	6.6 (5.6-7.6)	5.5 (4.6-6.3)	12.6 (11.8 - 13.4)	9.0 (8.4 - 9.7)	12.0 (11.5 - 12.5)	7.8 (7.4 - 8.2)
	Other	1.8 (1.0-2.9)	2.3 (1.3-3.7)	10.6 (7.3 - 15.0)	10.1 (7.1 - 14.1)	10.9 (8.5 - 13.7)	12.4 (9.3 - 16.2)
	S. aureus	3.9 (3.1-4.8)	5.0 (4.0-6.2)	4.7 (4.2 - 5.2)	5.6 (5.0 - 6.2)	5.0 (4.7 - 5.3)	6.7 (6.3 - 7.1)

Location	Aetiology	Neonatal		Under 5		All Ages	
		Nonfatal	Fatal	Nonfatal	Fatal	Nonfatal	Fatal
	S. pneumoniae	2.5 (2.1-3.1)	3.7 (3.0-4.5)	15.1 (14.0 - 16.1)	17.2 (15.8 - 18.5)	13.6 (12.9 - 14.2)	20.4 (19.3 - 21.5)
	Virus	34.3 (31.5-37.2)	9.7 (8.6-10.9)	26.9 (25.4 - 28.2)	7.4 (7.0 - 7.9)	34.5 (33.3 - 35.7)	9.8 (9.3 - 10.3)
Hungary	*E. coli*	6.6 (5.5-7.9)	13.1 (10.9-15.5)	6.7 (5.8 - 7.5)	13.3 (11.9 - 14.9)	5.5 (5.1 - 6.0)	12.3 (11.3 - 13.4)
	Group B *Streptococcus*	26.2 (23.5-29.1)	29.5 (26.2-32.9)	10.9 (10.0 - 11.8)	14.2 (13.2 - 15.4)	7.7 (7.3 - 8.0)	8.6 (8.1 - 9.1)
	H. influenzae	7.3 (5.6-9.2)	4.4 (3.3-5.7)	8.3 (7.5 - 9.0)	4.8 (4.3 - 5.3)	4.6 (4.3 - 4.9)	2.7 (2.4 - 2.9)
	K. pneumoniae	6.0 (4.7-7.7)	20.9 (16.8-25.4)	3.9 (3.2 - 4.7)	14.7 (12.5 - 17.1)	4.1 (3.7 - 4.5)	14.1 (12.9 - 15.4)
	L. monocytogenes	1.9 (1.3-2.7)	4.9 (3.5-6.9)	2.6 (2.0 - 3.3)	6.7 (5.2 - 8.4)	2.7 (2.4 - 2.9)	8.2 (7.5 - 8.9)
	N. meningitidis	6.6 (5.5-7.7)	5.6 (4.6-6.7)	10.1 (9.3 - 10.9)	8.2 (7.5 - 9.0)	10.4 (9.9 - 10.9)	7.4 (7.0 - 7.9)
	Other	2.0 (1.2-3.2)	2.6 (1.5-4.0)	10.8 (7.3 - 15.4)	11.9 (8.2 - 16.8)	10.7 (8.4 - 13.4)	13.2 (9.9 - 17.3)
	S. aureus	4.4 (3.5-5.4)	5.8 (4.7-7.2)	5.5 (4.9 - 6.1)	7.2 (6.4 - 8.0)	5.6 (5.3 - 6.0)	8.1 (7.6 - 8.6)
	S. pneumoniae	2.7 (2.2-3.4)	2.3 (1.9-2.9)	14.0 (13.0 - 15.1)	10.6 (9.7 - 11.5)	14.3 (13.7 - 14.9)	14.5 (13.6 - 15.3)
	Virus	36.3 (33.1-39.4)	10.8 (9.5-12.2)	27.3 (25.6 - 28.7)	8.2 (7.7 - 8.8)	34.5 (33.2 - 35.6)	10.9 (10.2 - 11.4)
Montenegro	*E. coli*	7.1 (6.1-8.2)	13.9 (12.0-15.9)	6.2 (5.4 - 7.0)	11.9 (10.7 - 13.1)	5.2 (4.8 - 5.7)	10.9 (10.1 - 11.8)
	Group B *Streptococcus*	28.1 (26.4-30.0)	31.1 (29.2-33.1)	9.7 (9.1 - 10.3)	13.9 (13.2 - 14.7)	6.9 (6.6 - 7.2)	7.6 (7.2 - 7.9)
	H. influenzae	6.9 (5.6-8.4)	4.1 (3.3-5.1)	8.1 (7.4 - 8.7)	4.3 (3.9 - 4.7)	4.9 (4.6 - 5.2)	2.6 (2.4 - 2.8)
	K. pneumoniae	5.3 (4.2-6.7)	18.2 (14.7-22.1)	3.9 (3.2 - 4.8)	13.8 (11.9 - 15.9)	4.1 (3.7 - 4.5)	13.8 (12.7 - 14.7)
	L. monocytogenes	1.8 (1.3-2.5)	4.7 (3.4-6.3)	2.5 (1.9 - 3.2)	5.8 (4.6 - 7.2)	2.5 (2.2 - 2.7)	7.1 (6.6 - 7.7)
	N. meningitidis	7.4 (6.5-8.2)	6.2 (5.3-7.0)	13.5 (12.7 - 14.3)	9.8 (9.2 - 10.5)	13.0 (12.5 - 13.5)	8.8 (8.3 - 9.1)
	Other	1.8 (1.1-3.0)	2.4 (1.4-3.7)	10.5 (7.3 - 14.6)	10.1 (7.0 - 13.9)	10.7 (8.6 - 13.3)	12.5 (9.6 - 15.9)
	S. aureus	4.0 (3.2-4.9)	5.2 (4.2-6.3)	4.7 (4.2 - 5.1)	5.6 (5.1 - 6.1)	4.9 (4.7 - 5.2)	6.7 (6.3 - 7.1)
	S. pneumoniae	2.6 (2.2-3.1)	4.0 (3.3-4.7)	14.4 (13.4 - 15.2)	17.0 (15.8 - 18.2)	12.8 (12.3 - 13.3)	19.9 (18.9 - 20.8)
	Virus	35.0 (32.2-37.7)	10.4 (9.2-11.6)	26.6 (25.1 - 27.8)	7.8 (7.3 - 8.2)	34.8 (33.6 - 35.9)	10.1 (9.6 - 10.5)
North Macedonia	*E. coli*	7.0 (6.0-8.1)	13.7 (11.8-15.6)	6.1 (5.3 - 6.9)	11.6 (10.4 - 12.8)	5.1 (4.7 - 5.5)	10.8 (10.0 - 11.6)
	Group B *Streptococcus*	26.4 (24.9-28.1)	29.5 (27.7-31.2)	9.5 (8.9 - 10.1)	13.0 (12.3 - 13.7)	6.8 (6.5 - 7.1)	8.4 (8.0 - 8.7)
	H. influenzae	7.4 (6.2-8.8)	4.5 (3.7-5.4)	8.7 (8.0 - 9.3)	4.7 (4.3 - 5.1)	5.5 (5.2 - 5.8)	3.2 (3.1 - 3.4)
	K. pneumoniae	5.3 (4.2-6.6)	18.0 (14.8-21.8)	3.9 (3.2 - 4.7)	13.5 (11.6 - 15.7)	4.1 (3.7 - 4.5)	13.6 (12.4 - 14.6)
	L. monocytogenes	1.8 (1.3-2.5)	4.7 (3.4-6.3)	2.4 (1.8 - 3.1)	5.7 (4.5 - 7.0)	2.4 (2.2 - 2.6)	6.7 (6.1 - 7.2)
	N. meningitidis	8.0 (7.2-8.9)	6.8 (5.9-7.7)	14.3 (13.4 - 15.0)	10.6 (9.8 - 11.2)	13.9 (13.4 - 14.3)	9.7 (9.3 - 10.1)
	Other	1.9 (1.1-3.0)	2.4 (1.5-3.9)	10.2 (7.2 - 14.2)	10.1 (7.1 - 14.0)	10.5 (8.6 - 12.9)	11.9 (9.4 - 14.9)
	S. aureus	4.0 (3.3-4.9)	5.3 (4.3-6.4)	4.6 (4.2 - 5.1)	5.6 (5.1 - 6.2)	4.9 (4.7 - 5.1)	6.5 (6.2 - 6.8)

Location	Aetiology	Neonatal		Under 5		All Ages	
		Nonfatal	Fatal	Nonfatal	Fatal	Nonfatal	Fatal
	S. pneumoniae	2.7 (2.3-3.2)	4.2 (3.6-5.0)	13.9 (13.0 - 14.6)	17.2 (16.0 - 18.4)	12.4 (12.0 - 12.8)	19.2 (18.3 - 20.0)
	Virus	35.4 (32.7-38.0)	11.0 (9.8-12.2)	26.4 (25.1 - 27.6)	8.0 (7.5 - 8.4)	34.4 (33.4 - 35.3)	10.0 (9.6 - 10.4)
Poland	*E. coli*	7.0 (6.1-8.1)	13.8 (11.9-15.8)	6.6 (5.8 - 7.4)	12.6 (11.4 - 13.9)	5.4 (5.0 - 5.8)	11.1 (10.3 - 12.0)
	Group B *Streptococcus*	28.2 (26.3-30.1)	31.3 (29.2-33.4)	10.0 (9.3 - 10.7)	14.6 (13.8 - 15.4)	7.1 (6.8 - 7.4)	7.6 (7.2 - 7.9)
	H. influenzae	6.8 (5.5-8.5)	4.1 (3.2-5.1)	8.0 (7.3 - 8.7)	4.4 (4.0 - 4.7)	4.7 (4.4 - 5.0)	2.4 (2.3 - 2.6)
	K. pneumoniae	5.5 (4.3-6.8)	18.6 (15.1-22.5)	4.0 (3.2 - 4.8)	14.2 (12.3 - 16.3)	4.1 (3.7 - 4.5)	13.8 (12.6 - 14.8)
	L. monocytogenes	1.8 (1.3-2.5)	4.7 (3.4-6.4)	2.6 (2.0 - 3.3)	6.1 (4.9 - 7.6)	2.5 (2.3 - 2.8)	7.4 (6.8 - 7.9)
	N. meningitidis	7.0 (6.1-7.9)	5.9 (5.0-6.7)	12.1 (11.3 - 12.9)	9.0 (8.3 - 9.6)	12.4 (12.0 - 12.9)	8.5 (8.1 - 8.9)
	Other	1.9 (1.1-3.0)	2.4 (1.4-3.8)	10.8 (7.4 - 15.4)	10.4 (7.3 - 14.5)	10.8 (8.6 - 13.3)	12.6 (9.6 - 16.1)
	S. aureus	4.0 (3.3-4.9)	5.2 (4.3-6.4)	5.0 (4.6 - 5.5)	6.1 (5.5 - 6.7)	5.1 (4.9 - 5.4)	7.0 (6.6 - 7.4)
	S. pneumoniae	2.6 (2.2-3.1)	3.6 (3.0-4.2)	13.6 (12.7 - 14.5)	14.6 (13.5 - 15.7)	13.1 (12.6 - 13.6)	19.3 (18.3 - 20.1)
	Virus	35.1 (32.3-37.9)	10.5 (9.3-11.7)	27.2 (25.7 - 28.6)	8.0 (7.6 - 8.5)	34.7 (33.5 - 35.7)	10.3 (9.9 - 10.8)
Romania	*E. coli*	6.8 (5.8-7.8)	13.4 (11.6-15.4)	6.4 (5.6 - 7.2)	12.5 (11.3 - 13.7)	5.4 (5.0 - 5.8)	10.9 (10.1 - 11.8)
	Group B *Streptococcus*	24.9 (23.6-26.3)	28.1 (26.5-29.6)	9.8 (9.1 - 10.5)	14.4 (13.6 - 15.2)	7.1 (6.9 - 7.4)	8.0 (7.7 - 8.4)
	H. influenzae	7.8 (6.6-9.1)	4.8 (3.9-5.6)	10.9 (10.1 - 11.8)	5.9 (5.5 - 6.4)	6.7 (6.4 - 7.0)	3.5 (3.3 - 3.7)
	K. pneumoniae	5.4 (4.3-6.7)	18.6 (15.4-22.2)	4.0 (3.3 - 4.8)	14.5 (12.6 - 16.6)	4.2 (3.7 - 4.6)	14.2 (13.1 - 15.3)
	L. monocytogenes	1.9 (1.3-2.5)	4.8 (3.5-6.5)	2.5 (1.9 - 3.1)	6.0 (4.8 - 7.3)	2.5 (2.3 - 2.7)	7.0 (6.5 - 7.5)
	N. meningitidis	8.4 (7.6-9.2)	7.2 (6.4-8.0)	12.1 (11.3 - 12.9)	9.3 (8.6 - 10.0)	12.5 (12.1 - 12.9)	9.3 (8.9 - 9.7)
	Other	1.9 (1.1-3.1)	2.5 (1.5-3.9)	10.5 (7.2 - 14.8)	10.1 (7.2 - 13.9)	10.7 (8.6 - 13.1)	12.7 (10.0 - 15.8)
	S. aureus	4.2 (3.4-5.0)	5.5 (4.5-6.6)	5.0 (4.5 - 5.5)	6.2 (5.6 - 6.8)	5.1 (4.9 - 5.3)	7.0 (6.7 - 7.3)
	S. pneumoniae	2.8 (2.5-3.2)	3.9 (3.3-4.5)	11.6 (10.8 - 12.4)	12.5 (11.6 - 13.4)	11.7 (11.3 - 12.1)	16.8 (16.0 - 17.4)
	Virus	35.9 (33.4-38.6)	11.2 (10.0-12.4)	27.2 (25.8 - 28.5)	8.6 (8.1 - 9.1)	34.1 (33.0 - 35.0)	10.6 (10.2 - 11.1)
Serbia	*E. coli*	7.0 (6.0-8.1)	13.7 (11.9-15.7)	6.4 (5.6 - 7.2)	12.3 (11.1 - 13.5)	5.3 (4.9 - 5.8)	11.3 (10.4 - 12.2)
	Group B *Streptococcus*	27.3 (25.6-29.0)	30.3 (28.5-32.2)	9.8 (9.2 - 10.4)	14.4 (13.6 - 15.1)	7.0 (6.7 - 7.3)	7.6 (7.2 - 8.0)
	H. influenzae	7.1 (5.8-8.6)	4.3 (3.4-5.2)	8.9 (8.2 - 9.6)	4.8 (4.4 - 5.2)	5.3 (5.0 - 5.6)	2.8 (2.6 - 3.0)
	K. pneumoniae	5.4 (4.3-6.8)	18.3 (14.9-22.1)	3.9 (3.3 - 4.8)	14.1 (12.2 - 16.1)	4.1 (3.7 - 4.5)	13.5 (12.4 - 14.6)
	L. monocytogenes	1.8 (1.3-2.5)	4.7 (3.4-6.4)	2.5 (1.9 - 3.2)	5.9 (4.7 - 7.3)	2.5 (2.3 - 2.7)	7.3 (6.8 - 7.9)
	N. meningitidis	7.5 (6.7-8.4)	6.4 (5.5-7.2)	12.8 (12.1 - 13.6)	9.4 (8.8 - 10.1)	12.9 (12.4 - 13.3)	8.4 (8.0 - 8.8)
	Other	1.9 (1.1-3.0)	2.4 (1.5-3.8)	10.6 (7.3 - 14.9)	10.1 (7.1 - 13.8)	10.7 (8.6 - 13.2)	12.2 (9.3 - 15.7)
	S. aureus	4.0 (3.3-4.9)	5.3 (4.3-6.3)	4.9 (4.4 - 5.4)	5.9 (5.3 - 6.5)	5.0 (4.8 - 5.3)	6.9 (6.5 - 7.3)

Location	Aetiology	Neonatal		Under 5		All Ages	
		Nonfatal	Fatal	Nonfatal	Fatal	Nonfatal	Fatal
	S. pneumoniae	2.7 (2.3-3.2)	3.9 (3.3-4.6)	13.1 (12.3 - 13.9)	14.9 (13.8 - 15.9)	12.7 (12.2 - 13.1)	19.6 (18.6 - 20.5)
	Virus	35.3 (32.6-38.0)	10.7 (9.5-11.9)	27.0 (25.5 - 28.3)	8.2 (7.7 - 8.6)	34.5 (33.4 - 35.4)	10.3 (9.8 - 10.8)
Slovakia	*E. coli*	6.5 (5.3-8.0)	13.0 (10.6-15.6)	6.6 (5.8 - 7.5)	13.4 (11.9 - 15.0)	5.3 (4.9 - 5.8)	12.1 (11.1 - 13.0)
	Group B *Streptococcus*	25.7 (22.7-28.7)	29.0 (25.4-32.6)	10.9 (10.0 - 11.8)	13.5 (12.4 - 14.7)	7.7 (7.3 - 8.1)	9.3 (8.8 - 9.9)
	H. influenzae	7.4 (5.7-9.4)	4.5 (3.3-5.8)	8.5 (7.8 - 9.3)	5.0 (4.5 - 5.5)	4.8 (4.4 - 5.1)	3.0 (2.7 - 3.2)
	K. pneumoniae	6.1 (4.7-7.8)	21.3 (17.1-26.1)	3.9 (3.2 - 4.7)	14.5 (12.2 - 17.0)	4.0 (3.6 - 4.5)	14.6 (13.4 - 15.9)
	L. monocytogenes	1.9 (1.3-2.7)	5.0 (3.5-7.0)	2.6 (2.0 - 3.3)	6.9 (5.3 - 8.7)	2.5 (2.3 - 2.8)	7.8 (7.2 - 8.5)
	N. meningitidis	6.6 (5.5-7.7)	5.6 (4.6-6.8)	9.8 (9.1 - 10.6)	8.3 (7.5 - 9.1)	10.3 (9.8 - 10.8)	7.9 (7.4 - 8.4)
	Other	2.0 (1.2-3.2)	2.6 (1.5-4.1)	10.8 (7.3 - 15.4)	12.6 (8.7 - 17.8)	10.7 (8.5 - 13.4)	13.6 (10.4 - 17.4)
	S. aureus	4.5 (3.5-5.6)	5.9 (4.7-7.4)	5.6 (5.0 - 6.2)	7.4 (6.6 - 8.2)	5.6 (5.3 - 6.0)	8.2 (7.7 - 8.8)
	S. pneumoniae	2.8 (2.1-3.5)	2.2 (1.7-2.8)	14.1 (13.0 - 15.1)	10.2 (9.4 - 11.2)	14.1 (13.4 - 14.8)	12.8 (12.1 - 13.6)
	Virus	36.5 (33.3-39.6)	10.9 (9.6-12.4)	27.2 (25.5 - 28.6)	8.1 (7.6 - 8.7)	34.9 (33.6 - 36.0)	10.6 (10.0 - 11.1)
Slovenia	*E. coli*	7.1 (6.0-8.2)	13.9 (12.0-16.0)	6.8 (6.0 - 7.7)	13.4 (12.1 - 14.7)	5.7 (5.3 - 6.1)	12.3 (11.2 - 13.3)
	Group B *Streptococcus*	29.2 (27.3-31.3)	32.4 (30.2-34.7)	10.5 (9.7 - 11.3)	17.4 (16.4 - 18.4)	7.5 (7.1 - 7.8)	7.9 (7.4 - 8.3)
	H. influenzae	6.5 (5.1-8.2)	3.8 (2.9-4.8)	8.4 (7.6 - 9.1)	4.4 (3.9 - 4.8)	4.6 (4.3 - 4.9)	2.4 (2.2 - 2.6)
	K. pneumoniae	5.6 (4.3-6.9)	19.2 (15.5-23.1)	4.0 (3.3 - 4.8)	15.3 (13.3 - 17.5)	4.2 (3.8 - 4.6)	14.1 (12.9 - 15.3)
	L. monocytogenes	1.8 (1.3-2.5)	4.7 (3.4-6.4)	2.7 (2.0 - 3.5)	6.3 (5.0 - 7.8)	2.7 (2.4 - 3.0)	8.2 (7.6 - 8.9)
	N. meningitidis	6.6 (5.7-7.5)	5.5 (4.7-6.4)	10.4 (9.7 - 11.1)	7.6 (7.0 - 8.2)	10.5 (10.1 - 11.0)	7.1 (6.6 - 7.4)
	Other	1.8 (1.1-2.9)	2.3 (1.4-3.7)	11.1 (7.4 - 16.0)	9.9 (6.8 - 13.7)	11.1 (8.5 - 14.2)	12.9 (9.4 - 17.2)
	S. aureus	4.0 (3.3-5.0)	5.3 (4.3-6.5)	5.1 (4.5 - 5.6)	6.1 (5.5 - 6.8)	5.2 (4.9 - 5.5)	7.2 (6.7 - 7.7)
	S. pneumoniae	2.6 (2.1-3.1)	3.1 (2.6-3.7)	13.3 (12.3 - 14.2)	11.8 (10.9 - 12.7)	13.3 (12.7 - 13.8)	17.9 (16.8 - 18.8)
	Virus	34.8 (32.0-37.8)	9.7 (8.5-10.9)	27.7 (26.0 - 29.2)	7.9 (7.4 - 8.4)	35.3 (33.9 - 36.5)	10.1 (9.6 - 10.7)
**Eastern Europe**	*E. coli*	6.5 (5.4-7.7)	13.1 (11.0-15.3)	5.9 (5.2 - 6.7)	11.7 (10.4 - 13.1)	5.1 (4.7 - 5.5)	10.7 (9.8 - 11.6)
	Group B *Streptococcus*	22.2 (21.2-23.4)	25.6 (24.2-26.9)	8.9 (8.3 - 9.5)	11.0 (10.3 - 11.7)	6.6 (6.3 - 6.9)	7.4 (7.1 - 7.8)
	H. influenzae	8.6 (7.5-10.0)	5.4 (4.5-6.3)	16.9 (15.8 - 18.0)	9.2 (8.5 - 10.0)	9.2 (8.8 - 9.6)	4.7 (4.5 - 5.0)
	K. pneumoniae	5.3 (4.2-6.7)	18.5 (15.2-22.5)	3.8 (3.1 - 4.6)	13.5 (11.5 - 16.0)	4.1 (3.7 - 4.5)	14.3 (13.1 - 15.5)
	L. monocytogenes	1.9 (1.4-2.5)	5.0 (3.6-6.6)	2.3 (1.8 - 2.9)	6.0 (4.7 - 7.4)	2.4 (2.1 - 2.6)	6.8 (6.3 - 7.4)
	N. meningitidis	9.9 (9.0-10.8)	8.5 (7.6-9.4)	11.8 (11.1 - 12.5)	10.1 (9.4 - 10.8)	12.5 (12.1 - 12.9)	9.9 (9.4 - 10.3)
	Other	1.9 (1.2-3.0)	2.6 (1.5-4.0)	10.0 (6.9 - 14.1)	11.4 (7.9 - 15.7)	10.5 (8.5 - 12.9)	13.1 (10.5 - 16.1)
	S. aureus	4.3 (3.5-5.1)	5.7 (4.7-6.8)	4.7 (4.2 - 5.1)	6.1 (5.5 - 6.6)	5.0 (4.8 - 5.2)	7.0 (6.7 - 7.4)

Location	Aetiology	Neonatal		Under 5		All Ages	
		Nonfatal	Fatal	Nonfatal	Fatal	Nonfatal	Fatal
	S. pneumoniae	3.0 (2.6-3.5)	4.1 (3.5-4.7)	9.9 (9.2 - 10.4)	12.9 (12.0 - 13.8)	10.4 (10.0 - 10.7)	15.1 (14.4 - 15.7)
	Virus	36.3 (33.9-38.7)	11.6 (10.4-12.9)	25.9 (24.5 - 27.1)	8.1 (7.6 - 8.6)	34.4 (33.3 - 35.3)	11.0 (10.5 - 11.4)
Belarus	*E. coli*	6.6 (5.4-7.9)	13.3 (11.1-15.7)	5.6 (4.9 - 6.4)	11.4 (10.0 - 12.8)	5.1 (4.6 - 5.5)	10.8 (10.0 - 11.8)
	Group B *Streptococcus*	22.3 (21.4-23.3)	25.5 (24.3-26.6)	8.5 (8.1 - 9.0)	10.8 (10.1 - 11.5)	6.4 (6.1 - 6.6)	7.5 (7.1 - 7.9)
	H. influenzae	8.6 (7.4-10.0)	5.3 (4.4-6.3)	20.0 (18.9 - 21.0)	11.3 (10.6 - 12.1)	10.4 (10.0 - 10.9)	5.8 (5.6 - 6.1)
	K. pneumoniae	5.1 (4.0-6.6)	18.0 (14.7-22.2)	3.7 (3.0 - 4.5)	13.4 (11.3 - 16.0)	4.1 (3.7 - 4.6)	14.4 (13.1 - 15.7)
	L. monocytogenes	1.9 (1.3-2.5)	5.0 (3.6-6.6)	2.2 (1.7 - 2.8)	5.8 (4.6 - 7.3)	2.4 (2.1 - 2.6)	6.9 (6.3 - 7.5)
	N. meningitidis	10.5 (9.6-11.4)	9.1 (8.2-10.0)	12.0 (11.3 - 12.6)	10.1 (9.5 - 10.8)	12.2 (11.8 - 12.6)	9.3 (8.9 - 9.7)
	Other	1.9 (1.1-2.9)	2.5 (1.4-3.9)	9.6 (6.7 - 13.6)	11.1 (7.7 - 15.5)	10.5 (8.5 - 13.1)	13.0 (10.2 - 16.3)
	S. aureus	4.2 (3.4-5.1)	5.6 (4.5-6.9)	4.2 (3.8 - 4.6)	5.6 (5.0 - 6.1)	4.7 (4.5 - 5.0)	6.6 (6.3 - 7.0)
	S. pneumoniae	3.0 (2.6-3.5)	4.6 (3.9-5.4)	9.0 (8.5 - 9.5)	12.8 (11.8 - 13.6)	9.5 (9.1 - 9.8)	15.2 (14.5 - 15.9)
	Virus	35.9 (33.5-38.4)	11.1 (10.0-12.3)	25.1 (23.9 - 26.2)	7.7 (7.2 - 8.1)	34.6 (33.5 - 35.7)	10.3 (9.8 - 10.8)
Estonia	*E. coli*	7.2 (6.2-8.4)	14.0 (12.1-16.1)	6.3 (5.5 - 7.2)	11.8 (10.4 - 13.2)	5.4 (5.0 - 5.8)	11.3 (10.3 - 12.2)
	Group B *Streptococcus*	30.0 (27.9-32.2)	33.0 (30.6-35.4)	10.0 (9.3 - 10.7)	11.9 (11.1 - 12.8)	7.0 (6.7 - 7.4)	7.4 (6.9 - 7.8)
	H. influenzae	6.4 (5.0-8.1)	3.8 (2.9-4.8)	6.5 (5.8 - 7.1)	3.5 (3.1 - 3.8)	4.0 (3.7 - 4.3)	2.2 (2.0 - 2.4)
	K. pneumoniae	5.4 (4.2-6.7)	18.2 (14.5-22.0)	3.9 (3.2 - 4.8)	13.1 (11.1 - 15.6)	4.2 (3.8 - 4.5)	13.4 (12.3 - 14.5)
	L. monocytogenes	1.8 (1.3-2.4)	4.6 (3.3-6.2)	2.5 (1.9 - 3.2)	6.0 (4.7 - 7.6)	2.6 (2.3 - 2.8)	7.4 (6.8 - 8.0)
	N. meningitidis	6.6 (5.6-7.6)	5.5 (4.6-6.4)	13.2 (12.3 - 14.0)	9.9 (9.1 - 10.6)	12.5 (12.0 - 13.0)	8.3 (7.9 - 8.7)
	Other	1.8 (1.1-2.9)	2.3 (1.4-3.7)	10.6 (7.3 - 14.8)	11.1 (7.7 - 15.6)	10.8 (8.5 - 13.6)	12.4 (9.3 - 16.0)
	S. aureus	3.9 (3.1-4.8)	5.0 (4.0-6.2)	4.7 (4.2 - 5.2)	5.6 (5.1 - 6.2)	5.0 (4.7 - 5.3)	6.7 (6.3 - 7.1)
	S. pneumoniae	2.5 (2.1-3.1)	3.7 (3.1-4.5)	15.6 (14.5 - 16.6)	19.8 (18.3 - 21.3)	13.8 (13.1 - 14.4)	21.0 (19.8 - 22.1)
	Virus	34.4 (31.6-37.3)	10.0 (8.8-11.2)	26.6 (25.2 - 27.9)	7.2 (6.7 - 7.6)	34.7 (33.5 - 35.9)	10.0 (9.5 - 10.4)
Latvia	*E. coli*	6.7 (5.5-8.0)	13.1 (11.0-15.5)	6.6 (5.7 - 7.4)	13.0 (11.5 - 14.5)	5.4 (5.0 - 5.9)	11.5 (10.6 - 12.5)
	Group B *Streptococcus*	26.3 (23.7-29.1)	29.5 (26.4-32.9)	10.7 (9.9 - 11.6)	14.0 (13.0 - 15.1)	7.7 (7.4 - 8.1)	8.6 (8.1 - 9.0)
	H. influenzae	7.3 (5.6-9.1)	4.4 (3.3-5.7)	7.7 (7.0 - 8.4)	4.5 (4.1 - 5.0)	4.5 (4.2 - 4.8)	2.5 (2.3 - 2.7)
	K. pneumoniae	6.0 (4.6-7.5)	20.4 (16.5-24.8)	3.9 (3.2 - 4.7)	14.4 (12.3 - 16.8)	4.0 (3.6 - 4.5)	14.1 (12.9 - 15.3)
	L. monocytogenes	1.9 (1.3-2.6)	4.9 (3.4-6.8)	2.6 (1.9 - 3.2)	6.5 (5.1 - 8.1)	2.6 (2.3 - 2.8)	7.6 (6.9 - 8.2)
	N. meningitidis	6.6 (5.5-7.7)	5.6 (4.7-6.6)	11.0 (10.1 - 11.8)	8.8 (8.0 - 9.6)	11.3 (10.8 - 11.8)	8.5 (8.0 - 8.9)
	Other	2.0 (1.2-3.2)	2.6 (1.6-4.1)	10.7 (7.3 - 15.1)	11.6 (8.0 - 16.2)	10.6 (8.4 - 13.2)	13.1 (10.1 - 16.6)
	S. aureus	4.4 (3.5-5.4)	5.7 (4.6-7.1)	5.5 (4.9 - 6.1)	7.1 (6.3 - 7.8)	5.6 (5.3 - 5.9)	7.9 (7.5 - 8.5)

Location	Aetiology	Neonatal		Under 5		All Ages	
		Nonfatal	Fatal	Nonfatal	Fatal	Nonfatal	Fatal
	S. pneumoniae	2.7 (2.2-3.3)	2.5 (2.0-3.1)	14.4 (13.4 - 15.5)	11.6 (10.7 - 12.6)	14.6 (14.0 - 15.2)	15.3 (14.5 - 16.1)
	Virus	36.2 (33.1-39.4)	11.2 (9.8-12.6)	27.0 (25.4 - 28.4)	8.4 (7.8 - 9.0)	33.6 (32.5 - 34.6)	10.9 (10.3 - 11.3)
Lithuania	*E. coli*	6.8 (5.8-8.0)	13.4 (11.3-15.6)	6.6 (5.7 - 7.4)	12.6 (11.1 - 14.2)	5.4 (5.0 - 5.9)	11.2 (10.3 - 12.1)
	Group B *Streptococcus*	27.2 (25.0-29.7)	30.3 (27.6-33.0)	10.4 (9.6 - 11.2)	12.4 (11.4 - 13.4)	7.4 (7.1 - 7.7)	7.9 (7.5 - 8.3)
	H. influenzae	7.1 (5.5-8.9)	4.3 (3.3-5.4)	7.5 (6.9 - 8.2)	4.3 (3.9 - 4.8)	4.4 (4.1 - 4.7)	2.4 (2.2 - 2.6)
	K. pneumoniae	5.7 (4.5-7.2)	19.5 (15.7-23.4)	3.9 (3.2 - 4.7)	13.6 (11.6 - 16.0)	4.1 (3.7 - 4.5)	13.7 (12.5 - 14.8)
	L. monocytogenes	1.8 (1.3-2.5)	4.8 (3.4-6.5)	2.5 (1.9 - 3.2)	6.4 (5.0 - 8.0)	2.6 (2.3 - 2.8)	7.3 (6.8 - 7.9)
	N. meningitidis	6.7 (5.7-7.7)	5.7 (4.8-6.6)	11.8 (10.9 - 12.7)	9.5 (8.7 - 10.3)	12.2 (11.7 - 12.6)	8.8 (8.4 - 9.2)
	Other	2.0 (1.2-3.1)	2.5 (1.5-4.0)	10.7 (7.3 - 15.1)	11.9 (8.2 - 16.8)	10.6 (8.5 - 13.1)	12.7 (9.9 - 16.1)
	S. aureus	4.2 (3.4-5.1)	5.5 (4.5-6.7)	5.4 (4.8 - 5.9)	6.8 (6.0 - 7.5)	5.4 (5.1 - 5.7)	7.5 (7.0 - 7.9)
	S. pneumoniae	2.7 (2.2-3.2)	3.0 (2.5-3.6)	14.2 (13.1 - 15.2)	14.4 (13.3 - 15.6)	14.3 (13.8 - 14.7)	17.8 (16.9 - 18.6)
	Virus	35.7 (32.8-38.7)	11.1 (9.8-12.4)	27.0 (25.5 - 28.4)	8.1 (7.5 - 8.6)	33.8 (32.7 - 34.7)	10.7 (10.1 - 11.1)
Republic of Moldova	*E. coli*	6.6 (5.5-7.7)	13.1 (11.0-15.1)	6.3 (5.4 - 7.2)	12.2 (10.9 - 13.6)	5.1 (4.6 - 5.6)	10.3 (9.5 - 11.2)
	Group B *Streptococcus*	24.1 (22.5-25.9)	27.2 (25.2-29.1)	9.8 (9.1 - 10.5)	14.0 (13.2 - 14.9)	7.0 (6.7 - 7.2)	8.1 (7.8 - 8.5)
	H. influenzae	8.0 (6.8-9.4)	5.0 (4.2-6.0)	9.8 (9.1 - 10.6)	5.6 (5.2 - 6.1)	5.9 (5.7 - 6.2)	3.3 (3.1 - 3.5)
	K. pneumoniae	5.6 (4.4-6.9)	18.8 (15.6-22.5)	3.9 (3.2 - 4.7)	14.3 (12.3 - 16.4)	4.1 (3.6 - 4.5)	14.0 (12.7 - 15.3)
	L. monocytogenes	1.9 (1.3-2.6)	4.8 (3.5-6.6)	2.4 (1.8 - 3.0)	5.8 (4.7 - 7.1)	2.4 (2.1 - 2.6)	6.5 (6.0 - 7.1)
	N. meningitidis	8.3 (7.4-9.2)	7.1 (6.3-8.0)	13.0 (12.2 - 13.9)	10.0 (9.2 - 10.7)	13.7 (13.3 - 14.2)	10.5 (10.0 - 10.9)
	Other	2.0 (1.2-3.2)	2.6 (1.6-4.1)	10.3 (7.2 - 14.3)	10.1 (7.2 - 13.8)	10.4 (8.6 - 12.6)	12.6 (10.2 - 15.4)
	S. aureus	4.2 (3.5-5.1)	5.6 (4.6-6.8)	5.2 (4.7 - 5.7)	6.4 (5.8 - 7.0)	5.2 (5.0 - 5.5)	7.2 (6.8 - 7.6)
	S. pneumoniae	2.9 (2.5-3.3)	3.7 (3.1-4.2)	12.4 (11.6 - 13.3)	12.6 (11.7 - 13.6)	12.5 (12.1 - 12.9)	16.6 (15.8 - 17.2)
	Virus	36.4 (33.7-39.1)	12.0 (10.7-13.4)	26.8 (25.5 - 28.1)	9.0 (8.5 - 9.6)	33.8 (32.8 - 34.7)	11.0 (10.5 - 11.4)
Russian Federation	*E. coli*	6.4 (5.3-7.7)	12.9 (10.8-15.3)	5.9 (5.1 - 6.8)	12.0 (10.6 - 13.5)	5.1 (4.7 - 5.6)	10.9 (10.0 - 11.8)
	Group B *Streptococcus*	21.3 (20.1-22.4)	24.5 (23.1-25.9)	8.8 (8.2 - 9.4)	11.2 (10.4 - 12.0)	6.6 (6.3 - 6.9)	7.5 (7.1 - 7.8)
	H. influenzae	8.9 (7.7-10.3)	5.6 (4.7-6.6)	18.6 (17.3 - 20.0)	10.8 (9.9 - 11.7)	10.2 (9.7 - 10.7)	5.2 (4.9 - 5.5)
	K. pneumoniae	5.4 (4.2-6.8)	18.8 (15.4-22.9)	3.7 (3.1 - 4.6)	13.7 (11.7 - 16.4)	4.1 (3.7 - 4.5)	14.5 (13.2 - 15.8)
	L. monocytogenes	1.9 (1.4-2.6)	5.0 (3.6-6.7)	2.3 (1.8 - 2.9)	6.1 (4.8 - 7.6)	2.4 (2.1 - 2.6)	7.0 (6.4 - 7.6)
	N. meningitidis	10.3 (9.4-11.3)	9.0 (8.0-10.0)	11.1 (10.3 - 11.8)	9.5 (8.8 - 10.3)	12.0 (11.5 - 12.4)	9.5 (9.0 - 9.9)
	Other	2.0 (1.2-3.1)	2.6 (1.5-4.0)	10.0 (6.9 - 14.0)	11.6 (8.1 - 16.0)	10.5 (8.5 - 12.9)	13.3 (10.5 - 16.3)
	S. aureus	4.3 (3.5-5.2)	5.9 (4.8-7.1)	4.7 (4.3 - 5.2)	6.3 (5.6 - 6.9)	5.0 (4.8 - 5.3)	7.2 (6.8 - 7.6)

Location	Aetiology	Neonatal		Under 5		All Ages	
		Nonfatal	Fatal	Nonfatal	Fatal	Nonfatal	Fatal
	S. pneumoniae	3.1 (2.6-3.6)	4.0 (3.4-4.6)	9.0 (8.3 - 9.7)	10.6 (9.7 - 11.6)	9.9 (9.5 - 10.2)	13.9 (13.2 - 14.6)
	Virus	36.5 (34.1-38.9)	11.7 (10.5-13.0)	25.8 (24.4 - 27.0)	8.2 (7.7 - 8.7)	34.3 (33.2 - 35.2)	11.1 (10.6 - 11.6)
Ukraine	*E. coli*	6.9 (5.8-8.0)	13.5 (11.5-15.5)	5.9 (5.1 - 6.7)	11.2 (9.8 - 12.5)	5.0 (4.6 - 5.4)	9.9 (9.2 - 10.8)
	Group B *Streptococcus*	24.9 (23.6-26.4)	28.0 (26.5-29.5)	9.1 (8.6 - 9.7)	10.6 (9.9 - 11.3)	6.5 (6.2 - 6.7)	7.2 (6.9 - 7.6)
	H. influenzae	7.8 (6.7-9.0)	4.8 (4.0-5.6)	10.8 (10.1 - 11.5)	6.0 (5.6 - 6.4)	6.3 (6.0 - 6.6)	3.5 (3.3 - 3.7)
	K. pneumoniae	5.3 (4.2-6.6)	17.9 (14.7-21.6)	3.8 (3.2 - 4.7)	12.9 (10.9 - 15.3)	4.1 (3.7 - 4.5)	13.9 (12.7 - 15.1)
	L. monocytogenes	1.8 (1.3-2.5)	4.8 (3.5-6.3)	2.3 (1.8 - 3.0)	5.7 (4.5 - 7.2)	2.3 (2.1 - 2.6)	6.3 (5.7 - 6.8)
	N. meningitidis	8.8 (7.9-9.6)	7.5 (6.6-8.3)	14.5 (13.7 - 15.2)	11.4 (10.6 - 12.1)	14.2 (13.7 - 14.6)	11.2 (10.7 - 11.7)
	Other	1.9 (1.1-3.0)	2.5 (1.5-3.9)	10.1 (7.1 - 14.0)	11.1 (7.8 - 15.1)	10.5 (8.6 - 12.7)	12.8 (10.4 - 15.6)
	S. aureus	4.1 (3.3-4.9)	5.4 (4.4-6.5)	4.6 (4.1 - 5.0)	5.7 (5.1 - 6.2)	4.9 (4.6 - 5.1)	6.5 (6.2 - 6.9)
	S. pneumoniae	2.8 (2.4-3.3)	4.4 (3.8-5.2)	12.7 (12.0 - 13.4)	17.7 (16.5 - 18.9)	11.6 (11.2 - 12.0)	18.1 (17.3 - 18.8)
	Virus	35.7 (33.2-38.2)	11.4 (10.2-12.6)	26.1 (24.8 - 27.2)	7.7 (7.3 - 8.2)	34.6 (33.6 - 35.5)	10.5 (10.1 - 11.0)
**High-income**	*E. coli*	6.6 (5.4-8.0)	13.1 (10.7-15.7)	6.8 (6.0 - 7.6)	13.5 (12.0 - 15.0)	5.6 (5.2 - 6.1)	12.8 (11.7 - 13.9)
	Group B *Streptococcus*	26.0 (23.3-28.8)	28.9 (25.5-32.6)	11.0 (10.1 - 11.9)	14.5 (13.3 - 15.7)	7.7 (7.2 - 8.0)	8.9 (8.4 - 9.4)
	H. influenzae	7.3 (5.7-9.2)	4.4 (3.3-5.8)	9.5 (8.6 - 10.3)	5.2 (4.7 - 5.8)	5.4 (5.1 - 5.8)	3.0 (2.7 - 3.2)
	K. pneumoniae	6.0 (4.7-7.7)	21.5 (17.3-26.3)	3.9 (3.2 - 4.7)	15.0 (12.8 - 17.3)	4.1 (3.8 - 4.6)	14.5 (13.1 - 15.8)
	L. monocytogenes	1.9 (1.3-2.7)	5.0 (3.6-6.9)	2.7 (2.0 - 3.4)	6.8 (5.3 - 8.6)	2.7 (2.4 - 3.0)	8.4 (7.7 - 9.1)
	N. meningitidis	6.8 (5.7-7.9)	5.7 (4.7-6.9)	9.2 (8.5 - 9.9)	7.9 (7.2 - 8.7)	9.3 (8.9 - 9.8)	7.2 (6.8 - 7.6)
	Other	1.9 (1.1-3.1)	2.6 (1.5-4.1)	10.9 (7.3 - 15.8)	12.0 (8.3 - 17.0)	11.0 (8.5 - 14.1)	13.5 (10.0 - 17.9)
	S. aureus	4.4 (3.5-5.5)	6.0 (4.7-7.5)	5.5 (4.9 - 6.1)	7.2 (6.5 - 8.0)	5.6 (5.3 - 5.9)	8.2 (7.6 - 8.7)
	S. pneumoniae	2.8 (2.2-3.5)	2.2 (1.7-2.7)	13.1 (12.1 - 14.1)	9.6 (8.8 - 10.5)	13.1 (12.5 - 13.7)	12.8 (12.0 - 13.6)
	Virus	36.2 (33.2-39.4)	10.6 (9.3-12.0)	27.6 (25.8 - 29.1)	8.2 (7.6 - 8.7)	35.5 (34.1 - 36.7)	10.8 (10.2 - 11.4)
**Australasia**	*E. coli*	6.4 (5.0-8.1)	13.0 (10.3-16.0)	6.8 (6.0 - 7.8)	14.0 (12.4 - 15.8)	5.6 (5.1 - 6.1)	13.2 (12.1 - 14.4)
	Group B *Streptococcus*	25.4 (21.8-29.1)	28.9 (24.8-33.3)	11.4 (10.4 - 12.5)	16.0 (14.5 - 17.5)	8.4 (7.8 - 9.0)	10.4 (9.7 - 11.1)
	H. influenzae	7.4 (5.5-9.6)	4.4 (3.2-5.8)	9.3 (8.4 - 10.3)	5.3 (4.7 - 5.9)	5.5 (5.0 - 5.9)	3.2 (2.9 - 3.5)
	K. pneumoniae	6.3 (4.8-8.3)	22.5 (17.8-27.7)	3.9 (3.2 - 4.7)	16.0 (13.6 - 18.4)	4.0 (3.6 - 4.5)	15.3 (13.9 - 16.7)
	L. monocytogenes	1.9 (1.3-2.7)	5.1 (3.6-7.2)	2.7 (2.0 - 3.5)	7.1 (5.5 - 8.9)	2.6 (2.3 - 3.0)	8.5 (7.7 - 9.4)
	N. meningitidis	6.4 (5.2-7.8)	5.5 (4.4-6.8)	8.0 (7.3 - 8.7)	6.8 (6.2 - 7.5)	8.5 (7.9 - 9.0)	6.7 (6.2 - 7.2)
	Other	2.0 (1.1-3.2)	2.6 (1.4-4.1)	11.0 (7.4 - 15.9)	11.9 (8.1 - 17.1)	11.0 (8.4 - 14.4)	13.9 (10.2 - 18.4)
	S. aureus	4.6 (3.5-5.9)	6.2 (4.8-7.9)	5.7 (5.0 - 6.4)	7.5 (6.7 - 8.4)	5.7 (5.3 - 6.1)	8.5 (7.8 - 9.1)

Location	Aetiology	Neonatal		Under 5		All Ages	
		Nonfatal	Fatal	Nonfatal	Fatal	Nonfatal	Fatal
	S. pneumoniae	2.8 (2.0-3.8)	1.8 (1.3-2.3)	13.6 (12.5 - 14.8)	7.3 (6.6 - 8.1)	13.6 (12.8 - 14.5)	9.9 (9.1 - 10.6)
	Virus	36.6 (33.3-40.2)	10.2 (8.9-11.6)	27.6 (25.7 - 29.1)	8.1 (7.5 - 8.7)	35.2 (33.6 - 36.5)	10.5 (9.9 - 11.1)
Australia	*E. coli*	6.4 (5.0-8.1)	12.9 (10.1-16.0)	6.8 (6.0 - 7.8)	14.1 (12.4 - 15.9)	5.6 (5.1 - 6.2)	13.3 (12.1 - 14.5)
	Group B *Streptococcus*	25.3 (21.6-29.1)	28.7 (24.4-33.4)	11.5 (10.4 - 12.5)	16.1 (14.6 - 17.6)	8.4 (7.8 - 9.0)	10.4 (9.7 - 11.2)
	H. influenzae	7.4 (5.5-9.7)	4.4 (3.2-5.9)	9.3 (8.4 - 10.3)	5.3 (4.7 - 5.9)	5.5 (5.0 - 6.0)	3.2 (2.9 - 3.5)
	K. pneumoniae	6.4 (4.8-8.3)	22.7 (17.9-28.2)	3.9 (3.2 - 4.7)	16.1 (13.7 - 18.6)	4.0 (3.6 - 4.5)	15.3 (13.9 - 16.8)
	L. monocytogenes	1.9 (1.3-2.7)	5.2 (3.6-7.3)	2.7 (2.0 - 3.5)	7.2 (5.5 - 9.0)	2.6 (2.3 - 3.0)	8.6 (7.7 - 9.5)
	N. meningitidis	6.4 (5.1-7.8)	5.5 (4.4-6.8)	7.9 (7.2 - 8.6)	6.7 (6.0 - 7.4)	8.3 (7.8 - 8.9)	6.6 (6.1 - 7.1)
	Other	2.0 (1.1-3.2)	2.6 (1.4-4.1)	11.0 (7.4 - 15.9)	12.0 (8.1 - 17.2)	11.0 (8.4 - 14.4)	14.0 (10.3 - 18.5)
	S. aureus	4.7 (3.6-6.0)	6.2 (4.8-8.0)	5.7 (5.1 - 6.4)	7.6 (6.7 - 8.6)	5.7 (5.3 - 6.2)	8.5 (7.9 - 9.2)
	S. pneumoniae	2.8 (2.0-3.8)	1.7 (1.2-2.3)	13.6 (12.5 - 14.8)	6.9 (6.3 - 7.7)	13.6 (12.8 - 14.5)	9.5 (8.8 - 10.3)
	Virus	36.7 (33.3-40.3)	10.2 (8.8-11.7)	27.6 (25.7 - 29.1)	8.1 (7.5 - 8.7)	35.2 (33.7 - 36.5)	10.5 (9.9 - 11.1)
New Zealand	*E. coli*	6.6 (5.2-8.1)	13.1 (10.7-15.8)	6.8 (6.0 - 7.8)	13.8 (12.3 - 15.5)	5.6 (5.1 - 6.1)	12.9 (11.9 - 14.0)
	Group B *Streptococcus*	25.9 (22.8-29.0)	29.3 (25.7-33.1)	11.2 (10.3 - 12.2)	15.7 (14.4 - 17.1)	8.1 (7.6 - 8.6)	10.2 (9.6 - 10.8)
	H. influenzae	7.3 (5.6-9.3)	4.3 (3.2-5.7)	9.2 (8.4 - 10.1)	5.2 (4.7 - 5.8)	5.3 (4.9 - 5.7)	3.2 (3.0 - 3.4)
	K. pneumoniae	6.2 (4.7-7.9)	21.7 (17.3-26.5)	3.9 (3.2 - 4.7)	15.7 (13.5 - 18.1)	4.1 (3.7 - 4.5)	15.1 (13.7 - 16.4)
	L. monocytogenes	1.9 (1.3-2.7)	5.1 (3.6-7.0)	2.7 (2.0 - 3.5)	7.0 (5.4 - 8.7)	2.6 (2.3 - 3.0)	8.3 (7.5 - 9.0)
	N. meningitidis	6.6 (5.4-7.8)	5.6 (4.6-6.8)	8.7 (8.0 - 9.4)	7.2 (6.5 - 7.9)	9.1 (8.6 - 9.6)	7.0 (6.6 - 7.5)
	Other	1.9 (1.1-3.1)	2.5 (1.5-4.1)	11.0 (7.4 - 15.9)	11.7 (8.0 - 16.6)	11.0 (8.5 - 14.2)	13.6 (10.2 - 17.7)
	S. aureus	4.5 (3.5-5.7)	6.0 (4.7-7.5)	5.6 (5.0 - 6.2)	7.3 (6.5 - 8.1)	5.6 (5.2 - 6.0)	8.1 (7.6 - 8.7)
	S. pneumoniae	2.8 (2.1-3.6)	2.0 (1.5-2.6)	13.4 (12.4 - 14.5)	8.3 (7.6 - 9.1)	13.6 (12.9 - 14.3)	11.2 (10.4 - 11.9)
	Virus	36.4 (33.2-39.7)	10.3 (9.0-11.7)	27.6 (25.8 - 29.1)	8.1 (7.5 - 8.7)	35.0 (33.6 - 36.2)	10.4 (9.8 - 10.9)
**High-income Asia Pacific**	*E. coli*	6.5 (5.5-7.8)	13.3 (11.2-15.7)	6.8 (5.9 - 7.7)	13.7 (12.2 - 15.2)	5.3 (4.8 - 5.8)	13.7 (12.2 - 15.1)
	Group B *Streptococcus*	24.0 (22.0-26.1)	27.6 (25.1-30.2)	10.2 (9.3 - 11.1)	13.9 (12.8 - 15.0)	6.9 (6.5 - 7.2)	8.1 (7.4 - 8.6)
	H. influenzae	7.9 (6.4-9.5)	4.7 (3.7-5.8)	12.1 (11.0 - 13.3)	6.6 (6.0 - 7.3)	6.7 (6.3 - 7.1)	3.8 (3.4 - 4.1)
	K. pneumoniae	5.8 (4.5-7.2)	20.6 (16.6-24.7)	4.0 (3.2 - 4.8)	15.3 (13.0 - 17.7)	4.2 (3.7 - 4.6)	14.3 (12.7 - 15.9)
	L. monocytogenes	1.9 (1.3-2.6)	5.1 (3.7-6.9)	2.7 (2.0 - 3.4)	7.0 (5.4 - 8.8)	2.6 (2.3 - 2.9)	9.2 (8.3 - 10.1)
	N. meningitidis	8.2 (7.1-9.3)	7.1 (6.1-8.1)	8.9 (8.2 - 9.6)	7.5 (6.8 - 8.2)	9.6 (9.1 - 10.0)	6.1 (5.6 - 6.5)
	Other	1.9 (1.1-3.0)	2.5 (1.5-4.0)	11.1 (7.4 - 16.2)	12.1 (8.2 - 17.3)	11.1 (8.6 - 14.2)	13.3 (9.1 - 18.9)
	S. aureus	4.4 (3.6-5.5)	6.0 (4.9-7.3)	5.3 (4.7 - 5.9)	6.9 (6.2 - 7.7)	5.3 (5.0 - 5.6)	7.9 (7.3 - 8.5)

Location	Aetiology	Neonatal		Under 5		All Ages	
		Nonfatal	Fatal	Nonfatal	Fatal	Nonfatal	Fatal
	S. pneumoniae	2.9 (2.4-3.6)	2.8 (2.3-3.4)	11.2 (10.3 - 12.2)	9.1 (8.3 - 10.0)	11.0 (10.4 - 11.5)	12.7 (11.7 - 13.6)
	Virus	36.4 (33.7-39.1)	10.4 (9.2-11.5)	27.8 (26.0 - 29.3)	8.0 (7.4 - 8.5)	37.4 (36.0 - 38.7)	11.0 (10.2 - 11.7)
Brunei Darussalam	*E. coli*	7.1 (6.0-8.4)	13.7 (11.6-15.9)	5.9 (5.1 - 6.8)	11.4 (10.2 - 12.6)	4.6 (4.1 - 5.2)	10.1 (9.2 - 10.9)
	Group B *Streptococcus*	28.5 (26.2-30.9)	31.3 (28.6-33.9)	9.6 (8.9 - 10.2)	15.1 (14.1 - 16.0)	6.4 (6.1 - 6.7)	8.2 (7.8 - 8.6)
	H. influenzae	6.8 (5.4-8.6)	4.2 (3.2-5.3)	6.1 (5.6 - 6.6)	3.5 (3.2 - 3.9)	3.6 (3.4 - 3.9)	2.2 (2.1 - 2.4)
	K. pneumoniae	5.4 (4.2-6.9)	17.8 (14.3-22.0)	3.8 (3.1 - 4.7)	13.4 (11.4 - 15.5)	3.9 (3.4 - 4.4)	12.9 (11.6 - 14.1)
	L. monocytogenes	1.8 (1.3-2.5)	4.5 (3.2-6.3)	2.3 (1.8 - 3.0)	5.3 (4.2 - 6.5)	2.2 (1.9 - 2.4)	6.2 (5.6 - 6.7)
	N. meningitidis	6.9 (5.9-8.0)	5.8 (4.9-6.7)	15.8 (14.8 - 16.7)	10.6 (9.9 - 11.2)	16.0 (15.4 - 16.6)	10.9 (10.4 - 11.4)
	Other	1.9 (1.2-3.1)	2.4 (1.5-3.9)	10.0 (7.0 - 13.9)	8.8 (6.4 - 12.1)	10.2 (8.3 - 12.4)	11.4 (9.2 - 13.9)
	S. aureus	3.9 (3.1-4.9)	5.1 (4.1-6.2)	4.7 (4.2 - 5.2)	5.5 (4.9 - 6.0)	4.7 (4.5 - 5.0)	6.4 (6.0 - 6.8)
	S. pneumoniae	2.6 (2.1-3.1)	4.0 (3.4-4.9)	16.1 (15.1 - 17.2)	18.2 (16.9 - 19.5)	13.8 (13.2 - 14.4)	21.3 (20.3 - 22.3)
	Virus	35.0 (32.0-38.1)	11.2 (9.9-12.6)	25.6 (24.3 - 26.8)	8.3 (7.8 - 8.8)	34.5 (33.4 - 35.5)	10.3 (9.9 - 10.7)
Japan	*E. coli*	6.5 (5.4-7.8)	13.2 (11.0-15.7)	6.8 (6.0 - 7.7)	13.8 (12.3 - 15.4)	5.4 (5.0 - 5.9)	13.8 (12.3 - 15.3)
	Group B *Streptococcus*	24.1 (21.9-26.4)	27.6 (24.8-30.4)	10.4 (9.5 - 11.4)	13.8 (12.6 - 14.9)	7.0 (6.7 - 7.4)	8.1 (7.4 - 8.7)
	H. influenzae	7.9 (6.3-9.6)	4.7 (3.7-5.8)	11.9 (10.8 - 13.0)	6.6 (5.9 - 7.3)	6.5 (6.0 - 6.9)	3.7 (3.4 - 4.1)
	K. pneumoniae	5.8 (4.5-7.4)	21.0 (16.9-25.2)	3.9 (3.2 - 4.7)	15.3 (13.0 - 17.7)	4.2 (3.8 - 4.6)	14.2 (12.6 - 15.9)
	L. monocytogenes	1.9 (1.4-2.6)	5.2 (3.7-7.0)	2.7 (2.0 - 3.5)	7.1 (5.5 - 8.9)	2.7 (2.4 - 3.0)	9.3 (8.3 - 10.2)
	N. meningitidis	8.0 (6.9-9.2)	6.9 (5.9-8.0)	8.6 (7.9 - 9.3)	7.3 (6.6 - 8.0)	9.2 (8.7 - 9.6)	6.0 (5.5 - 6.4)
	Other	1.9 (1.1-3.0)	2.5 (1.5-4.0)	11.1 (7.4 - 16.1)	12.4 (8.4 - 17.8)	11.1 (8.6 - 14.3)	13.4 (9.0 - 19.1)
	S. aureus	4.5 (3.6-5.6)	6.0 (4.9-7.4)	5.3 (4.8 - 6.0)	7.1 (6.3 - 7.9)	5.4 (5.1 - 5.7)	8.0 (7.3 - 8.6)
	S. pneumoniae	2.9 (2.4-3.6)	2.5 (2.0-3.1)	11.4 (10.5 - 12.4)	8.8 (8.0 - 9.7)	11.3 (10.7 - 11.9)	12.6 (11.5 - 13.5)
	Virus	36.5 (33.6-39.3)	10.3 (9.1-11.5)	27.9 (26.0 - 29.4)	7.9 (7.3 - 8.5)	37.2 (35.8 - 38.5)	11.0 (10.2 - 11.7)
Republic of Korea	*E. coli*	6.6 (5.5-7.8)	13.4 (11.3-15.7)	6.7 (5.8 - 7.6)	13.5 (12.0 - 14.9)	5.1 (4.7 - 5.6)	13.1 (11.8 - 14.4)
	Group B *Streptococcus*	23.9 (22.2-25.5)	27.3 (25.2-29.5)	9.9 (9.0 - 10.8)	14.1 (13.1 - 15.1)	6.7 (6.3 - 7.0)	7.8 (7.3 - 8.3)
	H. influenzae	8.0 (6.6-9.5)	4.8 (3.8-5.8)	12.7 (11.5 - 13.9)	6.8 (6.1 - 7.5)	7.0 (6.6 - 7.4)	4.0 (3.7 - 4.3)
	K. pneumoniae	5.6 (4.4-7.0)	20.1 (16.2-24.1)	4.0 (3.2 - 4.8)	15.3 (13.2 - 17.6)	4.2 (3.7 - 4.6)	14.4 (13.0 - 15.8)
	L. monocytogenes	1.9 (1.3-2.6)	5.1 (3.7-6.8)	2.6 (2.0 - 3.4)	6.7 (5.3 - 8.4)	2.5 (2.2 - 2.8)	8.7 (7.9 - 9.5)
	N. meningitidis	8.6 (7.6-9.7)	7.5 (6.5-8.5)	9.2 (8.5 - 10.0)	7.8 (7.1 - 8.4)	10.2 (9.7 - 10.6)	6.6 (6.2 - 7.0)
	Other	1.9 (1.1-3.0)	2.5 (1.4-3.9)	11.1 (7.4 - 16.1)	11.5 (7.8 - 16.2)	11.1 (8.6 - 14.2)	13.3 (9.4 - 18.3)
	S. aureus	4.4 (3.5-5.3)	5.9 (4.8-7.1)	5.2 (4.6 - 5.8)	6.7 (6.0 - 7.4)	5.1 (4.8 - 5.4)	7.6 (7.1 - 8.1)

Location	Aetiology	Neonatal		Under 5		All Ages	
		Nonfatal	Fatal	Nonfatal	Fatal	Nonfatal	Fatal
	S. pneumoniae	2.9 (2.5-3.5)	3.1 (2.6-3.7)	10.8 (10.0 - 11.7)	9.5 (8.6 - 10.3)	10.5 (10.0 - 11.0)	13.6 (12.6 - 14.4)
	Virus	36.3 (33.6-38.9)	10.4 (9.3-11.6)	27.8 (25.9 - 29.3)	8.1 (7.6 - 8.7)	37.8 (36.3 - 39.0)	10.9 (10.2 - 11.5)
Singapore	*E. coli*	6.5 (5.5-7.8)	13.2 (11.2-15.6)	6.7 (5.8 - 7.5)	13.5 (12.0 - 14.9)	5.1 (4.6 - 5.5)	12.4 (11.5 - 13.5)
	Group B *Streptococcus*	23.6 (21.7-25.6)	27.1 (24.7-29.5)	10.0 (9.2 - 10.9)	14.1 (13.1 - 15.2)	6.8 (6.5 - 7.2)	8.5 (8.0 - 9.0)
	H. influenzae	8.1 (6.6-9.6)	4.9 (3.9-5.9)	12.5 (11.5 - 13.7)	6.8 (6.2 - 7.5)	7.2 (6.7 - 7.6)	4.1 (3.8 - 4.4)
	K. pneumoniae	5.7 (4.5-7.1)	20.5 (16.6-24.6)	3.9 (3.2 - 4.7)	15.4 (13.2 - 17.7)	4.1 (3.7 - 4.6)	15.1 (13.8 - 16.3)
	L. monocytogenes	1.9 (1.4-2.6)	5.1 (3.7-6.9)	2.6 (2.0 - 3.4)	6.8 (5.3 - 8.5)	2.4 (2.2 - 2.7)	8.2 (7.5 - 8.9)
	N. meningitidis	8.5 (7.4-9.6)	7.3 (6.3-8.4)	9.2 (8.5 - 9.9)	7.8 (7.1 - 8.4)	10.1 (9.6 - 10.5)	7.2 (6.8 - 7.6)
	Other	1.9 (1.1-3.0)	2.5 (1.5-4.0)	11.0 (7.4 - 15.9)	11.6 (7.9 - 16.4)	11.0 (8.6 - 14.0)	13.8 (10.3 - 17.8)
	S. aureus	4.4 (3.6-5.5)	6.0 (4.9-7.3)	5.2 (4.7 - 5.8)	6.8 (6.1 - 7.5)	5.1 (4.8 - 5.4)	7.7 (7.3 - 8.2)
	S. pneumoniae	3.0 (2.4-3.6)	2.8 (2.3-3.4)	11.2 (10.4 - 12.1)	9.1 (8.3 - 9.9)	10.7 (10.2 - 11.2)	12.2 (11.5 - 12.9)
	Virus	36.5 (33.8-39.2)	10.5 (9.3-11.7)	27.6 (25.9 - 29.1)	8.1 (7.6 - 8.7)	37.5 (36.0 - 38.7)	10.7 (10.1 - 11.2)
**High-income North America**	*E. coli*	6.4 (5.0-8.0)	12.8 (10.3-15.8)	6.6 (5.8 - 7.6)	13.7 (12.1 - 15.3)	5.8 (5.3 - 6.3)	12.7 (11.6 - 13.8)
	Group B *Streptococcus*	24.8 (21.4-28.2)	28.0 (24.0-32.2)	11.4 (10.4 - 12.3)	14.8 (13.5 - 16.2)	7.8 (7.4 - 8.3)	9.5 (8.9 - 10.1)
	H. influenzae	7.6 (5.7-9.8)	4.6 (3.4-6.1)	9.5 (8.6 - 10.3)	5.5 (5.0 - 6.1)	4.6 (4.3 - 5.0)	3.0 (2.8 - 3.3)
	K. pneumoniae	6.3 (4.8-8.1)	22.2 (17.7-27.4)	3.9 (3.2 - 4.7)	15.3 (13.0 - 17.8)	4.2 (3.8 - 4.6)	14.9 (13.6 - 16.3)
	L. monocytogenes	1.9 (1.3-2.7)	5.1 (3.6-7.1)	2.6 (2.0 - 3.4)	7.0 (5.4 - 8.8)	2.9 (2.6 - 3.2)	8.3 (7.6 - 9.1)
	N. meningitidis	6.7 (5.4-8.0)	5.8 (4.6-7.0)	8.8 (8.1 - 9.5)	7.5 (6.8 - 8.3)	8.6 (8.1 - 9.1)	7.3 (6.8 - 7.8)
	Other	2.0 (1.1-3.2)	2.6 (1.5-4.2)	10.7 (7.2 - 15.4)	12.3 (8.4 - 17.4)	10.9 (8.4 - 14.0)	14.0 (10.6 - 18.1)
	S. aureus	4.6 (3.6-5.9)	6.2 (4.9-7.9)	5.6 (5.0 - 6.3)	7.6 (6.8 - 8.5)	6.1 (5.7 - 6.4)	8.6 (8.0 - 9.3)
	S. pneumoniae	2.9 (2.1-3.8)	1.8 (1.4-2.5)	13.5 (12.5 - 14.6)	8.0 (7.2 - 8.8)	14.5 (13.6 - 15.3)	10.7 (9.9 - 11.5)
	Virus	36.8 (33.6-40.2)	10.8 (9.4-12.3)	27.4 (25.8 - 28.9)	8.3 (7.6 - 8.9)	34.7 (33.4 - 35.8)	11.0 (10.4 - 11.6)
Canada	*E. coli*	6.7 (5.5-8.0)	13.4 (11.1-15.9)	6.8 (6.0 - 7.7)	13.8 (12.3 - 15.4)	5.9 (5.5 - 6.4)	13.0 (12.0 - 14.1)
	Group B *Streptococcus*	26.5 (23.9-29.1)	29.9 (26.8-33.2)	11.3 (10.4 - 12.2)	14.3 (13.2 - 15.4)	7.8 (7.4 - 8.2)	9.2 (8.7 - 9.8)
	H. influenzae	7.2 (5.6-9.0)	4.2 (3.2-5.5)	9.9 (9.0 - 10.7)	5.5 (5.0 - 6.1)	5.0 (4.6 - 5.3)	3.1 (2.9 - 3.3)
	K. pneumoniae	6.0 (4.6-7.5)	21.1 (16.9-25.5)	4.0 (3.3 - 4.7)	15.1 (12.9 - 17.4)	4.3 (3.9 - 4.7)	14.8 (13.5 - 16.1)
	L. monocytogenes	1.9 (1.3-2.6)	5.0 (3.6-6.9)	2.7 (2.0 - 3.4)	7.1 (5.5 - 8.9)	2.9 (2.6 - 3.2)	8.5 (7.8 - 9.3)
	N. meningitidis	6.8 (5.7-7.9)	5.8 (4.8-6.8)	8.8 (8.1 - 9.5)	7.4 (6.7 - 8.1)	8.7 (8.2 - 9.1)	6.8 (6.4 - 7.2)
	Other	1.9 (1.1-3.0)	2.5 (1.4-3.9)	10.8 (7.3 - 15.8)	12.4 (8.4 - 17.8)	11.1 (8.5 - 14.4)	13.7 (10.1 - 18.3)
	S. aureus	4.4 (3.5-5.5)	5.8 (4.7-7.3)	5.3 (4.8 - 5.9)	7.0 (6.3 - 7.8)	5.7 (5.3 - 6.0)	7.9 (7.4 - 8.5)

Location	Aetiology	Neonatal		Under 5		All Ages	
		Nonfatal	Fatal	Nonfatal	Fatal	Nonfatal	Fatal
	S. pneumoniae	2.8 (2.2-3.4)	2.3 (1.8-2.8)	12.7 (11.8 - 13.6)	9.6 (8.8 - 10.5)	13.5 (12.9 - 14.2)	12.6 (11.8 - 13.3)
	Virus	36.0 (32.9-39.1)	10.0 (8.8-11.3)	27.8 (26.1 - 29.3)	7.7 (7.2 - 8.3)	35.2 (33.8 - 36.4)	10.3 (9.7 - 10.9)
Greenland	*E. coli*	6.3 (5.0-7.8)	12.4 (10.0-15.3)	6.2 (5.3 - 7.1)	12.4 (10.7 - 14.3)	5.3 (4.7 - 5.9)	10.8 (9.7 - 12.0)
	Group B *Streptococcus*	23.3 (20.1-26.7)	26.4 (22.6-30.4)	10.7 (9.8 - 11.5)	12.5 (11.3 - 13.5)	7.6 (7.2 - 8.1)	8.8 (8.3 - 9.4)
	H. influenzae	8.2 (6.3-10.3)	5.2 (3.9-6.7)	8.2 (7.5 - 8.9)	5.1 (4.6 - 5.6)	4.4 (4.1 - 4.7)	2.9 (2.7 - 3.1)
	K. pneumoniae	6.2 (4.8-7.9)	20.9 (16.7-25.7)	3.9 (3.1 - 4.7)	13.6 (11.3 - 16.2)	4.1 (3.6 - 4.6)	13.9 (12.3 - 15.5)
	L. monocytogenes	1.9 (1.3-2.8)	5.0 (3.5-7.2)	2.4 (1.8 - 3.1)	6.3 (4.8 - 8.0)	2.6 (2.3 - 2.9)	7.0 (6.2 - 7.7)
	N. meningitidis	7.1 (5.8-8.5)	6.1 (5.0-7.4)	12.0 (11.2 - 12.9)	10.3 (9.4 - 11.3)	11.8 (11.2 - 12.4)	9.7 (9.1 - 10.4)
	Other	2.2 (1.3-3.4)	2.8 (1.7-4.4)	10.0 (6.9 - 13.8)	12.1 (8.4 - 16.6)	10.2 (8.3 - 12.6)	13.1 (10.4 - 16.1)
	S. aureus	4.6 (3.6-5.8)	6.1 (4.8-7.7)	5.7 (5.1 - 6.3)	7.6 (6.7 - 8.4)	6.1 (5.6 - 6.5)	8.4 (7.8 - 9.1)
	S. pneumoniae	2.9 (2.2-3.8)	2.3 (1.8-3.0)	14.5 (13.5 - 15.7)	11.3 (10.3 - 12.4)	15.9 (15.1 - 16.7)	14.0 (13.2 - 14.9)
	Virus	37.3 (34.0-40.7)	12.7 (11.2-14.4)	26.5 (25.1 - 27.8)	8.9 (8.2 - 9.5)	32.0 (30.9 - 33.0)	11.3 (10.8 - 11.9)
United States of America	*E. coli*	6.3 (5.0-8.0)	12.8 (10.2-15.8)	6.6 (5.8 - 7.5)	13.7 (12.1 - 15.3)	5.8 (5.3 - 6.3)	12.6 (11.5 - 13.8)
	Group B *Streptococcus*	24.5 (20.9-28.0)	27.8 (23.8-32.1)	11.4 (10.4 - 12.4)	14.9 (13.6 - 16.3)	7.8 (7.4 - 8.3)	9.5 (8.9 - 10.1)
	H. influenzae	7.7 (5.7-10.0)	4.6 (3.4-6.2)	9.4 (8.5 - 10.3)	5.5 (5.0 - 6.2)	4.6 (4.2 - 4.9)	3.0 (2.7 - 3.3)
	K. pneumoniae	6.3 (4.8-8.3)	22.3 (17.7-27.6)	3.9 (3.2 - 4.7)	15.4 (13.1 - 17.8)	4.2 (3.8 - 4.6)	14.9 (13.6 - 16.4)
	L. monocytogenes	1.9 (1.3-2.8)	5.1 (3.6-7.2)	2.6 (2.0 - 3.3)	7.0 (5.4 - 8.8)	2.9 (2.6 - 3.2)	8.3 (7.5 - 9.1)
	N. meningitidis	6.7 (5.4-8.0)	5.8 (4.6-7.0)	8.8 (8.1 - 9.5)	7.5 (6.8 - 8.3)	8.6 (8.0 - 9.1)	7.3 (6.8 - 7.8)
	Other	2.0 (1.2-3.2)	2.7 (1.5-4.2)	10.6 (7.1 - 15.3)	12.2 (8.4 - 17.3)	10.8 (8.3 - 13.9)	14.0 (10.6 - 18.1)
	S. aureus	4.7 (3.6-6.0)	6.2 (4.9-8.0)	5.7 (5.1 - 6.3)	7.6 (6.8 - 8.5)	6.1 (5.7 - 6.5)	8.7 (8.1 - 9.3)
	S. pneumoniae	2.9 (2.1-3.8)	1.8 (1.3-2.4)	13.6 (12.6 - 14.8)	7.8 (7.1 - 8.6)	14.6 (13.7 - 15.5)	10.5 (9.7 - 11.3)
	Virus	37.0 (33.7-40.4)	10.9 (9.5-12.4)	27.4 (25.7 - 28.8)	8.3 (7.7 - 8.9)	34.6 (33.3 - 35.8)	11.1 (10.4 - 11.7)
**Southern Latin America**	*E. coli*	6.7 (5.5-7.9)	13.0 (10.9-15.3)	6.3 (5.5 - 7.2)	12.2 (10.8 - 13.9)	5.4 (4.9 - 5.8)	11.0 (10.0 - 12.0)
	Group B *Streptococcus*	25.5 (23.2-28.1)	28.6 (25.9-31.4)	10.4 (9.6 - 11.1)	12.5 (11.6 - 13.5)	7.6 (7.2 - 8.0)	8.1 (7.7 - 8.5)
	H. influenzae	7.6 (6.0-9.3)	4.7 (3.6-5.9)	8.4 (7.8 - 9.1)	4.9 (4.5 - 5.4)	4.9 (4.6 - 5.2)	2.7 (2.5 - 2.8)
	K. pneumoniae	5.8 (4.6-7.3)	19.7 (16.0-23.6)	3.9 (3.2 - 4.7)	13.6 (11.6 - 16.0)	4.0 (3.6 - 4.5)	13.5 (12.2 - 14.6)
	L. monocytogenes	1.9 (1.3-2.6)	4.8 (3.4-6.7)	2.4 (1.9 - 3.1)	6.1 (4.8 - 7.6)	2.5 (2.2 - 2.7)	7.0 (6.4 - 7.6)
	N. meningitidis	7.1 (6.0-8.1)	6.1 (5.1-7.0)	12.3 (11.5 - 13.2)	10.2 (9.4 - 11.0)	12.7 (12.2 - 13.2)	9.8 (9.4 - 10.3)
	Other	2.0 (1.2-3.2)	2.6 (1.6-4.1)	10.3 (7.2 - 14.4)	11.5 (8.0 - 15.9)	10.4 (8.3 - 12.7)	12.6 (10.0 - 15.7)
	S. aureus	4.3 (3.5-5.3)	5.7 (4.6-7.0)	5.4 (4.8 - 5.9)	6.8 (6.1 - 7.5)	5.5 (5.2 - 5.8)	7.6 (7.1 - 8.1)

Location	Aetiology	Neonatal		Under 5		All Ages	
		Nonfatal	Fatal	Nonfatal	Fatal	Nonfatal	Fatal
	S. pneumoniae	2.8 (2.3-3.3)	3.0 (2.4-3.5)	13.9 (12.9 - 14.9)	13.5 (12.5 - 14.6)	14.2 (13.7 - 14.8)	16.7 (15.9 - 17.4)
	Virus	36.3 (33.4-39.3)	11.8 (10.4-13.2)	26.7 (25.3 - 28.0)	8.5 (7.9 - 9.0)	32.8 (31.8 - 33.7)	11.1 (10.6 - 11.6)
Argentina	*E. coli*	6.7 (5.5-7.9)	13.0 (10.9-15.3)	6.2 (5.4 - 7.2)	12.1 (10.6 - 13.8)	5.3 (4.8 - 5.8)	10.8 (9.8 - 11.9)
	Group B *Streptococcus*	25.4 (23.1-27.9)	28.5 (25.7-31.2)	10.2 (9.5 - 10.9)	12.3 (11.3 - 13.2)	7.5 (7.1 - 7.8)	8.0 (7.5 - 8.4)
	H. influenzae	7.6 (6.1-9.3)	4.7 (3.7-5.9)	8.4 (7.7 - 9.0)	4.9 (4.5 - 5.4)	4.9 (4.6 - 5.2)	2.7 (2.5 - 2.8)
	K. pneumoniae	5.8 (4.6-7.2)	19.5 (15.8-23.5)	3.9 (3.2 - 4.7)	13.4 (11.4 - 15.8)	4.0 (3.5 - 4.5)	13.3 (12.0 - 14.5)
	L. monocytogenes	1.9 (1.3-2.6)	4.8 (3.4-6.7)	2.4 (1.8 - 3.1)	6.1 (4.8 - 7.6)	2.4 (2.2 - 2.7)	6.9 (6.2 - 7.5)
	N. meningitidis	7.2 (6.1-8.2)	6.1 (5.2-7.1)	12.9 (12.1 - 13.8)	10.4 (9.6 - 11.3)	13.3 (12.8 - 13.8)	10.2 (9.7 - 10.6)
	Other	2.1 (1.3-3.2)	2.7 (1.6-4.1)	10.2 (7.1 - 14.3)	11.5 (8.0 - 15.9)	10.3 (8.3 - 12.6)	12.5 (10.0 - 15.4)
	S. aureus	4.3 (3.5-5.3)	5.6 (4.6-6.9)	5.3 (4.8 - 5.9)	6.8 (6.1 - 7.5)	5.4 (5.1 - 5.7)	7.5 (7.0 - 8.0)
	S. pneumoniae	2.8 (2.3-3.3)	3.1 (2.5-3.7)	13.9 (13.0 - 14.9)	13.9 (12.9 - 15.1)	14.3 (13.8 - 14.8)	17.1 (16.3 - 17.9)
	Virus	36.4 (33.4-39.3)	12.0 (10.6-13.4)	26.5 (25.1 - 27.8)	8.5 (7.9 - 9.1)	32.6 (31.6 - 33.5)	11.2 (10.7 - 11.6)
Chile	*E. coli*	6.7 (5.6-7.9)	13.2 (11.1-15.4)	6.5 (5.7 - 7.4)	12.9 (11.5 - 14.3)	5.5 (5.1 - 6.0)	11.4 (10.5 - 12.3)
	Group B *Streptococcus*	26.0 (23.6-28.6)	29.2 (26.4-32.3)	10.7 (9.9 - 11.5)	13.9 (12.9 - 15.0)	7.9 (7.5 - 8.3)	8.5 (8.1 - 9.0)
	H. influenzae	7.4 (5.8-9.2)	4.5 (3.4-5.7)	8.5 (7.8 - 9.1)	4.9 (4.4 - 5.4)	5.1 (4.8 - 5.4)	2.7 (2.5 - 2.8)
	K. pneumoniae	5.9 (4.6-7.3)	20.2 (16.4-24.3)	3.9 (3.2 - 4.7)	14.4 (12.4 - 16.8)	4.1 (3.7 - 4.5)	14.2 (13.0 - 15.3)
	L. monocytogenes	1.9 (1.3-2.6)	4.9 (3.5-6.7)	2.5 (1.9 - 3.2)	6.5 (5.1 - 8.0)	2.5 (2.3 - 2.8)	7.4 (6.8 - 8.0)
	N. meningitidis	6.9 (5.8-7.9)	5.9 (4.9-6.8)	11.0 (10.2 - 11.8)	8.9 (8.1 - 9.6)	11.4 (10.9 - 11.9)	8.8 (8.4 - 9.3)
	Other	2.0 (1.2-3.2)	2.6 (1.6-4.0)	10.6 (7.2 - 14.9)	11.5 (8.0 - 16.1)	10.6 (8.3 - 13.2)	13.2 (10.3 - 16.6)
	S. aureus	4.3 (3.5-5.3)	5.7 (4.7-7.0)	5.4 (4.9 - 6.0)	6.9 (6.2 - 7.6)	5.5 (5.2 - 5.8)	7.8 (7.3 - 8.2)
	S. pneumoniae	2.8 (2.2-3.3)	2.7 (2.2-3.2)	13.7 (12.8 - 14.7)	11.6 (10.7 - 12.6)	14.1 (13.5 - 14.6)	15.1 (14.4 - 15.9)
	Virus	36.2 (33.3-39.2)	11.2 (9.8-12.5)	27.2 (25.6 - 28.5)	8.4 (7.8 - 8.9)	33.4 (32.3 - 34.4)	10.9 (10.4 - 11.3)
Uruguay	*E. coli*	6.4 (5.2-7.8)	12.7 (10.4-15.3)	6.3 (5.4 - 7.2)	12.7 (11.1 - 14.3)	5.4 (4.8 - 5.9)	12.2 (10.9 - 13.7)
	Group B *Streptococcus*	24.3 (21.4-27.5)	27.5 (24.1-31.2)	10.5 (9.6 - 11.3)	13.4 (12.3 - 14.5)	7.6 (7.2 - 8.0)	8.2 (7.6 - 8.8)
	H. influenzae	7.9 (6.1-9.8)	4.9 (3.7-6.3)	8.5 (7.8 - 9.2)	5.2 (4.7 - 5.7)	4.7 (4.4 - 5.1)	2.7 (2.5 - 3.0)
	K. pneumoniae	6.1 (4.7-7.8)	20.8 (16.8-25.5)	3.8 (3.1 - 4.6)	14.2 (12.0 - 16.6)	4.0 (3.6 - 4.5)	13.1 (11.6 - 14.8)
	L. monocytogenes	1.9 (1.3-2.7)	5.0 (3.5-7.0)	2.5 (1.9 - 3.1)	6.4 (5.0 - 8.0)	2.5 (2.3 - 2.8)	8.0 (7.1 - 8.9)
	N. meningitidis	7.0 (5.8-8.2)	6.0 (4.9-7.1)	11.5 (10.6 - 12.3)	9.5 (8.7 - 10.3)	11.7 (11.1 - 12.3)	8.2 (7.6 - 8.7)
	Other	2.1 (1.3-3.3)	2.7 (1.7-4.3)	10.4 (7.1 - 14.6)	11.8 (8.3 - 16.3)	10.4 (8.3 - 12.7)	12.5 (9.1 - 16.8)
	S. aureus	4.5 (3.6-5.6)	6.0 (4.8-7.4)	5.6 (5.0 - 6.2)	7.3 (6.5 - 8.1)	5.7 (5.4 - 6.1)	8.3 (7.6 - 8.9)

Location	Aetiology	Neonatal		Under 5		All Ages	
		Nonfatal	Fatal	Nonfatal	Fatal	Nonfatal	Fatal
	S. pneumoniae	2.8 (2.2-3.6)	2.4 (1.8-3.0)	14.4 (13.4 - 15.5)	10.9 (10.0 - 11.9)	14.8 (14.1 - 15.5)	15.0 (13.9 - 16.0)
	Virus	36.9 (33.7-40.2)	12.0 (10.5-13.5)	26.5 (25.0 - 27.8)	8.7 (8.1 - 9.3)	33.1 (32.0 - 34.1)	11.8 (11.1 - 12.4)
**Western Europe**	*E. coli*	6.7 (5.4-8.1)	13.3 (10.9-15.9)	6.9 (6.0 - 7.8)	13.9 (12.4 - 15.6)	5.8 (5.3 - 6.3)	13.2 (12.1 - 14.4)
	Group B *Streptococcus*	26.9 (24.1-29.8)	30.1 (26.6-33.7)	11.3 (10.3 - 12.3)	15.2 (14.0 - 16.5)	8.0 (7.6 - 8.5)	9.0 (8.4 - 9.6)
	H. influenzae	7.0 (5.4-9.0)	4.2 (3.1-5.5)	8.8 (8.0 - 9.7)	5.0 (4.5 - 5.5)	4.9 (4.6 - 5.3)	2.8 (2.5 - 3.0)
	K. pneumoniae	6.1 (4.6-7.7)	21.5 (17.2-26.3)	4.0 (3.2 - 4.8)	15.4 (13.2 - 17.7)	4.1 (3.8 - 4.6)	14.5 (13.1 - 15.9)
	L. monocytogenes	1.9 (1.3-2.7)	5.0 (3.5-7.0)	2.7 (2.0 - 3.5)	7.1 (5.5 - 8.9)	2.8 (2.5 - 3.1)	8.8 (8.0 - 9.6)
	N. meningitidis	6.4 (5.3-7.5)	5.5 (4.5-6.5)	8.8 (8.1 - 9.5)	7.2 (6.5 - 7.9)	9.0 (8.5 - 9.4)	6.5 (6.1 - 6.9)
	Other	1.9 (1.1-3.1)	2.5 (1.4-4.0)	11.0 (7.3 - 16.0)	12.0 (8.2 - 17.1)	11.0 (8.4 - 14.4)	13.5 (9.7 - 18.2)
	S. aureus	4.4 (3.4-5.5)	5.9 (4.6-7.3)	5.5 (4.9 - 6.1)	7.2 (6.4 - 8.0)	5.6 (5.2 - 6.0)	8.0 (7.5 - 8.6)
	S. pneumoniae	2.7 (2.1-3.5)	2.1 (1.6-2.6)	13.4 (12.4 - 14.4)	9.2 (8.4 - 10.1)	13.7 (13.0 - 14.4)	13.2 (12.3 - 14.0)
	Virus	36.0 (32.8-39.2)	10.0 (8.8-11.4)	27.8 (26.0 - 29.3)	7.9 (7.3 - 8.5)	35.0 (33.7 - 36.2)	10.5 (9.8 - 11.1)
Andorra	*E. coli*	6.7 (5.4-8.1)	13.4 (11.0-16.0)	7.0 (6.1 - 8.0)	14.1 (12.5 - 15.8)	5.5 (5.1 - 6.0)	12.9 (11.8 - 14.2)
	Group B *Streptococcus*	27.2 (24.3-30.2)	30.6 (27.1-34.3)	11.2 (10.2 - 12.3)	14.9 (13.7 - 16.2)	7.7 (7.3 - 8.2)	9.0 (8.4 - 9.6)
	H. influenzae	6.9 (5.3-8.9)	4.0 (3.0-5.4)	8.6 (7.8 - 9.5)	4.8 (4.3 - 5.4)	4.7 (4.3 - 5.0)	2.7 (2.4 - 2.9)
	K. pneumoniae	6.1 (4.6-7.8)	21.4 (17.1-26.2)	3.9 (3.2 - 4.8)	15.3 (13.0 - 17.7)	4.1 (3.7 - 4.6)	14.9 (13.5 - 16.3)
	L. monocytogenes	1.9 (1.3-2.6)	5.0 (3.5-7.0)	2.8 (2.1 - 3.6)	7.2 (5.5 - 9.1)	2.7 (2.4 - 3.0)	8.7 (7.9 - 9.6)
	N. meningitidis	6.3 (5.2-7.4)	5.4 (4.4-6.4)	8.5 (7.8 - 9.2)	7.0 (6.4 - 7.7)	8.9 (8.4 - 9.3)	6.6 (6.1 - 7.0)
	Other	1.9 (1.1-3.0)	2.4 (1.4-3.9)	11.2 (7.5 - 16.2)	12.4 (8.4 - 17.8)	11.1 (8.6 - 14.4)	14.0 (10.2 - 18.7)
	S. aureus	4.4 (3.4-5.5)	5.8 (4.6-7.3)	5.5 (4.9 - 6.2)	7.2 (6.4 - 8.1)	5.6 (5.2 - 5.9)	8.2 (7.6 - 8.8)
	S. pneumoniae	2.7 (2.1-3.4)	2.1 (1.6-2.6)	13.5 (12.5 - 14.6)	9.2 (8.4 - 10.2)	13.6 (12.9 - 14.2)	12.6 (11.7 - 13.3)
	Virus	35.9 (32.7-39.1)	9.8 (8.6-11.1)	27.8 (26.0 - 29.4)	7.7 (7.2 - 8.3)	36.1 (34.7 - 37.4)	10.4 (9.8 - 11.0)
Austria	*E. coli*	7.0 (5.9-8.1)	13.9 (11.9-15.9)	6.8 (6.1 - 7.7)	13.5 (12.1 - 14.9)	5.9 (5.5 - 6.3)	12.8 (11.7 - 13.9)
	Group B *Streptococcus*	28.5 (26.6-30.5)	31.8 (29.5-34.1)	11.0 (10.2 - 11.8)	15.1 (14.1 - 16.1)	7.7 (7.3 - 8.1)	8.3 (7.8 - 8.8)
	H. influenzae	6.7 (5.3-8.4)	3.9 (3.0-4.9)	9.1 (8.3 - 9.9)	4.8 (4.4 - 5.3)	5.1 (4.7 - 5.4)	2.7 (2.5 - 2.9)
	K. pneumoniae	5.6 (4.4-6.9)	19.6 (15.9-23.5)	4.0 (3.3 - 4.8)	15.0 (12.9 - 17.2)	4.3 (3.9 - 4.6)	14.1 (12.9 - 15.4)
	L. monocytogenes	1.8 (1.3-2.5)	4.8 (3.5-6.5)	2.7 (2.0 - 3.4)	6.7 (5.3 - 8.4)	2.8 (2.5 - 3.1)	8.5 (7.8 - 9.2)
	N. meningitidis	6.7 (5.8-7.7)	5.7 (4.8-6.5)	9.8 (9.1 - 10.5)	7.7 (7.0 - 8.3)	9.8 (9.4 - 10.2)	6.7 (6.3 - 7.1)
	Other	1.8 (1.0-2.9)	2.3 (1.4-3.7)	10.9 (7.3 - 15.8)	11.3 (7.7 - 16.0)	11.1 (8.5 - 14.4)	12.9 (9.3 - 17.6)
	S. aureus	4.1 (3.3-5.1)	5.4 (4.4-6.7)	5.1 (4.6 - 5.6)	6.4 (5.7 - 7.0)	5.3 (5.0 - 5.6)	7.3 (6.8 - 7.8)

Location	Aetiology	Neonatal		Under 5		All Ages	
		Nonfatal	Fatal	Nonfatal	Fatal	Nonfatal	Fatal
	S. pneumoniae	2.6 (2.2-3.1)	3.0 (2.5-3.5)	12.6 (11.7 - 13.5)	12.0 (11.0 - 12.9)	13.0 (12.5 - 13.5)	16.5 (15.4 - 17.4)
	Virus	35.1 (32.2-38.0)	9.7 (8.5-10.8)	28.0 (26.2 - 29.4)	7.7 (7.2 - 8.2)	35.0 (33.7 - 36.2)	10.1 (9.5 - 10.7)
Belgium	*E. coli*	6.6 (5.2-8.1)	13.1 (10.5-16.0)	6.9 (6.0 - 7.8)	14.1 (12.5 - 15.9)	5.8 (5.3 - 6.3)	13.5 (12.3 - 14.7)
	Group B *Streptococcus*	26.2 (22.9-29.6)	29.6 (25.8-33.8)	11.5 (10.5 - 12.5)	14.5 (13.3 - 15.9)	8.3 (7.7 - 8.8)	9.5 (8.8 - 10.1)
	H. influenzae	7.2 (5.4-9.4)	4.2 (3.1-5.6)	8.7 (7.9 - 9.6)	5.0 (4.5 - 5.6)	5.1 (4.7 - 5.5)	2.9 (2.7 - 3.2)
	K. pneumoniae	6.2 (4.8-8.0)	22.0 (17.4-27.0)	3.9 (3.2 - 4.7)	15.2 (12.8 - 17.6)	4.1 (3.7 - 4.5)	14.6 (13.2 - 16.1)
	L. monocytogenes	1.9 (1.3-2.7)	5.1 (3.5-7.1)	2.7 (2.0 - 3.5)	7.3 (5.6 - 9.3)	2.8 (2.4 - 3.1)	8.9 (8.0 - 9.8)
	N. meningitidis	6.3 (5.1-7.6)	5.4 (4.3-6.6)	8.4 (7.6 - 9.1)	7.1 (6.4 - 7.9)	8.6 (8.1 - 9.1)	6.5 (6.0 - 6.9)
	Other	1.9 (1.1-3.1)	2.5 (1.4-4.1)	11.0 (7.3 - 15.9)	12.8 (8.6 - 18.4)	11.0 (8.4 - 14.4)	13.7 (9.9 - 18.4)
	S. aureus	4.5 (3.5-5.7)	6.0 (4.7-7.6)	5.6 (5.0 - 6.3)	7.5 (6.7 - 8.4)	5.7 (5.3 - 6.1)	8.4 (7.7 - 9.0)
	S. pneumoniae	2.8 (2.1-3.6)	1.9 (1.4-2.4)	13.7 (12.6 - 14.8)	8.6 (7.8 - 9.5)	13.9 (13.2 - 14.7)	11.5 (10.7 - 12.3)
	Virus	36.3 (33.1-39.7)	10.1 (8.9-11.6)	27.7 (25.9 - 29.2)	7.8 (7.2 - 8.4)	34.8 (33.4 - 36.1)	10.6 (9.9 - 11.3)
Cyprus	*E. coli*	6.7 (5.5-8.1)	13.4 (11.2-15.9)	6.9 (6.1 - 7.8)	13.8 (12.4 - 15.4)	5.8 (5.4 - 6.3)	13.3 (12.2 - 14.6)
	Group B *Streptococcus*	27.1 (24.5-29.9)	30.5 (27.3-33.8)	11.2 (10.3 - 12.2)	16.6 (15.4 - 18.0)	8.4 (7.9 - 8.9)	9.3 (8.7 - 9.9)
	H. influenzae	7.0 (5.4-8.9)	4.1 (3.1-5.3)	8.7 (7.9 - 9.5)	4.8 (4.3 - 5.3)	5.7 (5.2 - 6.1)	2.9 (2.6 - 3.1)
	K. pneumoniae	6.0 (4.6-7.5)	21.0 (16.7-25.5)	4.0 (3.3 - 4.8)	15.9 (13.7 - 18.2)	4.1 (3.7 - 4.5)	14.4 (13.1 - 15.9)
	L. monocytogenes	1.9 (1.3-2.6)	5.0 (3.5-6.9)	2.7 (2.0 - 3.5)	6.8 (5.3 - 8.4)	2.7 (2.4 - 3.0)	8.7 (7.9 - 9.6)
	N. meningitidis	6.5 (5.4-7.6)	5.5 (4.6-6.5)	8.9 (8.2 - 9.6)	7.1 (6.5 - 7.8)	9.3 (8.8 - 9.8)	6.5 (6.1 - 6.9)
	Other	1.9 (1.1-3.0)	2.4 (1.4-3.9)	11.0 (7.3 - 15.9)	11.0 (7.5 - 15.4)	11.0 (8.4 - 14.3)	13.1 (9.3 - 17.9)
	S. aureus	4.4 (3.4-5.4)	5.8 (4.6-7.1)	5.4 (4.9 - 6.1)	6.9 (6.2 - 7.7)	5.5 (5.1 - 5.8)	7.9 (7.3 - 8.4)
	S. pneumoniae	2.7 (2.2-3.4)	2.3 (1.8-2.8)	13.3 (12.3 - 14.2)	9.0 (8.2 - 9.8)	13.2 (12.6 - 13.9)	13.3 (12.4 - 14.2)
	Virus	35.9 (32.8-39.0)	10.0 (8.8-11.3)	27.9 (26.1 - 29.4)	8.1 (7.5 - 8.6)	34.3 (33.0 - 35.6)	10.5 (9.8 - 11.1)
Denmark	*E. coli*	6.5 (5.2-8.1)	13.1 (10.6-15.9)	6.8 (6.0 - 7.8)	13.9 (12.4 - 15.6)	5.7 (5.2 - 6.2)	13.6 (12.3 - 14.9)
	Group B *Streptococcus*	25.8 (22.6-29.1)	29.2 (25.5-33.2)	11.3 (10.4 - 12.3)	15.7 (14.4 - 17.1)	8.0 (7.5 - 8.5)	9.0 (8.3 - 9.7)
	H. influenzae	7.3 (5.6-9.4)	4.3 (3.2-5.7)	9.1 (8.2 - 10.0)	5.2 (4.6 - 5.7)	5.0 (4.6 - 5.4)	2.9 (2.6 - 3.1)
	K. pneumoniae	6.2 (4.7-8.0)	21.9 (17.5-26.8)	3.9 (3.2 - 4.7)	15.8 (13.5 - 18.1)	4.1 (3.7 - 4.5)	14.4 (13.0 - 16.0)
	L. monocytogenes	1.9 (1.3-2.7)	5.1 (3.6-7.1)	2.7 (2.0 - 3.5)	7.0 (5.5 - 8.8)	2.7 (2.4 - 3.0)	9.1 (8.2 - 10.0)
	N. meningitidis	6.5 (5.3-7.8)	5.6 (4.5-6.7)	8.5 (7.8 - 9.2)	7.1 (6.4 - 7.8)	8.7 (8.2 - 9.2)	6.2 (5.7 - 6.6)
	Other	1.9 (1.1-3.1)	2.5 (1.4-4.1)	11.0 (7.4 - 15.9)	11.8 (8.1 - 16.9)	11.0 (8.4 - 14.2)	13.5 (9.6 - 18.6)
	S. aureus	4.5 (3.5-5.7)	6.0 (4.7-7.6)	5.6 (5.0 - 6.3)	7.4 (6.6 - 8.2)	5.7 (5.3 - 6.1)	8.4 (7.8 - 9.1)

Location	Aetiology	Neonatal		Under 5		All Ages	
		Nonfatal	Fatal	Nonfatal	Fatal	Nonfatal	Fatal
	S. pneumoniae	2.8 (2.1-3.6)	1.9 (1.4-2.5)	13.5 (12.4 - 14.6)	8.0 (7.3 - 8.8)	13.8 (13.0 - 14.6)	12.0 (11.1 - 12.9)
	Virus	36.4 (33.2-39.8)	10.2 (9.0-11.6)	27.7 (25.9 - 29.2)	8.1 (7.5 - 8.7)	35.2 (33.7 - 36.4)	10.9 (10.1 - 11.5)
Finland	*E. coli*	6.8 (5.6-8.1)	13.5 (11.4-15.9)	6.9 (6.1 - 7.8)	13.8 (12.3 - 15.4)	5.8 (5.4 - 6.3)	13.1 (11.9 - 14.3)
	Group B *Streptococcus*	27.5 (25.1-30.1)	30.9 (27.9-34.0)	11.0 (10.1 - 11.9)	14.1 (13.0 - 15.3)	7.8 (7.3 - 8.2)	8.4 (7.8 - 8.9)
	H. influenzae	6.9 (5.4-8.7)	4.0 (3.0-5.2)	9.3 (8.5 - 10.1)	5.1 (4.6 - 5.7)	4.7 (4.4 - 5.1)	2.6 (2.4 - 2.9)
	K. pneumoniae	5.9 (4.5-7.4)	20.7 (16.6-25.1)	4.0 (3.2 - 4.8)	14.9 (12.7 - 17.3)	4.2 (3.8 - 4.6)	14.4 (13.1 - 15.8)
	L. monocytogenes	1.9 (1.3-2.6)	4.9 (3.5-6.8)	2.7 (2.0 - 3.5)	7.1 (5.5 - 9.0)	2.8 (2.5 - 3.1)	8.9 (8.1 - 9.7)
	N. meningitidis	6.5 (5.5-7.5)	5.5 (4.6-6.5)	9.0 (8.4 - 9.8)	7.5 (6.8 - 8.1)	9.2 (8.7 - 9.6)	6.4 (6.0 - 6.8)
	Other	1.9 (1.1-3.0)	2.4 (1.4-3.9)	11.1 (7.4 - 16.2)	12.4 (8.4 - 17.8)	11.1 (8.5 - 14.5)	13.5 (9.6 - 18.4)
	S. aureus	4.3 (3.4-5.3)	5.7 (4.6-7.0)	5.3 (4.8 - 5.9)	6.9 (6.2 - 7.7)	5.5 (5.2 - 5.9)	7.9 (7.3 - 8.4)
	S. pneumoniae	2.7 (2.2-3.3)	2.4 (1.9-2.9)	13.1 (12.1 - 14.0)	10.6 (9.7 - 11.5)	13.7 (13.0 - 14.3)	14.4 (13.4 - 15.3)
	Virus	35.6 (32.6-38.8)	9.8 (8.7-11.1)	27.7 (26.0 - 29.2)	7.6 (7.1 - 8.2)	35.2 (33.9 - 36.5)	10.5 (9.8 - 11.1)
France	*E. coli*	6.6 (5.2-8.1)	13.2 (10.7-16.0)	6.9 (6.0 - 7.8)	13.9 (12.4 - 15.6)	5.9 (5.4 - 6.4)	13.6 (12.3 - 14.9)
	Group B *Streptococcus*	26.4 (23.2-29.6)	29.8 (26.1-33.8)	11.5 (10.5 - 12.5)	16.7 (15.3 - 18.2)	8.4 (7.9 - 8.9)	9.5 (8.8 - 10.1)
	H. influenzae	7.1 (5.4-9.3)	4.2 (3.1-5.6)	8.9 (8.0 - 9.8)	5.0 (4.4 - 5.6)	5.2 (4.8 - 5.6)	2.9 (2.6 - 3.1)
	K. pneumoniae	6.2 (4.7-8.0)	21.9 (17.4-26.8)	3.9 (3.2 - 4.8)	16.1 (13.8 - 18.6)	4.1 (3.7 - 4.5)	14.6 (13.2 - 16.1)
	L. monocytogenes	1.9 (1.3-2.7)	5.1 (3.5-7.1)	2.7 (2.0 - 3.5)	7.0 (5.4 - 8.7)	2.8 (2.4 - 3.1)	9.0 (8.1 - 9.9)
	N. meningitidis	6.4 (5.2-7.6)	5.4 (4.4-6.6)	8.4 (7.7 - 9.1)	6.9 (6.2 - 7.6)	8.6 (8.1 - 9.1)	6.2 (5.7 - 6.6)
	Other	1.9 (1.1-3.1)	2.5 (1.4-4.0)	10.9 (7.3 - 15.9)	11.3 (7.7 - 16.0)	11.0 (8.4 - 14.4)	13.5 (9.5 - 18.4)
	S. aureus	4.5 (3.5-5.7)	6.0 (4.7-7.5)	5.6 (5.0 - 6.2)	7.2 (6.4 - 8.1)	5.7 (5.3 - 6.1)	8.3 (7.7 - 9.0)
	S. pneumoniae	2.8 (2.1-3.6)	1.9 (1.4-2.5)	13.5 (12.5 - 14.6)	7.8 (7.1 - 8.6)	13.9 (13.1 - 14.7)	11.9 (11.0 - 12.7)
	Virus	36.2 (33.0-39.6)	10.1 (8.8-11.5)	27.8 (26.0 - 29.3)	8.1 (7.5 - 8.7)	34.5 (33.2 - 35.8)	10.6 (9.9 - 11.3)
Germany	*E. coli*	6.9 (5.8-8.1)	13.7 (11.7-15.9)	6.9 (6.1 - 7.8)	13.6 (12.2 - 15.2)	5.9 (5.4 - 6.3)	12.9 (11.8 - 14.1)
	Group B *Streptococcus*	28.2 (26.0-30.6)	31.5 (28.9-34.3)	10.9 (10.1 - 11.9)	13.9 (12.8 - 15.0)	7.8 (7.4 - 8.3)	8.3 (7.7 - 8.8)
	H. influenzae	6.7 (5.2-8.5)	3.9 (3.0-5.1)	8.5 (7.7 - 9.3)	4.7 (4.2 - 5.2)	4.8 (4.5 - 5.2)	2.6 (2.4 - 2.8)
	K. pneumoniae	5.8 (4.5-7.2)	20.2 (16.3-24.3)	4.0 (3.3 - 4.8)	14.7 (12.5 - 17.1)	4.2 (3.8 - 4.6)	14.1 (12.8 - 15.4)
	L. monocytogenes	1.8 (1.3-2.6)	4.9 (3.5-6.6)	2.7 (2.1 - 3.5)	6.9 (5.4 - 8.8)	2.8 (2.5 - 3.1)	8.6 (7.9 - 9.4)
	N. meningitidis	6.5 (5.5-7.5)	5.5 (4.6-6.4)	9.5 (8.8 - 10.3)	7.7 (7.0 - 8.4)	9.7 (9.2 - 10.1)	6.7 (6.3 - 7.1)
	Other	1.8 (1.1-3.0)	2.4 (1.4-3.8)	11.1 (7.4 - 16.0)	12.2 (8.3 - 17.4)	11.0 (8.5 - 14.3)	13.1 (9.3 - 17.9)
	S. aureus	4.2 (3.4-5.2)	5.5 (4.5-6.8)	5.3 (4.7 - 5.9)	6.7 (6.0 - 7.5)	5.4 (5.1 - 5.8)	7.6 (7.1 - 8.1)

Location	Aetiology	Neonatal		Under 5		All Ages	
		Nonfatal	Fatal	Nonfatal	Fatal	Nonfatal	Fatal
	S. pneumoniae	2.6 (2.2-3.2)	2.7 (2.2-3.2)	13.2 (12.2 - 14.1)	11.8 (10.9 - 12.8)	13.6 (13.0 - 14.1)	15.7 (14.7 - 16.6)
	Virus	35.3 (32.4-38.3)	9.8 (8.6-11.0)	27.9 (26.1 - 29.4)	7.6 (7.1 - 8.2)	34.8 (33.4 - 36.0)	10.4 (9.7 - 10.9)
Greece	*E. coli*	6.8 (5.7-8.0)	13.5 (11.4-15.8)	6.9 (6.1 - 7.8)	13.7 (12.3 - 15.2)	5.9 (5.5 - 6.3)	12.8 (11.7 - 13.8)
	Group B *Streptococcus*	27.3 (24.9-29.7)	30.6 (27.8-33.5)	11.1 (10.2 - 12.0)	15.3 (14.2 - 16.5)	8.0 (7.6 - 8.5)	8.8 (8.3 - 9.3)
	H. influenzae	7.0 (5.5-8.8)	4.1 (3.1-5.3)	8.8 (8.0 - 9.6)	4.8 (4.4 - 5.4)	5.2 (4.9 - 5.6)	2.8 (2.5 - 3.0)
	K. pneumoniae	5.9 (4.5-7.3)	20.5 (16.5-24.7)	4.0 (3.3 - 4.8)	15.3 (13.2 - 17.6)	4.2 (3.8 - 4.6)	14.5 (13.2 - 15.7)
	L. monocytogenes	1.9 (1.3-2.6)	4.9 (3.5-6.7)	2.7 (2.0 - 3.5)	6.8 (5.3 - 8.6)	2.8 (2.5 - 3.1)	8.4 (7.7 - 9.1)
	N. meningitidis	6.7 (5.7-7.7)	5.7 (4.8-6.6)	9.3 (8.6 - 10.0)	7.5 (6.8 - 8.1)	9.5 (9.1 - 10.0)	7.0 (6.6 - 7.4)
	Other	1.9 (1.1-3.0)	2.4 (1.4-3.9)	11.0 (7.3 - 15.9)	11.5 (7.8 - 16.2)	11.0 (8.4 - 14.3)	13.3 (9.8 - 17.9)
	S. aureus	4.3 (3.4-5.3)	5.7 (4.6-7.0)	5.4 (4.8 - 6.0)	6.8 (6.1 - 7.6)	5.5 (5.2 - 5.8)	7.7 (7.2 - 8.2)
	S. pneumoniae	2.7 (2.2-3.3)	2.5 (2.0-3.1)	13.0 (12.0 - 14.0)	10.2 (9.4 - 11.1)	13.4 (12.8 - 14.0)	14.3 (13.4 - 15.1)
	Virus	35.7 (32.7-38.7)	10.0 (8.8-11.3)	27.9 (26.2 - 29.5)	8.0 (7.4 - 8.5)	34.5 (33.2 - 35.7)	10.5 (9.9 - 11.0)
Iceland	*E. coli*	6.8 (5.6-8.1)	13.5 (11.3-16.0)	6.9 (6.1 - 7.9)	13.9 (12.4 - 15.6)	5.7 (5.2 - 6.2)	13.2 (12.1 - 14.3)
	Group B *Streptococcus*	27.6 (25.0-30.3)	31.0 (27.8-34.2)	11.2 (10.2 - 12.2)	15.3 (14.2 - 16.6)	7.9 (7.4 - 8.4)	9.0 (8.4 - 9.6)
	H. influenzae	6.9 (5.3-8.8)	4.0 (3.0-5.2)	9.2 (8.4 - 10.1)	5.1 (4.6 - 5.6)	4.9 (4.5 - 5.3)	2.8 (2.5 - 3.0)
	K. pneumoniae	5.9 (4.5-7.5)	21.0 (16.8-25.4)	4.0 (3.2 - 4.8)	15.4 (13.2 - 17.8)	4.1 (3.7 - 4.6)	14.7 (13.4 - 16.1)
	L. monocytogenes	1.9 (1.3-2.6)	5.0 (3.5-6.8)	2.7 (2.1 - 3.5)	7.1 (5.5 - 8.9)	2.7 (2.4 - 3.0)	8.7 (7.9 - 9.6)
	N. meningitidis	6.5 (5.4-7.5)	5.5 (4.5-6.5)	8.6 (8.0 - 9.3)	7.1 (6.4 - 7.7)	9.0 (8.6 - 9.5)	6.5 (6.1 - 6.9)
	Other	1.8 (1.1-3.0)	2.4 (1.4-3.8)	11.1 (7.4 - 16.2)	11.9 (8.1 - 17.1)	11.1 (8.5 - 14.6)	13.6 (9.9 - 18.4)
	S. aureus	4.3 (3.4-5.4)	5.7 (4.6-7.1)	5.4 (4.8 - 6.0)	6.9 (6.2 - 7.7)	5.4 (5.1 - 5.8)	7.9 (7.3 - 8.4)
	S. pneumoniae	2.7 (2.1-3.3)	2.3 (1.8-2.8)	13.0 (12.0 - 13.9)	9.6 (8.7 - 10.4)	13.4 (12.7 - 14.0)	13.3 (12.4 - 14.1)
	Virus	35.7 (32.6-38.8)	9.7 (8.5-11.0)	27.9 (26.1 - 29.5)	7.7 (7.2 - 8.3)	35.7 (34.2 - 37.0)	10.3 (9.6 - 10.9)
Ireland	*E. coli*	6.7 (5.4-8.1)	13.4 (11.0-16.0)	6.9 (6.1 - 7.9)	14.0 (12.5 - 15.7)	5.8 (5.3 - 6.3)	13.0 (11.9 - 14.1)
	Group B *Streptococcus*	27.0 (24.2-29.9)	30.4 (27.0-34.0)	11.3 (10.4 - 12.4)	15.8 (14.6 - 17.2)	8.3 (7.8 - 8.9)	9.8 (9.2 - 10.4)
	H. influenzae	7.0 (5.3-9.0)	4.1 (3.0-5.4)	9.1 (8.2 - 10.0)	5.0 (4.5 - 5.6)	5.4 (5.0 - 5.8)	2.9 (2.7 - 3.2)
	K. pneumoniae	6.0 (4.6-7.7)	21.4 (17.1-26.1)	4.0 (3.2 - 4.8)	15.7 (13.4 - 18.1)	4.1 (3.7 - 4.5)	15.2 (13.8 - 16.6)
	L. monocytogenes	1.9 (1.3-2.6)	5.0 (3.6-7.0)	2.7 (2.0 - 3.5)	7.1 (5.5 - 8.9)	2.7 (2.3 - 3.0)	8.4 (7.6 - 9.2)
	N. meningitidis	6.4 (5.3-7.5)	5.5 (4.5-6.5)	8.4 (7.7 - 9.1)	6.9 (6.3 - 7.6)	8.8 (8.3 - 9.3)	6.9 (6.5 - 7.3)
	Other	1.9 (1.1-3.0)	2.4 (1.4-3.9)	11.1 (7.4 - 16.1)	11.8 (8.0 - 16.9)	11.1 (8.4 - 14.6)	13.9 (10.3 - 18.2)
	S. aureus	4.4 (3.4-5.5)	5.8 (4.6-7.3)	5.5 (4.9 - 6.1)	7.1 (6.3 - 7.9)	5.5 (5.1 - 5.9)	8.0 (7.4 - 8.5)

Location	Aetiology	Neonatal		Under 5		All Ages	
		Nonfatal	Fatal	Nonfatal	Fatal	Nonfatal	Fatal
	S. pneumoniae	2.7 (2.1-3.4)	2.1 (1.6-2.7)	13.1 (12.1 - 14.2)	8.7 (8.0 - 9.5)	13.3 (12.6 - 14.0)	11.7 (10.9 - 12.4)
	Virus	35.9 (32.8-39.1)	9.8 (8.6-11.1)	27.9 (26.1 - 29.5)	7.8 (7.3 - 8.4)	35.1 (33.6 - 36.4)	10.3 (9.7 - 10.8)
Israel	*E. coli*	6.6 (5.3-8.1)	13.1 (10.7-15.8)	6.8 (5.9 - 7.7)	13.7 (12.2 - 15.2)	5.7 (5.2 - 6.2)	13.0 (11.9 - 14.1)
	Group B *Streptococcus*	26.1 (23.1-29.1)	29.4 (25.9-33.1)	11.2 (10.2 - 12.1)	15.3 (14.1 - 16.6)	8.4 (7.9 - 8.9)	9.6 (9.1 - 10.2)
	H. influenzae	7.3 (5.6-9.3)	4.3 (3.2-5.7)	8.7 (7.9 - 9.6)	5.0 (4.5 - 5.6)	5.4 (5.0 - 5.8)	3.0 (2.7 - 3.2)
	K. pneumoniae	6.1 (4.7-7.8)	21.5 (17.2-26.2)	3.9 (3.2 - 4.7)	15.4 (13.2 - 17.7)	4.0 (3.6 - 4.5)	14.5 (13.2 - 15.9)
	L. monocytogenes	1.9 (1.3-2.7)	5.0 (3.5-6.9)	2.7 (2.0 - 3.4)	6.9 (5.4 - 8.6)	2.6 (2.2 - 2.9)	8.4 (7.6 - 9.1)
	N. meningitidis	6.5 (5.4-7.7)	5.6 (4.6-6.7)	9.1 (8.4 - 9.8)	7.5 (6.8 - 8.2)	9.6 (9.0 - 10.1)	7.1 (6.6 - 7.5)
	Other	2.0 (1.1-3.1)	2.5 (1.5-4.1)	10.9 (7.3 - 15.7)	11.8 (8.1 - 16.6)	10.9 (8.3 - 14.0)	13.3 (9.8 - 17.6)
	S. aureus	4.5 (3.5-5.6)	5.9 (4.7-7.4)	5.6 (5.0 - 6.2)	7.3 (6.5 - 8.1)	5.6 (5.2 - 6.0)	8.1 (7.6 - 8.7)
	S. pneumoniae	2.8 (2.1-3.5)	2.1 (1.6-2.7)	13.7 (12.6 - 14.8)	9.0 (8.2 - 9.8)	13.8 (13.1 - 14.6)	12.3 (11.5 - 13.1)
	Virus	36.3 (33.1-39.6)	10.5 (9.2-11.9)	27.5 (25.8 - 29.0)	8.2 (7.6 - 8.8)	34.0 (32.7 - 35.2)	10.7 (10.0 - 11.3)
Italy	*E. coli*	6.6 (5.3-8.2)	13.3 (10.7-16.1)	6.9 (6.1 - 7.9)	14.1 (12.6 - 15.9)	6.0 (5.5 - 6.5)	13.7 (12.4 - 15.1)
	Group B *Streptococcus*	26.8 (23.6-30.1)	30.2 (26.5-34.2)	11.4 (10.4 - 12.5)	15.6 (14.3 - 17.0)	7.8 (7.4 - 8.3)	9.0 (8.2 - 9.6)
	H. influenzae	7.0 (5.3-9.1)	4.1 (3.0-5.5)	8.7 (7.8 - 9.6)	4.9 (4.4 - 5.4)	4.4 (4.0 - 4.7)	2.7 (2.4 - 3.0)
	K. pneumoniae	6.2 (4.7-8.0)	21.9 (17.3-26.8)	3.9 (3.2 - 4.8)	15.6 (13.3 - 18.1)	4.3 (3.8 - 4.7)	14.5 (13.0 - 16.1)
	L. monocytogenes	1.9 (1.3-2.7)	5.0 (3.5-7.1)	2.7 (2.0 - 3.5)	7.2 (5.5 - 9.0)	3.0 (2.7 - 3.3)	9.3 (8.3 - 10.2)
	N. meningitidis	6.2 (5.1-7.4)	5.3 (4.3-6.5)	8.2 (7.5 - 8.9)	6.8 (6.2 - 7.5)	8.0 (7.6 - 8.5)	6.0 (5.6 - 6.5)
	Other	1.9 (1.1-3.1)	2.5 (1.4-4.0)	11.1 (7.4 - 16.0)	12.2 (8.2 - 17.5)	11.1 (8.3 - 14.6)	13.7 (9.5 - 18.9)
	S. aureus	4.5 (3.4-5.7)	5.9 (4.6-7.5)	5.6 (5.0 - 6.3)	7.3 (6.5 - 8.2)	5.9 (5.5 - 6.3)	8.3 (7.7 - 9.0)
	S. pneumoniae	2.7 (2.1-3.5)	1.9 (1.4-2.5)	13.6 (12.5 - 14.7)	8.4 (7.6 - 9.2)	14.3 (13.5 - 15.0)	12.2 (11.3 - 13.1)
	Virus	36.1 (32.8-39.4)	9.9 (8.6-11.3)	27.8 (26.0 - 29.4)	7.8 (7.3 - 8.4)	35.2 (33.8 - 36.5)	10.7 (9.9 - 11.4)
Luxembourg	*E. coli*	6.5 (5.0-8.2)	13.0 (10.2-16.1)	6.9 (6.0 - 7.9)	14.2 (12.5 - 16.0)	5.7 (5.1 - 6.2)	13.5 (12.1 - 14.8)
	Group B *Streptococcus*	25.9 (22.2-29.7)	29.3 (25.1-33.9)	11.6 (10.5 - 12.7)	15.9 (14.4 - 17.4)	8.4 (7.8 - 8.9)	9.7 (8.9 - 10.4)
	H. influenzae	7.2 (5.4-9.6)	4.3 (3.1-5.8)	8.7 (7.8 - 9.6)	5.0 (4.4 - 5.6)	5.1 (4.7 - 5.5)	2.9 (2.6 - 3.1)
	K. pneumoniae	6.4 (4.8-8.3)	22.6 (17.8-28.0)	3.9 (3.2 - 4.7)	15.9 (13.5 - 18.3)	4.1 (3.6 - 4.5)	14.9 (13.4 - 16.5)
	L. monocytogenes	1.9 (1.3-2.7)	5.1 (3.5-7.2)	2.7 (2.0 - 3.5)	7.2 (5.5 - 9.1)	2.7 (2.4 - 3.1)	9.0 (8.1 - 10.0)
	N. meningitidis	6.2 (4.9-7.5)	5.3 (4.2-6.6)	7.9 (7.2 - 8.7)	6.7 (6.1 - 7.4)	8.3 (7.8 - 8.8)	6.3 (5.8 - 6.8)
	Other	1.9 (1.1-3.2)	2.5 (1.4-4.1)	11.0 (7.3 - 15.9)	12.2 (8.2 - 17.5)	11.0 (8.4 - 14.4)	14.0 (10.2 - 18.7)
	S. aureus	4.6 (3.5-5.9)	6.1 (4.7-7.9)	5.7 (5.1 - 6.5)	7.6 (6.7 - 8.6)	5.8 (5.3 - 6.2)	8.6 (8.0 - 9.4)

Location	Aetiology	Neonatal		Under 5		All Ages	
		Nonfatal	Fatal	Nonfatal	Fatal	Nonfatal	Fatal
	S. pneumoniae	2.8 (2.0-3.7)	1.7 (1.2-2.3)	13.9 (12.7 - 15.1)	7.4 (6.6 - 8.2)	14.0 (13.1 - 14.9)	10.5 (9.6 - 11.3)
	Virus	36.5 (33.1-40.1)	10.1 (8.7-11.6)	27.7 (25.8 - 29.2)	8.0 (7.4 - 8.6)	35.1 (33.6 - 36.4)	10.7 (10.0 - 11.4)
Malta	*E. coli*	7.2 (6.2-8.3)	14.1 (12.2-16.1)	6.7 (5.9 - 7.5)	12.8 (11.5 - 14.1)	5.7 (5.3 - 6.2)	12.2 (11.2 - 13.3)
	Group B *Streptococcus*	29.6 (27.6-31.6)	32.7 (30.6-34.8)	10.4 (9.7 - 11.1)	14.6 (13.8 - 15.4)	7.4 (7.0 - 7.8)	8.1 (7.6 - 8.6)
	H. influenzae	6.5 (5.1-8.0)	3.8 (2.9-4.7)	7.9 (7.2 - 8.6)	4.2 (3.8 - 4.5)	4.8 (4.5 - 5.1)	2.6 (2.4 - 2.8)
	K. pneumoniae	5.4 (4.2-6.7)	18.5 (14.9-22.4)	4.0 (3.3 - 4.9)	14.3 (12.3 - 16.5)	4.2 (3.8 - 4.6)	13.7 (12.5 - 14.9)
	L. monocytogenes	1.8 (1.3-2.4)	4.7 (3.4-6.3)	2.6 (2.0 - 3.4)	6.3 (5.0 - 7.9)	2.7 (2.4 - 3.0)	7.9 (7.2 - 8.6)
	N. meningitidis	6.8 (5.9-7.8)	5.7 (4.8-6.5)	11.3 (10.6 - 12.1)	8.4 (7.8 - 9.1)	11.1 (10.6 - 11.6)	7.4 (7.0 - 7.8)
	Other	1.8 (1.0-2.9)	2.3 (1.3-3.7)	10.9 (7.4 - 15.5)	10.7 (7.3 - 14.8)	11.0 (8.4 - 14.2)	12.4 (9.0 - 16.6)
	S. aureus	3.9 (3.1-4.9)	5.1 (4.1-6.3)	4.8 (4.3 - 5.3)	5.8 (5.2 - 6.4)	5.0 (4.7 - 5.3)	6.7 (6.3 - 7.2)
	S. pneumoniae	2.6 (2.1-3.1)	3.6 (3.0-4.4)	13.6 (12.7 - 14.4)	15.4 (14.2 - 16.5)	12.9 (12.3 - 13.5)	19.0 (17.8 - 20.0)
	Virus	34.5 (31.7-37.4)	9.6 (8.5-10.7)	27.6 (26.1 - 29.0)	7.5 (7.0 - 7.9)	35.0 (33.7 - 36.2)	9.9 (9.3 - 10.4)
Monaco	*E. coli*	6.6 (5.2-8.2)	13.1 (10.5-16.1)	6.9 (6.1 - 7.9)	14.3 (12.6 - 16.2)	5.9 (5.5 - 6.5)	13.6 (12.3 - 15.0)
	Group B *Streptococcus*	26.5 (23.1-30.0)	29.9 (26.0-34.1)	11.5 (10.5 - 12.6)	14.3 (13.0 - 15.7)	8.2 (7.7 - 8.8)	9.1 (8.4 - 9.8)
	H. influenzae	7.1 (5.3-9.3)	4.2 (3.0-5.6)	8.6 (7.8 - 9.5)	5.0 (4.4 - 5.5)	4.8 (4.4 - 5.1)	2.7 (2.4 - 3.0)
	K. pneumoniae	6.3 (4.7-8.1)	22.2 (17.5-27.3)	3.9 (3.2 - 4.8)	15.1 (12.6 - 17.7)	4.2 (3.7 - 4.6)	14.6 (13.1 - 16.2)
	L. monocytogenes	1.9 (1.3-2.7)	5.1 (3.5-7.1)	2.7 (2.0 - 3.6)	7.4 (5.7 - 9.5)	2.9 (2.6 - 3.2)	9.2 (8.3 - 10.2)
	N. meningitidis	6.2 (5.0-7.5)	5.3 (4.2-6.5)	8.1 (7.4 - 8.8)	6.9 (6.2 - 7.7)	8.1 (7.6 - 8.6)	6.1 (5.6 - 6.5)
	Other	1.9 (1.1-3.1)	2.5 (1.4-4.0)	11.1 (7.3 - 16.0)	13.2 (8.8 - 19.0)	11.0 (8.4 - 14.6)	13.8 (9.8 - 18.9)
	S. aureus	4.5 (3.5-5.8)	6.0 (4.7-7.7)	5.6 (5.0 - 6.3)	7.6 (6.7 - 8.6)	5.9 (5.5 - 6.3)	8.5 (7.8 - 9.2)
	S. pneumoniae	2.8 (2.1-3.6)	1.8 (1.3-2.4)	13.7 (12.6 - 14.9)	8.5 (7.7 - 9.5)	14.3 (13.5 - 15.1)	11.6 (10.7 - 12.5)
	Virus	36.3 (33.0-39.7)	10.0 (8.7-11.4)	27.8 (25.9 - 29.3)	7.7 (7.1 - 8.3)	34.7 (33.3 - 36.0)	10.7 (10.0 - 11.4)
Netherlands	*E. coli*	6.5 (5.1-8.2)	13.1 (10.3-16.1)	6.9 (6.0 - 7.9)	14.1 (12.5 - 16.0)	5.9 (5.4 - 6.5)	13.9 (12.6 - 15.4)
	Group B *Streptococcus*	26.0 (22.4-29.7)	29.4 (25.3-33.9)	11.7 (10.7 - 12.8)	16.3 (14.8 - 17.8)	8.6 (8.0 - 9.2)	9.6 (8.9 - 10.4)
	H. influenzae	7.2 (5.4-9.5)	4.2 (3.1-5.7)	9.2 (8.3 - 10.1)	5.2 (4.6 - 5.8)	5.3 (4.9 - 5.7)	3.0 (2.7 - 3.3)
	K. pneumoniae	6.3 (4.8-8.2)	22.5 (17.8-27.8)	3.9 (3.2 - 4.7)	16.1 (13.7 - 18.6)	4.1 (3.7 - 4.6)	14.7 (13.2 - 16.4)
	L. monocytogenes	1.9 (1.3-2.7)	5.1 (3.6-7.2)	2.7 (2.0 - 3.5)	7.2 (5.5 - 9.0)	2.8 (2.5 - 3.2)	9.3 (8.3 - 10.3)
	N. meningitidis	6.3 (5.0-7.6)	5.4 (4.3-6.6)	7.9 (7.2 - 8.6)	6.6 (6.0 - 7.3)	8.0 (7.5 - 8.5)	5.9 (5.4 - 6.3)
	Other	1.9 (1.1-3.2)	2.5 (1.4-4.1)	10.9 (7.3 - 15.8)	11.9 (8.0 - 17.0)	11.0 (8.4 - 14.6)	13.7 (9.6 - 18.9)
	S. aureus	4.6 (3.5-5.9)	6.1 (4.7-7.8)	5.6 (5.0 - 6.3)	7.4 (6.6 - 8.4)	5.8 (5.4 - 6.3)	8.6 (7.9 - 9.3)

Location	Aetiology	Neonatal		Under 5		All Ages	
		Nonfatal	Fatal	Nonfatal	Fatal	Nonfatal	Fatal
	S. pneumoniae	2.8 (2.0-3.7)	1.7 (1.2-2.3)	13.4 (12.3 - 14.5)	7.2 (6.5 - 8.0)	13.9 (13.1 - 14.8)	10.7 (9.8 - 11.6)
	Virus	36.5 (33.1-39.9)	10.0 (8.7-11.4)	27.8 (26.0 - 29.3)	8.0 (7.4 - 8.6)	34.5 (33.1 - 35.8)	10.6 (9.9 - 11.3)
Norway	*E. coli*	6.5 (5.1-8.1)	13.1 (10.4-16.1)	6.9 (6.0 - 7.9)	14.2 (12.6 - 16.1)	5.5 (5.0 - 6.1)	13.6 (12.3 - 15.1)
	Group B *Streptococcus*	26.0 (22.6-29.6)	29.4 (25.5-33.8)	11.3 (10.3 - 12.4)	14.9 (13.6 - 16.3)	7.9 (7.4 - 8.5)	9.3 (8.6 - 10.0)
	H. influenzae	7.2 (5.5-9.4)	4.2 (3.1-5.7)	9.2 (8.2 - 10.1)	5.2 (4.7 - 5.8)	4.8 (4.4 - 5.2)	2.9 (2.6 - 3.2)
	K. pneumoniae	6.3 (4.8-8.2)	22.4 (17.7-27.5)	3.9 (3.2 - 4.7)	15.4 (13.0 - 18.0)	4.1 (3.6 - 4.6)	14.8 (13.3 - 16.5)
	L. monocytogenes	1.9 (1.3-2.7)	5.1 (3.6-7.2)	2.7 (2.0 - 3.6)	7.4 (5.7 - 9.4)	2.7 (2.3 - 3.0)	9.1 (8.2 - 10.1)
	N. meningitidis	6.4 (5.1-7.7)	5.4 (4.4-6.7)	7.9 (7.2 - 8.6)	6.8 (6.1 - 7.5)	8.4 (7.8 - 8.9)	6.2 (5.7 - 6.6)
	Other	1.9 (1.1-3.2)	2.5 (1.4-4.1)	11.1 (7.4 - 16.2)	12.8 (8.6 - 18.5)	11.1 (8.5 - 14.6)	14.0 (10.0 - 18.9)
	S. aureus	4.6 (3.5-5.9)	6.1 (4.7-7.8)	5.6 (5.0 - 6.4)	7.6 (6.7 - 8.5)	5.7 (5.3 - 6.1)	8.5 (7.8 - 9.3)
	S. pneumoniae	2.8 (2.1-3.7)	1.8 (1.3-2.4)	13.6 (12.5 - 14.7)	7.9 (7.1 - 8.7)	13.7 (12.9 - 14.5)	10.8 (9.9 - 11.6)
	Virus	36.4 (33.1-39.9)	10.0 (8.7-11.4)	27.7 (25.8 - 29.2)	7.8 (7.2 - 8.4)	36.2 (34.7 - 37.6)	10.7 (10.0 - 11.4)
Portugal	*E. coli*	7.0 (5.9-8.2)	13.8 (11.9-15.9)	6.9 (6.1 - 7.8)	13.5 (12.0 - 15.0)	5.6 (5.2 - 6.0)	12.3 (11.3 - 13.3)
	Group B *Streptococcus*	28.9 (26.8-31.1)	32.1 (29.8-34.6)	10.7 (9.8 - 11.6)	14.4 (13.4 - 15.5)	7.3 (6.9 - 7.6)	8.0 (7.5 - 8.4)
	H. influenzae	6.6 (5.1-8.4)	3.8 (2.9-4.9)	8.2 (7.4 - 9.0)	4.4 (4.0 - 4.9)	4.3 (4.0 - 4.6)	2.4 (2.2 - 2.6)
	K. pneumoniae	5.6 (4.4-7.0)	19.5 (15.8-23.4)	4.0 (3.3 - 4.8)	14.7 (12.6 - 16.9)	4.2 (3.8 - 4.6)	14.1 (12.9 - 15.3)
	L. monocytogenes	1.8 (1.3-2.5)	4.8 (3.4-6.5)	2.7 (2.0 - 3.5)	6.7 (5.3 - 8.5)	2.7 (2.5 - 3.0)	8.2 (7.5 - 8.9)
	N. meningitidis	6.5 (5.6-7.4)	5.5 (4.6-6.3)	10.1 (9.3 - 10.9)	7.8 (7.1 - 8.5)	10.4 (10.0 - 10.9)	7.3 (6.8 - 7.6)
	Other	1.8 (1.1-3.0)	2.3 (1.4-3.8)	11.2 (7.4 - 16.1)	11.6 (7.9 - 16.3)	11.0 (8.5 - 14.1)	13.0 (9.5 - 17.4)
	S. aureus	4.1 (3.3-5.0)	5.4 (4.3-6.6)	5.2 (4.7 - 5.8)	6.5 (5.8 - 7.2)	5.3 (5.0 - 5.6)	7.3 (6.8 - 7.8)
	S. pneumoniae	2.6 (2.2-3.1)	2.9 (2.4-3.5)	13.2 (12.2 - 14.2)	12.7 (11.6 - 13.8)	13.4 (12.9 - 13.9)	17.2 (16.1 - 18.0)
	Virus	35.0 (32.1-38.0)	9.8 (8.6-11.0)	27.9 (26.1 - 29.4)	7.7 (7.2 - 8.2)	35.8 (34.4 - 37.0)	10.3 (9.7 - 10.8)
San Marino	*E. coli*	7.1 (6.0-8.2)	14.0 (12.1-16.1)	6.9 (6.1 - 7.8)	13.3 (11.9 - 14.8)	5.8 (5.3 - 6.2)	12.8 (11.7 - 14.0)
	Group B *Streptococcus*	29.4 (27.4-31.3)	32.6 (30.4-34.8)	10.7 (9.8 - 11.5)	13.0 (12.1 - 14.0)	7.5 (7.1 - 7.9)	7.9 (7.4 - 8.4)
	H. influenzae	6.5 (5.1-8.2)	3.7 (2.9-4.8)	8.9 (8.1 - 9.7)	4.8 (4.3 - 5.2)	4.9 (4.6 - 5.3)	2.7 (2.4 - 2.9)
	K. pneumoniae	5.5 (4.3-6.8)	19.1 (15.4-23.1)	4.0 (3.3 - 4.9)	14.3 (12.2 - 16.7)	4.2 (3.8 - 4.6)	13.9 (12.6 - 15.2)
	L. monocytogenes	1.8 (1.3-2.5)	4.7 (3.4-6.4)	2.7 (2.0 - 3.5)	6.8 (5.3 - 8.6)	2.8 (2.5 - 3.1)	8.4 (7.7 - 9.2)
	N. meningitidis	6.7 (5.8-7.6)	5.6 (4.8-6.5)	10.1 (9.3 - 10.7)	8.0 (7.3 - 8.6)	10.2 (9.7 - 10.6)	6.9 (6.4 - 7.3)
	Other	1.8 (1.0-2.9)	2.3 (1.3-3.7)	11.1 (7.4 - 15.9)	12.1 (8.2 - 17.3)	11.1 (8.5 - 14.6)	12.9 (9.1 - 17.6)
	S. aureus	4.0 (3.2-4.9)	5.2 (4.2-6.5)	5.0 (4.4 - 5.5)	6.2 (5.6 - 6.9)	5.1 (4.8 - 5.5)	7.0 (6.5 - 7.5)

Location	Aetiology	Neonatal		Under 5		All Ages	
		Nonfatal	Fatal	Nonfatal	Fatal	Nonfatal	Fatal
	S. pneumoniae	2.6 (2.1-3.1)	3.2 (2.7-3.9)	12.7 (11.8 - 13.6)	14.2 (13.0 - 15.3)	12.7 (12.2 - 13.3)	17.5 (16.4 - 18.5)
	Virus	34.7 (31.8-37.6)	9.4 (8.3-10.5)	28.0 (26.2 - 29.5)	7.3 (6.7 - 7.8)	35.6 (34.2 - 36.9)	10.0 (9.4 - 10.5)
Spain	*E. coli*	7.1 (6.0-8.2)	14.0 (12.0-16.1)	7.0 (6.2 - 8.0)	13.6 (12.1 - 15.2)	5.8 (5.4 - 6.3)	12.8 (11.6 - 13.9)
	Group B *Streptococcus*	29.7 (27.6-31.8)	32.9 (30.6-35.3)	10.9 (9.9 - 11.8)	13.9 (12.9 - 15.0)	7.6 (7.2 - 8.0)	8.0 (7.5 - 8.5)
	H. influenzae	6.4 (5.0-8.1)	3.7 (2.8-4.7)	8.5 (7.7 - 9.4)	4.5 (4.1 - 5.0)	4.7 (4.3 - 5.0)	2.5 (2.3 - 2.7)
	K. pneumoniae	5.6 (4.3-6.9)	19.4 (15.6-23.4)	4.0 (3.3 - 4.9)	14.6 (12.4 - 17.0)	4.2 (3.9 - 4.6)	14.0 (12.8 - 15.4)
	L. monocytogenes	1.8 (1.3-2.5)	4.7 (3.4-6.4)	2.7 (2.1 - 3.6)	6.9 (5.4 - 8.7)	2.8 (2.5 - 3.1)	8.5 (7.7 - 9.3)
	N. meningitidis	6.4 (5.5-7.4)	5.4 (4.5-6.2)	9.6 (8.9 - 10.4)	7.6 (6.9 - 8.3)	9.9 (9.4 - 10.3)	6.8 (6.3 - 7.2)
	Other	1.8 (1.0-2.9)	2.3 (1.3-3.7)	11.2 (7.4 - 16.2)	12.0 (8.1 - 17.2)	11.2 (8.5 - 14.5)	13.0 (9.3 - 17.7)
	S. aureus	4.0 (3.2-5.0)	5.3 (4.3-6.6)	5.1 (4.5 - 5.7)	6.4 (5.7 - 7.1)	5.2 (4.9 - 5.6)	7.2 (6.6 - 7.7)
	S. pneumoniae	2.6 (2.1-3.1)	3.0 (2.5-3.6)	12.8 (11.8 - 13.7)	13.1 (12.0 - 14.3)	13.1 (12.5 - 13.6)	17.3 (16.1 - 18.2)
	Virus	34.6 (31.7-37.7)	9.4 (8.3-10.5)	28.1 (26.3 - 29.7)	7.4 (6.8 - 7.9)	35.5 (34.1 - 36.7)	10.0 (9.4 - 10.6)
Sweden	*E. coli*	6.7 (5.3-8.2)	13.3 (10.8-16.0)	6.9 (6.1 - 7.9)	14.1 (12.5 - 15.8)	5.8 (5.3 - 6.3)	13.8 (12.5 - 15.3)
	Group B *Streptococcus*	27.0 (23.9-30.1)	30.3 (26.8-34.1)	11.2 (10.2 - 12.3)	15.5 (14.3 - 16.9)	8.0 (7.5 - 8.5)	9.0 (8.3 - 9.6)
	H. influenzae	7.0 (5.3-9.1)	4.1 (3.0-5.4)	8.8 (7.9 - 9.6)	4.9 (4.4 - 5.5)	4.8 (4.4 - 5.2)	2.7 (2.5 - 3.0)
	K. pneumoniae	6.1 (4.7-7.9)	21.7 (17.2-26.5)	3.9 (3.2 - 4.8)	15.6 (13.3 - 18.0)	4.1 (3.7 - 4.6)	14.3 (12.8 - 16.0)
	L. monocytogenes	1.9 (1.3-2.7)	5.0 (3.5-7.0)	2.7 (2.0 - 3.6)	7.2 (5.5 - 9.0)	2.8 (2.4 - 3.1)	9.3 (8.3 - 10.3)
	N. meningitidis	6.3 (5.2-7.4)	5.4 (4.3-6.4)	8.3 (7.6 - 9.0)	6.9 (6.3 - 7.6)	8.7 (8.2 - 9.1)	6.0 (5.5 - 6.4)
	Other	1.9 (1.1-3.1)	2.5 (1.4-3.9)	11.2 (7.4 - 16.2)	12.1 (8.2 - 17.3)	11.1 (8.4 - 14.5)	13.4 (9.2 - 18.8)
	S. aureus	4.4 (3.4-5.6)	5.9 (4.6-7.4)	5.6 (4.9 - 6.2)	7.2 (6.4 - 8.1)	5.6 (5.2 - 6.0)	8.2 (7.6 - 8.9)
	S. pneumoniae	2.7 (2.1-3.5)	2.0 (1.5-2.5)	13.6 (12.5 - 14.7)	8.6 (7.9 - 9.5)	13.8 (13.0 - 14.5)	12.6 (11.6 - 13.5)
	Virus	36.0 (32.8-39.3)	9.9 (8.6-11.2)	27.8 (25.9 - 29.3)	7.8 (7.3 - 8.4)	35.4 (33.9 - 36.7)	10.6 (9.9 - 11.3)
Switzerland	*E. coli*	6.8 (5.5-8.1)	13.5 (11.2-16.0)	6.9 (6.1 - 7.9)	14.0 (12.5 - 15.6)	6.0 (5.5 - 6.5)	13.9 (12.4 - 15.4)
	Group B *Streptococcus*	27.6 (25.0-30.4)	31.0 (27.7-34.3)	11.3 (10.4 - 12.3)	15.7 (14.5 - 17.0)	8.3 (7.8 - 8.8)	8.5 (7.9 - 9.2)
	H. influenzae	6.8 (5.3-8.8)	4.0 (3.0-5.2)	8.7 (7.9 - 9.5)	4.8 (4.3 - 5.3)	5.1 (4.8 - 5.5)	2.7 (2.4 - 2.9)
	K. pneumoniae	6.0 (4.6-7.5)	21.1 (16.8-25.6)	4.0 (3.3 - 4.8)	15.6 (13.3 - 17.9)	4.2 (3.8 - 4.6)	14.0 (12.5 - 15.7)
	L. monocytogenes	1.9 (1.3-2.6)	5.0 (3.5-6.9)	2.7 (2.1 - 3.5)	7.0 (5.5 - 8.9)	2.8 (2.5 - 3.2)	9.3 (8.4 - 10.4)
	N. meningitidis	6.4 (5.3-7.4)	5.4 (4.5-6.4)	8.6 (8.0 - 9.3)	7.0 (6.4 - 7.7)	8.8 (8.3 - 9.2)	5.9 (5.5 - 6.3)
	Other	1.9 (1.1-3.0)	2.4 (1.4-3.9)	11.1 (7.4 - 16.1)	11.8 (8.0 - 16.8)	11.1 (8.4 - 14.6)	13.2 (8.8 - 18.8)
	S. aureus	4.3 (3.4-5.4)	5.7 (4.6-7.1)	5.4 (4.8 - 6.1)	7.0 (6.2 - 7.8)	5.5 (5.2 - 5.9)	8.0 (7.3 - 8.6)

Location	Aetiology	Neonatal		Under 5		All Ages	
		Nonfatal	Fatal	Nonfatal	Fatal	Nonfatal	Fatal
	S. pneumoniae	2.7 (2.1-3.3)	2.2 (1.8-2.8)	13.2 (12.3 - 14.2)	9.4 (8.6 - 10.3)	13.6 (12.9 - 14.2)	13.9 (12.8 - 14.9)
	Virus	35.7 (32.6-38.9)	9.7 (8.5-11.0)	28.0 (26.1 - 29.5)	7.8 (7.2 - 8.4)	34.6 (33.2 - 35.8)	10.6 (9.8 - 11.3)
United Kingdom	*E. coli*	6.5 (5.1-8.1)	13.0 (10.5-15.9)	6.8 (6.0 - 7.8)	14.0 (12.4 - 15.7)	5.6 (5.2 - 6.2)	13.0 (11.9 - 14.1)
	Group B *Streptococcus*	25.8 (22.4-29.2)	29.2 (25.4-33.3)	11.4 (10.4 - 12.4)	14.9 (13.6 - 16.3)	8.1 (7.6 - 8.7)	9.7 (9.1 - 10.3)
	H. influenzae	7.3 (5.5-9.5)	4.3 (3.2-5.7)	9.0 (8.1 - 9.9)	5.2 (4.6 - 5.8)	5.0 (4.6 - 5.4)	3.0 (2.7 - 3.2)
	K. pneumoniae	6.2 (4.8-8.0)	22.1 (17.5-27.1)	3.9 (3.2 - 4.7)	15.4 (13.0 - 17.8)	4.1 (3.7 - 4.5)	15.0 (13.6 - 16.4)
	L. monocytogenes	1.9 (1.3-2.7)	5.1 (3.6-7.1)	2.7 (2.0 - 3.5)	7.2 (5.5 - 9.0)	2.7 (2.4 - 3.0)	8.5 (7.7 - 9.3)
	N. meningitidis	6.4 (5.2-7.7)	5.5 (4.4-6.7)	8.5 (7.7 - 9.2)	7.2 (6.5 - 7.9)	8.8 (8.3 - 9.3)	7.0 (6.5 - 7.4)
	Other	2.0 (1.1-3.2)	2.5 (1.4-4.1)	10.9 (7.3 - 15.8)	12.5 (8.5 - 17.8)	11.0 (8.4 - 14.2)	14.0 (10.4 - 18.3)
	S. aureus	4.6 (3.5-5.7)	6.1 (4.7-7.6)	5.6 (5.0 - 6.3)	7.5 (6.7 - 8.4)	5.7 (5.3 - 6.1)	8.4 (7.8 - 9.0)
	S. pneumoniae	2.8 (2.1-3.7)	1.9 (1.4-2.5)	13.6 (12.5 - 14.7)	8.3 (7.6 - 9.1)	13.9 (13.1 - 14.7)	11.0 (10.2 - 11.7)
	Virus	36.5 (33.2-39.9)	10.3 (9.0-11.7)	27.6 (25.8 - 29.1)	8.0 (7.4 - 8.6)	35.1 (33.7 - 36.3)	10.6 (10.0 - 11.2)
**Latin America and Caribbean**	*E. coli*	6.5 (5.3-7.8)	12.3 (10.0-15.0)	5.9 (5.0 - 6.8)	10.7 (9.3 - 12.3)	4.8 (4.3 - 5.4)	10.1 (9.0 - 11.2)
	Group B *Streptococcus*	23.5 (21.3-26.1)	24.6 (22.1-27.4)	9.8 (9.2 - 10.5)	11.4 (10.5 - 12.3)	7.0 (6.6 - 7.4)	8.7 (8.1 - 9.2)
	H. influenzae	8.3 (6.8-9.9)	5.9 (4.9-7.0)	8.5 (7.9 - 9.1)	5.3 (5.0 - 5.7)	4.6 (4.4 - 4.9)	3.4 (3.2 - 3.6)
	K. pneumoniae	5.7 (4.5-7.2)	17.4 (13.9-21.4)	3.8 (3.1 - 4.6)	12.4 (10.3 - 14.9)	3.9 (3.4 - 4.4)	13.0 (11.4 - 14.6)
	L. monocytogenes	1.9 (1.3-2.7)	4.6 (3.2-6.5)	2.3 (1.7 - 2.9)	5.2 (4.1 - 6.6)	2.2 (2.0 - 2.5)	5.9 (5.2 - 6.7)
	N. meningitidis	8.1 (7.1-9.2)	7.9 (6.8-9.3)	14.8 (13.9 - 15.7)	13.4 (12.3 - 14.5)	14.8 (14.2 - 15.3)	12.6 (12.0 - 13.2)
	Other	2.2 (1.3-3.4)	2.8 (1.8-4.3)	9.8 (6.9 - 13.5)	10.1 (7.2 - 13.7)	10.0 (8.2 - 12.0)	11.8 (9.6 - 14.2)
	S. aureus	4.3 (3.4-5.4)	5.5 (4.3-7.0)	5.2 (4.7 - 5.8)	6.3 (5.6 - 6.9)	5.3 (5.0 - 5.7)	7.1 (6.6 - 7.6)
	S. pneumoniae	2.9 (2.4-3.4)	4.5 (3.8-5.4)	14.1 (13.3 - 15.0)	16.0 (14.8 - 17.3)	14.3 (13.8 - 14.9)	16.5 (15.7 - 17.4)
	Virus	36.7 (33.8-39.7)	14.3 (12.7-16.0)	25.8 (24.5 - 26.9)	9.2 (8.7 - 9.8)	33.0 (32.0 - 33.9)	11.0 (10.6 - 11.5)
**Andean Latin America**	*E. coli*	6.5 (5.2-7.8)	12.6 (10.3-15.2)	6.0 (5.1 - 7.0)	11.5 (10.0 - 13.2)	5.2 (4.6 - 5.9)	10.2 (9.0 - 11.3)
	Group B *Streptococcus*	23.7 (21.1-26.4)	26.7 (23.7-29.8)	10.2 (9.4 - 10.9)	12.7 (11.7 - 13.7)	8.0 (7.5 - 8.4)	8.7 (8.1 - 9.2)
	H. influenzae	8.2 (6.5-9.9)	5.3 (4.1-6.5)	8.6 (8.0 - 9.2)	5.0 (4.6 - 5.5)	5.9 (5.6 - 6.3)	3.1 (2.9 - 3.3)
	K. pneumoniae	5.9 (4.6-7.4)	19.2 (15.6-23.5)	3.8 (3.1 - 4.7)	13.3 (11.2 - 15.7)	3.9 (3.3 - 4.5)	13.2 (11.6 - 14.8)
	L. monocytogenes	1.9 (1.3-2.7)	4.8 (3.3-6.8)	2.3 (1.8 - 2.9)	5.6 (4.4 - 7.1)	2.3 (1.9 - 2.6)	6.1 (5.4 - 6.9)
	N. meningitidis	7.6 (6.5-8.7)	6.5 (5.5-7.6)	14.0 (13.0 - 14.9)	11.6 (10.6 - 12.6)	14.4 (13.8 - 15.0)	12.1 (11.5 - 12.8)
	Other	2.2 (1.3-3.4)	2.8 (1.7-4.4)	9.8 (6.8 - 13.6)	10.5 (7.5 - 14.5)	9.9 (7.9 - 12.3)	12.1 (9.9 - 14.5)
	S. aureus	4.4 (3.5-5.5)	5.7 (4.5-7.1)	5.4 (4.8 - 6.0)	6.8 (6.0 - 7.5)	5.4 (5.0 - 5.8)	7.4 (6.8 - 8.0)

Location	Aetiology	Neonatal		Under 5		All Ages	
		Nonfatal	Fatal	Nonfatal	Fatal	Nonfatal	Fatal
	S. pneumoniae	2.9 (2.3-3.4)	3.2 (2.6-3.8)	13.9 (13.0 - 14.8)	13.7 (12.6 - 14.9)	14.0 (13.4 - 14.6)	15.7 (14.9 - 16.6)
	Virus	36.9 (33.9-40.0)	13.2 (11.7-14.9)	26.1 (24.8 - 27.3)	9.3 (8.7 - 9.9)	31.0 (30.0 - 31.9)	11.5 (11.0 - 11.9)
Bolivia (Plurinational State of)	*E. coli*	6.5 (5.2-8.1)	12.5 (10.1-15.2)	5.8 (4.8 - 6.9)	11.0 (9.4 - 12.8)	5.1 (4.4 - 6.0)	9.8 (8.4 - 11.1)
	Group B *Streptococcus*	23.9 (21.1-27.0)	26.7 (23.4-29.9)	9.9 (9.1 - 10.7)	12.1 (11.1 - 13.1)	8.0 (7.4 - 8.6)	8.7 (8.1 - 9.4)
	H. influenzae	8.2 (6.5-10.1)	5.3 (4.1-6.6)	7.3 (6.8 - 7.9)	4.6 (4.2 - 5.0)	5.4 (5.0 - 5.8)	3.0 (2.8 - 3.2)
	K. pneumoniae	5.8 (4.5-7.4)	18.6 (14.8-23.1)	3.8 (3.1 - 4.7)	12.7 (10.5 - 15.3)	3.8 (3.1 - 4.6)	12.4 (10.6 - 14.3)
	L. monocytogenes	1.9 (1.3-2.7)	4.7 (3.2-6.7)	2.2 (1.7 - 2.9)	5.3 (4.2 - 6.8)	2.1 (1.8 - 2.6)	5.6 (4.8 - 6.5)
	N. meningitidis	7.5 (6.4-8.8)	6.4 (5.4-7.6)	16.1 (15.0 - 17.2)	12.6 (11.6 - 13.6)	16.8 (16.1 - 17.6)	13.3 (12.6 - 14.0)
	Other	2.2 (1.4-3.5)	2.9 (1.8-4.4)	9.5 (6.7 - 13.2)	10.2 (7.3 - 14.1)	9.5 (7.5 - 12.0)	11.1 (9.1 - 13.5)
	S. aureus	4.3 (3.3-5.5)	5.6 (4.3-7.1)	5.3 (4.7 - 6.0)	6.6 (5.8 - 7.4)	5.3 (4.8 - 5.8)	7.0 (6.4 - 7.7)
	S. pneumoniae	2.8 (2.3-3.4)	3.5 (2.8-4.2)	14.4 (13.4 - 15.5)	15.5 (14.4 - 16.8)	14.7 (13.9 - 15.4)	17.9 (17.0 - 19.0)
	Virus	36.9 (33.6-40.0)	13.9 (12.2-15.6)	25.6 (24.3 - 26.8)	9.4 (8.8 - 10.0)	29.2 (28.2 - 30.2)	11.2 (10.7 - 11.7)
Ecuador	*E. coli*	6.5 (5.2-7.8)	12.6 (10.3-15.2)	6.0 (5.2 - 6.9)	11.8 (10.4 - 13.4)	5.3 (4.6 - 5.9)	10.4 (9.3 - 11.4)
	Group B *Streptococcus*	23.9 (21.3-26.7)	27.0 (24.0-30.1)	10.4 (9.7 - 11.2)	13.6 (12.6 - 14.6)	8.2 (7.7 - 8.6)	8.8 (8.3 - 9.3)
	H. influenzae	8.1 (6.4-9.8)	5.1 (4.0-6.4)	8.6 (8.0 - 9.2)	5.2 (4.8 - 5.7)	5.9 (5.5 - 6.2)	3.0 (2.8 - 3.2)
	K. pneumoniae	5.9 (4.7-7.5)	19.6 (16.0-23.8)	3.9 (3.2 - 4.7)	13.9 (11.8 - 16.2)	3.9 (3.4 - 4.5)	13.4 (11.8 - 15.0)
	L. monocytogenes	1.9 (1.3-2.7)	4.8 (3.4-6.9)	2.3 (1.8 - 2.9)	5.8 (4.6 - 7.2)	2.3 (2.0 - 2.7)	6.3 (5.6 - 7.0)
	N. meningitidis	7.4 (6.3-8.5)	6.4 (5.3-7.4)	13.6 (12.7 - 14.4)	10.7 (9.9 - 11.6)	14.1 (13.5 - 14.7)	11.7 (11.1 - 12.3)
	Other	2.2 (1.3-3.4)	2.8 (1.7-4.3)	9.7 (6.8 - 13.5)	10.5 (7.4 - 14.3)	9.9 (7.9 - 12.2)	12.2 (10.0 - 14.7)
	S. aureus	4.4 (3.5-5.5)	5.8 (4.6-7.2)	5.4 (4.8 - 5.9)	6.8 (6.1 - 7.5)	5.4 (5.0 - 5.8)	7.5 (7.0 - 8.1)
	S. pneumoniae	2.9 (2.3-3.4)	2.9 (2.4-3.6)	13.9 (13.0 - 14.8)	12.3 (11.4 - 13.3)	14.1 (13.5 - 14.8)	15.2 (14.4 - 16.0)
	Virus	36.9 (33.8-39.9)	12.9 (11.4-14.4)	26.3 (25.0 - 27.5)	9.3 (8.7 - 9.9)	31.0 (30.0 - 31.9)	11.6 (11.1 - 12.0)
Peru	*E. coli*	6.4 (5.2-7.7)	12.6 (10.4-15.0)	6.1 (5.2 - 7.0)	12.1 (10.6 - 13.7)	5.2 (4.7 - 5.8)	10.4 (9.4 - 11.5)
	Group B *Streptococcus*	23.3 (20.8-26.0)	26.4 (23.6-29.4)	10.2 (9.4 - 10.9)	13.0 (12.0 - 14.0)	7.8 (7.4 - 8.3)	8.5 (8.0 - 9.0)
	H. influenzae	8.3 (6.7-10.0)	5.2 (4.1-6.5)	9.4 (8.7 - 10.1)	5.7 (5.2 - 6.2)	6.2 (5.9 - 6.6)	3.2 (3.0 - 3.4)
	K. pneumoniae	5.9 (4.7-7.4)	19.9 (16.3-23.8)	3.9 (3.2 - 4.7)	13.9 (11.8 - 16.3)	3.9 (3.4 - 4.5)	13.8 (12.3 - 15.2)
	L. monocytogenes	1.9 (1.3-2.7)	4.9 (3.5-6.8)	2.4 (1.8 - 3.0)	6.0 (4.8 - 7.5)	2.3 (2.0 - 2.7)	6.4 (5.8 - 7.1)
	N. meningitidis	7.7 (6.6-8.9)	6.7 (5.6-7.7)	12.9 (12.0 - 13.7)	10.4 (9.6 - 11.3)	13.4 (12.9 - 14.0)	11.4 (10.8 - 12.0)
	Other	2.1 (1.3-3.3)	2.8 (1.7-4.3)	10.0 (6.9 - 13.9)	11.1 (7.8 - 15.2)	10.1 (8.1 - 12.5)	12.8 (10.5 - 15.4)
	S. aureus	4.4 (3.6-5.5)	5.9 (4.8-7.3)	5.4 (4.9 - 6.0)	7.0 (6.2 - 7.7)	5.4 (5.1 - 5.8)	7.6 (7.2 - 8.2)

Location	Aetiology	Neonatal		Under 5		All Ages	
		Nonfatal	Fatal	Nonfatal	Fatal	Nonfatal	Fatal
	S. pneumoniae	2.9 (2.3-3.5)	2.9 (2.3-3.5)	13.5 (12.6 - 14.5)	11.8 (10.9 - 12.8)	13.6 (13.0 - 14.2)	14.1 (13.4 - 14.8)
	Virus	37.0 (34.0-40.0)	12.6 (11.2-14.2)	26.4 (25.0 - 27.6)	9.0 (8.5 - 9.6)	31.9 (30.9 - 32.9)	11.7 (11.2 - 12.2)
**Caribbean**	*E. coli*	6.5 (5.1-8.1)	12.2 (9.5-15.1)	5.3 (4.4 - 6.3)	9.4 (8.0 - 11.1)	4.8 (4.2 - 5.6)	9.2 (7.9 - 10.6)
	Group B *Streptococcus*	21.7 (19.5-24.3)	23.4 (20.7-26.3)	8.8 (8.2 - 9.5)	10.9 (10.0 - 11.9)	7.2 (6.7 - 7.6)	9.2 (8.5 - 9.9)
	H. influenzae	9.0 (7.8-10.5)	6.3 (5.2-7.5)	9.0 (8.4 - 9.5)	5.7 (5.3 - 6.1)	6.3 (6.0 - 6.7)	4.6 (4.3 - 4.9)
	K. pneumoniae	5.3 (4.0-6.8)	16.4 (12.8-20.6)	3.7 (2.9 - 4.7)	11.6 (9.5 - 14.1)	3.8 (3.2 - 4.5)	11.8 (10.0 - 13.9)
	L. monocytogenes	1.9 (1.3-2.6)	4.6 (3.1-6.4)	2.0 (1.5 - 2.6)	4.4 (3.4 - 5.6)	2.1 (1.8 - 2.5)	4.8 (4.0 - 5.8)
	N. meningitidis	9.7 (8.5-11.2)	8.6 (7.4-10.2)	20.0 (19.0 - 21.0)	15.9 (14.9 - 17.0)	18.9 (18.2 - 19.6)	15.5 (14.6 - 16.3)
	Other	2.2 (1.4-3.4)	2.9 (1.8-4.3)	8.8 (6.2 - 12.2)	8.4 (6.0 - 11.5)	9.3 (7.6 - 11.4)	9.4 (7.6 - 11.7)
	S. aureus	4.1 (3.2-5.3)	5.4 (4.1-7.0)	4.6 (4.1 - 5.1)	5.4 (4.8 - 6.1)	4.8 (4.5 - 5.2)	5.8 (5.3 - 6.4)
	S. pneumoniae	3.0 (2.5-3.5)	5.2 (4.3-6.2)	13.2 (12.5 - 13.9)	18.4 (17.3 - 19.6)	13.1 (12.6 - 13.6)	19.1 (18.2 - 20.2)
	Virus	36.6 (33.8-39.7)	15.1 (13.4-16.9)	24.5 (23.5 - 25.7)	9.8 (9.2 - 10.4)	29.6 (28.7 - 30.4)	10.5 (10.1 - 11.0)
Antigua and Barbuda	*E. coli*	7.1 (5.9-8.4)	13.6 (11.5-15.8)	5.9 (5.0 - 6.8)	11.0 (9.8 - 12.3)	5.0 (4.5 - 5.5)	10.0 (9.1 - 11.0)
	Group B *Streptococcus*	27.8 (25.5-30.2)	30.6 (27.9-33.2)	9.8 (9.1 - 10.4)	13.4 (12.6 - 14.2)	6.8 (6.5 - 7.2)	7.7 (7.3 - 8.1)
	H. influenzae	7.0 (5.6-8.7)	4.3 (3.4-5.5)	6.5 (5.9 - 7.0)	3.7 (3.4 - 4.0)	3.9 (3.7 - 4.2)	2.3 (2.1 - 2.5)
	K. pneumoniae	5.4 (4.2-6.9)	17.7 (14.3-21.8)	3.8 (3.1 - 4.7)	12.9 (11.0 - 14.9)	4.0 (3.5 - 4.5)	12.6 (11.3 - 13.8)
	L. monocytogenes	1.8 (1.3-2.5)	4.5 (3.2-6.3)	2.3 (1.7 - 2.9)	5.3 (4.2 - 6.5)	2.3 (2.1 - 2.6)	6.2 (5.6 - 6.9)
	N. meningitidis	7.1 (6.2-8.2)	6.0 (5.1-7.0)	16.2 (15.2 - 17.0)	11.4 (10.7 - 12.1)	15.8 (15.2 - 16.4)	10.9 (10.4 - 11.4)
	Other	2.0 (1.2-3.1)	2.5 (1.5-3.9)	9.7 (6.9 - 13.6)	9.3 (6.7 - 12.9)	10.1 (8.3 - 12.2)	11.3 (9.1 - 13.9)
	S. aureus	4.0 (3.2-4.9)	5.1 (4.1-6.3)	4.7 (4.2 - 5.1)	5.5 (5.0 - 6.1)	4.9 (4.6 - 5.2)	6.4 (6.0 - 6.9)
	S. pneumoniae	2.6 (2.2-3.1)	4.1 (3.5-5.0)	15.6 (14.6 - 16.5)	19.4 (18.0 - 20.7)	14.3 (13.7 - 14.9)	22.1 (21.1 - 23.1)
	Virus	35.2 (32.3-38.2)	11.5 (10.2-12.9)	25.6 (24.4 - 26.8)	8.2 (7.7 - 8.6)	32.8 (31.9 - 33.7)	10.4 (9.9 - 10.8)
Bahamas	*E. coli*	6.6 (5.4-7.9)	12.8 (10.6-15.3)	6.1 (5.2 - 7.1)	11.9 (10.4 - 13.5)	4.9 (4.3 - 5.5)	10.0 (8.9 - 11.1)
	Group B *Streptococcus*	24.7 (22.2-27.5)	27.7 (24.8-30.6)	10.1 (9.3 - 10.9)	13.8 (12.8 - 14.9)	7.1 (6.7 - 7.5)	8.0 (7.6 - 8.5)
	H. influenzae	7.8 (6.2-9.6)	5.0 (3.9-6.2)	7.9 (7.3 - 8.5)	4.8 (4.4 - 5.2)	4.5 (4.2 - 4.8)	2.5 (2.4 - 2.7)
	K. pneumoniae	5.8 (4.6-7.3)	19.3 (15.7-23.4)	3.8 (3.1 - 4.7)	13.8 (11.8 - 16.2)	3.9 (3.4 - 4.5)	13.3 (11.7 - 14.8)
	L. monocytogenes	1.9 (1.3-2.7)	4.8 (3.3-6.8)	2.3 (1.8 - 3.0)	5.7 (4.6 - 7.1)	2.3 (2.0 - 2.6)	6.3 (5.6 - 7.0)
	N. meningitidis	7.3 (6.2-8.4)	6.2 (5.2-7.2)	13.9 (13.0 - 14.9)	10.6 (9.9 - 11.5)	14.8 (14.2 - 15.3)	11.7 (11.1 - 12.3)
	Other	2.1 (1.3-3.3)	2.7 (1.7-4.2)	10.0 (7.0 - 13.9)	10.2 (7.3 - 14.0)	10.0 (8.2 - 12.1)	12.3 (10.1 - 14.8)
	S. aureus	4.3 (3.4-5.4)	5.7 (4.5-7.0)	5.4 (4.8 - 6.0)	6.7 (6.0 - 7.4)	5.4 (5.0 - 5.7)	7.4 (6.9 - 8.0)

Location	Aetiology	Neonatal		Under 5		All Ages	
		Nonfatal	Fatal	Nonfatal	Fatal	Nonfatal	Fatal
	S. pneumoniae	2.8 (2.3-3.3)	3.1 (2.6-3.8)	14.3 (13.3 - 15.3)	13.1 (12.2 - 14.2)	14.4 (13.9 - 15.0)	17.0 (16.2 - 17.8)
	Virus	36.6 (33.6-39.7)	12.7 (11.2-14.2)	26.2 (24.8 - 27.4)	9.2 (8.7 - 9.8)	32.7 (31.8 - 33.7)	11.5 (11.0 - 12.0)
Barbados	*E. coli*	6.5 (5.3-7.8)	12.8 (10.5-15.1)	6.2 (5.4 - 7.2)	12.4 (11.1 - 13.9)	5.3 (4.8 - 5.8)	11.2 (10.2 - 12.4)
	Group B *Streptococcus*	24.2 (21.6-27.1)	27.4 (24.4-30.6)	10.5 (9.7 - 11.3)	15.9 (14.7 - 17.1)	7.6 (7.2 - 8.0)	8.4 (7.9 - 8.8)
	H. influenzae	7.9 (6.3-9.7)	5.0 (3.8-6.3)	8.6 (8.0 - 9.3)	5.1 (4.7 - 5.7)	5.1 (4.8 - 5.4)	2.8 (2.6 - 3.0)
	K. pneumoniae	6.0 (4.7-7.5)	20.2 (16.4-24.3)	3.9 (3.2 - 4.7)	15.2 (13.1 - 17.4)	4.0 (3.6 - 4.5)	13.5 (12.2 - 14.8)
	L. monocytogenes	1.9 (1.3-2.7)	4.9 (3.5-6.9)	2.4 (1.8 - 3.1)	5.9 (4.8 - 7.3)	2.5 (2.2 - 2.8)	7.2 (6.5 - 8.0)
	N. meningitidis	7.3 (6.2-8.4)	6.3 (5.2-7.3)	12.2 (11.4 - 13.0)	9.3 (8.5 - 10.0)	12.5 (12.0 - 13.0)	9.4 (8.9 - 9.9)
	Other	2.1 (1.3-3.3)	2.7 (1.7-4.2)	10.1 (7.0 - 14.1)	9.7 (6.9 - 13.2)	10.3 (8.3 - 12.6)	12.5 (9.8 - 15.8)
	S. aureus	4.4 (3.5-5.5)	5.9 (4.7-7.3)	5.5 (4.9 - 6.0)	6.8 (6.2 - 7.6)	5.6 (5.3 - 6.0)	7.9 (7.4 - 8.5)
	S. pneumoniae	2.8 (2.3-3.5)	2.7 (2.2-3.3)	13.8 (12.9 - 14.8)	10.1 (9.4 - 11.0)	14.3 (13.7 - 14.9)	15.4 (14.6 - 16.3)
	Virus	36.8 (33.8-40.0)	12.3 (10.8-13.8)	26.7 (25.3 - 27.9)	9.5 (8.9 - 10.1)	32.8 (31.8 - 33.7)	11.7 (11.1 - 12.2)
Belize	*E. coli*	6.9 (5.7-8.3)	13.2 (10.9-15.7)	5.6 (4.7 - 6.5)	10.6 (9.4 - 12.0)	4.7 (4.1 - 5.3)	9.3 (8.3 - 10.3)
	Group B *Streptococcus*	25.7 (23.3-28.2)	28.4 (25.7-31.0)	9.2 (8.6 - 9.9)	13.5 (12.6 - 14.4)	6.8 (6.4 - 7.2)	8.6 (8.1 - 9.0)
	H. influenzae	7.7 (6.3-9.3)	4.8 (3.9-6.0)	7.2 (6.7 - 7.7)	4.2 (3.9 - 4.6)	4.7 (4.4 - 5.0)	2.8 (2.6 - 3.0)
	K. pneumoniae	5.4 (4.2-6.8)	17.3 (13.9-21.4)	3.7 (3.0 - 4.7)	12.7 (10.7 - 14.8)	3.8 (3.3 - 4.4)	12.5 (11.0 - 14.1)
	L. monocytogenes	1.8 (1.3-2.5)	4.6 (3.2-6.3)	2.2 (1.6 - 2.8)	4.9 (3.9 - 6.1)	2.1 (1.8 - 2.4)	5.3 (4.7 - 6.0)
	N. meningitidis	7.9 (6.9-9.1)	6.7 (5.7-7.8)	18.1 (17.1 - 19.0)	12.4 (11.7 - 13.2)	18.3 (17.6 - 18.9)	13.4 (12.8 - 14.0)
	Other	2.1 (1.3-3.2)	2.6 (1.6-4.0)	9.4 (6.6 - 13.0)	8.6 (6.3 - 11.9)	9.7 (8.0 - 11.7)	10.7 (9.0 - 12.7)
	S. aureus	4.0 (3.2-5.0)	5.2 (4.2-6.5)	4.6 (4.2 - 5.1)	5.4 (4.9 - 6.0)	4.7 (4.4 - 5.1)	6.1 (5.7 - 6.6)
	S. pneumoniae	2.7 (2.3-3.2)	4.5 (3.8-5.4)	15.0 (14.1 - 15.8)	18.6 (17.4 - 19.9)	13.8 (13.2 - 14.3)	20.7 (19.8 - 21.7)
	Virus	35.8 (33.0-38.8)	12.7 (11.3-14.1)	25.0 (23.9 - 26.1)	8.9 (8.4 - 9.4)	31.5 (30.5 - 32.4)	10.6 (10.2 - 11.0)
Bermuda	*E. coli*	6.5 (5.3-7.9)	13.3 (10.9-15.7)	5.4 (4.6 - 6.2)	11.4 (10.1 - 12.9)	5.2 (4.7 - 5.7)	12.0 (10.8 - 13.2)
	Group B *Streptococcus*	21.5 (20.6-22.5)	24.8 (23.4-26.0)	8.2 (7.7 - 8.7)	12.0 (11.4 - 12.7)	6.2 (5.9 - 6.5)	6.9 (6.5 - 7.3)
	H. influenzae	8.8 (7.4-10.5)	5.4 (4.4-6.6)	24.1 (22.7 - 25.3)	13.0 (12.1 - 13.9)	11.3 (10.8 - 11.8)	5.7 (5.3 - 6.1)
	K. pneumoniae	5.1 (3.9-6.5)	18.0 (14.5-22.4)	3.6 (2.9 - 4.4)	13.9 (11.8 - 16.3)	4.2 (3.8 - 4.7)	14.1 (12.6 - 15.5)
	L. monocytogenes	1.9 (1.3-2.5)	5.0 (3.6-6.7)	2.1 (1.6 - 2.7)	5.7 (4.5 - 7.1)	2.5 (2.3 - 2.8)	7.8 (7.1 - 8.6)
	N. meningitidis	11.2 (10.1-12.4)	9.8 (8.7-10.9)	11.1 (10.5 - 11.7)	9.8 (9.1 - 10.4)	11.2 (10.8 - 11.6)	7.8 (7.3 - 8.2)
	Other	1.8 (1.1-2.9)	2.4 (1.4-3.8)	9.3 (6.3 - 13.2)	10.1 (7.0 - 14.1)	10.6 (8.4 - 13.4)	12.7 (9.2 - 17.1)
	S. aureus	4.2 (3.3-5.2)	5.7 (4.4-7.1)	3.9 (3.5 - 4.4)	5.4 (4.9 - 6.0)	4.8 (4.5 - 5.0)	6.8 (6.4 - 7.3)

Location	Aetiology	Neonatal		Under 5		All Ages	
		Nonfatal	Fatal	Nonfatal	Fatal	Nonfatal	Fatal
	S. pneumoniae	3.1 (2.6-3.6)	4.7 (3.9-5.6)	7.9 (7.5 - 8.4)	10.9 (10.1 - 11.7)	8.9 (8.5 - 9.2)	15.3 (14.3 - 16.1)
	Virus	35.9 (33.5-38.4)	10.9 (9.8-12.1)	24.4 (23.2 - 25.4)	7.8 (7.3 - 8.3)	35.0 (33.8 - 36.1)	10.9 (10.3 - 11.5)
Cuba	*E. coli*	7.2 (6.1-8.4)	13.8 (11.8-15.9)	6.1 (5.3 - 7.0)	11.3 (10.0 - 12.7)	5.3 (4.9 - 5.8)	10.7 (9.8 - 11.7)
	Group B *Streptococcus*	28.9 (26.8-31.3)	31.8 (29.3-34.3)	10.0 (9.4 - 10.7)	12.5 (11.7 - 13.2)	7.0 (6.7 - 7.3)	7.3 (6.9 - 7.7)
	H. influenzae	6.7 (5.3-8.4)	4.0 (3.1-5.1)	6.3 (5.7 - 6.9)	3.5 (3.1 - 3.8)	3.9 (3.6 - 4.2)	2.2 (2.0 - 2.4)
	K. pneumoniae	5.4 (4.2-6.8)	17.9 (14.5-22.0)	3.9 (3.2 - 4.7)	12.9 (11.0 - 15.1)	4.1 (3.7 - 4.5)	12.9 (11.8 - 14.0)
	L. monocytogenes	1.8 (1.3-2.5)	4.6 (3.2-6.2)	2.4 (1.8 - 3.1)	5.6 (4.4 - 7.0)	2.5 (2.3 - 2.8)	6.9 (6.3 - 7.5)
	N. meningitidis	6.8 (5.9-7.8)	5.7 (4.8-6.6)	14.6 (13.7 - 15.4)	10.7 (9.9 - 11.4)	13.8 (13.3 - 14.3)	9.2 (8.8 - 9.7)
	Other	1.9 (1.1-3.0)	2.4 (1.5-3.8)	10.1 (7.0 - 14.2)	10.2 (7.2 - 14.2)	10.4 (8.4 - 12.9)	11.8 (9.0 - 14.9)
	S. aureus	3.9 (3.1-4.8)	5.1 (4.1-6.2)	4.7 (4.2 - 5.2)	5.6 (5.0 - 6.1)	5.1 (4.8 - 5.3)	6.6 (6.2 - 7.1)
	S. pneumoniae	2.6 (2.1-3.1)	3.9 (3.3-4.7)	15.7 (14.6 - 16.6)	20.0 (18.5 - 21.5)	14.3 (13.7 - 14.9)	22.1 (21.0 - 23.2)
	Virus	34.8 (31.9-37.7)	10.7 (9.5-12.0)	26.2 (24.9 - 27.4)	7.6 (7.2 - 8.1)	33.5 (32.5 - 34.4)	10.2 (9.7 - 10.6)
Dominica	*E. coli*	6.9 (5.7-8.4)	13.2 (10.8-15.7)	5.6 (4.7 - 6.5)	10.4 (9.1 - 11.8)	4.9 (4.2 - 5.5)	9.6 (8.4 - 10.7)
	Group B *Streptococcus*	26.0 (23.4-28.6)	28.6 (25.8-31.4)	9.4 (8.7 - 10.0)	12.6 (11.8 - 13.4)	6.8 (6.4 - 7.2)	7.9 (7.4 - 8.3)
	H. influenzae	7.6 (6.2-9.3)	4.8 (3.8-6.0)	6.5 (6.0 - 7.0)	3.9 (3.5 - 4.2)	4.4 (4.1 - 4.7)	2.6 (2.4 - 2.7)
	K. pneumoniae	5.4 (4.1-6.9)	17.3 (13.8-21.5)	3.7 (3.0 - 4.7)	12.3 (10.3 - 14.4)	3.9 (3.3 - 4.5)	12.0 (10.5 - 13.5)
	L. monocytogenes	1.8 (1.3-2.5)	4.5 (3.2-6.3)	2.1 (1.6 - 2.8)	4.9 (3.9 - 6.1)	2.2 (1.9 - 2.5)	5.7 (5.0 - 6.4)
	N. meningitidis	7.8 (6.7-8.9)	6.5 (5.6-7.7)	18.3 (17.3 - 19.2)	12.8 (12.0 - 13.6)	18.0 (17.3 - 18.6)	12.5 (11.9 - 13.1)
	Other	2.1 (1.3-3.2)	2.6 (1.6-4.0)	9.3 (6.6 - 12.9)	8.9 (6.4 - 12.2)	9.6 (8.0 - 11.6)	10.6 (8.6 - 12.9)
	S. aureus	4.0 (3.2-5.0)	5.2 (4.1-6.4)	4.6 (4.2 - 5.1)	5.4 (4.9 - 6.0)	4.9 (4.5 - 5.3)	6.2 (5.8 - 6.8)
	S. pneumoniae	2.7 (2.2-3.2)	4.5 (3.7-5.4)	15.5 (14.5 - 16.4)	20.1 (18.7 - 21.4)	14.3 (13.7 - 14.9)	22.4 (21.3 - 23.4)
	Virus	35.8 (32.9-38.8)	12.7 (11.3-14.2)	25.0 (23.9 - 26.1)	8.7 (8.2 - 9.2)	31.0 (30.1 - 31.9)	10.7 (10.3 - 11.2)
Dominican Republic	*E. coli*	6.6 (5.4-8.0)	12.8 (10.6-15.2)	5.8 (4.9 - 6.7)	11.3 (10.1 - 12.8)	5.1 (4.4 - 5.7)	10.2 (9.2 - 11.3)
	Group B *Streptococcus*	23.6 (21.7-25.7)	26.5 (24.2-28.8)	9.7 (9.0 - 10.3)	14.6 (13.6 - 15.5)	7.5 (7.1 - 7.9)	9.7 (9.2 - 10.2)
	H. influenzae	8.3 (7.0-9.7)	5.3 (4.4-6.3)	9.7 (9.1 - 10.3)	5.6 (5.2 - 6.1)	6.6 (6.3 - 7.0)	3.8 (3.6 - 4.0)
	K. pneumoniae	5.5 (4.3-6.8)	18.0 (14.6-21.8)	3.8 (3.1 - 4.7)	13.8 (11.8 - 15.8)	3.9 (3.3 - 4.5)	13.1 (11.6 - 14.6)
	L. monocytogenes	1.9 (1.3-2.6)	4.7 (3.3-6.5)	2.2 (1.7 - 2.8)	5.2 (4.2 - 6.4)	2.2 (1.9 - 2.6)	5.7 (5.0 - 6.5)
	N. meningitidis	8.5 (7.5-9.5)	7.3 (6.3-8.3)	15.8 (14.9 - 16.6)	11.3 (10.6 - 12.1)	16.4 (15.8 - 16.9)	12.1 (11.6 - 12.7)
	Other	2.1 (1.3-3.3)	2.7 (1.7-4.1)	9.5 (6.7 - 13.1)	8.8 (6.4 - 11.7)	9.7 (7.8 - 11.9)	10.6 (8.7 - 12.8)
	S. aureus	4.2 (3.4-5.2)	5.5 (4.4-6.7)	4.9 (4.5 - 5.4)	6.0 (5.4 - 6.6)	5.0 (4.7 - 5.4)	6.6 (6.1 - 7.0)

Location	Aetiology	Neonatal		Under 5		All Ages	
		Nonfatal	Fatal	Nonfatal	Fatal	Nonfatal	Fatal
	S. pneumoniae	2.9 (2.5-3.3)	4.1 (3.5-4.8)	12.6 (11.9 - 13.4)	13.6 (12.7 - 14.5)	12.8 (12.3 - 13.3)	17.1 (16.4 - 17.9)
	Virus	36.5 (33.7-39.2)	13.1 (11.7-14.5)	25.9 (24.8 - 27.1)	9.7 (9.1 - 10.3)	30.8 (29.9 - 31.6)	11.1 (10.7 - 11.5)
Grenada	*E. coli*	7.0 (5.8-8.4)	13.3 (11.1-15.8)	5.7 (4.8 - 6.6)	10.6 (9.3 - 12.1)	4.8 (4.2 - 5.4)	9.5 (8.5 - 10.6)
	Group B *Streptococcus*	26.9 (24.5-29.5)	29.6 (26.8-32.4)	9.4 (8.7 - 10.1)	12.9 (12.1 - 13.8)	6.7 (6.3 - 7.1)	7.4 (7.0 - 7.9)
	H. influenzae	7.3 (5.9-9.1)	4.6 (3.6-5.7)	6.5 (6.0 - 7.0)	3.8 (3.5 - 4.1)	4.0 (3.8 - 4.3)	2.3 (2.1 - 2.4)
	K. pneumoniae	5.4 (4.2-6.9)	17.4 (14.0-21.6)	3.8 (3.0 - 4.6)	12.5 (10.6 - 14.6)	3.9 (3.4 - 4.5)	12.3 (10.7 - 13.7)
	L. monocytogenes	1.8 (1.3-2.5)	4.5 (3.2-6.3)	2.2 (1.7 - 2.8)	5.0 (4.0 - 6.2)	2.2 (1.9 - 2.5)	5.9 (5.2 - 6.6)
	N. meningitidis	7.4 (6.4-8.5)	6.2 (5.3-7.3)	17.6 (16.5 - 18.5)	12.3 (11.5 - 13.0)	17.3 (16.7 - 18.0)	11.9 (11.4 - 12.5)
	Other	2.0 (1.2-3.2)	2.6 (1.6-4.0)	9.5 (6.7 - 13.2)	9.1 (6.6 - 12.4)	9.8 (8.1 - 11.8)	11.0 (9.0 - 13.4)
	S. aureus	4.0 (3.1-4.9)	5.1 (4.1-6.3)	4.6 (4.2 - 5.1)	5.5 (4.9 - 6.0)	4.9 (4.6 - 5.2)	6.4 (5.9 - 6.9)
	S. pneumoniae	2.7 (2.2-3.2)	4.3 (3.6-5.2)	15.6 (14.7 - 16.6)	19.9 (18.5 - 21.2)	14.4 (13.8 - 15.0)	22.7 (21.6 - 23.8)
	Virus	35.5 (32.6-38.6)	12.2 (10.8-13.7)	25.1 (23.9 - 26.2)	8.5 (8.0 - 9.0)	31.9 (30.9 - 32.8)	10.6 (10.2 - 11.1)
Guyana	*E. coli*	6.4 (5.0-8.0)	12.3 (9.9-15.1)	5.8 (4.8 - 6.9)	11.2 (9.6 - 13.0)	4.7 (4.0 - 5.5)	9.4 (8.1 - 10.8)
	Group B *Streptococcus*	23.1 (20.2-26.3)	25.9 (22.5-29.5)	9.8 (8.9 - 10.6)	13.2 (12.1 - 14.3)	7.2 (6.7 - 7.7)	8.3 (7.7 - 9.0)
	H. influenzae	8.4 (6.6-10.4)	5.5 (4.3-6.9)	7.5 (6.9 - 8.1)	4.8 (4.4 - 5.3)	4.7 (4.4 - 5.0)	2.7 (2.5 - 2.9)
	K. pneumoniae	5.9 (4.6-7.6)	19.1 (15.3-23.8)	3.8 (3.0 - 4.7)	13.3 (11.1 - 15.8)	3.8 (3.1 - 4.5)	12.8 (10.9 - 14.7)
	L. monocytogenes	1.9 (1.3-2.8)	4.8 (3.2-6.9)	2.2 (1.7 - 2.9)	5.4 (4.2 - 6.8)	2.1 (1.8 - 2.5)	5.6 (4.9 - 6.5)
	N. meningitidis	7.5 (6.3-8.9)	6.5 (5.4-7.7)	15.8 (14.7 - 16.8)	12.1 (11.1 - 13.1)	16.7 (16.0 - 17.5)	13.6 (12.8 - 14.3)
	Other	2.3 (1.4-3.5)	2.9 (1.8-4.5)	9.6 (6.8 - 13.3)	9.9 (7.1 - 13.5)	9.6 (7.8 - 11.7)	11.6 (9.7 - 13.8)
	S. aureus	4.4 (3.4-5.6)	5.8 (4.5-7.3)	5.5 (4.8 - 6.1)	6.8 (6.0 - 7.6)	5.4 (4.9 - 5.9)	7.4 (6.7 - 8.2)
	S. pneumoniae	2.9 (2.3-3.5)	3.1 (2.5-3.9)	14.8 (13.8 - 15.9)	13.4 (12.4 - 14.6)	14.9 (14.2 - 15.6)	16.6 (15.7 - 17.6)
	Virus	37.2 (33.9-40.5)	14.1 (12.4-15.9)	25.4 (24.0 - 26.6)	9.9 (9.3 - 10.5)	30.9 (29.9 - 31.9)	11.9 (11.4 - 12.4)
Haiti	*E. coli*	6.4 (4.8-8.1)	12.0 (9.2-15.1)	4.9 (4.0 - 6.0)	9.1 (7.7 - 10.9)	4.5 (3.7 - 5.4)	8.7 (7.4 - 10.3)
	Group B *Streptococcus*	20.3 (17.9-23.2)	22.6 (19.8-25.6)	8.2 (7.5 - 9.0)	10.5 (9.5 - 11.4)	7.0 (6.4 - 7.7)	9.4 (8.6 - 10.2)
	H. influenzae	9.6 (8.2-11.2)	6.5 (5.4-7.7)	9.2 (8.6 - 9.8)	5.7 (5.3 - 6.2)	7.6 (7.1 - 8.1)	5.1 (4.8 - 5.5)
	K. pneumoniae	5.2 (3.8-6.8)	16.1 (12.4-20.4)	3.6 (2.8 - 4.6)	11.3 (9.2 - 13.8)	3.6 (2.9 - 4.5)	11.3 (9.2 - 13.7)
	L. monocytogenes	1.9 (1.2-2.6)	4.5 (3.0-6.4)	1.9 (1.4 - 2.4)	4.3 (3.3 - 5.5)	1.8 (1.4 - 2.3)	4.3 (3.4 - 5.4)
	N. meningitidis	10.5 (9.2-12.2)	9.0 (7.6-10.6)	22.8 (21.6 - 24.0)	16.6 (15.6 - 17.7)	23.1 (22.1 - 24.1)	17.1 (16.1 - 18.1)
	Other	2.3 (1.4-3.5)	2.9 (1.8-4.4)	8.3 (5.8 - 11.5)	8.3 (5.9 - 11.4)	8.5 (6.7 - 11.0)	8.8 (6.8 - 11.4)
	S. aureus	4.1 (3.1-5.4)	5.4 (4.1-7.1)	4.5 (3.9 - 5.0)	5.4 (4.7 - 6.0)	4.6 (4.1 - 5.1)	5.5 (4.9 - 6.2)

Location	Aetiology	Neonatal		Under 5		All Ages	
		Nonfatal	Fatal	Nonfatal	Fatal	Nonfatal	Fatal
	S. pneumoniae	3.0 (2.5-3.7)	5.5 (4.4-6.6)	12.9 (12.2 - 13.7)	19.0 (17.7 - 20.3)	12.5 (11.9 - 13.2)	19.3 (18.2 - 20.5)
	Virus	36.8 (33.8-40.1)	15.5 (13.8-17.5)	23.7 (22.7 - 24.8)	9.9 (9.3 - 10.5)	26.7 (25.8 - 27.7)	10.4 (9.9 - 11.0)
Jamaica	*E. coli*	7.0 (5.9-8.4)	13.4 (11.3-15.8)	5.8 (4.9 - 6.7)	11.7 (10.5 - 13.2)	5.0 (4.4 - 5.5)	9.9 (8.9 - 11.0)
	Group B *Streptococcus*	27.4 (25.0-29.9)	30.2 (27.4-32.9)	9.7 (9.1 - 10.4)	18.9 (17.5 - 20.3)	7.1 (6.7 - 7.5)	8.1 (7.7 - 8.5)
	H. influenzae	7.1 (5.7-8.9)	4.4 (3.5-5.6)	6.2 (5.7 - 6.7)	3.9 (3.4 - 4.5)	4.2 (3.9 - 4.5)	2.2 (2.1 - 2.4)
	K. pneumoniae	5.4 (4.2-6.9)	17.6 (14.2-21.8)	3.8 (3.1 - 4.7)	14.4 (12.2 - 16.7)	3.9 (3.4 - 4.5)	12.6 (11.1 - 13.8)
	L. monocytogenes	1.8 (1.3-2.5)	4.5 (3.2-6.3)	2.2 (1.7 - 2.9)	5.0 (4.0 - 6.1)	2.2 (2.0 - 2.5)	6.1 (5.5 - 6.7)
	N. meningitidis	7.2 (6.2-8.3)	6.0 (5.1-7.1)	16.7 (15.7 - 17.5)	9.8 (9.2 - 10.5)	16.6 (16.0 - 17.2)	11.2 (10.7 - 11.7)
	Other	2.0 (1.2-3.1)	2.5 (1.5-3.9)	9.6 (6.8 - 13.3)	7.0 (5.2 - 9.4)	9.9 (8.1 - 11.9)	10.9 (8.8 - 13.3)
	S. aureus	4.0 (3.2-4.9)	5.1 (4.1-6.3)	4.7 (4.3 - 5.2)	5.4 (4.8 - 6.1)	4.9 (4.6 - 5.2)	6.4 (6.0 - 6.9)
	S. pneumoniae	2.6 (2.2-3.2)	4.2 (3.5-5.0)	15.7 (14.7 - 16.6)	14.4 (13.5 - 15.4)	14.4 (13.8 - 15.0)	21.6 (20.6 - 22.6)
	Virus	35.4 (32.5-38.4)	11.9 (10.5-13.3)	25.5 (24.3 - 26.6)	9.5 (8.8 - 10.3)	31.8 (30.9 - 32.7)	10.9 (10.5 - 11.4)
Puerto Rico	*E. coli*	6.5 (5.3-7.8)	12.9 (10.6-15.4)	6.5 (5.7 - 7.3)	13.1 (11.7 - 14.5)	5.4 (5.0 - 5.9)	12.0 (11.0 - 13.0)
	Group B *Streptococcus*	24.9 (22.1-27.9)	28.2 (24.8-31.7)	10.8 (10.0 - 11.6)	16.3 (15.1 - 17.6)	7.5 (7.1 - 7.9)	8.9 (8.4 - 9.4)
	H. influenzae	7.7 (6.0-9.6)	4.7 (3.5-6.0)	9.2 (8.4 - 10.0)	5.3 (4.8 - 5.9)	4.6 (4.3 - 4.9)	2.8 (2.6 - 3.0)
	K. pneumoniae	6.1 (4.8-7.8)	21.1 (17.0-25.8)	3.9 (3.2 - 4.7)	15.7 (13.7 - 18.1)	4.1 (3.7 - 4.5)	14.4 (13.2 - 15.7)
	L. monocytogenes	1.9 (1.4-2.7)	5.0 (3.6-7.0)	2.5 (1.9 - 3.2)	6.4 (5.1 - 7.9)	2.6 (2.4 - 2.9)	7.8 (7.2 - 8.5)
	N. meningitidis	6.9 (5.8-8.1)	5.9 (4.9-7.0)	10.3 (9.5 - 11.0)	8.1 (7.4 - 8.9)	10.5 (10.0 - 11.0)	8.2 (7.7 - 8.7)
	Other	2.0 (1.2-3.2)	2.6 (1.6-4.1)	10.6 (7.2 - 15.0)	10.4 (7.3 - 14.4)	10.7 (8.4 - 13.2)	13.3 (10.3 - 16.9)
	S. aureus	4.5 (3.6-5.6)	6.0 (4.8-7.4)	5.5 (5.0 - 6.1)	7.0 (6.3 - 7.8)	5.7 (5.4 - 6.1)	8.2 (7.7 - 8.7)
	S. pneumoniae	2.8 (2.2-3.6)	2.3 (1.8-2.9)	13.7 (12.7 - 14.7)	8.7 (8.0 - 9.5)	14.4 (13.7 - 15.1)	13.1 (12.3 - 13.9)
	Virus	36.7 (33.6-39.8)	11.3 (9.9-12.7)	27.0 (25.5 - 28.4)	8.9 (8.3 - 9.5)	34.5 (33.3 - 35.6)	11.3 (10.7 - 11.8)
Saint Kitts and Nevis	*E. coli*	7.1 (6.0-8.4)	13.6 (11.6-15.8)	5.9 (5.1 - 6.8)	11.5 (10.3 - 12.7)	5.1 (4.6 - 5.6)	10.1 (9.2 - 11.0)
	Group B *Streptococcus*	28.1 (25.8-30.4)	30.9 (28.2-33.4)	10.0 (9.3 - 10.7)	15.9 (14.9 - 16.8)	7.2 (6.8 - 7.5)	8.1 (7.7 - 8.5)
	H. influenzae	6.9 (5.5-8.6)	4.2 (3.3-5.4)	6.4 (5.9 - 6.9)	3.7 (3.3 - 4.1)	4.1 (3.8 - 4.4)	2.3 (2.1 - 2.5)
	K. pneumoniae	5.4 (4.2-6.9)	17.7 (14.4-21.9)	3.9 (3.2 - 4.7)	13.6 (11.7 - 15.7)	4.0 (3.5 - 4.5)	12.9 (11.6 - 14.1)
	L. monocytogenes	1.8 (1.3-2.5)	4.6 (3.2-6.3)	2.3 (1.8 - 2.9)	5.2 (4.2 - 6.4)	2.3 (2.1 - 2.6)	6.3 (5.8 - 6.9)
	N. meningitidis	7.1 (6.1-8.1)	5.9 (5.0-6.9)	15.7 (14.7 - 16.5)	10.4 (9.7 - 11.1)	15.4 (14.8 - 15.9)	10.3 (9.8 - 10.8)
	Other	1.9 (1.2-3.1)	2.5 (1.5-3.9)	9.8 (6.8 - 13.6)	8.5 (6.2 - 11.6)	10.1 (8.3 - 12.4)	11.3 (9.1 - 14.0)
	S. aureus	3.9 (3.2-4.9)	5.1 (4.1-6.3)	4.7 (4.2 - 5.1)	5.4 (4.9 - 6.0)	4.9 (4.6 - 5.2)	6.5 (6.1 - 6.9)

Location	Aetiology	Neonatal		Under 5		All Ages	
		Nonfatal	Fatal	Nonfatal	Fatal	Nonfatal	Fatal
	S. pneumoniae	2.6 (2.2-3.1)	4.1 (3.4-4.9)	15.5 (14.5 - 16.4)	17.2 (16.0 - 18.4)	14.3 (13.7 - 14.9)	21.8 (20.7 - 22.8)
	Virus	35.1 (32.2-38.1)	11.3 (10.0-12.7)	25.9 (24.6 - 27.0)	8.5 (8.0 - 9.1)	32.6 (31.6 - 33.5)	10.3 (9.9 - 10.7)
Saint Lucia	*E. coli*	7.0 (5.9-8.4)	13.4 (11.3-15.8)	5.7 (4.9 - 6.6)	11.0 (9.8 - 12.3)	4.9 (4.3 - 5.5)	9.7 (8.6 - 10.7)
	Group B *Streptococcus*	27.1 (24.7-29.5)	29.9 (27.2-32.6)	9.6 (8.9 - 10.2)	14.6 (13.7 - 15.6)	6.7 (6.3 - 7.1)	7.4 (7.0 - 7.8)
	H. influenzae	7.2 (5.9-9.0)	4.5 (3.6-5.7)	6.7 (6.1 - 7.2)	3.9 (3.5 - 4.3)	4.1 (3.8 - 4.4)	2.3 (2.1 - 2.4)
	K. pneumoniae	5.4 (4.2-6.9)	17.5 (14.1-21.7)	3.8 (3.1 - 4.7)	13.1 (11.2 - 15.2)	4.0 (3.4 - 4.5)	12.5 (11.0 - 13.8)
	L. monocytogenes	1.8 (1.3-2.5)	4.5 (3.2-6.3)	2.2 (1.7 - 2.8)	5.1 (4.1 - 6.2)	2.3 (2.0 - 2.5)	6.0 (5.4 - 6.7)
	N. meningitidis	7.4 (6.4-8.5)	6.2 (5.3-7.2)	17.0 (16.0 - 17.8)	11.4 (10.7 - 12.1)	16.6 (16.0 - 17.2)	11.7 (11.1 - 12.2)
	Other	2.0 (1.2-3.1)	2.5 (1.6-3.9)	9.6 (6.7 - 13.3)	8.5 (6.2 - 11.7)	9.9 (8.2 - 11.9)	11.2 (9.1 - 13.6)
	S. aureus	4.0 (3.2-4.9)	5.1 (4.1-6.3)	4.6 (4.2 - 5.1)	5.4 (4.9 - 6.0)	4.9 (4.6 - 5.3)	6.4 (6.0 - 6.9)
	S. pneumoniae	2.7 (2.2-3.2)	4.3 (3.6-5.1)	15.4 (14.5 - 16.3)	18.1 (16.9 - 19.3)	14.2 (13.6 - 14.7)	22.1 (21.0 - 23.1)
	Virus	35.4 (32.5-38.4)	12.0 (10.6-13.4)	25.3 (24.2 - 26.5)	8.7 (8.2 - 9.2)	32.4 (31.4 - 33.3)	10.8 (10.4 - 11.2)
Saint Vincent and the Grenadines	*E. coli*	7.0 (5.7-8.4)	13.3 (11.0-15.8)	5.6 (4.7 - 6.5)	10.6 (9.3 - 12.0)	4.8 (4.2 - 5.5)	9.3 (8.2 - 10.5)
	Group B *Streptococcus*	26.8 (24.1-29.6)	29.4 (26.5-32.4)	9.5 (8.8 - 10.1)	13.7 (12.8 - 14.7)	6.9 (6.4 - 7.3)	7.6 (7.1 - 8.0)
	H. influenzae	7.4 (5.9-9.2)	4.6 (3.6-5.9)	6.1 (5.6 - 6.6)	3.7 (3.3 - 4.1)	4.0 (3.7 - 4.3)	2.2 (2.1 - 2.4)
	K. pneumoniae	5.4 (4.1-6.9)	17.3 (13.8-21.6)	3.7 (3.0 - 4.6)	12.6 (10.6 - 14.7)	3.9 (3.3 - 4.5)	12.1 (10.5 - 13.6)
	L. monocytogenes	1.8 (1.3-2.5)	4.5 (3.1-6.3)	2.2 (1.6 - 2.8)	4.9 (3.9 - 6.0)	2.2 (1.9 - 2.5)	5.7 (5.0 - 6.4)
	N. meningitidis	7.4 (6.3-8.6)	6.2 (5.2-7.3)	18.2 (17.1 - 19.1)	12.3 (11.5 - 13.1)	17.9 (17.2 - 18.5)	12.4 (11.8 - 13.0)
	Other	2.1 (1.3-3.2)	2.6 (1.6-4.0)	9.3 (6.6 - 12.9)	8.5 (6.2 - 11.7)	9.7 (8.0 - 11.6)	10.7 (8.8 - 13.0)
	S. aureus	4.0 (3.1-5.0)	5.1 (4.1-6.4)	4.6 (4.2 - 5.1)	5.4 (4.9 - 6.0)	4.9 (4.5 - 5.3)	6.3 (5.8 - 6.9)
	S. pneumoniae	2.7 (2.2-3.2)	4.4 (3.6-5.3)	15.9 (14.9 - 16.9)	19.6 (18.2 - 20.9)	14.8 (14.1 - 15.4)	22.9 (21.8 - 24.0)
	Virus	35.6 (32.6-38.7)	12.5 (11.1-14.0)	24.9 (23.8 - 26.0)	8.8 (8.3 - 9.3)	31.0 (30.1 - 31.9)	10.8 (10.3 - 11.2)
Suriname	*E. coli*	7.0 (5.7-8.5)	13.2 (10.8-15.9)	5.5 (4.6 - 6.5)	10.4 (9.1 - 11.8)	4.8 (4.1 - 5.5)	9.2 (8.1 - 10.4)
	Group B *Streptococcus*	26.6 (23.8-29.6)	29.2 (26.1-32.4)	9.4 (8.6 - 10.1)	13.5 (12.5 - 14.5)	7.0 (6.5 - 7.5)	8.5 (8.0 - 9.1)
	H. influenzae	7.4 (5.9-9.3)	4.7 (3.7-5.9)	5.7 (5.2 - 6.1)	3.5 (3.1 - 3.9)	4.0 (3.7 - 4.3)	2.4 (2.2 - 2.6)
	K. pneumoniae	5.4 (4.1-7.0)	17.2 (13.6-21.5)	3.7 (3.0 - 4.7)	12.3 (10.3 - 14.5)	3.8 (3.2 - 4.5)	12.0 (10.4 - 13.6)
	L. monocytogenes	1.8 (1.3-2.6)	4.5 (3.1-6.3)	2.1 (1.6 - 2.7)	4.8 (3.8 - 5.9)	2.1 (1.8 - 2.5)	5.3 (4.6 - 6.1)
	N. meningitidis	7.4 (6.3-8.6)	6.2 (5.2-7.4)	19.0 (17.8 - 19.9)	12.7 (11.9 - 13.5)	18.8 (18.0 - 19.6)	13.2 (12.5 - 13.8)
	Other	2.1 (1.3-3.2)	2.6 (1.6-4.0)	9.2 (6.5 - 12.7)	8.4 (6.0 - 11.5)	9.5 (7.8 - 11.4)	10.2 (8.4 - 12.3)
	S. aureus	4.0 (3.1-5.0)	5.1 (4.0-6.4)	4.6 (4.1 - 5.2)	5.4 (4.8 - 6.0)	4.8 (4.4 - 5.2)	6.1 (5.6 - 6.6)

Location	Aetiology	Neonatal		Under 5		All Ages	
		Nonfatal	Fatal	Nonfatal	Fatal	Nonfatal	Fatal
	S. pneumoniae	2.7 (2.2-3.2)	4.4 (3.6-5.4)	16.3 (15.3 - 17.3)	20.3 (18.9 - 21.7)	15.1 (14.4 - 15.8)	22.7 (21.6 - 23.7)
	Virus	35.7 (32.6-38.9)	12.9 (11.3-14.4)	24.6 (23.5 - 25.7)	8.9 (8.4 - 9.4)	30.1 (29.2 - 31.0)	10.5 (10.1 - 10.9)
Trinidad and Tobago	*E. coli*	6.5 (5.3-7.7)	12.8 (10.6-15.1)	6.0 (5.1 - 6.9)	11.6 (10.2 - 13.2)	4.9 (4.4 - 5.5)	10.0 (8.9 - 11.1)
	Group B *Streptococcus*	23.6 (21.5-26.0)	26.6 (24.1-29.1)	9.6 (8.9 - 10.3)	12.9 (12.0 - 13.8)	6.8 (6.4 - 7.1)	7.3 (6.9 - 7.8)
	H. influenzae	8.2 (6.8-9.8)	5.3 (4.3-6.3)	9.5 (8.9 - 10.2)	5.6 (5.2 - 6.1)	5.2 (4.9 - 5.5)	2.8 (2.6 - 3.0)
	K. pneumoniae	5.7 (4.5-7.1)	18.9 (15.6-22.8)	3.8 (3.1 - 4.7)	13.6 (11.5 - 15.9)	4.0 (3.5 - 4.5)	13.1 (11.5 - 14.6)
	L. monocytogenes	1.9 (1.3-2.7)	4.8 (3.4-6.7)	2.3 (1.7 - 2.9)	5.6 (4.5 - 7.1)	2.3 (2.0 - 2.6)	6.4 (5.7 - 7.1)
	N. meningitidis	8.0 (7.0-9.1)	6.9 (6.0-7.9)	14.4 (13.5 - 15.3)	11.2 (10.4 - 12.0)	14.9 (14.4 - 15.4)	11.6 (11.1 - 12.2)
	Other	2.1 (1.3-3.3)	2.7 (1.7-4.2)	10.0 (7.0 - 13.8)	10.4 (7.4 - 14.2)	10.1 (8.4 - 12.1)	12.3 (10.0 - 14.8)
	S. aureus	4.3 (3.4-5.3)	5.7 (4.6-7.0)	5.2 (4.7 - 5.8)	6.5 (5.9 - 7.2)	5.3 (5.0 - 5.7)	7.4 (6.8 - 7.9)
	S. pneumoniae	2.9 (2.4-3.4)	3.5 (2.9-4.1)	13.3 (12.5 - 14.2)	13.4 (12.5 - 14.4)	13.5 (13.1 - 14.0)	17.2 (16.4 - 18.1)
	Virus	36.7 (33.9-39.6)	12.9 (11.5-14.4)	25.9 (24.6 - 27.1)	9.2 (8.6 - 9.7)	33.0 (32.0 - 33.9)	11.8 (11.3 - 12.4)
United States Virgin Islands	*E. coli*	6.3 (5.0-7.6)	12.5 (10.2-15.0)	5.9 (5.1 - 6.8)	12.1 (10.8 - 13.5)	5.2 (4.6 - 5.7)	11.3 (10.1 - 12.5)
	Group B *Streptococcus*	22.3 (19.7-25.0)	25.4 (22.4-28.4)	9.9 (9.2 - 10.5)	16.0 (14.8 - 17.2)	7.3 (6.9 - 7.7)	8.1 (7.6 - 8.6)
	H. influenzae	8.6 (7.0-10.3)	5.5 (4.3-6.8)	11.4 (10.7 - 12.2)	6.5 (6.0 - 7.1)	6.1 (5.8 - 6.4)	3.2 (3.0 - 3.4)
	K. pneumoniae	6.0 (4.7-7.5)	20.3 (16.6-24.3)	3.8 (3.1 - 4.6)	15.5 (13.4 - 17.9)	4.0 (3.5 - 4.5)	13.7 (12.2 - 15.1)
	L. monocytogenes	1.9 (1.3-2.7)	5.0 (3.5-7.0)	2.3 (1.8 - 2.9)	5.8 (4.7 - 7.0)	2.4 (2.1 - 2.7)	7.3 (6.6 - 8.2)
	N. meningitidis	8.1 (7.0-9.4)	7.0 (5.9-8.2)	12.6 (11.8 - 13.4)	9.6 (8.9 - 10.4)	12.6 (12.0 - 13.1)	9.1 (8.6 - 9.7)
	Other	2.1 (1.3-3.3)	2.8 (1.7-4.3)	10.0 (6.9 - 13.8)	9.1 (6.6 - 12.3)	10.3 (8.4 - 12.5)	12.6 (9.8 - 16.1)
	S. aureus	4.5 (3.6-5.6)	6.1 (4.9-7.5)	5.3 (4.8 - 5.8)	6.7 (6.1 - 7.5)	5.6 (5.3 - 6.0)	8.2 (7.6 - 8.8)
	S. pneumoniae	3.0 (2.4-3.7)	2.7 (2.2-3.4)	13.0 (12.3 - 13.9)	8.7 (8.1 - 9.4)	13.6 (13.0 - 14.2)	14.6 (13.7 - 15.5)
	Virus	37.3 (34.4-40.4)	12.7 (11.3-14.3)	25.9 (24.6 - 27.0)	9.9 (9.2 - 10.6)	33.0 (32.0 - 33.9)	12.0 (11.4 - 12.5)
**Central Latin America**	*E. coli*	6.5 (5.4-7.8)	12.8 (10.7-15.1)	6.0 (5.2 - 6.9)	11.7 (10.3 - 13.3)	5.0 (4.5 - 5.6)	10.3 (9.3 - 11.4)
	Group B *Streptococcus*	24.2 (21.9-26.7)	27.2 (24.6-30.0)	10.1 (9.4 - 10.8)	12.6 (11.7 - 13.5)	7.4 (7.0 - 7.8)	8.3 (7.9 - 8.8)
	H. influenzae	8.0 (6.5-9.6)	5.1 (4.0-6.2)	9.1 (8.5 - 9.7)	5.4 (5.0 - 5.9)	5.4 (5.1 - 5.7)	3.0 (2.8 - 3.2)
	K. pneumoniae	5.8 (4.6-7.2)	19.4 (15.8-23.4)	3.8 (3.1 - 4.7)	13.5 (11.4 - 15.8)	3.9 (3.4 - 4.4)	13.4 (11.9 - 14.8)
	L. monocytogenes	1.9 (1.3-2.7)	4.8 (3.4-6.7)	2.3 (1.8 - 3.0)	5.8 (4.6 - 7.2)	2.3 (2.0 - 2.6)	6.4 (5.7 - 7.0)
	N. meningitidis	7.6 (6.6-8.7)	6.5 (5.6-7.6)	13.5 (12.7 - 14.3)	11.0 (10.1 - 11.8)	14.0 (13.5 - 14.6)	11.5 (10.9 - 12.0)
	Other	2.1 (1.3-3.3)	2.7 (1.7-4.2)	10.0 (7.0 - 13.8)	10.9 (7.7 - 14.9)	10.1 (8.2 - 12.3)	12.4 (10.2 - 15.0)
	S. aureus	4.3 (3.5-5.3)	5.7 (4.6-7.0)	5.3 (4.8 - 5.8)	6.7 (6.0 - 7.3)	5.4 (5.0 - 5.7)	7.4 (6.9 - 8.0)

Location	Aetiology	Neonatal		Under 5		All Ages	
		Nonfatal	Fatal	Nonfatal	Fatal	Nonfatal	Fatal
	S. pneumoniae	2.8 (2.4-3.4)	3.2 (2.7-3.8)	13.7 (12.9 - 14.6)	13.5 (12.6 - 14.6)	13.9 (13.3 - 14.4)	15.9 (15.1 - 16.7)
	Virus	36.7 (33.7-39.6)	12.6 (11.1-14.0)	26.1 (24.8 - 27.3)	8.8 (8.3 - 9.4)	32.6 (31.6 - 33.5)	11.3 (10.9 - 11.8)
Colombia	*E. coli*	6.5 (5.4-7.8)	12.9 (10.7-15.2)	6.3 (5.5 - 7.2)	12.5 (11.0 - 14.0)	5.4 (4.9 - 5.9)	11.0 (10.1 - 12.0)
	Group B *Streptococcus*	24.8 (22.3-27.6)	28.0 (25.0-31.2)	10.7 (9.9 - 11.5)	13.5 (12.5 - 14.6)	8.0 (7.6 - 8.4)	8.9 (8.5 - 9.4)
	H. influenzae	7.8 (6.1-9.6)	4.8 (3.7-6.1)	8.4 (7.8 - 9.1)	5.0 (4.6 - 5.5)	5.3 (5.0 - 5.7)	2.9 (2.8 - 3.1)
	K. pneumoniae	5.9 (4.7-7.4)	20.1 (16.4-24.2)	3.9 (3.2 - 4.7)	14.1 (12.1 - 16.5)	4.0 (3.5 - 4.5)	13.9 (12.5 - 15.2)
	L. monocytogenes	1.9 (1.3-2.7)	4.9 (3.5-6.8)	2.4 (1.9 - 3.1)	6.2 (4.9 - 7.7)	2.4 (2.1 - 2.7)	6.8 (6.2 - 7.5)
	N. meningitidis	7.1 (6.0-8.2)	6.1 (5.1-7.1)	11.9 (11.1 - 12.7)	9.7 (8.9 - 10.5)	12.5 (11.9 - 13.0)	10.2 (9.7 - 10.7)
	Other	2.1 (1.3-3.3)	2.7 (1.6-4.2)	10.1 (7.0 - 14.1)	11.3 (7.9 - 15.5)	10.3 (8.2 - 12.7)	12.8 (10.3 - 15.6)
	S. aureus	4.4 (3.5-5.4)	5.8 (4.7-7.2)	5.4 (4.9 - 6.0)	7.0 (6.3 - 7.7)	5.5 (5.2 - 5.8)	7.7 (7.2 - 8.2)
	S. pneumoniae	2.8 (2.3-3.4)	2.7 (2.2-3.3)	13.8 (12.8 - 14.8)	11.9 (10.9 - 12.9)	14.1 (13.5 - 14.8)	14.6 (13.9 - 15.3)
	Virus	36.6 (33.6-39.7)	12.0 (10.5-13.4)	26.9 (25.5 - 28.2)	8.8 (8.2 - 9.3)	32.5 (31.5 - 33.4)	11.2 (10.7 - 11.6)
Costa Rica	*E. coli*	6.5 (5.2-7.9)	12.8 (10.5-15.3)	6.4 (5.6 - 7.3)	12.9 (11.5 - 14.4)	5.4 (4.9 - 5.9)	11.4 (10.4 - 12.3)
	Group B *Streptococcus*	24.6 (21.8-27.7)	27.9 (24.5-31.5)	11.0 (10.1 - 11.8)	15.0 (13.8 - 16.3)	8.1 (7.7 - 8.6)	8.9 (8.5 - 9.5)
	H. influenzae	7.8 (6.0-9.7)	4.8 (3.6-6.2)	8.3 (7.6 - 9.1)	5.0 (4.5 - 5.5)	5.2 (4.9 - 5.6)	2.8 (2.6 - 3.0)
	K. pneumoniae	6.1 (4.7-7.8)	20.9 (16.8-25.6)	3.9 (3.2 - 4.7)	15.0 (12.9 - 17.3)	4.0 (3.6 - 4.5)	14.3 (13.0 - 15.5)
	L. monocytogenes	1.9 (1.3-2.7)	5.0 (3.5-7.0)	2.5 (1.9 - 3.2)	6.4 (5.0 - 7.9)	2.5 (2.2 - 2.8)	7.3 (6.7 - 8.0)
	N. meningitidis	6.9 (5.8-8.2)	5.9 (4.9-7.0)	10.9 (10.1 - 11.7)	8.8 (8.0 - 9.6)	11.4 (10.8 - 11.9)	9.2 (8.7 - 9.8)
	Other	2.1 (1.2-3.3)	2.7 (1.6-4.2)	10.3 (7.0 - 14.5)	11.0 (7.7 - 15.1)	10.4 (8.2 - 12.9)	13.2 (10.4 - 16.4)
	S. aureus	4.5 (3.6-5.6)	6.0 (4.8-7.4)	5.6 (5.0 - 6.2)	7.2 (6.5 - 8.0)	5.6 (5.3 - 6.0)	8.1 (7.6 - 8.7)
	S. pneumoniae	2.8 (2.2-3.6)	2.4 (1.8-2.9)	14.0 (12.9 - 15.1)	9.8 (9.1 - 10.7)	14.3 (13.6 - 15.0)	13.4 (12.6 - 14.2)
	Virus	36.8 (33.6-40.0)	11.7 (10.3-13.2)	27.1 (25.6 - 28.4)	8.9 (8.4 - 9.5)	33.0 (31.9 - 34.0)	11.4 (10.8 - 11.9)
El Salvador	*E. coli*	6.5 (5.2-7.8)	12.7 (10.3-15.2)	6.1 (5.2 - 7.1)	12.0 (10.5 - 13.7)	5.1 (4.5 - 5.7)	10.5 (9.4 - 11.7)
	Group B *Streptococcus*	24.2 (21.5-27.3)	27.3 (24.1-30.8)	10.3 (9.5 - 11.1)	13.2 (12.2 - 14.3)	7.5 (7.1 - 7.9)	8.1 (7.6 - 8.5)
	H. influenzae	8.0 (6.2-9.8)	5.0 (3.8-6.3)	8.2 (7.5 - 8.8)	5.0 (4.6 - 5.5)	4.8 (4.5 - 5.1)	2.6 (2.4 - 2.7)
	K. pneumoniae	6.0 (4.7-7.6)	20.0 (16.2-24.2)	3.8 (3.1 - 4.7)	13.8 (11.6 - 16.2)	3.9 (3.4 - 4.4)	13.4 (11.8 - 14.8)
	L. monocytogenes	1.9 (1.3-2.7)	4.9 (3.4-6.9)	2.4 (1.8 - 3.0)	6.0 (4.7 - 7.5)	2.3 (2.0 - 2.6)	6.7 (6.0 - 7.4)
	N. meningitidis	7.1 (6.0-8.3)	6.1 (5.1-7.2)	13.1 (12.2 - 13.9)	10.5 (9.7 - 11.4)	13.6 (13.0 - 14.2)	10.9 (10.3 - 11.5)
	Other	2.2 (1.3-3.4)	2.8 (1.7-4.3)	10.0 (7.0 - 14.0)	11.1 (7.8 - 15.1)	10.1 (8.2 - 12.3)	12.6 (10.2 - 15.4)
	S. aureus	4.4 (3.5-5.5)	5.8 (4.6-7.3)	5.5 (4.9 - 6.1)	7.0 (6.3 - 7.8)	5.5 (5.1 - 5.9)	7.9 (7.3 - 8.5)

Location	Aetiology	Neonatal		Under 5		All Ages	
		Nonfatal	Fatal	Nonfatal	Fatal	Nonfatal	Fatal
	S. pneumoniae	2.8 (2.3-3.5)	2.7 (2.2-3.4)	14.5 (13.5 - 15.5)	12.3 (11.4 - 13.3)	14.7 (14.0 - 15.3)	15.6 (14.8 - 16.5)
	Virus	36.9 (33.7-40.1)	12.7 (11.1-14.3)	26.2 (24.8 - 27.4)	9.1 (8.5 - 9.6)	32.5 (31.5 - 33.4)	11.8 (11.3 - 12.4)
Guatemala	*E. coli*	6.5 (5.3-8.0)	12.6 (10.2-15.2)	5.8 (4.8 - 6.8)	10.9 (9.3 - 12.7)	4.8 (4.1 - 5.5)	9.3 (8.0 - 10.6)
	Group B *Streptococcus*	23.8 (21.2-26.6)	26.6 (23.7-29.6)	9.7 (8.9 - 10.4)	10.9 (9.9 - 11.7)	7.1 (6.6 - 7.5)	7.4 (6.9 - 8.0)
	H. influenzae	8.2 (6.6-10.0)	5.3 (4.2-6.5)	8.3 (7.7 - 8.9)	5.0 (4.6 - 5.4)	5.0 (4.7 - 5.4)	2.8 (2.6 - 3.0)
	K. pneumoniae	5.7 (4.5-7.3)	18.5 (15.0-22.7)	3.8 (3.0 - 4.7)	12.3 (10.1 - 14.9)	3.8 (3.2 - 4.5)	12.5 (10.6 - 14.3)
	L. monocytogenes	1.9 (1.3-2.7)	4.7 (3.3-6.7)	2.2 (1.7 - 2.9)	5.4 (4.2 - 6.9)	2.1 (1.8 - 2.5)	5.6 (4.9 - 6.5)
	N. meningitidis	7.8 (6.7-9.0)	6.6 (5.6-7.8)	16.1 (15.1 - 17.1)	13.0 (12.0 - 14.1)	16.9 (16.3 - 17.6)	13.9 (13.2 - 14.6)
	Other	2.2 (1.3-3.4)	2.8 (1.7-4.3)	9.6 (6.8 - 13.3)	10.9 (7.7 - 15.0)	9.7 (7.9 - 11.7)	11.8 (9.8 - 14.0)
	S. aureus	4.3 (3.3-5.4)	5.6 (4.4-7.0)	5.2 (4.6 - 5.8)	6.6 (5.8 - 7.3)	5.2 (4.8 - 5.6)	7.0 (6.4 - 7.7)
	S. pneumoniae	2.8 (2.3-3.4)	3.6 (2.9-4.3)	14.0 (13.1 - 15.0)	16.3 (15.1 - 17.5)	14.1 (13.5 - 14.7)	18.0 (17.2 - 19.0)
	Virus	36.8 (33.8-39.9)	13.6 (12.0-15.3)	25.5 (24.2 - 26.7)	8.9 (8.3 - 9.5)	31.2 (30.3 - 32.2)	11.6 (11.1 - 12.1)
Honduras	*E. coli*	6.4 (5.1-8.0)	12.4 (10.0-15.2)	5.8 (4.9 - 6.9)	11.5 (10.0 - 13.2)	5.0 (4.2 - 5.7)	10.0 (8.7 - 11.4)
	Group B *Streptococcus*	23.5 (20.6-26.6)	26.4 (23.0-29.9)	10.0 (9.2 - 10.8)	15.4 (14.1 - 16.8)	7.7 (7.2 - 8.3)	8.8 (8.1 - 9.4)
	H. influenzae	8.3 (6.4-10.2)	5.4 (4.1-6.7)	7.5 (7.0 - 8.2)	4.9 (4.4 - 5.4)	5.0 (4.7 - 5.4)	2.7 (2.5 - 2.9)
	K. pneumoniae	5.9 (4.6-7.6)	19.3 (15.4-23.9)	3.8 (3.0 - 4.7)	14.4 (12.0 - 16.8)	3.8 (3.2 - 4.5)	12.8 (11.1 - 14.5)
	L. monocytogenes	1.9 (1.3-2.8)	4.8 (3.2-6.9)	2.2 (1.7 - 2.9)	5.4 (4.3 - 6.6)	2.1 (1.8 - 2.5)	6.0 (5.2 - 6.9)
	N. meningitidis	7.4 (6.2-8.7)	6.3 (5.3-7.5)	15.2 (14.2 - 16.3)	10.9 (10.0 - 11.8)	16.1 (15.3 - 16.8)	12.1 (11.4 - 12.8)
	Other	2.3 (1.4-3.5)	2.9 (1.8-4.5)	9.6 (6.7 - 13.3)	8.9 (6.5 - 11.9)	9.6 (7.8 - 11.8)	11.3 (9.2 - 13.6)
	S. aureus	4.4 (3.4-5.6)	5.8 (4.5-7.3)	5.4 (4.8 - 6.1)	6.6 (5.9 - 7.4)	5.4 (4.9 - 5.9)	7.5 (6.8 - 8.2)
	S. pneumoniae	2.9 (2.3-3.5)	3.1 (2.4-3.8)	14.7 (13.8 - 15.8)	11.7 (10.8 - 12.6)	14.9 (14.2 - 15.7)	16.8 (15.9 - 17.8)
	Virus	37.1 (33.9-40.4)	13.8 (12.1-15.5)	25.6 (24.3 - 26.8)	10.4 (9.7 - 11.1)	30.4 (29.4 - 31.3)	12.0 (11.4 - 12.5)
Mexico	*E. coli*	6.4 (5.2-7.8)	12.6 (10.4-15.1)	6.0 (5.1 - 6.9)	11.9 (10.4 - 13.5)	4.9 (4.4 - 5.5)	10.4 (9.4 - 11.5)
	Group B *Streptococcus*	23.6 (21.1-26.5)	26.7 (23.8-29.9)	9.9 (9.2 - 10.6)	12.5 (11.5 - 13.4)	7.2 (6.8 - 7.6)	8.3 (7.8 - 8.8)
	H. influenzae	8.2 (6.5-9.9)	5.2 (4.0-6.4)	9.4 (8.7 - 10.1)	5.7 (5.2 - 6.2)	5.2 (4.9 - 5.5)	3.0 (2.8 - 3.2)
	K. pneumoniae	5.9 (4.7-7.4)	19.9 (16.3-23.9)	3.8 (3.1 - 4.6)	13.6 (11.4 - 16.0)	3.9 (3.4 - 4.4)	13.7 (12.2 - 15.1)
	L. monocytogenes	1.9 (1.3-2.7)	4.9 (3.4-6.8)	2.3 (1.8 - 3.0)	6.0 (4.7 - 7.5)	2.3 (2.0 - 2.6)	6.5 (5.9 - 7.2)
	N. meningitidis	7.5 (6.4-8.7)	6.5 (5.5-7.6)	13.2 (12.3 - 14.0)	10.8 (10.0 - 11.7)	13.8 (13.2 - 14.4)	11.2 (10.6 - 11.8)
	Other	2.1 (1.3-3.3)	2.8 (1.7-4.3)	10.1 (7.0 - 14.0)	11.4 (8.0 - 15.5)	10.2 (8.3 - 12.3)	12.8 (10.5 - 15.5)
	S. aureus	4.4 (3.5-5.5)	5.9 (4.7-7.3)	5.4 (4.8 - 5.9)	7.0 (6.2 - 7.7)	5.4 (5.1 - 5.8)	7.7 (7.2 - 8.3)

Location	Aetiology	Neonatal		Under 5		All Ages	
		Nonfatal	Fatal	Nonfatal	Fatal	Nonfatal	Fatal
	S. pneumoniae	2.9 (2.3-3.5)	2.8 (2.3-3.5)	14.0 (13.1 - 14.9)	12.4 (11.5 - 13.4)	14.0 (13.4 - 14.6)	14.8 (14.1 - 15.6)
	Virus	37.0 (33.9-40.0)	12.6 (11.2-14.2)	25.9 (24.6 - 27.1)	8.8 (8.3 - 9.4)	33.1 (32.1 - 34.1)	11.5 (11.0 - 12.0)
Nicaragua	*E. coli*	6.5 (5.2-7.9)	12.6 (10.2-15.3)	6.2 (5.3 - 7.3)	12.3 (10.7 - 14.1)	5.1 (4.5 - 5.8)	10.9 (9.7 - 12.0)
	Group B *Streptococcus*	24.6 (21.6-28.0)	27.6 (24.1-31.5)	10.7 (9.8 - 11.5)	13.0 (11.8 - 14.1)	8.0 (7.5 - 8.5)	9.4 (8.8 - 10.0)
	H. influenzae	7.8 (5.9-9.8)	4.9 (3.6-6.3)	7.4 (6.8 - 8.0)	4.5 (4.1 - 5.0)	4.7 (4.4 - 5.1)	2.9 (2.7 - 3.1)
	K. pneumoniae	6.1 (4.7-7.8)	20.4 (16.3-25.0)	3.9 (3.1 - 4.7)	13.7 (11.5 - 16.2)	3.9 (3.3 - 4.4)	13.7 (12.1 - 15.3)
	L. monocytogenes	1.9 (1.3-2.7)	4.9 (3.4-7.0)	2.4 (1.8 - 3.1)	6.2 (4.8 - 7.8)	2.3 (1.9 - 2.6)	6.5 (5.7 - 7.3)
	N. meningitidis	6.8 (5.6-8.1)	5.8 (4.8-6.9)	12.4 (11.5 - 13.3)	10.3 (9.4 - 11.2)	13.3 (12.6 - 13.9)	11.0 (10.3 - 11.6)
	Other	2.2 (1.3-3.4)	2.8 (1.7-4.3)	10.1 (6.9 - 14.1)	11.6 (8.1 - 15.9)	10.1 (8.1 - 12.4)	12.7 (10.3 - 15.4)
	S. aureus	4.5 (3.5-5.6)	5.9 (4.6-7.4)	5.6 (5.0 - 6.3)	7.3 (6.5 - 8.2)	5.5 (5.1 - 6.0)	7.8 (7.3 - 8.4)
	S. pneumoniae	2.8 (2.2-3.5)	2.5 (1.9-3.2)	14.9 (13.8 - 16.1)	12.2 (11.2 - 13.4)	14.9 (14.2 - 15.7)	14.1 (13.3 - 14.9)
	Virus	36.9 (33.5-40.2)	12.5 (11.0-14.1)	26.5 (25.0 - 27.8)	8.9 (8.3 - 9.5)	32.2 (31.2 - 33.2)	11.1 (10.6 - 11.6)
Panama	*E. coli*	6.5 (5.4-7.7)	12.9 (10.8-15.1)	6.2 (5.4 - 7.1)	12.2 (10.7 - 13.8)	5.3 (4.8 - 5.8)	11.0 (10.1 - 12.0)
	Group B *Streptococcus*	24.2 (22.0-26.6)	27.3 (24.8-30.0)	10.4 (9.6 - 11.1)	12.2 (11.3 - 13.1)	7.8 (7.5 - 8.2)	8.8 (8.4 - 9.3)
	H. influenzae	8.0 (6.5-9.6)	5.0 (4.0-6.1)	9.5 (8.9 - 10.2)	5.7 (5.2 - 6.2)	6.2 (5.8 - 6.5)	3.5 (3.3 - 3.8)
	K. pneumoniae	5.8 (4.6-7.2)	19.7 (16.2-23.5)	3.9 (3.2 - 4.7)	13.6 (11.6 - 16.1)	4.0 (3.5 - 4.5)	13.7 (12.4 - 15.1)
	L. monocytogenes	1.9 (1.3-2.6)	4.9 (3.5-6.7)	2.4 (1.8 - 3.0)	6.2 (4.8 - 7.7)	2.4 (2.1 - 2.7)	6.7 (6.0 - 7.3)
	N. meningitidis	7.7 (6.6-8.7)	6.6 (5.7-7.6)	12.4 (11.6 - 13.2)	10.3 (9.5 - 11.1)	12.9 (12.4 - 13.4)	10.4 (9.9 - 11.0)
	Other	2.1 (1.3-3.2)	2.7 (1.7-4.2)	10.1 (7.0 - 14.0)	11.7 (8.1 - 16.1)	10.3 (8.2 - 12.7)	12.7 (10.2 - 15.5)
	S. aureus	4.4 (3.5-5.3)	5.8 (4.7-7.0)	5.3 (4.8 - 5.8)	6.9 (6.1 - 7.6)	5.4 (5.1 - 5.7)	7.4 (7.0 - 7.9)
	S. pneumoniae	2.9 (2.4-3.4)	3.0 (2.5-3.6)	13.1 (12.2 - 14.0)	12.8 (11.9 - 13.8)	13.3 (12.8 - 13.9)	14.8 (14.1 - 15.5)
	Virus	36.7 (33.8-39.6)	12.1 (10.7-13.5)	26.7 (25.4 - 28.0)	8.6 (8.0 - 9.1)	32.4 (31.4 - 33.3)	10.8 (10.4 - 11.3)
Venezuela (Bolivarian Republic of)	*E. coli*	6.8 (5.7-7.9)	13.2 (11.2-15.4)	5.8 (5.0 - 6.6)	11.1 (9.8 - 12.4)	5.0 (4.5 - 5.6)	9.9 (9.0 - 10.8)
	Group B *Streptococcus*	24.6 (22.9-26.2)	27.5 (25.6-29.3)	9.6 (9.0 - 10.1)	11.8 (11.1 - 12.5)	7.1 (6.8 - 7.5)	7.9 (7.5 - 8.3)
	H. influenzae	7.9 (6.8-9.3)	5.0 (4.1-5.8)	10.9 (10.3 - 11.6)	6.2 (5.8 - 6.6)	6.8 (6.5 - 7.1)	3.6 (3.4 - 3.8)
	K. pneumoniae	5.3 (4.2-6.7)	17.8 (14.6-21.6)	3.8 (3.2 - 4.7)	13.0 (11.1 - 15.3)	4.0 (3.5 - 4.5)	13.3 (12.0 - 14.7)
	L. monocytogenes	1.8 (1.3-2.5)	4.7 (3.4-6.4)	2.2 (1.7 - 2.8)	5.5 (4.3 - 6.8)	2.3 (2.0 - 2.5)	6.0 (5.4 - 6.6)
	N. meningitidis	8.6 (7.7-9.6)	7.3 (6.5-8.2)	15.0 (14.3 - 15.8)	11.7 (10.9 - 12.4)	15.3 (14.8 - 15.8)	12.0 (11.5 - 12.5)
	Other	2.0 (1.2-3.1)	2.6 (1.6-4.0)	9.6 (6.8 - 13.3)	10.3 (7.3 - 14.0)	10.1 (8.2 - 12.3)	12.0 (9.9 - 14.5)
	S. aureus	4.1 (3.3-5.0)	5.4 (4.4-6.6)	4.7 (4.2 - 5.1)	5.8 (5.3 - 6.3)	4.9 (4.6 - 5.1)	6.5 (6.1 - 6.9)

Location	Aetiology	Neonatal		Under 5		All Ages	
		Nonfatal	Fatal	Nonfatal	Fatal	Nonfatal	Fatal
	S. pneumoniae	2.8 (2.4-3.3)	4.3 (3.7-5.1)	12.2 (11.6 - 12.8)	16.3 (15.3 - 17.3)	12.2 (11.8 - 12.5)	18.2 (17.5 - 18.9)
	Virus	36.0 (33.4-38.6)	12.0 (10.8-13.4)	26.0 (24.8 - 27.1)	8.4 (7.9 - 8.9)	32.4 (31.5 - 33.2)	10.6 (10.2 - 11.0)
**Tropical Latin America**	*E. coli*	6.5 (5.3-7.9)	12.7 (10.3-15.3)	6.1 (5.2 - 7.1)	11.9 (10.2 - 13.7)	4.7 (4.2 - 5.3)	10.4 (9.2 - 11.5)
	Group B *Streptococcus*	24.6 (21.9-27.7)	27.7 (24.5-31.0)	10.1 (9.3 - 10.9)	11.2 (10.2 - 12.2)	6.8 (6.4 - 7.2)	8.5 (8.0 - 9.0)
	H. influenzae	7.8 (6.1-9.7)	5.0 (3.8-6.2)	7.9 (7.3 - 8.6)	4.8 (4.4 - 5.3)	4.0 (3.7 - 4.2)	2.9 (2.7 - 3.1)
	K. pneumoniae	6.0 (4.7-7.5)	19.8 (16.0-24.0)	3.8 (3.1 - 4.7)	12.8 (10.6 - 15.5)	3.9 (3.4 - 4.4)	13.3 (11.7 - 14.9)
	L. monocytogenes	1.9 (1.3-2.7)	4.8 (3.4-6.8)	2.4 (1.8 - 3.0)	6.1 (4.6 - 7.7)	2.2 (2.0 - 2.5)	6.3 (5.5 - 7.1)
	N. meningitidis	7.1 (5.9-8.2)	6.0 (5.0-7.1)	13.5 (12.5 - 14.3)	11.3 (10.4 - 12.3)	14.3 (13.7 - 14.9)	11.8 (11.1 - 12.4)
	Other	2.2 (1.3-3.3)	2.8 (1.7-4.3)	10.1 (7.0 - 14.1)	12.1 (8.4 - 16.6)	10.1 (8.3 - 12.2)	12.7 (10.4 - 15.4)
	S. aureus	4.4 (3.5-5.5)	5.8 (4.6-7.2)	5.5 (4.9 - 6.1)	7.1 (6.3 - 7.9)	5.4 (5.0 - 5.8)	7.6 (7.0 - 8.2)
	S. pneumoniae	2.8 (2.3-3.4)	2.8 (2.3-3.5)	14.7 (13.8 - 15.8)	14.2 (13.1 - 15.5)	14.7 (14.1 - 15.3)	15.5 (14.8 - 16.4)
	Virus	36.8 (33.6-39.9)	12.6 (11.1-14.2)	26.0 (24.6 - 27.3)	8.5 (7.9 - 9.1)	33.9 (32.8 - 34.9)	11.0 (10.5 - 11.5)
Brazil	*E. coli*	6.5 (5.3-7.9)	12.7 (10.3-15.3)	6.1 (5.2 - 7.1)	11.9 (10.2 - 13.7)	4.7 (4.2 - 5.3)	10.4 (9.2 - 11.5)
	Group B *Streptococcus*	24.6 (21.9-27.7)	27.7 (24.5-31.0)	10.1 (9.3 - 10.8)	11.2 (10.2 - 12.2)	6.8 (6.4 - 7.2)	8.5 (8.0 - 9.0)
	H. influenzae	7.8 (6.1-9.7)	5.0 (3.8-6.2)	7.9 (7.3 - 8.6)	4.8 (4.4 - 5.3)	3.9 (3.7 - 4.2)	2.9 (2.7 - 3.1)
	K. pneumoniae	6.0 (4.7-7.5)	19.8 (16.0-24.0)	3.8 (3.1 - 4.6)	12.8 (10.6 - 15.4)	3.9 (3.4 - 4.4)	13.3 (11.7 - 14.9)
	L. monocytogenes	1.9 (1.3-2.7)	4.8 (3.4-6.8)	2.4 (1.8 - 3.0)	6.1 (4.6 - 7.7)	2.2 (2.0 - 2.5)	6.3 (5.6 - 7.1)
	N. meningitidis	7.0 (5.9-8.2)	6.0 (5.0-7.1)	13.4 (12.5 - 14.3)	11.3 (10.4 - 12.3)	14.3 (13.7 - 14.9)	11.8 (11.1 - 12.4)
	Other	2.2 (1.3-3.3)	2.8 (1.7-4.3)	10.1 (7.0 - 14.1)	12.1 (8.4 - 16.6)	10.1 (8.2 - 12.2)	12.7 (10.4 - 15.4)
	S. aureus	4.4 (3.5-5.5)	5.8 (4.6-7.2)	5.5 (4.9 - 6.1)	7.1 (6.3 - 7.9)	5.4 (5.0 - 5.8)	7.6 (7.0 - 8.2)
	S. pneumoniae	2.8 (2.3-3.4)	2.8 (2.3-3.4)	14.8 (13.8 - 15.8)	14.2 (13.1 - 15.5)	14.7 (14.1 - 15.3)	15.5 (14.7 - 16.4)
	Virus	36.8 (33.6-39.9)	12.6 (11.1-14.2)	26.0 (24.6 - 27.3)	8.5 (7.9 - 9.1)	33.9 (32.8 - 34.9)	11.0 (10.5 - 11.5)
Paraguay	*E. coli*	6.5 (5.3-7.9)	12.8 (10.4-15.3)	6.1 (5.2 - 7.1)	11.8 (10.1 - 13.6)	5.0 (4.4 - 5.6)	10.2 (9.1 - 11.3)
	Group B *Streptococcus*	24.5 (22.0-27.3)	27.5 (24.5-30.5)	10.2 (9.4 - 10.9)	11.4 (10.4 - 12.3)	7.6 (7.1 - 8.0)	8.3 (7.8 - 8.9)
	H. influenzae	7.9 (6.3-9.6)	5.0 (3.9-6.2)	7.9 (7.2 - 8.5)	4.7 (4.3 - 5.2)	5.0 (4.7 - 5.4)	2.9 (2.7 - 3.1)
	K. pneumoniae	5.9 (4.6-7.4)	19.4 (15.8-23.6)	3.8 (3.1 - 4.7)	12.9 (10.7 - 15.5)	3.8 (3.3 - 4.4)	13.2 (11.6 - 14.8)
	L. monocytogenes	1.9 (1.3-2.7)	4.8 (3.4-6.8)	2.3 (1.8 - 3.0)	5.9 (4.6 - 7.5)	2.2 (1.9 - 2.5)	6.2 (5.5 - 6.9)
	N. meningitidis	7.3 (6.2-8.4)	6.2 (5.2-7.3)	13.8 (12.8 - 14.7)	11.4 (10.4 - 12.4)	14.8 (14.2 - 15.4)	12.0 (11.4 - 12.6)
	Other	2.1 (1.3-3.3)	2.8 (1.7-4.3)	10.0 (6.9 - 13.9)	11.7 (8.2 - 16.0)	10.0 (8.1 - 12.2)	12.5 (10.2 - 15.0)
	S. aureus	4.3 (3.5-5.4)	5.7 (4.6-7.1)	5.4 (4.8 - 6.0)	7.0 (6.1 - 7.8)	5.3 (4.9 - 5.7)	7.4 (6.9 - 8.0)

Location	Aetiology	Neonatal		Under 5		All Ages	
		Nonfatal	Fatal	Nonfatal	Fatal	Nonfatal	Fatal
	S. pneumoniae	2.8 (2.3-3.4)	3.0 (2.5-3.7)	14.3 (13.4 - 15.4)	14.6 (13.5 - 15.9)	14.2 (13.6 - 14.8)	16.2 (15.4 - 17.0)
	Virus	36.7 (33.6-39.8)	12.8 (11.3-14.3)	26.2 (24.8 - 27.5)	8.7 (8.1 - 9.3)	32.1 (31.2 - 33.1)	11.1 (10.6 - 11.6)
**North Africa and Middle East**	*E. coli*	6.4 (5.2-7.7)	12.3 (10.0-14.8)	5.8 (4.9 - 6.7)	10.5 (9.1 - 12.2)	5.0 (4.4 - 5.6)	9.8 (8.7 - 11.0)
	Group B *Streptococcus*	22.3 (20.4-24.4)	24.4 (21.9-26.7)	9.2 (8.6 - 9.8)	11.4 (10.5 - 12.2)	7.0 (6.6 - 7.3)	8.3 (7.8 - 8.8)
	H. influenzae	8.8 (7.6-10.2)	6.0 (5.0-7.1)	9.7 (9.1 - 10.3)	6.1 (5.7 - 6.6)	6.4 (6.1 - 6.7)	4.0 (3.8 - 4.3)
	K. pneumoniae	5.4 (4.3-6.8)	17.6 (14.3-21.6)	3.8 (3.1 - 4.7)	12.5 (10.4 - 15.1)	3.9 (3.4 - 4.5)	12.9 (11.2 - 14.6)
	L. monocytogenes	1.9 (1.3-2.6)	4.7 (3.3-6.6)	2.2 (1.7 - 2.8)	5.1 (4.0 - 6.5)	2.2 (1.9 - 2.6)	5.7 (5.0 - 6.5)
	N. meningitidis	9.1 (8.1-10.2)	8.1 (7.0-9.3)	15.7 (14.9 - 16.6)	13.0 (11.9 - 14.2)	16.0 (15.5 - 16.6)	12.8 (12.1 - 13.5)
	Other	2.2 (1.3-3.3)	2.9 (1.8-4.3)	9.7 (6.8 - 13.3)	9.9 (7.1 - 13.4)	9.9 (8.1 - 12.0)	11.5 (9.5 - 13.8)
	S. aureus	4.3 (3.4-5.3)	5.6 (4.4-7.1)	5.0 (4.5 - 5.4)	6.2 (5.6 - 7.0)	5.1 (4.8 - 5.4)	6.8 (6.3 - 7.3)
	S. pneumoniae	2.9 (2.5-3.4)	4.3 (3.6-5.1)	13.2 (12.5 - 13.9)	15.6 (14.6 - 16.7)	12.8 (12.3 - 13.2)	16.9 (16.2 - 17.8)
	Virus	36.7 (33.9-39.5)	14.1 (12.5-15.9)	25.8 (24.6 - 26.9)	9.6 (9.0 - 10.2)	31.7 (30.8 - 32.5)	11.3 (10.8 - 11.7)
Afghanistan	*E. coli*	6.1 (4.6-7.9)	11.7 (8.9-14.7)	5.1 (4.0 - 6.3)	9.5 (7.8 - 11.4)	4.3 (3.5 - 5.2)	8.7 (7.2 - 10.3)
	Group B *Streptococcus*	19.3 (16.7-22.2)	21.8 (19.0-24.8)	8.4 (7.5 - 9.1)	10.0 (9.0 - 10.9)	6.0 (5.4 - 6.6)	8.2 (7.4 - 8.9)
	H. influenzae	9.9 (8.5-11.6)	6.8 (5.7-8.1)	10.5 (9.8 - 11.3)	6.7 (6.2 - 7.2)	6.3 (5.9 - 6.7)	5.1 (4.8 - 5.5)
	K. pneumoniae	5.4 (4.0-7.1)	16.9 (13.1-21.5)	3.6 (2.8 - 4.7)	11.5 (9.1 - 14.3)	3.7 (2.9 - 4.6)	11.5 (9.3 - 14.0)
	L. monocytogenes	1.9 (1.3-2.7)	4.6 (3.1-6.7)	1.9 (1.4 - 2.5)	4.6 (3.5 - 5.8)	1.9 (1.5 - 2.4)	4.6 (3.7 - 5.7)
	N. meningitidis	10.2 (8.7-11.9)	8.8 (7.4-10.5)	20.6 (19.4 - 21.9)	16.2 (15.0 - 17.5)	21.7 (20.7 - 22.7)	17.3 (16.3 - 18.4)
	Other	2.4 (1.5-3.6)	3.1 (1.9-4.6)	8.6 (6.1 - 12.0)	9.3 (6.6 - 12.8)	8.9 (7.3 - 10.7)	10.0 (8.1 - 12.6)
	S. aureus	4.3 (3.2-5.7)	5.7 (4.2-7.5)	4.9 (4.3 - 5.6)	6.1 (5.3 - 6.9)	5.0 (4.4 - 5.6)	6.3 (5.5 - 7.1)
	S. pneumoniae	3.1 (2.5-3.8)	4.6 (3.8-5.6)	12.3 (11.5 - 13.2)	16.2 (15.0 - 17.5)	12.5 (11.9 - 13.2)	17.1 (16.0 - 18.2)
	Virus	37.3 (34.3-40.7)	16.1 (14.3-18.1)	24.0 (22.9 - 25.2)	10.0 (9.5 - 10.7)	29.6 (28.6 - 30.6)	11.3 (10.7 - 11.9)
Algeria	*E. coli*	6.7 (5.6-7.9)	13.1 (11.1-15.3)	6.3 (5.4 - 7.2)	12.1 (10.7 - 13.6)	5.5 (4.9 - 6.0)	10.7 (9.7 - 11.6)
	Group B *Streptococcus*	25.0 (23.1-27.0)	28.0 (25.7-30.2)	10.0 (9.3 - 10.7)	13.1 (12.2 - 13.9)	7.7 (7.3 - 8.1)	8.2 (7.8 - 8.6)
	H. influenzae	7.8 (6.4-9.3)	4.9 (3.9-5.9)	8.9 (8.2 - 9.5)	5.1 (4.7 - 5.6)	5.9 (5.6 - 6.3)	3.1 (2.9 - 3.2)
	K. pneumoniae	5.6 (4.5-6.9)	18.8 (15.4-22.5)	3.9 (3.2 - 4.8)	13.7 (11.7 - 16.0)	4.0 (3.6 - 4.5)	13.3 (11.9 - 14.5)
	L. monocytogenes	1.9 (1.3-2.6)	4.8 (3.4-6.6)	2.4 (1.8 - 3.0)	5.9 (4.7 - 7.3)	2.4 (2.1 - 2.8)	6.5 (5.9 - 7.2)
	N. meningitidis	7.8 (6.8-8.7)	6.6 (5.8-7.6)	13.3 (12.4 - 14.2)	10.3 (9.5 - 11.1)	13.8 (13.2 - 14.3)	10.8 (10.3 - 11.3)
	Other	2.0 (1.3-3.2)	2.6 (1.6-4.1)	10.2 (7.1 - 14.3)	10.6 (7.5 - 14.6)	10.3 (8.2 - 12.6)	12.1 (9.8 - 14.8)
	S. aureus	4.2 (3.4-5.1)	5.5 (4.5-6.7)	5.2 (4.7 - 5.8)	6.5 (5.8 - 7.1)	5.3 (5.0 - 5.6)	7.0 (6.6 - 7.5)

Location	Aetiology	Neonatal		Under 5		All Ages	
		Nonfatal	Fatal	Nonfatal	Fatal	Nonfatal	Fatal
	S. pneumoniae	2.8 (2.4-3.2)	3.6 (3.0-4.1)	13.0 (12.1 - 14.0)	13.9 (12.9 - 15.1)	13.3 (12.7 - 13.8)	17.2 (16.4 - 18.0)
	Virus	36.2 (33.5-39.0)	12.1 (10.7-13.5)	26.8 (25.4 - 28.0)	8.8 (8.2 - 9.3)	31.8 (30.9 - 32.7)	11.1 (10.6 - 11.5)
Bahrain	*E. coli*	6.4 (5.2-7.9)	12.7 (10.3-15.5)	6.5 (5.6 - 7.4)	13.0 (11.4 - 14.8)	5.1 (4.6 - 5.6)	11.2 (10.2 - 12.2)
	Group B *Streptococcus*	24.9 (21.7-28.3)	28.1 (24.2-32.1)	10.8 (9.8 - 11.6)	13.0 (11.8 - 14.1)	7.5 (7.2 - 7.9)	8.8 (8.3 - 9.3)
	H. influenzae	7.7 (5.8-9.8)	4.7 (3.5-6.2)	7.9 (7.2 - 8.6)	4.8 (4.3 - 5.3)	4.4 (4.1 - 4.7)	2.6 (2.4 - 2.9)
	K. pneumoniae	6.2 (4.8-8.0)	21.2 (16.9-26.3)	3.8 (3.1 - 4.6)	14.0 (11.7 - 16.6)	4.0 (3.6 - 4.4)	14.4 (13.0 - 15.8)
	L. monocytogenes	1.9 (1.3-2.7)	5.0 (3.5-7.1)	2.5 (1.9 - 3.2)	6.7 (5.1 - 8.5)	2.4 (2.2 - 2.7)	7.3 (6.6 - 8.0)
	N. meningitidis	6.6 (5.4-7.9)	5.7 (4.6-6.8)	10.7 (9.9 - 11.5)	9.1 (8.2 - 10.0)	11.3 (10.7 - 11.8)	9.5 (9.0 - 10.2)
	Other	2.1 (1.2-3.3)	2.7 (1.6-4.2)	10.6 (7.2 - 15.0)	12.6 (8.7 - 17.6)	10.5 (8.4 - 12.9)	13.7 (10.9 - 17.0)
	S. aureus	4.5 (3.6-5.7)	6.0 (4.8-7.5)	5.7 (5.1 - 6.3)	7.6 (6.7 - 8.5)	5.7 (5.4 - 6.1)	8.3 (7.7 - 8.9)
	S. pneumoniae	2.8 (2.1-3.6)	2.2 (1.7-2.8)	14.8 (13.7 - 16.0)	10.8 (9.8 - 11.8)	14.8 (14.1 - 15.5)	12.7 (12.0 - 13.5)
	Virus	36.8 (33.6-40.1)	11.7 (10.2-13.2)	26.8 (25.2 - 28.1)	8.4 (7.8 - 9.1)	34.2 (33.0 - 35.3)	11.4 (10.9 - 12.0)
Egypt	*E. coli*	6.6 (5.4-7.9)	12.9 (10.7-15.3)	5.6 (4.8 - 6.5)	10.9 (9.7 - 12.2)	4.9 (4.3 - 5.5)	9.4 (8.4 - 10.4)
	Group B *Streptococcus*	22.5 (21.0-24.2)	25.4 (23.6-27.2)	8.7 (8.1 - 9.3)	13.0 (12.2 - 13.7)	6.8 (6.4 - 7.1)	8.2 (7.8 - 8.6)
	H. influenzae	8.6 (7.5-9.8)	5.5 (4.7-6.4)	9.3 (8.6 - 9.9)	5.4 (5.0 - 5.8)	6.9 (6.6 - 7.3)	3.9 (3.7 - 4.1)
	K. pneumoniae	5.2 (4.1-6.6)	17.2 (14.0-21.0)	3.8 (3.1 - 4.7)	13.2 (11.3 - 15.4)	3.9 (3.4 - 4.6)	13.2 (11.7 - 14.8)
	L. monocytogenes	1.9 (1.3-2.5)	4.7 (3.3-6.3)	2.2 (1.6 - 2.8)	5.1 (4.1 - 6.3)	2.2 (1.9 - 2.5)	5.4 (4.8 - 6.1)
	N. meningitidis	9.8 (8.9-10.8)	8.4 (7.5-9.4)	17.3 (16.3 - 18.2)	12.4 (11.6 - 13.1)	17.1 (16.6 - 17.7)	13.5 (13.0 - 14.1)
	Other	2.1 (1.2-3.2)	2.6 (1.6-4.1)	9.6 (6.7 - 13.3)	8.8 (6.4 - 11.9)	9.9 (8.1 - 12.0)	11.3 (9.5 - 13.4)
	S. aureus	4.1 (3.3-5.1)	5.4 (4.4-6.7)	4.6 (4.2 - 5.1)	5.6 (5.1 - 6.2)	4.8 (4.5 - 5.1)	6.2 (5.8 - 6.6)
	S. pneumoniae	2.9 (2.5-3.4)	4.9 (4.2-5.7)	13.3 (12.5 - 14.0)	16.5 (15.4 - 17.6)	12.1 (11.6 - 12.5)	17.7 (17.0 - 18.4)
	Virus	36.2 (33.7-38.9)	12.8 (11.5-14.2)	25.5 (24.3 - 26.7)	9.2 (8.7 - 9.7)	31.4 (30.5 - 32.3)	11.1 (10.7 - 11.5)
Iran (Islamic Republic of)	*E. coli*	6.8 (5.6-7.9)	13.3 (11.3-15.4)	6.0 (5.1 - 6.8)	11.4 (10.2 - 12.7)	5.2 (4.7 - 5.6)	10.6 (9.7 - 11.6)
	Group B *Streptococcus*	23.9 (22.7-25.2)	26.9 (25.5-28.4)	9.0 (8.4 - 9.6)	12.4 (11.8 - 13.1)	6.6 (6.3 - 6.9)	7.2 (6.9 - 7.5)
	H. influenzae	8.1 (7.1-9.4)	5.0 (4.3-5.9)	9.7 (9.0 - 10.4)	5.4 (4.9 - 5.8)	6.7 (6.4 - 7.0)	3.6 (3.4 - 3.8)
	K. pneumoniae	5.2 (4.2-6.5)	17.8 (14.6-21.5)	3.9 (3.2 - 4.7)	13.5 (11.6 - 15.7)	4.1 (3.7 - 4.5)	13.4 (12.2 - 14.6)
	L. monocytogenes	1.8 (1.3-2.5)	4.8 (3.5-6.4)	2.3 (1.8 - 3.0)	5.6 (4.5 - 6.9)	2.4 (2.1 - 2.7)	6.7 (6.1 - 7.3)
	N. meningitidis	9.3 (8.5-10.2)	8.0 (7.1-8.9)	14.9 (14.0 - 15.7)	11.1 (10.4 - 11.8)	14.4 (13.9 - 14.8)	10.4 (10.0 - 10.8)
	Other	1.9 (1.2-3.0)	2.5 (1.5-3.9)	10.2 (7.2 - 14.2)	10.0 (7.1 - 13.6)	10.4 (8.5 - 12.7)	12.2 (9.6 - 15.2)
	S. aureus	4.1 (3.3-5.0)	5.4 (4.4-6.6)	4.7 (4.2 - 5.1)	5.7 (5.2 - 6.2)	4.9 (4.6 - 5.1)	6.6 (6.3 - 7.0)

Location	Aetiology	Neonatal		Under 5		All Ages	
		Nonfatal	Fatal	Nonfatal	Fatal	Nonfatal	Fatal
	S. pneumoniae	2.9 (2.5-3.3)	4.6 (3.9-5.3)	13.1 (12.3 - 13.9)	16.6 (15.3 - 17.6)	11.6 (11.2 - 11.9)	18.2 (17.3 - 18.9)
	Virus	35.9 (33.4-38.3)	11.6 (10.4-12.9)	26.3 (25.0 - 27.5)	8.3 (7.9 - 8.8)	33.8 (32.8 - 34.7)	11.0 (10.5 - 11.5)
Iraq	*E. coli*	6.6 (5.4-7.8)	12.9 (10.7-15.1)	5.9 (5.1 - 6.7)	11.4 (10.2 - 12.7)	5.2 (4.6 - 5.7)	10.0 (9.1 - 11.0)
	Group B *Streptococcus*	22.4 (21.1-23.8)	25.3 (23.7-26.9)	9.2 (8.6 - 9.7)	13.0 (12.3 - 13.7)	7.2 (6.9 - 7.6)	8.5 (8.1 - 8.9)
	H. influenzae	8.7 (7.6-9.9)	5.5 (4.7-6.4)	11.7 (11.0 - 12.3)	6.6 (6.1 - 7.0)	8.4 (8.1 - 8.8)	4.4 (4.2 - 4.6)
	K. pneumoniae	5.3 (4.2-6.6)	17.7 (14.5-21.4)	3.9 (3.2 - 4.7)	13.7 (11.7 - 15.9)	4.0 (3.5 - 4.5)	13.6 (12.1 - 15.0)
	L. monocytogenes	1.9 (1.3-2.5)	4.8 (3.4-6.5)	2.2 (1.7 - 2.8)	5.4 (4.4 - 6.6)	2.2 (1.9 - 2.6)	5.8 (5.2 - 6.5)
	N. meningitidis	9.8 (8.9-10.7)	8.4 (7.5-9.4)	15.0 (14.2 - 15.8)	11.4 (10.7 - 12.1)	15.3 (14.9 - 15.9)	12.3 (11.8 - 12.8)
	Other	2.0 (1.2-3.1)	2.6 (1.6-4.1)	9.7 (6.8 - 13.4)	9.5 (6.8 - 12.9)	10.0 (8.0 - 12.3)	11.6 (9.6 - 14.0)
	S. aureus	4.2 (3.4-5.1)	5.5 (4.5-6.7)	4.8 (4.3 - 5.2)	5.9 (5.4 - 6.5)	4.9 (4.6 - 5.2)	6.5 (6.1 - 6.9)
	S. pneumoniae	3.0 (2.6-3.4)	4.6 (4.0-5.3)	11.5 (10.9 - 12.0)	14.1 (13.2 - 14.9)	11.2 (10.9 - 11.6)	16.2 (15.6 - 16.9)
	Virus	36.3 (33.8-38.9)	12.5 (11.2-13.9)	26.2 (25.0 - 27.4)	9.2 (8.6 - 9.7)	31.5 (30.6 - 32.3)	11.0 (10.6 - 11.5)
Jordan	*E. coli*	7.1 (6.0-8.4)	13.7 (11.7-15.9)	6.0 (5.2 - 6.8)	11.2 (10.0 - 12.5)	5.2 (4.7 - 5.7)	10.1 (9.3 - 11.0)
	Group B *Streptococcus*	28.5 (26.3-30.8)	31.4 (28.8-33.8)	9.9 (9.2 - 10.6)	13.2 (12.4 - 14.0)	7.4 (7.0 - 7.7)	8.7 (8.2 - 9.1)
	H. influenzae	6.8 (5.4-8.5)	4.1 (3.2-5.3)	6.6 (6.1 - 7.2)	3.7 (3.4 - 4.0)	4.4 (4.1 - 4.8)	2.5 (2.3 - 2.6)
	K. pneumoniae	5.4 (4.2-6.9)	17.8 (14.4-22.0)	3.8 (3.2 - 4.7)	13.0 (11.1 - 15.1)	4.0 (3.5 - 4.4)	13.1 (11.9 - 14.4)
	L. monocytogenes	1.8 (1.3-2.5)	4.6 (3.2-6.2)	2.3 (1.8 - 3.0)	5.5 (4.3 - 6.8)	2.3 (2.1 - 2.6)	6.0 (5.5 - 6.6)
	N. meningitidis	6.9 (6.0-8.0)	5.8 (4.9-6.7)	15.2 (14.3 - 16.0)	10.9 (10.2 - 11.6)	15.1 (14.5 - 15.6)	11.3 (10.8 - 11.8)
	Other	1.9 (1.2-3.1)	2.4 (1.5-3.8)	10.0 (7.0 - 13.9)	9.8 (6.9 - 13.4)	10.2 (8.2 - 12.5)	11.6 (9.5 - 14.1)
	S. aureus	3.9 (3.1-4.9)	5.1 (4.1-6.2)	4.7 (4.2 - 5.1)	5.5 (5.0 - 6.1)	4.8 (4.6 - 5.1)	6.2 (5.9 - 6.6)
	S. pneumoniae	2.6 (2.2-3.1)	4.0 (3.3-4.8)	15.5 (14.5 - 16.4)	19.3 (17.9 - 20.7)	14.2 (13.6 - 14.8)	20.6 (19.6 - 21.5)
	Virus	35.0 (32.0-37.9)	11.1 (9.8-12.4)	25.9 (24.6 - 27.1)	7.9 (7.4 - 8.3)	32.4 (31.4 - 33.3)	9.9 (9.6 - 10.3)
Kuwait	*E. coli*	6.4 (5.1-8.0)	12.8 (10.2-15.7)	6.6 (5.8 - 7.5)	13.5 (12.0 - 15.1)	5.5 (4.9 - 6.0)	12.0 (11.0 - 12.9)
	Group B *Streptococcus*	25.0 (21.5-28.6)	28.3 (24.3-32.5)	11.3 (10.4 - 12.3)	15.6 (14.3 - 17.0)	8.3 (7.8 - 8.8)	10.0 (9.4 - 10.6)
	H. influenzae	7.6 (5.7-9.8)	4.6 (3.3-6.1)	8.4 (7.6 - 9.2)	5.0 (4.4 - 5.5)	5.2 (4.8 - 5.6)	3.0 (2.7 - 3.2)
	K. pneumoniae	6.3 (4.8-8.2)	22.0 (17.5-27.2)	3.9 (3.2 - 4.7)	15.5 (13.3 - 17.9)	4.0 (3.6 - 4.4)	15.0 (13.7 - 16.5)
	L. monocytogenes	1.9 (1.3-2.8)	5.1 (3.5-7.2)	2.6 (2.0 - 3.3)	6.8 (5.3 - 8.4)	2.5 (2.2 - 2.8)	7.6 (6.9 - 8.3)
	N. meningitidis	6.5 (5.2-7.8)	5.6 (4.5-6.8)	9.4 (8.6 - 10.2)	7.8 (7.1 - 8.6)	9.9 (9.3 - 10.5)	8.4 (7.9 - 9.0)
	Other	2.0 (1.2-3.2)	2.7 (1.6-4.2)	10.6 (7.2 - 15.2)	11.5 (8.0 - 16.1)	10.6 (8.3 - 13.5)	13.8 (10.8 - 17.2)
	S. aureus	4.6 (3.6-5.8)	6.1 (4.8-7.8)	5.7 (5.1 - 6.4)	7.5 (6.7 - 8.4)	5.7 (5.3 - 6.1)	8.4 (7.8 - 9.0)

Location	Aetiology	Neonatal		Under 5		All Ages	
		Nonfatal	Fatal	Nonfatal	Fatal	Nonfatal	Fatal
	S. pneumoniae	2.8 (2.1-3.7)	1.9 (1.4-2.5)	14.3 (13.2 - 15.5)	8.3 (7.5 - 9.1)	14.3 (13.5 - 15.1)	10.8 (10.1 - 11.5)
	Virus	36.8 (33.6-40.2)	11.1 (9.7-12.6)	27.2 (25.6 - 28.7)	8.6 (8.0 - 9.2)	33.9 (32.6 - 35.1)	11.0 (10.5 - 11.6)
Lebanon	*E. coli*	6.8 (5.8-7.8)	13.5 (11.7-15.5)	6.5 (5.7 - 7.2)	12.7 (11.4 - 14.0)	5.8 (5.4 - 6.2)	11.8 (10.8 - 12.8)
	Group B *Streptococcus*	26.1 (24.5-27.8)	29.4 (27.4-31.2)	10.2 (9.5 - 10.9)	13.8 (12.9 - 14.6)	7.6 (7.3 - 7.9)	7.9 (7.5 - 8.3)
	H. influenzae	7.4 (6.1-8.9)	4.5 (3.6-5.4)	10.4 (9.7 - 11.2)	5.7 (5.3 - 6.2)	6.3 (5.9 - 6.6)	3.0 (2.9 - 3.2)
	K. pneumoniae	5.6 (4.4-6.9)	19.2 (15.8-22.9)	3.9 (3.3 - 4.7)	14.4 (12.4 - 16.5)	4.2 (3.8 - 4.6)	13.8 (12.7 - 14.9)
	L. monocytogenes	1.9 (1.3-2.5)	4.8 (3.5-6.5)	2.5 (1.9 - 3.2)	6.3 (5.0 - 7.7)	2.7 (2.4 - 3.0)	7.6 (7.0 - 8.2)
	N. meningitidis	7.6 (6.7-8.5)	6.5 (5.7-7.3)	11.4 (10.7 - 12.1)	9.0 (8.3 - 9.7)	11.5 (11.1 - 11.9)	8.5 (8.1 - 8.9)
	Other	1.9 (1.1-3.1)	2.5 (1.5-3.9)	10.6 (7.3 - 14.9)	11.0 (7.7 - 15.3)	10.7 (8.3 - 13.5)	12.7 (9.6 - 16.5)
	S. aureus	4.2 (3.4-5.1)	5.5 (4.5-6.6)	5.0 (4.6 - 5.5)	6.3 (5.7 - 6.9)	5.3 (5.0 - 5.5)	7.2 (6.8 - 7.6)
	S. pneumoniae	2.8 (2.4-3.2)	3.4 (2.9-4.0)	12.2 (11.4 - 13.0)	12.7 (11.8 - 13.6)	12.6 (12.1 - 13.0)	16.7 (15.8 - 17.4)
	Virus	35.8 (33.2-38.5)	10.8 (9.6-12.0)	27.2 (25.7 - 28.5)	8.2 (7.6 - 8.7)	33.4 (32.3 - 34.4)	10.8 (10.3 - 11.3)
Libya	*E. coli*	6.6 (5.5-7.7)	12.9 (10.8-15.1)	6.1 (5.2 - 7.1)	11.8 (10.3 - 13.5)	5.2 (4.6 - 5.7)	10.2 (9.2 - 11.3)
	Group B *Streptococcus*	24.1 (22.1-26.2)	27.1 (24.8-29.5)	9.7 (9.1 - 10.4)	11.7 (10.8 - 12.6)	6.9 (6.6 - 7.3)	7.4 (7.0 - 7.8)
	H. influenzae	8.1 (6.7-9.6)	5.1 (4.1-6.1)	9.2 (8.6 - 9.9)	5.4 (5.0 - 5.9)	5.3 (5.1 - 5.6)	2.9 (2.7 - 3.1)
	K. pneumoniae	5.7 (4.5-7.0)	19.0 (15.6-22.7)	3.9 (3.2 - 4.7)	13.3 (11.2 - 15.7)	4.0 (3.5 - 4.5)	13.3 (11.9 - 14.6)
	L. monocytogenes	1.9 (1.3-2.6)	4.8 (3.4-6.7)	2.4 (1.8 - 3.0)	5.9 (4.6 - 7.4)	2.4 (2.2 - 2.7)	6.5 (5.9 - 7.2)
	N. meningitidis	7.9 (7.0-8.9)	6.8 (5.9-7.8)	13.6 (12.7 - 14.5)	11.0 (10.1 - 11.8)	14.2 (13.7 - 14.6)	11.3 (10.8 - 11.9)
	Other	2.1 (1.3-3.3)	2.7 (1.7-4.2)	10.2 (7.1 - 14.2)	11.3 (7.9 - 15.5)	10.2 (8.4 - 12.4)	12.5 (10.2 - 15.1)
	S. aureus	4.3 (3.5-5.3)	5.7 (4.6-6.9)	5.3 (4.7 - 5.8)	6.6 (5.9 - 7.3)	5.4 (5.0 - 5.7)	7.3 (6.8 - 7.8)
	S. pneumoniae	2.9 (2.4-3.3)	3.4 (2.9-4.1)	13.2 (12.3 - 14.1)	14.3 (13.2 - 15.4)	13.3 (12.8 - 13.8)	16.9 (16.1 - 17.7)
	Virus	36.6 (33.8-39.4)	12.4 (11.1-13.9)	26.4 (25.0 - 27.6)	8.7 (8.1 - 9.2)	33.2 (32.2 - 34.0)	11.6 (11.1 - 12.1)
Morocco	*E. coli*	6.5 (5.2-7.9)	12.6 (10.3-15.3)	6.0 (5.1 - 7.1)	11.8 (10.3 - 13.4)	5.3 (4.6 - 6.0)	10.4 (9.2 - 11.7)
	Group B *Streptococcus*	24.2 (21.6-27.1)	27.1 (24.0-30.3)	10.1 (9.3 - 10.9)	14.6 (13.5 - 15.8)	7.7 (7.2 - 8.1)	9.0 (8.4 - 9.6)
	H. influenzae	8.0 (6.3-9.9)	5.1 (4.0-6.4)	7.5 (6.9 - 8.1)	4.7 (4.2 - 5.2)	4.9 (4.6 - 5.2)	2.9 (2.7 - 3.1)
	K. pneumoniae	5.9 (4.6-7.5)	19.3 (15.6-23.6)	3.8 (3.1 - 4.7)	14.1 (12.0 - 16.4)	3.9 (3.4 - 4.5)	13.0 (11.4 - 14.6)
	L. monocytogenes	1.9 (1.3-2.7)	4.8 (3.3-6.8)	2.3 (1.7 - 3.0)	5.6 (4.4 - 7.0)	2.4 (2.0 - 2.7)	6.2 (5.5 - 7.1)
	N. meningitidis	7.3 (6.2-8.5)	6.3 (5.2-7.3)	14.4 (13.4 - 15.4)	10.7 (9.9 - 11.5)	14.8 (14.2 - 15.4)	11.3 (10.7 - 11.9)
	Other	2.2 (1.3-3.4)	2.8 (1.7-4.3)	9.8 (6.9 - 13.7)	9.6 (6.9 - 13.1)	9.9 (8.0 - 12.1)	11.4 (9.3 - 13.9)
	S. aureus	4.3 (3.4-5.5)	5.7 (4.5-7.1)	5.5 (4.8 - 6.1)	6.7 (6.0 - 7.4)	5.6 (5.1 - 6.0)	7.4 (6.8 - 8.0)

Location	Aetiology	Neonatal		Under 5		All Ages	
		Nonfatal	Fatal	Nonfatal	Fatal	Nonfatal	Fatal
	S. pneumoniae	2.8 (2.3-3.4)	3.1 (2.5-3.8)	14.5 (13.5 - 15.6)	12.5 (11.5 - 13.5)	14.9 (14.2 - 15.6)	16.8 (15.9 - 17.7)
	Virus	36.8 (33.7-40.0)	13.2 (11.7-14.8)	26.0 (24.6 - 27.3)	9.8 (9.1 - 10.4)	30.8 (29.8 - 31.7)	11.5 (11.0 - 12.0)
Oman	*E. coli*	6.4 (5.2-7.8)	12.8 (10.4-15.4)	6.4 (5.6 - 7.2)	13.0 (11.6 - 14.5)	5.6 (5.0 - 6.1)	11.8 (10.9 - 12.9)
	Group B *Streptococcus*	24.4 (21.5-27.5)	27.6 (24.0-31.4)	10.9 (10.1 - 11.7)	14.7 (13.5 - 15.9)	8.6 (8.1 - 9.0)	9.5 (8.9 - 10.0)
	H. influenzae	7.8 (6.1-9.9)	4.8 (3.6-6.2)	9.0 (8.2 - 9.8)	5.4 (4.8 - 5.9)	6.2 (5.8 - 6.6)	3.2 (2.9 - 3.4)
	K. pneumoniae	6.2 (4.8-7.9)	21.2 (17.1-26.0)	3.9 (3.2 - 4.7)	15.0 (12.9 - 17.3)	4.0 (3.5 - 4.4)	14.3 (13.0 - 15.6)
	L. monocytogenes	1.9 (1.3-2.7)	5.0 (3.6-7.1)	2.5 (1.9 - 3.2)	6.5 (5.1 - 8.1)	2.5 (2.1 - 2.8)	7.5 (6.8 - 8.1)
	N. meningitidis	6.9 (5.8-8.2)	6.0 (4.9-7.1)	10.5 (9.8 - 11.3)	8.7 (7.9 - 9.5)	11.0 (10.4 - 11.5)	8.9 (8.4 - 9.4)
	Other	2.1 (1.2-3.2)	2.7 (1.6-4.2)	10.4 (7.1 - 14.7)	11.4 (7.9 - 15.8)	10.5 (8.1 - 13.2)	13.2 (10.3 - 16.5)
	S. aureus	4.5 (3.6-5.7)	6.0 (4.8-7.5)	5.6 (5.0 - 6.1)	7.3 (6.6 - 8.1)	5.6 (5.2 - 6.0)	8.1 (7.6 - 8.7)
	S. pneumoniae	2.9 (2.2-3.6)	2.2 (1.7-2.8)	13.9 (12.9 - 14.9)	9.5 (8.7 - 10.3)	13.9 (13.2 - 14.6)	12.3 (11.6 - 13.1)
	Virus	36.9 (33.7-40.1)	11.6 (10.2-13.1)	26.9 (25.4 - 28.3)	8.8 (8.2 - 9.4)	32.3 (31.2 - 33.3)	11.3 (10.7 - 11.8)
Palestine	*E. coli*	6.4 (5.2-7.8)	12.6 (10.3-15.1)	6.2 (5.3 - 7.2)	12.3 (10.7 - 14.0)	5.3 (4.7 - 5.9)	11.1 (10.0 - 12.3)
	Group B *Streptococcus*	23.8 (21.1-26.7)	26.9 (23.8-30.2)	10.3 (9.4 - 11.1)	12.9 (11.9 - 14.0)	7.9 (7.4 - 8.4)	9.4 (8.9 - 10.0)
	H. influenzae	8.1 (6.4-9.9)	5.1 (4.0-6.5)	8.2 (7.5 - 8.9)	5.0 (4.6 - 5.5)	5.5 (5.2 - 5.9)	3.4 (3.1 - 3.6)
	K. pneumoniae	6.0 (4.7-7.5)	20.1 (16.4-24.2)	3.9 (3.1 - 4.7)	13.8 (11.7 - 16.2)	3.9 (3.4 - 4.5)	13.6 (12.1 - 15.1)
	L. monocytogenes	1.9 (1.3-2.7)	4.9 (3.4-6.9)	2.4 (1.8 - 3.1)	6.1 (4.8 - 7.7)	2.3 (2.0 - 2.7)	6.6 (5.8 - 7.4)
	N. meningitidis	7.4 (6.2-8.6)	6.3 (5.3-7.4)	12.6 (11.7 - 13.5)	10.3 (9.4 - 11.2)	13.3 (12.7 - 13.9)	10.7 (10.1 - 11.4)
	Other	2.1 (1.3-3.3)	2.8 (1.7-4.3)	10.2 (7.0 - 14.2)	11.4 (7.9 - 15.5)	10.2 (8.1 - 12.6)	12.4 (9.9 - 15.2)
	S. aureus	4.5 (3.5-5.5)	5.9 (4.7-7.3)	5.6 (5.0 - 6.2)	7.2 (6.4 - 8.0)	5.5 (5.1 - 5.9)	7.7 (7.1 - 8.2)
	S. pneumoniae	2.9 (2.3-3.5)	2.7 (2.2-3.4)	14.2 (13.2 - 15.3)	12.0 (11.0 - 13.1)	14.1 (13.4 - 14.8)	14.0 (13.2 - 14.9)
	Virus	37.0 (33.9-40.2)	12.6 (11.1-14.2)	26.5 (25.1 - 27.8)	9.0 (8.4 - 9.6)	32.0 (31.0 - 33.0)	11.2 (10.7 - 11.6)
Qatar	*E. coli*	6.3 (5.0-7.9)	12.6 (10.1-15.7)	6.5 (5.7 - 7.4)	13.4 (11.8 - 15.1)	4.9 (4.4 - 5.4)	11.5 (10.4 - 12.5)
	Group B *Streptococcus*	24.1 (20.6-27.7)	27.4 (23.4-31.6)	11.0 (10.1 - 11.9)	14.5 (13.3 - 15.9)	7.5 (7.0 - 7.9)	9.8 (9.2 - 10.4)
	H. influenzae	7.8 (5.9-10.2)	4.8 (3.5-6.3)	9.1 (8.2 - 9.9)	5.4 (4.9 - 6.0)	5.0 (4.6 - 5.4)	3.2 (3.0 - 3.5)
	K. pneumoniae	6.4 (4.8-8.3)	22.1 (17.6-27.4)	3.8 (3.1 - 4.7)	15.1 (12.8 - 17.5)	3.9 (3.5 - 4.5)	15.2 (13.8 - 16.9)
	L. monocytogenes	1.9 (1.4-2.8)	5.1 (3.6-7.3)	2.6 (1.9 - 3.3)	6.9 (5.3 - 8.6)	2.3 (2.1 - 2.7)	7.3 (6.5 - 8.1)
	N. meningitidis	6.7 (5.4-8.1)	5.8 (4.7-7.1)	9.5 (8.7 - 10.3)	8.0 (7.3 - 8.8)	10.4 (9.8 - 11.0)	9.3 (8.7 - 10.0)
	Other	2.1 (1.2-3.3)	2.7 (1.6-4.3)	10.6 (7.2 - 15.2)	12.2 (8.4 - 17.1)	10.6 (8.3 - 13.3)	14.3 (11.3 - 17.7)
	S. aureus	4.7 (3.6-6.0)	6.2 (4.9-8.0)	5.7 (5.1 - 6.4)	7.7 (6.9 - 8.6)	5.6 (5.2 - 6.0)	8.4 (7.8 - 9.0)

Location	Aetiology	Neonatal		Under 5		All Ages	
		Nonfatal	Fatal	Nonfatal	Fatal	Nonfatal	Fatal
	S. pneumoniae	2.9 (2.1-3.8)	1.9 (1.4-2.5)	14.2 (13.1 - 15.4)	8.3 (7.5 - 9.2)	13.7 (12.9 - 14.5)	9.7 (9.0 - 10.4)
	Virus	37.1 (33.8-40.5)	11.3 (9.9-12.9)	27.0 (25.3 - 28.4)	8.5 (7.9 - 9.1)	36.0 (34.6 - 37.3)	11.4 (10.8 - 11.9)
Saudi Arabia	*E. coli*	6.5 (5.2-7.9)	12.8 (10.5-15.4)	6.4 (5.6 - 7.4)	12.9 (11.4 - 14.6)	5.2 (4.7 - 5.7)	10.7 (9.7 - 11.8)
	Group B *Streptococcus*	25.0 (22.1-28.1)	28.2 (24.7-31.9)	10.6 (9.8 - 11.5)	13.1 (12.0 - 14.2)	7.6 (7.2 - 8.0)	8.2 (7.7 - 8.7)
	H. influenzae	7.6 (5.8-9.7)	4.7 (3.5-6.1)	8.1 (7.4 - 8.9)	4.9 (4.4 - 5.4)	4.8 (4.5 - 5.1)	2.4 (2.2 - 2.6)
	K. pneumoniae	6.1 (4.7-7.8)	20.9 (16.8-25.7)	3.9 (3.1 - 4.6)	14.1 (11.8 - 16.6)	3.9 (3.5 - 4.4)	14.5 (13.0 - 16.0)
	L. monocytogenes	1.9 (1.3-2.7)	5.0 (3.5-7.0)	2.5 (1.9 - 3.2)	6.6 (5.1 - 8.3)	2.4 (2.1 - 2.7)	7.1 (6.4 - 7.8)
	N. meningitidis	6.7 (5.6-8.0)	5.8 (4.7-6.9)	10.9 (10.1 - 11.7)	9.1 (8.3 - 10.0)	11.6 (11.1 - 12.2)	10.2 (9.6 - 10.8)
	Other	2.1 (1.2-3.3)	2.7 (1.6-4.2)	10.6 (7.2 - 14.9)	12.3 (8.5 - 17.2)	10.5 (8.4 - 12.9)	13.9 (11.2 - 17.2)
	S. aureus	4.5 (3.6-5.6)	5.9 (4.7-7.4)	5.6 (5.0 - 6.2)	7.4 (6.6 - 8.3)	5.6 (5.2 - 5.9)	8.1 (7.6 - 8.7)
	S. pneumoniae	2.8 (2.2-3.5)	2.3 (1.8-2.9)	14.5 (13.5 - 15.7)	11.0 (10.1 - 12.1)	14.3 (13.6 - 15.0)	13.0 (12.3 - 13.8)
	Virus	36.7 (33.6-40.0)	11.6 (10.2-13.1)	26.8 (25.2 - 28.1)	8.5 (7.8 - 9.1)	34.1 (32.9 - 35.1)	11.8 (11.2 - 12.4)
Sudan	*E. coli*	5.9 (4.3-7.8)	11.5 (8.7-14.8)	6.0 (5.0 - 7.1)	11.3 (9.6 - 13.2)	5.5 (4.6 - 6.4)	10.3 (8.8 - 11.9)
	Group B *Streptococcus*	21.5 (17.4-26.2)	24.6 (19.9-29.8)	10.0 (8.9 - 11.1)	11.7 (10.4 - 13.1)	8.3 (7.4 - 9.2)	9.2 (8.2 - 10.2)
	H. influenzae	9.5 (7.3-12.1)	6.3 (4.8-8.3)	10.5 (9.5 - 11.6)	6.4 (5.7 - 7.1)	7.9 (7.2 - 8.7)	4.6 (4.1 - 5.0)
	K. pneumoniae	5.4 (4.0-7.2)	17.8 (13.5-22.6)	4.1 (3.2 - 5.1)	13.3 (10.8 - 16.2)	4.1 (3.4 - 5.0)	13.4 (11.4 - 15.9)
	L. monocytogenes	1.9 (1.3-2.9)	4.9 (3.2-7.2)	2.3 (1.7 - 3.1)	5.6 (4.3 - 7.3)	2.3 (1.9 - 2.9)	5.8 (4.8 - 7.0)
	N. meningitidis	8.7 (6.0-12.0)	7.6 (5.2-10.7)	9.6 (8.2 - 11.5)	7.9 (6.6 - 9.3)	9.9 (8.3 - 11.6)	8.1 (6.8 - 9.6)
	Other	2.5 (1.6-3.7)	3.3 (2.1-4.8)	10.1 (7.0 - 14.1)	11.0 (7.8 - 15.1)	10.2 (8.2 - 12.9)	12.0 (9.7 - 14.7)
	S. aureus	4.4 (3.1-5.9)	5.9 (4.2-7.9)	5.5 (4.8 - 6.5)	6.9 (6.0 - 8.0)	5.6 (5.0 - 6.4)	7.3 (6.4 - 8.3)
	S. pneumoniae	2.9 (2.2-3.8)	3.8 (2.8-4.8)	14.4 (13.2 - 15.7)	16.1 (14.5 - 17.7)	14.6 (13.5 - 15.7)	17.6 (16.1 - 19.1)
	Virus	37.2 (33.6-41.0)	14.3 (12.4-16.4)	27.4 (25.8 - 29.0)	9.9 (9.2 - 10.6)	31.6 (30.2 - 32.9)	11.8 (11.1 - 12.4)
Syrian Arab Republic	*E. coli*	6.9 (5.9-8.2)	13.4 (11.3-15.6)	5.8 (5.0 - 6.6)	10.7 (9.3 - 12.1)	5.1 (4.6 - 5.7)	9.7 (8.8 - 10.6)
	Group B *Streptococcus*	25.9 (24.0-27.8)	28.8 (26.7-30.9)	9.5 (8.9 - 10.0)	10.5 (9.8 - 11.3)	6.8 (6.5 - 7.1)	7.2 (6.8 - 7.6)
	H. influenzae	7.6 (6.3-9.0)	4.7 (3.8-5.7)	8.9 (8.4 - 9.5)	5.0 (4.6 - 5.4)	5.0 (4.7 - 5.3)	2.8 (2.6 - 2.9)
	K. pneumoniae	5.3 (4.2-6.7)	17.7 (14.4-21.5)	3.8 (3.2 - 4.7)	12.4 (10.4 - 14.8)	4.0 (3.6 - 4.5)	12.9 (11.6 - 14.2)
	L. monocytogenes	1.8 (1.3-2.5)	4.7 (3.3-6.3)	2.3 (1.7 - 2.9)	5.4 (4.2 - 6.8)	2.4 (2.1 - 2.6)	6.0 (5.4 - 6.5)
	N. meningitidis	8.1 (7.2-9.1)	6.8 (5.9-7.8)	15.9 (15.1 - 16.7)	12.3 (11.5 - 13.1)	15.4 (14.8 - 15.9)	12.2 (11.7 - 12.8)
	Other	2.0 (1.2-3.1)	2.5 (1.5-3.9)	9.7 (6.9 - 13.5)	10.6 (7.5 - 14.6)	10.2 (8.3 - 12.3)	12.0 (9.9 - 14.5)
	S. aureus	4.0 (3.3-4.9)	5.2 (4.3-6.4)	4.6 (4.2 - 5.0)	5.6 (5.1 - 6.1)	5.0 (4.7 - 5.3)	6.4 (6.0 - 6.8)

Location	Aetiology	Neonatal		Under 5		All Ages	
		Nonfatal	Fatal	Nonfatal	Fatal	Nonfatal	Fatal
	S. pneumoniae	2.7 (2.3-3.2)	4.4 (3.7-5.2)	13.7 (13.0 - 14.5)	19.6 (18.3 - 20.9)	13.4 (12.9 - 13.9)	20.4 (19.5 - 21.2)
	Virus	35.6 (32.9-38.4)	11.8 (10.5-13.1)	25.7 (24.5 - 26.8)	7.8 (7.4 - 8.3)	32.7 (31.8 - 33.5)	10.6 (10.1 - 11.0)
Tunisia	*E. coli*	6.9 (5.9-8.0)	13.5 (11.6-15.5)	6.0 (5.2 - 6.9)	11.6 (10.4 - 12.8)	5.5 (5.0 - 6.0)	10.9 (10.0 - 11.9)
	Group B *Streptococcus*	25.3 (23.8-26.8)	28.3 (26.8-29.9)	9.4 (8.8 - 10.0)	13.4 (12.7 - 14.1)	6.9 (6.6 - 7.2)	7.5 (7.1 - 7.8)
	H. influenzae	7.7 (6.6-9.0)	4.7 (3.9-5.6)	8.9 (8.3 - 9.5)	4.9 (4.5 - 5.3)	5.9 (5.6 - 6.2)	3.2 (3.0 - 3.4)
	K. pneumoniae	5.3 (4.2-6.6)	17.9 (14.7-21.6)	3.9 (3.2 - 4.7)	13.7 (11.8 - 15.8)	4.2 (3.8 - 4.6)	13.3 (12.2 - 14.4)
	L. monocytogenes	1.8 (1.3-2.5)	4.8 (3.5-6.3)	2.4 (1.8 - 3.0)	5.6 (4.5 - 6.8)	2.6 (2.3 - 2.8)	6.9 (6.3 - 7.5)
	N. meningitidis	8.6 (7.8-9.5)	7.3 (6.5-8.2)	14.5 (13.7 - 15.3)	10.7 (10.0 - 11.3)	13.6 (13.2 - 14.1)	9.7 (9.3 - 10.1)
	Other	1.9 (1.1-3.0)	2.5 (1.5-3.9)	10.2 (7.2 - 14.2)	9.7 (6.9 - 13.3)	10.5 (8.5 - 12.9)	12.0 (9.2 - 15.2)
	S. aureus	4.1 (3.3-4.9)	5.3 (4.4-6.5)	4.7 (4.2 - 5.1)	5.6 (5.2 - 6.2)	5.0 (4.8 - 5.3)	6.6 (6.3 - 7.0)
	S. pneumoniae	2.8 (2.4-3.3)	4.4 (3.8-5.1)	13.6 (12.7 - 14.3)	16.5 (15.3 - 17.5)	12.4 (12.0 - 12.8)	19.0 (18.1 - 19.8)
	Virus	35.7 (33.1-38.1)	11.3 (10.1-12.5)	26.5 (25.1 - 27.6)	8.3 (7.8 - 8.8)	33.3 (32.4 - 34.2)	10.8 (10.3 - 11.3)
Turkey	*E. coli*	6.4 (5.1-7.8)	12.7 (10.4-15.3)	6.4 (5.6 - 7.3)	12.9 (11.3 - 14.7)	5.7 (5.2 - 6.2)	11.7 (10.6 - 12.7)
	Group B *Streptococcus*	23.9 (21.0-27.0)	27.2 (23.7-30.8)	10.7 (9.8 - 11.5)	12.2 (11.1 - 13.4)	7.8 (7.4 - 8.1)	8.9 (8.4 - 9.4)
	H. influenzae	8.0 (6.2-10.0)	5.0 (3.8-6.3)	8.8 (8.0 - 9.6)	5.3 (4.8 - 5.9)	5.2 (4.8 - 5.5)	3.2 (3.0 - 3.4)
	K. pneumoniae	6.1 (4.8-7.9)	21.1 (17.0-25.8)	3.9 (3.2 - 4.7)	13.8 (11.5 - 16.4)	4.1 (3.7 - 4.6)	14.1 (12.7 - 15.4)
	L. monocytogenes	1.9 (1.3-2.7)	5.0 (3.6-7.1)	2.5 (1.9 - 3.2)	6.7 (5.1 - 8.5)	2.7 (2.5 - 3.0)	7.4 (6.7 - 8.1)
	N. meningitidis	7.1 (5.9-8.4)	6.1 (5.0-7.3)	10.8 (10.0 - 11.6)	9.3 (8.5 - 10.2)	10.7 (10.2 - 11.3)	9.2 (8.7 - 9.8)
	Other	2.1 (1.3-3.3)	2.7 (1.6-4.3)	10.5 (7.1 - 14.7)	12.8 (8.9 - 17.8)	10.5 (8.2 - 13.0)	13.4 (10.6 - 16.7)
	S. aureus	4.5 (3.6-5.7)	6.1 (4.8-7.5)	5.6 (5.0 - 6.3)	7.6 (6.7 - 8.5)	5.9 (5.6 - 6.3)	8.2 (7.7 - 8.8)
	S. pneumoniae	2.9 (2.2-3.7)	2.3 (1.8-2.9)	14.0 (12.9 - 15.1)	10.9 (9.9 - 11.9)	14.5 (13.8 - 15.2)	12.7 (12.0 - 13.5)
	Virus	37.0 (33.8-40.2)	11.9 (10.4-13.3)	26.9 (25.4 - 28.2)	8.4 (7.8 - 9.0)	32.9 (31.9 - 33.9)	11.3 (10.7 - 11.8)
United Arab Emirates	*E. coli*	6.2 (4.9-7.8)	12.3 (9.8-15.2)	6.1 (5.2 - 7.1)	12.3 (10.7 - 14.1)	4.8 (4.2 - 5.4)	9.6 (8.4 - 10.8)
	Group B *Streptococcus*	23.0 (19.7-26.7)	26.1 (22.2-30.3)	10.4 (9.5 - 11.2)	13.3 (12.0 - 14.5)	7.1 (6.7 - 7.5)	8.1 (7.5 - 8.7)
	H. influenzae	8.3 (6.3-10.5)	5.3 (3.9-6.9)	7.9 (7.3 - 8.7)	5.0 (4.5 - 5.5)	4.4 (4.1 - 4.7)	2.5 (2.3 - 2.7)
	K. pneumoniae	6.3 (4.8-8.1)	21.0 (16.7-26.0)	3.8 (3.0 - 4.6)	13.9 (11.7 - 16.4)	3.9 (3.3 - 4.5)	14.3 (12.3 - 16.2)
	L. monocytogenes	1.9 (1.3-2.8)	5.0 (3.4-7.2)	2.4 (1.8 - 3.1)	6.2 (4.8 - 7.8)	2.3 (2.0 - 2.6)	6.2 (5.5 - 7.1)
	N. meningitidis	7.0 (5.8-8.5)	6.1 (4.9-7.4)	12.3 (11.4 - 13.3)	10.3 (9.4 - 11.3)	13.1 (12.4 - 13.8)	12.6 (11.7 - 13.5)
	Other	2.2 (1.3-3.4)	2.9 (1.8-4.4)	10.1 (7.0 - 14.0)	11.6 (8.1 - 15.8)	10.1 (8.2 - 12.2)	13.8 (11.2 - 16.7)
	S. aureus	4.6 (3.6-5.9)	6.1 (4.8-7.8)	5.8 (5.1 - 6.4)	7.6 (6.7 - 8.5)	5.7 (5.3 - 6.2)	8.3 (7.6 - 9.0)

Location	Aetiology	Neonatal		Under 5		All Ages	
		Nonfatal	Fatal	Nonfatal	Fatal	Nonfatal	Fatal
	S. pneumoniae	2.9 (2.2-3.8)	2.3 (1.7-3.0)	15.2 (14.1 - 16.4)	10.6 (9.7 - 11.7)	15.0 (14.2 - 15.9)	12.1 (11.3 - 13.0)
	Virus	37.5 (34.1-41.0)	13.0 (11.4-14.7)	26.0 (24.6 - 27.4)	9.2 (8.6 - 9.9)	33.6 (32.5 - 34.7)	12.5 (11.9 - 13.2)
Yemen	*E. coli*	6.3 (4.9-7.8)	12.2 (9.8-14.8)	5.5 (4.5 - 6.5)	10.5 (9.0 - 12.3)	4.9 (4.1 - 5.7)	9.4 (8.1 - 10.8)
	Group B *Streptococcus*	21.4 (18.9-24.0)	24.1 (21.3-27.0)	9.1 (8.4 - 9.9)	11.4 (10.5 - 12.2)	7.3 (6.8 - 7.9)	8.3 (7.7 - 9.0)
	H. influenzae	9.1 (7.6-10.7)	6.0 (4.9-7.3)	10.0 (9.4 - 10.7)	6.2 (5.8 - 6.7)	7.0 (6.6 - 7.5)	4.0 (3.8 - 4.3)
	K. pneumoniae	5.7 (4.4-7.3)	18.3 (14.7-22.6)	3.7 (3.0 - 4.7)	12.5 (10.3 - 15.2)	3.8 (3.1 - 4.5)	12.5 (10.5 - 14.5)
	L. monocytogenes	1.9 (1.3-2.7)	4.8 (3.2-6.7)	2.1 (1.6 - 2.7)	5.1 (4.0 - 6.5)	2.1 (1.7 - 2.5)	5.3 (4.5 - 6.3)
	N. meningitidis	8.8 (7.7-10.2)	7.7 (6.5-9.0)	17.1 (16.1 - 18.1)	13.6 (12.6 - 14.6)	17.7 (17.0 - 18.5)	14.5 (13.7 - 15.3)
	Other	2.3 (1.4-3.5)	2.9 (1.8-4.4)	9.3 (6.5 - 12.8)	10.1 (7.2 - 13.7)	9.4 (7.6 - 11.7)	11.2 (9.2 - 13.4)
	S. aureus	4.4 (3.4-5.5)	5.8 (4.5-7.3)	5.1 (4.5 - 5.7)	6.4 (5.7 - 7.2)	5.2 (4.7 - 5.7)	6.9 (6.3 - 7.6)
	S. pneumoniae	3.0 (2.5-3.6)	3.8 (3.1-4.6)	13.1 (12.3 - 13.9)	14.5 (13.5 - 15.6)	13.2 (12.6 - 13.8)	16.3 (15.4 - 17.2)
	Virus	37.3 (34.2-40.3)	14.5 (12.9-16.3)	25.0 (23.8 - 26.1)	9.6 (9.1 - 10.2)	29.5 (28.5 - 30.4)	11.6 (11.0 - 12.1)
**South Asia**	*E. coli*	6.3 (4.9-7.8)	12.1 (9.6-14.8)	5.3 (4.3 - 6.3)	10.0 (8.5 - 11.7)	4.6 (3.9 - 5.4)	8.9 (7.6 - 10.4)
	Group B *Streptococcus*	20.5 (18.4-22.8)	23.1 (20.7-25.6)	8.3 (7.6 - 9.0)	10.4 (9.5 - 11.2)	6.5 (6.0 - 7.0)	7.5 (6.9 - 8.1)
	H. influenzae	9.4 (8.2-10.8)	6.3 (5.3-7.4)	9.6 (9.0 - 10.3)	5.9 (5.4 - 6.3)	7.1 (6.7 - 7.4)	4.2 (3.9 - 4.4)
	K. pneumoniae	5.3 (4.1-6.8)	17.2 (13.7-21.4)	3.7 (2.9 - 4.7)	11.9 (9.7 - 14.6)	3.8 (3.1 - 4.6)	12.0 (10.0 - 14.1)
	L. monocytogenes	1.9 (1.3-2.6)	4.7 (3.2-6.5)	2.0 (1.5 - 2.6)	4.8 (3.7 - 6.1)	2.0 (1.6 - 2.4)	5.1 (4.3 - 6.0)
	N. meningitidis	10.2 (9.1-11.6)	8.6 (7.5-10.0)	19.7 (18.6 - 20.7)	14.9 (13.9 - 15.9)	19.9 (19.2 - 20.7)	15.4 (14.6 - 16.2)
	Other	2.2 (1.4-3.4)	2.9 (1.8-4.4)	9.1 (6.3 - 12.5)	9.6 (6.9 - 13.1)	9.3 (7.7 - 11.3)	10.8 (9.0 - 13.0)
	S. aureus	4.2 (3.3-5.4)	5.6 (4.4-7.2)	4.8 (4.2 - 5.3)	6.1 (5.4 - 6.8)	4.9 (4.4 - 5.3)	6.4 (5.8 - 7.1)
	S. pneumoniae	3.0 (2.6-3.6)	4.7 (3.9-5.6)	13.1 (12.4 - 13.8)	16.9 (15.7 - 18.1)	12.2 (11.8 - 12.7)	18.3 (17.3 - 19.2)
	Virus	36.9 (34.1-39.9)	14.8 (13.2-16.6)	24.5 (23.4 - 25.6)	9.5 (9.0 - 10.1)	29.7 (28.8 - 30.6)	11.5 (11.0 - 12.0)
Bangladesh	*E. coli*	6.5 (5.2-7.9)	12.5 (10.2-15.0)	5.8 (4.8 - 6.9)	10.8 (9.1 - 12.7)	4.7 (4.0 - 5.5)	8.8 (7.5 - 10.1)
	Group B *Streptococcus*	22.5 (20.2-25.0)	25.2 (22.6-27.9)	9.0 (8.2 - 9.8)	10.6 (9.6 - 11.4)	6.7 (6.2 - 7.2)	6.7 (6.2 - 7.2)
	H. influenzae	8.7 (7.3-10.3)	5.7 (4.7-6.8)	8.7 (8.0 - 9.4)	5.3 (4.9 - 5.7)	5.6 (5.3 - 5.9)	2.8 (2.6 - 3.0)
	K. pneumoniae	5.6 (4.3-7.1)	18.1 (14.6-22.1)	3.8 (3.0 - 4.7)	12.3 (10.0 - 15.0)	3.8 (3.2 - 4.5)	12.5 (10.5 - 14.4)
	L. monocytogenes	1.9 (1.3-2.7)	4.7 (3.3-6.6)	2.2 (1.7 - 2.9)	5.3 (4.1 - 6.8)	2.1 (1.8 - 2.5)	5.4 (4.7 - 6.3)
	N. meningitidis	8.6 (7.5-9.8)	7.4 (6.4-8.6)	16.7 (15.6 - 17.8)	13.2 (12.2 - 14.3)	17.8 (17.1 - 18.5)	14.8 (14.0 - 15.6)
	Other	2.2 (1.4-3.4)	2.9 (1.8-4.3)	9.8 (6.8 - 13.5)	10.7 (7.6 - 14.7)	9.7 (8.0 - 11.7)	11.8 (9.9 - 14.0)
	S. aureus	4.3 (3.4-5.4)	5.6 (4.4-7.0)	5.3 (4.6 - 5.9)	6.5 (5.7 - 7.3)	5.2 (4.7 - 5.6)	7.0 (6.3 - 7.6)

Location	Aetiology	Neonatal		Under 5		All Ages	
		Nonfatal	Fatal	Nonfatal	Fatal	Nonfatal	Fatal
	S. pneumoniae	2.9 (2.5-3.5)	4.0 (3.3-4.7)	13.5 (12.5 - 14.5)	16.2 (15.0 - 17.5)	13.3 (12.7 - 13.9)	18.1 (17.2 - 19.0)
	Virus	36.9 (34.1-39.9)	14.0 (12.5-15.7)	25.4 (24.1 - 26.7)	9.2 (8.6 - 9.8)	31.2 (30.2 - 32.1)	12.1 (11.6 - 12.7)
Bhutan	*E. coli*	6.7 (5.4-8.1)	12.9 (10.6-15.4)	5.5 (4.6 - 6.4)	9.9 (8.4 - 11.4)	4.8 (4.1 - 5.5)	9.3 (8.1 - 10.6)
	Group B *Streptococcus*	23.6 (21.5-25.9)	26.3 (23.8-28.7)	8.9 (8.2 - 9.5)	9.6 (8.9 - 10.4)	6.8 (6.4 - 7.2)	7.4 (6.9 - 7.9)
	H. influenzae	8.3 (7.1-9.7)	5.4 (4.5-6.4)	7.4 (6.9 - 8.0)	4.3 (4.0 - 4.6)	5.5 (5.2 - 5.8)	3.3 (3.1 - 3.5)
	K. pneumoniae	5.3 (4.1-6.7)	17.1 (13.7-21.1)	3.8 (3.0 - 4.7)	11.6 (9.5 - 14.1)	3.9 (3.3 - 4.5)	11.9 (10.2 - 13.7)
	L. monocytogenes	1.8 (1.3-2.5)	4.6 (3.2-6.3)	2.1 (1.6 - 2.7)	4.9 (3.7 - 6.3)	2.1 (1.8 - 2.5)	5.4 (4.6 - 6.2)
	N. meningitidis	9.0 (8.0-10.1)	7.6 (6.6-8.8)	18.9 (17.8 - 19.8)	14.4 (13.4 - 15.3)	18.8 (18.1 - 19.4)	13.9 (13.3 - 14.6)
	Other	2.1 (1.3-3.2)	2.7 (1.7-4.1)	9.3 (6.5 - 12.8)	9.9 (7.0 - 13.6)	9.6 (7.8 - 11.6)	10.8 (8.8 - 13.2)
	S. aureus	4.1 (3.2-5.1)	5.3 (4.2-6.7)	4.6 (4.2 - 5.2)	5.5 (5.0 - 6.1)	4.8 (4.4 - 5.2)	6.1 (5.6 - 6.6)
	S. pneumoniae	2.9 (2.4-3.4)	4.8 (4.0-5.7)	14.5 (13.7 - 15.3)	21.6 (20.1 - 23.0)	13.3 (12.7 - 13.8)	21.4 (20.4 - 22.5)
	Virus	36.2 (33.5-39.1)	13.3 (11.9-14.9)	25.0 (23.9 - 26.1)	8.3 (7.9 - 8.8)	30.5 (29.6 - 31.4)	10.5 (10.0 - 10.9)
India	*E. coli*	6.4 (5.0-7.8)	12.2 (9.6-15.0)	5.2 (4.3 - 6.2)	9.7 (8.3 - 11.3)	4.5 (3.8 - 5.3)	8.7 (7.4 - 10.1)
	Group B *Streptococcus*	20.3 (18.4-22.5)	22.8 (20.6-25.2)	8.0 (7.4 - 8.6)	10.1 (9.3 - 10.8)	6.2 (5.7 - 6.6)	6.9 (6.3 - 7.4)
	H. influenzae	9.5 (8.3-10.9)	6.3 (5.4-7.4)	9.9 (9.2 - 10.6)	5.9 (5.5 - 6.4)	7.2 (6.8 - 7.5)	4.1 (3.9 - 4.3)
	K. pneumoniae	5.2 (3.9-6.6)	16.5 (12.9-20.6)	3.7 (2.9 - 4.7)	11.8 (9.7 - 14.4)	3.8 (3.1 - 4.6)	12.0 (10.0 - 13.9)
	L. monocytogenes	1.9 (1.3-2.6)	4.6 (3.2-6.4)	2.0 (1.5 - 2.6)	4.7 (3.6 - 5.9)	2.0 (1.6 - 2.4)	5.1 (4.3 - 5.9)
	N. meningitidis	10.8 (9.7-12.3)	9.3 (8.1-10.7)	20.4 (19.4 - 21.5)	15.3 (14.3 - 16.2)	20.3 (19.6 - 21.1)	15.5 (14.7 - 16.2)
	Other	2.2 (1.3-3.4)	2.8 (1.7-4.3)	9.1 (6.3 - 12.3)	9.2 (6.4 - 12.4)	9.3 (7.7 - 11.3)	10.7 (8.9 - 12.7)
	S. aureus	4.2 (3.2-5.3)	5.5 (4.3-7.0)	4.6 (4.1 - 5.1)	5.5 (4.9 - 6.2)	4.8 (4.3 - 5.2)	6.2 (5.6 - 6.8)
	S. pneumoniae	3.1 (2.6-3.6)	5.4 (4.5-6.4)	12.9 (12.2 - 13.6)	18.7 (17.5 - 19.9)	11.8 (11.4 - 12.3)	19.4 (18.4 - 20.3)
	Virus	36.6 (33.9-39.5)	14.5 (12.9-16.2)	24.3 (23.2 - 25.4)	9.3 (8.8 - 9.8)	30.0 (29.1 - 31.0)	11.6 (11.1 - 12.1)
Nepal	*E. coli*	6.5 (5.1-8.0)	12.4 (10.0-15.1)	5.6 (4.6 - 6.7)	10.4 (8.9 - 12.2)	5.0 (4.2 - 5.8)	9.9 (8.4 - 11.5)
	Group B *Streptococcus*	22.2 (19.9-24.8)	24.8 (22.1-27.6)	9.0 (8.3 - 9.7)	11.1 (10.2 - 11.9)	7.3 (6.7 - 7.8)	7.6 (7.0 - 8.2)
	H. influenzae	8.8 (7.4-10.3)	5.8 (4.8-7.0)	8.8 (8.2 - 9.4)	5.4 (5.0 - 5.8)	6.5 (6.1 - 6.9)	3.5 (3.3 - 3.8)
	K. pneumoniae	5.5 (4.2-7.0)	17.5 (13.9-21.6)	3.8 (3.0 - 4.8)	12.2 (10.0 - 14.8)	3.8 (3.1 - 4.6)	11.6 (9.8 - 13.5)
	L. monocytogenes	1.9 (1.3-2.7)	4.7 (3.2-6.6)	2.1 (1.6 - 2.8)	5.0 (3.9 - 6.4)	2.1 (1.7 - 2.6)	5.8 (4.9 - 6.9)
	N. meningitidis	8.9 (7.8-10.3)	7.7 (6.6-9.0)	18.0 (16.9 - 19.1)	13.8 (12.8 - 14.8)	18.6 (17.8 - 19.4)	12.7 (12.0 - 13.4)
	Other	2.2 (1.4-3.4)	2.9 (1.8-4.3)	9.3 (6.6 - 12.9)	9.8 (7.0 - 13.4)	9.4 (7.6 - 11.8)	10.5 (8.1 - 13.2)
	S. aureus	4.2 (3.3-5.4)	5.5 (4.3-7.1)	5.1 (4.5 - 5.7)	6.2 (5.4 - 6.9)	5.1 (4.6 - 5.6)	6.8 (6.1 - 7.5)

Location	Aetiology	Neonatal		Under 5		All Ages	
		Nonfatal	Fatal	Nonfatal	Fatal	Nonfatal	Fatal
	S. pneumoniae	2.9 (2.5-3.5)	4.4 (3.7-5.2)	13.1 (12.3 - 14.0)	16.7 (15.5 - 17.9)	13.2 (12.5 - 13.8)	20.2 (19.1 - 21.5)
	Virus	36.9 (34.0-39.8)	14.4 (12.8-16.1)	25.2 (24.0 - 26.4)	9.5 (9.0 - 10.1)	29.1 (28.1 - 30.1)	11.5 (10.9 - 12.1)
Pakistan	*E. coli*	6.2 (4.8-7.8)	11.9 (9.4-14.8)	5.4 (4.4 - 6.6)	10.2 (8.6 - 12.2)	4.8 (4.0 - 5.8)	9.3 (7.9 - 11.0)
	Group B *Streptococcus*	20.6 (17.9-23.5)	23.2 (20.2-26.4)	8.9 (8.1 - 9.7)	10.7 (9.7 - 11.6)	7.5 (6.8 - 8.2)	8.8 (8.0 - 9.5)
	H. influenzae	9.4 (7.8-11.1)	6.3 (5.1-7.7)	9.2 (8.5 - 9.9)	5.8 (5.4 - 6.4)	7.2 (6.7 - 7.6)	4.5 (4.2 - 4.8)
	K. pneumoniae	5.7 (4.3-7.4)	18.0 (14.3-22.5)	3.7 (2.9 - 4.8)	12.1 (9.8 - 14.9)	3.7 (3.0 - 4.6)	12.1 (10.0 - 14.5)
	L. monocytogenes	1.9 (1.3-2.8)	4.7 (3.2-6.8)	2.1 (1.5 - 2.7)	5.0 (3.8 - 6.4)	2.0 (1.6 - 2.5)	5.0 (4.1 - 6.1)
	N. meningitidis	9.0 (7.8-10.5)	7.8 (6.6-9.3)	18.2 (17.0 - 19.4)	14.5 (13.4 - 15.7)	18.9 (18.0 - 19.9)	15.5 (14.5 - 16.4)
	Other	2.4 (1.4-3.6)	3.0 (1.9-4.5)	9.2 (6.5 - 12.7)	10.1 (7.2 - 13.8)	9.2 (7.2 - 11.6)	10.7 (8.7 - 13.4)
	S. aureus	4.4 (3.3-5.7)	5.8 (4.4-7.5)	5.2 (4.5 - 5.9)	6.6 (5.7 - 7.4)	5.2 (4.6 - 5.8)	6.8 (6.0 - 7.6)
	S. pneumoniae	3.0 (2.4-3.7)	3.8 (3.1-4.7)	13.5 (12.6 - 14.5)	15.2 (14.0 - 16.4)	13.4 (12.7 - 14.2)	16.1 (15.0 - 17.2)
	Virus	37.5 (34.3-40.6)	15.3 (13.5-17.3)	24.7 (23.5 - 25.9)	9.8 (9.2 - 10.5)	28.1 (27.0 - 29.1)	11.2 (10.7 - 11.8)
**Southeast Asia, East Asia, and Oceania**	*E. coli*	6.5 (5.3-7.8)	12.7 (10.4-15.1)	5.6 (4.8 - 6.4)	10.4 (9.0 - 11.9)	5.0 (4.5 - 5.6)	10.0 (9.0 - 11.0)
	Group B *Streptococcus*	22.3 (20.7-24.0)	24.7 (22.8-26.7)	8.9 (8.3 - 9.4)	10.0 (9.3 - 10.7)	7.0 (6.7 - 7.3)	7.6 (7.2 - 8.0)
	H. influenzae	8.8 (7.7-10.0)	5.8 (5.0-6.7)	10.9 (10.3 - 11.5)	6.4 (5.9 - 6.8)	7.7 (7.4 - 8.1)	4.4 (4.2 - 4.6)
	K. pneumoniae	5.3 (4.2-6.6)	17.2 (13.9-21.0)	3.8 (3.1 - 4.7)	12.2 (10.1 - 14.7)	3.9 (3.4 - 4.5)	12.9 (11.4 - 14.4)
	L. monocytogenes	1.9 (1.3-2.5)	4.7 (3.3-6.4)	2.2 (1.7 - 2.8)	5.2 (4.0 - 6.5)	2.2 (1.9 - 2.6)	6.0 (5.3 - 6.7)
	N. meningitidis	9.7 (8.8-10.8)	8.5 (7.5-9.6)	16.6 (15.8 - 17.4)	13.6 (12.7 - 14.5)	16.4 (15.8 - 16.9)	12.6 (12.1 - 13.2)
	Other	2.1 (1.3-3.2)	2.7 (1.7-4.2)	9.5 (6.7 - 13.1)	10.4 (7.4 - 14.1)	9.9 (8.0 - 12.1)	11.7 (9.5 - 14.3)
	S. aureus	4.2 (3.4-5.1)	5.5 (4.4-6.8)	4.7 (4.2 - 5.1)	5.8 (5.2 - 6.4)	4.8 (4.6 - 5.1)	6.4 (6.0 - 6.8)
	S. pneumoniae	3.0 (2.6-3.4)	4.8 (4.1-5.6)	12.4 (11.8 - 13.0)	17.5 (16.4 - 18.6)	11.8 (11.5 - 12.2)	17.9 (17.1 - 18.6)
	Virus	36.4 (33.8-39.1)	13.4 (12.0-14.9)	25.4 (24.3 - 26.5)	8.6 (8.1 - 9.1)	31.2 (30.3 - 32.0)	10.6 (10.2 - 11.0)
**East Asia**	*E. coli*	6.8 (5.8-7.9)	13.6 (11.6-15.7)	6.1 (5.3 - 6.9)	11.7 (10.3 - 13.0)	5.6 (5.2 - 6.1)	11.2 (10.3 - 12.1)
	Group B *Streptococcus*	24.9 (23.8-26.2)	28.1 (26.7-29.4)	9.5 (8.9 - 10.0)	10.8 (10.2 - 11.5)	7.4 (7.1 - 7.7)	7.6 (7.3 - 8.0)
	H. influenzae	7.8 (6.7-9.1)	4.7 (4.0-5.6)	11.2 (10.4 - 11.8)	6.2 (5.7 - 6.7)	7.7 (7.3 - 8.1)	3.9 (3.7 - 4.1)
	K. pneumoniae	5.3 (4.2-6.6)	18.1 (14.9-21.8)	3.9 (3.3 - 4.7)	13.3 (11.3 - 15.7)	4.2 (3.8 - 4.6)	13.8 (12.6 - 14.9)
	L. monocytogenes	1.8 (1.3-2.5)	4.8 (3.5-6.4)	2.4 (1.9 - 3.1)	6.0 (4.7 - 7.5)	2.6 (2.3 - 2.9)	7.0 (6.5 - 7.6)
	N. meningitidis	8.9 (8.0-9.7)	7.6 (6.8-8.4)	13.2 (12.4 - 13.9)	10.5 (9.8 - 11.2)	12.7 (12.2 - 13.1)	9.4 (9.0 - 9.8)
	Other	1.9 (1.1-3.0)	2.4 (1.5-3.8)	10.3 (7.2 - 14.2)	11.5 (8.0 - 15.9)	10.6 (8.4 - 13.3)	12.6 (9.8 - 15.9)
	S. aureus	4.1 (3.4-4.9)	5.4 (4.4-6.6)	4.6 (4.2 - 5.0)	5.8 (5.2 - 6.3)	4.9 (4.7 - 5.2)	6.6 (6.3 - 7.0)

Location	Aetiology	Neonatal		Under 5		All Ages	
		Nonfatal	Fatal	Nonfatal	Fatal	Nonfatal	Fatal
	S. pneumoniae	2.8 (2.4-3.3)	4.3 (3.7-5.1)	12.2 (11.5 - 12.8)	16.6 (15.4 - 17.7)	11.7 (11.2 - 12.1)	17.5 (16.7 - 18.3)
	Virus	35.7 (33.2-38.1)	10.9 (9.8-12.1)	26.7 (25.3 - 27.9)	7.6 (7.2 - 8.1)	32.6 (31.6 - 33.6)	10.3 (9.8 - 10.8)
China	*E. coli*	6.9 (5.9-7.9)	13.6 (11.7-15.7)	6.1 (5.4 - 6.9)	11.7 (10.4 - 13.0)	5.6 (5.2 - 6.1)	11.2 (10.4 - 12.1)
	Group B *Streptococcus*	25.0 (23.8-26.2)	28.1 (26.7-29.5)	9.5 (8.9 - 10.0)	10.9 (10.2 - 11.6)	7.4 (7.1 - 7.8)	7.6 (7.3 - 8.0)
	H. influenzae	7.8 (6.7-9.0)	4.7 (4.0-5.6)	11.3 (10.6 - 12.0)	6.3 (5.8 - 6.7)	7.9 (7.5 - 8.3)	4.0 (3.8 - 4.2)
	K. pneumoniae	5.3 (4.2-6.5)	18.2 (14.9-21.9)	3.9 (3.3 - 4.8)	13.4 (11.4 - 15.7)	4.2 (3.8 - 4.6)	13.8 (12.6 - 14.9)
	L. monocytogenes	1.8 (1.3-2.5)	4.8 (3.5-6.4)	2.4 (1.9 - 3.1)	6.0 (4.7 - 7.6)	2.6 (2.3 - 2.9)	7.1 (6.5 - 7.7)
	N. meningitidis	8.9 (8.1-9.7)	7.6 (6.8-8.4)	13.0 (12.3 - 13.7)	10.4 (9.7 - 11.1)	12.6 (12.1 - 13.0)	9.3 (8.9 - 9.7)
	Other	1.9 (1.1-3.0)	2.4 (1.4-3.8)	10.3 (7.2 - 14.2)	11.5 (8.0 - 16.0)	10.6 (8.4 - 13.3)	12.6 (9.8 - 16.0)
	S. aureus	4.1 (3.4-4.9)	5.4 (4.4-6.6)	4.6 (4.2 - 5.0)	5.8 (5.2 - 6.3)	4.9 (4.7 - 5.1)	6.6 (6.3 - 7.0)
	S. pneumoniae	2.8 (2.4-3.3)	4.3 (3.7-5.0)	12.1 (11.4 - 12.7)	16.5 (15.3 - 17.6)	11.6 (11.1 - 11.9)	17.5 (16.7 - 18.3)
	Virus	35.6 (33.2-38.1)	10.9 (9.7-12.1)	26.8 (25.4 - 28.0)	7.6 (7.1 - 8.1)	32.7 (31.7 - 33.7)	10.3 (9.8 - 10.7)
Democratic People's Republic of Korea	*E. coli*	6.7 (5.5-8.0)	12.9 (10.8-15.3)	5.6 (4.7 - 6.5)	10.2 (8.8 - 11.7)	5.0 (4.4 - 5.7)	9.2 (8.2 - 10.3)
	Group B *Streptococcus*	23.3 (21.5-25.2)	26.1 (24.0-28.2)	8.8 (8.2 - 9.4)	10.0 (9.3 - 10.7)	6.8 (6.4 - 7.2)	6.8 (6.4 - 7.2)
	H. influenzae	8.4 (7.3-9.7)	5.4 (4.6-6.3)	8.2 (7.6 - 8.7)	4.7 (4.4 - 5.1)	6.1 (5.7 - 6.4)	3.2 (3.0 - 3.4)
	K. pneumoniae	5.3 (4.1-6.6)	17.2 (14.0-21.1)	3.8 (3.1 - 4.7)	12.0 (10.0 - 14.5)	4.0 (3.4 - 4.6)	12.6 (11.0 - 14.1)
	L. monocytogenes	1.8 (1.3-2.5)	4.7 (3.3-6.3)	2.2 (1.6 - 2.8)	5.1 (3.9 - 6.4)	2.2 (1.9 - 2.6)	5.6 (5.0 - 6.4)
	N. meningitidis	9.2 (8.4-10.3)	7.9 (6.9-8.9)	17.9 (16.9 - 18.8)	13.6 (12.7 - 14.5)	17.4 (16.9 - 18.0)	13.3 (12.8 - 13.9)
	Other	2.1 (1.3-3.2)	2.7 (1.6-4.0)	9.5 (6.7 - 13.1)	10.1 (7.2 - 13.8)	9.8 (8.0 - 11.9)	11.6 (9.6 - 13.9)
	S. aureus	4.1 (3.3-5.1)	5.4 (4.3-6.6)	4.7 (4.2 - 5.1)	5.6 (5.0 - 6.2)	4.9 (4.6 - 5.2)	6.4 (5.9 - 6.9)
	S. pneumoniae	2.9 (2.5-3.4)	4.8 (4.1-5.7)	14.0 (13.2 - 14.8)	20.3 (18.9 - 21.7)	12.9 (12.4 - 13.3)	20.1 (19.2 - 21.0)
	Virus	36.2 (33.6-39.0)	12.9 (11.6-14.4)	25.3 (24.2 - 26.5)	8.3 (7.9 - 8.8)	30.9 (30.1 - 31.8)	11.1 (10.6 - 11.5)
Taiwan (Province of China)	*E. coli*	6.5 (5.2-8.0)	13.0 (10.6-15.8)	6.7 (5.9 - 7.6)	13.7 (12.1 - 15.4)	6.0 (5.5 - 6.4)	12.7 (11.5 - 13.8)
	Group B *Streptococcus*	25.8 (22.7-29.0)	29.2 (25.6-33.0)	11.2 (10.2 - 12.1)	13.7 (12.5 - 15.0)	8.3 (7.8 - 8.7)	8.7 (8.2 - 9.3)
	H. influenzae	7.3 (5.6-9.4)	4.4 (3.2-5.8)	8.5 (7.7 - 9.3)	5.0 (4.5 - 5.6)	5.0 (4.7 - 5.4)	2.7 (2.4 - 2.9)
	K. pneumoniae	6.2 (4.8-7.9)	21.6 (17.3-26.5)	3.9 (3.2 - 4.7)	14.7 (12.3 - 17.1)	4.1 (3.7 - 4.5)	14.4 (13.1 - 15.9)
	L. monocytogenes	1.9 (1.3-2.7)	5.0 (3.5-6.9)	2.6 (2.0 - 3.4)	7.1 (5.5 - 9.0)	2.8 (2.6 - 3.1)	8.5 (7.8 - 9.3)
	N. meningitidis	6.5 (5.3-7.7)	5.5 (4.5-6.7)	9.2 (8.5 - 10.0)	7.8 (7.1 - 8.6)	9.2 (8.7 - 9.7)	7.0 (6.5 - 7.5)
	Other	2.0 (1.1-3.2)	2.6 (1.5-4.1)	10.8 (7.3 - 15.6)	12.9 (8.7 - 18.3)	10.8 (8.3 - 13.9)	13.7 (10.1 - 18.2)
	S. aureus	4.5 (3.5-5.6)	6.0 (4.7-7.5)	5.6 (5.0 - 6.2)	7.5 (6.7 - 8.4)	5.9 (5.5 - 6.2)	8.4 (7.8 - 9.0)

Location	Aetiology	Neonatal		Under 5		All Ages	
		Nonfatal	Fatal	Nonfatal	Fatal	Nonfatal	Fatal
	S. pneumoniae	2.8 (2.1-3.6)	2.0 (1.5-2.6)	13.9 (12.9 - 15.1)	9.7 (8.8 - 10.7)	14.5 (13.8 - 15.3)	12.9 (12.0 - 13.7)
	Virus	36.5 (33.2-39.7)	10.6 (9.3-12.1)	27.4 (25.7 - 28.9)	8.0 (7.4 - 8.6)	33.4 (32.2 - 34.6)	11.0 (10.3 - 11.6)
**Oceania**	*E. coli*	6.2 (4.8-7.9)	11.9 (9.2-14.8)	5.0 (4.1 - 6.1)	9.3 (7.6 - 11.1)	4.6 (3.7 - 5.5)	8.6 (7.2 - 10.3)
	Group B *Streptococcus*	20.1 (17.7-22.7)	22.5 (19.8-25.3)	8.4 (7.7 - 9.1)	9.3 (8.4 - 10.2)	6.9 (6.3 - 7.5)	8.1 (7.3 - 8.8)
	H. influenzae	9.6 (8.3-11.1)	6.5 (5.5-7.7)	11.8 (11.0 - 12.5)	7.4 (6.9 - 8.0)	8.5 (8.0 - 9.0)	5.8 (5.4 - 6.3)
	K. pneumoniae	5.3 (4.0-6.9)	16.8 (13.1-21.1)	3.6 (2.8 - 4.6)	11.2 (9.0 - 14.1)	3.7 (3.0 - 4.5)	11.4 (9.4 - 13.9)
	L. monocytogenes	1.9 (1.3-2.7)	4.6 (3.1-6.5)	1.9 (1.4 - 2.5)	4.5 (3.4 - 5.9)	1.9 (1.5 - 2.4)	4.6 (3.6 - 5.7)
	N. meningitidis	10.2 (8.9-11.8)	8.8 (7.5-10.3)	20.2 (19.1 - 21.3)	16.4 (15.3 - 17.6)	20.7 (19.8 - 21.6)	17.2 (16.2 - 18.2)
	Other	2.3 (1.4-3.5)	3.0 (1.8-4.5)	8.6 (6.1 - 12.0)	9.6 (6.8 - 13.2)	8.9 (7.1 - 11.1)	10.2 (8.1 - 12.8)
	S. aureus	4.2 (3.2-5.5)	5.6 (4.2-7.3)	4.7 (4.1 - 5.2)	5.8 (5.1 - 6.5)	4.8 (4.3 - 5.3)	6.0 (5.4 - 6.7)
	S. pneumoniae	3.1 (2.5-3.7)	4.9 (4.0-5.9)	11.9 (11.2 - 12.6)	17.2 (16.0 - 18.4)	12.1 (11.5 - 12.7)	17.5 (16.5 - 18.6)
	Virus	37.1 (34.2-40.3)	15.5 (13.7-17.4)	23.9 (22.8 - 25.1)	9.3 (8.8 - 9.9)	28.0 (27.1 - 29.0)	10.5 (10.0 - 11.1)
American Samoa	*E. coli*	6.2 (4.8-7.7)	12.3 (9.7-15.0)	4.9 (4.1 - 5.8)	9.7 (8.4 - 11.3)	4.7 (4.1 - 5.4)	9.0 (7.9 - 10.2)
	Group B *Streptococcus*	19.2 (17.9-20.6)	21.9 (20.4-23.5)	7.6 (7.1 - 8.1)	9.6 (9.0 - 10.3)	5.9 (5.5 - 6.2)	6.5 (6.1 - 7.0)
	H. influenzae	9.8 (8.5-11.3)	6.5 (5.5-7.6)	21.0 (19.9 - 22.0)	12.5 (11.7 - 13.3)	11.4 (10.9 - 11.9)	6.1 (5.7 - 6.4)
	K. pneumoniae	5.0 (3.8-6.5)	16.7 (13.3-20.9)	3.5 (2.7 - 4.3)	12.0 (9.9 - 14.5)	3.9 (3.3 - 4.6)	12.9 (11.1 - 14.7)
	L. monocytogenes	1.9 (1.3-2.6)	4.8 (3.3-6.6)	1.9 (1.4 - 2.5)	4.9 (3.8 - 6.1)	2.2 (1.9 - 2.5)	5.5 (4.8 - 6.3)
	N. meningitidis	12.0 (10.9-13.4)	10.5 (9.3-11.9)	16.4 (15.6 - 17.2)	13.6 (12.8 - 14.5)	16.6 (16.0 - 17.1)	13.8 (13.2 - 14.5)
	Other	2.1 (1.2-3.2)	2.7 (1.6-4.2)	8.6 (5.9 - 12.0)	9.7 (6.8 - 13.3)	9.6 (7.8 - 11.5)	11.8 (9.8 - 14.0)
	S. aureus	4.2 (3.3-5.3)	5.6 (4.4-7.0)	4.1 (3.6 - 4.5)	5.3 (4.8 - 5.9)	4.7 (4.4 - 5.1)	6.4 (5.9 - 6.9)
	S. pneumoniae	3.2 (2.7-3.7)	5.4 (4.6-6.4)	8.9 (8.4 - 9.4)	14.0 (13.0 - 14.9)	9.7 (9.3 - 10.0)	16.3 (15.5 - 17.0)
	Virus	36.4 (33.8-39.0)	13.5 (12.1-15.0)	23.2 (22.2 - 24.2)	8.6 (8.1 - 9.1)	31.4 (30.4 - 32.3)	11.7 (11.2 - 12.2)
Cook Islands	*E. coli*	6.9 (5.9-8.0)	13.5 (11.5-15.5)	5.9 (5.1 - 6.7)	11.2 (9.9 - 12.5)	5.6 (5.1 - 6.1)	10.4 (9.5 - 11.4)
	Group B *Streptococcus*	25.4 (23.8-27.0)	28.4 (26.6-30.1)	9.1 (8.5 - 9.7)	11.7 (11.0 - 12.4)	6.7 (6.4 - 7.0)	6.7 (6.3 - 7.0)
	H. influenzae	7.7 (6.6-9.0)	4.7 (3.9-5.6)	9.1 (8.5 - 9.8)	5.1 (4.7 - 5.4)	5.5 (5.3 - 5.8)	2.9 (2.7 - 3.0)
	K. pneumoniae	5.3 (4.2-6.6)	17.8 (14.6-21.6)	3.8 (3.1 - 4.7)	13.0 (11.1 - 15.3)	4.2 (3.8 - 4.6)	13.2 (11.9 - 14.3)
	L. monocytogenes	1.8 (1.3-2.5)	4.7 (3.4-6.3)	2.3 (1.8 - 3.0)	5.6 (4.4 - 6.9)	2.7 (2.4 - 2.9)	6.8 (6.2 - 7.4)
	N. meningitidis	8.5 (7.6-9.4)	7.2 (6.3-8.1)	15.2 (14.3 - 16.0)	11.4 (10.7 - 12.1)	13.9 (13.4 - 14.3)	10.2 (9.8 - 10.7)
	Other	1.9 (1.2-3.1)	2.5 (1.5-3.9)	10.1 (7.1 - 14.1)	10.4 (7.3 - 14.2)	10.4 (8.4 - 12.9)	12.2 (9.5 - 15.4)
	S. aureus	4.1 (3.3-4.9)	5.3 (4.3-6.4)	4.6 (4.2 - 5.1)	5.6 (5.1 - 6.2)	5.2 (4.9 - 5.4)	6.7 (6.3 - 7.1)

Location	Aetiology	Neonatal		Under 5		All Ages	
		Nonfatal	Fatal	Nonfatal	Fatal	Nonfatal	Fatal
	S. pneumoniae	2.8 (2.4-3.3)	4.4 (3.8-5.2)	13.7 (12.9 - 14.5)	18.0 (16.7 - 19.2)	12.8 (12.4 - 13.3)	19.8 (18.9 - 20.7)
	Virus	35.7 (33.0-38.3)	11.5 (10.3-12.8)	26.0 (24.7 - 27.1)	8.0 (7.6 - 8.5)	33.0 (32.0 - 33.9)	11.1 (10.6 - 11.6)
Fiji	*E. coli*	6.4 (5.0-7.9)	12.3 (9.9-15.0)	5.7 (4.7 - 6.8)	10.8 (9.1 - 12.7)	4.9 (4.1 - 5.7)	9.2 (7.8 - 10.5)
	Group B *Streptococcus*	22.8 (20.0-25.8)	25.6 (22.3-28.8)	9.5 (8.6 - 10.3)	11.2 (10.2 - 12.1)	7.0 (6.5 - 7.6)	7.9 (7.2 - 8.5)
	H. influenzae	8.5 (6.8-10.4)	5.6 (4.4-7.0)	8.0 (7.4 - 8.6)	5.0 (4.6 - 5.4)	4.8 (4.5 - 5.1)	2.9 (2.7 - 3.1)
	K. pneumoniae	5.8 (4.5-7.5)	18.8 (15.0-23.3)	3.7 (3.0 - 4.7)	12.4 (10.1 - 15.0)	3.8 (3.2 - 4.5)	12.5 (10.5 - 14.5)
	L. monocytogenes	1.9 (1.3-2.8)	4.7 (3.2-6.8)	2.2 (1.6 - 2.8)	5.3 (4.1 - 6.9)	2.2 (1.8 - 2.6)	5.4 (4.6 - 6.4)
	N. meningitidis	7.8 (6.7-9.2)	6.7 (5.7-8.0)	16.4 (15.3 - 17.5)	13.2 (12.2 - 14.4)	16.9 (16.2 - 17.6)	14.6 (13.8 - 15.4)
	Other	2.3 (1.4-3.5)	2.9 (1.8-4.5)	9.5 (6.7 - 13.3)	10.7 (7.6 - 14.7)	9.6 (7.8 - 11.6)	11.7 (9.7 - 13.9)
	S. aureus	4.4 (3.3-5.6)	5.7 (4.4-7.3)	5.4 (4.7 - 6.0)	6.8 (5.9 - 7.6)	5.5 (5.0 - 6.0)	7.2 (6.5 - 7.9)
	S. pneumoniae	2.9 (2.3-3.5)	3.4 (2.7-4.1)	14.5 (13.5 - 15.5)	15.3 (14.2 - 16.5)	14.9 (14.2 - 15.6)	17.0 (16.0 - 17.9)
	Virus	37.2 (34.0-40.4)	14.2 (12.6-16.0)	25.2 (23.9 - 26.4)	9.3 (8.7 - 9.9)	30.4 (29.5 - 31.4)	11.7 (11.2 - 12.2)
Guam	*E. coli*	6.3 (5.0-7.8)	12.6 (10.2-15.3)	5.0 (4.3 - 5.9)	10.3 (8.9 - 11.7)	5.0 (4.5 - 5.7)	9.8 (8.8 - 10.9)
	Group B *Streptococcus*	19.9 (18.8-21.0)	22.8 (21.6-24.1)	7.8 (7.3 - 8.4)	10.3 (9.6 - 10.9)	6.2 (5.9 - 6.5)	7.2 (6.8 - 7.6)
	H. influenzae	9.5 (8.2-10.9)	6.1 (5.1-7.2)	21.9 (20.8 - 22.9)	12.8 (11.9 - 13.6)	12.2 (11.7 - 12.6)	7.0 (6.6 - 7.3)
	K. pneumoniae	5.0 (3.9-6.5)	17.2 (13.8-21.2)	3.5 (2.8 - 4.3)	12.6 (10.5 - 15.0)	4.0 (3.5 - 4.6)	13.3 (11.8 - 14.9)
	L. monocytogenes	1.9 (1.3-2.6)	4.9 (3.4-6.6)	2.0 (1.5 - 2.5)	5.1 (4.0 - 6.4)	2.3 (2.1 - 2.7)	5.9 (5.2 - 6.7)
	N. meningitidis	11.8 (10.7-13.0)	10.3 (9.2-11.5)	14.6 (13.8 - 15.3)	12.3 (11.5 - 13.0)	14.5 (14.0 - 14.9)	12.1 (11.6 - 12.6)
	Other	2.0 (1.2-3.1)	2.6 (1.6-4.1)	8.8 (6.1 - 12.4)	9.9 (6.9 - 13.7)	9.8 (8.0 - 12.1)	11.9 (9.7 - 14.4)
	S. aureus	4.2 (3.3-5.2)	5.6 (4.4-7.0)	4.0 (3.6 - 4.5)	5.4 (4.9 - 6.0)	4.8 (4.5 - 5.1)	6.3 (5.9 - 6.7)
	S. pneumoniae	3.1 (2.7-3.6)	5.2 (4.4-6.1)	8.6 (8.1 - 9.1)	13.1 (12.2 - 13.9)	9.5 (9.2 - 9.8)	15.4 (14.6 - 16.1)
	Virus	36.3 (33.8-38.8)	12.6 (11.4-14.0)	23.7 (22.7 - 24.7)	8.4 (7.9 - 8.9)	31.6 (30.7 - 32.5)	11.1 (10.6 - 11.6)
Kiribati	*E. coli*	6.2 (4.7-7.9)	11.8 (9.1-14.8)	5.2 (4.1 - 6.3)	9.6 (7.9 - 11.6)	4.3 (3.5 - 5.2)	7.8 (6.3 - 9.4)
	Group B *Streptococcus*	20.1 (17.4-23.1)	22.6 (19.6-25.8)	8.5 (7.7 - 9.3)	9.9 (8.9 - 10.8)	6.2 (5.6 - 6.8)	6.6 (6.0 - 7.3)
	H. influenzae	9.6 (8.0-11.4)	6.6 (5.3-7.9)	9.5 (8.8 - 10.2)	6.0 (5.6 - 6.6)	5.7 (5.3 - 6.1)	3.3 (3.0 - 3.6)
	K. pneumoniae	5.5 (4.2-7.3)	17.3 (13.4-21.9)	3.6 (2.8 - 4.7)	11.4 (9.1 - 14.4)	3.7 (2.9 - 4.5)	11.6 (9.2 - 14.0)
	L. monocytogenes	1.9 (1.3-2.7)	4.6 (3.1-6.7)	2.0 (1.4 - 2.6)	4.6 (3.5 - 6.0)	1.9 (1.5 - 2.3)	4.6 (3.7 - 5.6)
	N. meningitidis	9.5 (8.1-11.2)	8.2 (6.9-9.8)	20.3 (19.0 - 21.6)	16.1 (14.9 - 17.4)	21.5 (20.5 - 22.5)	18.5 (17.3 - 19.5)
	Other	2.4 (1.5-3.6)	3.1 (1.9-4.6)	8.8 (6.2 - 12.2)	9.7 (6.8 - 13.3)	9.0 (7.4 - 10.8)	10.9 (9.0 - 12.9)
	S. aureus	4.3 (3.2-5.7)	5.7 (4.2-7.6)	5.0 (4.4 - 5.8)	6.2 (5.4 - 7.1)	5.1 (4.5 - 5.7)	6.6 (5.8 - 7.5)

Location	Aetiology	Neonatal		Under 5		All Ages	
		Nonfatal	Fatal	Nonfatal	Fatal	Nonfatal	Fatal
	S. pneumoniae	3.0 (2.4-3.7)	4.3 (3.5-5.3)	13.1 (12.3 - 14.1)	16.6 (15.4 - 18.0)	13.1 (12.5 - 13.8)	17.8 (16.8 - 18.9)
	Virus	37.4 (34.3-40.8)	15.9 (14.1-18.0)	24.0 (22.9 - 25.3)	9.8 (9.2 - 10.5)	29.7 (28.6 - 30.7)	12.4 (11.7 - 13.0)
Marshall Islands	*E. coli*	6.3 (4.9-7.9)	12.1 (9.6-14.8)	5.3 (4.3 - 6.4)	9.9 (8.3 - 11.8)	4.5 (3.7 - 5.3)	8.1 (6.8 - 9.6)
	Group B *Streptococcus*	20.9 (18.5-23.6)	23.6 (20.8-26.5)	8.6 (7.8 - 9.3)	10.4 (9.5 - 11.2)	6.5 (5.9 - 7.0)	6.8 (6.2 - 7.3)
	H. influenzae	9.2 (7.8-10.9)	6.2 (5.1-7.5)	10.4 (9.8 - 11.2)	6.5 (6.0 - 7.0)	6.2 (5.8 - 6.5)	3.1 (2.9 - 3.4)
	K. pneumoniae	5.5 (4.2-7.1)	17.6 (13.9-21.9)	3.6 (2.8 - 4.7)	11.8 (9.6 - 14.6)	3.7 (3.0 - 4.5)	12.1 (9.9 - 14.4)
	L. monocytogenes	1.9 (1.3-2.7)	4.7 (3.1-6.7)	2.0 (1.5 - 2.6)	4.9 (3.7 - 6.2)	2.0 (1.6 - 2.4)	4.8 (4.0 - 5.8)
	N. meningitidis	9.2 (8.1-10.7)	8.0 (6.8-9.4)	19.0 (17.8 - 20.1)	15.0 (13.9 - 16.1)	19.6 (18.9 - 20.5)	17.0 (16.1 - 18.0)
	Other	2.3 (1.4-3.5)	3.0 (1.8-4.5)	9.1 (6.4 - 12.6)	9.9 (7.0 - 13.5)	9.3 (7.6 - 11.2)	11.3 (9.5 - 13.4)
	S. aureus	4.3 (3.3-5.6)	5.7 (4.3-7.3)	5.0 (4.4 - 5.6)	6.2 (5.4 - 6.9)	5.1 (4.6 - 5.6)	6.7 (6.0 - 7.5)
	S. pneumoniae	3.0 (2.5-3.6)	4.2 (3.5-5.1)	12.9 (12.1 - 13.7)	16.0 (14.8 - 17.2)	13.2 (12.6 - 13.9)	17.8 (16.8 - 18.8)
	Virus	37.2 (34.2-40.3)	15.1 (13.4-16.9)	24.2 (23.1 - 25.4)	9.5 (9.0 - 10.2)	29.9 (29.0 - 30.9)	12.2 (11.6 - 12.8)
Micronesia (Federated States of)	*E. coli*	6.2 (4.9-7.7)	12.1 (9.7-14.8)	5.3 (4.4 - 6.4)	10.4 (8.8 - 12.2)	4.6 (3.9 - 5.4)	8.6 (7.3 - 9.9)
	Group B *Streptococcus*	20.8 (18.4-23.4)	23.6 (20.9-26.3)	8.8 (8.1 - 9.5)	10.8 (9.9 - 11.6)	6.5 (6.0 - 7.0)	6.8 (6.3 - 7.4)
	H. influenzae	9.3 (7.8-10.8)	6.2 (5.1-7.4)	11.8 (11.1 - 12.6)	7.3 (6.8 - 7.9)	6.5 (6.1 - 6.8)	3.1 (2.9 - 3.3)
	K. pneumoniae	5.6 (4.4-7.2)	18.3 (14.7-22.4)	3.6 (2.9 - 4.6)	12.3 (10.0 - 15.0)	3.8 (3.1 - 4.5)	12.7 (10.6 - 14.8)
	L. monocytogenes	1.9 (1.3-2.7)	4.8 (3.3-6.8)	2.1 (1.6 - 2.7)	5.1 (4.0 - 6.5)	2.1 (1.8 - 2.5)	5.3 (4.5 - 6.1)
	N. meningitidis	9.2 (8.0-10.5)	8.0 (6.8-9.3)	17.1 (16.1 - 18.1)	13.9 (12.9 - 14.9)	17.7 (17.0 - 18.4)	15.5 (14.6 - 16.4)
	Other	2.3 (1.4-3.5)	2.9 (1.8-4.4)	9.2 (6.5 - 12.8)	10.3 (7.4 - 14.1)	9.5 (7.9 - 11.4)	12.0 (10.1 - 14.2)
	S. aureus	4.4 (3.4-5.6)	5.8 (4.5-7.3)	5.0 (4.4 - 5.6)	6.4 (5.7 - 7.1)	5.2 (4.8 - 5.7)	7.1 (6.4 - 7.8)
	S. pneumoniae	3.0 (2.5-3.6)	3.8 (3.1-4.6)	12.5 (11.8 - 13.3)	14.2 (13.2 - 15.2)	13.1 (12.5 - 13.7)	16.4 (15.5 - 17.4)
	Virus	37.3 (34.4-40.3)	14.6 (13.0-16.3)	24.5 (23.3 - 25.6)	9.4 (8.9 - 10.0)	30.9 (30.0 - 31.9)	12.5 (11.8 - 13.0)
Nauru	*E. coli*	6.8 (5.6-8.2)	13.1 (10.8-15.6)	5.6 (4.7 - 6.5)	9.9 (8.4 - 11.5)	4.8 (4.1 - 5.5)	8.8 (7.6 - 9.9)
	Group B *Streptococcus*	25.0 (22.7-27.4)	27.7 (25.1-30.3)	9.0 (8.3 - 9.7)	9.7 (8.9 - 10.4)	7.0 (6.6 - 7.4)	7.5 (7.0 - 8.0)
	H. influenzae	7.9 (6.6-9.4)	5.0 (4.1-6.1)	6.8 (6.2 - 7.3)	3.9 (3.6 - 4.2)	4.9 (4.6 - 5.2)	2.8 (2.6 - 3.0)
	K. pneumoniae	5.3 (4.1-6.8)	17.2 (13.7-21.2)	3.7 (3.0 - 4.7)	11.5 (9.4 - 14.0)	3.8 (3.2 - 4.5)	12.1 (10.4 - 14.0)
	L. monocytogenes	1.8 (1.3-2.5)	4.6 (3.2-6.3)	2.1 (1.6 - 2.8)	5.0 (3.8 - 6.3)	2.1 (1.8 - 2.4)	5.0 (4.3 - 5.8)
	N. meningitidis	8.2 (7.3-9.4)	7.0 (6.0-8.1)	18.6 (17.5 - 19.5)	14.1 (13.1 - 15.0)	18.7 (18.0 - 19.4)	15.2 (14.5 - 15.9)
	Other	2.1 (1.3-3.2)	2.6 (1.6-4.1)	9.4 (6.6 - 13.0)	10.0 (7.1 - 13.8)	9.6 (7.8 - 11.7)	11.1 (9.2 - 13.5)
	S. aureus	4.0 (3.2-5.0)	5.2 (4.2-6.5)	4.7 (4.2 - 5.2)	5.5 (4.9 - 6.1)	4.7 (4.4 - 5.1)	5.9 (5.5 - 6.4)

Location	Aetiology	Neonatal		Under 5		All Ages	
		Nonfatal	Fatal	Nonfatal	Fatal	Nonfatal	Fatal
	S. pneumoniae	2.8 (2.3-3.3)	4.6 (3.9-5.5)	15.2 (14.3 - 16.1)	22.4 (20.8 - 23.9)	13.9 (13.3 - 14.5)	21.2 (20.3 - 22.3)
	Virus	36.0 (33.2-38.9)	12.9 (11.5-14.4)	24.9 (23.8 - 26.1)	8.1 (7.6 - 8.5)	30.5 (29.5 - 31.3)	10.2 (9.8 - 10.6)
Niue	*E. coli*	6.4 (5.2-7.8)	12.7 (10.4-15.1)	6.1 (5.3 - 7.0)	12.1 (10.6 - 13.8)	5.6 (5.0 - 6.2)	10.7 (9.7 - 11.8)
	Group B *Streptococcus*	23.7 (21.1-26.6)	26.9 (23.9-30.1)	10.2 (9.4 - 10.9)	12.1 (11.2 - 13.1)	7.4 (7.0 - 7.8)	8.6 (8.1 - 9.1)
	H. influenzae	8.1 (6.4-9.9)	5.1 (4.0-6.4)	9.1 (8.4 - 9.8)	5.5 (5.0 - 6.0)	4.9 (4.7 - 5.2)	3.1 (2.9 - 3.3)
	K. pneumoniae	6.0 (4.7-7.5)	20.1 (16.4-24.1)	3.8 (3.1 - 4.7)	13.5 (11.3 - 16.0)	4.1 (3.6 - 4.5)	13.6 (12.1 - 15.1)
	L. monocytogenes	1.9 (1.3-2.7)	4.9 (3.5-6.9)	2.4 (1.8 - 3.0)	6.1 (4.8 - 7.7)	2.6 (2.4 - 2.9)	6.6 (5.9 - 7.3)
	N. meningitidis	7.4 (6.3-8.6)	6.4 (5.4-7.5)	12.7 (11.9 - 13.5)	10.6 (9.8 - 11.5)	12.4 (11.9 - 13.0)	10.9 (10.3 - 11.5)
	Other	2.1 (1.3-3.3)	2.8 (1.7-4.3)	10.1 (7.0 - 14.0)	11.8 (8.3 - 16.2)	10.2 (8.2 - 12.5)	12.8 (10.4 - 15.6)
	S. aureus	4.4 (3.5-5.5)	5.9 (4.8-7.3)	5.4 (4.9 - 6.0)	7.1 (6.3 - 7.9)	5.8 (5.4 - 6.2)	7.8 (7.3 - 8.3)
	S. pneumoniae	2.9 (2.3-3.5)	2.8 (2.2-3.4)	13.9 (13.0 - 14.9)	12.4 (11.5 - 13.5)	14.8 (14.1 - 15.4)	14.5 (13.8 - 15.3)
	Virus	37.0 (33.9-40.0)	12.5 (11.1-14.1)	26.3 (24.9 - 27.5)	8.7 (8.1 - 9.3)	32.2 (31.2 - 33.1)	11.4 (10.9 - 11.8)
Northern Mariana Islands	*E. coli*	6.4 (5.2-7.8)	12.8 (10.5-15.3)	5.1 (4.4 - 6.0)	10.6 (9.2 - 12.0)	5.1 (4.6 - 5.7)	10.0 (9.1 - 11.1)
	Group B *Streptococcus*	20.4 (19.4-21.4)	23.4 (22.2-24.5)	8.0 (7.5 - 8.5)	10.5 (9.9 - 11.2)	6.1 (5.8 - 6.4)	6.8 (6.5 - 7.2)
	H. influenzae	9.3 (8.0-10.8)	5.9 (4.9-7.0)	22.6 (21.4 - 23.6)	13.0 (12.2 - 13.8)	11.0 (10.6 - 11.5)	5.9 (5.6 - 6.2)
	K. pneumoniae	5.1 (3.9-6.6)	17.4 (14.0-21.6)	3.5 (2.8 - 4.3)	12.9 (10.8 - 15.3)	4.2 (3.7 - 4.7)	14.0 (12.5 - 15.5)
	L. monocytogenes	1.9 (1.3-2.5)	4.9 (3.5-6.6)	2.0 (1.5 - 2.6)	5.3 (4.2 - 6.6)	2.5 (2.2 - 2.7)	6.4 (5.7 - 7.1)
	N. meningitidis	11.6 (10.6-12.7)	10.2 (9.1-11.3)	13.4 (12.7 - 14.1)	11.5 (10.7 - 12.1)	13.2 (12.8 - 13.6)	10.7 (10.2 - 11.2)
	Other	1.9 (1.2-3.0)	2.6 (1.5-4.0)	9.0 (6.2 - 12.5)	10.2 (7.2 - 14.1)	10.2 (8.2 - 12.6)	12.7 (10.1 - 15.5)
	S. aureus	4.2 (3.3-5.2)	5.6 (4.4-6.9)	4.0 (3.6 - 4.4)	5.4 (4.9 - 6.0)	4.9 (4.7 - 5.2)	6.7 (6.3 - 7.0)
	S. pneumoniae	3.1 (2.6-3.6)	5.1 (4.3-5.9)	8.4 (7.9 - 8.9)	12.6 (11.7 - 13.4)	9.6 (9.2 - 9.9)	15.7 (14.9 - 16.4)
	Virus	36.2 (33.8-38.6)	12.1 (10.9-13.4)	23.9 (22.9 - 24.9)	8.1 (7.7 - 8.6)	33.2 (32.2 - 34.1)	11.1 (10.6 - 11.6)
Palau	*E. coli*	6.5 (5.3-7.7)	12.8 (10.6-15.0)	6.1 (5.2 - 7.0)	12.0 (10.5 - 13.5)	5.3 (4.8 - 5.8)	10.4 (9.4 - 11.4)
	Group B *Streptococcus*	23.7 (21.5-26.1)	26.8 (24.2-29.5)	9.9 (9.2 - 10.5)	12.6 (11.6 - 13.4)	7.0 (6.7 - 7.3)	7.8 (7.4 - 8.2)
	H. influenzae	8.1 (6.7-9.7)	5.2 (4.1-6.3)	9.9 (9.2 - 10.6)	5.9 (5.4 - 6.4)	5.0 (4.7 - 5.2)	2.9 (2.7 - 3.1)
	K. pneumoniae	5.8 (4.6-7.2)	19.6 (16.0-23.4)	3.8 (3.1 - 4.7)	13.7 (11.6 - 16.0)	4.1 (3.6 - 4.6)	13.7 (12.2 - 15.0)
	L. monocytogenes	1.9 (1.3-2.6)	4.9 (3.5-6.8)	2.4 (1.8 - 3.0)	6.0 (4.7 - 7.4)	2.5 (2.3 - 2.8)	6.6 (6.0 - 7.3)
	N. meningitidis	7.8 (6.8-8.9)	6.7 (5.8-7.8)	13.1 (12.3 - 13.9)	10.6 (9.8 - 11.4)	13.0 (12.5 - 13.5)	10.8 (10.3 - 11.3)
	Other	2.1 (1.3-3.3)	2.7 (1.7-4.2)	10.1 (7.0 - 14.1)	11.2 (7.9 - 15.3)	10.3 (8.4 - 12.5)	12.8 (10.4 - 15.6)
	S. aureus	4.4 (3.5-5.4)	5.8 (4.7-7.1)	5.3 (4.8 - 5.8)	6.8 (6.1 - 7.5)	5.6 (5.3 - 6.0)	7.6 (7.2 - 8.1)

Location	Aetiology	Neonatal		Under 5		All Ages	
		Nonfatal	Fatal	Nonfatal	Fatal	Nonfatal	Fatal
	S. pneumoniae	2.9 (2.4-3.4)	3.1 (2.6-3.7)	13.2 (12.4 - 14.1)	12.6 (11.7 - 13.5)	14.1 (13.6 - 14.6)	15.8 (15.1 - 16.6)
	Virus	36.8 (33.9-39.7)	12.5 (11.1-14.0)	26.2 (24.9 - 27.4)	8.8 (8.3 - 9.4)	33.0 (32.1 - 33.9)	11.6 (11.1 - 12.1)
Papua New Guinea	*E. coli*	6.2 (4.8-7.9)	11.9 (9.2-14.8)	5.0 (4.0 - 6.1)	9.2 (7.6 - 11.1)	4.5 (3.7 - 5.4)	8.7 (7.2 - 10.3)
	Group B *Streptococcus*	19.9 (17.5-22.5)	22.4 (19.7-25.2)	8.3 (7.6 - 9.1)	9.3 (8.4 - 10.1)	6.9 (6.3 - 7.5)	8.2 (7.4 - 8.9)
	H. influenzae	9.7 (8.3-11.2)	6.6 (5.5-7.8)	11.9 (11.2 - 12.7)	7.5 (6.9 - 8.1)	8.9 (8.3 - 9.4)	6.1 (5.7 - 6.6)
	K. pneumoniae	5.3 (4.0-6.9)	16.8 (13.1-21.1)	3.6 (2.8 - 4.6)	11.2 (8.9 - 14.1)	3.7 (2.9 - 4.5)	11.3 (9.2 - 13.9)
	L. monocytogenes	1.9 (1.3-2.7)	4.6 (3.1-6.5)	1.9 (1.4 - 2.5)	4.5 (3.4 - 5.9)	1.9 (1.5 - 2.4)	4.5 (3.6 - 5.6)
	N. meningitidis	10.2 (9.0-11.9)	8.8 (7.6-10.4)	20.5 (19.3 - 21.6)	16.5 (15.3 - 17.7)	21.1 (20.2 - 22.0)	17.3 (16.2 - 18.3)
	Other	2.3 (1.4-3.5)	3.0 (1.8-4.5)	8.5 (6.0 - 11.9)	9.6 (6.7 - 13.2)	8.8 (7.0 - 11.1)	10.1 (7.9 - 12.8)
	S. aureus	4.2 (3.2-5.5)	5.6 (4.2-7.3)	4.6 (4.1 - 5.2)	5.8 (5.1 - 6.5)	4.8 (4.2 - 5.3)	6.0 (5.3 - 6.7)
	S. pneumoniae	3.1 (2.5-3.7)	4.9 (4.0-5.9)	11.7 (11.1 - 12.5)	17.2 (16.0 - 18.4)	11.9 (11.3 - 12.5)	17.5 (16.5 - 18.6)
	Virus	37.1 (34.1-40.3)	15.5 (13.8-17.4)	23.8 (22.8 - 25.0)	9.3 (8.8 - 9.9)	27.6 (26.7 - 28.6)	10.4 (9.9 - 10.9)
Samoa	*E. coli*	6.6 (5.3-8.0)	12.8 (10.5-15.2)	5.3 (4.5 - 6.2)	10.1 (8.8 - 11.5)	4.8 (4.1 - 5.4)	8.7 (7.5 - 9.8)
	Group B *Streptococcus*	22.6 (20.7-24.6)	25.3 (23.1-27.4)	8.4 (7.8 - 9.0)	10.7 (9.9 - 11.4)	6.3 (5.9 - 6.6)	6.6 (6.2 - 7.0)
	H. influenzae	8.7 (7.5-10.0)	5.6 (4.8-6.5)	10.6 (10.0 - 11.2)	6.1 (5.7 - 6.5)	6.5 (6.2 - 6.8)	3.4 (3.2 - 3.6)
	K. pneumoniae	5.2 (4.1-6.6)	17.0 (13.8-20.8)	3.7 (2.9 - 4.6)	12.1 (10.1 - 14.5)	3.9 (3.3 - 4.6)	12.6 (10.8 - 14.3)
	L. monocytogenes	1.9 (1.3-2.5)	4.7 (3.3-6.4)	2.1 (1.6 - 2.7)	4.9 (3.8 - 6.2)	2.2 (1.9 - 2.5)	5.3 (4.6 - 6.0)
	N. meningitidis	9.6 (8.6-10.7)	8.2 (7.2-9.3)	18.6 (17.6 - 19.5)	14.0 (13.1 - 14.8)	18.2 (17.7 - 18.8)	14.9 (14.2 - 15.6)
	Other	2.1 (1.3-3.2)	2.7 (1.7-4.1)	9.3 (6.5 - 12.8)	9.6 (6.8 - 13.1)	9.7 (8.0 - 11.6)	11.6 (9.7 - 13.6)
	S. aureus	4.1 (3.3-5.2)	5.4 (4.3-6.8)	4.5 (4.1 - 5.0)	5.5 (5.0 - 6.1)	4.8 (4.5 - 5.2)	6.3 (5.8 - 6.8)
	S. pneumoniae	2.9 (2.5-3.4)	5.0 (4.2-5.8)	12.9 (12.2 - 13.6)	18.3 (17.2 - 19.5)	12.2 (11.7 - 12.6)	19.2 (18.3 - 20.0)
	Virus	36.3 (33.7-39.2)	13.4 (12.0-14.9)	24.6 (23.5 - 25.6)	8.7 (8.3 - 9.2)	31.5 (30.6 - 32.4)	11.6 (11.1 - 12.1)
Solomon Islands	*E. coli*	6.4 (5.0-8.0)	12.3 (9.8-15.0)	5.6 (4.5 - 6.7)	10.4 (8.7 - 12.2)	4.6 (3.9 - 5.5)	8.2 (6.9 - 9.6)
	Group B *Streptococcus*	22.1 (19.6-24.9)	24.7 (21.8-27.8)	8.9 (8.1 - 9.7)	10.6 (9.6 - 11.5)	6.8 (6.2 - 7.3)	7.0 (6.4 - 7.5)
	H. influenzae	8.8 (7.3-10.5)	5.9 (4.8-7.1)	8.5 (7.9 - 9.1)	5.2 (4.8 - 5.7)	5.5 (5.2 - 5.9)	2.8 (2.6 - 3.0)
	K. pneumoniae	5.6 (4.3-7.2)	17.8 (14.2-22.1)	3.7 (2.9 - 4.7)	12.0 (9.7 - 14.7)	3.7 (3.0 - 4.5)	12.2 (10.1 - 14.4)
	L. monocytogenes	1.9 (1.3-2.7)	4.7 (3.2-6.7)	2.1 (1.6 - 2.8)	5.0 (3.9 - 6.5)	2.0 (1.7 - 2.4)	4.9 (4.1 - 5.8)
	N. meningitidis	8.6 (7.5-10.0)	7.4 (6.3-8.7)	18.0 (16.9 - 19.2)	14.1 (13.0 - 15.3)	19.2 (18.4 - 20.0)	16.9 (16.0 - 17.8)
	Other	2.3 (1.4-3.4)	2.9 (1.8-4.4)	9.4 (6.7 - 13.0)	10.2 (7.2 - 14.0)	9.4 (7.7 - 11.5)	11.5 (9.6 - 13.7)
	S. aureus	4.3 (3.3-5.5)	5.6 (4.3-7.2)	5.2 (4.5 - 5.9)	6.4 (5.6 - 7.2)	5.1 (4.6 - 5.6)	6.7 (6.0 - 7.4)

Location	Aetiology	Neonatal		Under 5		All Ages	
		Nonfatal	Fatal	Nonfatal	Fatal	Nonfatal	Fatal
	S. pneumoniae	2.9 (2.4-3.5)	4.0 (3.3-4.9)	13.7 (12.8 - 14.7)	16.6 (15.4 - 17.9)	13.6 (12.9 - 14.2)	17.7 (16.8 - 18.7)
	Virus	37.1 (34.0-40.2)	14.6 (13.0-16.4)	24.9 (23.7 - 26.1)	9.4 (8.9 - 10.1)	30.1 (29.1 - 31.1)	12.0 (11.4 - 12.6)
Tokelau	*E. coli*	6.3 (4.9-7.7)	12.4 (9.8-15.0)	4.9 (4.1 - 5.8)	9.7 (8.3 - 11.3)	4.8 (4.2 - 5.5)	9.2 (8.0 - 10.4)
	Group B *Streptococcus*	19.3 (18.1-20.7)	22.1 (20.7-23.6)	7.6 (7.1 - 8.2)	9.2 (8.5 - 9.9)	6.1 (5.7 - 6.4)	6.6 (6.1 - 7.0)
	H. influenzae	9.8 (8.5-11.2)	6.4 (5.4-7.5)	21.2 (20.1 - 22.2)	12.8 (12.0 - 13.7)	12.2 (11.7 - 12.7)	6.4 (6.1 - 6.8)
	K. pneumoniae	5.0 (3.9-6.5)	16.8 (13.4-20.9)	3.5 (2.8 - 4.3)	11.9 (9.8 - 14.5)	3.9 (3.3 - 4.6)	12.9 (11.2 - 14.7)
	L. monocytogenes	1.9 (1.3-2.6)	4.8 (3.3-6.6)	1.9 (1.5 - 2.5)	4.9 (3.8 - 6.2)	2.2 (1.9 - 2.5)	5.6 (4.8 - 6.4)
	N. meningitidis	12.0 (10.9-13.3)	10.5 (9.3-11.8)	16.0 (15.2 - 16.8)	13.5 (12.6 - 14.3)	16.1 (15.6 - 16.6)	13.5 (12.9 - 14.1)
	Other	2.1 (1.2-3.2)	2.7 (1.6-4.2)	8.7 (6.0 - 12.2)	10.1 (7.0 - 14.0)	9.6 (7.8 - 11.6)	11.8 (9.8 - 14.1)
	S. aureus	4.2 (3.3-5.3)	5.6 (4.4-7.0)	4.1 (3.6 - 4.5)	5.3 (4.8 - 5.9)	4.7 (4.4 - 5.0)	6.3 (5.9 - 6.8)
	S. pneumoniae	3.2 (2.7-3.7)	5.4 (4.5-6.3)	8.9 (8.4 - 9.4)	14.2 (13.2 - 15.1)	9.6 (9.2 - 9.9)	16.1 (15.3 - 16.9)
	Virus	36.4 (33.8-39.0)	13.3 (12.0-14.8)	23.3 (22.3 - 24.3)	8.3 (7.8 - 8.9)	30.9 (30.0 - 31.7)	11.5 (11.0 - 12.0)
Tonga	*E. coli*	6.7 (5.5-8.2)	12.9 (10.6-15.4)	5.7 (4.8 - 6.7)	10.6 (9.1 - 12.2)	5.1 (4.4 - 5.9)	9.3 (8.0 - 10.5)
	Group B *Streptococcus*	24.3 (22.0-26.8)	27.0 (24.3-29.7)	9.3 (8.6 - 9.9)	11.4 (10.6 - 12.2)	6.9 (6.4 - 7.3)	7.6 (7.1 - 8.0)
	H. influenzae	8.1 (6.7-9.7)	5.2 (4.3-6.3)	9.1 (8.5 - 9.8)	5.3 (4.9 - 5.8)	5.5 (5.2 - 5.9)	3.1 (2.9 - 3.3)
	K. pneumoniae	5.4 (4.1-6.8)	17.3 (13.8-21.3)	3.8 (3.0 - 4.7)	12.3 (10.2 - 14.7)	3.9 (3.3 - 4.6)	12.1 (10.4 - 13.8)
	L. monocytogenes	1.8 (1.3-2.6)	4.6 (3.2-6.3)	2.2 (1.6 - 2.8)	5.1 (4.0 - 6.4)	2.3 (2.0 - 2.7)	5.4 (4.7 - 6.2)
	N. meningitidis	8.4 (7.4-9.6)	7.1 (6.1-8.3)	17.4 (16.4 - 18.4)	13.1 (12.2 - 14.0)	17.6 (17.0 - 18.3)	13.9 (13.2 - 14.5)
	Other	2.1 (1.3-3.3)	2.7 (1.7-4.1)	9.5 (6.6 - 13.1)	9.8 (6.9 - 13.3)	9.6 (7.9 - 11.7)	10.9 (9.0 - 13.1)
	S. aureus	4.1 (3.2-5.1)	5.3 (4.2-6.7)	4.9 (4.4 - 5.4)	5.9 (5.2 - 6.5)	5.1 (4.7 - 5.5)	6.3 (5.8 - 6.9)
	S. pneumoniae	2.8 (2.4-3.3)	4.6 (3.8-5.4)	12.8 (12.0 - 13.7)	17.7 (16.4 - 19.0)	13.4 (12.8 - 13.9)	20.4 (19.4 - 21.4)
	Virus	36.2 (33.4-39.2)	13.3 (11.9-14.9)	25.4 (24.2 - 26.6)	9.0 (8.5 - 9.5)	30.5 (29.6 - 31.4)	11.0 (10.6 - 11.5)
Tuvalu	*E. coli*	6.6 (5.2-8.0)	12.6 (10.2-15.2)	5.3 (4.4 - 6.2)	9.7 (8.4 - 11.3)	4.8 (4.0 - 5.5)	8.4 (7.2 - 9.7)
	Group B *Streptococcus*	22.1 (20.2-24.3)	24.8 (22.5-27.2)	8.4 (7.7 - 9.0)	10.1 (9.3 - 10.8)	6.2 (5.8 - 6.6)	6.4 (6.0 - 6.9)
	H. influenzae	8.8 (7.6-10.2)	5.8 (4.9-6.8)	10.2 (9.5 - 10.8)	6.0 (5.6 - 6.4)	6.3 (6.0 - 6.6)	3.3 (3.1 - 3.5)
	K. pneumoniae	5.2 (4.0-6.6)	16.8 (13.4-20.8)	3.7 (2.9 - 4.6)	11.8 (9.7 - 14.3)	3.9 (3.3 - 4.6)	12.2 (10.3 - 14.1)
	L. monocytogenes	1.9 (1.3-2.6)	4.6 (3.2-6.4)	2.0 (1.5 - 2.6)	4.8 (3.7 - 6.1)	2.2 (1.8 - 2.5)	5.1 (4.4 - 5.9)
	N. meningitidis	9.7 (8.7-11.0)	8.3 (7.2-9.5)	19.5 (18.5 - 20.4)	14.8 (13.9 - 15.7)	18.9 (18.3 - 19.6)	15.6 (14.9 - 16.3)
	Other	2.2 (1.3-3.3)	2.7 (1.7-4.2)	9.1 (6.4 - 12.6)	9.5 (6.7 - 13.1)	9.5 (7.9 - 11.5)	11.3 (9.5 - 13.3)
	S. aureus	4.1 (3.3-5.2)	5.4 (4.3-6.8)	4.5 (4.1 - 5.0)	5.5 (4.9 - 6.1)	4.9 (4.5 - 5.3)	6.2 (5.7 - 6.8)

Location	Aetiology	Neonatal		Under 5		All Ages	
		Nonfatal	Fatal	Nonfatal	Fatal	Nonfatal	Fatal
	S. pneumoniae	2.9 (2.5-3.5)	5.1 (4.3-6.0)	13.0 (12.3 - 13.7)	19.1 (18.0 - 20.4)	12.4 (12.0 - 12.9)	19.7 (18.8 - 20.6)
	Virus	36.4 (33.8-39.3)	13.8 (12.3-15.4)	24.4 (23.3 - 25.4)	8.8 (8.3 - 9.3)	30.8 (29.9 - 31.7)	11.6 (11.1 - 12.1)
Vanuatu	*E. coli*	6.3 (4.8-8.0)	12.1 (9.3-15.0)	4.9 (4.0 - 6.0)	9.0 (7.5 - 10.7)	4.4 (3.6 - 5.2)	7.8 (6.4 - 9.2)
	Group B *Streptococcus*	20.1 (17.9-22.7)	22.6 (20.0-25.3)	7.8 (7.1 - 8.5)	9.3 (8.4 - 10.1)	6.0 (5.5 - 6.5)	6.4 (5.9 - 7.0)
	H. influenzae	9.6 (8.3-11.1)	6.5 (5.5-7.6)	11.2 (10.5 - 11.9)	6.8 (6.3 - 7.3)	7.4 (6.9 - 7.8)	4.1 (3.8 - 4.4)
	K. pneumoniae	5.2 (3.9-6.7)	16.2 (12.5-20.4)	3.5 (2.7 - 4.6)	11.1 (8.9 - 13.8)	3.7 (3.0 - 4.5)	11.5 (9.4 - 13.7)
	L. monocytogenes	1.9 (1.3-2.6)	4.6 (3.1-6.4)	1.9 (1.4 - 2.5)	4.4 (3.3 - 5.6)	1.9 (1.5 - 2.3)	4.5 (3.7 - 5.4)
	N. meningitidis	10.7 (9.5-12.3)	9.2 (7.9-10.7)	22.1 (21.0 - 23.2)	16.9 (15.8 - 18.0)	22.1 (21.3 - 23.0)	18.3 (17.4 - 19.3)
	Other	2.3 (1.4-3.5)	2.9 (1.8-4.3)	8.6 (6.0 - 11.9)	9.0 (6.3 - 12.4)	9.0 (7.4 - 10.9)	10.5 (8.8 - 12.4)
	S. aureus	4.1 (3.2-5.4)	5.4 (4.1-7.1)	4.4 (3.9 - 5.0)	5.4 (4.7 - 6.0)	4.7 (4.2 - 5.2)	5.9 (5.3 - 6.6)
	S. pneumoniae	3.1 (2.5-3.6)	5.5 (4.5-6.6)	12.3 (11.7 - 13.1)	19.1 (17.9 - 20.4)	11.9 (11.4 - 12.4)	19.3 (18.3 - 20.3)
	Virus	36.7 (33.8-39.8)	15.2 (13.5-17.0)	23.3 (22.3 - 24.4)	9.2 (8.7 - 9.7)	29.0 (28.0 - 30.0)	11.7 (11.1 - 12.2)
**Southeast Asia**	*E. coli*	6.5 (5.2-7.8)	12.5 (10.1-15.0)	5.5 (4.6 - 6.4)	10.1 (8.6 - 11.7)	4.8 (4.2 - 5.5)	9.3 (8.2 - 10.6)
	Group B *Streptococcus*	21.6 (19.9-23.6)	23.9 (21.8-26.1)	8.6 (8.0 - 9.2)	9.7 (9.0 - 10.4)	6.8 (6.4 - 7.2)	7.5 (7.0 - 8.0)
	H. influenzae	9.0 (7.8-10.3)	6.0 (5.1-7.0)	10.7 (10.2 - 11.3)	6.3 (5.9 - 6.7)	7.7 (7.3 - 8.0)	4.5 (4.3 - 4.8)
	K. pneumoniae	5.3 (4.1-6.7)	17.0 (13.6-21.0)	3.7 (3.0 - 4.7)	11.9 (9.7 - 14.5)	3.9 (3.3 - 4.5)	12.4 (10.6 - 14.2)
	L. monocytogenes	1.9 (1.3-2.6)	4.7 (3.3-6.5)	2.1 (1.6 - 2.7)	5.0 (3.8 - 6.3)	2.1 (1.8 - 2.5)	5.4 (4.6 - 6.2)
	N. meningitidis	9.9 (8.9-11.1)	8.8 (7.7-10.0)	17.7 (16.8 - 18.6)	14.4 (13.4 - 15.3)	17.7 (17.1 - 18.3)	14.3 (13.7 - 15.0)
	Other	2.1 (1.3-3.3)	2.8 (1.7-4.2)	9.3 (6.5 - 12.8)	10.1 (7.1 - 13.8)	9.6 (7.9 - 11.7)	11.3 (9.3 - 13.6)
	S. aureus	4.2 (3.3-5.2)	5.5 (4.4-6.9)	4.7 (4.2 - 5.2)	5.8 (5.2 - 6.5)	4.8 (4.5 - 5.1)	6.3 (5.8 - 6.8)
	S. pneumoniae	3.0 (2.6-3.5)	5.0 (4.2-5.8)	12.5 (11.9 - 13.2)	17.8 (16.7 - 19.0)	11.9 (11.5 - 12.3)	18.1 (17.3 - 19.0)
	Virus	36.5 (33.9-39.3)	13.9 (12.5-15.5)	25.0 (23.9 - 26.1)	8.9 (8.4 - 9.4)	30.7 (29.8 - 31.6)	10.8 (10.4 - 11.3)
Cambodia	*E. coli*	6.4 (5.1-7.8)	12.3 (10.0-14.9)	5.7 (4.7 - 6.8)	10.7 (9.0 - 12.6)	4.9 (4.2 - 5.7)	10.3 (8.7 - 12.0)
	Group B *Streptococcus*	21.9 (19.6-24.4)	24.6 (22.0-27.3)	9.1 (8.4 - 9.9)	10.2 (9.3 - 11.1)	7.4 (6.8 - 8.0)	9.5 (8.6 - 10.3)
	H. influenzae	8.9 (7.5-10.4)	5.9 (4.8-7.0)	8.9 (8.3 - 9.6)	5.5 (5.0 - 5.9)	6.7 (6.3 - 7.1)	4.9 (4.5 - 5.3)
	K. pneumoniae	5.6 (4.3-7.1)	18.0 (14.5-22.1)	3.8 (3.0 - 4.8)	12.1 (9.9 - 14.9)	3.8 (3.1 - 4.5)	12.2 (10.1 - 14.7)
	L. monocytogenes	1.9 (1.3-2.7)	4.7 (3.2-6.7)	2.2 (1.6 - 2.8)	5.3 (4.0 - 6.8)	2.1 (1.7 - 2.5)	5.3 (4.2 - 6.5)
	N. meningitidis	8.9 (7.8-10.1)	7.6 (6.6-8.9)	16.8 (15.7 - 17.9)	13.5 (12.5 - 14.6)	17.8 (17.1 - 18.6)	13.9 (13.0 - 14.9)
	Other	2.2 (1.4-3.4)	2.9 (1.8-4.3)	9.6 (6.7 - 13.2)	10.8 (7.6 - 14.8)	9.6 (7.6 - 11.9)	11.0 (8.3 - 14.4)
	S. aureus	4.3 (3.4-5.4)	5.7 (4.4-7.1)	5.2 (4.6 - 5.9)	6.5 (5.7 - 7.4)	5.1 (4.7 - 5.6)	6.6 (5.9 - 7.4)

Location	Aetiology	Neonatal		Under 5		All Ages	
		Nonfatal	Fatal	Nonfatal	Fatal	Nonfatal	Fatal
	S. pneumoniae	3.0 (2.5-3.5)	4.0 (3.3-4.8)	13.2 (12.3 - 14.2)	16.2 (15.0 - 17.5)	13.0 (12.4 - 13.6)	16.5 (15.5 - 17.7)
	Virus	37.0 (34.1-40.0)	14.3 (12.7-16.0)	25.5 (24.2 - 26.7)	9.2 (8.6 - 9.8)	29.6 (28.6 - 30.6)	9.9 (9.3 - 10.4)
Indonesia	*E. coli*	6.4 (5.1-7.8)	12.4 (9.9-15.0)	5.3 (4.4 - 6.2)	9.7 (8.3 - 11.3)	4.7 (4.0 - 5.5)	9.0 (7.7 - 10.3)
	Group B *Streptococcus*	20.8 (19.0-22.8)	23.4 (21.4-25.6)	8.3 (7.7 - 8.9)	9.7 (9.0 - 10.4)	6.8 (6.3 - 7.2)	7.5 (6.9 - 8.0)
	H. influenzae	9.3 (8.1-10.6)	6.1 (5.2-7.1)	10.5 (9.9 - 11.2)	6.2 (5.8 - 6.7)	8.1 (7.7 - 8.6)	4.6 (4.4 - 4.9)
	K. pneumoniae	5.2 (4.0-6.6)	16.7 (13.2-20.6)	3.7 (2.9 - 4.7)	11.8 (9.7 - 14.4)	3.8 (3.1 - 4.5)	12.1 (10.2 - 14.1)
	L. monocytogenes	1.9 (1.3-2.6)	4.7 (3.2-6.5)	2.0 (1.5 - 2.6)	4.8 (3.7 - 6.1)	2.0 (1.6 - 2.4)	5.1 (4.3 - 5.9)
	N. meningitidis	10.6 (9.5-11.9)	9.1 (8.0-10.4)	19.4 (18.4 - 20.4)	15.0 (14.0 - 15.9)	19.6 (18.9 - 20.3)	15.3 (14.6 - 16.0)
	Other	2.2 (1.3-3.3)	2.8 (1.7-4.2)	9.1 (6.3 - 12.3)	9.6 (6.8 - 13.0)	9.3 (7.6 - 11.5)	10.7 (8.9 - 13.0)
	S. aureus	4.1 (3.3-5.2)	5.5 (4.3-6.9)	4.5 (4.1 - 5.0)	5.5 (5.0 - 6.1)	4.6 (4.3 - 5.0)	6.0 (5.5 - 6.6)
	S. pneumoniae	3.0 (2.6-3.5)	5.3 (4.5-6.2)	12.5 (11.8 - 13.2)	18.8 (17.6 - 20.0)	11.8 (11.3 - 12.2)	18.9 (18.0 - 19.8)
	Virus	36.5 (33.9-39.3)	14.0 (12.6-15.7)	24.7 (23.6 - 25.7)	8.9 (8.4 - 9.4)	29.3 (28.4 - 30.2)	10.9 (10.4 - 11.3)
Lao People's Democratic Republic	*E. coli*	6.2 (4.8-7.8)	12.0 (9.4-14.7)	5.3 (4.3 - 6.4)	9.8 (8.2 - 11.7)	4.7 (3.9 - 5.5)	8.9 (7.5 - 10.4)
	Group B *Streptococcus*	20.1 (17.9-22.6)	22.7 (20.2-25.4)	8.7 (8.0 - 9.4)	9.3 (8.4 - 10.2)	7.1 (6.5 - 7.7)	7.7 (7.0 - 8.4)
	H. influenzae	9.6 (8.3-11.1)	6.5 (5.4-7.7)	11.1 (10.4 - 11.9)	7.0 (6.4 - 7.5)	8.3 (7.8 - 8.8)	5.1 (4.8 - 5.5)
	K. pneumoniae	5.5 (4.2-7.0)	17.4 (13.8-21.7)	3.7 (2.9 - 4.7)	11.5 (9.2 - 14.4)	3.7 (3.0 - 4.5)	11.8 (9.7 - 14.2)
	L. monocytogenes	1.9 (1.3-2.7)	4.7 (3.2-6.6)	2.0 (1.5 - 2.6)	4.9 (3.7 - 6.2)	1.9 (1.6 - 2.4)	4.8 (3.9 - 5.9)
	N. meningitidis	9.9 (8.7-11.4)	8.6 (7.4-10.1)	18.6 (17.5 - 19.7)	15.3 (14.2 - 16.5)	19.5 (18.7 - 20.4)	16.4 (15.5 - 17.4)
	Other	2.3 (1.4-3.5)	3.0 (1.8-4.5)	8.9 (6.3 - 12.4)	10.3 (7.3 - 14.2)	9.1 (7.3 - 11.4)	11.0 (8.9 - 13.6)
	S. aureus	4.3 (3.3-5.5)	5.7 (4.4-7.3)	4.9 (4.3 - 5.5)	6.2 (5.4 - 7.0)	4.9 (4.4 - 5.4)	6.4 (5.8 - 7.1)
	S. pneumoniae	3.1 (2.6-3.7)	4.4 (3.7-5.3)	12.1 (11.4 - 12.9)	16.4 (15.3 - 17.7)	12.0 (11.5 - 12.7)	16.8 (15.8 - 17.8)
	Virus	37.2 (34.3-40.2)	15.1 (13.5-17.0)	24.6 (23.5 - 25.8)	9.2 (8.7 - 9.9)	28.7 (27.7 - 29.6)	11.0 (10.4 - 11.5)
Malaysia	*E. coli*	7.0 (5.9-8.3)	13.5 (11.5-15.7)	5.9 (5.0 - 6.8)	10.9 (9.6 - 12.3)	5.1 (4.6 - 5.7)	9.6 (8.7 - 10.6)
	Group B *Streptococcus*	27.2 (25.1-29.5)	30.1 (27.6-32.5)	9.6 (8.9 - 10.3)	12.2 (11.5 - 13.0)	7.4 (7.0 - 7.7)	7.0 (6.6 - 7.4)
	H. influenzae	7.2 (5.8-8.8)	4.4 (3.5-5.5)	6.9 (6.4 - 7.5)	3.9 (3.6 - 4.2)	4.9 (4.5 - 5.2)	2.3 (2.1 - 2.4)
	K. pneumoniae	5.4 (4.2-6.8)	17.7 (14.4-21.8)	3.8 (3.1 - 4.7)	12.7 (10.7 - 14.9)	3.9 (3.4 - 4.4)	12.9 (11.5 - 14.1)
	L. monocytogenes	1.8 (1.3-2.5)	4.6 (3.3-6.3)	2.3 (1.7 - 2.9)	5.3 (4.2 - 6.6)	2.3 (2.0 - 2.6)	6.1 (5.5 - 6.7)
	N. meningitidis	7.4 (6.5-8.5)	6.2 (5.3-7.2)	16.1 (15.2 - 16.9)	11.7 (11.0 - 12.4)	16.0 (15.4 - 16.6)	11.8 (11.2 - 12.3)
	Other	2.0 (1.2-3.1)	2.5 (1.5-3.9)	9.8 (6.9 - 13.6)	9.8 (7.0 - 13.6)	10.0 (8.1 - 12.2)	11.9 (9.7 - 14.4)
	S. aureus	4.0 (3.2-4.9)	5.1 (4.1-6.3)	4.7 (4.2 - 5.1)	5.5 (5.0 - 6.1)	4.8 (4.5 - 5.1)	6.5 (6.1 - 6.9)

Location	Aetiology	Neonatal		Under 5		All Ages	
		Nonfatal	Fatal	Nonfatal	Fatal	Nonfatal	Fatal
	S. pneumoniae	2.7 (2.2-3.2)	4.2 (3.5-5.0)	15.2 (14.3 - 16.1)	19.8 (18.4 - 21.2)	14.0 (13.4 - 14.6)	21.2 (20.3 - 22.1)
	Virus	35.4 (32.5-38.3)	11.6 (10.3-12.9)	25.7 (24.5 - 26.8)	8.0 (7.6 - 8.5)	31.6 (30.7 - 32.5)	10.8 (10.3 - 11.2)
Maldives	*E. coli*	6.8 (5.8-8.0)	13.4 (11.5-15.5)	6.0 (5.2 - 6.8)	11.2 (9.8 - 12.5)	5.0 (4.6 - 5.5)	10.5 (9.7 - 11.4)
	Group B *Streptococcus*	24.8 (23.4-26.3)	27.8 (26.3-29.4)	9.2 (8.7 - 9.8)	10.7 (10.1 - 11.4)	6.9 (6.6 - 7.2)	7.8 (7.4 - 8.1)
	H. influenzae	7.9 (6.8-9.1)	4.8 (4.1-5.7)	9.6 (9.0 - 10.3)	5.4 (4.9 - 5.8)	6.8 (6.4 - 7.1)	3.8 (3.6 - 4.0)
	K. pneumoniae	5.3 (4.2-6.6)	17.8 (14.7-21.6)	3.9 (3.2 - 4.7)	12.9 (11.0 - 15.3)	4.0 (3.5 - 4.4)	13.3 (12.1 - 14.5)
	L. monocytogenes	1.8 (1.3-2.5)	4.8 (3.4-6.3)	2.3 (1.8 - 3.0)	5.7 (4.5 - 7.1)	2.2 (2.0 - 2.5)	6.4 (5.8 - 7.0)
	N. meningitidis	8.8 (8.0-9.7)	7.5 (6.7-8.4)	14.7 (13.9 - 15.5)	11.4 (10.6 - 12.2)	14.8 (14.3 - 15.3)	11.1 (10.6 - 11.5)
	Other	1.9 (1.2-3.0)	2.5 (1.5-3.9)	10.1 (7.1 - 14.0)	10.9 (7.7 - 15.0)	10.3 (8.3 - 12.6)	12.1 (9.7 - 15.0)
	S. aureus	4.1 (3.3-4.9)	5.4 (4.4-6.5)	4.6 (4.2 - 5.1)	5.7 (5.2 - 6.2)	4.7 (4.5 - 4.9)	6.4 (6.0 - 6.7)
	S. pneumoniae	2.8 (2.4-3.3)	4.5 (3.8-5.2)	13.2 (12.4 - 13.9)	18.2 (16.9 - 19.4)	11.8 (11.3 - 12.2)	18.3 (17.5 - 19.1)
	Virus	35.7 (33.2-38.2)	11.4 (10.2-12.7)	26.3 (25.0 - 27.4)	7.8 (7.4 - 8.3)	33.4 (32.4 - 34.3)	10.3 (9.9 - 10.7)
Mauritius	*E. coli*	6.8 (5.7-8.0)	13.3 (11.3-15.4)	6.2 (5.4 - 7.1)	12.0 (10.7 - 13.4)	5.3 (4.8 - 5.8)	10.3 (9.4 - 11.2)
	Group B *Streptococcus*	25.8 (23.9-27.9)	28.8 (26.5-30.9)	10.0 (9.4 - 10.7)	14.8 (13.9 - 15.6)	7.5 (7.1 - 7.8)	8.3 (7.9 - 8.6)
	H. influenzae	7.6 (6.2-9.1)	4.7 (3.8-5.8)	8.7 (8.1 - 9.4)	4.9 (4.5 - 5.4)	5.7 (5.4 - 6.0)	2.9 (2.7 - 3.0)
	K. pneumoniae	5.5 (4.3-6.8)	18.4 (15.0-22.1)	3.9 (3.2 - 4.8)	14.0 (12.1 - 16.1)	4.0 (3.5 - 4.5)	13.4 (12.1 - 14.6)
	L. monocytogenes	1.8 (1.3-2.6)	4.7 (3.3-6.4)	2.4 (1.8 - 3.0)	5.6 (4.5 - 6.9)	2.4 (2.1 - 2.7)	6.3 (5.8 - 6.9)
	N. meningitidis	7.7 (6.7-8.6)	6.5 (5.6-7.4)	13.9 (13.0 - 14.7)	10.1 (9.4 - 10.8)	14.4 (13.9 - 14.9)	10.9 (10.4 - 11.3)
	Other	2.0 (1.2-3.2)	2.6 (1.6-4.0)	10.1 (7.1 - 14.1)	9.5 (6.8 - 13.0)	10.2 (8.2 - 12.5)	11.9 (9.6 - 14.5)
	S. aureus	4.1 (3.4-5.0)	5.4 (4.4-6.6)	5.1 (4.6 - 5.6)	6.1 (5.5 - 6.7)	5.1 (4.8 - 5.4)	6.8 (6.5 - 7.2)
	S. pneumoniae	2.8 (2.4-3.2)	3.8 (3.2-4.4)	13.0 (12.1 - 13.9)	13.9 (12.9 - 14.9)	13.1 (12.6 - 13.6)	18.4 (17.6 - 19.1)
	Virus	35.9 (33.1-38.7)	11.9 (10.6-13.3)	26.7 (25.3 - 27.9)	9.0 (8.4 - 9.5)	32.2 (31.3 - 33.1)	10.9 (10.5 - 11.3)
Myanmar	*E. coli*	6.3 (4.9-7.7)	12.2 (9.7-14.8)	5.5 (4.6 - 6.6)	10.2 (8.7 - 12.1)	4.9 (4.2 - 5.7)	9.8 (8.3 - 11.4)
	Group B *Streptococcus*	20.4 (18.4-22.5)	23.1 (20.9-25.4)	8.8 (8.1 - 9.5)	9.6 (8.8 - 10.4)	7.3 (6.8 - 7.8)	8.6 (7.9 - 9.3)
	H. influenzae	9.4 (8.2-10.8)	6.3 (5.3-7.3)	11.5 (10.7 - 12.2)	7.0 (6.5 - 7.6)	8.8 (8.3 - 9.3)	6.0 (5.6 - 6.5)
	K. pneumoniae	5.4 (4.2-6.8)	17.3 (14.0-21.5)	3.8 (3.0 - 4.8)	11.9 (9.7 - 14.7)	3.8 (3.2 - 4.6)	12.0 (10.1 - 14.4)
	L. monocytogenes	1.9 (1.3-2.6)	4.7 (3.2-6.6)	2.1 (1.6 - 2.7)	5.1 (3.9 - 6.5)	2.0 (1.7 - 2.5)	5.1 (4.1 - 6.3)
	N. meningitidis	10.2 (9.1-11.5)	8.8 (7.7-10.1)	17.4 (16.4 - 18.4)	14.2 (13.1 - 15.3)	18.2 (17.5 - 19.0)	14.6 (13.7 - 15.5)
	Other	2.2 (1.4-3.4)	2.9 (1.8-4.3)	9.2 (6.5 - 12.7)	10.5 (7.4 - 14.5)	9.3 (7.5 - 11.7)	10.8 (8.3 - 13.9)
	S. aureus	4.3 (3.3-5.4)	5.6 (4.4-7.1)	4.9 (4.4 - 5.5)	6.2 (5.4 - 6.9)	4.9 (4.5 - 5.4)	6.3 (5.7 - 7.0)

Location	Aetiology	Neonatal		Under 5		All Ages	
		Nonfatal	Fatal	Nonfatal	Fatal	Nonfatal	Fatal
	S. pneumoniae	3.1 (2.6-3.6)	4.6 (3.9-5.5)	11.5 (10.8 - 12.3)	16.1 (14.9 - 17.3)	11.6 (11.0 - 12.1)	16.5 (15.6 - 17.6)
	Virus	37.0 (34.2-39.9)	14.5 (12.9-16.2)	25.3 (24.2 - 26.5)	9.2 (8.6 - 9.8)	29.1 (28.1 - 30.0)	10.1 (9.6 - 10.6)
Philippines	*E. coli*	6.4 (5.1-7.8)	12.5 (10.1-15.0)	5.6 (4.7 - 6.6)	10.3 (8.7 - 12.1)	4.6 (4.0 - 5.3)	9.0 (7.7 - 10.3)
	Group B *Streptococcus*	21.8 (19.9-24.1)	24.6 (22.3-27.0)	8.7 (8.0 - 9.3)	9.3 (8.5 - 10.1)	6.7 (6.2 - 7.1)	7.4 (6.8 - 7.9)
	H. influenzae	8.9 (7.7-10.3)	5.8 (4.9-6.9)	9.8 (9.2 - 10.5)	5.9 (5.5 - 6.4)	6.6 (6.3 - 7.0)	4.0 (3.8 - 4.3)
	K. pneumoniae	5.4 (4.3-6.9)	17.6 (14.2-21.7)	3.7 (3.0 - 4.7)	11.8 (9.5 - 14.5)	3.8 (3.1 - 4.5)	12.4 (10.4 - 14.4)
	L. monocytogenes	1.9 (1.3-2.6)	4.7 (3.2-6.6)	2.1 (1.6 - 2.8)	5.2 (3.9 - 6.6)	2.0 (1.7 - 2.4)	5.1 (4.3 - 6.0)
	N. meningitidis	9.3 (8.3-10.5)	8.0 (7.0-9.2)	17.4 (16.4 - 18.4)	14.1 (13.1 - 15.1)	18.4 (17.8 - 19.1)	15.6 (14.8 - 16.3)
	Other	2.2 (1.3-3.4)	2.8 (1.8-4.3)	9.6 (6.7 - 13.2)	10.9 (7.7 - 15.0)	9.6 (7.9 - 11.7)	11.7 (9.7 - 14.0)
	S. aureus	4.2 (3.4-5.3)	5.6 (4.4-7.0)	5.0 (4.4 - 5.5)	6.2 (5.5 - 6.9)	4.9 (4.5 - 5.3)	6.5 (5.9 - 7.0)
	S. pneumoniae	3.0 (2.5-3.5)	4.4 (3.7-5.2)	12.9 (12.2 - 13.7)	17.6 (16.4 - 18.8)	12.4 (11.9 - 12.9)	17.4 (16.6 - 18.3)
	Virus	36.8 (34.1-39.7)	14.0 (12.5-15.6)	25.1 (23.9 - 26.3)	8.7 (8.1 - 9.3)	30.9 (30.0 - 31.9)	11.0 (10.5 - 11.5)
Seychelles	*E. coli*	6.8 (5.7-8.0)	13.2 (11.1-15.4)	5.9 (5.0 - 6.7)	11.0 (9.8 - 12.4)	5.3 (4.7 - 5.9)	9.9 (8.8 - 10.9)
	Group B *Streptococcus*	24.6 (22.8-26.4)	27.5 (25.5-29.6)	9.4 (8.8 - 9.9)	12.1 (11.4 - 12.8)	7.2 (6.8 - 7.5)	6.6 (6.3 - 7.0)
	H. influenzae	8.0 (6.8-9.3)	5.0 (4.2-5.9)	8.6 (8.1 - 9.2)	4.9 (4.6 - 5.3)	6.0 (5.7 - 6.3)	2.8 (2.7 - 3.0)
	K. pneumoniae	5.3 (4.2-6.7)	17.6 (14.3-21.4)	3.9 (3.2 - 4.7)	12.9 (11.0 - 15.2)	4.0 (3.5 - 4.6)	12.8 (11.3 - 14.1)
	L. monocytogenes	1.8 (1.3-2.5)	4.7 (3.3-6.3)	2.3 (1.7 - 2.9)	5.3 (4.2 - 6.6)	2.4 (2.1 - 2.7)	6.3 (5.7 - 7.0)
	N. meningitidis	8.6 (7.7-9.6)	7.3 (6.4-8.3)	16.0 (15.1 - 16.8)	11.9 (11.1 - 12.6)	15.7 (15.2 - 16.2)	11.6 (11.1 - 12.1)
	Other	2.0 (1.2-3.1)	2.6 (1.6-4.0)	9.8 (6.8 - 13.5)	9.8 (7.0 - 13.4)	10.0 (8.2 - 12.3)	11.9 (9.5 - 14.5)
	S. aureus	4.1 (3.3-5.0)	5.3 (4.3-6.5)	4.7 (4.3 - 5.2)	5.7 (5.2 - 6.3)	5.0 (4.7 - 5.3)	6.7 (6.2 - 7.1)
	S. pneumoniae	2.8 (2.4-3.3)	4.5 (3.8-5.3)	13.5 (12.8 - 14.2)	17.6 (16.4 - 18.7)	12.9 (12.4 - 13.3)	20.1 (19.1 - 21.0)
	Virus	36.0 (33.3-38.6)	12.3 (11.0-13.6)	26.0 (24.8 - 27.1)	8.6 (8.1 - 9.1)	31.5 (30.7 - 32.3)	11.4 (10.9 - 11.9)
Sri Lanka	*E. coli*	7.0 (6.0-8.2)	13.6 (11.6-15.7)	6.0 (5.2 - 6.8)	11.2 (9.9 - 12.5)	5.1 (4.6 - 5.6)	10.8 (9.7 - 11.9)
	Group B *Streptococcus*	26.9 (25.0-28.8)	29.8 (27.6-31.9)	9.4 (8.8 - 10.1)	12.1 (11.4 - 12.8)	6.8 (6.5 - 7.1)	6.8 (6.4 - 7.2)
	H. influenzae	7.3 (6.0-8.8)	4.5 (3.6-5.5)	7.2 (6.6 - 7.8)	4.0 (3.7 - 4.4)	4.8 (4.5 - 5.0)	2.5 (2.3 - 2.7)
	K. pneumoniae	5.3 (4.2-6.7)	17.8 (14.5-21.8)	3.9 (3.2 - 4.7)	12.9 (11.0 - 15.1)	4.0 (3.6 - 4.5)	12.6 (11.3 - 13.8)
	L. monocytogenes	1.8 (1.3-2.5)	4.6 (3.3-6.3)	2.3 (1.8 - 3.0)	5.5 (4.3 - 6.8)	2.4 (2.1 - 2.6)	7.0 (6.3 - 7.7)
	N. meningitidis	7.7 (6.8-8.7)	6.5 (5.6-7.4)	15.4 (14.5 - 16.2)	11.3 (10.5 - 12.0)	15.0 (14.5 - 15.5)	9.7 (9.2 - 10.1)
	Other	1.9 (1.2-3.1)	2.5 (1.5-3.9)	10.1 (7.0 - 14.1)	10.1 (7.1 - 14.0)	10.3 (8.4 - 12.5)	11.7 (8.8 - 15.1)
	S. aureus	4.0 (3.2-4.9)	5.2 (4.2-6.4)	4.7 (4.2 - 5.1)	5.6 (5.1 - 6.1)	4.9 (4.6 - 5.2)	6.7 (6.2 - 7.1)

Location	Aetiology	Neonatal		Under 5		All Ages	
		Nonfatal	Fatal	Nonfatal	Fatal	Nonfatal	Fatal
	S. pneumoniae	2.7 (2.3-3.2)	4.2 (3.6-5.0)	15.0 (14.0 - 15.8)	19.3 (17.9 - 20.6)	13.3 (12.8 - 13.8)	21.4 (20.2 - 22.4)
	Virus	35.4 (32.6-38.2)	11.4 (10.1-12.7)	26.0 (24.7 - 27.2)	7.9 (7.5 - 8.4)	33.4 (32.4 - 34.2)	10.9 (10.4 - 11.4)
Thailand	*E. coli*	6.4 (5.2-7.8)	12.8 (10.5-15.3)	5.1 (4.4 - 6.0)	10.5 (9.2 - 12.0)	5.0 (4.4 - 5.5)	10.3 (9.3 - 11.4)
	Group B *Streptococcus*	20.4 (19.5-21.4)	23.5 (22.3-24.6)	7.9 (7.4 - 8.4)	10.2 (9.6 - 10.9)	6.0 (5.7 - 6.3)	6.3 (5.9 - 6.7)
	H. influenzae	9.3 (8.0-10.8)	5.9 (4.9-7.0)	22.7 (21.6 - 23.8)	13.2 (12.3 - 14.0)	11.2 (10.8 - 11.6)	5.1 (4.8 - 5.4)
	K. pneumoniae	5.1 (3.9-6.6)	17.4 (14.0-21.6)	3.5 (2.8 - 4.4)	12.8 (10.7 - 15.2)	4.1 (3.6 - 4.6)	13.8 (12.3 - 15.3)
	L. monocytogenes	1.9 (1.3-2.5)	4.9 (3.5-6.7)	2.0 (1.5 - 2.6)	5.4 (4.2 - 6.6)	2.4 (2.1 - 2.6)	6.7 (6.0 - 7.4)
	N. meningitidis	11.6 (10.5-12.7)	10.1 (9.1-11.3)	13.4 (12.6 - 14.0)	11.4 (10.7 - 12.1)	13.5 (13.1 - 14.0)	10.6 (10.1 - 11.1)
	Other	1.9 (1.1-3.0)	2.6 (1.5-4.0)	9.0 (6.2 - 12.7)	10.4 (7.3 - 14.4)	10.2 (8.2 - 12.5)	12.8 (10.1 - 15.9)
	S. aureus	4.2 (3.3-5.2)	5.6 (4.4-6.9)	4.0 (3.6 - 4.4)	5.4 (4.9 - 6.0)	4.8 (4.5 - 5.0)	6.7 (6.3 - 7.2)
	S. pneumoniae	3.1 (2.6-3.6)	5.1 (4.3-5.9)	8.4 (7.9 - 8.9)	12.7 (11.8 - 13.5)	9.3 (8.9 - 9.5)	15.9 (15.0 - 16.6)
	Virus	36.1 (33.8-38.6)	12.0 (10.8-13.3)	23.9 (22.8 - 24.9)	8.0 (7.5 - 8.5)	33.7 (32.7 - 34.6)	11.7 (11.2 - 12.2)
Timor-Leste	*E. coli*	6.4 (5.0-8.0)	12.3 (9.7-15.1)	5.1 (4.2 - 6.1)	9.2 (7.7 - 10.9)	4.6 (3.8 - 5.4)	8.6 (7.3 - 10.1)
	Group B *Streptococcus*	20.9 (18.8-23.3)	23.5 (21.1-25.9)	8.3 (7.6 - 8.9)	8.4 (7.7 - 9.1)	6.7 (6.2 - 7.2)	7.3 (6.7 - 7.8)
	H. influenzae	9.3 (8.1-10.6)	6.2 (5.2-7.2)	10.6 (9.9 - 11.2)	6.3 (5.9 - 6.8)	8.0 (7.5 - 8.4)	5.0 (4.7 - 5.4)
	K. pneumoniae	5.2 (4.0-6.7)	16.5 (13.0-20.6)	3.6 (2.9 - 4.7)	11.0 (8.8 - 13.8)	3.7 (3.0 - 4.5)	11.4 (9.5 - 13.7)
	L. monocytogenes	1.9 (1.3-2.6)	4.6 (3.2-6.4)	2.0 (1.5 - 2.6)	4.6 (3.5 - 6.0)	1.9 (1.6 - 2.3)	4.7 (3.8 - 5.7)
	N. meningitidis	10.4 (9.3-11.8)	8.9 (7.8-10.2)	20.5 (19.4 - 21.5)	16.3 (15.3 - 17.4)	20.8 (20.0 - 21.6)	16.7 (15.8 - 17.6)
	Other	2.2 (1.3-3.4)	2.8 (1.7-4.3)	8.8 (6.1 - 12.1)	9.9 (6.9 - 13.6)	9.1 (7.3 - 11.2)	10.5 (8.4 - 13.1)
	S. aureus	4.1 (3.2-5.3)	5.4 (4.2-6.9)	4.5 (4.0 - 5.0)	5.4 (4.8 - 6.1)	4.6 (4.2 - 5.0)	5.8 (5.2 - 6.3)
	S. pneumoniae	3.0 (2.5-3.6)	5.3 (4.4-6.3)	12.5 (11.8 - 13.2)	20.3 (19.0 - 21.7)	11.9 (11.4 - 12.4)	19.9 (18.9 - 21.0)
	Virus	36.6 (33.9-39.5)	14.4 (12.9-16.2)	24.2 (23.1 - 25.3)	8.5 (8.0 - 9.0)	28.8 (27.8 - 29.7)	10.1 (9.6 - 10.6)
Viet Nam	*E. coli*	6.8 (5.6-8.0)	13.2 (11.1-15.4)	5.8 (5.0 - 6.6)	10.8 (9.5 - 12.2)	5.2 (4.6 - 5.7)	10.0 (9.1 - 11.0)
	Group B *Streptococcus*	24.1 (22.5-25.8)	27.0 (25.2-28.8)	9.3 (8.6 - 9.8)	11.4 (10.7 - 12.0)	7.4 (7.1 - 7.8)	8.1 (7.7 - 8.4)
	H. influenzae	8.1 (7.0-9.4)	5.1 (4.3-6.0)	9.2 (8.6 - 9.8)	5.2 (4.9 - 5.6)	7.0 (6.7 - 7.4)	3.7 (3.5 - 3.9)
	K. pneumoniae	5.3 (4.2-6.6)	17.5 (14.3-21.3)	3.8 (3.2 - 4.7)	12.8 (10.8 - 15.1)	4.0 (3.5 - 4.5)	13.0 (11.6 - 14.4)
	L. monocytogenes	1.8 (1.3-2.5)	4.7 (3.4-6.3)	2.2 (1.7 - 2.8)	5.3 (4.2 - 6.6)	2.2 (1.9 - 2.6)	5.9 (5.3 - 6.6)
	N. meningitidis	9.0 (8.1-10.0)	7.7 (6.8-8.6)	16.2 (15.3 - 16.9)	12.3 (11.5 - 13.0)	16.1 (15.5 - 16.6)	12.2 (11.7 - 12.7)
	Other	2.0 (1.2-3.1)	2.6 (1.6-4.0)	9.7 (6.8 - 13.4)	10.1 (7.2 - 13.8)	9.9 (8.0 - 12.2)	11.5 (9.4 - 14.0)
	S. aureus	4.1 (3.3-5.0)	5.4 (4.4-6.6)	4.6 (4.2 - 5.0)	5.6 (5.1 - 6.2)	4.8 (4.5 - 5.0)	6.3 (5.9 - 6.6)

Location	Aetiology	Neonatal		Under 5		All Ages	
		Nonfatal	Fatal	Nonfatal	Fatal	Nonfatal	Fatal
	S. pneumoniae	2.9 (2.5-3.3)	4.6 (4.0-5.4)	13.3 (12.5 - 14.0)	18.2 (17.0 - 19.4)	12.4 (12.0 - 12.9)	18.9 (18.2 - 19.7)
	Virus	36.0 (33.4-38.6)	12.2 (10.9-13.5)	25.9 (24.7 - 27.0)	8.3 (7.9 - 8.8)	30.9 (30.0 - 31.8)	10.4 (10.1 - 10.8)
**Sub-Saharan Africa**	*E. coli*	6.0 (4.5-7.7)	11.4 (8.7-14.6)	5.4 (4.3 - 6.5)	9.9 (8.2 - 11.8)	4.8 (3.9 - 5.8)	9.0 (7.5 - 10.8)
	Group B *Streptococcus*	20.0 (17.1-23.3)	22.5 (19.1-25.9)	9.0 (8.1 - 9.9)	10.1 (9.1 - 11.2)	7.5 (6.8 - 8.3)	8.2 (7.4 - 9.1)
	H. influenzae	9.9 (8.3-11.8)	6.8 (5.6-8.4)	10.7 (10.0 - 11.5)	7.0 (6.5 - 7.7)	8.1 (7.6 - 8.7)	5.0 (4.6 - 5.5)
	K. pneumoniae	5.4 (4.1-7.0)	16.9 (13.3-21.2)	3.8 (2.9 - 4.9)	11.9 (9.5 - 14.9)	3.8 (3.1 - 4.7)	12.0 (9.8 - 14.5)
	L. monocytogenes	1.9 (1.3-2.8)	4.7 (3.1-6.8)	2.0 (1.5 - 2.7)	4.8 (3.7 - 6.3)	2.0 (1.6 - 2.5)	4.9 (4.0 - 6.1)
	N. meningitidis	9.7 (7.9-11.7)	8.5 (6.8-10.7)	16.2 (14.9 - 17.5)	12.3 (11.0 - 13.6)	17.0 (15.8 - 18.1)	13.3 (12.2 - 14.4)
	Other	2.5 (1.6-3.7)	3.2 (2.1-4.7)	9.1 (6.4 - 12.6)	10.1 (7.1 - 13.7)	9.2 (7.3 - 11.7)	10.8 (8.8 - 13.4)
	S. aureus	4.3 (3.2-5.7)	5.7 (4.2-7.6)	5.1 (4.5 - 5.9)	6.3 (5.5 - 7.3)	5.2 (4.6 - 5.9)	6.7 (5.9 - 7.6)
	S. pneumoniae	3.0 (2.4-3.7)	4.5 (3.6-5.6)	13.5 (12.5 - 14.4)	17.5 (16.2 - 19.0)	13.6 (12.8 - 14.4)	18.3 (17.1 - 19.6)
	Virus	37.3 (34.1-40.7)	15.7 (13.9-17.8)	25.2 (24.0 - 26.5)	10.0 (9.4 - 10.7)	28.6 (27.5 - 29.8)	11.6 (11.0 - 12.2)
**Central Sub-Saharan Africa**	*E. coli*	6.1 (4.6-7.9)	11.6 (8.9-14.7)	5.1 (4.0 - 6.2)	9.6 (8.0 - 11.5)	4.5 (3.6 - 5.4)	8.1 (6.6 - 9.6)
	Group B *Streptococcus*	19.5 (16.8-22.3)	21.8 (19.0-24.9)	8.5 (7.6 - 9.3)	11.1 (10.0 - 12.1)	6.8 (6.2 - 7.5)	7.4 (6.7 - 8.1)
	H. influenzae	9.9 (8.4-11.6)	6.8 (5.6-8.2)	10.4 (9.7 - 11.1)	6.8 (6.2 - 7.4)	7.5 (7.0 - 8.0)	4.0 (3.8 - 4.4)
	K. pneumoniae	5.5 (4.1-7.2)	17.0 (13.2-21.6)	3.6 (2.8 - 4.7)	11.9 (9.6 - 14.6)	3.6 (2.9 - 4.6)	11.7 (9.4 - 14.0)
	L. monocytogenes	1.9 (1.3-2.7)	4.6 (3.1-6.7)	1.9 (1.4 - 2.5)	4.5 (3.5 - 5.8)	1.9 (1.5 - 2.3)	4.6 (3.7 - 5.6)
	N. meningitidis	10.0 (8.6-11.7)	8.7 (7.3-10.4)	20.6 (19.4 - 21.8)	15.7 (14.6 - 16.9)	21.4 (20.4 - 22.5)	17.9 (16.8 - 18.9)
	Other	2.4 (1.5-3.7)	3.1 (1.9-4.6)	8.6 (6.0 - 11.9)	8.7 (6.3 - 11.8)	8.8 (7.0 - 11.0)	10.3 (8.6 - 12.3)
	S. aureus	4.3 (3.2-5.7)	5.7 (4.2-7.6)	4.9 (4.3 - 5.6)	6.0 (5.2 - 6.8)	5.0 (4.4 - 5.6)	6.5 (5.7 - 7.3)
	S. pneumoniae	3.1 (2.5-3.8)	4.5 (3.6-5.5)	12.5 (11.7 - 13.4)	15.1 (14.0 - 16.2)	12.6 (11.9 - 13.3)	17.2 (16.2 - 18.3)
	Virus	37.4 (34.3-40.7)	16.2 (14.3-18.3)	24.0 (22.8 - 25.2)	10.6 (9.9 - 11.3)	27.9 (26.9 - 29.0)	12.3 (11.7 - 13.0)
Angola	*E. coli*	6.2 (4.7-7.8)	11.8 (9.1-14.7)	5.0 (4.0 - 6.1)	9.5 (8.0 - 11.3)	4.4 (3.6 - 5.3)	8.2 (6.9 - 9.7)
	Group B *Streptococcus*	19.5 (17.1-22.2)	22.0 (19.3-24.8)	8.3 (7.5 - 9.1)	10.4 (9.5 - 11.3)	6.8 (6.2 - 7.4)	7.5 (6.9 - 8.2)
	H. influenzae	9.8 (8.5-11.4)	6.7 (5.6-7.9)	12.2 (11.4 - 13.0)	7.6 (7.0 - 8.1)	8.8 (8.2 - 9.3)	4.8 (4.5 - 5.2)
	K. pneumoniae	5.3 (4.0-7.0)	16.8 (13.1-21.1)	3.6 (2.8 - 4.7)	11.7 (9.5 - 14.4)	3.6 (2.9 - 4.5)	11.7 (9.6 - 14.0)
	L. monocytogenes	1.9 (1.3-2.7)	4.6 (3.1-6.5)	1.9 (1.4 - 2.5)	4.5 (3.5 - 5.8)	1.9 (1.5 - 2.3)	4.6 (3.8 - 5.6)
	N. meningitidis	10.4 (9.1-12.1)	9.0 (7.7-10.6)	20.5 (19.3 - 21.6)	15.9 (14.8 - 17.0)	21.3 (20.3 - 22.2)	17.5 (16.5 - 18.5)
	Other	2.3 (1.4-3.6)	3.0 (1.9-4.5)	8.5 (6.0 - 11.8)	9.0 (6.4 - 12.2)	8.8 (7.0 - 11.0)	10.3 (8.6 - 12.3)
	S. aureus	4.3 (3.2-5.6)	5.6 (4.2-7.4)	4.7 (4.1 - 5.3)	5.8 (5.1 - 6.6)	4.8 (4.2 - 5.3)	6.2 (5.5 - 7.0)

Location	Aetiology	Neonatal		Under 5		All Ages	
		Nonfatal	Fatal	Nonfatal	Fatal	Nonfatal	Fatal
	S. pneumoniae	3.1 (2.6-3.7)	4.9 (4.0-5.9)	11.6 (11.0 - 12.3)	15.7 (14.7 - 16.8)	11.8 (11.2 - 12.4)	17.3 (16.4 - 18.3)
	Virus	37.1 (34.2-40.4)	15.7 (13.9-17.7)	23.8 (22.7 - 25.0)	10.0 (9.4 - 10.6)	27.9 (26.9 - 28.9)	11.8 (11.2 - 12.4)
Central African Republic	*E. coli*	5.9 (4.3-7.9)	11.1 (8.1-14.6)	4.6 (3.6 - 5.9)	8.6 (7.0 - 10.7)	4.1 (3.1 - 5.1)	7.4 (5.8 - 9.1)
	Group B *Streptococcus*	17.8 (15.0-21.1)	19.9 (16.8-23.4)	7.7 (6.9 - 8.6)	9.5 (8.4 - 10.6)	6.3 (5.5 - 7.0)	6.9 (6.1 - 7.7)
	H. influenzae	10.6 (8.9-12.7)	7.5 (6.2-9.1)	11.1 (10.3 - 12.0)	7.3 (6.7 - 7.9)	8.0 (7.4 - 8.7)	4.7 (4.3 - 5.1)
	K. pneumoniae	5.3 (3.8-7.2)	16.0 (11.7-20.8)	3.5 (2.6 - 4.7)	10.8 (8.4 - 13.8)	3.5 (2.6 - 4.6)	10.7 (8.3 - 13.4)
	L. monocytogenes	1.9 (1.2-2.8)	4.5 (2.9-6.7)	1.7 (1.3 - 2.3)	4.1 (3.0 - 5.3)	1.7 (1.3 - 2.2)	4.0 (3.1 - 5.1)
	N. meningitidis	11.1 (9.4-13.3)	9.6 (7.9-11.7)	24.2 (22.7 - 25.6)	18.6 (17.2 - 20.0)	25.2 (23.9 - 26.5)	20.5 (19.2 - 21.9)
	Other	2.5 (1.5-3.9)	3.2 (2.0-4.8)	7.9 (5.5 - 11.1)	8.4 (5.9 - 11.5)	8.2 (6.4 - 10.4)	9.5 (7.6 - 11.7)
	S. aureus	4.3 (3.0-5.9)	5.6 (4.0-7.7)	4.6 (4.0 - 5.4)	5.7 (4.9 - 6.6)	4.7 (4.1 - 5.4)	6.1 (5.2 - 7.0)
	S. pneumoniae	3.2 (2.5-4.0)	5.1 (4.0-6.4)	11.8 (11.0 - 12.7)	16.5 (15.3 - 17.8)	11.9 (11.2 - 12.7)	18.0 (16.8 - 19.3)
	Virus	37.4 (34.1-41.0)	17.6 (15.4-20.0)	22.8 (21.6 - 24.0)	10.7 (10.0 - 11.4)	26.4 (25.3 - 27.6)	12.3 (11.6 - 13.0)
Congo	*E. coli*	6.1 (4.7-7.8)	11.8 (9.2-14.7)	5.2 (4.2 - 6.3)	10.2 (8.7 - 12.0)	4.5 (3.7 - 5.4)	8.3 (6.9 - 9.8)
	Group B *Streptococcus*	19.8 (17.2-22.6)	22.4 (19.6-25.4)	8.7 (7.9 - 9.5)	12.7 (11.5 - 13.8)	6.8 (6.2 - 7.4)	7.2 (6.6 - 7.9)
	H. influenzae	9.7 (8.2-11.4)	6.6 (5.5-8.0)	10.5 (9.8 - 11.2)	6.6 (6.1 - 7.2)	7.2 (6.7 - 7.6)	3.5 (3.3 - 3.8)
	K. pneumoniae	5.6 (4.2-7.2)	17.6 (13.8-22.1)	3.7 (2.8 - 4.7)	13.0 (10.7 - 15.4)	3.7 (3.0 - 4.6)	12.2 (9.9 - 14.5)
	L. monocytogenes	1.9 (1.3-2.8)	4.7 (3.1-6.8)	2.0 (1.5 - 2.6)	4.8 (3.8 - 6.0)	1.9 (1.6 - 2.4)	4.9 (4.1 - 5.9)
	N. meningitidis	9.7 (8.4-11.3)	8.4 (7.1-9.9)	19.1 (17.9 - 20.2)	13.9 (13.0 - 14.9)	19.9 (19.0 - 20.9)	16.6 (15.6 - 17.5)
	Other	2.4 (1.5-3.6)	3.0 (1.9-4.6)	8.8 (6.3 - 12.2)	8.4 (6.2 - 11.3)	9.1 (7.3 - 11.2)	10.9 (9.2 - 12.8)
	S. aureus	4.4 (3.3-5.7)	5.8 (4.4-7.5)	5.0 (4.4 - 5.7)	6.2 (5.4 - 7.0)	5.1 (4.6 - 5.7)	6.9 (6.1 - 7.7)
	S. pneumoniae	3.1 (2.5-3.7)	4.2 (3.4-5.1)	12.6 (11.8 - 13.5)	13.1 (12.2 - 14.1)	12.7 (12.1 - 13.4)	16.8 (15.8 - 17.8)
	Virus	37.4 (34.4-40.7)	15.6 (13.8-17.6)	24.4 (23.2 - 25.6)	11.0 (10.3 - 11.7)	29.0 (28.1 - 30.0)	12.7 (12.1 - 13.4)
Democratic Republic of the Congo	*E. coli*	6.1 (4.6-7.9)	11.6 (8.9-14.7)	5.1 (4.1 - 6.3)	9.9 (8.2 - 11.7)	4.5 (3.6 - 5.5)	8.1 (6.7 - 9.7)
	Group B *Streptococcus*	19.6 (16.8-22.5)	22.0 (19.0-25.3)	8.6 (7.7 - 9.4)	11.7 (10.6 - 12.9)	6.9 (6.2 - 7.6)	7.5 (6.7 - 8.2)
	H. influenzae	9.8 (8.2-11.7)	6.8 (5.5-8.2)	9.5 (8.9 - 10.3)	6.2 (5.7 - 6.8)	6.9 (6.5 - 7.4)	3.6 (3.4 - 3.9)
	K. pneumoniae	5.6 (4.1-7.3)	17.3 (13.3-21.9)	3.6 (2.8 - 4.8)	12.3 (10.0 - 15.0)	3.7 (2.9 - 4.6)	11.7 (9.5 - 14.2)
	L. monocytogenes	1.9 (1.3-2.8)	4.6 (3.1-6.8)	1.9 (1.4 - 2.6)	4.6 (3.6 - 5.9)	1.9 (1.5 - 2.4)	4.6 (3.8 - 5.6)
	N. meningitidis	9.7 (8.2-11.4)	8.4 (7.0-10.0)	20.3 (19.1 - 21.6)	15.0 (13.9 - 16.2)	21.2 (20.2 - 22.3)	17.7 (16.6 - 18.8)
	Other	2.4 (1.5-3.7)	3.1 (1.9-4.7)	8.7 (6.1 - 12.1)	8.6 (6.3 - 11.7)	8.8 (7.1 - 11.0)	10.4 (8.7 - 12.4)
	S. aureus	4.4 (3.2-5.8)	5.7 (4.3-7.7)	5.1 (4.4 - 5.8)	6.2 (5.4 - 7.1)	5.1 (4.5 - 5.7)	6.7 (5.9 - 7.5)

Location	Aetiology	Neonatal		Under 5		All Ages	
		Nonfatal	Fatal	Nonfatal	Fatal	Nonfatal	Fatal
	S. pneumoniae	3.1 (2.5-3.8)	4.3 (3.4-5.3)	12.9 (12.1 - 13.9)	14.4 (13.4 - 15.6)	13.0 (12.3 - 13.8)	17.0 (16.0 - 18.2)
	Virus	37.5 (34.4-40.9)	16.2 (14.3-18.4)	24.2 (22.9 - 25.4)	11.0 (10.2 - 11.7)	27.9 (26.9 - 29.0)	12.6 (11.9 - 13.2)
Equatorial Guinea	*E. coli*	6.3 (4.9-7.8)	12.2 (9.6-15.0)	5.1 (4.2 - 6.0)	10.0 (8.7 - 11.6)	4.5 (3.8 - 5.3)	8.6 (7.4 - 9.8)
	Group B *Streptococcus*	19.7 (18.0-21.7)	22.3 (20.3-24.4)	8.2 (7.5 - 8.8)	11.8 (11.0 - 12.6)	6.6 (6.1 - 7.0)	7.5 (7.0 - 8.0)
	H. influenzae	9.7 (8.5-11.1)	6.5 (5.5-7.5)	13.6 (12.8 - 14.3)	7.9 (7.4 - 8.4)	9.9 (9.4 - 10.4)	4.9 (4.7 - 5.2)
	K. pneumoniae	5.1 (3.9-6.6)	16.5 (12.9-20.5)	3.6 (2.9 - 4.6)	12.5 (10.4 - 14.9)	3.7 (3.1 - 4.5)	12.5 (10.5 - 14.4)
	L. monocytogenes	1.9 (1.3-2.6)	4.7 (3.2-6.5)	1.9 (1.5 - 2.5)	4.7 (3.7 - 5.8)	1.9 (1.6 - 2.3)	4.9 (4.2 - 5.7)
	N. meningitidis	11.3 (10.2-12.8)	9.8 (8.6-11.3)	19.4 (18.4 - 20.3)	14.3 (13.5 - 15.2)	19.7 (19.1 - 20.5)	15.9 (15.1 - 16.7)
	Other	2.2 (1.3-3.3)	2.8 (1.7-4.2)	8.7 (6.0 - 11.9)	8.3 (5.9 - 11.1)	9.1 (7.4 - 11.2)	10.7 (9.0 - 12.6)
	S. aureus	4.2 (3.2-5.3)	5.5 (4.3-7.0)	4.4 (3.9 - 4.9)	5.5 (4.9 - 6.1)	4.5 (4.2 - 5.0)	6.1 (5.6 - 6.7)
	S. pneumoniae	3.1 (2.6-3.7)	5.4 (4.6-6.4)	11.0 (10.5 - 11.6)	15.1 (14.2 - 16.0)	10.7 (10.3 - 11.1)	17.1 (16.3 - 17.9)
	Virus	36.6 (33.9-39.4)	14.3 (12.8-16.0)	24.2 (23.2 - 25.3)	10.0 (9.4 - 10.6)	29.2 (28.3 - 30.1)	11.8 (11.3 - 12.4)
Gabon	*E. coli*	6.6 (5.2-8.1)	12.5 (10.1-15.3)	5.2 (4.3 - 6.2)	10.2 (8.9 - 11.7)	4.7 (3.9 - 5.5)	8.5 (7.2 - 9.8)
	Group B *Streptococcus*	22.2 (20.0-24.7)	24.7 (22.2-27.4)	8.7 (8.0 - 9.3)	13.4 (12.4 - 14.5)	6.8 (6.3 - 7.2)	7.2 (6.7 - 7.7)
	H. influenzae	8.8 (7.6-10.3)	5.8 (4.9-6.9)	8.7 (8.1 - 9.2)	5.3 (4.9 - 5.7)	6.2 (5.9 - 6.6)	3.2 (3.0 - 3.3)
	K. pneumoniae	5.3 (4.0-6.8)	16.7 (13.2-20.7)	3.7 (2.9 - 4.7)	12.7 (10.5 - 14.9)	3.8 (3.1 - 4.6)	12.1 (10.1 - 14.0)
	L. monocytogenes	1.9 (1.3-2.6)	4.6 (3.2-6.4)	2.0 (1.5 - 2.6)	4.6 (3.7 - 5.7)	2.0 (1.7 - 2.4)	5.0 (4.3 - 5.8)
	N. meningitidis	9.6 (8.5-11.0)	8.2 (7.0-9.5)	20.4 (19.3 - 21.4)	13.7 (12.9 - 14.6)	20.4 (19.6 - 21.1)	15.4 (14.7 - 16.1)
	Other	2.2 (1.3-3.3)	2.8 (1.7-4.2)	8.8 (6.2 - 12.1)	7.7 (5.6 - 10.4)	9.2 (7.5 - 11.2)	10.6 (8.9 - 12.4)
	S. aureus	4.1 (3.2-5.2)	5.3 (4.2-6.9)	4.5 (4.0 - 5.1)	5.4 (4.8 - 6.1)	4.7 (4.3 - 5.2)	6.2 (5.6 - 6.8)
	S. pneumoniae	2.9 (2.4-3.5)	5.1 (4.2-6.1)	13.5 (12.8 - 14.3)	16.8 (15.7 - 17.9)	12.9 (12.4 - 13.5)	20.2 (19.2 - 21.2)
	Virus	36.5 (33.8-39.6)	14.2 (12.7-15.9)	24.5 (23.4 - 25.5)	10.1 (9.5 - 10.8)	29.3 (28.4 - 30.2)	11.8 (11.3 - 12.3)
**Eastern Sub-Saharan Africa**	*E. coli*	6.1 (4.6-7.8)	11.5 (8.8-14.6)	5.3 (4.3 - 6.5)	9.8 (8.1 - 11.7)	4.7 (3.8 - 5.7)	8.7 (7.2 - 10.3)
	Group B *Streptococcus*	20.3 (17.5-23.5)	22.7 (19.4-26.1)	9.0 (8.1 - 9.9)	10.9 (9.8 - 11.9)	7.3 (6.6 - 8.0)	8.0 (7.2 - 8.7)
	H. influenzae	9.7 (8.0-11.6)	6.7 (5.4-8.2)	9.5 (8.8 - 10.2)	6.2 (5.7 - 6.7)	6.9 (6.5 - 7.4)	4.0 (3.6 - 4.3)
	K. pneumoniae	5.5 (4.2-7.2)	17.2 (13.5-21.6)	3.7 (2.9 - 4.8)	11.9 (9.6 - 14.8)	3.8 (3.0 - 4.7)	11.9 (9.7 - 14.3)
	L. monocytogenes	1.9 (1.3-2.8)	4.7 (3.1-6.9)	2.0 (1.5 - 2.7)	4.7 (3.6 - 6.0)	2.0 (1.6 - 2.5)	4.9 (4.0 - 6.0)
	N. meningitidis	9.3 (7.8-11.0)	8.2 (6.6-10.0)	17.8 (16.6 - 18.9)	14.3 (13.2 - 15.4)	18.3 (17.3 - 19.3)	15.0 (14.0 - 15.9)
	Other	2.5 (1.6-3.7)	3.2 (2.1-4.7)	8.9 (6.3 - 12.5)	9.4 (6.7 - 12.7)	9.1 (7.4 - 11.4)	10.6 (8.8 - 12.8)
	S. aureus	4.4 (3.2-5.8)	5.7 (4.2-7.7)	5.2 (4.5 - 5.9)	6.3 (5.4 - 7.2)	5.2 (4.6 - 5.9)	6.8 (6.0 - 7.7)

Location	Aetiology	Neonatal		Under 5		All Ages	
		Nonfatal	Fatal	Nonfatal	Fatal	Nonfatal	Fatal
	S. pneumoniae	3.0 (2.4-3.7)	4.2 (3.4-5.2)	13.9 (12.9 - 14.9)	16.3 (15.1 - 17.5)	14.0 (13.2 - 14.8)	18.0 (16.9 - 19.2)
	Virus	37.4 (34.1-40.9)	15.9 (14.0-18.0)	24.8 (23.5 - 26.1)	10.3 (9.6 - 10.9)	28.7 (27.6 - 29.7)	12.2 (11.5 - 12.8)
Burundi	*E. coli*	6.2 (4.5-8.1)	11.6 (8.8-15.2)	5.3 (4.2 - 6.5)	10.1 (8.2 - 12.2)	4.7 (3.7 - 5.7)	8.5 (6.9 - 10.3)
	Group B *Streptococcus*	21.3 (17.7-25.5)	23.8 (19.6-28.3)	9.5 (8.4 - 10.5)	11.7 (10.5 - 13.0)	7.8 (6.9 - 8.5)	8.2 (7.3 - 9.0)
	H. influenzae	9.1 (6.8-11.5)	6.2 (4.6-8.1)	6.5 (5.9 - 7.1)	4.4 (4.0 - 5.0)	4.9 (4.5 - 5.3)	2.7 (2.5 - 3.0)
	K. pneumoniae	6.0 (4.4-8.1)	18.6 (14.1-24.0)	3.7 (2.8 - 4.7)	12.1 (9.6 - 15.0)	3.6 (2.8 - 4.6)	11.6 (9.3 - 14.2)
	L. monocytogenes	1.9 (1.2-2.9)	4.6 (3.0-7.1)	2.0 (1.5 - 2.7)	4.8 (3.7 - 6.3)	1.9 (1.5 - 2.5)	4.8 (3.9 - 5.9)
	N. meningitidis	7.7 (6.2-9.5)	6.6 (5.3-8.3)	19.0 (17.7 - 20.4)	14.9 (13.6 - 16.3)	19.8 (18.7 - 21.0)	16.9 (15.8 - 18.1)
	Other	2.5 (1.5-3.8)	3.2 (1.9-4.9)	8.7 (6.1 - 12.1)	9.5 (6.7 - 12.9)	8.8 (6.9 - 11.3)	10.6 (8.7 - 12.9)
	S. aureus	4.5 (3.2-6.1)	5.8 (4.3-7.9)	5.5 (4.7 - 6.4)	6.9 (5.9 - 8.0)	5.5 (4.8 - 6.2)	7.3 (6.3 - 8.3)
	S. pneumoniae	2.9 (2.2-3.8)	3.2 (2.4-4.2)	15.7 (14.4 - 17.0)	14.9 (13.6 - 16.4)	15.8 (14.8 - 17.0)	17.0 (15.8 - 18.3)
	Virus	37.8 (34.1-41.6)	16.3 (14.2-18.7)	24.2 (22.9 - 25.6)	10.6 (9.9 - 11.3)	27.2 (26.1 - 28.4)	12.3 (11.7 - 13.0)
Comoros	*E. coli*	6.5 (5.0-8.4)	12.3 (9.6-15.5)	5.0 (4.1 - 6.1)	9.4 (8.0 - 11.0)	4.5 (3.7 - 5.4)	8.2 (6.8 - 9.7)
	Group B *Streptococcus*	22.0 (19.4-25.0)	24.4 (21.4-27.5)	8.5 (7.8 - 9.3)	11.3 (10.4 - 12.4)	6.4 (5.9 - 7.0)	6.9 (6.3 - 7.5)
	H. influenzae	8.9 (7.4-10.5)	6.0 (4.9-7.2)	7.5 (7.0 - 8.0)	4.7 (4.3 - 5.1)	5.2 (4.9 - 5.6)	3.0 (2.7 - 3.2)
	K. pneumoniae	5.3 (3.9-6.9)	16.4 (12.6-20.6)	3.6 (2.8 - 4.6)	11.5 (9.4 - 14.0)	3.7 (3.0 - 4.6)	11.2 (9.1 - 13.2)
	L. monocytogenes	1.9 (1.3-2.6)	4.5 (3.0-6.4)	1.9 (1.4 - 2.5)	4.3 (3.4 - 5.5)	1.9 (1.6 - 2.4)	4.8 (3.9 - 5.7)
	N. meningitidis	9.4 (8.1-11.1)	8.0 (6.8-9.5)	22.4 (21.2 - 23.6)	15.7 (14.7 - 16.8)	22.3 (21.4 - 23.3)	16.4 (15.5 - 17.3)
	Other	2.3 (1.4-3.5)	2.9 (1.8-4.3)	8.4 (6.0 - 11.7)	8.1 (5.8 - 11.1)	8.9 (7.2 - 10.8)	9.9 (8.2 - 11.8)
	S. aureus	4.1 (3.1-5.3)	5.3 (4.0-7.0)	4.5 (4.0 - 5.1)	5.3 (4.7 - 6.0)	4.8 (4.3 - 5.3)	6.1 (5.4 - 6.8)
	S. pneumoniae	2.9 (2.4-3.5)	5.2 (4.2-6.3)	14.2 (13.4 - 15.0)	19.8 (18.5 - 21.2)	13.5 (12.9 - 14.2)	22.0 (20.8 - 23.2)
	Virus	36.6 (33.6-40.0)	15.1 (13.3-16.9)	23.8 (22.8 - 24.9)	9.7 (9.2 - 10.4)	28.7 (27.7 - 29.6)	11.7 (11.1 - 12.2)
Djibouti	*E. coli*	6.2 (4.8-7.8)	12.0 (9.5-14.8)	5.3 (4.3 - 6.3)	10.0 (8.4 - 11.8)	4.6 (3.8 - 5.5)	8.8 (7.5 - 10.4)
	Group B *Streptococcus*	20.4 (18.0-23.0)	23.0 (20.4-25.8)	8.9 (8.1 - 9.7)	10.6 (9.7 - 11.5)	7.0 (6.4 - 7.6)	7.7 (7.0 - 8.3)
	H. influenzae	9.5 (8.0-11.1)	6.4 (5.3-7.7)	10.8 (10.1 - 11.5)	6.8 (6.3 - 7.3)	7.4 (7.0 - 7.9)	4.2 (4.0 - 4.5)
	K. pneumoniae	5.5 (4.2-7.1)	17.7 (14.0-22.0)	3.7 (2.9 - 4.7)	12.0 (9.8 - 14.8)	3.8 (3.0 - 4.6)	12.1 (10.0 - 14.3)
	L. monocytogenes	1.9 (1.3-2.7)	4.7 (3.2-6.7)	2.0 (1.5 - 2.6)	4.9 (3.8 - 6.2)	2.0 (1.6 - 2.4)	5.1 (4.2 - 6.1)
	N. meningitidis	9.5 (8.3-11.0)	8.2 (7.0-9.7)	18.5 (17.4 - 19.6)	14.8 (13.8 - 15.9)	19.2 (18.4 - 20.0)	15.6 (14.7 - 16.5)
	Other	2.3 (1.4-3.5)	3.0 (1.9-4.5)	8.8 (6.3 - 12.2)	9.8 (7.0 - 13.3)	9.1 (7.4 - 11.2)	10.9 (9.1 - 13.1)
	S. aureus	4.3 (3.3-5.6)	5.7 (4.4-7.4)	4.9 (4.4 - 5.6)	6.2 (5.5 - 7.0)	5.1 (4.6 - 5.6)	6.8 (6.0 - 7.5)

Location	Aetiology	Neonatal		Under 5		All Ages	
		Nonfatal	Fatal	Nonfatal	Fatal	Nonfatal	Fatal
	S. pneumoniae	3.0 (2.5-3.7)	4.2 (3.4-5.1)	12.4 (11.7 - 13.2)	15.2 (14.2 - 16.4)	12.7 (12.1 - 13.3)	17.1 (16.1 - 18.1)
	Virus	37.3 (34.3-40.3)	15.2 (13.5-17.1)	24.6 (23.5 - 25.8)	9.7 (9.2 - 10.4)	29.2 (28.2 - 30.1)	11.7 (11.2 - 12.3)
Eritrea	*E. coli*	6.3 (4.7-8.2)	11.9 (9.1-15.2)	5.3 (4.2 - 6.6)	9.7 (7.9 - 11.9)	4.5 (3.6 - 5.4)	8.0 (6.4 - 9.7)
	Group B *Streptococcus*	21.3 (18.2-24.8)	23.8 (20.2-27.5)	8.9 (8.0 - 9.9)	10.3 (9.2 - 11.4)	6.8 (6.1 - 7.5)	6.9 (6.2 - 7.6)
	H. influenzae	9.2 (7.3-11.2)	6.2 (4.9-7.8)	7.2 (6.6 - 7.8)	4.6 (4.2 - 5.1)	4.9 (4.6 - 5.3)	2.7 (2.5 - 2.9)
	K. pneumoniae	5.7 (4.2-7.5)	17.4 (13.2-22.1)	3.7 (2.8 - 4.8)	11.4 (9.0 - 14.4)	3.6 (2.8 - 4.6)	11.2 (8.9 - 13.6)
	L. monocytogenes	1.9 (1.2-2.8)	4.6 (3.0-6.7)	2.0 (1.5 - 2.7)	4.6 (3.5 - 6.1)	1.9 (1.5 - 2.4)	4.6 (3.7 - 5.7)
	N. meningitidis	8.6 (7.2-10.3)	7.3 (6.1-9.0)	20.4 (19.0 - 21.8)	15.8 (14.5 - 17.2)	21.7 (20.6 - 22.8)	17.8 (16.6 - 18.8)
	Other	2.4 (1.5-3.7)	3.1 (1.9-4.7)	8.8 (6.1 - 12.3)	9.5 (6.7 - 13.2)	8.8 (7.1 - 11.0)	10.4 (8.7 - 12.6)
	S. aureus	4.3 (3.1-5.7)	5.6 (4.1-7.5)	5.2 (4.5 - 6.0)	6.4 (5.4 - 7.4)	5.2 (4.5 - 5.8)	6.7 (5.9 - 7.6)
	S. pneumoniae	3.0 (2.3-3.6)	4.1 (3.2-5.1)	14.3 (13.2 - 15.5)	17.6 (16.1 - 19.1)	14.4 (13.6 - 15.3)	19.6 (18.4 - 20.9)
	Virus	37.4 (34.0-41.0)	16.1 (14.1-18.3)	24.3 (23.0 - 25.6)	10.0 (9.4 - 10.7)	28.2 (27.2 - 29.3)	12.1 (11.4 - 12.7)
Ethiopia	*E. coli*	5.7 (4.0-7.6)	11.0 (8.0-14.5)	5.5 (4.4 - 6.8)	10.3 (8.5 - 12.3)	4.9 (4.0 - 6.0)	9.2 (7.6 - 10.9)
	Group B *Streptococcus*	19.4 (15.4-23.9)	22.1 (17.5-27.2)	9.2 (8.0 - 10.3)	11.9 (10.4 - 13.4)	7.5 (6.6 - 8.4)	8.3 (7.4 - 9.3)
	H. influenzae	10.4 (8.2-13.1)	7.2 (5.5-9.3)	12.0 (11.0 - 13.3)	7.5 (6.7 - 8.3)	8.7 (7.9 - 9.6)	4.7 (4.2 - 5.2)
	K. pneumoniae	5.3 (3.8-7.1)	16.8 (12.6-21.8)	3.9 (3.0 - 5.1)	12.9 (10.3 - 15.9)	4.0 (3.2 - 5.1)	12.8 (10.5 - 15.6)
	L. monocytogenes	1.9 (1.2-2.9)	4.8 (3.0-7.2)	2.1 (1.6 - 2.9)	5.0 (3.8 - 6.5)	2.1 (1.6 - 2.7)	5.2 (4.2 - 6.4)
	N. meningitidis	9.7 (6.8-13.5)	8.6 (5.9-11.9)	11.8 (10.0 - 13.9)	9.4 (8.0 - 11.1)	12.1 (10.3 - 14.2)	9.8 (8.3 - 11.5)
	Other	2.7 (1.7-4.0)	3.5 (2.3-5.1)	9.6 (6.7 - 13.4)	9.6 (6.9 - 13.1)	9.8 (7.8 - 12.4)	11.2 (9.2 - 13.7)
	S. aureus	4.4 (3.0-6.0)	5.9 (4.1-8.1)	5.4 (4.6 - 6.3)	6.5 (5.6 - 7.6)	5.5 (4.8 - 6.4)	7.1 (6.2 - 8.3)
	S. pneumoniae	3.0 (2.2-3.9)	4.3 (3.1-5.6)	14.2 (12.9 - 15.4)	15.8 (14.3 - 17.4)	14.3 (13.1 - 15.5)	18.5 (16.9 - 20.3)
	Virus	37.5 (33.8-41.7)	15.9 (13.8-18.3)	26.2 (24.7 - 27.8)	11.0 (10.2 - 11.8)	31.0 (29.5 - 32.5)	13.1 (12.3 - 13.8)
Kenya	*E. coli*	6.3 (4.8-8.0)	12.0 (9.4-15.0)	5.4 (4.4 - 6.6)	10.5 (8.8 - 12.4)	4.6 (3.7 - 5.4)	8.7 (7.2 - 10.2)
	Group B *Streptococcus*	21.9 (18.8-25.4)	24.6 (20.9-28.3)	9.3 (8.4 - 10.1)	12.8 (11.6 - 14.0)	7.0 (6.3 - 7.6)	7.7 (7.0 - 8.3)
	H. influenzae	8.9 (6.9-11.0)	6.0 (4.6-7.5)	7.5 (6.9 - 8.1)	5.0 (4.5 - 5.5)	4.7 (4.4 - 5.1)	2.6 (2.4 - 2.8)
	K. pneumoniae	5.9 (4.4-7.7)	18.5 (14.4-23.4)	3.7 (2.9 - 4.7)	12.8 (10.4 - 15.4)	3.7 (2.9 - 4.5)	12.0 (9.8 - 14.2)
	L. monocytogenes	1.9 (1.3-2.8)	4.7 (3.1-6.9)	2.1 (1.5 - 2.7)	5.0 (3.9 - 6.3)	2.0 (1.6 - 2.5)	5.1 (4.3 - 6.2)
	N. meningitidis	7.9 (6.7-9.5)	6.8 (5.6-8.3)	18.2 (17.0 - 19.4)	13.6 (12.5 - 14.7)	19.1 (18.2 - 20.0)	15.6 (14.6 - 16.6)
	Other	2.4 (1.5-3.6)	3.1 (1.9-4.6)	9.1 (6.4 - 12.6)	9.2 (6.6 - 12.4)	9.1 (7.5 - 11.3)	11.0 (9.1 - 13.1)
	S. aureus	4.4 (3.3-5.8)	5.8 (4.3-7.6)	5.3 (4.6 - 6.1)	6.6 (5.7 - 7.5)	5.4 (4.8 - 6.0)	7.2 (6.4 - 8.1)

Location	Aetiology	Neonatal		Under 5		All Ages	
		Nonfatal	Fatal	Nonfatal	Fatal	Nonfatal	Fatal
	S. pneumoniae	2.9 (2.3-3.6)	3.4 (2.7-4.3)	14.9 (13.9 - 16.0)	14.1 (13.0 - 15.3)	15.1 (14.2 - 15.9)	17.7 (16.6 - 18.8)
	Virus	37.5 (34.0-41.0)	15.3 (13.4-17.4)	24.5 (23.2 - 25.7)	10.5 (9.8 - 11.2)	29.3 (28.3 - 30.3)	12.5 (11.8 - 13.1)
Madagascar	*E. coli*	6.1 (4.6-7.9)	11.6 (8.9-14.8)	5.3 (4.2 - 6.5)	10.0 (8.3 - 12.0)	4.5 (3.6 - 5.5)	8.3 (6.8 - 10.0)
	Group B *Streptococcus*	20.1 (17.1-23.5)	22.6 (19.1-26.3)	9.0 (8.1 - 10.0)	11.7 (10.5 - 12.9)	7.1 (6.4 - 7.8)	7.7 (6.9 - 8.4)
	H. influenzae	9.6 (7.8-11.6)	6.6 (5.2-8.2)	8.2 (7.6 - 8.9)	5.5 (5.0 - 6.0)	5.8 (5.4 - 6.3)	3.3 (3.0 - 3.5)
	K. pneumoniae	5.8 (4.3-7.6)	17.9 (13.9-22.8)	3.7 (2.8 - 4.8)	12.3 (9.9 - 15.1)	3.7 (2.9 - 4.6)	11.8 (9.5 - 14.3)
	L. monocytogenes	1.9 (1.3-2.8)	4.7 (3.1-6.9)	2.0 (1.5 - 2.6)	4.7 (3.7 - 6.1)	1.9 (1.5 - 2.4)	4.8 (3.9 - 5.8)
	N. meningitidis	8.9 (7.4-10.6)	7.7 (6.3-9.4)	19.4 (18.2 - 20.8)	14.8 (13.6 - 16.1)	20.5 (19.4 - 21.5)	17.1 (16.0 - 18.2)
	Other	2.5 (1.5-3.7)	3.1 (2.0-4.7)	8.7 (6.1 - 12.1)	9.1 (6.5 - 12.4)	8.9 (7.1 - 11.1)	10.6 (8.8 - 12.7)
	S. aureus	4.4 (3.3-5.9)	5.8 (4.3-7.8)	5.3 (4.5 - 6.1)	6.6 (5.6 - 7.5)	5.3 (4.6 - 6.0)	7.0 (6.1 - 7.9)
	S. pneumoniae	3.0 (2.4-3.8)	3.8 (2.9-4.7)	13.9 (12.9 - 15.1)	14.4 (13.3 - 15.7)	14.0 (13.2 - 15.0)	16.9 (15.8 - 18.1)
	Virus	37.7 (34.3-41.2)	16.2 (14.3-18.5)	24.4 (23.2 - 25.8)	10.8 (10.1 - 11.5)	28.3 (27.3 - 29.4)	12.6 (11.9 - 13.2)
Malawi	*E. coli*	6.2 (4.7-8.1)	11.8 (9.2-15.1)	5.4 (4.3 - 6.6)	10.2 (8.4 - 12.3)	4.7 (3.8 - 5.6)	8.7 (7.1 - 10.4)
	Group B *Streptococcus*	21.7 (18.3-25.5)	24.3 (20.4-28.3)	9.5 (8.5 - 10.5)	11.5 (10.3 - 12.6)	7.6 (6.8 - 8.3)	8.2 (7.4 - 9.0)
	H. influenzae	8.9 (6.9-11.2)	6.1 (4.6-7.8)	6.9 (6.4 - 7.6)	4.6 (4.1 - 5.1)	4.9 (4.5 - 5.3)	2.8 (2.6 - 3.1)
	K. pneumoniae	5.9 (4.4-7.9)	18.4 (14.2-23.5)	3.7 (2.9 - 4.8)	12.1 (9.7 - 14.9)	3.7 (2.9 - 4.5)	11.7 (9.5 - 14.2)
	L. monocytogenes	1.9 (1.3-2.9)	4.7 (3.1-6.9)	2.0 (1.5 - 2.7)	4.9 (3.7 - 6.4)	2.0 (1.6 - 2.5)	4.9 (4.0 - 6.0)
	N. meningitidis	7.8 (6.5-9.5)	6.7 (5.5-8.3)	18.5 (17.2 - 19.8)	14.7 (13.5 - 16.0)	19.4 (18.4 - 20.5)	16.3 (15.3 - 17.4)
	Other	2.4 (1.5-3.7)	3.1 (1.9-4.7)	8.8 (6.2 - 12.3)	9.7 (6.9 - 13.2)	8.9 (7.1 - 11.3)	10.7 (8.8 - 13.0)
	S. aureus	4.4 (3.3-5.9)	5.8 (4.3-7.7)	5.4 (4.7 - 6.2)	6.8 (5.8 - 7.8)	5.4 (4.7 - 6.1)	7.1 (6.3 - 8.0)
	S. pneumoniae	2.9 (2.3-3.7)	3.4 (2.6-4.2)	15.1 (14.0 - 16.3)	15.4 (14.2 - 16.8)	15.3 (14.4 - 16.3)	17.3 (16.2 - 18.5)
	Virus	37.6 (34.0-41.2)	15.8 (13.8-17.9)	24.6 (23.3 - 25.9)	10.2 (9.5 - 10.9)	28.2 (27.1 - 29.3)	12.1 (11.4 - 12.7)
Mozambique	*E. coli*	6.1 (4.6-7.9)	11.6 (8.9-14.8)	5.3 (4.2 - 6.6)	9.9 (8.1 - 12.1)	4.7 (3.8 - 5.7)	8.8 (7.1 - 10.5)
	Group B *Streptococcus*	20.2 (17.2-23.6)	22.7 (19.2-26.4)	9.1 (8.1 - 10.0)	10.4 (9.2 - 11.4)	7.4 (6.6 - 8.1)	7.9 (7.1 - 8.7)
	H. influenzae	9.5 (7.8-11.6)	6.5 (5.2-8.1)	7.9 (7.3 - 8.6)	5.1 (4.7 - 5.6)	5.9 (5.5 - 6.4)	3.6 (3.3 - 3.9)
	K. pneumoniae	5.8 (4.3-7.6)	17.9 (13.9-22.8)	3.7 (2.9 - 4.8)	11.7 (9.2 - 14.7)	3.7 (2.9 - 4.6)	11.5 (9.2 - 14.1)
	L. monocytogenes	1.9 (1.3-2.8)	4.7 (3.1-6.9)	2.0 (1.5 - 2.7)	4.8 (3.6 - 6.3)	1.9 (1.5 - 2.5)	4.9 (3.9 - 6.0)
	N. meningitidis	8.9 (7.4-10.6)	7.7 (6.3-9.4)	19.2 (17.9 - 20.6)	15.5 (14.2 - 16.9)	20.2 (19.1 - 21.3)	16.5 (15.5 - 17.6)
	Other	2.5 (1.5-3.7)	3.1 (2.0-4.7)	8.8 (6.2 - 12.3)	9.9 (6.9 - 13.7)	8.9 (7.1 - 11.3)	10.6 (8.7 - 13.1)
	S. aureus	4.4 (3.3-5.9)	5.8 (4.3-7.7)	5.3 (4.6 - 6.2)	6.7 (5.7 - 7.7)	5.3 (4.7 - 6.0)	7.0 (6.1 - 7.9)

Location	Aetiology	Neonatal		Under 5		All Ages	
		Nonfatal	Fatal	Nonfatal	Fatal	Nonfatal	Fatal
	S. pneumoniae	3.0 (2.4-3.8)	3.8 (3.0-4.7)	14.0 (12.9 - 15.2)	15.8 (14.5 - 17.4)	14.0 (13.2 - 15.0)	17.3 (16.1 - 18.6)
	Virus	37.7 (34.3-41.2)	16.2 (14.2-18.4)	24.6 (23.3 - 25.9)	10.1 (9.5 - 10.8)	27.9 (26.8 - 29.0)	11.9 (11.3 - 12.5)
Rwanda	*E. coli*	6.1 (4.6-8.0)	11.7 (8.9-15.0)	5.5 (4.5 - 6.7)	10.7 (8.9 - 12.8)	4.8 (3.9 - 5.8)	9.3 (7.6 - 10.9)
	Group B *Streptococcus*	21.3 (17.8-25.2)	24.0 (20.0-28.3)	9.8 (8.8 - 10.7)	11.9 (10.6 - 13.1)	7.9 (7.1 - 8.6)	8.6 (7.8 - 9.4)
	H. influenzae	9.0 (6.8-11.4)	6.1 (4.5-7.9)	7.0 (6.5 - 7.7)	4.7 (4.2 - 5.2)	5.1 (4.7 - 5.5)	3.0 (2.7 - 3.2)
	K. pneumoniae	6.1 (4.5-8.0)	19.3 (14.9-24.5)	3.7 (2.9 - 4.8)	12.6 (10.2 - 15.4)	3.7 (3.0 - 4.6)	12.2 (10.0 - 14.6)
	L. monocytogenes	1.9 (1.3-2.9)	4.8 (3.1-7.1)	2.1 (1.5 - 2.8)	5.2 (4.0 - 6.8)	2.0 (1.6 - 2.5)	5.3 (4.4 - 6.4)
	N. meningitidis	7.7 (6.3-9.3)	6.6 (5.3-8.1)	16.9 (15.7 - 18.1)	13.8 (12.6 - 15.0)	17.6 (16.6 - 18.6)	15.0 (14.0 - 16.0)
	Other	2.5 (1.5-3.8)	3.1 (1.9-4.8)	9.0 (6.4 - 12.6)	10.2 (7.3 - 13.9)	9.1 (7.3 - 11.5)	11.3 (9.3 - 13.7)
	S. aureus	4.6 (3.3-6.1)	6.0 (4.4-8.0)	5.6 (4.9 - 6.5)	7.2 (6.2 - 8.3)	5.6 (5.0 - 6.3)	7.7 (6.8 - 8.7)
	S. pneumoniae	3.0 (2.2-3.8)	2.9 (2.1-3.8)	15.4 (14.2 - 16.7)	13.6 (12.4 - 14.9)	15.6 (14.6 - 16.7)	15.5 (14.4 - 16.6)
	Virus	37.9 (34.3-41.6)	15.5 (13.6-17.7)	24.9 (23.6 - 26.2)	10.2 (9.5 - 10.9)	28.5 (27.4 - 29.7)	12.2 (11.6 - 12.9)
Somalia	*E. coli*	6.0 (4.3-8.0)	11.3 (8.2-14.8)	4.4 (3.5 - 5.6)	8.1 (6.5 - 10.0)	4.0 (3.1 - 5.0)	7.4 (5.9 - 9.0)
	Group B *Streptococcus*	17.6 (15.1-20.6)	19.7 (16.9-22.9)	7.4 (6.6 - 8.2)	8.5 (7.6 - 9.5)	6.1 (5.4 - 6.7)	6.7 (6.0 - 7.4)
	H. influenzae	10.7 (9.0-12.7)	7.5 (6.1-9.1)	12.9 (11.9 - 13.8)	8.2 (7.5 - 8.8)	9.6 (8.9 - 10.3)	5.8 (5.3 - 6.2)
	K. pneumoniae	5.0 (3.6-6.8)	15.2 (11.2-20.0)	3.4 (2.5 - 4.5)	10.3 (7.9 - 13.1)	3.5 (2.6 - 4.5)	10.5 (8.1 - 13.1)
	L. monocytogenes	1.9 (1.2-2.8)	4.5 (2.8-6.6)	1.7 (1.2 - 2.2)	3.9 (2.9 - 5.1)	1.7 (1.3 - 2.1)	4.0 (3.1 - 5.0)
	N. meningitidis	12.2 (10.5-14.4)	10.5 (8.8-12.7)	25.1 (23.8 - 26.5)	19.6 (18.2 - 21.0)	25.8 (24.6 - 26.9)	20.5 (19.2 - 21.8)
	Other	2.4 (1.4-3.7)	3.0 (1.9-4.6)	7.7 (5.3 - 10.8)	8.2 (5.8 - 11.4)	8.1 (6.4 - 10.3)	9.2 (7.4 - 11.5)
	S. aureus	4.2 (3.0-5.7)	5.4 (3.9-7.4)	4.2 (3.6 - 4.9)	5.2 (4.4 - 6.0)	4.4 (3.8 - 5.0)	5.6 (4.9 - 6.4)
	S. pneumoniae	3.2 (2.5-3.9)	6.0 (4.7-7.4)	11.0 (10.3 - 11.7)	18.3 (17.0 - 19.7)	10.9 (10.3 - 11.5)	19.0 (17.8 - 20.2)
	Virus	36.9 (33.6-40.3)	16.9 (14.9-19.1)	22.3 (21.2 - 23.5)	9.8 (9.2 - 10.4)	26.1 (25.1 - 27.2)	11.5 (10.8 - 12.1)
South Sudan	*E. coli*	6.2 (4.5-8.0)	11.6 (8.6-15.0)	4.7 (3.7 - 5.8)	8.4 (6.9 - 10.3)	4.3 (3.4 - 5.3)	8.0 (6.5 - 9.8)
	Group B *Streptococcus*	18.7 (16.3-21.6)	20.9 (18.1-24.0)	7.7 (6.9 - 8.5)	8.8 (7.9 - 9.6)	6.5 (5.9 - 7.1)	7.6 (6.9 - 8.4)
	H. influenzae	10.2 (8.7-11.9)	7.1 (5.9-8.4)	10.5 (9.8 - 11.2)	6.5 (6.1 - 7.0)	8.3 (7.8 - 8.9)	5.5 (5.1 - 5.9)
	K. pneumoniae	5.1 (3.7-6.8)	15.6 (11.7-20.2)	3.5 (2.6 - 4.6)	10.5 (8.3 - 13.3)	3.6 (2.8 - 4.5)	10.6 (8.4 - 13.2)
	L. monocytogenes	1.9 (1.2-2.7)	4.5 (2.9-6.4)	1.8 (1.3 - 2.4)	4.1 (3.0 - 5.3)	1.8 (1.4 - 2.3)	4.2 (3.2 - 5.3)
	N. meningitidis	11.4 (9.9-13.4)	9.8 (8.3-11.7)	24.3 (23.1 - 25.7)	18.6 (17.4 - 19.9)	24.6 (23.4 - 25.7)	18.9 (17.7 - 20.1)
	Other	2.4 (1.4-3.6)	3.0 (1.8-4.5)	8.1 (5.6 - 11.2)	8.5 (6.0 - 11.8)	8.3 (6.6 - 10.7)	9.1 (7.1 - 11.6)
	S. aureus	4.2 (3.1-5.6)	5.4 (4.0-7.2)	4.4 (3.8 - 5.0)	5.3 (4.6 - 6.0)	4.5 (4.0 - 5.1)	5.5 (4.9 - 6.3)

Location	Aetiology	Neonatal		Under 5		All Ages	
		Nonfatal	Fatal	Nonfatal	Fatal	Nonfatal	Fatal
	S. pneumoniae	3.1 (2.5-3.8)	5.8 (4.7-7.1)	12.2 (11.4 - 12.9)	19.6 (18.3 - 21.0)	11.9 (11.3 - 12.5)	19.9 (18.7 - 21.2)
	Virus	36.9 (33.8-40.2)	16.3 (14.5-18.4)	23.0 (21.9 - 24.1)	9.6 (9.0 - 10.2)	26.3 (25.3 - 27.3)	10.6 (10.0 - 11.2)
Uganda	*E. coli*	6.4 (4.9-8.1)	12.1 (9.5-15.1)	5.4 (4.4 - 6.5)	10.1 (8.4 - 12.0)	4.9 (4.0 - 5.9)	9.1 (7.6 - 10.6)
	Group B *Streptococcus*	21.9 (19.0-25.1)	24.5 (21.1-27.9)	9.3 (8.4 - 10.1)	11.0 (10.0 - 12.0)	7.9 (7.2 - 8.6)	8.7 (7.9 - 9.4)
	H. influenzae	8.9 (7.2-10.8)	6.0 (4.7-7.4)	8.0 (7.4 - 8.6)	5.1 (4.6 - 5.5)	6.3 (5.9 - 6.8)	3.6 (3.4 - 3.9)
	K. pneumoniae	5.7 (4.3-7.4)	17.9 (13.9-22.4)	3.7 (2.9 - 4.7)	11.9 (9.6 - 14.6)	3.7 (2.9 - 4.6)	11.8 (9.6 - 14.1)
	L. monocytogenes	1.9 (1.3-2.8)	4.6 (3.1-6.7)	2.0 (1.5 - 2.7)	4.8 (3.7 - 6.2)	2.0 (1.6 - 2.5)	4.9 (4.0 - 6.0)
	N. meningitidis	8.4 (7.1-9.9)	7.2 (6.0-8.6)	18.9 (17.7 - 20.1)	14.8 (13.7 - 16.0)	19.5 (18.5 - 20.5)	15.8 (14.9 - 16.8)
	Other	2.4 (1.4-3.6)	3.0 (1.9-4.5)	8.9 (6.3 - 12.3)	9.7 (6.9 - 13.2)	9.0 (7.0 - 11.5)	10.4 (8.5 - 12.9)
	S. aureus	4.3 (3.2-5.6)	5.6 (4.3-7.3)	5.2 (4.5 - 5.9)	6.4 (5.5 - 7.2)	5.2 (4.6 - 5.8)	6.7 (5.9 - 7.4)
	S. pneumoniae	2.9 (2.4-3.6)	3.9 (3.1-4.8)	14.0 (13.1 - 15.0)	16.5 (15.2 - 17.8)	14.2 (13.4 - 15.1)	17.9 (16.8 - 19.0)
	Virus	37.3 (33.9-40.6)	15.3 (13.5-17.3)	24.7 (23.5 - 25.9)	9.8 (9.2 - 10.4)	27.4 (26.3 - 28.4)	11.2 (10.7 - 11.8)
United Republic of Tanzania	*E. coli*	6.2 (4.8-7.9)	11.9 (9.3-14.8)	5.5 (4.5 - 6.7)	10.4 (8.7 - 12.3)	4.9 (4.0 - 5.9)	9.2 (7.7 - 10.7)
	Group B *Streptococcus*	21.0 (18.1-24.2)	23.6 (20.3-27.0)	9.3 (8.4 - 10.2)	11.6 (10.5 - 12.7)	7.8 (7.0 - 8.5)	8.5 (7.7 - 9.3)
	H. influenzae	9.2 (7.5-11.1)	6.2 (5.0-7.7)	8.2 (7.6 - 8.8)	5.3 (4.8 - 5.8)	6.3 (5.9 - 6.8)	3.6 (3.3 - 3.9)
	K. pneumoniae	5.8 (4.4-7.6)	18.2 (14.3-22.8)	3.7 (2.9 - 4.8)	12.5 (10.1 - 15.2)	3.7 (3.0 - 4.6)	12.1 (10.0 - 14.4)
	L. monocytogenes	1.9 (1.3-2.8)	4.7 (3.2-6.9)	2.1 (1.6 - 2.7)	5.0 (3.8 - 6.4)	2.0 (1.6 - 2.5)	5.1 (4.2 - 6.2)
	N. meningitidis	8.6 (7.4-10.2)	7.5 (6.2-9.0)	18.0 (16.8 - 19.2)	14.0 (12.9 - 15.3)	18.7 (17.8 - 19.7)	15.3 (14.4 - 16.2)
	Other	2.4 (1.5-3.6)	3.0 (1.9-4.6)	9.0 (6.4 - 12.5)	9.7 (6.9 - 13.2)	9.1 (7.2 - 11.6)	10.7 (8.8 - 13.0)
	S. aureus	4.4 (3.3-5.8)	5.8 (4.4-7.6)	5.3 (4.6 - 6.1)	6.7 (5.8 - 7.6)	5.3 (4.7 - 6.0)	7.0 (6.2 - 7.8)
	S. pneumoniae	3.0 (2.4-3.7)	3.7 (2.9-4.5)	13.8 (12.9 - 14.9)	14.6 (13.5 - 15.9)	14.0 (13.2 - 14.9)	16.6 (15.6 - 17.7)
	Virus	37.5 (34.2-40.9)	15.4 (13.6-17.4)	25.0 (23.8 - 26.3)	10.3 (9.6 - 10.9)	28.1 (27.1 - 29.2)	11.9 (11.3 - 12.5)
Zambia	*E. coli*	6.4 (4.9-8.1)	12.1 (9.5-15.1)	5.5 (4.5 - 6.8)	10.5 (8.8 - 12.5)	4.8 (3.9 - 5.6)	8.7 (7.2 - 10.2)
	Group B *Streptococcus*	22.5 (19.4-25.9)	25.1 (21.5-28.7)	9.6 (8.7 - 10.5)	12.0 (10.9 - 13.1)	7.6 (6.9 - 8.2)	7.9 (7.2 - 8.6)
	H. influenzae	8.7 (6.8-10.7)	5.8 (4.5-7.3)	7.1 (6.5 - 7.7)	4.6 (4.2 - 5.1)	5.0 (4.7 - 5.4)	2.7 (2.5 - 2.9)
	K. pneumoniae	5.8 (4.4-7.6)	18.3 (14.3-23.2)	3.7 (2.9 - 4.8)	12.4 (10.1 - 15.1)	3.7 (3.0 - 4.6)	12.0 (9.9 - 14.2)
	L. monocytogenes	1.9 (1.3-2.8)	4.7 (3.1-6.8)	2.1 (1.6 - 2.8)	5.0 (3.9 - 6.4)	2.0 (1.6 - 2.5)	5.0 (4.2 - 6.0)
	N. meningitidis	7.8 (6.6-9.3)	6.7 (5.5-8.1)	17.9 (16.7 - 19.1)	13.8 (12.7 - 15.0)	18.9 (18.0 - 19.8)	15.9 (15.0 - 16.9)
	Other	2.4 (1.4-3.6)	3.0 (1.9-4.6)	9.1 (6.4 - 12.6)	9.6 (6.8 - 13.2)	9.1 (7.3 - 11.4)	11.0 (9.1 - 13.1)
	S. aureus	4.4 (3.3-5.7)	5.7 (4.3-7.4)	5.4 (4.6 - 6.1)	6.6 (5.7 - 7.5)	5.3 (4.7 - 5.9)	7.0 (6.2 - 7.8)

Location	Aetiology	Neonatal		Under 5		All Ages	
		Nonfatal	Fatal	Nonfatal	Fatal	Nonfatal	Fatal
	S. pneumoniae	2.9 (2.3-3.6)	3.5 (2.8-4.3)	14.7 (13.7 - 15.9)	15.4 (14.2 - 16.7)	14.8 (14.0 - 15.7)	17.7 (16.7 - 18.9)
	Virus	37.3 (33.9-40.7)	15.1 (13.2-17.1)	25.0 (23.7 - 26.2)	10.1 (9.4 - 10.7)	28.8 (27.8 - 29.8)	12.0 (11.4 - 12.6)
**Southern Sub-Saharan Africa**	*E. coli*	6.2 (4.8-7.8)	12.0 (9.5-14.8)	5.5 (4.5 - 6.6)	10.4 (8.9 - 12.3)	4.7 (4.0 - 5.5)	8.9 (7.6 - 10.4)
	Group B *Streptococcus*	21.2 (18.5-24.1)	23.9 (20.8-27.1)	9.3 (8.5 - 10.0)	11.2 (10.2 - 12.1)	6.8 (6.3 - 7.3)	7.3 (6.7 - 7.9)
	H. influenzae	9.1 (7.5-10.9)	6.1 (4.9-7.5)	9.4 (8.8 - 10.1)	5.8 (5.4 - 6.3)	5.6 (5.2 - 5.9)	3.1 (2.9 - 3.3)
	K. pneumoniae	5.7 (4.4-7.4)	18.4 (14.6-22.9)	3.7 (2.9 - 4.7)	12.4 (10.1 - 15.0)	3.8 (3.1 - 4.6)	12.3 (10.3 - 14.4)
	L. monocytogenes	1.9 (1.3-2.8)	4.7 (3.2-6.9)	2.1 (1.6 - 2.7)	5.1 (4.0 - 6.5)	2.1 (1.8 - 2.5)	5.4 (4.5 - 6.4)
	N. meningitidis	8.6 (7.5-10.1)	7.5 (6.3-8.9)	17.1 (16.1 - 18.2)	14.0 (12.9 - 15.1)	17.5 (16.8 - 18.3)	15.2 (14.3 - 16.1)
	Other	2.3 (1.4-3.5)	3.0 (1.9-4.5)	9.2 (6.6 - 12.8)	10.1 (7.2 - 13.8)	9.5 (7.8 - 11.3)	11.6 (9.7 - 13.6)
	S. aureus	4.4 (3.4-5.6)	5.8 (4.5-7.4)	5.2 (4.6 - 5.9)	6.6 (5.8 - 7.5)	5.4 (4.9 - 5.9)	7.2 (6.5 - 8.0)
	S. pneumoniae	3.0 (2.4-3.6)	3.6 (2.9-4.4)	13.6 (12.7 - 14.5)	14.7 (13.6 - 15.9)	13.9 (13.3 - 14.6)	16.7 (15.7 - 17.7)
	Virus	37.4 (34.2-40.6)	15.0 (13.3-16.9)	24.9 (23.7 - 26.1)	9.8 (9.2 - 10.4)	30.7 (29.8 - 31.7)	12.4 (11.8 - 13.0)
Botswana	*E. coli*	6.3 (5.0-7.6)	12.3 (10.0-14.7)	5.8 (4.9 - 6.8)	11.1 (9.5 - 13.0)	4.8 (4.2 - 5.5)	9.4 (8.2 - 10.6)
	Group B *Streptococcus*	21.1 (19.0-23.5)	24.1 (21.6-26.6)	9.3 (8.6 - 10.0)	10.7 (9.7 - 11.5)	6.9 (6.4 - 7.3)	7.5 (7.0 - 8.0)
	H. influenzae	9.1 (7.7-10.6)	6.0 (4.9-7.1)	10.4 (9.7 - 11.1)	6.4 (5.9 - 6.9)	6.6 (6.2 - 6.9)	3.8 (3.6 - 4.0)
	K. pneumoniae	5.7 (4.4-7.1)	18.7 (15.3-22.8)	3.8 (3.1 - 4.7)	12.7 (10.5 - 15.4)	3.9 (3.3 - 4.5)	13.1 (11.2 - 14.9)
	L. monocytogenes	1.9 (1.3-2.7)	4.9 (3.4-6.8)	2.2 (1.7 - 2.8)	5.5 (4.3 - 7.0)	2.2 (1.8 - 2.5)	5.7 (4.9 - 6.5)
	N. meningitidis	9.1 (8.0-10.3)	7.9 (6.8-9.1)	15.2 (14.2 - 16.1)	12.6 (11.6 - 13.6)	16.1 (15.5 - 16.7)	13.9 (13.1 - 14.6)
	Other	2.2 (1.3-3.4)	2.9 (1.8-4.4)	9.6 (6.8 - 13.3)	11.2 (7.9 - 15.3)	9.8 (8.1 - 11.8)	12.3 (10.4 - 14.6)
	S. aureus	4.4 (3.5-5.5)	5.9 (4.7-7.3)	5.2 (4.7 - 5.9)	6.7 (6.0 - 7.5)	5.3 (4.9 - 5.7)	7.2 (6.6 - 7.8)
	S. pneumoniae	3.0 (2.5-3.6)	3.6 (3.0-4.3)	12.6 (11.8 - 13.5)	14.0 (12.9 - 15.2)	12.6 (12.0 - 13.1)	15.2 (14.4 - 16.0)
	Virus	37.2 (34.4-40.2)	13.9 (12.4-15.5)	25.8 (24.6 - 27.1)	9.1 (8.5 - 9.8)	32.0 (31.0 - 32.9)	12.0 (11.4 - 12.5)
Eswatini	*E. coli*	6.3 (4.9-7.9)	12.1 (9.5-14.9)	5.5 (4.5 - 6.7)	10.2 (8.4 - 12.2)	4.5 (3.7 - 5.3)	8.5 (7.0 - 10.0)
	Group B *Streptococcus*	21.4 (18.7-24.3)	24.0 (21.0-27.2)	9.2 (8.3 - 10.0)	9.9 (8.9 - 10.9)	6.5 (6.0 - 7.1)	7.0 (6.4 - 7.6)
	H. influenzae	9.1 (7.5-10.8)	6.1 (4.9-7.4)	8.5 (7.9 - 9.2)	5.3 (4.9 - 5.8)	5.2 (4.9 - 5.5)	3.1 (2.9 - 3.3)
	K. pneumoniae	5.6 (4.3-7.3)	17.9 (14.1-22.2)	3.7 (2.9 - 4.8)	11.7 (9.3 - 14.6)	3.7 (3.0 - 4.5)	11.9 (9.7 - 14.2)
	L. monocytogenes	1.9 (1.3-2.7)	4.7 (3.2-6.8)	2.1 (1.6 - 2.8)	5.0 (3.8 - 6.5)	2.0 (1.6 - 2.4)	5.0 (4.1 - 6.0)
	N. meningitidis	8.8 (7.6-10.3)	7.6 (6.4-9.0)	18.2 (17.0 - 19.3)	14.8 (13.6 - 16.0)	19.5 (18.7 - 20.3)	16.5 (15.5 - 17.4)
	Other	2.3 (1.4-3.5)	3.0 (1.9-4.5)	9.1 (6.5 - 12.6)	10.4 (7.4 - 14.4)	9.2 (7.7 - 11.1)	11.3 (9.4 - 13.3)
	S. aureus	4.3 (3.3-5.6)	5.7 (4.3-7.3)	5.2 (4.6 - 5.9)	6.5 (5.6 - 7.5)	5.2 (4.7 - 5.7)	6.9 (6.1 - 7.6)

Location	Aetiology	Neonatal		Under 5		All Ages	
		Nonfatal	Fatal	Nonfatal	Fatal	Nonfatal	Fatal
	S. pneumoniae	3.0 (2.4-3.6)	4.0 (3.2-4.8)	13.5 (12.6 - 14.6)	16.7 (15.5 - 18.1)	13.6 (12.9 - 14.2)	17.8 (16.8 - 18.9)
	Virus	37.3 (34.1-40.4)	15.1 (13.3-17.0)	25.0 (23.8 - 26.3)	9.4 (8.8 - 10.1)	30.6 (29.6 - 31.6)	12.1 (11.5 - 12.6)
Lesotho	*E. coli*	6.3 (4.8-8.2)	12.0 (9.3-15.1)	5.4 (4.3 - 6.7)	9.9 (8.1 - 12.0)	4.3 (3.5 - 5.2)	8.0 (6.5 - 9.6)
	Group B *Streptococcus*	21.5 (18.6-24.7)	24.0 (20.6-27.4)	9.0 (8.0 - 9.9)	10.2 (9.1 - 11.2)	6.2 (5.6 - 6.8)	6.5 (5.8 - 7.1)
	H. influenzae	9.1 (7.4-11.0)	6.2 (4.9-7.6)	7.5 (6.8 - 8.1)	4.7 (4.3 - 5.2)	4.5 (4.2 - 4.8)	2.6 (2.4 - 2.8)
	K. pneumoniae	5.6 (4.2-7.3)	17.4 (13.5-22.0)	3.7 (2.9 - 4.8)	11.5 (9.2 - 14.4)	3.7 (2.9 - 4.5)	11.4 (9.1 - 13.7)
	L. monocytogenes	1.9 (1.3-2.8)	4.6 (3.1-6.7)	2.0 (1.5 - 2.7)	4.8 (3.6 - 6.2)	1.9 (1.6 - 2.4)	4.8 (3.9 - 5.8)
	N. meningitidis	8.7 (7.4-10.4)	7.5 (6.2-9.0)	19.6 (18.2 - 20.9)	15.3 (14.1 - 16.7)	21.2 (20.2 - 22.2)	17.5 (16.4 - 18.5)
	Other	2.4 (1.4-3.6)	3.0 (1.9-4.5)	9.0 (6.3 - 12.6)	9.8 (6.8 - 13.7)	9.0 (7.5 - 10.8)	10.8 (9.0 - 12.7)
	S. aureus	4.3 (3.2-5.6)	5.6 (4.2-7.4)	5.3 (4.5 - 6.0)	6.4 (5.5 - 7.4)	5.1 (4.6 - 5.8)	6.7 (5.9 - 7.6)
	S. pneumoniae	3.0 (2.4-3.6)	4.2 (3.3-5.1)	13.9 (12.9 - 15.1)	17.6 (16.1 - 19.1)	14.0 (13.3 - 14.8)	19.5 (18.4 - 20.7)
	Virus	37.3 (34.0-40.7)	15.6 (13.8-17.7)	24.7 (23.4 - 26.0)	9.8 (9.1 - 10.4)	30.1 (29.0 - 31.1)	12.3 (11.7 - 12.9)
Namibia	*E. coli*	6.4 (5.1-7.8)	12.3 (10.0-14.9)	5.7 (4.7 - 6.8)	10.8 (9.1 - 12.6)	4.9 (4.1 - 5.7)	9.2 (7.9 - 10.5)
	Group B *Streptococcus*	22.1 (19.7-24.7)	24.8 (22.1-27.6)	9.3 (8.5 - 10.1)	10.7 (9.8 - 11.6)	7.0 (6.5 - 7.5)	7.2 (6.6 - 7.8)
	H. influenzae	8.8 (7.4-10.4)	5.8 (4.8-7.0)	8.7 (8.1 - 9.4)	5.3 (4.9 - 5.8)	5.7 (5.4 - 6.0)	3.1 (2.9 - 3.3)
	K. pneumoniae	5.6 (4.4-7.1)	18.2 (14.6-22.4)	3.8 (3.0 - 4.8)	12.3 (10.1 - 15.0)	3.9 (3.2 - 4.6)	12.3 (10.4 - 14.3)
	L. monocytogenes	1.9 (1.3-2.7)	4.7 (3.2-6.7)	2.2 (1.6 - 2.8)	5.3 (4.1 - 6.8)	2.2 (1.8 - 2.6)	5.5 (4.7 - 6.5)
	N. meningitidis	8.6 (7.5-9.9)	7.4 (6.4-8.7)	16.6 (15.5 - 17.7)	13.3 (12.3 - 14.4)	17.4 (16.7 - 18.1)	14.4 (13.6 - 15.1)
	Other	2.2 (1.4-3.4)	2.9 (1.8-4.3)	9.5 (6.7 - 13.2)	10.6 (7.6 - 14.6)	9.6 (7.8 - 11.6)	11.6 (9.7 - 13.8)
	S. aureus	4.3 (3.4-5.4)	5.7 (4.5-7.2)	5.3 (4.6 - 5.9)	6.6 (5.8 - 7.4)	5.3 (4.8 - 5.7)	7.0 (6.4 - 7.7)
	S. pneumoniae	2.9 (2.4-3.5)	3.8 (3.2-4.6)	13.4 (12.5 - 14.4)	15.8 (14.6 - 17.0)	13.5 (12.9 - 14.1)	17.7 (16.8 - 18.7)
	Virus	37.1 (34.1-40.1)	14.2 (12.7-15.9)	25.5 (24.3 - 26.8)	9.3 (8.7 - 9.9)	30.7 (29.7 - 31.6)	12.0 (11.4 - 12.5)
South Africa	*E. coli*	6.3 (4.9-7.7)	12.2 (9.8-14.9)	5.5 (4.6 - 6.6)	10.8 (9.2 - 12.5)	4.9 (4.2 - 5.6)	9.4 (8.1 - 10.7)
	Group B *Streptococcus*	21.7 (19.1-24.5)	24.5 (21.6-27.5)	9.3 (8.6 - 10.1)	11.7 (10.8 - 12.6)	6.8 (6.4 - 7.3)	7.4 (6.8 - 8.0)
	H. influenzae	8.9 (7.3-10.6)	5.9 (4.7-7.1)	10.1 (9.4 - 10.7)	6.3 (5.8 - 6.8)	5.6 (5.3 - 5.9)	3.1 (2.9 - 3.3)
	K. pneumoniae	5.8 (4.5-7.4)	18.9 (15.2-23.2)	3.7 (3.0 - 4.6)	12.8 (10.6 - 15.4)	3.9 (3.2 - 4.6)	12.7 (10.7 - 14.6)
	L. monocytogenes	1.9 (1.3-2.8)	4.8 (3.3-6.9)	2.1 (1.6 - 2.8)	5.3 (4.2 - 6.8)	2.2 (1.9 - 2.6)	5.8 (5.0 - 6.7)
	N. meningitidis	8.4 (7.3-9.7)	7.3 (6.2-8.6)	16.1 (15.1 - 17.1)	13.0 (12.0 - 14.0)	16.3 (15.6 - 16.9)	13.8 (13.0 - 14.6)
	Other	2.3 (1.4-3.5)	2.9 (1.8-4.4)	9.4 (6.6 - 13.0)	10.4 (7.5 - 14.1)	9.6 (8.0 - 11.6)	11.9 (10.0 - 14.1)
	S. aureus	4.4 (3.5-5.6)	5.9 (4.6-7.4)	5.2 (4.6 - 5.8)	6.6 (5.9 - 7.4)	5.5 (5.0 - 6.0)	7.4 (6.7 - 8.1)

Location	Aetiology	Neonatal		Under 5		All Ages	
		Nonfatal	Fatal	Nonfatal	Fatal	Nonfatal	Fatal
	S. pneumoniae	3.0 (2.4-3.6)	3.4 (2.7-4.1)	13.5 (12.7 - 14.3)	13.6 (12.6 - 14.7)	14.0 (13.4 - 14.7)	16.2 (15.3 - 17.2)
	Virus	37.3 (34.3-40.4)	14.2 (12.6-16.0)	25.0 (23.8 - 26.2)	9.5 (8.9 - 10.1)	31.2 (30.2 - 32.1)	12.4 (11.8 - 13.0)
Zimbabwe	*E. coli*	6.2 (4.7-7.9)	11.8 (9.2-14.8)	5.3 (4.3 - 6.5)	10.1 (8.3 - 12.0)	4.5 (3.6 - 5.4)	8.3 (6.8 - 10.0)
	Group B *Streptococcus*	20.6 (17.7-23.8)	23.2 (19.8-26.6)	9.1 (8.2 - 10.0)	10.9 (9.9 - 11.9)	6.7 (6.1 - 7.4)	7.2 (6.5 - 7.9)
	H. influenzae	9.4 (7.7-11.3)	6.4 (5.1-7.9)	8.5 (7.9 - 9.1)	5.5 (5.0 - 6.0)	5.4 (5.1 - 5.8)	3.1 (2.8 - 3.3)
	K. pneumoniae	5.7 (4.3-7.6)	18.0 (14.0-22.6)	3.7 (2.9 - 4.8)	12.0 (9.6 - 14.8)	3.7 (2.9 - 4.5)	11.8 (9.5 - 14.2)
	L. monocytogenes	1.9 (1.3-2.8)	4.7 (3.1-6.9)	2.0 (1.5 - 2.7)	4.8 (3.7 - 6.2)	1.9 (1.6 - 2.4)	4.9 (4.0 - 5.9)
	N. meningitidis	8.8 (7.5-10.4)	7.6 (6.3-9.2)	18.9 (17.7 - 20.1)	15.0 (13.8 - 16.2)	20.1 (19.1 - 21.0)	17.1 (16.0 - 18.1)
	Other	2.4 (1.5-3.6)	3.1 (1.9-4.7)	8.9 (6.3 - 12.3)	9.7 (6.8 - 13.3)	9.0 (7.4 - 11.0)	11.0 (9.2 - 13.0)
	S. aureus	4.4 (3.3-5.8)	5.8 (4.3-7.6)	5.2 (4.5 - 6.0)	6.6 (5.7 - 7.5)	5.2 (4.7 - 5.9)	7.0 (6.2 - 7.9)
	S. pneumoniae	3.0 (2.4-3.7)	3.8 (3.0-4.7)	13.8 (12.8 - 14.8)	15.3 (14.2 - 16.7)	13.9 (13.1 - 14.7)	17.2 (16.1 - 18.3)
	Virus	37.6 (34.3-40.9)	15.8 (13.9-17.9)	24.6 (23.4 - 25.9)	10.1 (9.5 - 10.8)	29.5 (28.5 - 30.6)	12.5 (11.9 - 13.1)
**Western Sub-Saharan Africa**	*E. coli*	5.9 (4.4-7.6)	11.4 (8.7-14.6)	5.4 (4.4 - 6.6)	9.9 (8.2 - 12.0)	5.0 (4.1 - 6.0)	9.3 (7.7 - 11.1)
	Group B *Streptococcus*	19.9 (16.6-23.3)	22.4 (18.8-26.1)	9.0 (8.1 - 10.0)	9.8 (8.7 - 10.9)	7.8 (7.1 - 8.7)	8.5 (7.6 - 9.5)
	H. influenzae	10.0 (8.3-12.2)	6.9 (5.6-8.6)	11.6 (10.7 - 12.5)	7.3 (6.7 - 8.0)	9.3 (8.7 - 10.0)	5.8 (5.3 - 6.3)
	K. pneumoniae	5.3 (4.0-6.9)	16.7 (13.0-20.9)	3.9 (3.0 - 5.0)	11.9 (9.4 - 15.0)	3.9 (3.1 - 4.9)	12.1 (9.8 - 14.7)
	L. monocytogenes	1.9 (1.2-2.8)	4.7 (3.1-7.0)	2.1 (1.5 - 2.8)	4.9 (3.7 - 6.4)	2.1 (1.6 - 2.6)	5.0 (3.9 - 6.2)
	N. meningitidis	9.8 (7.7-12.3)	8.7 (6.8-11.1)	14.5 (13.0 - 16.1)	11.4 (10.1 - 12.9)	15.0 (13.6 - 16.5)	11.9 (10.6 - 13.3)
	Other	2.5 (1.6-3.8)	3.2 (2.1-4.7)	9.3 (6.6 - 12.9)	10.3 (7.3 - 14.2)	9.4 (7.4 - 12.1)	10.8 (8.5 - 13.8)
	S. aureus	4.3 (3.2-5.7)	5.7 (4.2-7.7)	5.2 (4.5 - 6.0)	6.3 (5.5 - 7.4)	5.2 (4.6 - 5.9)	6.6 (5.8 - 7.6)
	S. pneumoniae	3.0 (2.4-3.8)	4.6 (3.6-5.8)	13.4 (12.4 - 14.4)	18.1 (16.6 - 19.7)	13.6 (12.7 - 14.4)	18.7 (17.3 - 20.1)
	Virus	37.3 (34.0-40.9)	15.6 (13.7-17.7)	25.7 (24.3 - 27.1)	9.9 (9.2 - 10.6)	28.7 (27.4 - 29.9)	11.2 (10.6 - 11.8)
Benin	*E. coli*	6.0 (4.5-7.8)	11.5 (8.6-14.7)	5.5 (4.5 - 6.7)	10.2 (8.4 - 12.4)	5.1 (4.1 - 6.1)	9.5 (7.8 - 11.3)
	Group B *Streptococcus*	21.0 (17.5-24.7)	23.6 (19.8-27.6)	9.6 (8.6 - 10.6)	10.5 (9.4 - 11.7)	8.3 (7.5 - 9.2)	8.9 (8.0 - 9.9)
	H. influenzae	9.5 (7.5-11.7)	6.4 (4.9-8.2)	8.4 (7.7 - 9.1)	5.3 (4.9 - 5.9)	6.8 (6.3 - 7.3)	4.2 (3.8 - 4.5)
	K. pneumoniae	5.7 (4.3-7.5)	18.0 (14.0-22.6)	3.8 (3.0 - 4.9)	12.0 (9.4 - 15.0)	3.8 (3.0 - 4.8)	12.0 (9.7 - 14.6)
	L. monocytogenes	1.9 (1.2-2.8)	4.7 (3.1-7.0)	2.1 (1.6 - 2.8)	5.0 (3.8 - 6.7)	2.1 (1.6 - 2.6)	5.1 (4.0 - 6.4)
	N. meningitidis	8.4 (6.7-10.2)	7.2 (5.6-9.0)	15.3 (14.0 - 16.6)	12.5 (11.2 - 13.8)	15.7 (14.4 - 17.0)	13.0 (11.9 - 14.2)
	Other	2.5 (1.6-3.8)	3.3 (2.1-4.8)	9.2 (6.5 - 12.8)	10.5 (7.5 - 14.5)	9.3 (7.3 - 12.0)	11.0 (8.8 - 13.9)
	S. aureus	4.4 (3.2-5.8)	5.8 (4.3-7.8)	5.5 (4.7 - 6.3)	6.9 (5.9 - 8.0)	5.5 (4.8 - 6.2)	7.1 (6.2 - 8.1)

Location	Aetiology	Neonatal		Under 5		All Ages	
		Nonfatal	Fatal	Nonfatal	Fatal	Nonfatal	Fatal
	S. pneumoniae	3.0 (2.3-3.7)	3.6 (2.8-4.5)	15.0 (13.9 - 16.2)	17.0 (15.4 - 18.5)	15.2 (14.2 - 16.2)	17.7 (16.4 - 19.1)
	Virus	37.7 (34.0-41.3)	15.8 (13.9-18.0)	25.5 (24.2 - 26.9)	10.0 (9.3 - 10.7)	28.3 (27.1 - 29.5)	11.4 (10.8 - 12.0)
Burkina Faso	*E. coli*	5.8 (4.1-8.0)	11.1 (8.0-15.0)	5.8 (4.6 - 7.2)	10.6 (8.6 - 13.0)	5.3 (4.2 - 6.5)	9.8 (8.0 - 11.9)
	Group B *Streptococcus*	21.8 (17.0-27.0)	24.5 (19.0-30.5)	10.3 (8.9 - 11.6)	10.9 (9.4 - 12.5)	8.7 (7.6 - 9.9)	9.4 (8.2 - 10.7)
	H. influenzae	9.5 (7.0-12.4)	6.5 (4.7-8.8)	7.5 (6.7 - 8.3)	4.7 (4.1 - 5.3)	5.9 (5.3 - 6.6)	3.7 (3.3 - 4.2)
	K. pneumoniae	5.6 (4.0-7.7)	17.7 (13.1-23.3)	4.0 (3.1 - 5.2)	12.3 (9.5 - 15.6)	4.0 (3.1 - 5.1)	12.3 (9.8 - 15.4)
	L. monocytogenes	1.9 (1.2-3.0)	4.8 (3.0-7.4)	2.2 (1.6 - 3.0)	5.2 (3.8 - 7.0)	2.2 (1.6 - 2.8)	5.2 (4.0 - 6.7)
	N. meningitidis	7.7 (5.2-10.8)	6.7 (4.5-9.5)	11.2 (9.5 - 13.3)	9.1 (7.6 - 10.9)	11.6 (9.8 - 13.6)	9.4 (7.9 - 11.2)
	Other	2.7 (1.7-4.1)	3.5 (2.3-5.2)	9.6 (6.7 - 13.5)	10.9 (7.6 - 15.1)	9.7 (7.5 - 12.6)	11.3 (8.8 - 14.6)
	S. aureus	4.4 (3.0-6.2)	5.8 (4.0-8.2)	5.8 (5.0 - 6.9)	7.2 (6.1 - 8.6)	5.8 (5.0 - 6.8)	7.4 (6.3 - 8.8)
	S. pneumoniae	2.9 (2.0-3.8)	3.5 (2.4-4.7)	16.9 (15.2 - 18.5)	18.8 (16.7 - 20.9)	17.1 (15.7 - 18.7)	19.7 (17.8 - 21.6)
	Virus	37.6 (33.4-41.9)	15.9 (13.6-18.4)	26.7 (25.1 - 28.4)	10.3 (9.5 - 11.1)	29.7 (28.1 - 31.1)	11.6 (10.8 - 12.4)
Cabo Verde	*E. coli*	6.8 (5.7-8.2)	13.1 (10.9-15.5)	5.7 (4.8 - 6.6)	10.6 (9.3 - 12.0)	5.0 (4.4 - 5.7)	9.7 (8.5 - 10.9)
	Group B *Streptococcus*	24.8 (22.8-27.0)	27.6 (25.3-29.9)	9.1 (8.5 - 9.8)	12.3 (11.6 - 13.1)	7.0 (6.6 - 7.4)	6.8 (6.4 - 7.2)
	H. influenzae	7.9 (6.7-9.4)	5.0 (4.1-6.1)	7.2 (6.7 - 7.7)	4.2 (3.9 - 4.6)	5.3 (5.0 - 5.6)	2.7 (2.5 - 2.8)
	K. pneumoniae	5.3 (4.2-6.7)	17.3 (14.0-21.3)	3.8 (3.1 - 4.7)	12.6 (10.6 - 14.8)	3.9 (3.4 - 4.5)	12.3 (10.7 - 13.7)
	L. monocytogenes	1.8 (1.3-2.5)	4.6 (3.3-6.3)	2.2 (1.6 - 2.8)	5.0 (4.0 - 6.3)	2.2 (1.9 - 2.6)	6.0 (5.3 - 6.8)
	N. meningitidis	8.4 (7.5-9.5)	7.1 (6.2-8.2)	17.7 (16.7 - 18.5)	12.6 (11.8 - 13.4)	17.4 (16.8 - 18.0)	12.1 (11.6 - 12.6)
	Other	2.1 (1.2-3.2)	2.6 (1.6-4.0)	9.5 (6.7 - 13.2)	9.2 (6.6 - 12.5)	9.8 (8.0 - 11.8)	11.3 (9.0 - 13.8)
	S. aureus	4.0 (3.3-5.0)	5.3 (4.2-6.5)	4.7 (4.2 - 5.2)	5.5 (5.0 - 6.1)	4.8 (4.5 - 5.2)	6.5 (6.0 - 7.0)
	S. pneumoniae	2.8 (2.4-3.3)	4.6 (3.9-5.4)	14.8 (13.9 - 15.6)	19.2 (17.9 - 20.4)	13.6 (13.1 - 14.1)	21.4 (20.4 - 22.4)
	Virus	36.0 (33.2-38.7)	12.6 (11.3-14.1)	25.3 (24.2 - 26.5)	8.7 (8.2 - 9.2)	30.9 (30.0 - 31.7)	11.3 (10.8 - 11.8)
Cameroon	*E. coli*	5.9 (4.5-7.6)	11.5 (8.8-14.5)	5.6 (4.5 - 6.7)	10.5 (8.7 - 12.5)	5.0 (4.1 - 5.9)	9.3 (7.7 - 10.9)
	Group B *Streptococcus*	20.0 (17.0-23.2)	22.8 (19.4-26.1)	9.4 (8.4 - 10.3)	10.7 (9.6 - 11.8)	7.8 (7.0 - 8.5)	8.2 (7.4 - 9.0)
	H. influenzae	9.8 (8.1-11.8)	6.7 (5.4-8.2)	10.0 (9.2 - 10.7)	6.3 (5.8 - 6.9)	7.6 (7.0 - 8.1)	4.3 (4.0 - 4.6)
	K. pneumoniae	5.6 (4.3-7.2)	17.9 (14.2-22.2)	3.8 (3.0 - 4.9)	12.4 (9.9 - 15.3)	3.9 (3.1 - 4.8)	12.5 (10.3 - 14.9)
	L. monocytogenes	1.9 (1.3-2.8)	4.8 (3.2-7.0)	2.1 (1.6 - 2.8)	5.1 (3.9 - 6.7)	2.1 (1.7 - 2.6)	5.2 (4.3 - 6.4)
	N. meningitidis	9.1 (7.5-11.0)	8.0 (6.4-9.7)	14.9 (13.6 - 16.1)	12.1 (10.9 - 13.4)	15.4 (14.3 - 16.5)	13.0 (11.9 - 14.1)
	Other	2.5 (1.6-3.7)	3.2 (2.1-4.8)	9.3 (6.6 - 12.9)	10.5 (7.5 - 14.5)	9.5 (7.6 - 11.9)	11.5 (9.6 - 13.9)
	S. aureus	4.5 (3.3-5.8)	5.9 (4.4-7.8)	5.4 (4.7 - 6.1)	6.8 (5.9 - 7.8)	5.5 (4.8 - 6.1)	7.2 (6.4 - 8.1)

Location	Aetiology	Neonatal		Under 5		All Ages	
		Nonfatal	Fatal	Nonfatal	Fatal	Nonfatal	Fatal
	S. pneumoniae	3.0 (2.4-3.8)	3.7 (3.0-4.7)	13.8 (12.8 - 14.9)	15.4 (14.1 - 16.7)	13.9 (13.1 - 14.8)	16.6 (15.5 - 17.8)
	Virus	37.6 (34.4-41.1)	15.5 (13.7-17.6)	25.8 (24.4 - 27.1)	10.1 (9.4 - 10.7)	29.5 (28.4 - 30.7)	12.2 (11.5 - 12.8)
Chad	*E. coli*	5.8 (4.0-7.9)	11.0 (7.8-14.8)	5.1 (4.0 - 6.4)	8.9 (7.1 - 11.0)	4.8 (3.8 - 6.0)	8.4 (6.7 - 10.3)
	Group B *Streptococcus*	19.3 (15.2-23.8)	21.7 (17.3-26.5)	8.6 (7.5 - 9.8)	9.0 (7.9 - 10.2)	7.7 (6.7 - 8.7)	7.8 (6.8 - 8.9)
	H. influenzae	10.6 (8.4-13.4)	7.3 (5.6-9.6)	12.4 (11.2 - 13.6)	7.5 (6.7 - 8.3)	10.3 (9.3 - 11.4)	6.1 (5.4 - 6.7)
	K. pneumoniae	4.9 (3.5-6.7)	15.3 (11.2-19.9)	3.9 (2.9 - 5.1)	11.2 (8.5 - 14.4)	4.0 (3.0 - 5.1)	11.4 (9.0 - 14.4)
	L. monocytogenes	1.9 (1.2-2.9)	4.6 (2.8-7.0)	2.0 (1.4 - 2.7)	4.4 (3.1 - 5.9)	2.0 (1.4 - 2.6)	4.5 (3.4 - 5.8)
	N. meningitidis	10.7 (7.6-14.8)	9.3 (6.4-13.0)	14.9 (12.7 - 17.3)	11.4 (9.7 - 13.3)	14.9 (12.8 - 17.3)	11.6 (9.8 - 13.6)
	Other	2.7 (1.7-4.0)	3.4 (2.1-5.0)	8.9 (6.3 - 12.5)	9.5 (6.6 - 13.3)	9.2 (7.0 - 12.1)	10.1 (7.8 - 13.2)
	S. aureus	4.2 (2.8-5.9)	5.5 (3.7-7.8)	4.8 (4.1 - 5.6)	5.7 (4.8 - 6.7)	4.9 (4.3 - 5.8)	6.0 (5.1 - 7.0)
	S. pneumoniae	3.0 (2.2-4.0)	5.6 (4.0-7.3)	13.9 (12.8 - 15.2)	22.6 (20.6 - 24.7)	13.9 (12.7 - 15.1)	22.9 (20.9 - 24.9)
	Virus	37.0 (33.1-41.2)	16.3 (14.1-18.8)	25.4 (23.9 - 27.0)	9.9 (9.2 - 10.7)	28.3 (26.7 - 29.9)	11.2 (10.5 - 12.0)
Côte d'Ivoire	*E. coli*	6.1 (4.6-7.8)	11.7 (9.1-14.7)	5.4 (4.4 - 6.6)	10.0 (8.3 - 11.9)	4.8 (4.0 - 5.8)	8.7 (7.2 - 10.4)
	Group B *Streptococcus*	20.4 (17.6-23.5)	23.0 (19.8-26.2)	9.0 (8.1 - 9.9)	10.9 (9.9 - 11.9)	7.5 (6.8 - 8.2)	7.9 (7.1 - 8.6)
	H. influenzae	9.6 (8.1-11.4)	6.6 (5.4-8.0)	9.6 (8.9 - 10.3)	6.0 (5.6 - 6.6)	7.4 (6.9 - 7.9)	4.0 (3.7 - 4.3)
	K. pneumoniae	5.5 (4.2-7.1)	17.2 (13.5-21.5)	3.8 (2.9 - 4.9)	12.1 (9.7 - 14.9)	3.8 (3.0 - 4.7)	11.9 (9.8 - 14.3)
	L. monocytogenes	1.9 (1.3-2.7)	4.7 (3.1-6.7)	2.0 (1.5 - 2.7)	4.8 (3.7 - 6.2)	2.0 (1.6 - 2.5)	4.9 (4.0 - 5.9)
	N. meningitidis	9.4 (7.9-11.0)	8.1 (6.6-9.7)	17.5 (16.2 - 18.7)	13.5 (12.4 - 14.7)	18.1 (17.0 - 19.1)	14.8 (13.8 - 15.8)
	Other	2.4 (1.5-3.6)	3.1 (2.0-4.6)	9.0 (6.4 - 12.5)	9.6 (6.9 - 13.1)	9.2 (7.3 - 11.6)	10.7 (8.9 - 12.9)
	S. aureus	4.3 (3.2-5.7)	5.7 (4.2-7.5)	5.2 (4.5 - 5.9)	6.3 (5.5 - 7.2)	5.2 (4.6 - 5.8)	6.7 (5.9 - 7.5)
	S. pneumoniae	3.0 (2.4-3.7)	4.3 (3.5-5.3)	13.4 (12.5 - 14.4)	16.5 (15.2 - 17.8)	13.5 (12.8 - 14.3)	18.3 (17.2 - 19.5)
	Virus	37.4 (34.2-40.7)	15.7 (13.9-17.8)	25.2 (23.9 - 26.5)	10.3 (9.6 - 10.9)	28.5 (27.4 - 29.6)	12.1 (11.5 - 12.7)
Gambia	*E. coli*	5.7 (4.0-7.9)	11.0 (7.8-14.9)	5.8 (4.6 - 7.2)	10.9 (9.0 - 13.3)	5.1 (4.1 - 6.3)	9.5 (7.7 - 11.4)
	Group B *Streptococcus*	21.5 (16.7-27.0)	24.4 (18.8-30.3)	10.3 (9.0 - 11.7)	12.3 (10.7 - 13.9)	8.3 (7.2 - 9.3)	8.3 (7.2 - 9.4)
	H. influenzae	9.5 (6.9-12.7)	6.5 (4.6-8.9)	7.4 (6.6 - 8.2)	4.9 (4.3 - 5.5)	5.1 (4.6 - 5.7)	2.6 (2.3 - 2.9)
	K. pneumoniae	5.8 (4.1-8.0)	18.4 (13.7-24.3)	4.0 (3.0 - 5.2)	13.0 (10.2 - 16.1)	4.0 (3.1 - 5.0)	12.7 (10.2 - 15.5)
	L. monocytogenes	1.9 (1.2-3.0)	4.8 (3.0-7.6)	2.2 (1.6 - 3.0)	5.4 (4.0 - 7.2)	2.2 (1.7 - 2.8)	5.6 (4.5 - 6.9)
	N. meningitidis	7.4 (5.0-10.5)	6.5 (4.2-9.2)	10.5 (8.8 - 12.4)	8.6 (7.1 - 10.2)	10.8 (9.1 - 12.7)	9.0 (7.5 - 10.7)
	Other	2.8 (1.8-4.2)	3.6 (2.3-5.3)	9.7 (6.8 - 13.6)	10.7 (7.6 - 14.7)	9.8 (7.7 - 12.4)	11.9 (9.7 - 14.6)
	S. aureus	4.6 (3.1-6.4)	6.0 (4.1-8.5)	6.0 (5.1 - 7.1)	7.5 (6.3 - 8.9)	6.0 (5.2 - 7.0)	8.1 (7.0 - 9.4)

Location	Aetiology	Neonatal		Under 5		All Ages	
		Nonfatal	Fatal	Nonfatal	Fatal	Nonfatal	Fatal
	S. pneumoniae	2.9 (2.0-3.9)	3.0 (2.0-4.1)	17.6 (15.9 - 19.4)	16.0 (14.2 - 17.8)	18.1 (16.5 - 19.8)	18.9 (17.2 - 20.8)
	Virus	37.8 (33.6-42.3)	15.8 (13.5-18.3)	26.5 (24.8 - 28.2)	10.7 (10.0 - 11.6)	30.6 (29.0 - 32.1)	13.3 (12.4 - 14.1)
Ghana	*E. coli*	6.3 (4.8-8.0)	12.0 (9.4-15.1)	5.7 (4.7 - 6.9)	10.8 (9.2 - 12.8)	5.0 (4.2 - 5.9)	9.1 (7.6 - 10.6)
	Group B *Streptococcus*	22.7 (19.5-26.2)	25.4 (21.9-29.0)	10.0 (9.0 - 10.9)	12.0 (10.9 - 13.1)	7.7 (7.1 - 8.4)	7.8 (7.1 - 8.5)
	H. influenzae	8.7 (6.8-10.8)	5.8 (4.5-7.3)	7.3 (6.7 - 7.9)	4.7 (4.3 - 5.1)	5.0 (4.7 - 5.4)	2.6 (2.4 - 2.8)
	K. pneumoniae	5.8 (4.5-7.5)	18.5 (14.5-23.0)	3.8 (3.0 - 4.9)	12.7 (10.3 - 15.4)	3.9 (3.1 - 4.7)	12.3 (10.3 - 14.5)
	L. monocytogenes	1.9 (1.3-2.8)	4.7 (3.2-6.9)	2.2 (1.6 - 2.9)	5.2 (4.1 - 6.8)	2.1 (1.7 - 2.6)	5.4 (4.5 - 6.4)
	N. meningitidis	7.7 (6.4-9.2)	6.6 (5.3-8.0)	15.3 (14.1 - 16.4)	12.1 (11.0 - 13.2)	16.0 (15.1 - 16.9)	13.3 (12.5 - 14.2)
	Other	2.4 (1.5-3.6)	3.1 (1.9-4.6)	9.3 (6.6 - 13.0)	10.2 (7.3 - 14.0)	9.5 (7.6 - 11.8)	11.5 (9.6 - 13.5)
	S. aureus	4.4 (3.3-5.7)	5.7 (4.3-7.5)	5.5 (4.8 - 6.3)	6.8 (5.9 - 7.8)	5.5 (4.9 - 6.1)	7.3 (6.5 - 8.2)
	S. pneumoniae	2.9 (2.3-3.5)	3.4 (2.6-4.2)	15.1 (14.0 - 16.3)	15.5 (14.2 - 16.9)	15.5 (14.7 - 16.4)	18.3 (17.3 - 19.5)
	Virus	37.3 (33.8-40.7)	14.8 (13.0-16.7)	25.7 (24.4 - 27.1)	9.9 (9.3 - 10.6)	29.8 (28.8 - 30.9)	12.3 (11.7 - 12.9)
Guinea	*E. coli*	6.2 (4.7-8.0)	11.7 (9.0-15.0)	4.9 (3.9 - 6.0)	8.6 (7.0 - 10.6)	4.5 (3.7 - 5.6)	8.2 (6.7 - 9.9)
	Group B *Streptococcus*	20.2 (17.6-23.4)	22.6 (19.6-25.9)	8.3 (7.5 - 9.1)	8.9 (8.0 - 9.8)	7.0 (6.4 - 7.7)	7.5 (6.8 - 8.2)
	H. influenzae	9.7 (8.3-11.4)	6.7 (5.5-8.0)	10.6 (9.8 - 11.3)	6.5 (5.9 - 7.0)	8.2 (7.6 - 8.8)	5.0 (4.6 - 5.4)
	K. pneumoniae	5.1 (3.8-6.8)	15.8 (12.1-20.1)	3.6 (2.8 - 4.7)	10.7 (8.4 - 13.6)	3.7 (2.9 - 4.7)	10.9 (8.8 - 13.3)
	L. monocytogenes	1.9 (1.2-2.7)	4.5 (3.0-6.4)	1.9 (1.4 - 2.5)	4.2 (3.1 - 5.6)	1.9 (1.5 - 2.4)	4.3 (3.4 - 5.4)
	N. meningitidis	10.3 (8.8-12.3)	8.9 (7.4-10.7)	21.1 (19.7 - 22.3)	16.3 (15.0 - 17.4)	21.2 (20.0 - 22.4)	16.5 (15.3 - 17.5)
	Other	2.4 (1.5-3.6)	3.0 (1.9-4.5)	8.4 (5.9 - 11.8)	9.0 (6.4 - 12.6)	8.7 (6.8 - 11.2)	9.7 (7.7 - 12.3)
	S. aureus	4.1 (3.1-5.5)	5.4 (3.9-7.3)	4.5 (3.9 - 5.1)	5.4 (4.7 - 6.1)	4.7 (4.1 - 5.3)	5.7 (5.1 - 6.5)
	S. pneumoniae	3.0 (2.4-3.7)	5.5 (4.4-6.7)	13.0 (12.2 - 13.9)	21.1 (19.6 - 22.7)	12.9 (12.2 - 13.6)	21.5 (20.2 - 22.9)
	Virus	36.9 (33.7-40.3)	15.9 (14.0-18.0)	23.8 (22.6 - 25.0)	9.3 (8.8 - 9.9)	27.1 (26.1 - 28.2)	10.7 (10.2 - 11.3)
Guinea-Bissau	*E. coli*	6.3 (4.7-8.2)	11.8 (9.0-15.1)	5.1 (4.1 - 6.4)	9.5 (7.8 - 11.6)	4.5 (3.6 - 5.5)	8.0 (6.4 - 9.6)
	Group B *Streptococcus*	20.7 (17.7-23.9)	23.1 (19.7-26.6)	8.6 (7.7 - 9.5)	10.6 (9.5 - 11.7)	6.9 (6.2 - 7.6)	7.2 (6.4 - 7.8)
	H. influenzae	9.4 (7.7-11.3)	6.4 (5.1-7.9)	8.4 (7.8 - 9.1)	5.4 (5.0 - 5.9)	6.1 (5.6 - 6.5)	3.2 (3.0 - 3.5)
	K. pneumoniae	5.5 (4.1-7.3)	17.0 (13.0-21.7)	3.6 (2.8 - 4.7)	11.5 (9.1 - 14.3)	3.6 (2.8 - 4.5)	11.2 (8.9 - 13.5)
	L. monocytogenes	1.9 (1.2-2.8)	4.6 (3.0-6.7)	1.9 (1.4 - 2.5)	4.5 (3.4 - 5.8)	1.9 (1.5 - 2.4)	4.5 (3.6 - 5.5)
	N. meningitidis	9.1 (7.7-10.9)	7.8 (6.5-9.5)	21.3 (19.9 - 22.6)	16.1 (14.9 - 17.4)	22.3 (21.2 - 23.4)	18.1 (17.0 - 19.2)
	Other	2.4 (1.5-3.6)	3.1 (1.9-4.6)	8.6 (6.0 - 12.0)	9.0 (6.4 - 12.4)	8.7 (6.9 - 10.9)	10.1 (8.4 - 12.2)
	S. aureus	4.3 (3.2-5.7)	5.6 (4.1-7.5)	5.0 (4.3 - 5.8)	6.1 (5.2 - 7.0)	5.0 (4.4 - 5.7)	6.5 (5.7 - 7.3)

Location	Aetiology	Neonatal		Under 5		All Ages	
		Nonfatal	Fatal	Nonfatal	Fatal	Nonfatal	Fatal
	S. pneumoniae	3.0 (2.4-3.7)	4.4 (3.5-5.5)	13.5 (12.6 - 14.5)	17.1 (15.8 - 18.4)	13.6 (12.8 - 14.4)	19.3 (18.1 - 20.5)
	Virus	37.3 (34.0-40.8)	16.2 (14.3-18.4)	23.9 (22.7 - 25.2)	10.2 (9.6 - 10.9)	27.5 (26.5 - 28.6)	12.0 (11.4 - 12.7)
Liberia	*E. coli*	6.3 (4.8-7.9)	11.9 (9.4-14.9)	5.4 (4.4 - 6.6)	10.1 (8.4 - 12.0)	4.7 (3.9 - 5.7)	8.5 (7.0 - 10.1)
	Group B *Streptococcus*	20.8 (18.1-23.7)	23.3 (20.3-26.5)	9.0 (8.1 - 9.8)	10.8 (9.7 - 11.7)	7.2 (6.6 - 7.8)	7.3 (6.6 - 7.9)
	H. influenzae	9.3 (7.8-11.1)	6.3 (5.2-7.7)	8.6 (8.0 - 9.3)	5.5 (5.0 - 6.0)	6.3 (5.9 - 6.8)	3.3 (3.1 - 3.6)
	K. pneumoniae	5.6 (4.2-7.2)	17.5 (13.7-22.0)	3.7 (2.9 - 4.8)	12.0 (9.7 - 14.7)	3.7 (3.0 - 4.6)	11.7 (9.5 - 13.9)
	L. monocytogenes	1.9 (1.3-2.7)	4.7 (3.1-6.7)	2.0 (1.5 - 2.7)	4.8 (3.7 - 6.2)	2.0 (1.6 - 2.5)	4.9 (4.0 - 5.9)
	N. meningitidis	9.2 (7.9-10.7)	7.9 (6.7-9.4)	18.9 (17.7 - 20.2)	14.8 (13.6 - 16.0)	19.9 (19.0 - 20.9)	16.6 (15.6 - 17.5)
	Other	2.4 (1.4-3.5)	3.0 (1.9-4.5)	9.0 (6.3 - 12.4)	9.6 (6.8 - 13.3)	9.1 (7.3 - 11.3)	10.8 (9.0 - 12.8)
	S. aureus	4.3 (3.3-5.7)	5.7 (4.3-7.4)	5.2 (4.5 - 5.9)	6.4 (5.5 - 7.3)	5.2 (4.6 - 5.8)	6.8 (6.0 - 7.5)
	S. pneumoniae	3.0 (2.4-3.6)	4.2 (3.4-5.1)	13.2 (12.3 - 14.3)	16.0 (14.8 - 17.4)	13.3 (12.6 - 14.1)	18.0 (17.0 - 19.1)
	Virus	37.3 (34.2-40.6)	15.5 (13.7-17.5)	24.9 (23.6 - 26.2)	10.0 (9.4 - 10.7)	28.6 (27.6 - 29.6)	12.1 (11.5 - 12.7)
Mali	*E. coli*	5.7 (4.0-7.8)	11.0 (8.0-14.7)	5.6 (4.5 - 6.9)	10.3 (8.3 - 12.5)	5.2 (4.2 - 6.4)	9.6 (7.8 - 11.6)
	Group B *Streptococcus*	20.2 (15.9-25.1)	23.0 (18.0-28.4)	9.7 (8.5 - 11.0)	10.5 (9.1 - 11.9)	8.5 (7.5 - 9.6)	9.0 (7.9 - 10.3)
	H. influenzae	10.1 (7.7-13.0)	7.0 (5.2-9.2)	9.7 (8.8 - 10.7)	6.1 (5.5 - 6.8)	8.0 (7.3 - 8.9)	4.9 (4.4 - 5.4)
	K. pneumoniae	5.4 (3.9-7.5)	17.2 (12.8-22.6)	4.0 (3.0 - 5.2)	12.2 (9.5 - 15.5)	4.0 (3.1 - 5.1)	12.4 (9.9 - 15.5)
	L. monocytogenes	1.9 (1.2-2.9)	4.8 (3.0-7.3)	2.2 (1.6 - 2.9)	5.1 (3.7 - 6.8)	2.1 (1.6 - 2.8)	5.2 (3.9 - 6.6)
	N. meningitidis	8.8 (6.1-12.1)	7.7 (5.2-10.8)	11.7 (9.9 - 13.8)	9.5 (7.9 - 11.3)	12.0 (10.1 - 14.0)	9.8 (8.2 - 11.6)
	Other	2.7 (1.7-4.0)	3.5 (2.3-5.1)	9.5 (6.7 - 13.4)	10.8 (7.5 - 14.9)	9.6 (7.4 - 12.7)	11.2 (8.8 - 14.3)
	S. aureus	4.4 (3.0-6.2)	5.9 (4.0-8.2)	5.6 (4.8 - 6.6)	6.9 (5.9 - 8.2)	5.6 (4.9 - 6.6)	7.2 (6.2 - 8.4)
	S. pneumoniae	3.0 (2.1-3.9)	3.9 (2.8-5.1)	15.4 (13.9 - 16.8)	18.2 (16.3 - 20.1)	15.5 (14.1 - 16.8)	19.0 (17.2 - 20.8)
	Virus	37.6 (33.6-42.0)	16.1 (13.8-18.6)	26.6 (24.9 - 28.2)	10.3 (9.6 - 11.2)	29.3 (27.8 - 30.8)	11.7 (11.0 - 12.5)
Mauritania	*E. coli*	6.0 (4.6-7.6)	11.7 (9.1-14.6)	5.7 (4.7 - 6.8)	10.8 (9.2 - 12.5)	5.2 (4.3 - 6.1)	9.4 (8.0 - 10.9)
	Group B *Streptococcus*	20.5 (17.5-23.7)	23.3 (19.9-26.7)	9.4 (8.6 - 10.4)	13.1 (11.9 - 14.4)	7.8 (7.2 - 8.6)	7.9 (7.2 - 8.6)
	H. influenzae	9.7 (8.1-11.6)	6.5 (5.4-8.0)	11.2 (10.4 - 12.0)	6.8 (6.3 - 7.4)	8.5 (7.9 - 9.1)	3.9 (3.6 - 4.2)
	K. pneumoniae	5.4 (4.1-6.9)	17.4 (13.8-21.5)	3.9 (3.1 - 5.0)	13.5 (11.2 - 16.1)	4.0 (3.3 - 4.9)	12.8 (10.8 - 15.0)
	L. monocytogenes	1.9 (1.3-2.7)	4.8 (3.2-6.9)	2.2 (1.6 - 2.8)	5.1 (4.1 - 6.4)	2.2 (1.7 - 2.7)	5.6 (4.7 - 6.6)
	N. meningitidis	9.5 (7.6-11.6)	8.3 (6.5-10.3)	13.3 (12.0 - 14.7)	10.3 (9.3 - 11.4)	13.7 (12.4 - 15.0)	10.9 (9.8 - 12.0)
	Other	2.4 (1.5-3.6)	3.2 (2.0-4.6)	9.5 (6.7 - 13.2)	9.1 (6.6 - 12.3)	9.7 (7.8 - 12.2)	11.4 (9.4 - 13.5)
	S. aureus	4.4 (3.2-5.7)	5.8 (4.3-7.5)	5.3 (4.7 - 6.0)	6.4 (5.6 - 7.3)	5.4 (4.8 - 6.0)	7.1 (6.4 - 8.0)

Location	Aetiology	Neonatal		Under 5		All Ages	
		Nonfatal	Fatal	Nonfatal	Fatal	Nonfatal	Fatal
	S. pneumoniae	3.0 (2.4-3.7)	4.2 (3.3-5.1)	13.1 (12.2 - 14.0)	14.0 (12.9 - 15.1)	13.4 (12.6 - 14.2)	18.1 (17.0 - 19.3)
	Virus	37.3 (34.1-40.6)	14.9 (13.1-16.8)	26.4 (25.0 - 27.7)	10.8 (10.1 - 11.5)	30.2 (29.1 - 31.3)	12.8 (12.2 - 13.5)
Niger	*E. coli*	5.8 (4.1-7.8)	11.0 (7.9-14.9)	5.5 (4.4 - 6.8)	9.8 (7.8 - 12.0)	5.2 (4.1 - 6.4)	9.4 (7.5 - 11.5)
	Group B *Streptococcus*	20.1 (15.9-24.8)	22.8 (18.0-28.0)	9.3 (8.2 - 10.6)	9.6 (8.3 - 11.0)	8.4 (7.3 - 9.5)	8.9 (7.7 - 10.1)
	H. influenzae	10.2 (7.9-13.1)	7.0 (5.4-9.2)	10.5 (9.5 - 11.6)	6.5 (5.8 - 7.3)	9.0 (8.1 - 9.9)	5.7 (5.1 - 6.4)
	K. pneumoniae	5.3 (3.8-7.2)	16.5 (12.2-21.5)	4.0 (3.0 - 5.2)	11.7 (9.0 - 15.0)	4.0 (3.1 - 5.2)	11.8 (9.2 - 15.0)
	L. monocytogenes	1.9 (1.2-2.9)	4.7 (2.9-7.1)	2.1 (1.5 - 2.9)	4.9 (3.5 - 6.5)	2.1 (1.5 - 2.7)	4.9 (3.6 - 6.4)
	N. meningitidis	9.3 (6.4-12.9)	8.1 (5.5-11.4)	12.7 (10.8 - 15.0)	10.2 (8.5 - 12.1)	13.0 (11.0 - 15.2)	10.3 (8.6 - 12.2)
	Other	2.7 (1.7-4.0)	3.5 (2.3-5.1)	9.4 (6.5 - 13.2)	10.6 (7.4 - 14.7)	9.5 (7.2 - 12.7)	10.8 (8.1 - 14.4)
	S. aureus	4.4 (3.0-6.0)	5.7 (3.9-8.0)	5.4 (4.6 - 6.3)	6.5 (5.5 - 7.8)	5.4 (4.7 - 6.4)	6.7 (5.7 - 7.9)
	S. pneumoniae	3.0 (2.2-3.9)	4.4 (3.2-5.8)	14.9 (13.5 - 16.2)	20.2 (18.2 - 22.3)	15.0 (13.7 - 16.3)	20.6 (18.6 - 22.5)
	Virus	37.4 (33.5-41.8)	16.2 (14.0-18.7)	26.2 (24.6 - 27.8)	10.1 (9.3 - 10.9)	28.5 (26.9 - 30.0)	10.9 (10.2 - 11.7)
Nigeria	*E. coli*	5.9 (4.4-7.6)	11.4 (8.7-14.3)	5.4 (4.4 - 6.5)	9.9 (8.3 - 11.9)	5.1 (4.2 - 6.1)	9.5 (7.9 - 11.2)
	Group B *Streptococcus*	19.1 (16.3-22.1)	21.8 (18.6-25.1)	8.7 (7.8 - 9.6)	9.6 (8.6 - 10.7)	7.7 (7.0 - 8.5)	8.6 (7.7 - 9.5)
	H. influenzae	10.3 (8.7-12.3)	7.1 (5.8-8.6)	13.5 (12.5 - 14.5)	8.3 (7.6 - 9.1)	11.3 (10.5 - 12.1)	7.0 (6.4 - 7.6)
	K. pneumoniae	5.1 (3.9-6.7)	16.5 (12.8-20.7)	3.8 (3.0 - 5.0)	12.0 (9.6 - 15.1)	3.9 (3.1 - 4.9)	12.2 (10.0 - 14.8)
	L. monocytogenes	1.9 (1.3-2.7)	4.8 (3.1-6.9)	2.1 (1.5 - 2.7)	4.9 (3.7 - 6.4)	2.0 (1.6 - 2.6)	5.0 (3.9 - 6.3)
	N. meningitidis	10.7 (8.6-13.2)	9.4 (7.3-11.7)	14.3 (12.9 - 15.9)	11.6 (10.3 - 12.9)	14.7 (13.2 - 16.2)	11.8 (10.5 - 13.1)
	Other	2.4 (1.6-3.6)	3.2 (2.0-4.6)	9.3 (6.6 - 13.0)	10.4 (7.3 - 14.2)	9.5 (7.3 - 12.3)	10.8 (8.4 - 13.9)
	S. aureus	4.3 (3.2-5.7)	5.7 (4.2-7.5)	5.0 (4.4 - 5.8)	6.2 (5.4 - 7.1)	5.1 (4.5 - 5.8)	6.4 (5.7 - 7.3)
	S. pneumoniae	3.1 (2.4-3.8)	4.9 (3.8-6.1)	12.2 (11.3 - 13.0)	17.3 (15.9 - 18.7)	12.2 (11.4 - 13.0)	17.9 (16.6 - 19.2)
	Virus	37.1 (34.0-40.5)	15.4 (13.6-17.4)	25.7 (24.3 - 27.1)	9.8 (9.2 - 10.5)	28.5 (27.3 - 29.7)	10.9 (10.3 - 11.5)
Sao Tome and Principe	*E. coli*	6.2 (4.9-7.7)	12.1 (9.6-14.8)	5.7 (4.7 - 6.8)	11.0 (9.4 - 12.8)	5.0 (4.2 - 5.8)	9.4 (8.1 - 10.8)
	Group B *Streptococcus*	21.3 (18.6-24.2)	24.1 (21.0-27.2)	9.4 (8.5 - 10.2)	12.5 (11.4 - 13.5)	7.7 (7.0 - 8.3)	7.8 (7.2 - 8.4)
	H. influenzae	9.1 (7.4-10.8)	6.0 (4.9-7.4)	8.6 (7.9 - 9.3)	5.5 (5.0 - 6.0)	6.4 (6.0 - 6.9)	3.2 (3.0 - 3.4)
	K. pneumoniae	5.8 (4.5-7.4)	18.7 (15.0-23.2)	3.8 (3.0 - 4.8)	13.2 (10.9 - 15.7)	3.8 (3.1 - 4.6)	12.5 (10.5 - 14.5)
	L. monocytogenes	1.9 (1.3-2.8)	4.8 (3.3-6.9)	2.2 (1.6 - 2.8)	5.3 (4.1 - 6.7)	2.1 (1.7 - 2.6)	5.6 (4.8 - 6.6)
	N. meningitidis	8.6 (7.4-10.0)	7.4 (6.3-8.7)	16.3 (15.2 - 17.4)	12.6 (11.6 - 13.7)	17.1 (16.3 - 17.9)	13.9 (13.1 - 14.7)
	Other	2.3 (1.4-3.5)	3.0 (1.9-4.5)	9.5 (6.7 - 13.2)	9.8 (7.1 - 13.3)	9.5 (7.5 - 11.8)	11.5 (9.5 - 13.6)
	S. aureus	4.4 (3.4-5.6)	5.9 (4.5-7.4)	5.4 (4.7 - 6.1)	6.8 (5.9 - 7.6)	5.4 (4.8 - 5.9)	7.4 (6.6 - 8.1)

Location	Aetiology	Neonatal		Under 5		All Ages	
		Nonfatal	Fatal	Nonfatal	Fatal	Nonfatal	Fatal
	S. pneumoniae	3.0 (2.4-3.6)	3.5 (2.8-4.2)	13.7 (12.7 - 14.8)	13.2 (12.2 - 14.4)	13.7 (13.0 - 14.5)	16.3 (15.4 - 17.3)
	Virus	37.4 (34.3-40.6)	14.6 (12.9-16.5)	25.5 (24.1 - 26.8)	10.2 (9.5 - 10.8)	29.3 (28.3 - 30.3)	12.4 (11.8 - 12.9)
Senegal	*E. coli*	6.2 (4.6-8.0)	11.7 (9.0-15.0)	5.6 (4.6 - 6.9)	10.5 (8.8 - 12.5)	4.9 (4.0 - 6.0)	8.9 (7.4 - 10.6)
	Group B *Streptococcus*	22.2 (18.7-25.9)	24.8 (20.9-28.9)	9.7 (8.7 - 10.7)	12.5 (11.2 - 13.7)	7.8 (7.0 - 8.5)	8.0 (7.2 - 8.8)
	H. influenzae	9.0 (7.0-11.3)	6.1 (4.6-7.8)	7.3 (6.7 - 7.9)	4.8 (4.3 - 5.3)	5.2 (4.8 - 5.6)	2.8 (2.5 - 3.0)
	K. pneumoniae	5.7 (4.3-7.5)	17.9 (13.9-22.6)	3.8 (3.0 - 4.9)	12.7 (10.2 - 15.4)	3.9 (3.0 - 4.8)	12.1 (9.8 - 14.4)
	L. monocytogenes	1.9 (1.3-2.8)	4.7 (3.1-7.0)	2.1 (1.6 - 2.8)	5.0 (3.9 - 6.5)	2.1 (1.7 - 2.6)	5.2 (4.2 - 6.3)
	N. meningitidis	7.9 (6.3-9.7)	6.8 (5.3-8.4)	15.4 (14.0 - 16.7)	11.7 (10.5 - 12.8)	16.0 (14.8 - 17.2)	12.9 (11.9 - 14.0)
	Other	2.5 (1.6-3.8)	3.2 (2.0-4.7)	9.3 (6.6 - 13.0)	9.5 (6.8 - 13.0)	9.4 (7.5 - 11.8)	11.0 (9.1 - 13.1)
	S. aureus	4.4 (3.2-5.7)	5.7 (4.2-7.6)	5.5 (4.8 - 6.3)	6.7 (5.8 - 7.7)	5.5 (4.9 - 6.2)	7.2 (6.3 - 8.1)
	S. pneumoniae	2.9 (2.2-3.6)	3.6 (2.8-4.5)	15.6 (14.3 - 16.8)	16.0 (14.6 - 17.5)	15.8 (14.9 - 16.8)	19.4 (18.2 - 20.7)
	Virus	37.4 (33.7-41.1)	15.5 (13.5-17.6)	25.6 (24.2 - 27.0)	10.6 (9.9 - 11.3)	29.4 (28.2 - 30.5)	12.5 (11.9 - 13.2)
Sierra Leone	*E. coli*	6.1 (4.6-7.8)	11.7 (9.1-14.7)	5.4 (4.3 - 6.5)	10.0 (8.1 - 12.1)	4.8 (3.9 - 5.7)	9.1 (7.5 - 10.9)
	Group B *Streptococcus*	20.4 (17.4-23.7)	23.0 (19.5-26.6)	9.2 (8.3 - 10.1)	9.9 (8.8 - 10.9)	7.6 (6.8 - 8.3)	8.3 (7.4 - 9.1)
	H. influenzae	9.4 (7.7-11.5)	6.4 (5.1-8.0)	8.3 (7.7 - 9.0)	5.3 (4.9 - 5.8)	6.3 (5.8 - 6.7)	4.0 (3.7 - 4.4)
	K. pneumoniae	5.8 (4.3-7.7)	18.2 (14.2-23.0)	3.7 (2.9 - 4.8)	11.5 (9.1 - 14.5)	3.7 (2.9 - 4.6)	11.6 (9.3 - 14.2)
	L. monocytogenes	1.9 (1.3-2.8)	4.7 (3.1-6.9)	2.0 (1.5 - 2.7)	4.9 (3.7 - 6.5)	2.0 (1.6 - 2.5)	4.9 (4.0 - 6.2)
	N. meningitidis	8.7 (7.3-10.3)	7.5 (6.2-9.1)	18.5 (17.3 - 19.8)	15.6 (14.3 - 17.0)	19.3 (18.3 - 20.4)	16.3 (15.2 - 17.5)
	Other	2.4 (1.5-3.7)	3.1 (1.9-4.7)	8.8 (6.2 - 12.2)	10.4 (7.3 - 14.4)	9.0 (7.1 - 11.3)	10.9 (8.7 - 13.7)
	S. aureus	4.5 (3.3-5.8)	5.8 (4.4-7.7)	5.3 (4.6 - 6.1)	6.8 (5.8 - 7.8)	5.3 (4.7 - 6.0)	7.0 (6.1 - 7.9)
	S. pneumoniae	3.0 (2.4-3.8)	3.6 (2.8-4.5)	13.9 (12.9 - 15.1)	15.9 (14.6 - 17.3)	14.0 (13.2 - 14.9)	16.5 (15.3 - 17.7)
	Virus	37.7 (34.3-41.1)	15.9 (14.0-18.0)	24.7 (23.5 - 26.0)	9.7 (9.0 - 10.4)	28.1 (27.0 - 29.1)	11.3 (10.7 - 11.9)
Togo	*E. coli*	6.1 (4.7-7.8)	11.7 (9.1-14.8)	5.6 (4.6 - 6.8)	10.4 (8.6 - 12.4)	5.0 (4.1 - 5.9)	8.8 (7.3 - 10.4)
	Group B *Streptococcus*	21.3 (18.2-24.6)	23.9 (20.5-27.4)	9.4 (8.5 - 10.3)	11.1 (10.0 - 12.1)	7.7 (7.0 - 8.4)	7.6 (6.8 - 8.3)
	H. influenzae	9.3 (7.6-11.3)	6.3 (5.1-7.8)	8.6 (7.9 - 9.3)	5.4 (4.9 - 5.9)	6.4 (5.9 - 6.8)	3.2 (3.0 - 3.5)
	K. pneumoniae	5.6 (4.2-7.2)	17.6 (13.9-21.9)	3.8 (3.0 - 4.9)	12.3 (9.9 - 15.1)	3.9 (3.1 - 4.8)	12.1 (10.0 - 14.5)
	L. monocytogenes	1.9 (1.3-2.8)	4.7 (3.1-6.9)	2.1 (1.6 - 2.8)	5.0 (3.9 - 6.5)	2.1 (1.7 - 2.6)	5.1 (4.2 - 6.2)
	N. meningitidis	8.7 (7.2-10.4)	7.5 (6.0-9.2)	15.8 (14.4 - 17.0)	12.4 (11.2 - 13.6)	16.4 (15.3 - 17.5)	13.5 (12.5 - 14.6)
	Other	2.5 (1.6-3.6)	3.1 (2.0-4.6)	9.3 (6.7 - 13.0)	10.1 (7.1 - 13.7)	9.4 (7.5 - 11.8)	11.2 (9.4 - 13.4)
	S. aureus	4.4 (3.2-5.7)	5.7 (4.3-7.5)	5.4 (4.7 - 6.1)	6.6 (5.7 - 7.6)	5.4 (4.8 - 6.1)	7.1 (6.2 - 7.9)

Location	Aetiology	Neonatal		Under 5		All Ages	
		**Nonfatal**	**Fatal**	**Nonfatal**	**Fatal**	**Nonfatal**	**Fatal**
	S. pneumoniae	3.0 (2.4-3.6)	4.0 (3.2-4.9)	14.3 (13.2 - 15.4)	16.7 (15.4 - 18.1)	14.5 (13.7 - 15.4)	19.0 (17.8 - 20.2)
	Virus	37.4 (34.0-40.8)	15.4 (13.6-17.4)	25.7 (24.3 - 27.1)	10.1 (9.4 - 10.8)	29.4 (28.2 - 30.5)	12.3 (11.7 - 13.0)

Appendix Table S3: Global meningitis cases and deaths in 1990 and 2019 and percent change between 1990 and 2019 by aetiology, children under 5 years old and all ages

	Under 5						All Age					
	Deaths		Incidence		Percent Change 1990-2019		Deaths		Incidence		Percent Change 1990-2019	
**Aetiology**	**1990**	**2019**	**1990**	**2019**	**Deaths**	**Cases**	**1990**	**2019**	**1990**	**2019**	**Deaths**	**Cases**
**Total Meningitis**	**284,000 (237,000 to 339,000)**	**112,000 (87,400 to 145,000)**	**1,970,000 (1,460,000 to 2,650,000)**	**1,280,000 (947,000 to 1,710,000)**	**-60.5 (-69.2 to -49.8)**	**-35.3 (-36.5 to -34.0)**	**433,000 (376,000 to 494,000)**	**236,000 (204,000 to 277,000)**	**3,290,000 (2,700,000 to 4,000,000)**	**2,510,000 (2,110,000 to 2,990,000)**	**-45.4 (-53.5 to -35.8)**	**-23.8 (-26.5 to -20.4)**
E. coli	23,300 (17,900 to 30,400)	11,200 (8,160 to 15,000)	86,400 (59,500 to 122,000)	69,000 (47,900 to 95,900)	-51.8 (-62.8 to -38.3)	-19.9 (-26.4 to -12.9)	34,100 (27,100 to 42,900)	21,800 (17,600 to 27,300)	135,000 (102,000 to 178,000)	121,000 (94,500 to 153,000)	-35.9 (-46.5 to -23.6)	-10.2 (-17.2 to -3.1)
Group B *Streptococcus*	23,200 (19,100 to 28,600)	11,500 (8,850 to 14,700)	139,000 (102,000 to 185,000)	113,000 (83,300 to 152,000)	-50.3 (-61.0 to -36.8)	-19.0 (-23.8 to -14.1)	30,600 (25,800 to 36,300)	19,000 (15,700 to 22,900)	198,000 (156,000 to 247,000)	178,000 (146,000 to 220,000)	-37.6 (-48.1 to -25.9)	-9.8 (-14.5 to -4.6)
H. influenzae	32,200 (26,100 to 39,200)	7,550 (5,770 to 9,730)	354,000 (260,000 to 475,000)	132,000 (97,900 to 179,000)	-76.5 (-81.8 to -69.5)	-62.6 (-64.5 to -60.5)	38,000 (31,500 to 45,400)	11,100 (9,080 to 13,500)	435,000 (340,000 to 561,000)	187,000 (151,000 to 234,000)	-70.8 (-76.2 to -64.2)	-56.9 (-59.5 to -54.1)
K. pneumoniae	29,600 (21,900 to 39,300)	13,400 (9,650 to 18,100)	65,500 (43,700 to 96,200)	48,000 (32,900 to 67,900)	-54.3 (-64.9 to -41.8)	-26.6 (-32.9 to -19.4)	46,600 (36,100 to 59,300)	28,800 (23,000 to 35,900)	115,000 (84,500 to 154,000)	96,500 (74,600 to 124,000)	-37.9 (-48.3 to -26.0)	-15.9 (-22.4 to -9.0)
L. monocytogenes	11,400 (7,990 to 15,600)	5,480 (3,760 to 7,750)	33,200 (20,900 to 49,900)	26,400 (16,800 to 39,300)	-51.8 (-62.6 to -38.1)	-20.2 (-27.5 to -11.4)	18,200 (13,700 to 23,300)	12,200 (9,710 to 15,200)	56,900 (41,600 to 77,200)	52,100 (40,000 to 67,700)	-32.3 (-42.9 to -19.5)	-8.1 (-15.8 to 1.0)
N. meningitidis	52,300 (42,200 to 64,300)	14,400 (11,300 to 18,800)	442,000 (329,000 to 588,000)	216,000 (159,000 to 293,000)	-72.3 (-78.5 to -64.4)	-51.1 (-53.7 to -48.4)	80,900 (69,300 to 95,100)	32,100 (27,600 to 38,200)	744,000 (608,000 to 902,000)	433,000 (361,000 to 518,000)	-60.2 (-66.4 to -52.7)	-41.7 (-44.8 to -38.4)
Other	24,800 (16,300 to 35,100)	11,200 (7,540 to 16,800)	154,000 (93,900 to 235,000)	117,000 (72,300 to 177,000)	-54.5 (-65.1 to -40.5)	-23.5 (-31.0 to -15.0)	40,900 (30,900 to 53,300)	25,900 (20,200 to 33,400)	274,000 (202,000 to 362,000)	237,000 (180,000 to 306,000)	-36.5 (-47.7 to -23.8)	-13.0 (-20.9 to -4.0)
S. aureus	14,600 (11,700 to 18,200)	7,010 (5,180 to 9,250)	79,700 (58,500 to 108,000)	63,900 (46,500 to 86,600)	-51.7 (-62.7 to -37.9)	-19.8 (-25.9 to -13.6)	23,800 (19,900 to 28,400)	15,700 (12,900 to 18,900)	141,000 (114,000 to 174,000)	128,000 (104,000 to 154,000)	-33.8 (-43.4 to -22.2)	-9.6 (-15.2 to -3.9)
S. pneumoniae	46,300 (37,600 to 56,900)	19,400 (15,100 to 25,200)	187,000 (138,000 to 252,000)	169,000 (125,000 to 228,000)	-57.8 (-67.1 to -46.1)	-9.4 (-14.1 to -4.7)	73,500 (62,700 to 85,600)	42,700 (36,600 to 50,600)	318,000 (261,000 to 387,000)	326,000 (272,000 to 390,000)	-41.6 (-50.6 to -31.8)	2.6 (-2.1 to 7.5)

Virus	26,400 (21,700 to 31,900)	11,000 (8,550 to 14,100)	432,000 (320,000 to 584,000)	320,000 (237,000 to 431,000)	-58.2 (-67.3 to -47.1)	-25.9 (-28.2 to -23.3)	45,900 (40,100 to 52,500)	27,000 (23,300 to 31,400)	871,000 (723,000 to 1,040,000)	748,000 (622,000 to 880,000)	-41.0 (-49.4 to -31.0)	-14.0 (-17.2 to -10.3)

Appendix Table S4: Meningitis belt meningitis cases and deaths in 1990 and 2019 and percent change between 1990 and 2019 by aetiology, children under 5 years old and all ages

	**Under 5**						**All Age**					
	**Deaths**		**Incidence**		**Percent Change 1990-2019**		**Deaths**		**Incidence**		**Percent Change 1990-2019**	
**Aetiology**	**1990**	**2019**	**1990**	**2019**	**Deaths**	**Cases**	**1990**	**2019**	**1990**	**2019**	**Deaths**	**Cases**
**Total Meningitis**	**103,000 (81,500 to 132,000)**	**70,600 (52,700 to 93,200)**	**675,000 (517,000 to 884,000)**	**637,000 (480,000 to 843,000)**	**-0.31 (-0.48 to -0.10)**	**-0.06 (-0.08 to -0.03)**	**145,000 (119,000 to 176,000)**	**121,000 (98,600 to 149,000)**	**960,000 (791,000 to 1,170,000)**	**995,000 (825,000 to 1,210,000)**	**-0.16 (-0.32 to 0.04)**	**0.04 (0.01 to 0.06)**
E. coli	8,240 (5,960 to 11,300)	7,010 (4,910 to 9,720)	28,800 (20,100 to 40,400)	34,400 (24,100 to 47,600)	-0.14 (-0.36 to 0.13)	0.20 (0.09 to 0.32)	11,100 (8,310 to 14,800)	11,100 (8,410 to 14,700)	39,000 (28,500 to 52,000)	48,900 (36,400 to 63,800)	0.01 (-0.20 to 0.29)	0.26 (0.16 to 0.37)
Group B *Streptococcus*	8,340 (6,500 to 10,900)	7,140 (5,310 to 9,500)	47,400 (35,400 to 62,600)	57,300 (42,600 to 76,400)	-0.14 (-0.35 to 0.12)	0.21 (0.12 to 0.30)	10,300 (8,350 to 13,100)	10,200 (8,030 to 12,900)	59,600 (46,000 to 75,000)	76,100 (59,700 to 96,000)	-0.01 (-0.22 to 0.24)	0.28 (0.20 to 0.36)
H. influenzae	11,400 (8,670 to 14,800)	5,040 (3,660 to 6,710)	116,000 (87,000 to 152,000)	69,600 (52,200 to 93,000)	-0.55 (-0.67 to -0.41)	-0.40 (-0.44 to -0.35)	13,000 (10,100 to 16,500)	6,410 (4,930 to 8,240)	132,000 (102,000 to 168,000)	84,100 (66,100 to 107,000)	-0.50 (-0.62 to -0.36)	-0.36 (-0.40 to -0.32)
K. pneumoniae	10,500 (7,280 to 14,800)	8,470 (5,720 to 11,800)	22,300 (14,800 to 33,200)	24,300 (16,500 to 34,800)	-0.19 (-0.40 to 0.07)	0.09 (-0.01 to 0.21)	15,100 (11,100 to 20,100)	14,700 (10,800 to 19,300)	32,800 (23,300 to 45,300)	38,400 (28,600 to 50,600)	-0.02 (-0.22 to 0.22)	0.18 (0.08 to 0.29)
L. monocytogenes	4,000 (2,690 to 5,720)	3,450 (2,260 to 4,990)	11,000 (7,060 to 16,400)	13,100 (8,460 to 19,700)	-0.13 (-0.35 to 0.15)	0.20 (0.07 to 0.34)	5,780 (4,170 to 7,730)	6,050 (4,460 to 7,990)	15,900 (11,200 to 22,000)	20,200 (14,700 to 27,900)	0.05 (-0.16 to 0.33)	0.28 (0.16 to 0.41)
N. meningitidis	20,000 (15,300 to 25,900)	8,300 (6,100 to 11,400)	161,000 (121,000 to 210,000)	98,700 (73,400 to 132,000)	-0.58 (-0.69 to -0.43)	-0.39 (-0.44 to -0.34)	28,600 (23,100 to 35,000)	15,300 (12,200 to 19,000)	234,000 (191,000 to 286,000)	161,000 (131,000 to 197,000)	-0.46 (-0.57 to -0.32)	-0.31 (-0.37 to -0.26)
Other	8,710 (5,690 to 12,800)	7,170 (4,540 to 10,900)	50,800 (31,500 to 76,000)	58,400 (36,500 to 87,600)	-0.17 (-0.39 to 0.11)	0.15 (0.03 to 0.29)	13,100 (9,420 to 17,800)	13,100 (9,660 to 17,800)	75,600 (54,000 to 103,000)	92,800 (68,400 to 124,000)	0.01 (-0.21 to 0.28)	0.23 (0.11 to 0.37)

S. aureus	5,260 (3,930 to 6,920)	4,480 (3,160 to 6,130)	27,300 (20,100 to 36,200)	32,900 (23,900 to 43,900)	-0.14 (-0.37 to 0.14)	0.21 (0.10 to 0.32)	7,850 (6,260 to 9,870)	8,150 (6,310 to 10,400)	40,600 (32,500 to 50,200)	52,100 (42,100 to 64,300)	0.04 (-0.16 to 0.30)	0.28 (0.19 to 0.38)
S. pneumoniae	17,100 (13,100 to 22,200)	12,500 (9,150 to 17,000)	64,400 (48,100 to 83,800)	86,300 (64,300 to 116,000)	-0.26 (-0.45 to -0.02)	0.34 (0.26 to 0.43)	25,100 (20,500 to 30,900)	22,500 (17,900 to 28,100)	93,900 (76,200 to 114,000)	136,000 (112,000 to 168,000)	-0.10 (-0.28 to 0.11)	0.45 (0.37 to 0.54)
Virus	9,850 (7,740 to 12,600)	7,060 (5,260 to 9,420)	147,000 (111,000 to 192,000)	162,000 (121,000 to 216,000)	-0.28 (-0.45 to -0.05)	0.10 (0.06 to 0.15)	15,500 (12,900 to 18,500)	14,000 (11,400 to 17,200)	237,000 (197,000 to 283,000)	286,000 (239,000 to 342,000)	-0.09 (-0.26 to 0.11)	0.21 (0.17 to 0.25)

Appendix Table S5: Proportions of global meningitis cases and deaths occurring in the meningitis belt, 1990 and 2019, children under 5 and all ages

	Under 5 Proportions (%)				All Age Proportions (%)			
	1990		2019		1990		2019	
	Deaths	Cases	Deaths	Cases	Deaths	Cases	Deaths	Cases
Proportion of Global Meningitis	36.3 (32.6 to 39.9)	34.3 (32.5 to 35.8)	62.9 (57.5 to 67.8)	50.0 (48.1 to 51.7)	33.6 (30.9 to 36.4)	29.2 (27.6 to 30.8)	51.4 (47.1 to 55.2)	39.7 (37.4 to 41.8)

Appendix Table S6: Aetiology proportions (%) and mortality rates (per 100,000 population) in the meningitis belt, 1990 and 2019, children under 5 and all ages

	**Under 5**					**All Age**				
	**Mortality Rate**			**Proportion**		**Mortality Rate**			**Proportion**	
**Aetiology**	**1990**	**2019**	**Percent Change 1990-2019**	**1990**	**2019**	**1990**	**2019**	**Percent Change 1990-2019**	**1990**	**2019**
E. coli	11.3 (8.2 to 15.4)	5.1 (3.6 to 7.1)	-54.0 (-65.9 to -39.4)	8.0 (6.4 to 9.9)	9.9 (8.2 to 11.9)	2.9 (2.1 to 3.8)	1.3 (1.0 to 1.7)	-55.2 (-64.6 to -42.7)	7.7 (6.1 to 9.4)	9.2 (7.6 to 10.9)
Group B *Streptococcus*	11.4 (8.9 to 14.9)	5.2 (3.9 to 7.0)	-53.9 (-65.4 to -40.3)	8.1 (7.2 to 9.0)	10.1 (9.1 to 11.2)	2.7 (2.2 to 3.4)	1.2 (0.9 to 1.5)	-55.9 (-65.2 to -45.0)	7.1 (6.3 to 7.9)	8.4 (7.6 to 9.3)
H. influenzae	15.6 (11.9 to 20.2)	3.7 (2.7 to 4.9)	-76.1 (-82.2 to -68.3)	11.0 (10.1 to 12.0)	7.1 (6.5 to 7.8)	3.4 (2.6 to 4.3)	0.7 (0.6 to 0.9)	-77.9 (-83.1 to -71.5)	8.9 (8.2 to 9.7)	5.3 (4.8 to 5.7)
K. pneumoniae	14.4 (10.0 to 20.2)	6.2 (4.2 to 8.7)	-56.6 (-67.7 to -42.4)	10.2 (7.9 to 13.2)	12.0 (9.5 to 14.9)	3.9 (2.9 to 5.2)	1.7 (1.2 to 2.2)	-56.5 (-65.5 to -46.0)	10.4 (8.1 to 13.1)	12.1 (9.9 to 14.6)
L. monocytogenes	5.5 (3.7 to 7.8)	2.5 (1.7 to 3.7)	-53.5 (-65.3 to -38.3)	3.9 (2.9 to 5.1)	4.9 (3.7 to 6.4)	1.5 (1.1 to 2.0)	0.7 (0.5 to 0.9)	-53.2 (-62.7 to -40.7)	4.0 (3.0 to 5.1)	5.0 (4.0 to 6.2)
N. meningitidis	27.3 (20.9 to 35.5)	6.1 (4.5 to 8.4)	-77.6 (-83.2 to -69.7)	19.3 (17.9 to 20.7)	11.7 (10.5 to 13.1)	7.4 (6.0 to 9.0)	1.7 (1.4 to 2.2)	-76.2 (-81.1 to -69.7)	19.7 (18.4 to 20.9)	12.6 (11.4 to 13.8)
Other	11.9 (7.8 to 17.5)	5.3 (3.3 to 8.0)	-55.5 (-67.5 to -40.5)	8.4 (5.8 to 11.6)	10.1 (7.2 to 13.9)	3.4 (2.4 to 4.6)	1.5 (1.1 to 2.0)	-55.0 (-64.8 to -43.2)	9.0 (6.9 to 11.7)	10.8 (8.7 to 13.5)
S. aureus	7.2 (5.4 to 9.5)	3.3 (2.3 to 4.5)	-54.0 (-66.0 to -38.9)	5.1 (4.4 to 5.9)	6.3 (5.5 to 7.3)	2.0 (1.6 to 2.5)	0.9 (0.7 to 1.2)	-53.6 (-62.7 to -42.2)	5.4 (4.7 to 6.2)	6.7 (5.9 to 7.6)
S.pneumoniae	23.4 (17.9 to 30.4)	9.2 (6.7 to 12.5)	-60.5 (-70.8 to -47.6)	16.5 (15.3 to 17.8)	17.7 (16.2 to 19.2)	6.5 (5.3 to 8.0)	2.6 (2.1 to 3.2)	-60.0 (-68.0 to -50.6)	17.3 (16.1 to 18.4)	18.5 (17.2 to 19.8)
Virus	13.5 (10.6 to 17.3)	5.2 (3.9 to 6.9)	-61.3 (-70.6 to -49.3)	9.5 (9.0 to 10.2)	10.0 (9.4 to 10.7)	4.0 (3.3 to 4.8)	1.6 (1.3 to 2.0)	-59.7 (-67.3 to -50.5)	10.6 (10.1 to 11.2)	11.5 (10.9 to 12.1)
